# Novel Bis-Ammonium Salts of Pyridoxine: Synthesis and Antimicrobial Properties

**DOI:** 10.3390/molecules25184341

**Published:** 2020-09-22

**Authors:** Nikita V. Shtyrlin, Mikhail V. Pugachev, Sergey V. Sapozhnikov, Marsel R. Garipov, Rusalia M. Vafina, Denis Y. Grishaev, Roman S. Pavelyev, Renata R. Kazakova, Mariya N. Agafonova, Alfiya G. Iksanova, Svetlana A. Lisovskaya, Marina I. Zeldi, Elena S. Krylova, Elena V. Nikitina, Alina E. Sabirova, Airat R. Kayumov, Yurii G. Shtyrlin

**Affiliations:** 1 Kazan (Volga region) Federal University, Scientific and Educational Center of Pharmaceutics, Kremlyovskaya St. 18, Kazan 420008, Russia; Nikita.Shtyrlin@kpfu.ru (N.V.S.); pugachev.mihail@gmail.com (M.V.P.); sapozhnikovsergei@gmail.com (S.V.S.); MarRGaripov@kpfu.ru (M.R.G.); rusalia.wafina@kpfu.ru (R.M.V.); DJGrishaev@kpfu.ru (D.Y.G.); rpavelyev@gmail.com (R.S.P.); kazakova.renata@mail.ru (R.R.K.); Mariya.Agafonova@kpfu.ru (M.N.A.); Alfija.Iksanova@kpfu.ru (A.G.I.); zeldimarina@gmail.com (M.I.Z.); elena_krylova.stud2015@mail.ru (E.S.K.); ev-nikitina@inbox.ru (E.V.N.); alinka.zam@mail.ru (A.E.S.); Ajrat.Kajumov@kpfu.ru (A.R.K.); 2 Kazan Scientific Research Institute of Epidemiology and Microbiology, Kazan 420015, Russia; s_lisovskaya@mail.ru

**Keywords:** quaternary ammonium salts, pyridoxine, antibacterial activity, antifungal activity, cytotoxicity, biofilms, antiseptics

## Abstract

A series of 108 novel quaternary bis-ammonium pyridoxine derivatives carrying various substituents at the quaternary nitrogen’s and acetal carbon was synthesized. Thirteen compounds exhibited antibacterial and antifungal activity (minimum inhibitory concentration (MIC) 0.25–16 µg/mL) comparable or superior than miramistin, benzalkonium chloride, and chlorhexidine. A strong correlation between the lipophilicity and antibacterial activity was found. The most active compounds had logP values in the range of 1–3, while compounds with logP > 6 and logP < 0 were almost inactive. All active compounds demonstrated cytotoxicity comparable with miramistin and chlorhexidine on HEK-293 cells and were three-fold less toxic when compared to benzalkonium chloride. The antibacterial activity of leading compound **5c_12_** on biofilm-embedded *Staphylococcus aureus*, *Staphylococcus epidermidis*, *Escherichia coli* or *Pseudomonas aeruginosa* was comparable or even higher than that of the benzalkonium chloride. In vivo **5c_12_** was considerably less toxic (LD_50_ 1705 mg/kg) than benzalkonium chloride, miramistine, and chlorhexidine at oral administration on CD-1 mice. An aqueous solution of **5c_12_** (0.2%) was shown to be comparable to reference drugs efficiency on the rat’s skin model. The molecular target of **5c_12_** seems to be a cellular membrane as other quaternary ammonium salts. The obtained results make the described quaternary bis-ammonium pyridoxine derivatives promising and lead molecules in the development of the new antiseptics with a broad spectrum of antimicrobial activity.

## 1. Introduction

The drastic worldwide rise of antimicrobial drug resistance would lead to the death of 10 million people every year by 2050, which becomes a proven and challenging problem [1,2]. In comparison with antibiotics for systemic application, antiseptics are characterized by a wider spectrum of antimicrobial action against bacteria, fungi, viruses, and protozoa [3,4]. Therefore, antiseptics are an important integral part of stewardship strategies for preventing and treating the surgical site and chronic open wound infections [5].

QAC (quaternary ammonium compounds) is one of the best-known group of antiseptics. The prominent representatives of this class are benzalkonium chloride, cetylpyridinium chloride, miramistin, and didecyldimethylammonium chloride [6,7]. The mechanism of the antibacterial action of QAC consists of their adsorption and penetration through the cell wall of bacteria, which is followed by an interaction with phospholipids of the cytoplasmic membrane and subsequent complete structural disorganization of the membrane and subsequent cell death [3].

Since QAC-based antiseptics could be inefficient because of acquired resistance [3,8], have high toxicity [9], lead to skin irritation [10], and demonstrate low activity against spores, simple viruses, Gram-negative bacteria, mycobacteria, and fungi [11], the development of new antiseptic molecules is strictly required. Therefore, the goal of this study was developing new drugs that would strengthen all the advantages of QAC and eliminate their disadvantages as well.

**Figure 1:**
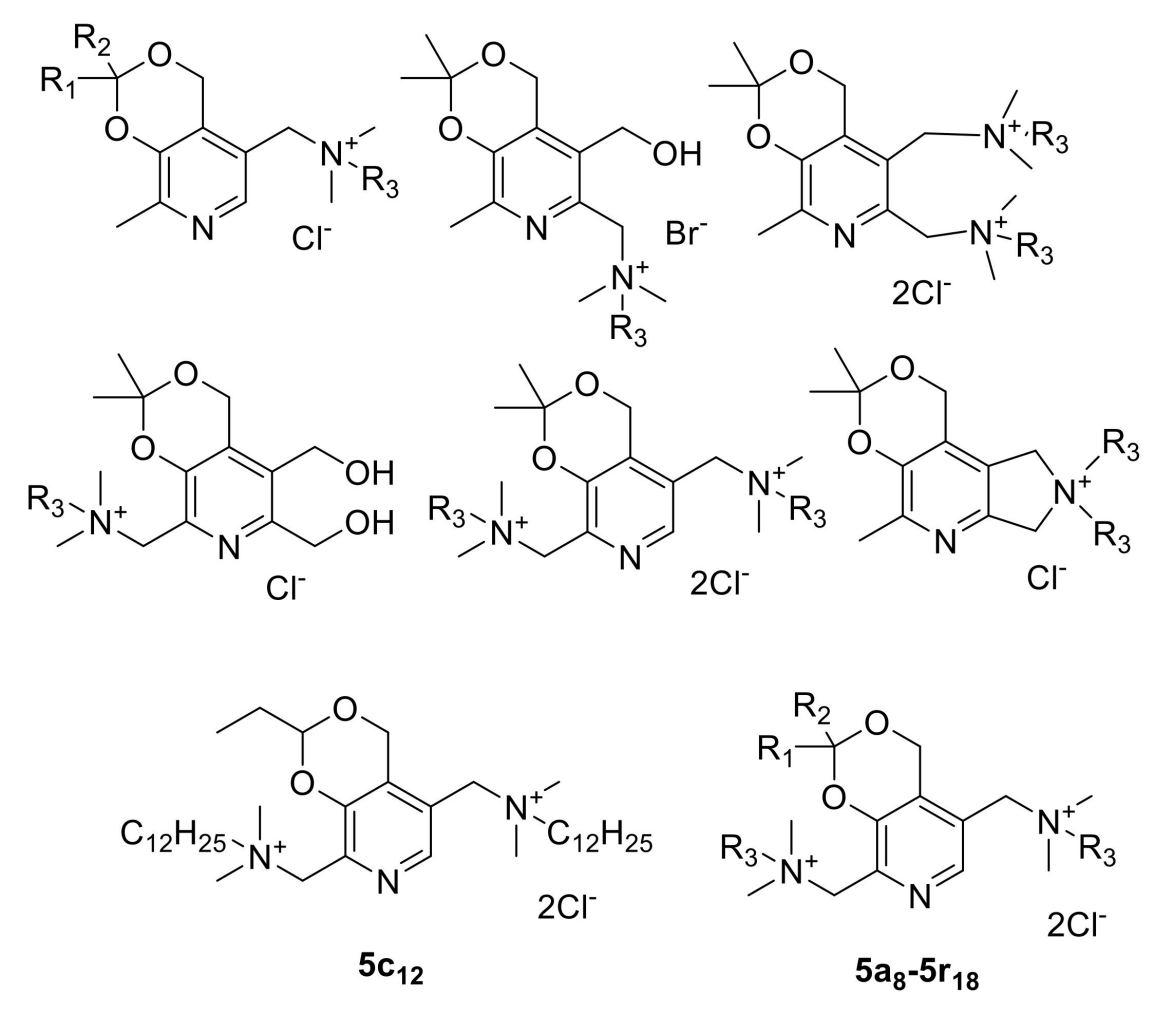
The structures of QAC based on pyridoxine derivatives obtained by us earlier [13,14,15,16,17,18,19] and compounds **5a_8_**–**5r_18_** synthesized in this work.

In our group, we have systematically studied chemistry and biological properties of the pyridoxine (vitamin B6) derivatives [12]. In previous works, we described a series of QAC based on pyridoxine derivatives [13,14,15,16,17]. Some of them possess high antibacterial activity against various Gram-positive and Gram-negative bacteria including multi-resistant clinical isolates (MICs = 0.5–16 µg/mL) and low toxicity in vitro. In our patents [18,19], we described the synthesis of the bis-ammonium salt **5c_12_** with advantageous antimicrobial properties (Figure 1). These promising results encouraged us to synthesize and study an extended set of modified structural analogs of **5c_12_**, which bears various substituents at the quaternary nitrogens and acetal carbon.

## 2. Results and Discussion

**Scheme 1:**
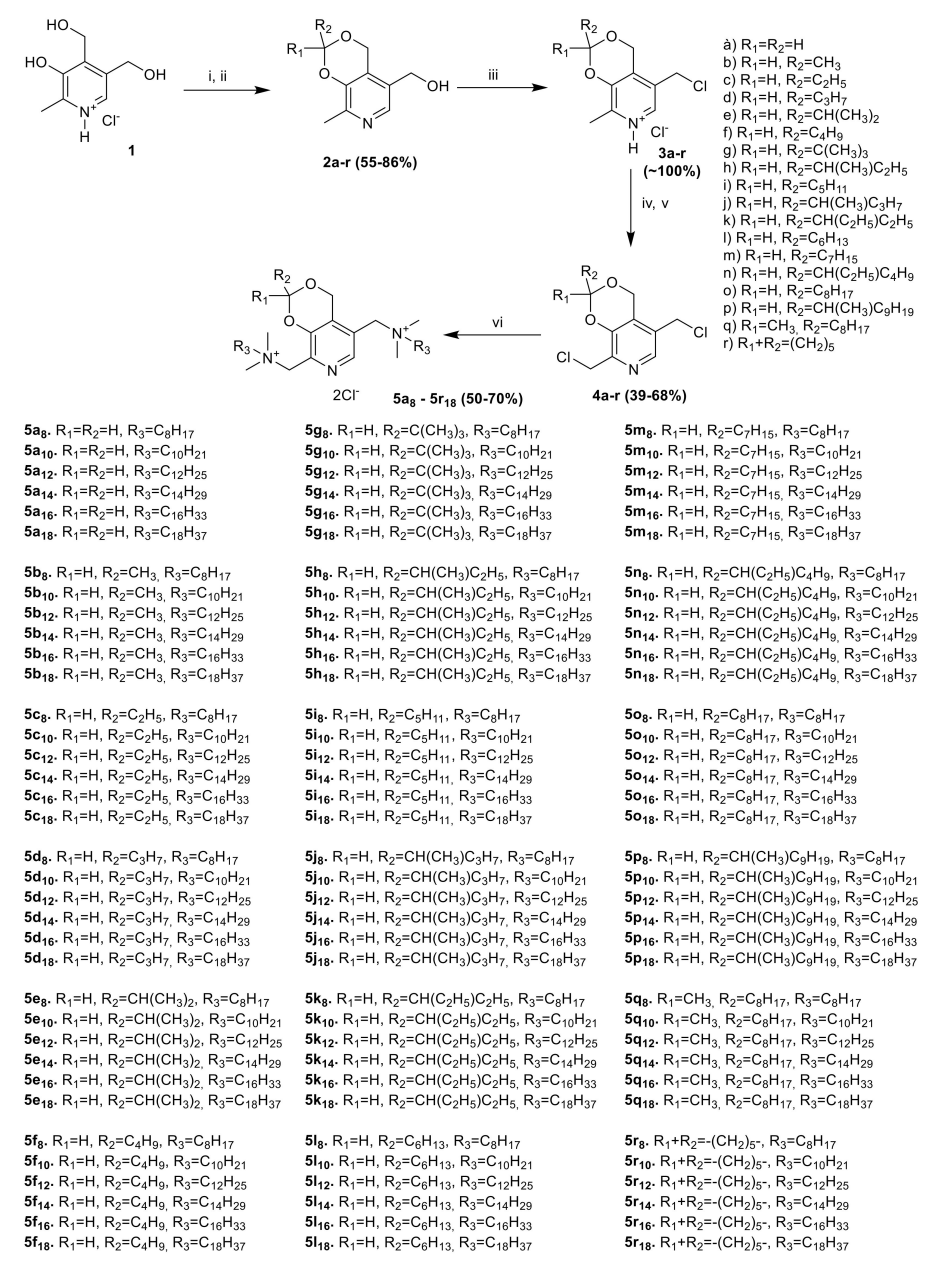
Reagents and conditions. (i) R_1_C(O)R_2_, HCl, 3–5 °C, 3 h; or C_6_H_6_, R_1_C(O)R_2_, *p*-TsOH, reflux, 17 h, (ii) H_2_O_,_ NaOH, 20 °C, (iii) CH_2_Cl_2_, SOCl_2_, 20 °C, 17 h, (iv) H_2_O/CHCl_3,_ NaHCO_3_, (v) CHCl_3_, TCICA, reflux, 4 h, (vi) C_2_H_5_OH, R_3_N(CH_3_)_2_, 70 °C, 5 h.

In the work [18], we described the first synthesis of compound **5c_12_** in seven steps with the overall yield of only 13%. Then we developed a new synthetic approach in four steps with an overall yield of 22% [19]. For the synthesis of its analogues **5a_8_**–**5r_18_**, we used the same approach with some modifications (Scheme 1). An approach includes the initial preparation of six-membered acetals **2a**–**r** with different substituents at acetal carbon [20,21]. At the second stage, in reaction of **2a**–**r** with thionyl chloride in chloroform led to the corresponding chlorides **3a**–**r** with quantitative yields. Then, via chlorination of aromatic methyl group with trichloroisocyanuric acid (TCICA) in chloroform dichloro derivatives **4a**–**r** were obtained. The use of other chlorinating agents (*N*-chlorosuccinimide, sulfuryl chloride), varying solvents (methylene chloride, tetrachloroethane), and radical reactions initiators (AIBN, ACHN, benzoyl peroxide) did not lead to desired products. At the last stage, in reaction of dichlorides **4a**–**r** with a two-fold molar excess of tertiary amines (*N*,*N*-dimethyoctylamine, *N*,*N*-dimethyldecylamine, *N*,*N*-dimethyldodecylamine, *N*,*N*-dimethyltetradecylamine, *N*,*N*-dimethylhexadecylamine, *N*,*N*-dimethyloctadecylamine) in ethanol at 70 °C 108 bis-ammonium compounds **5a_8_**–**5r_18_** were obtained. The substituent’s variations allowed varying compound’s lipophilicity and, thus, investigate the lipophilicity-activity relationships.

### 2.1. Antibacterial Activity

The antibacterial activity of 108 newly synthesized compounds was evaluated against three Gram-positive and three Gram-negative bacterial strains. Table 1 shows MICs of compounds in comparison with miramistin, benzalkonium chloride, and chlorhexidine. The lipophilicity of the synthesized ammonium salts was expressed in terms of their partition coefficient values (logP) calculated using online platform Chemicalize (ChemAxon) [20].

All compounds with tetradecyl, hexadecyl, and octadecyl substituents at the quaternary nitrogens were almost inactive regardless of the substituent on the acetal carbon atom. For compounds with small substituents at acetal carbon (H, CH_3_, C_2_H_5_, C_3_H_7_, CH(CH_3_)_2_) against all bacterial strains, the most active were compounds with decyl substituents at quaternary nitrogens (**5a_10_**, **5b_10_**, **5c_10_**, **5d_10_**, **5e_10_**, MICs 0.5–8 µg/mL on Gram-positive and 2–64 µg/mL on Gram-negative strains). Compounds with dodecyl substituents were slightly less active (**5a_12_**, **5b_12_**, **5c_12_**, **5d_12_**, and **5e_12_**) and compounds with octyl substituents (**5a_8_**, **5b_8_**, **5c_8_**, **5d_8_**, and **5e_8_**) were inactive.

Due to an increase of the length of substituents on acetal carbon (C_4_H_9_, C(CH_3_)_3_, CH(CH_3_)C_2_H_5_, C_5_H_11_, and CH(C_2_H_5_)C_2_H_5_, C_6_H_13_), the most active were compounds with decyl substituents at quaternary nitrogens (**5f_10_**, **5g_10_**, **5h_10_**, **5i_10_**, **5j_10_, 5k_10_**, **5l_10_**, MICs 0.25–4 µg/mL on Gram-positive and 0.5–8 µg/mL on Gram-negative strains). Compounds with octyl and dodecyl substituents had similar activity and were less active than compounds with decyl substituents. A similar activity pattern was observed for compounds with a cyclohexyl substitutent at acetal carbon (**5r_8_–5r_12_**).

With the further increase in the length of the substituents at the acetal carbon (C_7_H_15_, CH(C_2_H_5_)C_4_H_9_, C_8_H_17_, CH(CH_3_)C_9_H_19_), compounds with octyl substituents at quaternary nitrogens (**5m_8_**, **5n_8_**, **5o_8_**, **5p_8_**, **5q_8_**) became the most active (MICs 0.5–2 µg/mL on Gram-positive and 1–16 µg/mL on Gram-negative strains). Compounds with decyl substituents were slightly less active (**5m_10_**, **5n_10_**, **5o_10_**, **5p_10_**, and **5q_10_**) and compounds with dodecyl substituents were almost inactive.

Based on the results of the primary screening, 13 compounds (**5a_12_, 5c_10_, 5c_12_, 5d_10_, 5f_10_, 5g_10_**, **5i_10_, 5l_10_, 5m_8_, 5n_10_, 5o_8_, 5p_8_**, and **5q_8_** in bold in Table 1) with an activity similar or superior when compared reference drugs were selected for further in-depth investigation for antibacterial activity, antifungal activity, and cytotoxicity in vitro.

The results of investigation of antibacterial activity on clinical Gram-positive and Gram-negative bacterial isolates is presented in Table 2. The most active were **5c_12_**, **5i_10_**, **5l_10_**, **5n_10_**, **5o_8_**, **5p_8_**, and **5q_8_**, whose activity significantly exceeded that of all reference drugs. Compounds **5a_12_**, **5f_10_**, **5g_10_**, and **5m_8_** exhibited, in general, antibacterial activity comparable to that of benzalkonium chloride and significantly exceeded miramistin and chlorhexidine in activity. Compounds **5c_10_** and **5d_10_** were almost inactive on all strains besides *S. aureus* 25 and *S. intermedius* 1143 MRSI (methicillin-resistant *Staphylococcus intermedius).*

### 2.2. Structure-Activity Relationship (SAR)

**Figure 2:**
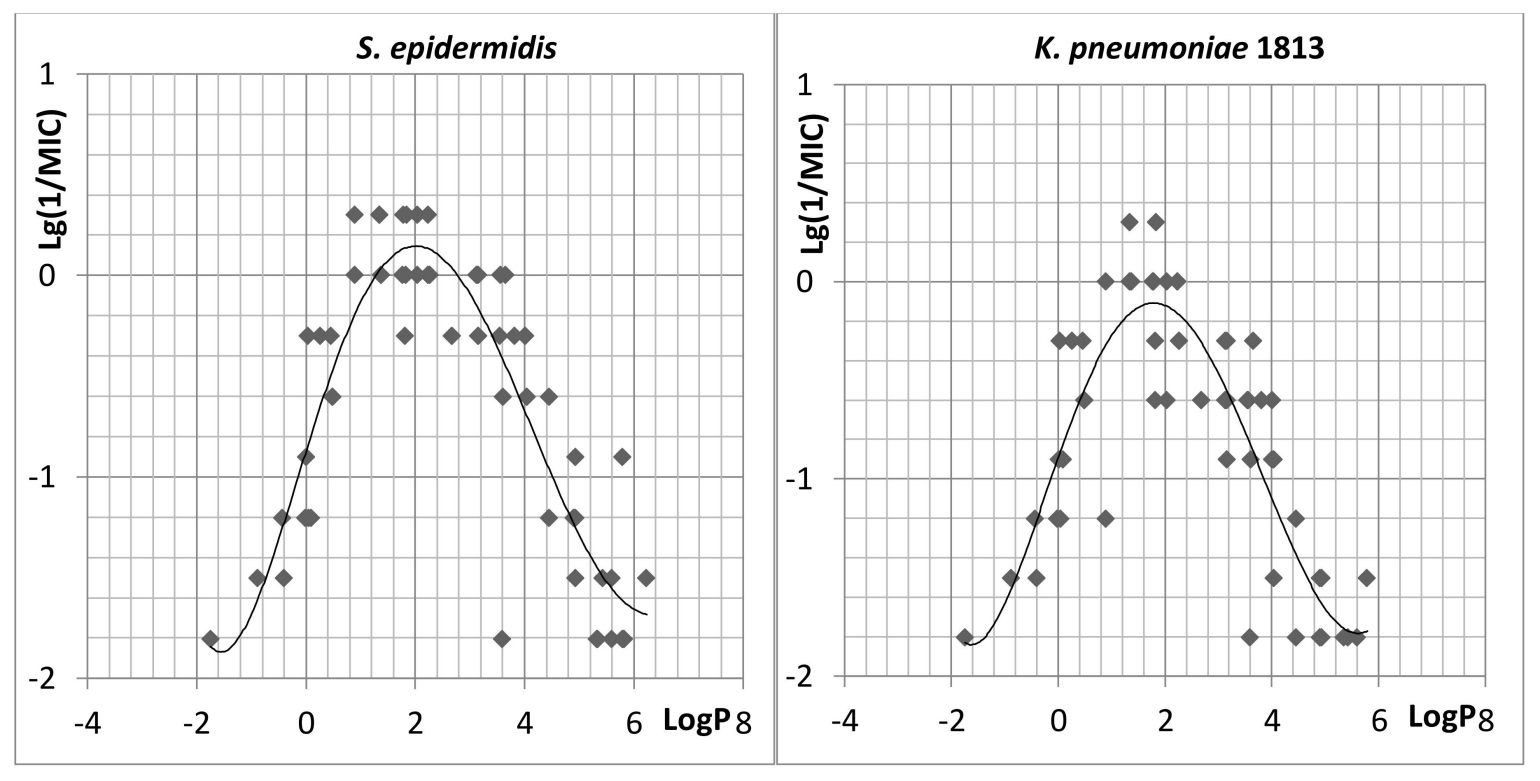
Lg1/MIC versus calculated logP relationship for novel bis-ammonium salts of pyridoxine. The parabolic line shows the polynomial approximation by using a second order model. Inactive compounds (MIC > 64 mg/mL) are not shown.

To identify quantitative relationships “structure–antibacterial activity” in a series of synthesized QACs based on vitamin B6 derivatives **5a_8_**–**5r_18_**, the dependences of antibacterial activity on calculated in ChemAxon lipophilicity [22] were evaluated (Figure 2, for *S. epidermidis* and *K. pneumoniae* 1813). A strong correlation between the lipophilicity and antimicrobial activity of quaternary ammonium pyridoxine derivatives on *S. epidermidis* and *K. pneumonia* was observed. Thus, all the most active compounds had logP values in the range of 1–3, while compounds with logP > 6 and logP < 0 were almost inactive. Similar relationships were found for the other studied bacteria. Apparently, relationships reflect the important features of the active compounds essential for their effective interaction with the hydrophobic membrane core of bacterial cells.

### 2.3. Antifungal Activity

The antifungal activity is an important characteristic of antiseptics. Therefore, the effect of **5a_12_, 5c_10_, 5c_12_, 5d_10_, 5f_10_, 5g_10_**, **5i_10_, 5l_10_, 5m_8_, 5n_10_, 5o_8_, 5p_8_**, and **5q_8_** on growth of various fungal pathogens has been evaluated (Table 3).

Compound **5c_10_, 5c_12_**, **5d_10_**, **5f_10_**, **5n_10_**, and **5p_8_** exhibited a pronounced antifungal activity against all tested fungi with minimum inhibitory concentrations (MIC) lower than all reference drugs. The highest activity demonstrated compound **5d_10_** with MIC values of 2–4 µg/mL against all tested fungi. Compounds **5a_12_**, **5g_10_**, **5i_10_**, **5l_10_**, **5o_8_**, and **5q_8_** had a comparable activity with miramistin. Compound **5m_8_** was almost inactive on all tested fungi similar to chlorhexidine.

### 2.4. Cytotoxicity

Cytotoxicity of compounds **5a_12_**, **5c_10_**, **5c_12_**, **5d_10_**, **5f_10_**, **5g_10_**, **5i_10_**, **5l_10_**, **5m_8_**, **5n_10_**, **5o_8_**, **5p_8_**, and **5q_8_** was evaluated on HEK-293 cells (Table 4). According to the results obtained, all compounds had values of cytotoxicity comparable with miramistin and chlorhexidine and were 2–4-fold less toxic when compared to benzalkonium chloride.

### 2.5. In Vivo Toxicity

For the lead antibacterial and antifungal compounds **5c_12_**, **5d_10_**, and **5p_8_** in vivo toxicity at oral administration on CD-1 mice were performed as described in Section 3.5 (Table 5). No remarkable findings were noted in both sexes in compound **5c_12_** treatment group at necropsy. Compounds **5d_10_** and **5p_8_** treatment groups revealed stomach walls hypertrophy. Thus, all studied substances belong to category 4 of the globally harmonized system of classification and labeling of chemicals [23]. Compound **5c_12_** was considerably less toxic (LD_50_ 1705 mg/kg) than benzalkonium chloride (LD_50_ 180 mg/kg [24]), miramistine (LD_50_ 1000 mg/kg [25]), and chlorhexidine (LD_50_ 1260 mg/kg) [26]). Compounds **5d_10_** and **5p_8_** are 2-fold and 4-fold more toxic when compared to **5c_12_**.

Thus, the extensive screening of the antibacterial activity and toxicity of the modified structural analogs of compound **5c_12_** did not reveal compounds superior in antibacterial activity. In addition, its in vivo toxicity was significantly lower than that of compounds with comparable activity. For this reason, **5c_12_** was selected as a lead compound for further investigations.

### 2.6. Anti-Biofilm Activity

The anti-biofilm activity of **5c_12_** has been tested on biofilms formed by *S. aureus*, *S. epidermidis*, *E. coli*, or *P. aeruginosa*. For that, 48-h-old bacterial biofilms prepared in 24-well plates were washed with sterile phosphate-buffered saline (PBS) and filled with 125 μL of fresh basal medium (BM) broth containing **5c_12_**, miramistin, or benzalkonium chloride in concentrations of their respective 8–16 × MBC (minimum bactericidal concentration, see Table 6 for values). After 24 h of incubation, the viability of biofilm-embedded cells was evaluated by colony-forming units (CFUs) counting.

**Figure 3:**
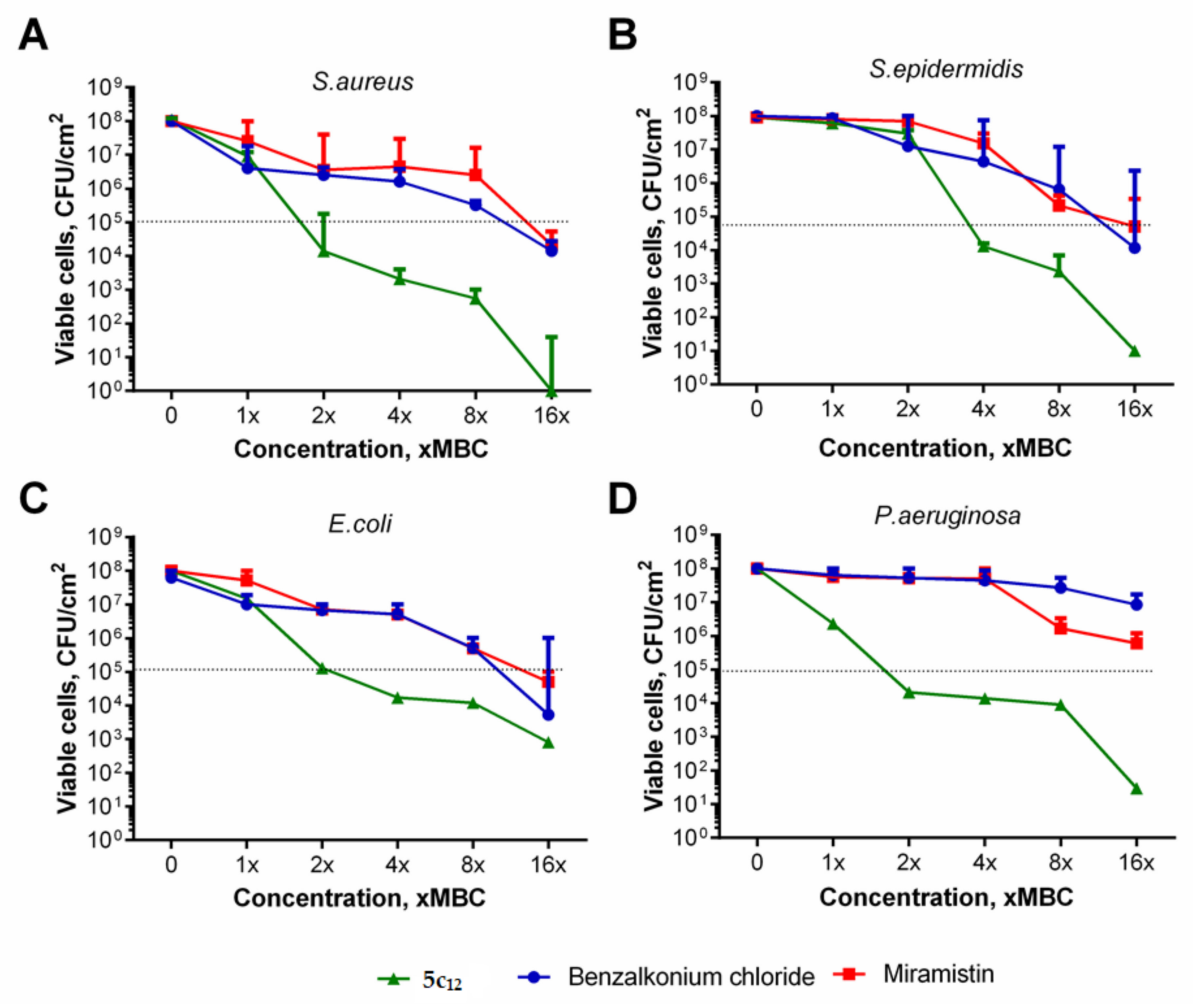
The effect of **5c_12_** and reference antiseptics on biofilm-embedded bacteria. (**A**)—*S. aureus*; (**B**)—*S. intermedius;* (**C**)—*E. coli*, (**D**)—*P. aeruginosa*. The 48-h biofilms were treated with 1–16 × MBCs of benzalkonium chloride (circles), miramistin (squares), or **5c_12_** (triangles) for 24 h and then quantified by CFU counting. Data are present as medians from five independent experiments with interquartile range (M ± IQR). Dotted line shows 3 log-decrease of viable cells.

In these experiments, **5c_12_** exhibited higher activity compared with the reference antimicrobials: a 3-log drop of CFUs of these bacteria was observed at 2 × MBC of **5c_12_** (Figure 3), while 8 × MBCs of both benzalkonium chloride and miramistin were required to obtain the same effect. Moreover, both reference antimicrobials were less active against *P. aeruginosa* biofilm even at 16 × MBC, while **5c_12_** reduced the CFUs number by three orders of magnitude at 8 × MBC (Figure 3D). Thus, **5c_12_** demonstrated anti-biofilm activity comparable or even higher than that of the benzalkonium chloride.

### 2.7. In Vivo Efficiency

In vivo efficiency of **5c_12_** was estimated in a rat skin model in comparison with miramistin, benzalkonium chloride and chlorhexidine. Aqueous solution of 0.2% **5c_12_** reduced CFU (colony-forming unit) of *E. coli* CD CF-50 after 5 min of exposure on rat’s skin (Table 7).

**Figure 4:**
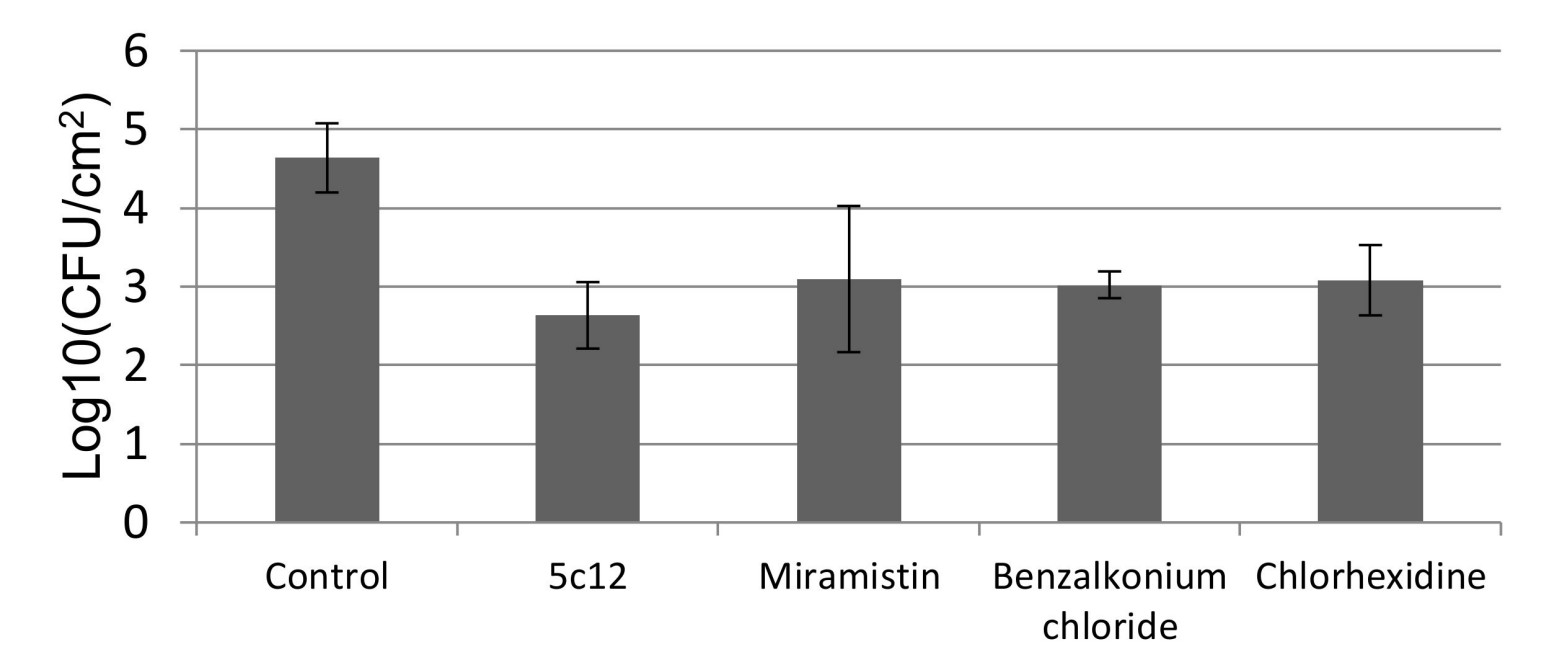
In vivo antibacterial efficacy test (Mean ± SD) using a rat skin model. log10 number of viable cells in the control and the number of viable cells after exposure with **5c_12_**, miramistin, benzalkonium chloride, and chlorhexidine, *n* = 6.

Compound **5c_12_** demonstrated the strongest *E. coli* CD CF-50 growth inhibition (Figure 4). The growth inhibition percentage reached 97.5%, which is comparable to reference drugs’ efficiency [27].

### 2.8. Cytoplasmic Membrane Depolarization

**Figure 5:**
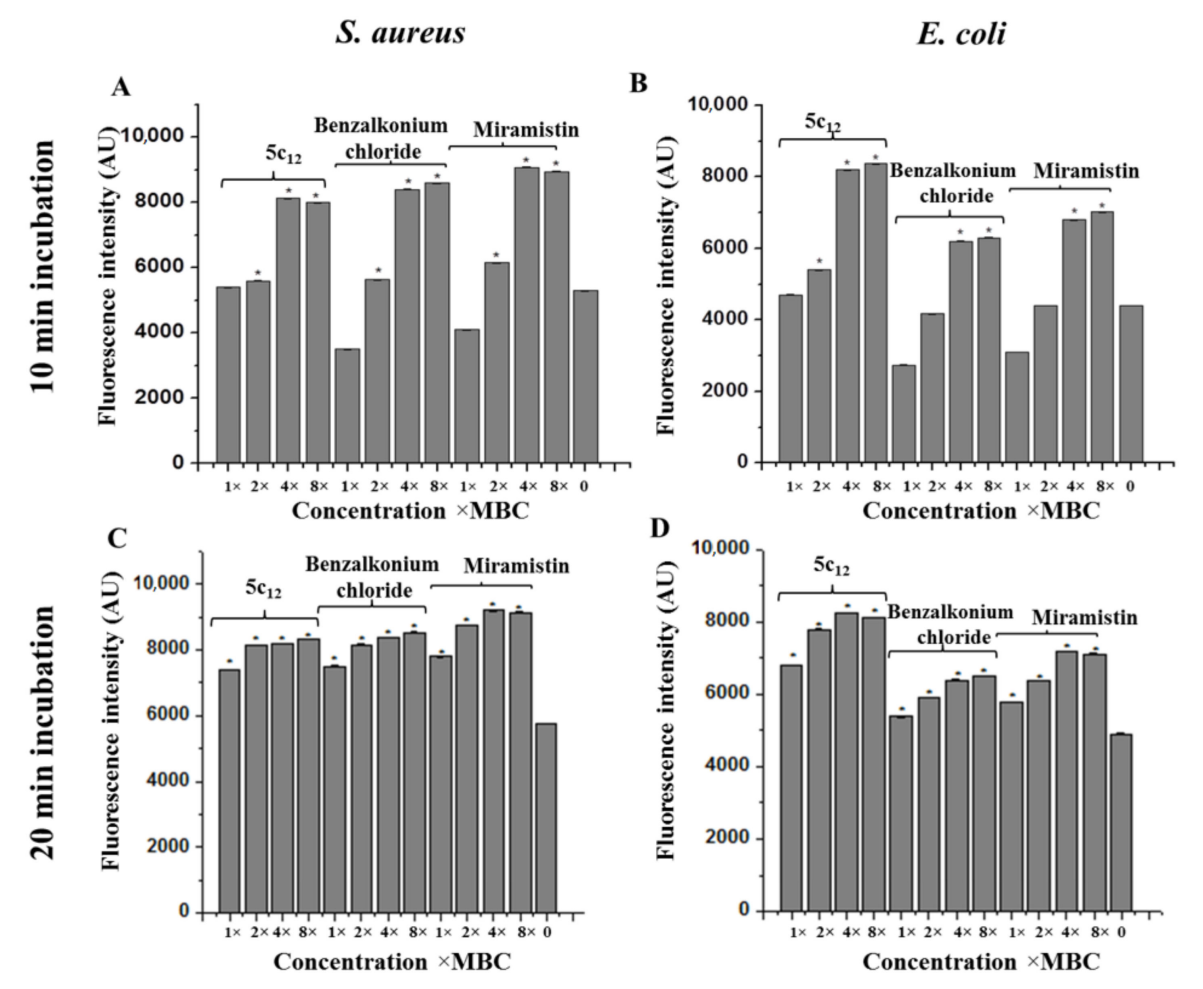
The effect of **5c_12_** on the electric potential of the bacterial membrane determined using a membrane-potential-sensitive cyanine dye DiSC_3_(5) (10 min treatment by 1–8 × MBC (**A**,**B**) and 20 min treatment by 1–8 × MBC (**C**,**D**)). Asterisks denote a statistically significant difference in comparison with untreated cells. * statistically significant difference from the control.

Quaternary ammonium salts are well known for their membrane-damaging action on bacterial cells [28]. To evaluate the effect of **5c_12_** on the membrane polarization, the fluorescence of membrane-potential-sensitive cyanine dye DiSC_3_(5) in suspensions of either *S. aureus* ATCC209p or *E. coli* CDCF-50 has been measured in the presence of **5c_12_** or either benzalkonium chloride or miramistin (Figure 5). In the presence of **5c_12_** and reference compounds, a rapid and intense increase of fluorescence of DiSC_3_(5) has been observed in both *S. aureus* and *E. coli* suspensions. It was shown that the ability of **5c_12_** to change the electric potential of the bacterial membrane was comparable to the reference compounds. Significant differences between untreated cells and treated by **5c_12_** (*p ≤* 0.05) cells were observed after 20 min of incubation (Figure 5C,D). When the **5c_12_** concentration increased from 1 × MBC to 2, 4, and 8 × MBC, the membrane-damaging effect has been achieved two times faster. We found that a 10-min incubation of cells with **5c_12_** led to significant differences in the control (Figure 5A,B).

### 2.9. Outer Membrane Permeability

Since the membrane depolarization can be driven with either membrane permeabilization or a metabolism drop, the ability of **5c_12_** to permeate the bacterial outer membrane was determined using the fluorescent dye *N*-phenyl-1-naphthylamine (NPN). A strong dose-dependent response of **5c_12_** on the Gram-negative bacteria outer membrane penetration was observed. For untreated *E. coli* cells, the permeability was 45%. In the presence of 1 × MBC of **5c_12_**, the outer membrane permeability increased to 70% with increasing concentrations (2 × and 4 × MBC) of **5c_12_**. The membrane permeability reached more than 80%.

### 2.10. Scanning Electron Microscopy

**Figure 6:**
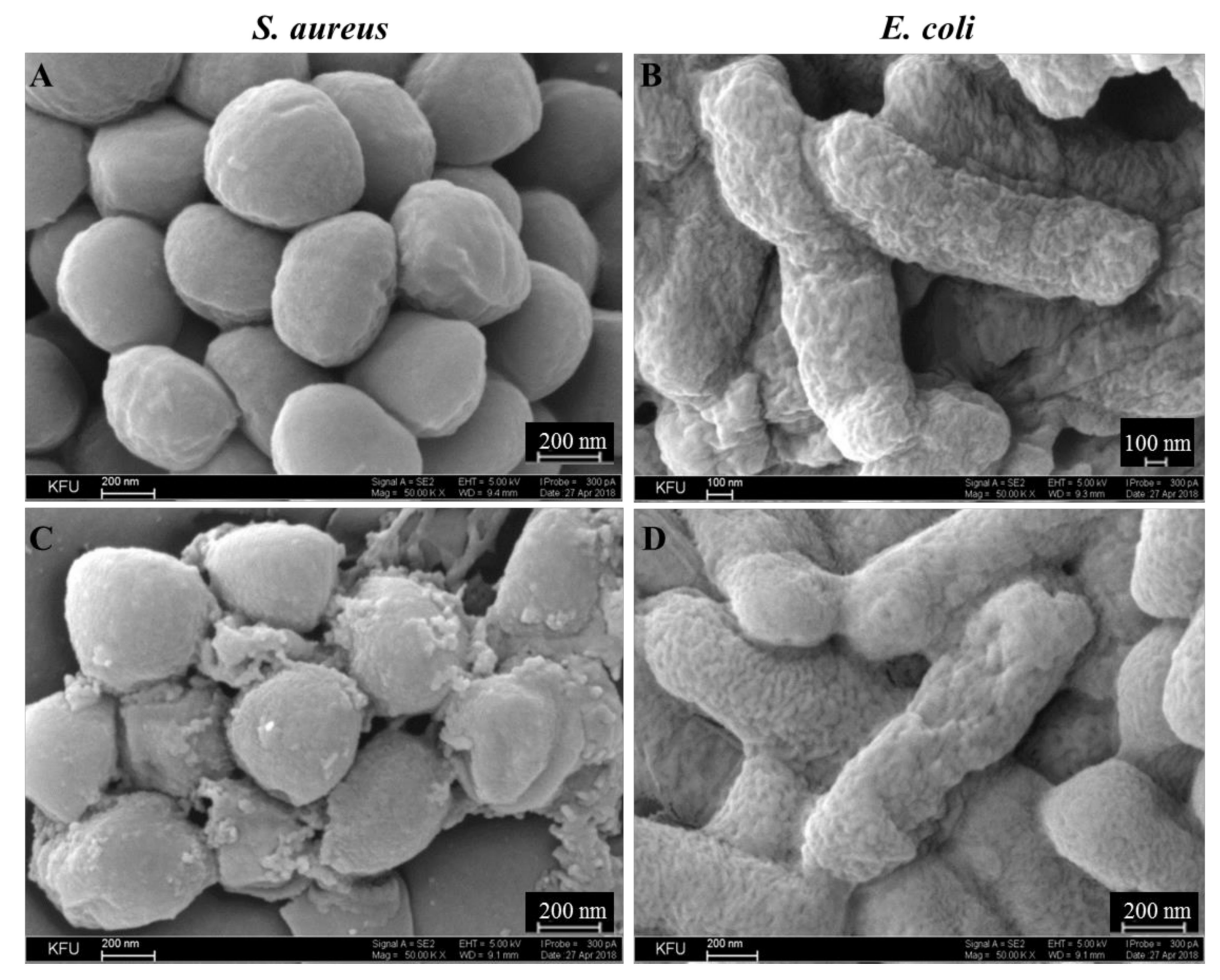
Scanning Electron Microscopy microphotographs of *S. aureus* ATCC209p (**A**,**C**) and *E. coli* CDC F-50 (**B**,**D**). Untreated cells (**A**,**B**) and bacteria treated by 0.2% solution of **5c_12_** for 30 min (**C**,**D**). 50,000× magnification.

The data presented above allow suggesting the destabilizing effect of **5c_12_** on the bacterial membrane, which leads to an antiseptic effect of the drug. These assumptions are confirmed by the SEM images of treated cells (Figure 6).

Thus, while the untreated *S. aureus* cells had a spherical regular shape with a dense surface with small gentle tubercles without vortices, dead cells were not detected in the visual fields (Figure 6A). After **5c_12_** treatment, dead cells with a shrunken cell membrane were observed. The shape of most cells became non-spherical and irregular with a losing surface and organic matter on most of the cells, possibly due to damage of the cytoplasmic membrane and leakage of internal contents (Figure 6C). A number of cells exhibited deep creases, which indicated the folding of the cell wall into the cell due to the outflow of cytoplasm. In contrast to *S. aureus*, untreated *E. coli* cells had a wrinkled surface (Figure 6B). After **5c_12_** treatment, the cell surface became more spongier due to this, which indicated the change in the structure of the cell wall (Figure 6D).

## 3. Materials and Methods

### 3.1. Chemistry

All reagents were obtained from commercial sources and were used without further purification unless otherwise stated. ^1^H and ^13^C NMR spectra were recorded on a ‘Bruker AVANCE 400′ at an operating frequency of 400 and 101.56 MHz, respectively. Chemical shifts were measured with reference to the residual protons of the solvent (CDCl_3_, ^1^H, 7.26 ppm, ^13^C, 77.16 ppm, DMSO-*d_6_*, ^1^H, 2.50 ppm, ^13^C, 39.51 ppm). Coupling constants (J) are given in Hertz (Hz). The following abbreviations are used to describe coupling: s = singlet, d = doublet, t = triplet, m = multiplet, br s = broad singlet, br m = broad multiplet, AB = AB system. Melting points were determined using a Stanford Research Systems MPA-100 OptiMelt melting point apparatus and are uncorrected. For TLC (thin-layer chromatography) analysis, silica gel plates from Sorbfil (Krasnodar, Russia) were used with UV light (254 nm/365 nm) or iron (III) chloride as a developing agent. Column chromatography was performed on silica gel (60–200 mesh) from Acros.

HRMS (high-resolution mass spectrometry) spectra were obtained on a quadrupole time-of-flight (t, qTOF) AB Sciex Triple TOF 5600 mass spectrometer using a turbo-ion spray source (nebulizer gas nitrogen, a positive ionization polarity, needle voltage 5500V). Recording of the spectra was performed in the “TOF MS” mode with collision energy 10 eV, declustering potential of 100 eV, and a resolution of more than 30,000 full-width half-maximum. Samples with the analyte concentration of 5 μmol/L were prepared by dissolving the test compounds in a mixture of methanol (HPLC-UV Grade, LabScan) and water (LC-MS Grade, Panreac) in a 1:1 ratio.

Methods of synthesis and analytical characteristics of all new synthesized compounds are described in the Electronic Supplementary Material.

### 3.2. Antibacterial Activity

The antibacterial activity of the obtained compounds was evaluated on a number of Gram-positive bacteria: *Staphylococcus aureus* ATCC 29213, *Staphylococcus epidermidis* (clinical isolate), *Bacillus subtilis 168*, and Gram-negative bacteria: *Escherichia coli* ATCC 25922, *Klebsiella pneumoniae 1813* (clinical isolate), *Pseudomonas aeruginosa* ATCC 27853. The antibacterial activity of compounds **5a_12_**, **5c_10_**, **5c_12_**, **5d_10_**, **5f_10_**, **5g_10_**, **5i_10_**, **5l_10_**, **5m_8_**, **5n_10_**, **5o_8_**, **5p_8_**, and **5q_8_** was additionally evaluated on clinical methicillin-resistant strains of *Staphylococcus haemolyticus 837* MRSH (methicillin-resistant *Staphylococcus haemolyticus)*, *Staphylococcus aureus 967* MRSA (methicillin-resistant *Staphylococcus aureus)*, *Staphylococcus aureus* 983 MRSA, *Staphylococcus aureus* 25, *Staphylococcus intermedius* 1143 MRSI, *Staphylococcus aureus* 2020 MRSA, *Moraxella *sp. 764, *Moraxella *sp. 765, *Moraxella *sp. 829, *Acinetobacter *spp. 1, *Acinetobacter *spp. 3, *Pseudomonas *spp. 5, *Pseudomonas *spp. 6, *Klebsiella *spp. 11, *Klebsiella *spp. 12, and *Proteus *spp. 17, which were obtained from patients with bronchopulmonary diseases in the laboratory of bacteriology of the republic clinical hospital (Kazan, Russia).

The minimum inhibitory concentration (MIC) of compounds was determined by the broth microdilution method in 96-well microtiter plates (Eppendorf), according to the EUCAST (European Committee on Antimicrobial Susceptibility Testing) rules for antimicrobial susceptibility testing with some modifications [16]. The bacterial culture adjusted to 3–9 × 10^6^ cells/mL in the Mueller Hinton (MH) broth was seeded into 96-well polystyrol culture plates (Eppendorf). The concentrations of substances to be tested ranged from 0.25 to 64 µg/mL in two-fold serial dilutions. The minimal inhibitory concentration was determined as the lowest concentration of the compound, which leads to no visible bacterial growth after 24 h of incubation. To determine a minimum bactericidal concentration (MBC), a culture liquid from wells without visible growth was diluted one thousand-fold into new 96-well microtiter plates in fresh MH-broth and incubated for 24 h growth at 35 °C. MBC was assumed at concentrations where no viable growth could be observed.

### 3.3. Antifungal Activity

Antifungal activity of compounds **5a_12_**, **5c_10_**, **5c_12_**, **5d_10_**, **5f_10_**, **5g_10_**, **5i_10_**, **5l_10_**, **5m_8_**, **5n_10_**, **5o_8_**, **5p_8_**, and **5q_8_** was evaluated on several fungal strains, which causes cutaneous and systemic mycoses. The fungal isolates were obtained from a collection of the Kazan Institute of Microbiology and Epidemiology (Kazan, Russia): *Candida albicans* NCTC- 885–653 (etalon strain), *Aspergillus niger* F-1119 (etalon strain), *Trichophyton rubrum 1336* (clinical strain), and *Fusarium oxysporum* KM-19 (clinical strain). For the inoculum preparation, the pure 2-day and 5-day cultures of yeasts and filamentous fungi, respectively, grown on Sabouraud dense nutrient medium, were used. Yeast cultures of *C. albicans* were prepared by flushing the culture from the surface of the solid agar medium. Cultures of mycelial fungi *T. rubrum*, *F. oxysporum*, and *A. niger* were preground in a mortar. A suspension of microorganisms was prepared in a sterile 0.9% NaCl in pure water. The cell concentrations were (1–5) × 10^3^ for yeast fungi and (1–5) × 10^4^ for mycelial fungi.

The in vitro evaluation of the antifungal activity of compounds was carried out in a liquid glucose Sabouraud broth in biological test tubes by a two-fold serial dilution approach. Test compounds were prepared at concentrations ranging from 400 to 0.38 µg/mL. A test tube in the absence of test compounds served as a control. To each tube, 50 µL of inoculum was added. The tubes were incubated for 2–7 days at 30 °C. To the end of this period, the results were assessed by visual analysis of optical density of the medium. The following MIC endpoints were determined: 0 = clear solution, no growth, 1 = weak growth (25% control), 2 = significant inhibition of growth (50% control), 3 = insignificant growth inhibition (75% control), and 4 = no growth inhibition. All experiments were carried out in duplicate.

### 3.4. Cytotoxicity

The cytotoxicity was evaluated on HEK 293 (human embryonic kidney) cells by using the MTT (3-(4,5-dimethylthiazol-2-yl)-2,5-diphenyltetrazolium bromide) assay. Cells were cultured in α-MEM (minimum essential medium) supplemented with 10% FBS (fetal bovine serum), 2 mM L-glutamine, 100 μg/mL penicillin, and 100 μg/mL streptomycin. Cells were seeded in 96-well plates at the density of 10,000 cells per well and grown overnight at 37 °C and 5% CO_2_ in humidified atmosphere. Then the medium was changed to the fresh one containing compounds to be tested in a concentration of 0.1–100 μg/mL. After 72 h of cultivation, the cultural fluid was discarded and the MTT solution (in DPBS (Dulbecco’s phosphate-buffered saline)) was added to the fresh media until reaching a final concentration of 0.5 mg/mL. After 2 h, the liquid was replaced with dimethyl sulfoxide (Sigma-Aldrich, St. Louis, MO) to dissolve formazan crystals, and absorption was measured on Tecan Infinite 200Pro at 550 nm with a reference of 700 nm. Based on data obtained, the CC_50_ values (concentrations decreasing the metabolic activity by two-fold) were calculated.

### 3.5. Anti-Biofilm Activity

Bacterial biofilms were grown under static conditions in BM broth [29,30] for 48 h at 37 °C, washed, and exposed to 1–16 × MBCs of antimicrobials for 24 h in fresh BM. Then the culture liquid was discarded. The biofilms were washed with sterile 0.9% NaCl and suspended in 0.9% NaCl by scratching the well bottoms with the following treatment in a sonicator bath for 2 min at 20 kHz to favor the disintegration of cell clumps, and viable cells were counted using a drop plate assay [31] with modifications [32,33]. Serial 10-fold dilutions of each well were prepared in sterile 0.9% NaCl and 5 µL of the suspension was dropped onto solid LB (Luria broth) plates. CFUs were counted from those drops containing 5–10 colonies, which were averaged and presented as CFU per cm^2^.

### 3.6. Animals

Male and female CD-1 (ICR) mice (weighing 18–22 g) were purchased from the Federal Research Center Institute of Cytology and Genetics, Siberian Branch of the Russian Academy of Sciences. Male Sprague-Dawley rats weighing 200–230 g were purchased from the laboratory animals nursery “Pushchino,” Russia. Animals were given a laboratory rodent diet and water ad libitum. They were housed in polypropylene cages with sawdust at room temperature of 22 ± 3 °C and a relative humidity of 50 ± 20% with a constant 12-h light/dark cycle. All animals acclimatized for two weeks before the experiment. All experimental procedures were performed in accordance with the Ethical Principles in Animal Research and were approved by the Local Ethics Committee №2-2017 of the Kazan Federal University.

### 3.7. In Vivo Toxicity

Mice were stratified by weight and randomly assigned into one sex group of three per group (for initiating dose). The number of animals in the group was increased to six per group if necessary. Prior to the experiment, animals were weighed, marked, and fasted for 8 h with free access to water. Compounds **5c_12_**, **5d_10_**, and **5p_8_** were dissolved in distilled water and administered to the mice by oral gavage at doses 50, 100, 500, 1000, 1500, and 2000 mg/kg body weight. The initial dose of 1000 mg/kg was selected as the dose expected to produce mortality in some animals. Further groups of animals have been dosed at higher or lower fixed doses, depending on the presence of mortality, until the study objective was achieved. All animals were observed twice daily for symptoms and mortality for two weeks. All surviving animals were euthanized with CO_2_ inhalation at the end of the study on day 14, and their vital organs were individually observed for overt pathology by necropsy, and the LD_50_ was calculated by Probit analysis in IBM SPSS Statistics software.

### 3.8. In Vivo Efficiency in a Rat Skin Model

Gram-negative (*E. coli* CDC F-50™) bacteria were used as test organisms for the in vivo rat skin model. The culture of *E. coli* CDC F-50, grown on Mueller-Hinton’s medium (MHB, NICF, Russia) for 18–20 h, were washed off with sterile isotonic chloride solution, and centrifuged at 5000 rpm for 5 min. The supernatant was discharged and the cells were resuspended with sterile isotonic sodium chloride solution.

Endo medium (HiMedia Laboratories Pvt. Ltd., Mumbai, India) were used according to the manufacturers’ instructions. Phosphate-buffered saline (PBS, Ekoservice, Russia), Tween 80 (Sigma-Aldrich, St Louis, MO, USA), saponin (Dia-M, Moscow, Russia), histidine (Sigma-Aldrich, St Louis, MO, USA), and cysteine (Sigma-Aldrich, St Louis, MO, USA) were used for neutralizing solution (3% *v/v* Tween 80, 3% *w/v* saponin, 0.1% *w/v* histidine, 0.1% *w/v* cysteine, PBS).

Thirty rats were randomly assigned to five groups: four experimental groups and one control group with six rats in each group. All rats were anesthetized with isoflurane (induction 3.0% in air, maintenance of 1.5% in air) [34]. On the experimental day, dorsal sites of the animals more than 5 × 5 cm were shaved, and squares 5 × 5 cm were outlined with the marker. Suspension (0.25 mL) containing 1 × 10^7^ CFU *E. coli CDC F-50* were applied on the selected area, distributed with a sterile disposable spatula over the entire area of the square, and left for 3–4 min until it dries. Then a gauze cloth (5 × 5 cm) was applied to the rat’s skin for 5 min. In experimental groups, gauze cloths were preliminarily wetted in 1 mL of 0.2% **5c_12_**, miramistin, benzalkonium chloride or chlorhexidine solutions. In a negative control group, the tissue was immersed in 1 mL of sterile saline. At the end of the exposure time in experimental groups, the neutralizer was used to stop antiseptic action on bacteria. The rat’s skin was washed with a sterile gauze (5 × 5 cm) moistened with a sterile neutralizer solution. Then, the gauze cloth was transferred into 50-mL tubes with sterile physiological saline (10 mL) and shaken for 10 min. Then the washing liquid was placed into two to three plates (0.1 mL on each plate) in a selective Endo medium, and cultured for 24–48 h at 37 °C.

After the time required for cultivation of microorganisms, the results were recorded by the number of colonies grown on the Petri dish. The results were taken into account by assessing the residual contamination of surfaces after treating them with antiseptic solution. After counting the number of colonies grown on Petri dishes, the density of contamination per 1 cm^2^ of the surface and the percentage of disinfection were calculated, which corresponds to the number of colonies removed from the control surfaces that were not exposed to antiseptics as 100%. The percentage of growth inhibition of *E. coli* CDC F-50 was calculated using the following formula:

(1) $$I=100\%-\frac{E\times 100\%}{C},\;\mathrm{where}$$

I—percentage of growth inhibition, %,

C—the number of colonies in the control group,

E—the number of colonies in the experimental group.

### 3.9. Outer Membrane Permeability

The outer membrane permeability was assessed by measuring the fluorescent dye NPN (1-N-phenylnaphthylamine) uptake [35,36]. Gram-negative bacteria *(E. coli* CDCF-50) were incubated to mid-log phase in Mueller Hinton broth (37 °C, 120 rpm), harvested by centrifugation (3.000 rpm for 10 min), washed twice, and diluted to 10^5^ CFU/mL with 5 mM HEPES (2-[4-(2-hydroxyethyl)piperazin-1-yl]ethanesulfonic acid) buffer (pH 7.4, containing 5 mM glucose). Then, 200 μL of suspension were placed into a 96-well plate which were mixed with 10 μM of NPN. The background fluorescence was recorded (excitation λ = 350 nm, emission λ = 420 nm) by Spectrophotometer Infinite M200 PRO (Tecan, Switzerland). Next, 20 μL of **5c_12_** with final concentrations equal to minimal bactericidal concentration (MBC), 2 × MBC, and 4 × MBC, was added to the *E. coli* suspension into a 96-well plate. The fluorescence was recorded until no further increase of fluorescence was detected. The NPN uptake percent was calculated using the following formula:

NPN uptake (%) = (Fobs − F0)/(F100 − F0) × 100%, where(2)

Fobs—fluorescence of **5c_12_** in MBC/2 × MBC/4 × MBC,

F0—the initial NPN fluorescence with *E. coli* in the absence of **5c_12_**,

F100—NPN fluorescence with *E. coli* after addition of 10 μg/mL polymyxin B (Sigma-Aldrich, St Louis, MO, USA), which was used as a positive control.

### 3.10. Cytoplasmic Membrane Depolarization Assays

The cytoplasmic membrane depolarization was determined by using the membrane potential-sensitive fluorescent dye 3,3-Dipropylthiadicarbocyanine Iodide DiSC_3_(5), (Sigma-Aldrich). Gram-positive (*S. aureus* ATCC209p) and Gram-negative (*E. coli* CDCF-50) bacteria were incubated to a mid-log phase in Mueller Hinton broth (37 °C, 120 rpm), harvested by centrifugation (3.000 rpm for 10 min), washed twice, and diluted to an optical density of 0.05 at 600 nm with 5 mM HEPES buffer (pH 7.4, containing 20 mM glucose) containing 0.1 M KCl to equilibrate the cytoplasmic and external K^+^. Then, the cell suspensions (180 μL) were mixed with 20 μL of 0.8 μM DiSC_3_(5) in a 96-well plate and incubated for 15 min in darkness (until maximal dye uptake was reached, the background fluorescence was recorded (excitation λ = 620 nm, emission λ = 670 nm) by Spectrophotometer Infinite M200 PRO (Tecan, Switzerland). After that, **5c_12_** and reference compounds (benzalkonium chloride, miramistin) in 0.5, 1, and 2 × MBCs were added to cell suspensions into a 96-well plate. Changes in fluorescence were observed during 20 min of recording.

### 3.11. Scanning Electron Microscopy

The analysis was carried out using a high-resolution scanning electron microscope Merlin (Carl Zeiss, oberkochen, Germany). The surface morphology was studied at an accelerating voltage of primary electrons of 5 kV and a probe current of 300 pA for a minimal impact on the study object. Scanning electron microscopy was carried out in the Interdisciplinary center for Analytical Microscopy (Kazan Federal University).

## 4. Conclusions

In conclusion, an extended set of 108 novel quaternary bis-ammonium pyridoxine derivatives carrying various substituents at the quaternary nitrogens and acetal carbon have been synthesized. In the course of primary in vitro screening 13 compounds (**5a_12_**, **5c_10_**, **5c_12_**, **5d_10_**, **5f_10_**, **5g_10_**, **5i_10_**, **5l_10_**, **5m_8_**, **5n_10_**, **5o_8_**, **5p_8_**, and **5q_8_**) exhibiting comparable or superior antibacterial activity compared to miramistin, benzalkonium chloride and chlorhexidine were identified (MICs 0.25–4 µg/mL on Gram-positive and 0.5–16 µg/mL on Gram-negative bacteria). A strong correlation between the lipophilicity and antimicrobial activity was found. Most active compounds had logP values in the range of 1–3, while compounds with logP > 6 and logP < 0 were almost inactive. In further investigation of antibacterial and antifungal activity on clinical bacterial and fungal isolates, compounds **5c_12_**, **5i_10_**, **5l_10_**, **5n_10_**, **5o_8_**, **5p_8_**, and **5q_8_** significantly exceeded miramistin, benzalkonium chloride and chlorhexidine in antibacterial activity. Compounds **5c_10_**, **5c_12_**, **5d_10_**, **5f_10_**, **5n_10_**, and **5p_8_** exhibited a pronounced antimycotic activity than all reference antiseptics against all fungi tested. All active compounds had comparable values of cytotoxicity with miramistin and chlorhexidine and were less toxic when compared to benzalkonium chloride. In vivo, the leading compound **5c_12_** was considerably less toxic (LD_50_ 1705 mg/kg) than benzalkonium chloride (LD_50_ 180 mg/kg), miramistine (LD_50_ 1000 mg/kg) and chlorhexidine (LD_50_ 1260 mg/kg) at oral administration on CD-1 mice. Moreover, **5c_12_** demonstrated comparable or even higher activity than that of the benzalkonium chloride on biofilm-embedded *S. aureus*, *S. epidermidis*, *E. coli*, and *P. aeruginosa*. A 0.2% aqueous solution of **5c_12_** showed 97.5% growth inhibition of *E. coli* CD CF-50 after 5 min of exposure on rat’s skin comparable to reference drugs efficiency. The molecular mechanism of antibacterial activity of **5c_12_** apparently is the membrane damage since a **5c_12_** dose-dependent effect on membrane permeability and polarization was observed. The obtained results allow suggesting described quaternary bis-ammonium pyridoxine derivatives as promising lead molecules for the development of the new antiseptics with a broad spectrum of antimicrobial and antifungal activity.

## Figures and Tables

**Table 1:** In vitro antibacterial activity of novel bis-ammonium salts of pyridoxine ^a^.

Compound	MICs (µg/mL)						LogP
	Gram-Positive Bacteria			Gram-Negative Bacteria			
	*S. aureus* ATCC 29213	*S. epidermidis* *(clinical)*	*B. subtilis* 168	*E. coli* ATCC 25922	*K. pneumoniae* 1813	*P. aeruginosa* ATCC 27853	
**5a_8_**	64	64	64	>64	64	>64	–1.74
**5a_10_**	2	2	2	32	2	>64	0.03
**5a_12_**	**4**	**2**	**8**	**16**	**4**	**32**	**1.81**
**5a_14_**	32	64	>64	>64	64	>64	3.59
**5a_16_**	>64	>64	>64	>64	>64	>64	5.37
**5a_18_**	>64	>64	>64	>64	>64	>64	7.15
**5b_8_**	64	>64	>64	>64	>64	>64	–1.51
**5b_10_**	2	2	2	32	2	64	0.26
**5b_12_**	8	1	8	16	4	16	2.04
**5b_14_**	>64	>64	>64	>64	>64	>64	3.82
**5b_16_**	64	64	64	64	64	64	5.60
**5b_18_**	>64	>64	>64	>64	>64	>64	7.38
**5c_8_**	32	32	64	>64	32	>64	–0.88
**5c_10_**	**0.5**	**0.5**	**32**	**8**	**1**	**16**	**0.90**
**5c_12_**	**1**	**2**	**4**	**16**	**4**	**32**	**2.68**
**5c_14_**	16	16	64	64	64	64	4.46
**5c_16_**	>64	>64	>64	>64	>64	>64	6.24
**5c_18_**	>64	>64	>64	>64	>64	>64	8.01
**5d_8_**	16	16	>64	>64	16	>64	–0.43
**5d_10_**	**0.25**	**0.5**	**8**	**2**	**1**	**8**	**1.35**
**5d_12_**	1	1–2	2	16	2	16	3.12
**5d_14_**	16	16	>64	>64	64	64	4.90
**5d_16_**	>64	>64	>64	>64	>64	>64	6.68
**5d_16_**	>64	>64	>64	>64	>64	>64	8.46
**5e_8_**	32	32	32	>64	32	>64	–0.40
**Chlorhexidine**	2	1	1	1	2	4	4.51
**Miramistin**	2	2	2	4	>64	64	2.43
**Benzalkonium chloride**	1	2	0.5	1	>64	64	-
**5e_10_**	2	1	2	8	1	16	1.38
**5e_12_**	8	2	4	8	8	32	3.16
**5e_14_**	16	32	64	>64	32	>64	4.94
**5e_16_**	>64	>64	>64	>64	>64	>64	6.72
**5e_18_**	>64	>64	>64	>64	>64	>64	8.49
**5f_8_**	4	8	64	64	8	64	0.01
**5f_10_**	**0.25**	**0.5**	**4**	**2**	**1**	**4**	**1.79**
**5f_12_**	1	1	2	32	4	32	3.57
**5f_14_**	32	64	>64	>64	64	>64	5.35
**5f_16_**	>64	>64	>64	>64	>64	>64	7.13
**5f_18_**	>64	>64	>64	>64	>64	>64	8.90
**5g_8_**	8	16	8	64	8	64	0.09
**5g_10_**	**0.5**	**0.5**	**1**	**2**	**0.5**	**8**	**1.84**
**5g_12_**	1	1	4	2	2	8	3.65
**5g_14_**	32	32	64	64	64	>64	5.43
**5g_16_**	>64	>64	>64	>64	>64	>64	7.21
**5g_18_**	>64	>64	>64	>64	>64	>64	8.99
**5h_8_**	8	16	8	64	16	>64	0.05
**5h_10_**	2	1	1	4	2	8	1.83
**5h_12_**	4	4	4	8	8	32	3.60
**5h_14_**	16	>64	>64	>64	>64	>64	5.38
**5h_16_**	>64	>64	>64	>64	>64	>64	7.16
**5h_18_**	>64	>64	>64	>64	>64	>64	8.94
**5i_8_**	2	2	2	32	2	32	0.46
**5i_10_**	**0.5**	**0.5**	**2**	**1**	**1**	**4**	**2.24**
**5i_12_**	1	2	8	16	8	32	4.01
**5i_14_**	64	64	>64	>64	>64	>64	5.79
**5i_16_**	>64	>64	>64	>64	>64	>64	7.57
**5i_18_**	>64	>64	>64	>64	>64	>64	9.35
**5j_8_**	4	4	4	64	4	64	0.49
**5j_10_**	1	1	1	2	2	4	2.27
**Chlorhexidine**	2	1	1	1	2	4	4.51
**Miramistin**	2	2	2	4	>64	64	2.43
**Benzalkonium chloride**	1	2	0.5	1	>64	64	-
**5j_12_**	8	4	16	8	8	32	4.05
**5j_14_**	32	64	>64	>64	>64	>64	5.83
**5j_16_**	>64	>64	>64	>64	>64	>64	7.60
**5j_18_**	>64	>64	>64	>64	>64	>64	9.38
**5k_8_**	4	4	4	64	4	64	0.49
**5k_10_**	1	1	2	2	2	4	2.27
**5k_12_**	8	4	16	8	32	32	4.05
**5k_14_**	32	64	>64	>64	>64	>64	5.83
**5k_16_**	>64	>64	>64	>64	>64	>64	7.60
**5k_18_**	>64	>64	>64	>64	>64	>64	9.38
**5l_8_**	1	1	8	1	16	1	0.90
**5l_10_**	**0.5**	**2**	**1**	**0.5**	**4**	**0.5**	**2.68**
**5l_12_**	8	4	16	16	16	>64	4.46
**5l_14_**	32	32	>64	>64	>64	>64	6.24
**5l_16_**	>64	>64	>64	>64	>64	>64	8.01
**5l_18_**	>64	>64	>64	>64	>64	>64	9.79
**5m_8_**	**0.5**	**0.5**	**1**	**2**	**0.5**	**16**	**1.35**
**5m_10_**	1	1	2	2	4	8	3.12
**5m_12_**	8	16	32	>64	32	>64	4.90
**5m_14_**	>64	>64	>64	>64	>64	>64	6.68
**5m_16_**	>64	>64	>64	>64	>64	>64	8.46
**5m_18_**	>64	>64	>64	>64	>64	>64	10.24
**5n_8_**	1	1	1	8	1	16	1.38
**5n_10_**	**1**	**1**	**2**	**4**	**2**	**4**	**3.16**
**5n_12_**	8	16	32	32	32	64	4.94
**5n_14_**	>64	>64	>64	>64	>64	>64	6.72
**5n_16_**	>64	>64	>64	>64	>64	>64	8.49
**5n_18_**	>64	>64	>64	>64	>64	>64	10.27
**5o_8_**	**1**	**1**	**2**	**2**	**1**	**8**	**2.24**
**5o_10_**	8	2	8	16	4	16	4.01
**5o_12_**	16	8	64	32	32	64	5.79
**Chlorhexidine**	2	1	1	1	2	4	4.51
**Miramistin**	2	2	2	4	>64	64	2.43
**Benzalkonium chloride**	1	2	0.5	1	>64	64	-
**5o_14_**	>64	>64	>64	>64	>64	>64	7.57
**5o_16_**	>64	>64	>64	>64	>64	>64	9.35
**5o_18_**	>64	>64	>64	>64	>64	>64	11.13
**5p_8_**	**2**	**2**	**2**	**4**	**4**	**8**	**3.16**
**5p_10_**	8	8	32	32	64	>64	4.94
**5p_12_**	>64	>64	>64	>64	>64	>64	6.72
**5p_14_**	>64	>64	>64	>64	>64	>64	8.49
**5p_16_**	>64	>64	>64	>64	>64	>64	10.27
**5p_18_**	>64	>64	>64	>64	>64	>64	12.05
**5q_8_**	**2**	**0.5**	**2**	**2**	**1**	**8**	**2.04**
**5q_10_**	2	2	4	8	4	8	3.82
**5q_12_**	16	32	>64	>64	>64	>64	5.60
**5q_14_**	>64	>64	>64	>64	>64	>64	7.37
**5q_16_**	>64	>64	>64	>64	>64	>64	9.15
**5q_18_**	>64	>64	>64	>64	>64	>64	10.93
**5r_8_**	16	16	16	64	16	>64	–0.01
**5r_10_**	2	1	1	2	1	16	1.77
**5r_12_**	8	2	4	16	4	16	3.55
**5r_14_**	32	64	>64	64	>64	>64	5.32
**5r_16_**	>64	>64	>64	>64	>64	>64	7.10
**5r_18_**	>64	>64	>64	>64	>64	>64	8.88
**Chlorhexidine**	2	1	1	1	2	4	4.51
**Miramistin**	2	2	2	4	>64	64	2.43
**Benzalkonium chloride**	1	2	0.5	1	>64	64	-

^a^ MICs for the most active compounds are highlighted in bold.

**Table 2:** In vitro antimicrobial activity of novel bis-ammonium salts of pyridoxine on clinical bacterial isolates.

Strains	MICs (µg/mL)															
	5a_12_	5c_10_	5c_12_	5d_10_	5f_10_	5g_10_	5i_10_	5l_10_	5m_8_	5n_10_	5o_8_	5p_8_	5q_8_	Chlorhexidine	Benzalkonium Chloride	Miramistin
*S. haemolyticus* 837 MRSH	16	64	4	64	16	16	8	4	32	4	8	4	8	64	32	64
*S. aureus* 967 MRSA	16	64	4	64	16	16	8	4	32	4	8	4	8	64	32	64
*S. aureus* 983 MRSA	16	64	4	64	16	16	8	4	32	4	8	4	8	64	32	64
*S. aureus* 25	2	1	4	2	1	4	2	4	1	2	1	2	1	2	2	16
*S. intermedius* 1143 MRSI	2	2	2	4	2	2	2	2	2	2	1	1	1	4	4	32
*S. aureus* 2020 MRSA	32	>64	8	64	16	16	16	8	64	4	8	4	8	64	32	64
*Moraxella *sp.764	16	64	4	64	8	8	8	4	32	2	4	2	4	>64	32	64
*Moraxella *sp.765	8	64	4	64	8	8	8	4	32	2	4	2	4	>64	32	64
*Moraxella *sp.829	16	64	4	64	8	8	8	4	32	4	8	2	4	>64	32	64
*Acinetobacter *spp. 1	32	64	8	64	16	16	16	4	64	4	8	8	8	64	32	64
*Acinetobacter *spp. 3	32	64	8	64	16	16	16	8	32	4	8	4	8	64	32	64
*Pseudomonas *spp. 5	16	>64	4	64	16	16	8	4	64	4	8	4	8	>64	32	64
*Pseudomonas *spp. 6	32	64	4	64	16	16	16	4	32	4	8	2	8	>64	32	64
*Klebsiella *spp. 11	32	64	8	64	16	16	8	4	32	4	8	4	8	>64	32	>64
*Klebsiella *spp. 12	32	>64	8	>64	16	16	16	4	64	4	8	4	8	>64	32	64
*Proteus *spp. 17	>64	>64	64	>64	64	32	64	>64	>64	>64	64	>64	64	>64	64	>64

**Table 3:** In vitro antifungal activity of novel bis-ammonium salts of pyridoxine.

Compound	Minimum Inhibitory Concentrations (MICs) (µg/mL)			
	*Trichophyton rubrum* 1336 (clinical)	*Aspergillus niger* F-1119	*Candida albicans* NCTC- 885-653	*Fusarium oxysporum* KM-19 (clinical)
**5a_12_**	32	16	16	32
**5c_10_**	16	8	4	8
**5c_12_**	16	8	4	8
**5d_10_**	2	4	2	4
**5f_10_**	16	16	8	16
**5g_10_**	32	32	32	32
**5i_10_**	32	16	16	32
**5l_10_**	32	16	16	32
**5m_8_**	128	128	128	128
**5n_10_**	8	4	4	8
**5o_8_**	32	32	32	32
**5p_8_**	8	4	4	8
**5q_8_**	64	32	32	64
**Miramistin**	32	32	32	32
**Chlorhexidine**	128	128	64	128
**Benzalkonium chloride**	16	8	8	16

**Table 4:** Cytotoxicity of novel bis-ammonium salts of pyridoxine.

Compound	CC_50_, µg/mL (HEK-293)
**5a_12_**	3.11 ± 0.5
**5c_10_**	3.83 ± 0.7
**5c_12_**	3.38 ± 0.8
**5d_10_**	2.81 ± 1.2
**5f_10_**	4.04 ± 0.6
**5g_10_**	3.86 ± 0.8
**5i_10_**	2.22 ± 0.4
**5l_10_**	2.79 ± 0.8
**5m_8_**	3.48 ± 1.2
**5n_10_**	2.02 ± 0.4
**5o_8_**	4.26 ± 0.9
**5p_8_**	3.11 ± 0.8
**5q_8_**	3.69 ± 0.7
**Miramistin**	6.83 ± 1.1
**Chlorhexidine**	5.48 ± 1.5
**Benzalkonium chloride**	1.02 ± 0.4

**Table 5:** Toxicology data obtained on oral administration of compounds **5c_12_**, **5d_10_**, and **5p_8_** to CD-1 mice.

Compound	LD_50_, mg/kg
**5c_12_**	1705
**5d_10_**	809
**5p_8_**	474
**Benzalkonium chloride**	180 [22]
**Miramistin**	1000 [23]
**Chlorhexidine**	1260 [24]

**Table 6:** Bactericidal activity of compound **5c_12_**, benzalkonium chloride, and miramistin.

Strain	MBC (µg/mL)		
	5c_12_	Benzalkonium Chloride	Miramistin
*S. aureus* ATCC 29213	4	4	8
*S. epidermidis*	8	8	4
*P. aeruginosa* ATCC 27853	128	128	128
*E. coli* ATCC 25922	64	2	8

**Table 7:** In vivo antibacterial efficacy (Mean ± SD) of 0.2% aqueous solution **5c_12_**, miramistin, benzalkonium chloride and chlorhexidine in a rat skin model, *n* = 6.

Compound	Growth Inhibition Percentage (CFU/cm^2^)
**5c_12_**	97.5 ± 1.7
**Miramistin**	97.2 ± 1.8
**Benzalkonium chloride**	96.8 ± 2.0
**Chlorhexidine**	95.7 ± 3.4

## Supplementary Materials

The following are available online at https://www.mdpi.com/1420-3049/25/18/4341/s1, methods of synthesis, analytical characteristics, and copies of ^1^H and ^13^C NMR spectra of all new synthesized compounds are described in the Electronic Supplementary Material.
