# Supplementary material for: Novel Bis-Ammonium Salts of Pyridoxine: Synthesis and Antimicrobial Properties
Source: Molecules. 2020 Sep 22;25(18):4341. doi: 10.3390/molecules25184341 (PMC7570726; doi:10.3390/molecules25184341)
Supplement: Supplementary file 1 [file molecules-25-04341-s001.pdf]

molecules

Supplementary Material

Novel bis-ammonium salts of pyridoxine: synthesis and antimicrobial properties

Nikita V. Shtyrlin[1], Mikhail V. Pugachev[1], Sergey V. Sapozhnikov[1], Marsel R. Garipov[1], Rusalia M. Vafina[1], Denis Yu. Grishaev[1], Roman S. Pavelyev[1], Renata R. Kazakova[1], Mariya N. Agafonova[1], Alfiya G. Iksanova[1], Svetlana A. Lisovskaya[2], Marina I. Zeldi[1], Elena S. Krylova[1], Elena V. Nikitina[1], Alina E. Sabirova[1], Airat R. Kayumov[1] and Yurii G. Shtyrlin[1]*

[1] Kazan (Volga region) Federal University, Scientific and Educational Center of Pharmaceutics, Kremlyovskaya St. 18, Kazan, 420008, Russian Federation;

[2] Kazan Scientific Research Institute of Epidemiology and Microbiology, Kazan 420015, Russian Federation;

* Correspondence: yurii.shtyrlin@kpfu.ru; Tel.: +7-843-233-7363

Contents

General information	S2
Methods for the preparation of compounds	S3
References	S62
NMR Spectra	S63

General information

Chromatography

Chromatographic purification of compounds was carried out using column chromatography on Acros silica gel (60—200 mesh). Reaction progress and purity of compounds were monitored by TLC on Sorbfil PTLC-AF-A-UF plates.

Melting Points

Melting points of the products were determined using a Stanford Research Systems MPA-100 OptiMelt appliance.

NMR Spectroscopy

^{1}H, ^{13}C, HSQC, NOESY NMR spectra were recorded on a "Bruker Avance 400" spectrometer (operating frequency 400.17 and 100.62 MHz, respectively). Signals of chloroform-d (δ_H 7.24, δ_C 77.23) and dimethyl sulfoxide (δ_H 2.50, δ_C 39.51) were used as references in the ^{1}H and ^{13}C NMR spectra [1, 2]. Coupling constants (*J*) are reported in Hz (splitting abbreviations: s, singlet; d, doublet; t, triplet; q, quartet; m, multiplet; br, broad; AB, AB-system and combinations thereof).

Mass Spectrometry

High-resolution mass spectra (HRMS) were obtained on a quadrupole time-of-flight (qTOF) AB Sciex Triple TOF 5600 mass spectrometer using turbo-ion spray source (nebulizer gas nitrogen, a positive ionization polarity, needle voltage 5500 V). Recording of the spectra was performed in "TOF MS" mode with collision energy 10 eV, declustering potential 100 eV and with resolution more than 30 000 full-width half-maximum. Samples with the analyte concentration 5 μmol/l were prepared by dissolving the test compounds in a mixture of methanol (HPLC-UV Grade, LabScan) and water (LC-MS Grade, Panreac) in 1:1 ratio.

Methods for the preparation of compounds

Compounds **2a,c,d,f,g,o,p** and **3a,b,c,d,f,g,o,p** has been described in our previous work [3]. Compound **2b** has been reported by Arustamova and Kulnevich [4]. Compounds **2e** and **3e** have been described in a patent [5]. Compound **2i** has been described in our recent work [6]. Compound **2r** has been described in our previous work [7]. Other compounds reported in this paper are novel and not described in literature. Compounds **2h,j,k,n** were obtained in the reactions without isolation. The synthesis of compound **5c12** was described earlier in patent [8], but slightly modified in this work.

General method for the preparation pyridoxine acetals and ketals 2

To 10.00 g (1 equiv) a suspension of pyridoxine hydrochloride **1** in 140 ml of toluene were added *para*-toluenesulfonic acid monohydrate (2.1 equiv) and aldehyde (or ketone) (1.5 equiv). The reaction mixture was refluxed with a Dean-Stark trap for 17 h. Then the solvent was evaporated under reduced pressure and the residue was neutralized with aqueous solution of NaOH. The aqueous solution was washed with 100 ml of chloroform, organic layer was separated and dried. The precipitate was washed successively with petroleum ether, methyl *tert*-butyl ether, an aqueous alkali solution and dried.

5-(Hydroxymethyl)-2-hexyl-8-methyl-4H-[1,3]dioxino[4,5-c]pyridin (2l)

Yield 55%; white solid; mp 104-105 °C; ^{1}H NMR (DMSO-*d*$_6$, 400 MHz) δ 0.87 (t, 3H, $^3J_{HH}$ = 6.7 Hz, $\underline{CH_3}(CH_2)_5$), 1.20-1.39 (m, 6H, $CH_3\underline{(CH_2)_3}(CH_2)_2$), 1.42-1.51 (m, 2H, $CH_3(CH_2)_3\underline{CH_2}CH_2$), 1.72-1.84 (m, 2H, $CH_3(CH_2)_3CH_2\underline{CH_2}$), 2.29 (s, 3H, CH_{3pyr}), 4.38 (s, 2H, CH_2), 4.94 (s, 2H, CH_2), 5.10 (t, 1H, $^3J_{HH}$ = 5.1 Hz, $\underline{CH}C_6H_{13}$), 5.17 (br s, 1H, OH), 7.93 (s, 1H, CH_{pyr}); ^{13}C NMR (DMSO-*d*$_6$, 100 MHz) δ 13.97 (CH_3), 18.13 (CH_{3pyr}), 22.05 (CH_2), 23.09 (CH_2), 28.43 (CH_2), 31.21 (CH_2), 33.76 (CH_2), 58.19 (CH_2O), 63.41 (CH_2O), 99.31 ($\underline{C}HC_6H_{13}$), 126.88 (C_{pyr}), 130.91 (C_{pyr}), 138.94 (C_{pyr}), 145.06 (C_{pyr}), 146.91 (C_{pyr}); HRMS-ESI $[M+H]^+$ 266.1756 (calculated for $C_{15}H_{24}NO_3$, 266.1751).

5-(Hydroxymethyl)-2-heptyl-8-methyl-4H-[1,3]dioxino[4,5-c]pyridin (2m)

Yield 34%; white solid; mp 70-71 °C; ^{1}H NMR (DMSO-d_6, 400 MHz) δ 0.86 (t, 3H, $^3J_{HH}$ = 6.5 Hz, $\underline{CH_3}(CH_2)_6$), 1.21-1.35 (m, 8H, $CH_3\underline{(CH_2)_4}(CH_2)_2$), 1.41-1.52 (m, 2H, $CH_3(CH_2)_4\underline{CH_2}CH_2$), 1.71-1.83 (m, 2H, $CH_3(CH_2)_4CH_2\underline{CH_2}$), 2.29 (s, 3H, CH_{3pyr}), 4.38 (d, 2H, $^3J_{HH}$ = 4.8 Hz, CH_2), 4.94 (s, 2H, CH_2), 5.10 (t, 1H, $^3J_{HH}$ = 4.8 Hz, OH), 5.18 (t, 1H, $^3J_{HH}$ = 4.9 Hz, $\underline{CH}C_7H_{15}$), 7.93 (s, 1H, CH_{pyr}); ^{13}C NMR (DMSO-d_6, 100 MHz) δ 14.00 (CH_3), 18.13 (CH_{3pyr}), 22.11 (CH_2), 23.14 (CH_2), 28.66 (CH_2), 28.74 (CH_2), 31.24 (CH_2), 33.76 (CH_2), 58.19 (CH_2O), 63.42 (CH_2O), 99.31 ($\underline{C}HC_7H_{15}$), 126.88 (C_{pyr}), 130.91 (C_{pyr}), 138.94 (C_{pyr}), 145.05 (C_{pyr}), 146.91 (C_{pyr}); HRMS-ESI $[M+H]^+$ 280.1913 (calculated for $C_{16}H_{26}NO_3$, 280.1907).

5-(Hydroxymethyl)-2,8-dimethyl-2-octyl-4H-[1,3]dioxino[4,5-c]pyridin (2q)

Yield 87 %; white solid; mp 108-109 °C; ^{1}H NMR ($CDCl_3$, 400 MHz) δ 0.87 (t, 3H, $^3J_{HH}$ = 6.8 Hz, $\underline{CH_3}(CH_2)_7$), 1.22-1.32 (m, 10H, $CH_3\underline{(CH_2)_5}(CH_2)_2$), 1.42-1.50 (m, 2H, $CH_3(CH_2)_5\underline{CH_2}CH_2$), 1.47 (s, 3H, CH_3), 1.74-1.83 (m, 2H, $CH_3(CH_2)_6\underline{CH_2}$), 2.36 (s, 3H, CH_{3pyr}), 4.54 (s, 2H, CH_2), 4.91 (s, 2H, CH_2), 7.81 (s, 1H, CH_{pyr}); ^{13}C NMR (DMSO-d_6, 100 MHz) δ 13.98 (CH_3), 18.23 (CH_{3pyr}), 21.92 (CH_3), 22.11 (CH_2), 22.89 (CH_2), 28.63 (CH_2), 28.91 (CH_2), 29.08 (CH_2), 31.29 (CH_2), 37.27 (CH_2), 57.59 (CH_2O), 58.21 (CH_2O), 100.84 ($\underline{C}(CH_3)C_8H_{17}$), 125.25 ($C_{pyr}$), 130.55 ($C_{pyr}$), 138.34 ($C_{pyr}$), 144.95 ($C_{pyr}$), 145.36 ($C_{pyr}$); HRMS-ESI $[M+H]^+$ 308.2225 (calculated for $C_{18}H_{30}NO_3$, 308.2220).

General methods for the preparation of chloro derivatives of pyridoxine 3

Method 1: Thionyl chloride (7 equiv) was added to a solution of 4.00 g (1 equiv) of compound **2** in 25 ml of chloroform. The resulting reaction mixture was stirred at room temperature for 17 h. The solvent was removed under reduced pressure.

Method 2: To 10.00 g (1 equiv) a suspension of pyridoxine hydrochloride **1** in 140 ml of toluene were added *para*-toluenesulfonic acid monohydrate (2.1 equiv) and aldehyde (1.5 equiv). The reaction mixture was refluxed with a Dean-Stark trap for 17 h. Then the solvent was evaporated under reduced pressure and the residue was neutralized with aqueous solution of NaOH. The aqueous solution was washed with 100 ml of chloroform, organic layer was separated and dried. The oily residue was

dissolved in 40 ml of chloroform and thionyl chloride (2.5 equiv) was added to the resulting solution in small portions with stirring. The resulting reaction mixture was kept at room temperature for 17 h. The solvent was removed under reduced pressure. The precipitate was washed twice with methyl *tert*-butyl ether and dried.

2-(Sec-butyl)-5-(chloromethyl)-8-methyl-4H-[1,3]dioxino[4,5-c]pyridine hydrochloride (mixture of two diastereomers) (3h)

The target compound was synthesized according to general method 2.

Yield 80 %; gray solid; mp 168 °C (dec.); ^{1}H NMR (DMSO-d_6, 400 MHz) δ 0.94 (t, 6H, $^3J_{HH}$ = 7.4 Hz, $\underline{CH_3}CH_2CH(CH_3)$), 1.00-1.02 (m, 6H, $C_3H_7CH(\underline{CH_3})$), 1.25-1.36 (m, 2H, $CH_3\underline{CH_2}CH(CH_3)$), 1.59-1.71 (m, 2H, $CH_3\underline{CH_2}CH(CH_3)$), 1.81-1.90 (m, 2H, $CH_3CH_2\underline{CH}(CH_3)$), 2.55 (s, 6H, CH_{3pyr}), 4.87, 4.93 (AB, 4H, $^2J_{HH}$ = 12.0 Hz, CH_2), 5.20, 5.31 (AB, 4H, $^2J_{HH}$ = 16.0 Hz, CH_2), 5.21 (d, 2H, $^3J_{HH}$ = 4.3 Hz, CH), 8.42 (s, 2H, CH_{pyr}); ^{13}C NMR (DMSO-d_6, 100 MHz) δ 11.18 (CH_3), 11.22 (CH_3), 12.76 (CH_3), 12.87 (CH_3), 14.40 (CH_3), 14.45 (CH_3), 23.26 (CH_2), 23.37 (CH_2), 37.92 (CH), 37.94 (CH), 39.06 (CH_2Cl), 63.52 (CH_2O), 102.81 ($\underline{C}HCH(CH_3)C_2H_5$), 102.85 ($\underline{C}HCH(CH_3)C_2H_5$), 130.42 ($C_{pyr}$), 133.07 ($C_{pyr}$), 135.45 ($C_{pyr}$), 143.87 ($C_{pyr}$), 143.90 ($C_{pyr}$), 149.42 ($C_{pyr}$); HRMS-ESI [M-Cl]$^+$ 256.1104 (calculated for $C_{13}H_{19}ClNO_2$, 256.1099).

5-(Chloromethyl)-8-methyl-2-pentyl-4H-[1,3]dioxino[4,5-c]pyridine hydrochloride (3i)

The target compound was synthesized according to general method 1.

Yield 100%; white solid; mp 171 °C (dec.); ^{1}H NMR (DMSO-d_6, 400 MHz) δ 0.88 (t, 3H, $^3J_{HH}$ = 6.9 Hz, $\underline{CH_3}(CH_2)_4$), 1.26-1.37 (m, 4H, $CH_3\underline{(CH_2)_2}(CH_2)_2$), 1.45-1.53 (m, 2H, $CH_3(CH_2)_2\underline{CH_2}CH_2$), 1.78-1.88 (m, 2H, $CH_3(CH_2)_3\underline{CH_2}$), 2.55 (s, 3H, CH_{3pyr}), 4.88, 4.93 (AB, 2H, $^2J_{HH}$ = 12.0 Hz, CH_2), 5.21, 5.29 (AB, 2H, $^2J_{HH}$ = 17.2 Hz, CH_2), 5.35 (t, 1H, $^3J_{HH}$ = 5.1 Hz, CH), 8.44 (s, 1H, CH_{pyr}); ^{13}C NMR (DMSO-d_6, 100 MHz) δ 13.87 (CH_3), 14.26 (CH_{3pyr}), 21.98 (CH_2), 22.50 (CH_2), 30.84 (CH_2), 33.25 (CH_2), 38.99 (CH_2Cl), 63.37 (CH_2O), 100.68 (CH), 130.57 (C_{pyr}), 132.78 (C_{pyr}), 135.72 (C_{pyr}), 143.66 (C_{pyr}), 149.37 (C_{pyr}); HRMS-ESI [M-Cl]$^+$ 270.1261(calculated for $C_{14}H_{21}ClNO_2$, 270.1255).

2-(Sec-pentyl)-5-(chloromethyl)-8-methyl-4H-[1,3]dioxino[4,5-c]pyridine hydrochloride (mixture of two diastereomers) (3j)

The target compound was synthesized according to general method 2.

Yield 80 %; gray solid; mp 153 °C (dec.); ^{1}H NMR (DMSO-d_6, 400 MHz) δ 0.91 (t, 3H, $^3J_{HH}$ = 7.0 Hz, $\underline{CH_3}C_2H_4CH(CH_3)$), 1.00-1.03 (m, 3H, $C_3H_7CH(\underline{CH_3})$), 1.22-1.37 (m, 2H, $CH_3\underline{CH_2}CH_2CH(CH_3)$), 1.40-1.52 (m, 1H, $C_2H_5\underline{CH_2}CH(CH_3)$), 1.53-1.63 (m, 1H, $CH_3CH_2\underline{CH_2}CH(CH_3)$), 1.91-2.01 (m, 1H, $C_3H_7\underline{CH}(CH_3)$), 2.58 (s, 3H, CH_{3pyr}), 4.90, 4.95 (AB, 2H, $^2J_{HH}$ = 12.0 Hz, CH_2), 5.22, 5.34 (AB, 2H, $^2J_{HH}$ = 16.0 Hz, CH_2), 5.21 (d, 1H, $^3J_{HH}$ = 3.8 Hz, CH), 8.46 (s, 1H, CH_{pyr}); ^{13}C NMR (DMSO-d_6, 100 MHz) δ 13.21 (CH_3), 13.26 (CH_3), 14.08 (CH_3), 14.14 (CH_3), 14.17 (CH_3), 19.41 (CH_2), 32.53 (CH_2), 32.65 (CH_2), 36.09 (CH), 38.95 (CH_2Cl), 63.55 (CH_2O), 103.01 (CH), 103.03 (CH), 130.67 (C_{pyr}), 132.48 (C_{pyr}), 135.98 (C_{pyr}), 143.58 (C_{pyr}), 143.61 (C_{pyr}), 149.55 (C_{pyr}), 149.59 (C_{pyr}); HRMS-ESI $[M-Cl]^+$ 270.1261 (calculated for $C_{14}H_{21}ClNO_2$, 270.1255).

5-(Chloromethyl)-8-methyl-2-(pentan-3-yl)-4H-[1,3]dioxino[4,5-c]pyridine hydrochloride (3k)

The target compound was synthesized according to general method 2.

Yield 96 %; gray solid; mp 156 °C (dec.); ^{1}H NMR (DMSO-d_6, 400 MHz) δ 0.93 (t, 6H, $^3J_{HH}$ = 7.4 Hz, $(\underline{CH_3}CH_2)_2CH$), 1.38-1.51 (m, 2H, $(CH_3\underline{CH_2})_2CH$), 1.54-1.63 (m, 2H, $(CH_3\underline{CH_2})_2CH$), 1.66-1.73 (m, 1H, $(CH_3CH_2)_2\underline{CH}$), 2.57 (s, 3H, CH_{3pyr}), 4.89, 4.95 (AB, 2H, $^2J_{HH}$ = 12.0 Hz, CH_2), 5.23, 5.34 (AB, 2H, $^2J_{HH}$ = 16.0 Hz, CH_2), 5.32 (d, 1H, $^3J_{HH}$ = 3.3 Hz, CH), 8.45 (s, 1H, CH_{pyr}); ^{13}C NMR (DMSO-d_6, 100 MHz) δ 1.18 (CH_3), 11.27 (CH_3), 14.05 (CH_{3pyr}), 20.23 (CH_2), 38.93 (CH), 43.95 (CH_2Cl), 63.65 (CH_2O), 102.22 (CH), 130.72 (C_{pyr}), 132.34 (C_{pyr}), 136.16 (C_{pyr}), 143.53 (C_{pyr}), 149.68 (C_{pyr}); HRMS-ESI $[M-Cl]^+$ 270.1255 (calculated for $C_{14}H_{21}ClNO_2$, 270.1255).

5-(Chloromethyl)-2-hexyl-8-methyl-4H-[1,3]dioxino[4,5-c]pyridine hydrochloride (3l)

The target compound was synthesized according to general method 1.

Yield 100%; gray solid; mp 157 °C (dec.); ^{1}H NMR (DMSO-d_6, 400 MHz) δ 0.12 (t, 3H, $^3J_{HH}$ = 6.8 Hz, $\underline{CH_3}(CH_2)_5$), 0.50-0.67 (m, 6H, $CH_3\underline{(CH_2)_3}(CH_2)_2$), 0.71-0.82 (m, 2H, $CH_3(CH_2)_3\underline{CH_2}CH_2$), 1.07-1.18 (m, 2H, $CH_3(CH_2)_3CH_2\underline{CH_2}$), 1.82 (s, 3H, CH_{3pyr}), 3.96, 4.01 (AB, 2H, $^2J_{HH}$ = 12.7 Hz, CH_2), 4.47, 4.53 (AB, 2H, $^2J_{HH}$ = 18.0 Hz, CH_2), 4.54 (t, 1H, $^3J_{HH}$ = 5.1 Hz, $\underline{CH}C_6H_{13}$), 7.57 (s, 1H, CH_{pyr}); ^{13}C NMR (DMSO-d_6, 100 MHz) δ 4.86 (CH_3), 4.93 (CH_2), 14.12 (CH_{3pyr}), 14.80 (CH_2), 20.60 (CH_2), 23.36 (CH_2), 25.42 (CH_2), 29.80 (CH_2Cl),

55.58 (CH_2O), 93.59 ($\underline{C}HC_6H_{13}$), 123.21 (C_{pyr}), 123.99 (C_{pyr}), 130.02 (C_{pyr}), 135.46 (C_{pyr}), 142.57 (C_{pyr}); HRMS-ESI $[M-Cl]^+$ 284.1417 (calculated for $C_{15}H_{23}ClNO_2$, 284.1412).

5-(Chloromethyl)-2-heptyl-8-methyl-4H-[1,3]dioxino[4,5-c]pyridine hydrochloride (3m)

The target compound was synthesized according to general method 1.

Yield 100%; gray solid; mp 157 °C (dec.); ^1H NMR (DMSO-d_6, 400 MHz) δ 0.11 (t, 3H, $^3J_{HH}$ = 6.9 Hz, $\underline{CH_3}(CH_2)_6$), 0.47-0.65 (m, 8H, $CH_3\underline{(CH_2)_4}(CH_2)_2$), 0.72-0.82 (m, 2H, $CH_3(CH_2)_4\underline{CH_2}CH_2$), 1.08-1.20 (m, 2H, $CH_3(CH_2)_4CH_2\underline{CH_2}$), 1.82 (s, 3H, CH_{3pyr}), 3.95, 4.01 (AB, 2H, $^2J_{HH}$ = 12.7 Hz, CH_2), 4.46, 4.51 (AB, 2H, $^2J_{HH}$ = 17.6 Hz, CH_2), 4.54 (t, 1H, $^3J_{HH}$ = 5.1 Hz, $\underline{CH}C_7H_{15}$), 7.56 (s, 1H, CH_{pyr}); ^{13}C NMR (DMSO-d_6, 100 MHz) δ 14.01 (CH_{3pyr}), 14.84 (CH_3), 22.11 (CH_2), 22.88 (CH_2), 28.61 (CH_2), 28.63 (CH_2), 31.21 (CH_2), 33.34 (CH_2), 39.18 (CH_2Cl), 63.33 (CH_2O), 100.59 ($\underline{C}HC_7H_{15}$), 130.17 (C_{pyr}), 133.78 (C_{pyr}), 134.88 (C_{pyr}), 144.14 (C_{pyr}), 149.14 (C_{pyr}); HRMS-ESI $[M-Cl]^+$ 298.1574 (calculated for $C_{16}H_{25}ClNO_2$, 298.1568).

5-(Chloromethyl)-2-(heptan-3-yl)-8-methyl-4H-[1,3]dioxino[4,5-c]pyridine hydrochloride (mixture of two diastereomers) (3n)

The target compound was synthesized according to general method 2.

Yield 90 %; gray solid; mp 120 °C (dec.); ^1H NMR (DMSO-d_6, 400 MHz) δ 0.88 (t, 3H, $^3J_{HH}$ = 6.5 Hz, $\underline{CH_3}C_3H_6CH(CH_3CH_2)$), 0.93 (t, 3H, $^3J_{HH}$ = 7.4 Hz, $C_4H_9CH(\underline{CH_3}CH_2)$), 1.25-1.62 (m, 8H, $CH_3\underline{C_3H_6}CH(CH_3\underline{CH_2})$), 1.69-1.81 (m, 1H, $C_4H_9\underline{CH}(CH_3CH_2)$), 2.56 (s, 3H, CH_{3pyr}), 4.89, 4.94 (AB, 2H, $^2J_{HH}$ = 12.0 Hz, CH_2), 5.22, 5.33 (AB, 2H, $^2J_{HH}$ = 16.0 Hz, CH_2), 5.31 (br s, 1H, CH), 8.44 (s, 1H, CH_{pyr}); ^{13}C NMR (DMSO-d_6, 100 MHz) δ 11.20 (CH_3), 11.34 (CH_3), 13.94 (CH_3), 14.19 (CH_{3pyr}), 20.84 (CH_2), 22.47 (CH_2), 22.53 (CH_2), 27.09 (CH_2), 27.11 (CH_2), 28.60 (CH_2), 28.63 (CH_2), 38.99 (CH), 42.40 (CH_2Cl), 42.52 (CH_2Cl), 63.64 (CH_2O), 102.27 (CH), 130.62 (C_{pyr}), 132.61 (C_{pyr}), 135.93 (C_{pyr}), 143.64 (C_{pyr}), 149.62 (C_{pyr}); HRMS-ESI $[M-Cl]^+$ 298.1575 (calculated for $C_{16}H_{25}ClNO_2$, 298.1568).

5-(Chloromethyl)-2,8-dimethyl-2-octyl-4H-[1,3]dioxino[4,5-c]pyridine hydrochloride (3q)

The target compound was synthesized according to general method 1.

Yield 100%; white solid; mp 146 °C (dec.); ^1H NMR (DMSO-d_6, 400 MHz) δ 0.82 (t, 3H, $^3J_{HH}$ = 6.1 Hz, $\underline{CH_3}(CH_2)_7$), 1.17-1.29 (m, 10H, $CH_3\underline{(CH_2)_5}(CH_2)_2$), 1.36-1.44 (m, 2H,

$CH_3(CH_2)_5\underline{CH_2}CH_2$), 1.49 (3H, CH_3), 1.75-1.82 (m, 2H, $CH_3(CH_2)_6\underline{CH_2}$), 2.56 (s, 3H, CH_{3pyr}), 4.96 (s, 2H, CH_2), 5.18 (s, 2H, CH_2), 8.47 (s, 1H, CH_{pyr}); ^{13}C NMR (DMSO-*d6*, 100 MHz) δ 13.74 (CH_3), 14.03 (CH_{3pyr}), 21.77 (CH_3), 22.17 (CH_2), 22.68 (CH_2), 28.70 (CH_2), 28.91 (CH_2), 29.00 (CH_2), 31.35 (CH_2), 37.08 (CH_2), 38.86 (CH_2Cl), 57.89 (CH_2O), 103.66 ($\underline{C}(CH_3)C_8H_{17}$), 130.88 ($C_{pyr}$), 131.15 ($C_{pyr}$), 135.38 ($C_{pyr}$), 143.53 ($C_{pyr}$), 148.05 ($C_{pyr}$); HRMS-ESI $[M-Cl]^+$ 326.1887 (calculated for $C_{18}H_{29}ClNO_2$, 326.1881).

5-(Chloromethyl)-8-methyl-4H-spiro[[1,3]dioxino[4,5-c]pyridine-2,1′-cyclohexane] hydrochloride (3r)

The target compound was synthesized according to general method 1.

Yield 100%; white solid; mp 200 °C (dec.); ^1H NMR (DMSO-*d6*, 400 MHz) δ 1.40-1.87 (m, 10H, $5CH_2$), 2.56 (s, 3H, CH_{3pyr}), 4.90 (s, 2H, CH_2), 5.16 (s, 2H, CH_2), 8.42 (s, 1H, CH_{pyr}); ^{13}C NMR (DMSO-*d6*, 100 MHz) δ 14.37 (CH_{3pyr}), 21.99 (CH_3), 24.35 (CH_2), 32.66 (CH_2), 38.97 (CH_2Cl), 57.26 (CH_2O), 101.87 (C_{spiro}), 130.43 (C_{pyr}), 132.30 (C_{pyr}), 134.43 (C_{pyr}), 144.21 (C_{pyr}), 147.41 (C_{pyr}); HRMS-ESI $[M-Cl]^+$ 268.1099 (calculated for $C_{14}H_{19}ClNO_2$, 268.1099).

General method for the preparation of dichloro derivatives of acetals and ketals of pyridoxine 4

Sodium hydrogen carbonate (1.1 equiv) in 40 ml of water was added to a solution of 4.00 g (1 equiv) of compound **3a-r** in 40 ml of chloroform. The resulting reaction mixture was stirred for 10 minutes. The organic layer was separated and removed under reduced pressure. The residue was dissolved in 30 ml of chloroform and trichloroisocyanuric acid (1.1 equiv) was added. The solution was refluxed during 3 h with stirring. Then, the reaction mixture was cooled to room temperature and the formed precipitate was filtered off. The filtrate was concentrated and purified by column chromatography (eluent chloroform).

5,8-Bis(chloromethyl)-4H-[1,3]dioxino[4,5-c]pyridine (4a)

Yield 51%; beige solid; mp 70-72 °C (dec.); ^1H NMR ($CDCl_3$, 400 MHz) δ 4.46 (s, 2H, CH_2), 4.68 (s, 2H, CH_2), 5.03 (s, 2H, CH_2), 5.36 (s, 2H, CH_2), 8.15 (s, 1H, CH_{pyr}); ^{13}C NMR ($CDCl_3$, 100 MHz) δ 39.89 (CH_2Cl), 41.10 (CH_2Cl), 63.59 (CH_2O), 91.51 (OCH_2O),

129.37 (C_{pyr}), 141.71 (C_{pyr}), 142.21 (C_{pyr}), 145.71 (C_{pyr}), 147.79 (C_{pyr}); HRMS-ESI $[M+H]^+$ 234.0083 (calculated for $C_9H_{10}Cl_2NO_2$, 234.0083)

5,8-Bis(chloromethyl)-2-methyl-4H-[1,3]dioxino[4,5-c]pyridine (4b)

Yield 54%; beige solid; mp 70 °C (dec.); ^{1}H NMR ($CDCl_3$, 400 MHz) δ 1.62 (d, 3H, $^3J_{HH}$ = 5.1 Hz, CH$\underline{CH_3}$), 4.44, 4.48 (AB, 2H, $^2J_{HH}$ = 12.1 Hz, CH_2), 4.65, 4.71 (AB, 2H, $^2J_{HH}$ = 11.0 Hz, CH_2), 5.00, 5.06 (AB, 2H, $^2J_{HH}$ = 16.1 Hz, CH_2), 5.25 (q, 1H, $^3J_{HH}$ = 5.1 Hz, $\underline{CH}CH_3$), 8.13 (s, 1H, CH_{pyr}); ^{13}C NMR ($CDCl_3$, 100 MHz) δ 20.65 (CH$\underline{C}H_3$), 40.02 (CH_2Cl), 41.15 (CH_2Cl), 63.72 (CH_2O), 97.87 ($\underline{C}HCH_3$), 128.77 (C_{pyr}), 129.09 (C_{pyr}), 141.56 (C_{pyr}), 145.51 (C_{pyr}), 148.10 (C_{pyr}); HRMS-ESI $[M+H]^+$ 248.0244 (calculated for $C_{10}H_{12}Cl_2NO_2$, 248.0240).

5,8-Bis(chloromethyl)-2-ethyl-4H-[1,3]dioxino[4,5-c]pyridine (4c)

Yield 39%; yellow oil; ^{1}H NMR ($CDCl_3$, 400 MHz) δ 1.09 (t, 3H, $^3J_{HH}$ = 7.5 Hz, $CH_2\underline{CH_3}$), 1.90-1.94 (m, 2H, $\underline{CH_2}CH_3$), 4.44, 4.48 (AB, 2H, $^2J_{HH}$ = 12.0 Hz, CH_2), 4.67, 4.73 (AB, 2H, $^2J_{HH}$ = 11.0 Hz, CH_2), 5.02, 5.07 (AB, 2H, $^2J_{HH}$ = 16.3 Hz, CH_2), 5.07 (t, 1H, $^3J_{HH}$ = 5.1 Hz, $\underline{CH}C_2H_5$), 8.13 (s, 1H, CH_{pyr}); ^{13}C NMR ($CDCl_3$, 100 MHz) δ 7.79 (CH_3), 27.52 (CH_2), 40.01 (CH_2Cl), 41.06 (CH_2Cl), 63.80 (CH_2O), 101.40 ($\underline{CH}C_2H_5$), 129.16 (C_{pyr}), 129.21 (C_{pyr}), 141.24 (C_{pyr}), 145.46 (C_{pyr}), 148.28 (C_{pyr}); HRMS-ESI $[M+H]^+$ 262.0402 (calculated for $C_{11}H_{14}Cl_2NO_2$, 262.0396).

5,8-Bis(chloromethyl)-2-propyl-4H-[1,3]dioxino[4,5-c]pyridine (4d)

Yield 58%; yellow oil; ^{1}H NMR ($CDCl_3$, 400 MHz) δ 1.01 (t, 3H, $^3J_{HH}$ = 7.4 Hz, $\underline{CH_3}CH_2CH_2$), 1.51-1.64 (m, 2H, $CH_3\underline{CH_2}CH_2$), 1.82-1.96 (m, 2H, $CH_3CH_2\underline{CH_2}$), 4.44, 4.47 (AB, 2H, $^2J_{HH}$ = 12.2 Hz, CH_2), 4.66, 4.72 (AB, 2H, $^2J_{HH}$ = 11.0 Hz, CH_2), 5.00, 5.06 (AB, 2H, $^2J_{HH}$ = 16.0 Hz, CH_2), 5.10 (t, 1H, $^3J_{HH}$ = 5.2 Hz, CH), 8.13 (s, 1H, CH_{pyr}); ^{13}C NMR ($CDCl_3$, 100 MHz) δ 13.99 ($\underline{C}H_3CH_2CH_2$), 16.94 ($CH_3\underline{C}H_2CH_2$), 36.21 ($CH_3CH_2\underline{C}H_2$), 40.01 (CH_2Cl), 41.08 (CH_2Cl), 63.79 (CH_2O), 100.52 ($\underline{C}HC_3H_7$), 129.11 (C_{pyr}), 129.19 (C_{pyr}), 141.33 (C_{pyr}), 145.53 (C_{pyr}), 148.27 (C_{pyr}); HRMS-ESI $[M+H]^+$ 276.0559 (calculated for $C_{12}H_{16}Cl_2NO_2$, 276.0553).

5,8-Bis(chloromethyl)-2-isopropyl-4H-[1,3]dioxino[4,5-c]pyridine (4e)

Yield 42 %; yellow solid; mp 61-63 °C (dec.); ^{1}H NMR ($CDCl_3$, 400 MHz) δ 1.09 (d, 3H, $^3J_{HH}$ = 6.7 Hz, CH_3), 1.10 (d, 3H, $^3J_{HH}$ = 6.7 Hz, CH_3), 2.10-2.13 (m, 1H, CH$\underline{CH}(CH_3)_2$), 4.43, 4.47 (AB, 2H, $^2J_{HH}$ = 12.5 Hz, CH_2), 4.65, 4.72 (AB, 2H, $^2J_{HH}$ = 11.0 Hz, CH_2), 4.85 (d,

1H, $^{3}J_{HH}$ = 4.8 Hz, $\underline{CH}CH(CH_3)_2$), 5.04 (s, 2H, CH_2), 8.11 (s, 1H, CH_{pyr}); ^{13}C NMR ($CDCl_3$, 100 MHz) δ 16.49 (CH_3), 16.63 (CH_3), 32.41 ($CH\underline{C}H(CH_3)_2$), 40.04 (CH_2Cl), 41.27 (CH_2Cl), 63.95 (CH_2O), 103.82 ($\underline{C}HCH(CH_3)_2$), 129.08 (C_{pyr}), 129.16 (C_{pyr}), 141.17 (C_{pyr}), 145.53 (C_{pyr}), 148.42 (C_{pyr}).

2-Butyl-5,8-bis(chloromethyl)-4H-[1,3]dioxino[4,5-c]pyridine (4f)

Yield 60%; yellow oil; ^{1}H NMR ($CDCl_3$, 400 MHz) δ 0.95 (t, 3H, $^{3}J_{HH}$ = 7.2 Hz, $\underline{CH_3}(CH_2)_3$), 1.37-1.46 (m, 2H, $CH_3(\underline{CH_2})_3$), 1.49-1.57 (m, 2H, $CH_3(\underline{CH_2})_3$), 1.84-1.98 (m, 2H, $CH_3(\underline{CH_2})_3$), 4.44, 4.47 (AB, 2H, $^{2}J_{HH}$ = 12.1 Hz, CH_2), 4.67, 4.73 (AB, 2H, $^{2}J_{HH}$ = 11.0 Hz, CH_2), 5.01, 5.06 (AB, 2H, $^{2}J_{HH}$ = 16.2 Hz, CH_2), 5.09 (t, 1H, $^{3}J_{HH}$ = 5.0 Hz, CH), 8.14 (s, 1H, CH_{pyr}); ^{13}C NMR ($CDCl_3$, 100 MHz) δ 14.09 ($\underline{C}H_3(CH_2)_3$), 22.52 ($CH_3(\underline{C}H_2)_3$), 25.63 ($CH_3(\underline{C}H_2)_3$), 33.91 ($CH_3(\underline{C}H_2)_3$), 39.98 (CH_2Cl), 40.93 (CH_2Cl), 63.80 (CH_2O), 100.75 ($\underline{C}HC_4H_9$), 129.28 (C_{pyr}), 141.15 (C_{pyr}), 141.78 (C_{pyr}), 145.44 (C_{pyr}), 148.32 (C_{pyr}); HRMS-ESI $[M+H]^+$ 290.0715 (calculated for $C_{13}H_{18}Cl_2NO_2$, 290.0709).

2-(Tert-butyl)-5,8-bis(chloromethyl)-4H-[1,3]dioxino[4,5-c]pyridine (4g)

Yield 68 %; yellow oil; ^{1}H NMR ($CDCl_3$, 400 MHz) δ 1.08 (s, 9H, $3CH_3$), 4.45, 4.48 (AB, 2H, $^{2}J_{HH}$ = 12.0 Hz, CH_2), 4.67, 4.71 (AB, 2H, $^{2}J_{HH}$ = 12.0 Hz, CH_2), 4.73 (s, 1H, CH), 5.05 (s, 2H, CH_2), 8.11 (s, 1H, CH_{pyr}); ^{13}C NMR ($CDCl_3$, 100 MHz) δ 24.31 (CH_3), 35.21 ($\underline{C}(CH_3)_3$), 40.04 (CH_2Cl), 41.23 (CH_2Cl), 64.24 (CH_2O), 105.74 ($\underline{CH}C(CH_3)_3$), 129.27 (C_{pyr}), 131.30 (C_{pyr}), 140.84 (C_{pyr}), 144.22 (C_{pyr}), 150.95 (C_{pyr}); HRMS-ESI $[M+H]^+$ 290.0715 (calculated for $C_{13}H_{18}Cl_2NO_2$, 290.0709).

2-(Sec-butyl)-5,8-bis(chloromethyl)-4H-[1,3]dioxino[4,5-c]pyridine (mixture of two diastereomers) (4h)

Yield 53 %; yellow oil; ^{1}H NMR ($CDCl_3$, 400 MHz) δ 0.98 (t, 6H, $^{3}J_{HH}$ = 7.5 Hz, $2CH_3$), 1.06 (d, 3H, $^{3}J_{HH}$ = 6.9 Hz, CH_3), 1.08 (d, 3H, $^{3}J_{HH}$ = 6.8 Hz, CH_3), 1.30-1.42 (m, 2H, CH_2), 1.63-1.79 (m, 2H, CH_2), 1.85-1.97 (m, 2H, 2CH), 4.44, 4.48 (2AB, 4H, $^{2}J_{HH}$ = 12.1 Hz, $2CH_2$), 4.65, 4.71 (AB, 2H, $^{2}J_{HH}$ = 11.0 Hz, CH_2), 4.66, 4.71 (AB, 2H, $^{2}J_{HH}$ = 11.0 Hz, CH_2), 4.93-4.95 (m, 2H, 2CH), 5.02, 5.06 (2AB, 4H, $^{2}J_{HH}$ = 15.3 Hz, $2CH_2$), 8.11 (s, 2H, $2CH_{pyr}$); ^{13}C NMR ($CDCl_3$, 100 MHz) δ 11.48 (CH_3), 11.55 (CH_3), 13.06 (CH_3), 13.16 (CH_3), 23.80 (CH_2), 24.02 (CH_2), 38.79 ($CH\underline{C}H(CH_3)C_2H_5$), 38.87 ($CH\underline{C}H(CH_3)C_2H_5$), 39.99 ($CH_2Cl$), 41.12 ($CH_2Cl$), 63.97 ($CH_2O$), 103.08 ($\underline{C}HCH(CH_3)C_2H_5$), 103.18 ($\underline{C}HCH(CH_3)C_2H_5$), 129.25

(C_{pyr}), 140.96 (C_{pyr}), 145.37 (C_{pyr}), 145.42 (C_{pyr}), 148.52 (C_{pyr}); HRMS-ESI $[M+H]^+$ 290.0713 (calculated for $C_{13}H_{18}Cl_2NO_2$, 290.0709).

5,8-Bis(chloromethyl)-2-pentyl-4H-[1,3]dioxino[4,5-c]pyridine (4i)

Yield 31%; beige solid; mp 58 °C (dec.); ^1H NMR ($CDCl_3$, 400 MHz) δ 0.92 (t, 3H, $^3J_{HH}$ = 6.9 Hz, $\underline{CH_3}(CH_2)_4$), 1.31-1.41 (m, 4H, $CH_3(\underline{CH_2})_4$), 1.51-1.62 (m, 2H, $CH_3(\underline{CH_2})_4$), 1.85-1.97 (m, 2H, $CH_3(\underline{CH_2})_4$), 4.44, 4.48 (AB, 2H, $^2J_{HH}$ = 12.1 Hz, CH_2), 4.66, 4.72 (AB, 2H, $^2J_{HH}$ = 11.0 Hz, CH_2), 5.01, 5.06 (AB, 2H, $^2J_{HH}$ = 16.1 Hz, CH_2), 5.10 (t, 1H, $^3J_{HH}$ = 5.2 Hz, CH), 8.13 (s, 1H, CH_{pyr}); ^{13}C NMR ($CDCl_3$, 100 MHz) δ 14.11 ($\underline{C}H_3(CH_2)_4$), 22.65 ($CH_3(\underline{C}H_2)_4$), 23.19 ($CH_3(\underline{C}H_2)_4$), 31.57 ($CH_3(\underline{C}H_2)_4$), 34.16 ($CH_3(\underline{C}H_2)_4$), 39.99 (CH_2Cl), 41.03 (CH_2Cl), 63.81 (CH_2O), 100.75 ($\underline{C}HC_5H_{11}$), 129.23 (C_{pyr}), 141.19 (C_{pyr}), 141.41 (C_{pyr}), 145.43 (C_{pyr}), 148.31 (C_{pyr}); HRMS-ESI $[M+H]^+$ 304.0871 (calculated for $C_{14}H_{20}Cl_2NO_2$, 304.0866).

5,8-Bis(chloromethyl)-2-(pentan-2-yl)-4H-[1,3]dioxino[4,5-c]pyridine (mixture of two diastereomers) (4j)

Yield 45 %; yellow oil; ^1H NMR ($CDCl_3$, 400 MHz) δ 0.93 (t, 3H, $^3J_{HH}$ = 7.1 Hz, CH_3), 0.94 (t, 3H, $^3J_{HH}$ = 7.1 Hz, CH_3), 1.05 (d, 3H, $^3J_{HH}$ = 6.6 Hz, CH_3), 1.07 (d, 3H, $^3J_{HH}$ = 6.5 Hz, CH_3), 1.21-1.40 (m, 4H, $2CH_2$), 1.41-1.54 (m, 2H, CH_2), 1.56-1.67 (m, 2H, CH_2), 1.93-2.05 (m, 2H, 2CH), 4.42, 4.46 (2AB, 4H, $^2J_{HH}$ = 12.1 Hz, $2CH_2$), 4.64, 4.70 (AB, 2H, $^2J_{HH}$ = 11.0 Hz, CH_2), 4.65, 4.70 (AB, 2H, $^2J_{HH}$ = 11.0 Hz, CH_2), 4.92 (d, 1H, $^3J_{HH}$ = 4.1 Hz, CH), 4.93 (d, 1H, $^3J_{HH}$ = 4.1 Hz, CH), 5.00, 5.05 (2AB, 4H, $^2J_{HH}$ = 15.6 Hz, $2CH_2$), 8.09 (s, 2H, $2CH_{pyr}$); ^{13}C NMR ($CDCl_3$, 100 MHz) δ 13.52 (CH_3), 13.56 (CH_3), 14.33 (CH_3), 14.35 (CH_3), 20.08 (CH_2), 20.10 (CH_2), 33.10 (CH_2), 33.31 (CH_2), 36.94 ($CH\underline{C}H(CH_3)C_3H_7$), 36.99 ($CH\underline{C}H(CH_3)C_3H_7$), 39.98 ($CH_2Cl$), 41.17 ($CH_2Cl$), 63.95 ($CH_2O$), 103.21 ($\underline{C}HCH(CH_3)C_3H_7$), 103.27 ($\underline{C}HCH(CH_3)C_3H_7$), 129.17 ($C_{pyr}$), 129.20 ($C_{pyr}$), 141.00 ($C_{pyr}$), 145.40 ($C_{pyr}$), 145.45 ($C_{pyr}$), 148.48 ($C_{pyr}$); HRMS-ESI $[M+H]^+$ 304.0873 (calculated for $C_{14}H_{20}Cl_2NO_2$, 304.0866).

5,8-Bis(chloromethyl)-2-(pentan-3-yl)-4H-[1,3]dioxino[4,5-c]pyridine (4k)

Yield 36%; yellow oil; the substance was used in the next reaction without identification by NMR spectra; HRMS-ESI $[M+H]^+$ 304.0871 (calculated for $C_{14}H_{20}Cl_2NO_2$, 304.0866).

5,8-Bis(chloromethyl)-2-hexyl-4H-[1,3]dioxino[4,5-c]pyridine (4l)

Yield 42%; beige solid; mp 71 °C (dec.); ^{1}H NMR ($CDCl_3$, 400 MHz) δ 0.90 (t, 3H, $^3J_{HH}$ = 6.7 Hz, CH_3), 1.23-1.40 (m, 6H, $3CH_2$), 1.50-1.56 (m, 2H, CH_2), 1.87-2.04 (m, 2H, CH_2), 4.44, 4.48 (AB, 2H, $^2J_{HH}$ = 12.0 Hz, CH_2), 4.67, 4.73 (AB, 2H, $^2J_{HH}$ = 11.1 Hz, CH_2), 5.01, 5.06 (AB, 2H, $^2J_{HH}$ = 16.2 Hz, CH_2), 5.10 (t, 1H, $^3J_{HH}$ = 4.7 Hz, $\underline{CH}(C_6H_{13})$), 8.04 (s, 1H, CH_{pyr}); ^{13}C NMR ($CDCl_3$, 100 MHz) δ 14.21 (CH_3), 22.69 (CH_2), 23.48 (CH_2), 29.09 (CH_2), 31.81 (CH_2), 34.20 (CH_2), 39.97 (CH_2Cl), 40.87 (CH_2Cl), 63.81 (CH_2O), 100.78 ($\underline{C}HC_6H_{13}$), 129.32 (C_{pyr}), 129.40 (C_{pyr}), 141.04 (C_{pyr}), 145.36 (C_{pyr}), 148.37 (C_{pyr}); HRMS-ESI $[M+H]^+$ 318.1027 (calculated for $C_{15}H_{22}Cl_2NO_2$, 318.1022).

5,8-Bis(chloromethyl)-2-heptyl-4H-[1,3]dioxino[4,5-c]pyridine (4m)

Yield 56 %; beige solid; mp 94 °C (dec.); ^{1}H NMR ($CDCl_3$, 400 MHz) δ 0.89 (t, 3H, $^3J_{HH}$ = 6.7 Hz, CH_3), 1.24-1.42 (m, 8H, $4CH_2$), 1.51-1.56 (m, 2H, CH_2), 1.84-1.96 (m, 2H, CH_2), 4.44, 4.48 (AB, 2H, $^2J_{HH}$ = 12.1 Hz, CH_2), 4.66, 4.73 (AB, 2H, $^2J_{HH}$ = 11.0 Hz, CH_2), 5.01, 5.06 (AB, 2H, $^2J_{HH}$ = 16.1 Hz, CH_2), 5.10 (t, 1H, $^3J_{HH}$ = 5.1 Hz, $\underline{CH}C_7H_{15}$), 8.13 (s, 1H, CH_{pyr}); ^{13}C NMR ($CDCl_3$, 100 MHz) δ 14.25 (CH_3), 22.78 (CH_2), 23.54 (CH_2), 29.29 (CH_2), 29.39 (CH_2), 31.88 (CH_2), 34.22 (CH_2), 40.02 (CH_2Cl), 41.11 (CH_2Cl), 63.82 (CH_2O), 100.75 ($\underline{C}HC_7H_{15}$), 129.15 (C_{pyr}), 129.20 (C_{pyr}), 141.26 (C_{pyr}), 145.47 (C_{pyr}), 148.30 (C_{pyr}); HRMS-ESI $[M+H]^+$ 332.1184 (calculated for $C_{16}H_{24}Cl_2NO_2$, 332.1179).

5,8-Bis(chloromethyl)-2-(heptan-3-yl)-4H-[1,3]dioxino[4,5-c]pyridine (mixture of two diastereomers) (4n)

Yield 55 %; yellow oil; ^{1}H NMR ($CDCl_3$, 400 MHz) δ 0.88-0.94 (m, 6H, $2CH_3$), 0.98 (t, 6H, $^3J_{HH}$ = 7.5 Hz, $2CH_3$), 1.26-1.71 (m, 16H, $8CH_2$), 1.75-1.83 (m, 2H, 2CH), 4.44, 4.48 (2AB, 4H, $^2J_{HH}$ = 12.1 Hz, $2CH_2$), 4.65, 4.71 (2AB, 4H, $^2J_{HH}$ = 11.0 Hz, $2CH_2$), 5.00-5.08 (m, 6H, $2CH_2$ +2CH), 8.11 (s, 2H, $2CH_{pyr}$); ^{13}C NMR ($CDCl_3$, 100 MHz) δ 11.52 (CH_3), 11.68 (CH_3), 14.19 (CH_3), 21.31 (CH_2), 21.48 (CH_2), 23.15 (CH_2), 23.20 (CH_2), 27.76 (CH_2), 27.80 (CH_2), 29.33 (CH_2), 29.40 (CH_2), 40.01 (CH_2Cl), 41.12 (CH_2Cl), 43.38 ($CH\underline{C}H(C_2H_5)C_4H_9$), 43.47 ($CH\underline{C}H(C_2H_5)C_4H_9$), 64.07 ($CH_2O$), 102.42 ($\underline{C}HCH(C_2H_5)C_4H_9$), 102.47 ($\underline{C}HCH(C_2H_5)C_4H_9$), 129.29 ($C_{pyr}$), 129.39 ($C_{pyr}$), 140.92 ($C_{pyr}$), 145.44 ($C_{pyr}$), 148.64 ($C_{pyr}$); HRMS-ESI $[M+H]^+$ 332.1185 (calculated for $C_{16}H_{24}Cl_2NO_2$, 332.1179).

5,8-Bis(chloromethyl)-2-octyl-4H-[1,3]dioxino[4,5-c]pyridine (4o)

Yield 51%; white solid; mp 84-86 °C (dec.); ^{1}H NMR ($CDCl_3$, 400 MHz) δ 0.89 (t, 3H, $^{3}J_{HH}$ = 6.7 Hz, CH_3), 1.24-1.44 (m, 10H, 5CH_2), 1.51-1.59 (m, 2H, CH_2), 1.87-1.94 (m, 2H, CH_2), 4.44, 4.48 (AB, 2H, $^{2}J_{HH}$ = 12.0 Hz, CH_2), 4.64, 4.73 (AB, 2H, $^{2}J_{HH}$ = 11.0 Hz, CH_2), 5.01, 5.06 (AB, 2H, $^{2}J_{HH}$ = 16.1 Hz, CH_2), 5.10 (t, 1H, $^{3}J_{HH}$ = 5.1 Hz, $\underline{CH}C_8H_{17}$), 8.13 (s, 1H, CH_{pyr}); ^{13}C NMR ($CDCl_3$, 100 MHz) δ 14.26 (CH_3), 22.82 (CH_2), 23.55 (CH_2), 29.35 (CH_2), 29.45 (CH_2), 29.60 (CH_2), 32.00 (CH_2), 34.25 (CH_2), 40.06 (CH_2Cl), 41.27 (CH_2Cl), 63.83 (CH_2O), 100.73 ($\underline{C}HC_8H_{17}$), 128.97 (C_{pyr}), 129.12 (C_{pyr}), 141.47 (C_{pyr}), 145.48 (C_{pyr}), 148.26 (C_{pyr}).

5,8-Bis(chloromethyl)-2-(undecan-2-yl)-4H-[1,3]dioxino[4,5-c]pyridine (mixture of two diastereomers) (4p)

Yield 29%; yellow oil; ^{1}H NMR ($CDCl_3$, 400 MHz) δ 0.87 (t, 6H, $^{3}J_{HH}$ = 6.6 Hz, 2CH_3), 1.07 (t, 6H, $^{3}J_{HH}$ = 6.5 Hz, 2CH_3), 1.19-1.44 (m, 32H, 16CH_2), 1.62-1.67 (m, 1H, $CH\underline{CH}(CH_3)C_9H_{19}$), 1.96-1.98 (m, 1H, $CH\underline{CH}(CH_3)C_9H_{19}$), 4.43, 4.47 (2AB, 4H, $^{2}J_{HH}$ = 12.0 Hz, 2CH_2), 4.64, 4.70 (AB, 2H, $^{2}J_{HH}$ = 11.0 Hz, CH_2), 4.65, 4.70 (AB, 2H, $^{2}J_{HH}$ = 11.0 Hz, CH_2), 4.93 (d, 2H, $^{3}J_{HH}$ = 4.3 Hz, 2$\underline{CH}CH(CH_3)C_9H_{19}$), 5.03 (s, 2H, CH_2), 5.04 (s, 2H, CH_2), 8.10 (s, 2H, 2CH_{pyr}); ^{13}C NMR ($CDCl_3$, 100 MHz) δ 13.60 (CH_3), 13.65 (CH_3), 14.26 (CH_3), 22.81 (CH_2), 26.97 (CH_2), 27.01 (CH_2), 29.46 (CH_2), 29.72 (CH_2), 29.93 (CH_2), 29.94 (CH_2), 29.95 (CH_2), 30.94 (CH_2), 31.13 (CH_2), 32.02 (CH_2), 37.21 ($CH\underline{C}H(CH_3)C_9H_{19}$), 37.27 ($CH\underline{C}H(CH_3)C_9H_{19}$), 40.04 ($CH_2Cl$), 41.30 ($CH_2Cl$), 63.98 ($CH_2O$), 103.18 ($\underline{C}HCH(CH_3)C_9H_{19}$), 103.27 ($\underline{C}HCH(CH_3)C_9H_{19}$), 129.05 ($C_{pyr}$), 129.14 ($C_{pyr}$), 141.16 ($C_{pyr}$), 145.52 ($C_{pyr}$), 145.57 ($C_{pyr}$), 148.47 ($C_{pyr}$).

5,8-Bis(chloromethyl)-2-methyl-2-octyl-4H-[1,3]dioxino[4,5-c]pyridine (4q)

Yield 39%; yellow oil; ^{1}H NMR ($CDCl_3$, 400 MHz) δ 0.87 (t, 3H, $^{3}J_{HH}$ = 6.6 Hz, CH_3), 1.23-1.33 (m, 10H, 5CH_2), 1.43-1.56 (m, 5H, CH_3 + CH_2), 1.80-184 (m, 2H, CH_2), 4.47 (s, 2H, CH_2), 4.67 (s, 2H, CH_2), 4.94 (s, 2H, CH_2), 8.10 (s, 1H, CH_{pyr}); ^{13}C NMR ($CDCl_3$, 100 MHz) δ 14.23 (CH_3), 22.10 (CH_2), 22.77 (CH_2), 23.28 (CH_2), 29.31 (CH_2), 29.58 (CH_2), 29.74 (CH_2), 31.95 (CH_2), 38.02 (CH_2), 40.04 (CH_2Cl), 41.38 (CH_2Cl), 57.98 (CH_2O), 102.55 ($\underline{C}(CH_3)C_8H_{17}$), 127.71 ($C_{pyr}$), 128.86 ($C_{pyr}$), 140.53 ($C_{pyr}$), 145.79 ($C_{pyr}$), 146.63 ($C_{pyr}$); HRMS-ESI $[M+H]^+$ 360.1499 (calculated for $C_{18}H_{28}Cl_2NO_2$, 360.1492).

5,8-Bis(chloromethyl)-4H-spiro[[1,3]dioxino[4,5-c]pyridine-2,1′-cyclohexane] (4r)

Yield 36%; brown oil; ^{1}H NMR ($CDCl_3$, 400 MHz) δ 1.42-1.93 (m, 10H, 5CH_2), 4.46 (s, 2H, CH_2), 4.68 (s, 2H, CH_2), 4.94 (s, 2H, CH_2), 8.09 (s, 1H, CH_{pyr}).

General procedure for preparation of quaternary ammonium salts $5a_8$-$5r_{18}$

Amine (2 equiv) was added to a solution of compound **4a-4r** (1 equiv) in 10 ml of ethanol. The reaction mixture was heated at 70 °C for 5h, then the solvent was evaporated under reduced pressure. The oily residue was refluxed in acetone for 1h and the crystalline precipitate was filtered and dried under reduced pressure.

5,8-Bis((*N*,*N*-dimethyl-*N*-(octyl)ammonio)methyl)-4*H*-[1,3]dioxino[4,5-*c*]pyridine dichloride ($5a_8$)

Yield 52%; white solid; mp 123 °C (dec.); ^{1}H NMR ($CDCl_3$, 400 MHz) δ 0.85-0.89 (m, 6H, 2$\underline{CH_3}C_7H_{14}$), 1.16-1.42 (m, 20H, 10$CH_2$), 1.70-1.90 (m, 4H, 2$CH_2$), 3.31 (s, 6H, 2$CH_3N^+$), 3.34 (s, 6H, 2$CH_3N^+$), 3.55-3.59 (m, 2H, CH_2N^+), 3.74-3.78 (m, 2H, CH_2N^+), 4.71 (s, 2H, CH_2), 5.19 (s, 2H, CH_2), 5.31 (s, 2H, CH_2), 5.49 (s, 2H, CH_2), 8.65 (s, 1H, CH_{pyr}); ^{13}C NMR ($CDCl_3$, 100 MHz) δ 14.21 (CH_3), 22.73 (CH_2), 23.19 (CH_2), 23.20 (CH_2), 26.46 (CH_2), 26.48 (CH_2), 29.20 (CH_2), 29.41 (CH_2), 29.48 (CH_2), 31.80 (CH_2), 49.65 (CH_3N^+), 51.37 (CH_3N^+), 62.23 (CH_2), 65.37 (CH_2), 65.61 (CH_2), 66.19 (CH_2), 92.13 (OCH_2O), 122.88 (C_{pyr}), 134.44 (C_{pyr}), 137.48 (C_{pyr}), 147.00 (C_{pyr}), 150.02 (C_{pyr}); HRMS-ESI $[M-2Cl]^{2+}$ 238.7147 (calculated for $C_{29}H_{55}N_3O_2$, 238.7142).

5,8-Bis((*N*,*N*-dimethyl-*N*-(decyl)ammonio)methyl)-4*H*-[1,3]dioxino[4,5-*c*]pyridine dichloride ($5a_{10}$)

Yield 53%; white solid; mp 127-128 °C (dec.); ^{1}H NMR ($CDCl_3$, 400 MHz) δ 0.86 (t, 6H, $^3J_{HH}$ = 6.1 Hz, 2$\underline{CH_3}C_9H_{18}$), 1.16-1.45 (m, 28H, 14$CH_2$), 1.70-1.87 (m, 4H, 2$CH_2$), 3.30 (s, 6H, 2$CH_3N^+$), 3.33 (s, 6H, 2$CH_3N^+$), 3.55-3.59 (m, 2H, CH_2N^+), 3.73-3.77 (m, 2H, CH_2N^+), 4.71 (s, 2H, CH_2), 5.18 (s, 2H, CH_2), 5.30 (s, 2H, CH_2), 5.48 (s, 2H, CH_2), 8.65 (s, 1H, CH_{pyr}); ^{13}C NMR ($CDCl_3$, 100 MHz) δ 14.23 (CH_3), 22.78 (CH_2), 23.19 (CH_2), 26.46 (CH_2), 26.49 (CH_2), 29.38 (CH_2), 29.47 (CH_2), 29.56 (CH_2), 29.58 (CH_2), 31.96 (CH_2), 49.62 (CH_3N^+), 51.31 (CH_3N^+), 62.20 (CH_2), 65.35 (CH_2), 65.53 (CH_2), 66.14 (CH_2), 92.11 (OCH_2O), 122.88 (C_{pyr}), 134.42 (C_{pyr}), 137.49 (C_{pyr}), 146.98 (C_{pyr}), 149.99 (C_{pyr}); HRMS-ESI $[M-2Cl]^{2+}$ 266.7460 (calculated for $C_{33}H_{63}N_3O_2$, 266.7455).

5,8-Bis((*N*,*N*-dimethyl-*N*-(dodecyl)ammonio)methyl)-4*H*-[1,3]dioxino[4,5-*c*]pyridine dichloride (5a₁₂)

Yield 46%; white solid; mp 158-160 °C (dec.); ^{1}H NMR ($CDCl_3$, 400 MHz) δ 0.81-0.90 (m, 6H, 2$\underline{CH_3}C_{11}H_{22}$), 1.15-1.45 (m, 36H, 18$CH_2$), 1.68-1.88 (m, 4H, 2$CH_2$), 3.30 (s, 6H, 2$CH_3N^+$), 3.32 (s, 6H, 2$CH_3N^+$), 3.54-3.58 (m, 2H, CH_2N^+), 3.72-3.76 (m, 2H, CH_2N^+), 4.70 (s, 2H, CH_2), 5.17 (s, 2H, CH_2), 5.29 (s, 2H, CH_2), 5.47 (s, 2H, CH_2), 8.64 (s, 1H, CH_{pyr}); ^{13}C NMR ($CDCl_3$, 100 MHz) δ 14.24 (CH_3), 22.79 (CH_2), 23.18 (CH_2), 26.46 (CH_2), 26.49 (CH_2), 29.45 (CH_2), 29.49 (CH_2), 29.57 (CH_2), 29.64 (CH_2), 29.73 (CH_2), 32.01 (CH_2), 49.58 (CH_3N^+), 51.27 (CH_3N^+), 62.16 (CH_2), 65.32 (CH_2), 65.42 (CH_2), 66.06 (CH_2), 92.09 (OCH_2O), 122.87 (C_{pyr}), 134.40 (C_{pyr}), 137.48 (C_{pyr}), 146.95 (C_{pyr}), 149.96 (C_{pyr}); HRMS-ESI [M-2Cl]$^{2+}$ 294.7772 (calculated for $C_{37}H_{71}N_3O_2$, 294.7768).

5,8-Bis((*N*,*N*-dimethyl-*N*-(tetradecyl)ammonio)methyl)-4*H*-[1,3]dioxino[4,5-*c*]pyridine dichloride (5a₁₄)

Yield 53%; white solid; mp 169-171 °C (dec.); ^{1}H NMR ($CDCl_3$, 400 MHz) δ 0.87 (t, 6H, $^3J_{HH}$ = 6.7 Hz, 2$\underline{CH_3}C_{13}H_{26}$), 1.14-1.45 (m, 44H, 22$CH_2$), 1.70-1.90 (m, 4H, 2$CH_2$), 3.30 (s, 6H, 2$CH_3N^+$), 3.32 (s, 6H, 2$CH_3N^+$), 3.56-3.60 (m, 2H, CH_2N^+), 3.73-3.77 (m, 2H, CH_2N^+), 4.67 (s, 2H, CH_2), 5.07 (s, 2H, CH_2), 5.30 (s, 2H, CH_2), 5.50 (s, 2H, CH_2), 8.59 (s, 1H, CH_{pyr}); ^{13}C NMR ($CDCl_3$, 100 MHz) δ 14.28 (CH_3), 22.84 (CH_2), 23.24 (CH_2), 26.51 (CH_2), 26.54 (CH_2), 29.52 (CH_2), 29.54 (CH_2), 29.60 (CH_2), 29.65 (CH_2), 29.72 (CH_2), 29.81 (CH_2), 29.83 (CH_2), 29.85 (CH_2), 32.07 (CH_2), 49.81 (CH_3N^+), 51.36 (CH_3N^+), 61.90 (CH_2), 65.29 (CH_2), 65.57 (CH_2), 66.17 (CH_2), 92.18 (OCH_2O), 122.93 (C_{pyr}), 134.65 (C_{pyr}), 137.42 (C_{pyr}), 146.65 (C_{pyr}), 150.19 (C_{pyr}); HRMS-ESI [M-2Cl]$^{2+}$ 322.8084 (calculated for $C_{41}H_{79}N_3O_2$, 322.8081).

5,8-Bis((*N*,*N*-dimethyl-*N*-(hexadecyl)ammonio)methyl)-4*H*-[1,3]dioxino[4,5-*c*]pyridine dichloride (5a₁₆)

Yield 52%; white solid; mp 153-155 °C (dec.); ^{1}H NMR ($CDCl_3$, 400 MHz) δ 0.86 (t, 6H, $^3J_{HH}$ = 6.7 Hz, 2$\underline{CH_3}C_{15}H_{30}$), 1.15-1.44 (m, 52H, 26$CH_2$), 1.71-1.89 (m, 4H, 2$CH_2$), 3.30 (s, 6H, 2$CH_3N^+$), 3.33 (s, 6H, 2$CH_3N^+$), 3.55-3.58 (m, 2H, CH_2N^+), 3.73-3.77 (m, 2H, CH_2N^+), 4.71 (s, 2H, CH_2), 5.16 (s, 2H, CH_2), 5.30 (s, 2H, CH_2), 5.48 (s, 2H, CH_2), 8.64 (s, 1H, CH_{pyr}); ^{13}C NMR ($CDCl_3$, 100 MHz) δ 14.26 (CH_3), 22.82 (CH_2), 23.20 (CH_2), 26.48

(CH_2), 26.50 (CH_2), 29.50 (CH_2), 29.59 (CH_2), 29.68 (CH_2), 29.78 (CH_2), 29.81 (CH_2), 29.84 (CH_2), 32.05 (CH_2), 49.65 (CH_3N^+), 51.31 (CH_3N^+), 62.23 (CH_2), 65.33 (CH_2), 65.56 (CH_2), 66.18 (CH_2), 92.12 (OCH_2O), 122.87 (C_{pyr}), 134.43 (C_{pyr}), 137.48 (C_{pyr}), 146.97 (C_{pyr}), 150.04 (C_{pyr}); HRMS-ESI [M-2Cl]$^{2+}$ 350.8398 (calculated for $C_{45}H_{87}N_3O_2$, 350.8394).

5,8-Bis((*N*,*N*-dimethyl-*N*-(octadecyl)ammonio)methyl)-4*H*-[1,3]dioxino[4,5-*c*]pyridine dichloride (5a18)

Yield 46%; white solid; mp 165-167 °C (dec.); ^{1}H NMR ($CDCl_3$, 400 MHz) δ 0.87 (t, 6H, $^3J_{HH}$ = 6.6 Hz, 2$\underline{CH_3}C_{17}H_{34}$), 1.15-1.45 (m, 60H, 30$CH_2$), 1.70-1.89 (m, 4H, 2$CH_2$), 3.32 (s, 6H, 2$CH_3N^+$), 3.35 (s, 6H, 2$CH_3N^+$), 3.53-3.57 (m, 2H, CH_2N^+), 3.75-3.79 (m, 2H, CH_2N^+), 4.70 (s, 2H, CH_2), 5.18 (s, 2H, CH_2), 5.31 (s, 2H, CH_2), 5.50 (s, 2H, CH_2), 8.63 (s, 1H, CH_{pyr}); ^{13}C NMR ($CDCl_3$, 100 MHz) δ 14.29 (CH_3), 22.84 (CH_2), 23.21 (CH_2), 23.24 (CH_2), 26.48 (CH_2), 29.52 (CH_2), 29.57 (CH_2), 29.67 (CH_2), 29.78 (CH_2), 29.82 (CH_2), 29.86 (CH_2), 32.07 (CH_2), 49.75 (CH_3N^+), 51.44 (CH_3N^+), 62.31 (CH_2), 65.39 (CH_2), 65.83 (CH_2), 66.34 (CH_2), 92.15 (OCH_2O), 122.84 (C_{pyr}), 134.47 (C_{pyr}), 137.38 (C_{pyr}), 147.01 (C_{pyr}), 150.14 (C_{pyr}); HRMS-ESI [M-2Cl]$^{2+}$ 387.8710 (calculated for $C_{49}H_{95}N_3O_2$, 387.8707).

5,8-Bis((*N*,*N*-dimethyl-*N*-(octyl)ammonio)methyl)-2-methyl-4*H*-[1,3]dioxino[4,5-*c*]pyridine dichloride (5b8)

Yield 61%; white solid; mp 156-157 °C (dec.); ^{1}H NMR ($CDCl_3$, 400 MHz) δ 0.83-0.92 (m, 6H, 2$\underline{CH_3}C_7H_{14}$), 1.17-1.47 (m, 20H, 10$CH_2$), 1.52 (d, 3H, $^3J_{HH}$ = 4.7 Hz, CH_3), 1.69-1.94 (m, 4H, 2CH_2), 3.32 (s, 6H, 2CH_3N^+), 3.35 (s, 6H, 2CH_3N^+), 3.46-3.75 (m, 3H, 2CH_2N^+), 3.80-3.92 (m, 1H, CH_2N^+), 4.68, 4.73 (AB, 2H, $^2J_{HH}$ = 12.8 Hz, CH_2), 5.04, 5.26 (AB, 2H, $^2J_{HH}$ = 13.3 Hz, CH_2), 5.14, 5.49 (AB, 2H, $^2J_{HH}$ = 16.2 Hz, CH_2), 5.65 (q, 1H, $^3J_{HH}$ = 4.7 Hz, CH), 8.61 (s, 1H, CH_{pyr}); ^{13}C NMR ($CDCl_3$, 100 MHz) δ 14.21 (CH_3), 20.69 (CH_3), 22.72 (CH_2), 23.17 (CH_2), 23.21 (CH_2), 26.45 (CH_2), 29.20 (CH_2), 29.41 (CH_2), 29.46 (CH_2), 31.79 (CH_2), 49.63 (CH_3N^+), 49.85 (CH_3N^+), 51.39 (CH_3N^+), 62.29 (CH_2), 62.37 (CH_2), 65.56 (CH_2), 65.74 (CH_2), 66.21 (CH_2), 98.54 ($\underline{C}HCH_3$), 122.68 (C_{pyr}), 134.00 (C_{pyr}), 137.14 (C_{pyr}), 146.87 (C_{pyr}), 150.78 (C_{pyr}); HRMS-ESI [M-2Cl]$^{2+}$ 245.7226 (calculated for $C_{30}H_{57}N_3O_2$, 245.7220).

5,8-Bis((*N*,*N*-dimethyl-*N*-(decyl)ammonio)methyl)-2-methyl-4*H*-[1,3]dioxino[4,5-*c*]pyridine dichloride (5b10)

Yield 52%; white solid; mp 153-154 °C (dec.); ^{1}H NMR ($CDCl_3$, 400 MHz) δ 0.86 (t, 6H, $^3J_{HH}$ = 6.3 Hz, 2$\underline{CH_3}C_9H_{18}$), 1.14-1.45 (m, 28H, 14$CH_2$), 1.50 (d, 3H, $^3J_{HH}$ = 4.6 Hz, CH_3), 1.68-1.91 (m, 4H, 2CH_2), 3.31 (s, 6H, 2CH_3N^+), 3.34 (s, 6H, 2CH_3N^+), 3.48-3.72 (m, 3H, 2CH_2N^+), 3.77-3.91 (m, 1H, CH_2N^+), 4.69, 4.73 (AB, 2H, $^2J_{HH}$ = 13.6 Hz, CH_2), 5.06, 5.28 (AB, 2H, $^2J_{HH}$ = 13.2 Hz, CH_2), 5.12, 5.51 (AB, 2H, $^2J_{HH}$ = 16.2 Hz, CH_2), 5.63 (q, 1H, $^3J_{HH}$ = 4.6 Hz, CH), 8.63 (s, 1H, CH_{pyr}); ^{13}C NMR ($CDCl_3$, 100 MHz) δ 14.24 (CH_3), 20.68 (CH_3), 22.78 (CH_2), 23.17 (CH_2), 23.21 (CH_2), 26.46 (CH_2), 29.37 (CH_2), 29.47 (CH_2), 29.53 (CH_2), 29.55 (CH_2), 31.95 (CH_2), 49.55 (CH_3N^+), 49.78 (CH_3N^+), 51.36 (CH_3N^+), 62.24 (CH_2), 62.33 (CH_2), 65.59 (CH_2), 65.62 (CH_2), 66.12 (CH_2), 98.51 ($\underline{C}HCH_3$), 122.68 (C_{pyr}), 133.97 (C_{pyr}), 137.19 (C_{pyr}), 146.91 (C_{pyr}), 150.71 (C_{pyr}); HRMS-ESI [M-2Cl]$^{2+}$ 273.7538 (calculated for $C_{34}H_{65}N_3O_2$, 273.7538).

5,8-Bis((*N*,*N*-dimethyl-*N*-(dodecyl)ammonio)methyl)-2-methyl-4*H*-[1,3]dioxino[4,5-*c*]pyridine dichloride (5b$_{12}$)

Yield 54%; white solid; mp 174-177 °C (dec.); ^{1}H NMR ($CDCl_3$, 400 MHz) δ 0.87 (t, 6H, $^3J_{HH}$ = 6.7 Hz, 2$\underline{CH_3}C_{11}H_{22}$), 1.14-1.45 (m, 36H, 18$CH_2$), 1.52 (d, 3H, $^3J_{HH}$ = 5.0 Hz, CH_3), 1.69-1.92 (m, 4H, 2CH_2), 3.32 (s, 6H, 2CH_3N^+), 3.35 (s, 6H, 2CH_3N^+), 3.46-3.74 (m, 3H, 2CH_2N^+), 3.77-3.93 (m, 1H, CH_2N^+), 4.68, 4.73 (AB, 2H, $^2J_{HH}$ = 12.7 Hz, CH_2), 5.04, 5.27 (AB, 2H, $^2J_{HH}$ = 13.3 Hz, CH_2), 5.14, 5.49 (AB, 2H, $^2J_{HH}$ = 16.2 Hz, CH_2), 5.65 (q, 1H, $^3J_{HH}$ = 5.0 Hz, CH), 8.61 (s, 1H, CH_{pyr}); ^{13}C NMR ($CDCl_3$, 100 MHz) δ 14.27 (CH_3), 20.69 (CH_3), 22.82 (CH_2), 23.19 (CH_2), 23.24 (CH_2), 26.48 (CH_2), 29.47 (CH_2), 29.55 (CH_2), 29.57 (CH_2), 29.64 (CH_2), 29.74 (CH_2), 32.04 (CH_2), 49.64 (CH_3N^+), 49.87 (CH_3N^+), 51.40 (CH_3N^+), 62.35 (CH_2), 62.39 (CH_2), 65.57 (CH_2), 65.77 (CH_2), 66.23 (CH_2), 98.54 ($\underline{C}HCH_3$), 122.67 (C_{pyr}), 134.00 (C_{pyr}), 137.16 (C_{pyr}), 146.88 (C_{pyr}), 150.78 (C_{pyr}); HRMS-ESI [M-2Cl]$^{2+}$ 301.7855 (calculated for $C_{38}H_{73}N_3O_2$, 301.7846).

5,8-Bis((*N*,*N*-dimethyl-*N*-(tetradecyl)ammonio)methyl)-2-methyl-4*H*-[1,3]dioxino[4,5-*c*]pyridine dichloride (5b$_{14}$)

Yield 55%; white solid; mp 178-180 °C (dec.); ^{1}H NMR ($CDCl_3$, 400 MHz) δ 0.87 (t, 6H, $^3J_{HH}$ = 6.4 Hz, 2$\underline{CH_3}C_{13}H_{26}$), 1.12-1.45 (m, 44H, 22$CH_2$), 1.53 (d, 3H, $^3J_{HH}$ = 4.2 Hz, CH_3), 1.69-1.92 (m, 4H, 2CH_2), 3.32 (s, 6H, 2CH_3N^+), 3.36 (s, 6H, 2CH_3N^+), 3.46-3.74 (m, 3H, 2CH_2N^+), 3.77-3.93 (m, 1H, CH_2N^+), 4.62, 4.73 (AB, 2H, $^2J_{HH}$ = 12.5 Hz, CH_2), 5.00, 5.24

(AB, 2H, $^2J_{HH}$ = 13.2 Hz, CH_2), 5.15, 5.47 (AB, 2H, $^2J_{HH}$ = 16.2 Hz, CH_2), 5.67 (q, 1H, $^3J_{HH}$ = 4.2 Hz, CH), 8.58 (s, 1H, CH_{pyr}); ^{13}C NMR ($CDCl_3$, 100 MHz) δ 14.27 (CH_3), 20.69 (CH_3), 22.82 (CH_2), 23.19 (CH_2), 23.23 (CH_2), 26.48 (CH_2), 29.50 (CH_2), 29.57 (CH_2), 29.66 (CH_2), 29.76 (CH_2), 29.79 (CH_2), 29.82 (CH_2), 32.05 (CH_2), 49.59 (CH_3N^+), 49.80 (CH_3N^+), 51.32 (CH_3N^+), 62.31 (CH_2), 62.39 (CH_2), 65.55 (CH_2), 66.09 (CH_2), 66.24 (CH_2), 98.52 ($\underline{C}HCH_3$), 122.71 (C_{pyr}), 133.97 (C_{pyr}), 137.17 (C_{pyr}), 146.86 (C_{pyr}), 150.70 (C_{pyr}); HRMS-ESI $[M-2Cl]^{2+}$ 329.8169 (calculated for $C_{42}H_{81}N_3O_2$, 329.8159).

5,8-Bis((*N*,*N*-dimethyl-*N*-(hexadecyl)ammonio)methyl)-2-methyl-4*H*-[1,3]dioxino[4,5-*c*]pyridine dichloride (5b$_{16}$)

Yield 51%; white solid; mp 179-180 °C (dec.); ^{1}H NMR ($CDCl_3$, 400 MHz) δ 0.87 (t, 6H, $^3J_{HH}$ = 6.7 Hz, 2$\underline{CH_3}C_{15}H_{30}$), 1.16-1.45 (m, 52H, 26$CH_2$), 1.52 (d, 3H, $^3J_{HH}$ = 4.9 Hz, CH_3), 1.67-1.91 (m, 4H, 2CH_2), 3.32 (s, 6H, 2CH_3N^+), 3.36 (s, 6H, 2CH_3N^+), 3.45-3.74 (m, 3H, 2CH_2N^+), 3.79-3.93 (m, 1H, CH_2N^+), 4.67, 4.74 (AB, 2H, $^2J_{HH}$ = 12.7 Hz, CH_2), 5.03, 5.28 (AB, 2H, $^2J_{HH}$ = 13.3 Hz, CH_2), 5.14, 5.49 (AB, 2H, $^2J_{HH}$ = 16.2 Hz, CH_2), 5.65 (q, 1H, $^3J_{HH}$ = 4.9 Hz, CH), 8.61 (s, 1H, CH_{pyr}); ^{13}C NMR ($CDCl_3$, 100 MHz) δ 14.28 (CH_3), 20.70 (CH_3), 22.83 (CH_2), 23.20 (CH_2), 23.24 (CH_2), 26.48 (CH_2), 29.51 (CH_2), 29.56 (CH_2), 29.66 (CH_2), 29.77 (CH_2), 29.81 (CH_2), 29.84 (CH_2), 32.06 (CH_2), 49.66 (CH_3N^+), 49.88 (CH_3N^+), 51.41 (CH_3N^+), 62.37 (CH_2), 62.40 (CH_2), 65.58 (CH_2), 65.82 (CH_2), 66.25 (CH_2), 98.54 ($\underline{C}HCH_3$), 122.66 (C_{pyr}), 134.01 (C_{pyr}), 137.14 (C_{pyr}), 146.88 (C_{pyr}), 150.80 (C_{pyr}); HRMS-ESI $[M-2Cl]^{2+}$ 357.8472 (calculated for $C_{46}H_{89}N_3O_2$, 357.8472).

5,8-Bis((*N*,*N*-dimethyl-*N*-(octadecyl)ammonio)methyl)-2-methyl-4*H*-[1,3]dioxino[4,5-*c*]pyridine dichloride (5b$_{18}$)

Yield 47%; white solid; mp 178-180 °C (dec.); ^{1}H NMR ($CDCl_3$, 400 MHz) δ 0.87 (t, 6H, $^3J_{HH}$ = 6.7 Hz, 2$\underline{CH_3}C_{17}H_{34}$), 1.14-1.46 (m, 60H, 30$CH_2$), 1.53 (d, 3H, $^3J_{HH}$ = 5.0 Hz, CH_3), 1.68-1.93 (m, 4H, 2CH_2), 3.32 (s, 6H, 2CH_3N^+), 3.36 (s, 6H, 2CH_3N^+), 3.44-3.76 (m, 3H, 2CH_2N^+), 3.80-3.92 (m, 1H, CH_2N^+), 4.65, 4.73 (AB, 2H, $^2J_{HH}$ = 12.6 Hz, CH_2), 5.01, 5.26 (AB, 2H, $^2J_{HH}$ = 13.3 Hz, CH_2), 5.15, 5.48 (AB, 2H, $^2J_{HH}$ = 16.2 Hz, CH_2), 5.66 (q, 1H, $^3J_{HH}$ = 5.0 Hz, CH), 8.59 (s, 1H, CH_{pyr}); ^{13}C NMR ($CDCl_3$, 100 MHz) δ 14.28 (CH_3), 20.70 (CH_3), 22.84 (CH_2), 23.21 (CH_2), 23.25 (CH_2), 26.48 (CH_2), 29.51 (CH_2), 29.56 (CH_2), 29.58 (CH_2), 29.67 (CH_2), 29.77 (CH_2), 29.81 (CH_2), 29.86 (CH_2), 32.07 (CH_2), 49.73 (CH_3N^+), 49.94

(CH_3N^+), 51.42 (CH_3N^+), 51.46 (CH_3N^+), 62.44 (CH_2), 65.89 (CH_2), 66.34 (CH_2), 98.57 ($\underline{C}HCH_3$), 122.67 (C_{pyr}), 134.05 (C_{pyr}), 137.07 (C_{pyr}), 146.84 (C_{pyr}), 150.86 (C_{pyr}); HRMS-ESI $[M-2Cl]^{2+}$ 385.8795 (calculated for $C_{50}H_{97}N_3O_2$, 385.8785).

5,8-Bis((*N*,*N*-dimethyl-*N*-(octyl)ammonio)methyl)-2-ethyl-4*H*-[1,3]dioxino[4,5-*c*]pyridine dichloride (5c8)

Yield 30%; white solid; mp 170-171 °C (dec.); ^{1}H NMR ($CDCl_3$, 400 MHz) δ 0.88 (m, 6H, 2$\underline{CH_3}C_7H_{14}$), 1.05 (t, 3H, $^3J_{HH}$ = 7.1 Hz, $\underline{CH_3}CH_2$), 1.18-1.49 (m, 20H, 10CH_2), 1.68-1.96 (m, 6H, 3CH_2), 3.33 (s, 6H, 2CH_3N^+), 3.38 (s, 6H, 2CH_3N^+), 3.44-3.56 (m, 1H, CH_2N^+), 3.58-3.76 (m, 2H, CH_2N^+), 3.81-3.93 (m, 1H, CH_2N^+), 4.64, 4.72 (AB, 2H, $^2J_{HH}$ = 12.4 Hz, CH_2), 5.12, 5.22 (AB, 2H, $^2J_{HH}$ = 12.9 Hz, CH_2), 5.13, 5.57 (AB, 2H, $^2J_{HH}$ = 15.9 Hz, CH_2), 5.44 (br s, 1H, CH), 8.59 (s, 1H, CH_{pyr}); ^{13}C NMR ($CDCl_3$, 100 MHz) δ 8.04 (CH_3), 14.17 (CH_3), 22.68 (CH_2), 23.17 (CH_2), 26.44 (CH_2), 27.57 (CH_2), 29.17 (CH_2), 29.39 (CH_2), 29.45 (CH_2), 31.77 (CH_2), 49.66 (CH_3N^+), 49.82 (CH_3N^+), 51.18 (CH_3N^+), 51.41 (CH_3N^+), 61.86 (CH_2), 62.23 (CH_2), 65.57 (CH_2), 65.68 (CH_2), 66.36 (CH_2), 102.10 ($\underline{C}HC_2H_5$), 122.98 (C_{pyr}), 134.71 (C_{pyr}), 136.79 (C_{pyr}), 146.48 (C_{pyr}), 150.92 (C_{pyr}); HRMS-ESI $[M-2Cl]^{2+}$ 252.7303 (calculated for $C_{31}H_{59}N_3O_2$, 252.7298).

5,8-Bis((*N*,*N*-dimethyl-*N*-(decyl)ammonio)methyl)-2-ethyl-4*H*-[1,3]dioxino[4,5-*c*]pyridine dichloride (5c10)

Yield 20%; white solid; mp 170-172 °C (dec.); ^{1}H NMR ($CDCl_3$, 400 MHz) δ 0.88 (t, 6H, $^3J_{HH}$ = 6.4 Hz, 2$\underline{CH_3}C_9H_{18}$), 1.04 (t, 3H, $^3J_{HH}$ = 7.4 Hz, $\underline{CH_3}CH_2$), 1.18-1.48 (m, 28H, 14CH_2), 1.69-1.93 (m, 6H, 3CH_2), 3.33 (s, 6H, 2CH_3N^+), 3.37 (s, 6H, 2CH_3N^+), 3.43-3.55 (m, 1H, CH_2N^+), 3.57-3.77 (m, 2H, CH_2N^+), 3.81-3.93 (m, 1H, CH_2N^+), 4.64, 4.72 (AB, 2H, $^2J_{HH}$ = 12.5 Hz, CH_2), 5.11, 5.20 (AB, 2H, $^2J_{HH}$ = 13.3 Hz, CH_2), 5.13, 5.54 (AB, 2H, $^2J_{HH}$ = 16.1 Hz, CH_2), 5.43 (t, 1H, $^3J_{HH}$ = 4.8 Hz, CH), 8.55 (s, 1H, CH_{pyr}); ^{13}C NMR ($CDCl_3$, 100 MHz) δ 8.02 (CH_3), 14.21 (CH_3), 22.76 (CH_2), 23.19 (CH_2), 26.45 (CH_2), 27.57 (CH_2), 29.35 (CH_2), 29.45 (CH_2), 29.52 (CH_2), 29.56 (CH_2), 31.93 (CH_2), 49.56 (CH_3N^+), 49.76 (CH_3N^+), 51.19 (CH_3N^+), 51.31 (CH_3N^+), 61.73 (CH_2), 62.17 (CH_2), 65.60 (CH_2), 65.72 (CH_2), 66.36 (CH_2), 102.12 ($\underline{C}HC_2H_5$), 123.10 (C_{pyr}), 134.98 (C_{pyr}), 136.58 (C_{pyr}), 146.22 (C_{pyr}), 150.99 (C_{pyr}); HRMS-ESI $[M-2Cl]^{2+}$ 280.7616 (calculated for $C_{35}H_{67}N_3O_2$, 280.7611).

5,8-Bis((*N*,*N*-dimethyl-*N*-(dodecyl)ammonio)methyl)-2-ethyl-4*H*-[1,3]dioxino[4,5-c]pyridine dichloride (5c$_{12}$)

Yield 65%; white solid; mp 180-190 °C (dec.); ^{1}H NMR ($CDCl_3$, 400 MHz) δ 0.84 (t, 6H, $^3J_{HH}$ = 6.7 Hz, 2$\underline{CH_3}C_{11}H_{22}$), 1.00 (t, 3H, $^3J_{HH}$ = 7.5 Hz, $\underline{CH_3}CH_2$), 1.22-1.33 (m, 32H, 16CH_2), 1.70-1.84 (m, 6H, 3CH_2), 2.96 (m, 2H, CH_2), 3.29-3.32 (m, 12H, 4CH_3N^+), 3.50-3.83 (m, 4H, 2CH_2N^+), 4.69, 4.74 (AB, 2H, $^2J_{HH}$ = 13.6 Hz, CH_2), 5.10, 5.55 (AB, 2H, $^2J_{HH}$ = 16.7 Hz, CH_2), 5.11, 5.21 (AB, 2H, $^2J_{HH}$ = 13.6 Hz, CH_2), 5.41 (t, 1H, $^3J_{HH}$ = 4.4 Hz, CH), 8.60 (s, 1H, CH_{Pyr}); ^{13}C NMR ($CDCl_3$, 100 MHz) δ 8.01 (CH_3), 14.21 (CH_3), 22.77 (CH_3), 23.18 (CH_2), 26.46 (CH_2), 27.57 (CH_2), 29.43 (CH_3), 29.46 (CH_2), 29.53 (CH_2), 29.62 (CH_2), 29.70 (CH_2), 31.99 (CH_2), 49.60 (CH_3N^+), 49.76 (CH_3N^+), 51.11 (CH_3N^+), 51.34 (CH_3N^+), 61.94 (CH_2), 62.26 (CH_2), 65.60 (CH_2), 65.66 (CH_2N^+), 66.34 (CH_2N^+), 102.04 ($\underline{C}HC_2H_5$), 122.92 (C_{Pyr}), 134.60 (C_{Pyr}), 136.87 (C_{Pyr}), 146.54 (C_{Pyr}), 150.88 (C_{Pyr}); HRMS-ESI [M-2Cl]$^{2+}$ 308.7924 (calculated for $C_{39}H_{75}N_3O_2$, 308.7924).

5,8-Bis((*N*,*N*-dimethyl-*N*-(tetradecyl)ammonio)methyl)-2-ethyl-4*H*-[1,3]dioxino[4,5-*c*]pyridine dichloride (5c$_{14}$)

Yield 25%; white solid; mp 177-178 °C (dec.); ^{1}H NMR ($CDCl_3$, 400 MHz) δ 0.87 (t, 6H, $^3J_{HH}$ = 6.3 Hz, 2$\underline{CH_3}C_{13}H_{26}$), 1.04 (t, 3H, $^3J_{HH}$ = 7.3 Hz, $\underline{CH_3}CH_2$), 1.14-1.50 (m, 44H, 22CH_2), 1.71-1.91 (m, 6H, 3CH_2), 3.32 (s, 3H, CH_3N^+), 3.37 (s, 3H, CH_3N^+), 3.44-3.56 (m, 1H, CH_2N^+), 3.58-3.74 (m, 2H, CH_2N^+), 3.79-3.92 (m, 1H, CH_2N^+), 4.66, 4.72 (AB, 2H, $^2J_{HH}$ = 12.6 Hz, CH_2), 5.10, 5.19 (AB, 2H, $^2J_{HH}$ = 13.2 Hz, CH_2), 5.11, 5.54 (AB, 2H, $^2J_{HH}$ = 16.1 Hz, CH_2), 5.42 (t, 1H, $^3J_{HH}$ = 4.4 Hz, CH), 8.55 (s, 1H, CH_{pyr}); ^{13}C NMR ($CDCl_3$, 100 MHz) δ 8.04 (CH_3), 14.25 (CH_3), 22.81 (CH_2), 23.19 (CH_2), 26.47 (CH_2), 27.60 (CH_2), 29.48 (CH_2), 29.55 (CH_2), 29.65 (CH_2), 29.75 (CH_2), 29.77 (CH_2), 29.80 (CH_2), 32.03 (CH_2), 49.63 (CH_3N^+), 49.76 (CH_3N^+), 51.11 (CH_3N^+), 51.37 (CH_3N^+), 62.15 (CH_2), 62.33 (CH_2), 65.64 (CH_2), 66.29 (CH_2), 101.97 ($\underline{C}HC_2H_5$), 122.70 (C_{pyr}), 134.12 (C_{pyr}), 137.17 (C_{pyr}), 146.85 (C_{pyr}), 150.70 (C_{pyr}); HRMS-ESI [M-2Cl]$^{2+}$ 336.8243 (calculated for $C_{43}H_{83}N_3O_2$, 336.8237).

5,8-Bis((*N*,*N*-dimethyl-*N*-(hexadecyl)ammonio)methyl)-2-ethyl-4*H*-[1,3]dioxino[4,5-*c*]pyridine dichloride (5c$_{16}$)

Yield 38%; white solid; mp 170-172 °C (dec.); ^{1}H NMR ($CDCl_3$, 400 MHz) δ 0.87 (t, 6H, $^3J_{HH}$ = 6.6 Hz, 2$\underline{CH_3}C_{15}H_{30}$), 1.03 (t, 3H, $^3J_{HH}$ = 7.4 Hz, $\underline{CH_3}CH_2$), 1.15-1.49 (m, 52H,

$26CH_2$), 1.70-1.95 (m, 6H, $3CH_2$), 3.33 (s, 6H, $2CH_3N^+$), 3.37 (s, 6H, $2CH_3N^+$), 3.45-3.56 (m, 1H, CH_2N^+), 3.58-3.75 (m, 2H, CH_2N^+), 3.80-3.93 (m, 1H, CH_2N^+), 4.66, 4.72 (AB, 2H, $^2J_{HH}$ = 12.7 Hz, CH_2), 5.11, 5.20 (AB, 2H, $^2J_{HH}$ = 13.4 Hz, CH_2), 5.12, 5.54 (AB, 2H, $^2J_{HH}$ = 16.2 Hz, CH_2), 5.43 (t, 1H, $^3J_{HH}$ = 4.9 Hz, CH), 8.56 (s, 1H, CH_{pyr}); ^{13}C NMR ($CDCl_3$, 100 MHz) δ 8.01 (CH_3), 14.22 (CH_3), 22.78 (CH_2), 23.18 (CH_2), 26.46 (CH_2), 27.56 (CH_2), 29.46 (CH_2), 29.54 (CH_2), 29.63 (CH_2), 29.76 (CH_2), 29.80 (CH_2), 32.01 (CH_2), 49.55 (CH_3N^+), 49.74 (CH_3N^+), 51.15 (CH_3N^+), 51.30 (CH_3N^+), 61.84 (CH_2), 62.20 (CH_2), 65.70 (CH_2), 66.30 (CH_2), 102.06 ($\underline{C}HC_2H_5$), 122.98 (C_{pyr}), 134.74 (C_{pyr}), 136.76 (C_{pyr}), 146.45 (C_{pyr}), 150.89 (C_{pyr}); HRMS-ESI $[M-2Cl]^{2+}$ 364.8559 (calculated for $C_{47}H_{91}N_3O_2$, 364.8550).

5,8-Bis((*N*,*N*-dimethyl-*N*-(octadecyl)ammonio)methyl)-2-ethyl-4*H*-[1,3]dioxino[4,5-*c*]pyridine dichloride (5c$_{18}$)

Yield 41%; white solid; mp 180-181 °C (dec.); ^1H NMR ($CDCl_3$, 400 MHz) δ 0.86 (t, 6H, $^3J_{HH}$ = 6.7 Hz, $2\underline{CH_3}C_{17}H_{34}$), 1.02 (t, 3H, $^3J_{HH}$ = 7.5 Hz, $\underline{CH_3}CH_2$), 1.11-1.46 (m, 60H, $30CH_2$), 1.69-1.92 (m, 6H, $3CH_2$), 3.30 (s, 3H, CH_3N^+), 3.30-3.33 (m, 6H, $2CH_3N^+$), 3.47-3.59 (m, 1H, CH_2N^+), 3.60-3.74 (m, 2H, CH_2N^+), 3.76-3.90 (m, 1H, CH_2N^+), 4.72, 4.77 (AB, 2H, $^2J_{HH}$ = 13.6 Hz, CH_2), 5.11, 5.57 (AB, 2H, $^2J_{HH}$ = 16.2 Hz, CH_2), 5.13, 5.25 (AB, 2H, $^2J_{HH}$ = 13.3 Hz, CH_2), 5.42 (t, 1H, $^3J_{HH}$ = 5.0 Hz, CH), 8.61 (s, 1H, CH_{pyr}); ^{13}C NMR ($CDCl_3$, 100 MHz) δ 8.04 (CH_3), 14.25 (CH_3), 22.80 (CH_2), 23.20 (CH_2), 26.47 (CH_2), 27.58 (CH_2), 29.48 (CH_2), 29.56 (CH_2), 29.66 (CH_2), 29.76 (CH_2), 29.79 (CH_2), 29.83 (CH_2), 32.03 (CH_2), 49.61 (CH_3N^+), 49.79 (CH_3N^+), 51.16 (CH_3N^+), 51.31 (CH_3N^+), 61.84 (CH_2), 62.22 (CH_2), 65.70 (CH_2), 66.42 (CH_2), 101.12 ($\underline{C}HC_2H_5$), 123.05 (C_{pyr}), 134.97 (C_{pyr}), 136.62 (C_{pyr}), 146.30 (C_{pyr}), 151.04 (C_{pyr}); HRMS-ESI $[M-2Cl]^{2+}$ 392.8867 (calculated for $C_{51}H_{99}N_3O_2$, 392.8863).

5,8-Bis((*N*,*N*-dimethyl-*N*-(octyl)ammonio)methyl)-2-propyl-4*H*-[1,3]dioxino[4,5-*c*]pyridine dichloride (5d$_8$)

Yield 35%; white solid; mp 174-175 °C (dec.); ^1H NMR ($CDCl_3$, 400 MHz) δ 0.84-0.88 (m, 6H, $2\underline{CH_3}C_7H_{14}$), 0.97 (t, 3H, $^3J_{HH}$ = 7.3 Hz, $\underline{CH_3}CH_2CH_2$), 1.14-1.42 (m, 20H, $10CH_2$), 1.44-1.53 (m, 2H, CH_2), 1.67-1.91 (m, 6H, $3CH_2$), 3.28-3.32 (m, 12H, $4CH_3N^+$), 3.48-3.55 (m, 1H, CH_2N^+), 3.60-3.69 (m, 2H, CH_2N^+), 3.78-3.85 (m, 1H, CH_2N^+), 4.65, 4.71 (AB, 2H, $^2J_{HH}$ = 12.8 Hz, CH_2), 5.07, 5.16 (AB, 2H, $^2J_{HH}$ = 13.3 Hz, CH_2), 5.09, 5.52 (AB, 2H, $^2J_{HH}$ = 16.2 Hz, CH_2), 5.47 (t, 1H, $^3J_{HH}$ = 5.2 Hz, CH), 8.56 (s, 1H, CH_{pyr}); ^{13}C NMR ($CDCl_3$,

100 MHz) δ 14.11 (CH_3), 14.19 (CH_3), 17.11 (CH_2), 22.70 (CH_2), 23.19 (CH_2), 26.46 (CH_2), 29.18 (CH_2), 29.42 (CH_2), 29.46 (CH_2), 31.79 (CH_2), 36.44 (CH_2), 49.61 (CH_3N^+), 49.78 (CH_3N^+), 51.10 (CH_3N^+), 51.41 (CH_3N^+), 62.11 (CH_2), 62.35 (CH_2), 65.63 (CH_2), 66.30 (CH_2), 101.15 ($\underline{C}HC_3H_7$), 122.70 (C_{pyr}), 134.10 (C_{pyr}), 137.12 (C_{pyr}), 146.83 (C_{pyr}), 150.71 (C_{pyr}); HRMS-ESI $[M-2Cl]^{2+}$ 259.7381 (calculated for $C_{32}H_{61}N_3O_2$, 259.7376).

5,8-Bis((*N*,*N*-dimethyl-*N*-(decyl)ammonio)methyl)-2-propyl-4*H*-[1,3]dioxino[4,5-*c*]pyridine dichloride (5d$_{10}$)

Yield 31%; white solid; mp 179-182 °C (dec.); ^1H NMR ($CDCl_3$, 400 MHz) δ 0.87 (t, 6H, $^3J_{HH}$ = 6.4 Hz, $2\underline{CH_3}C_9H_{18}$), 0.98 (t, 3H, $^3J_{HH}$ = 7.3 Hz, $\underline{CH_3}CH_2CH_2$), 1.16-1.42 (m, 28H, $14CH_2$), 1.44-1.54 (m, 2H, CH_2), 1.68-1.92 (m, 6H, $3CH_2$), 3.30-3.35 (m, 12H, $4CH_3N^+$), 3.47-3.55 (m, 1H, CH_2N^+), 3.60-3.70 (m, 2H, CH_2N^+), 3.80-3.88 (m, 1H, CH_2N^+), 4.68, 4.71 (AB, 2H, $^2J_{HH}$ = 13.6 Hz, CH_2), 5.10, 5.53 (AB, 2H, $^2J_{HH}$ = 16.2 Hz, CH_2), 5.10, 5.20 (AB, 2H, $^2J_{HH}$ = 13.3 Hz, CH_2), 5.47 (t, 1H, $^3J_{HH}$ = 5.0 Hz, CH), 8.57 (s, 1H, CH_{pyr}); ^{13}C NMR ($CDCl_3$, 100 MHz) δ 14.13 (CH_3), 14.24 (CH_3), 17.11 (CH_2), 22.78 (CH_2), 23.21 (CH_2), 26.48 (CH_2), 29.38 (CH_2), 29.49 (CH_2), 29.53 (CH_2), 29.58 (CH_2), 31.96 (CH_2), 36.46 (CH_2), 49.65 (CH_3N^+), 49.85 (CH_3N^+), 51.21 (CH_3N^+), 51.43 (CH_3N^+), 62.23 (CH_2), 62.38 (CH_2), 65.67 (CH_2), 65.78 (CH_2), 66.34 (CH_2), 101.18 ($\underline{C}HC_3H_7$), 122.68 (C_{pyr}), 134.12 (C_{pyr}), 137.14 (C_{pyr}), 146.85 (C_{pyr}), 150.76 (C_{pyr}); HRMS-ESI $[M-2Cl]^{2+}$ 287.7694 (calculated for $C_{36}H_{69}N_3O_2$, 287.7689).

5,8-Bis((*N*,*N*-dimethyl-*N*-(dodecyl)ammonio)methyl)-2-propyl-4*H*-[1,3]dioxino[4,5-*c*]pyridine dichloride (5d$_{12}$)

Yield 28%; white solid; mp 183-184 °C (dec.); ^1H NMR ($CDCl_3$, 400 MHz) δ 0.87 (t, 6H, $^3J_{HH}$ = 6.6 Hz, $2\underline{CH_3}C_{11}H_{22}$), 0.98 (t, 3H, $^3J_{HH}$ = 7.3 Hz, $\underline{CH_3}CH_2CH_2$), 1.16-1.45 (m, 36H, $18CH_2$), 1.45-1.56 (m, 2H, CH_2), 1.68-2.06 (m, 6H, $3CH_2$), 3.31-3.36 (m, 12H, $4CH_3N^+$), 3.44-3.56 (m, 1H, CH_2N^+), 3.57-3.74 (m, 2H, CH_2N^+), 3.78-3.93 (m, 1H, CH_2N^+), 4.67, 4.70 (AB, 2H, $^2J_{HH}$ = 12.5 Hz, CH_2), 5.10, 5.53 (AB, 2H, $^2J_{HH}$ = 16.1 Hz, CH_2), 5.10, 5.19 (AB, 2H, $^2J_{HH}$ = 13.4 Hz, CH_2), 5.48 (t, 1H, $^3J_{HH}$ = 4.3 Hz, CH), 8.56 (s, 1H, CH_{pyr}); ^{13}C NMR ($CDCl_3$, 100 MHz) δ 14.12 (CH_3), 14.25 (CH_3), 17.11 (CH_2), 22.80 (CH_2), 23.21 (CH_2), 26.48 (CH_2), 29.45 (CH_2), 29.51 (CH_2), 29.54 (CH_2), 29.64 (CH_2), 29.72 (CH_2), 32.02 (CH_2), 36.45 (CH_2), 49.62 (CH_3N^+), 49.81 (CH_3N^+), 51.14 (CH_3N^+), 51.41 (CH_3N^+), 62.21 (CH_2), 62.37 (CH_2),

65.65 (CH_2), 65.76 (CH_2), 66.33 (CH_2), 101.16 ($\underline{C}HC_3H_7$), 122.67 (C_{pyr}), 134.10 (C_{pyr}), 137.14 (C_{pyr}), 146.86 (C_{pyr}), 150.72 (C_{pyr}); HRMS-ESI $[M-2Cl]^{2+}$ 315.8002 (calculated for $C_{40}H_{77}N_3O_2$, 315.8008).

5,8-Bis((*N*,*N*-dimethyl-*N*-(tetradecyl)ammonio)methyl)-2-propyl-4*H*-[1,3]dioxino[4,5-*c*]pyridine dichloride (5d$_{14}$)

Yield 26%; white solid; mp 180-182 °C (dec.); ^{1}H NMR ($CDCl_3$, 400 MHz) δ 0.87 (t, 6H, $^3J_{HH}$ = 6.7 Hz, 2$\underline{CH_3}C_{13}H_{26}$), 0.98 (t, 3H, $^3J_{HH}$ = 7.4 Hz, $\underline{CH_3}CH_2CH_2$), 1.16-1.44 (m, 44H, 22CH_2), 1.45-1.54 (m, 2H, CH_2), 1.70-1.90 (m, 6H, 3CH_2), 3.30-3.35 (m, 12H, 4CH_3N^+), 3.47-3.54 (m, 1H, CH_2N^+), 3.59-3.70 (m, 2H, CH_2N^+), 3.80-3.87 (m, 1H, CH_2N^+), 4.68, 4.71 (AB, 2H, $^2J_{HH}$ = 13.2 Hz, CH_2), 5.10, 5.19 (AB, 2H, $^2J_{HH}$ = 13.3 Hz, CH_2), 5.10, 5.53 (AB, 2H, $^2J_{HH}$ = 16.2 Hz, CH_2), 5.47 (t, 1H, $^3J_{HH}$ = 5.1 Hz, CH), 8.56 (s, 1H, CH_{pyr}); ^{13}C NMR ($CDCl_3$, 100 MHz) δ 14.14 (CH_3), 14.26 (CH_3), 17.12 (CH_2), 22.82 (CH_2), 23.23 (CH_2), 26.49 (CH_2), 29.50 (CH_2), 29.55 (CH_2), 29.66 (CH_2), 29.75 (CH_2), 29.78 (CH_2), 29.81 (CH_2), 32.05 (CH_2), 36.47 (CH_2), 49.67 (CH_3N^+), 49.86 (CH_3N^+), 51.21 (CH_3N^+), 51.43 (CH_3N^+), 62.27 (CH_2), 62.41 (CH_2), 65.67 (CH_2), 65.83 (CH_2), 66.38 (CH_2), 101.19 ($\underline{C}HC_3H_7$), 122.68 (C_{pyr}), 134.13 (C_{pyr}), 137.13 (C_{pyr}), 146.84 (C_{pyr}), 150.78 (C_{pyr}); HRMS-ESI $[M-2Cl]^{2+}$ 343.8321 (calculated for $C_{44}H_{85}N_3O_2$, 343.8315).

5,8-Bis((*N*,*N*-dimethyl-*N*-(hexadecyl)ammonio)methyl)-2-propyl-4*H*-[1,3]dioxino[4,5-*c*]pyridine dichloride (5d$_{16}$)

Yield 49%; white solid; mp 182-183 °C (dec.); ^{1}H NMR ($CDCl_3$, 400 MHz) δ 0.87 (t, 6H, $^3J_{HH}$ = 6.7 Hz, 2$\underline{CH_3}C_{15}H_{30}$), 0.99 (t, 3H, $^3J_{HH}$ = 7.4 Hz, $\underline{CH_3}CH_2CH_2$), 1.16-1.44 (m, 52H, 26CH_2), 1.45-1.55 (m, 2H, CH_2), 1.70-1.87 (m, 6H, 3CH_2), 3.31-3.36 (m, 12H, 4CH_3N^+), 3.47-3.54 (m, 1H, CH_2N^+), 3.59-3.73 (m, 2H, CH_2N^+), 3.81-3.88 (m, 1H, CH_2N^+), 4.67, 4.70 (AB, 2H, $^2J_{HH}$ = 13.2 Hz, CH_2), 5.10, 5.19 (AB, 2H, $^2J_{HH}$ = 13.4 Hz, CH_2), 5.10, 5.53 (AB, 2H, $^2J_{HH}$ = 16.2 Hz, CH_2), 5.48 (t, 1H, $^3J_{HH}$ = 5.2 Hz, CH), 8.56 (s, 1H, CH_{pyr}); ^{13}C NMR ($CDCl_3$, 100 MHz) δ 14.14 (CH_3), 14.27 (CH_3), 17.12 (CH_2), 22.83 (CH_2), 23.22 (CH_2), 26.49 (CH_2), 29.51 (CH_2), 29.56 (CH_2), 29.67 (CH_2), 29.76 (CH_2), 29.80 (CH_2), 29.84 (CH_2), 32.06 (CH_2), 36.48 (CH_2), 49.71 (CH_3N^+), 49.91 (CH_3N^+), 51.27 (CH_3N^+), 51.45 (CH_3N^+), 62.34 (CH_2), 62.42 (CH_2), 65.67 (CH_2), 65.90 (CH_2), 66.43 (CH_2), 101.21 ($\underline{C}HC_3H_7$), 122.68 (C_{pyr}), 134.17

(C_{pyr}), 137.10 (C_{pyr}), 146.82 (C_{pyr}), 150.82 (C_{pyr}); HRMS-ESI $[M-2Cl]^{2+}$ 371.8632 (calculated for $C_{48}H_{93}N_3O_2$, 371.8628).

5,8-Bis((*N*,*N*-dimethyl-*N*-(octadecyl)ammonio)methyl)-2-propyl-4*H*-[1,3]dioxino[4,5-*c*]pyridine dichloride (5d18)

Yield 28%; white solid; mp 166-165 °C (dec.); ^1H NMR ($CDCl_3$, 400 MHz) δ 0.87 (t, 6H, $^3J_{HH}$ = 6.7 Hz, 2$\underline{CH_3}C_{17}H_{34}$), 0.99 (t, 3H, $^3J_{HH}$ = 7.3 Hz, $\underline{CH_3}CH_2CH_2$), 1.14-1.44 (m, 60H, 30CH_2), 1.45-1.55 (m, 2H, CH_2), 1.67-1.87 (m, 6H, 3CH_2), 3.31-3.36 (m, 12H, 4CH_3N^+), 3.44-3.54 (m, 1H, CH_2N^+), 3.58-3.73 (m, 2H, CH_2N^+), 3.79-3.89 (m, 1H, CH_2N^+), 4.65, 4.73 (AB, 2H, $^2J_{HH}$ = 12.5 Hz, CH_2), 5.09, 5.17 (AB, 2H, $^2J_{HH}$ = 13.6 Hz, CH_2), 5.11, 5.53 (AB, 2H, $^2J_{HH}$ = 16.4 Hz, CH_2), 5.48 (t, 1H, $^3J_{HH}$ = 5.2 Hz, CH), 8.54 (s, 1H, CH_{pyr}); ^{13}C NMR ($CDCl_3$, 100 MHz) δ 14.08 (CH_3), 14.22 (CH_3), 17.08 (CH_2), 22.77 (CH_2), 23.17 (CH_2), 26.45 (CH_2), 29.45 (CH_2), 29.51 (CH_2), 29.56 (CH_2), 29.66 (CH_2), 29.75 (CH_2), 29.80 (CH_2), 32.00 (CH_2), 36.40 (CH_2), 49.56 (CH_3N^+), 49.67 (CH_3N^+), 50.93 (CH_3N^+), 51.33 (CH_3N^+), 61.97 (CH_2), 62.27 (CH_2), 65.52 (CH_2), 65.56 (CH_2), 66.26 (CH_2), 101.07 ($\underline{C}HC_3H_7$), 122.70 (C_{pyr}), 134.04 (C_{pyr}), 137.07 (C_{pyr}), 146.81 (C_{pyr}), 150.65 (C_{pyr}); HRMS-ESI $[M-2Cl]^{2+}$ 399.8948 (calculated for $C_{52}H_{101}N_3O_2$, 399.8941).

5,8-Bis((*N*,*N*-dimethyl-*N*-(octyl)ammonio)methyl)-2-isopropyl-4*H*-[1,3]dioxino[4,5-*c*]pyridine dichloride (5e8)

Yield 49%; white solid; mp 162-164 °C (dec.); ^1H NMR ($CDCl_3$, 400 MHz) δ 0.84-0.89 (m, 6H, 2$\underline{CH_3}C_7H_{14}$), 1.03 (d, 3H, $^3J_{HH}$ = 7.7 Hz, CH_3), 1.05 (d, 3H, $^3J_{HH}$ = 7.6 Hz, CH_3),1.20-1.43 (m, 18H, 9CH_2), 1.74-1.88 (m, 4H, 2CH_2), 1.99-2.09 (m, 3H, CH_2 + CH), 3.32-3.87 (m, 16H, 4CH_3N^+ + 2CH_2N^+), 4.66, 4.71 (AB, 2H, $^2J_{HH}$ = 13.2 Hz, CH_2), 5.20 (d, 1H, $^3J_{HH}$ = 5.4 Hz, CH), 5.10, 5.20 (AB, 2H, $^2J_{HH}$ = 13.4 Hz, CH_2), 5.10, 5.61 (AB, 2H, $^2J_{HH}$ = 15.8 Hz, CH_2), 8.54 (s, 1H, CH_{pyr}); ^{13}C NMR ($CDCl_3$, 100 MHz) δ 14.20 (CH_3), 16.79 (CH_3), 16.89 (CH_3), 22.72 (CH_2), 23.19 (CH_2), 23.22 (CH_2), 26.45 (CH_2), 29.17 (CH_2), 29.40 (CH_2), 29.47 (CH_2), 31.79 (CH_2), 32.51 ($\underline{C}H(CH_3)_2$), 49.71 (CH_3N^+), 49.90 (CH_3N^+), 51.07 (CH_3N^+), 51.43 (CH_3N^+), 62.25 (CH_2), 62.39 (CH_2), 65.84 (CH_2), 66.56 (CH_2), 104.48 ($\underline{C}HCH(CH_3)_2$), 122.71 (C_{pyr}), 134.26 (C_{pyr}), 137.15 (C_{pyr}), 146.77 (C_{pyr}), 150.92 (C_{pyr}); HRMS-ESI $[M-2Cl]^{2+}$ 259.7381 (calculated for $C_{32}H_{61}N_3O_2$, 259.7376).

5,8-Bis((*N,N*-dimethyl-*N*-(decyl)ammonio)methyl)-2-isopropyl-4*H*-[1,3]dioxino[4,5-*c*]pyridine dichloride (5e$_{10}$)

Yield 55%; white solid; mp 167-170 °C (dec.); ^{1}H NMR (CDCl$_3$, 400 MHz) δ 0.86 (t, 6H, $^3J_{HH}$ = 6.7 Hz, 2$\underline{CH_3}$C$_9$H$_{18}$), 1.01 (d, 3H, $^3J_{HH}$ = 7.3 Hz, CH$_3$), 1.03 (d, 3H, $^3J_{HH}$ = 7.6 Hz, CH$_3$),1.23-1.39 (m, 26H, 13CH$_2$), 1.72-2.05 (m, 5H, 2CH$_2$ + CH), 2.25-2.51 (m, 2H, CH$_2$), 3.23-3.89 (m, 16H, 4CH$_3$N$^+$ + 2CH$_2$N$^+$), 4.64, 4.72 (AB, 2H, $^2J_{HH}$ = 12.6 Hz, CH$_2$), 5.15 (s, 1H, CH), 5.09, 5.18 (AB, 2H, $^2J_{HH}$ = 13.4 Hz, CH$_2$), 5.11, 5.59 (AB, 2H, $^2J_{HH}$ = 16.2 Hz, CH$_2$), 8.56 (s, 1H, CH$_{pyr}$); ^{13}C NMR (CDCl$_3$, 100 MHz) δ 14.23 (CH$_3$), 16.79 (CH$_3$), 16.86 (CH$_3$), 22.77 (CH$_2$), 23.18 (CH$_2$), 23.20 (CH$_2$), 26.44 (CH$_2$), 26.46 (CH$_2$), 29.37 (CH$_2$), 29.45 (CH$_2$), 29.52 (CH$_2$), 29.57 (CH$_2$), 31.94 (CH$_2$), 32.47 ($\underline{C}$H(CH$_3$)$_2$), 49.64 (CH$_3$N$^+$), 49.79 (CH$_3$N$^+$), 50.92 (CH$_3$N$^+$), 51.37 (CH$_3$N$^+$), 62.12 (CH$_2$), 62.35 (CH$_2$), 65.69 (CH$_2$), 65.79 (CH$_2$), 66.49 (CH$_2$), 104.43 ($\underline{C}$HCH(CH$_3$)$_2$), 122.70 (C$_{pyr}$), 134.19 (C$_{pyr}$), 137.17 (C$_{pyr}$), 146.80 (C$_{pyr}$), 150.84 (C$_{pyr}$); HRMS-ESI [M-2Cl]$^{2+}$ 287.7694 (calculated for C$_{36}$H$_{69}$N$_3$O$_2$, 287.7689).

5,8-Bis((*N,N*-dimethyl-*N*-(dodecyl)ammonio)methyl)-2-isopropyl-4*H*-[1,3]dioxino[4,5-*c*]pyridine dichloride (5e$_{12}$)

Yield 45%; white solid; mp 170-171 °C (dec.); ^{1}H NMR (CDCl$_3$, 400 MHz) δ 0.87 (t, 6H, $^3J_{HH}$ = 6.7 Hz, 2$\underline{CH_3}$C$_{11}$H$_{22}$), 1.03 (d, 3H, $^3J_{HH}$ = 7.7 Hz, CH$_3$), 1.05 (d, 3H, $^3J_{HH}$ = 7.6 Hz, CH$_3$), 1.24-1.41 (m, 34H, 17CH$_2$), 1.74-2.06 (m, 7H, 3CH$_2$ + CH), 3.24-3.90 (m, 16H, 4CH$_3$N$^+$ + 2CH$_2$N$^+$), 4.66, 4.71 (AB, 2H, $^2J_{HH}$ = 12.8 Hz, CH$_2$), 5.20 (d, 1H, $^3J_{HH}$ = 4.9 Hz, CH), 5.11, 5.19 (AB, 2H, $^2J_{HH}$ = 14.0 Hz, CH$_2$), 5.09, 5.60 (AB, 2H, $^2J_{HH}$ = 16.4 Hz, CH$_2$), 8.53 (s, 1H, CH$_{pyr}$); ^{13}C NMR (CDCl$_3$, 100 MHz) δ 14.28 (CH$_3$), 16.80 (CH$_3$), 16.90 (CH$_3$), 22.83 (CH$_2$), 23.21 (CH$_2$), 23.25 (CH$_2$), 26.47 (CH$_2$), 29.48 (CH$_2$), 29.54 (CH$_2$), 29.65 (CH$_2$), 29.74 (CH$_2$), 32.04 (CH$_2$), 32.51 ($\underline{C}$H(CH$_3$)$_2$), 49.72 (CH$_3$N$^+$), 49.91 (CH$_3$N$^+$), 51.04 (CH$_3$N$^+$), 51.41 (CH$_3$N$^+$), 62.28 (CH$_2$), 62.41 (CH$_2$), 65.84 (CH$_2$), 66.59 (CH$_2$), 104.47 ($\underline{C}$HCH(CH$_3$)$_2$), 122.68 (C$_{pyr}$), 134.25 (C$_{pyr}$), 137.14 (C$_{pyr}$), 146.76 (C$_{pyr}$), 150.91 (C$_{pyr}$); HRMS-ESI [M-2Cl]$^{2+}$ 315.8008 (calculated for C$_{40}$H$_{77}$N$_3$O$_2$, 315.8002).

5,8-Bis((*N,N*-dimethyl-*N*-(tetradecyl)ammonio)methyl)-2-isopropyl-4*H*-[1,3]dioxino[4,5-*c*]pyridine dichloride (5e$_{14}$)

Yield 59%; white solid; mp 169-172 °C (dec.); ^{1}H NMR (CDCl$_3$, 400 MHz) δ 0.87 (t, 6H, $^3J_{HH}$ = 6.6 Hz, 2$\underline{CH_3}$C$_{13}$H$_{26}$), 1.02 (d, 3H, $^3J_{HH}$ = 7.6 Hz, CH$_3$), 1.04 (d, 3H, $^3J_{HH}$ = 7.6 Hz,

CH_3), 1.18-1.42 (m, 42H, 21CH_2), 1.73-2.20 (m, 7H, 3CH_2 + CH), 3.30-3.85 (m, 16H, 4CH_3N^+ + 2CH_2N^+), 4.66, 4.71 (AB, 2H, $^2J_{HH}$ = 12.8 Hz, CH_2), 5.20 (d, 1H, $^3J_{HH}$ = 5.2 Hz, CH), 5.11, 5.19 (AB, 2H, $^2J_{HH}$ = 14.0 Hz, CH_2), 5.09, 5.60 (AB, 2H, $^2J_{HH}$ = 16.2 Hz, CH_2), 8.54 (s, 1H, CH_{pyr}); ^{13}C NMR ($CDCl_3$, 100 MHz) δ 14.26 (CH_3), 16.79 (CH_3), 16.88 (CH_3), 22.82 (CH_2), 23.21 (CH_2), 23.22 (CH_2), 26.47 (CH_2), 26.48 (CH_2), 29.49 (CH_2), 29.54 (CH_2), 29.65 (CH_2), 29.74 (CH_2), 29.78 (CH_2), 29.81 (CH_2), 32.04 (CH_2), 32.49 ($\underline{C}H(CH_3)_3$), 49.67 (CH_3N^+), 49.86 (CH_3N^+), 50.99 (CH_3N^+), 51.39 (CH_3N^+), 62.22 (CH_2), 62.39 (CH_2), 65.81 (CH_2), 66.54 (CH_2), 104.46 ($\underline{C}HCH(CH_3)_2$), 122.69 (C_{pyr}), 134.21 (C_{pyr}), 137.16 (C_{pyr}), 146.80 (C_{pyr}), 150.88 (C_{pyr}); HRMS-ESI [M-2Cl]$^{2+}$ 343.8322 (calculated for $C_{44}H_{85}N_3O_2$, 343.8315).

5,8-Bis((*N,N*-dimethyl-*N*-(hexadecyl)ammonio)methyl)-2-isopropyl-4*H*-[1,3]dioxino[4,5-*c*]pyridine dichloride (5e$_{16}$)

Yield 68%; white solid; mp 170-172 °C (dec.); ^1H NMR ($CDCl_3$, 400 MHz) δ 0.87 (t, 6H, $^3J_{HH}$ = 6.7 Hz, 2$\underline{CH_3}C_{15}H_{30}$), 1.03 (d, 3H, $^3J_{HH}$ = 7.6 Hz, CH_3), 1.04 (d, 3H, $^3J_{HH}$ = 7.7 Hz, CH_3), 1.17-1.45 (m, 50H, 25CH_2), 1.71-2.20 (m, 7H, 3CH_2 + CH), 3.30-3.85 (m, 16H, 4CH_3N^+ + 2CH_2N^+), 4.66, 4.71 (AB, 2H, $^2J_{HH}$ = 12.8 Hz, CH_2), 5.20 (d, 1H, $^3J_{HH}$ = 4.8 Hz, CH), 5.10, 5.18 (AB, 2H, $^2J_{HH}$ = 14.0 Hz, CH_2), 5.09, 5.60 (AB, 2H, $^2J_{HH}$ = 16.2 Hz, CH_2), 8.54 (s, 1H, CH_{pyr}); ^{13}C NMR ($CDCl_3$, 100 MHz) δ 14.27 (CH_3), 16.80 (CH_3), 16.89 (CH_3), 22.83 (CH_2), 23.19 (CH_2), 23.22 (CH_2), 26.47 (CH_2), 29.49 (CH_2), 29.55 (CH_2), 29.66 (CH_2), 29.75 (CH_2), 29.79 (CH_2), 29.84 (CH_2), 32.05 (CH_2), 32.49 ($\underline{C}H(CH_3)_2$), 49.68 (CH_3N^+), 49.85 (CH_3N^+), 50.97 (CH_3N^+), 51.38 (CH_3N^+), 62.25 (CH_2), 62.38 (CH_2), 65.82 (CH_2), 66.55 (CH_2), 104.46 ($\underline{C}HCH(CH_3)_2$), 122.68 (C_{pyr}), 134.22 (C_{pyr}), 137.14 (C_{pyr}), 146.77 (C_{pyr}), 150.89 (C_{pyr}); HRMS-ESI [M-2Cl]$^{2+}$ 371.8637 (calculated for $C_{48}H_{93}N_3O_2$, 371.8628).

5,8-Bis((*N,N*-dimethyl-*N*-(octadecyl)ammonio)methyl)-2-isopropyl-4*H*-[1,3]dioxino[4,5-*c*]pyridine dichloride (5e$_{18}$)

Yield 56%; white solid; mp 169-170 °C (dec.); ^1H NMR ($CDCl_3$, 400 MHz) δ 0.87 (t, 6H, $^3J_{HH}$ = 6.7 Hz, 2$\underline{CH_3}C_{15}H_{30}$), 1.04 (d, 3H, $^3J_{HH}$ = 6.7 Hz, CH_3), 1.06 (d, 3H, $^3J_{HH}$ = 6.7 Hz, CH_3), 1.19-1.47 (m, 58H, 29CH_2), 1.72-2.08 (m, 7H, 3CH_2 + CH), 3.24-3.92 (m, 16H, 4CH_3N^+ + 2CH_2N^+), 4.67 (s, 2H, CH_2), 5.09, 5.19 (AB, 2H, $^2J_{HH}$ = 14.0 Hz, CH_2), 5.21 (d, 1H, $^3J_{HH}$ = 4.7 Hz, CH), 5.09, 5.60 (AB, 2H, $^2J_{HH}$ = 16.2 Hz, CH_2), 8.51 (s, 1H, CH_{pyr}); ^{13}C NMR ($CDCl_3$, 100 MHz) δ 14.28 (CH_3), 16.79 (CH_3), 16.90 (CH_3), 22.84 (CH_2), 23.26 (CH_2), 26.48

(CH_2), 29.47 (CH_2), 29.51 (CH_2), 29.55 (CH_2), 29.67 (CH_2), 29.77 (CH_2), 29.81 (CH_2), 29.86 (CH_2), 32.07 (CH_2), 32.52 ($\underline{C}H(CH_3)_2$), 49.77 (CH_3N^+), 50.00 (CH_3N^+), 51.11 (CH_3N^+), 51.44 (CH_3N^+), 62.37 (CH_2), 62.45 (CH_2), 65.84 (CH_2), 65.96 (CH_2), 66.69 (CH_2), 104.51 ($\underline{C}HCH(CH_3)_2$), 122.71 (C_{pyr}), 134.31 (C_{pyr}), 137.12 (C_{pyr}), 146.73 (C_{pyr}), 150.98 (C_{pyr}); HRMS-ESI $[M-2Cl]^{2+}$ 399.8946 (calculated for $C_{52}H_{101}N_3O_2$, 399.8941).

5,8-Bis((*N*,*N*-dimethyl-*N*-(octyl)ammonio)methyl)-2-butyl-4*H*-[1,3]dioxino[4,5-*c*]pyridine dichloride (5f$_8$)

Yield 26%; white solid; mp 175-177 °C (dec.); ^{1}H NMR ($CDCl_3$, 400 MHz) δ 0.84 (t, 3H, $^3J_{HH}$ = 6.9 Hz, $\underline{CH_3}C_7H_{14}$), 0.85 (t, 3H, $^2J_{HH}$ = 6.0 Hz, $\underline{CH_3}C_7H_{14}$), 0.91 (t, 3H, $^3J_{HH}$ = 7.0 Hz, $\underline{CH_3}C_3H_6$), 1.12-1.49 (m, 24H, 12CH_2), 1.65-1.90 (m, 6H, 3CH_2), 3.03-3.11 (m, 13H, 4CH_3N^+ + CH_2N^+), 3.15-3.88 (m, 3H, 2CH_2N^+), 4.68, 4.76 (AB, 2H, $^2J_{HH}$ = 11.9 Hz, CH_2), 5.08, 5.56 (AB, 2H, $^2J_{HH}$ = 15.9 Hz, CH_2), 5.12, 5.23 (AB, 2H, $^2J_{HH}$ = 12.3 Hz, CH_2), 5.45 (br s, 1H, CH), 8.62 (s, 1H, CH_{pyr}); ^{13}C NMR ($CDCl_3$, 100 MHz) δ 14.09 (CH_3), 14.16 (CH_3), 22.56 (CH_2), 22.68 (CH_2), 23.18 (CH_2), 25.79 (CH_2), 26.45 (CH_2), 29.17 (CH_2), 29.42 (CH_2), 29.43 (CH_2), 31.76 (CH_2), 34.09 (CH_2), 49.59 (CH_3N^+), 49.76 (CH_3N^+), 51.13 (CH_3N^+), 51.43 (CH_3N^+), 61.72 (CH_2), 62.22 (CH_2), 65.62 (CH_2), 65.69 (CH_2), 66.35 (CH_2), 101.45 ($\underline{C}HC_4H_9$), 122.97 (C_{pyr}), 134.71 (C_{pyr}), 136.70 (C_{pyr}), 146.47 (C_{pyr}), 150.90 (C_{pyr}); HRMS-ESI $[M-2Cl]^{2+}$ 266.7469 (calculated for $C_{33}H_{63}N_3O_2$, 266.7465).

5,8-Bis((*N*,*N*-dimethyl-*N*-(decyl)ammonio)methyl)-2-butyl-4*H*-[1,3]dioxino[4,5-*c*]pyridine dichloride (5f$_{10}$)

Yield 77%; white solid; mp 183-184 °C (dec.); ^{1}H NMR ($CDCl_3$, 400 MHz) δ 0.85 (t, 3H, $^3J_{HH}$ = 7.1 Hz, $\underline{CH_3}C_9H_{18}$), 0.86 (t, 3H, $^2J_{HH}$ = 7.1 Hz, $\underline{CH_3}C_9H_{18}$), 0.92 (t, 3H, $^3J_{HH}$ = 7.0 Hz, $\underline{CH_3}C_3H_6$), 1.16-1.49 (m, 32H, 16CH_2), 1.66-1.92 (m, 6H, 3CH_2), 3.20-3.43 (m, 13H, 4CH_3N^+ + CH_2N^+), 3.47-3.89 (m, 3H, 2CH_2N^+), 4.72, 4.81 (AB, 2H, $^2J_{HH}$ = 12.2 Hz, CH_2), 5.10, 5.29 (AB, 2H, $^2J_{HH}$ = 16.1 Hz, CH_2), 5.15, 5.59 (AB, 2H, $^2J_{HH}$ = 13.0 Hz, CH_2), 5.48 (br s, 1H, CH), 8.64 (s, 1H, CH_{pyr}); ^{13}C NMR ($CDCl_3$, 100 MHz) δ 14.11 (CH_3), 14.22 (CH_3), 22.58 (CH_2), 22.77 (CH_2), 23.22 (CH_2), 25.82 (CH_2), 26.49 (CH_2), 29.37 (CH_2), 29.50 (CH_2), 29.53 (CH_2), 29.57 (CH_2), 31.95 (CH_2), 34.10 (CH_2), 49.65 (CH_3N^+), 49.83 (CH_3N^+), 51.20 (CH_3N^+), 51.41 (CH_3N^+), 61.60 (CH_2), 62.19 (CH_2), 65.74 (CH_2), 65.77 (CH_2), 66.50 (CH_2),

101.57 ($\underline{C}HC_4H_9$), 123.19 (C_{pyr}), 135.26 (C_{pyr}), 136.36 (C_{pyr}), 146.12 (C_{pyr}), 151.14 (C_{pyr}); HRMS-ESI $[M-2Cl]^{2+}$ 294.7774 (calculated for $C_{37}H_{71}N_3O_2$, 294.7768).

5,8-Bis((*N*,*N*-dimethyl-*N*-(dodecyl)ammonio)methyl)-2-butyl-4*H*-[1,3]dioxino[4,5-*c*]pyridine dichloride (5f$_{12}$)

Yield 27%; white solid; mp 178-180 °C (dec.); ^1H NMR ($CDCl_3$, 400 MHz) δ 0.88 (t, 6H, $^3J_{HH}$ = 6.6 Hz, $2\underline{CH_3}C_{11}H_{22}$), 0.94 (t, 3H, $^3J_{HH}$ = 7.0 Hz, $\underline{CH_3}C_3H_6$), 1.16-1.51 (m, 40H, $20CH_2$), 1.67-1.94 (m, 6H, $3CH_2$), 3.22-3.94 (m, 16H, $4CH_3N^+$ + $2CH_2N^+$), 4.62, 4.70 (AB, 2H, $^2J_{HH}$ = 12.5 Hz, CH_2), 5.06-5.22 (m, 3H, $2CH_2$), 5.46-5.58 (m, 2H, CH+CH_2), 8.54 (s, 1H, CH_{pyr}); ^{13}C NMR ($CDCl_3$, 100 MHz) δ 14.11 (CH_3), 14.23 (CH_3), 22.58 (CH_2), 22.78 (CH_2), 23.22 (CH_2), 25.82 (CH_2), 26.49 (CH_2), 29.44 (CH_2), 29.54 (CH_2), 29.63 (CH_2), 29.71 (CH_2), 32.00 (CH_2), 34.11 (CH_2), 49.70 (CH_3N^+), 49.87 (CH_3N^+), 51.21 (CH_3N^+), 51.51 (CH_3N^+), 61.82 (CH_2), 62.28 (CH_2), 65.73 (CH_2), 66.44 (CH_2), 101.51 ($\underline{C}HC_4H_9$), 122.95 (C_{pyr}), 134.69 (C_{pyr}), 136.74 (C_{pyr}), 146.52 (C_{pyr}), 150.93 (C_{pyr}); HRMS-ESI $[M-2Cl]^{2+}$ 322.8086 (calculated for $C_{41}H_{79}N_3O_2$, 322.8081).

5,8-Bis((*N*,*N*-dimethyl-*N*-(tetradecyl)ammonio)methyl)-2-butyl-4*H*-[1,3]dioxino[4,5-*c*]pyridine dichloride (5f$_{14}$)

Yield 83%; white solid; mp 185-186 °C (dec.); ^1H NMR ($CDCl_3$, 400 MHz) δ 0.85 (t, 6H, $^3J_{HH}$ = 6.7 Hz, $2\underline{CH_3}C_{13}H_{26}$), 0.91 (t, 3H, $^3J_{HH}$ = 7.1 Hz, $\underline{CH_3}C_3H_6$), 1.14-1.48 (m, 48H, $24CH_2$), 1.68-1.90 (m, 6H, $3CH_2$), 3.23-3.87 (m, 16H, $4CH_3N^+$ + $2CH_2N^+$), 4.70, 4.79 (AB, 2H, $^2J_{HH}$ = 12.6 Hz, CH_2), 5.08, 5.57 (AB, 2H, $^2J_{HH}$ = 16.2 Hz, CH_2), 5.15, 5.27 (AB, 2H, $^2J_{HH}$ = 13.2 Hz, CH_2), 5.45 (t, 1H, $^3J_{HH}$ = 5.1 Hz, CH), 8.64 (s, 1H, CH_{pyr}); ^{13}C NMR ($CDCl_3$, 100 MHz) δ 14.10 (CH_3), 14.23 (CH_3), 22.57 (CH_2), 22.78 (CH_2), 23.20 (CH_2), 25.80 (CH_2), 26.48 (CH_2), 29.46 (CH_2), 29.53 (CH_2), 29.56 (CH_2), 29.63 (CH_2), 29.72 (CH_2), 29.75 (CH_2), 29.78 (CH_2), 32.01 (CH_2), 34.09 (CH_2), 49.56 (CH_3N^+), 49.76 (CH_3N^+), 51.13 (CH_3N^+), 51.38 (CH_3N^+), 61.69 (CH_2), 62.20 (CH_2), 65.71 (CH_2), 66.38 (CH_2), 101.47 ($\underline{C}HC_4H_9$), 123.01 (C_{pyr}), 134.87 (C_{pyr}), 136.59 (C_{pyr}), 146.36 (C_{pyr}), 150.97 (C_{pyr}); HRMS-ESI $[M-2Cl]^{2+}$ 350.8402 (calculated for $C_{45}H_{87}N_3O_2$, 350.8394).

5,8-Bis((*N*,*N*-dimethyl-*N*-(hexadecyl)ammonio)methyl)-2-butyl-4*H*-[1,3]dioxino[4,5-*c*]pyridine dichloride (5f$_{16}$)

Yield 79%; white solid; mp 183-185 °C (dec.); ^{1}H NMR ($CDCl_3$, 400 MHz) δ 0.85 (t, 6H, $^3J_{HH}$ = 6.7 Hz, 2$\underline{CH_3}C_{15}H_{30}$), 0.91 (t, 3H, $^3J_{HH}$ = 7.0 Hz, $\underline{CH_3}C_3H_6$), 1.11-1.52 (m, 56H, 28CH_2), 1.65-1.91 (m, 6H, 3CH_2), 3.14-3.44 (m, 13H, 4CH_3N^+ + CH_2N^+), 3.46-3.57 (m, 1H, CH_2N^+), 3.58-3.70 (m, 1H, CH_2N^+), 3.76-3.88 (m, 1H, CH_2N^+), 4.69, 4.78 (AB, 2H, $^2J_{HH}$ = 12.6 Hz, CH_2), 5.07, 5.56 (AB, 2H, $^2J_{HH}$ = 16.2 Hz, CH_2), 5.14, 5.26 (AB, 2H, $^2J_{HH}$ = 13.2 Hz, CH_2), 5.44 (t, 1H, $^3J_{HH}$ = 5.1 Hz, CH), 8.63 (s, 1H, CH_{pyr}); ^{13}C NMR ($CDCl_3$, 100 MHz) δ 14.08 (CH_3), 14.21 (CH_3), 22.55 (CH_2), 22.77 (CH_2), 23.19 (CH_2), 25.79 (CH_2), 26.47 (CH_2), 29.45 (CH_2), 29.53 (CH_2), 29.56 (CH_2), 29.63 (CH_2), 29.73 (CH_2), 29.75 (CH_2), 29.79 (CH_2), 32.00 (CH_2), 34.08 (CH_2), 49.54 (CH_3N^+), 49.72 (CH_3N^+), 51.10 (CH_3N^+), 51.37 (CH_3N^+), 61.70 (CH_2), 62.20 (CH_2), 65.68 (CH_2), 66.34 (CH_2), 101.43 ($\underline{C}HC_4H_9$), 122.96 (C_{pyr}), 134.75 (C_{pyr}), 136.65 (C_{pyr}), 146.42 (C_{pyr}), 150.91 (C_{pyr}); HRMS-ESI [M-2Cl]$^{2+}$ 378.8707 (calculated for $C_{49}H_{95}N_3O_2$, 378.8707).

5,8-Bis((*N,N*-dimethyl-*N*-(octadecyl)ammonio)methyl)-2-butyl-4*H*-[1,3]dioxino[4,5-*c*]pyridine dichloride (5f$_{18}$)

Yield 34%; white solid; mp 175-177 °C (dec.); ^{1}H NMR ($CDCl_3$, 400 MHz) δ 0.85 (t, 6H, $^3J_{HH}$ = 6.7 Hz, 2$\underline{CH_3}C_{17}H_{34}$), 0.91 (t, 3H, $^3J_{HH}$ = 7.0 Hz, $\underline{CH_3}C_3H_6$), 1.12-1.51 (m, 64H, 32CH_2), 1.66-1.90 (m, 6H, 3CH_2), 3.18-3.44 (m, 13H, 4CH_3N^+ + CH_2N^+), 3.45-3.56 (m, 1H, CH_2N^+), 3.58-3.72 (m, 1H, CH_2N^+), 3.76-3.88 (m, 1H, CH_2N^+), 4.67, 4.73 (AB, 2H, $^2J_{HH}$ = 12.6 Hz, CH_2), 5.08, 5.53 (AB, 2H, $^2J_{HH}$ = 16.2 Hz, CH_2), 5.10, 5.20 (AB, 2H, $^2J_{HH}$ = 13.2 Hz, CH_2), 5.44 (t, 1H, $^3J_{HH}$ = 4.8 Hz, CH), 8.59 (s, 1H, CH_{pyr}); ^{13}C NMR ($CDCl_3$, 100 MHz) δ 14.10 (CH_3), 14.23 (CH_3), 22.57 (CH_2), 22.79 (CH_2), 23.20 (CH_2), 25.80 (CH_2), 26.48 (CH_2), 29.46 (CH_2), 29.52 (CH_2), 29.55 (CH_2), 29.57 (CH_2), 29.65 (CH_2), 29.76 (CH_2), 29.81 (CH_2), 32.02 (CH_2), 34.10 (CH_2), 49.58 (CH_3N^+), 49.75 (CH_3N^+), 51.09 (CH_3N^+), 51.41 (CH_3N^+), 61.95 (CH_2), 62.31 (CH_2), 65.66 (CH_2), 66.32 (CH_2), 101.36 ($\underline{C}HC_4H_9$), 122.78 (C_{pyr}), 134.31 (C_{pyr}), 136.98 (C_{pyr}), 146.72 (C_{pyr}), 150.78 (C_{pyr}); HRMS-ESI [M-2Cl]$^{2+}$ 406.9026 (calculated for $C_{53}H_{103}N_3O_2$, 406.9020).

5,8-Bis((*N,N*-dimethyl-*N*-(octyl)ammonio)methyl)-2-(*tert*-butyl)-4*H*-[1,3]dioxino[4,5-*c*]pyridine dichloride (5g$_8$)

Yield 64%; white solid; mp 175-177 °C (dec.); ^{1}H NMR ($CDCl_3$, 400 MHz) δ 0.84-0.89 (m, 6H, 2$\underline{CH_3}C_7H_{14}$), 1.01 (s, 9H, $C(CH_3)_3$), 1.21-1.43 (m, 20H, 10CH_2), 1.73-1.88 (m,

4H, 2CH_2), 3.33-3.38 (m, 12H, 4CH_3N^+), 3.50-3.89 (m, 4H, 2CH_2N^+), 4.68, 4.77 (AB, 2H, $^2J_{HH}$ = 12.4 Hz, CH_2), 5.11 (s, 1H, CH), 5.11, 5.32 (AB, 2H, $^2J_{HH}$ = 14.0 Hz, CH_2), 5.11, 5.71 (AB, 2H, $^2J_{HH}$ = 16.0 Hz, CH_2), 8.60 (s, 1H, CH_{pyr}); ^{13}C NMR ($CDCl_3$, 100 MHz) δ 14.19 (CH_3), 22.70 (CH_2), 23.20 (CH_2), 24.45 (C($\underline{C}H_3)_3$), 26.42 (CH_2), 26.47 (CH_2), 29.13 (CH_2), 29.17 (CH_2), 29.36 (CH_2), 29.46 (CH_2), 31.78 (CH_2), 35.15 ($\underline{C}(CH_3)_3$), 49.66 (CH_3N^+), 49.83 (CH_3N^+), 50.87 (CH_3N^+), 51.25 (CH_3N^+), 61.65 (CH_2), 62.17 (CH_2), 65.72 (CH_2), 66.04 (CH_2), 66.81 (CH_2), 106.36 ($\underline{CH}C(CH_3)_3$), 123.31 (C_{pyr}), 135.58 (C_{pyr}), 136.34 (C_{pyr}), 145.91 (C_{pyr}), 151.52 (C_{pyr}); HRMS-ESI [M-2Cl]$^{2+}$ 266.7457 (calculated for $C_{33}H_{63}N_3O_2$, 266.7455).

5,8-Bis((*N,N*-dimethyl-*N*-(decyl)ammonio)methyl)-2-(*tert*-butyl)-4*H*-[1,3]dioxino[4,5-*c*]pyridine dichloride (5g$_{10}$)

Yield 54%; white solid; mp 182-183 °C (dec.); ^{1}H NMR ($CDCl_3$, 400 MHz) δ 0.85 (t, 6H, $^3J_{HH}$ = 6.7 Hz, 2$\underline{CH_3}C_9H_{18}$), 1.00 (s, 9H, $C(CH_3)_3$), 1.15-1.46 (m, 28H, 14CH_2), 1.68-1.91 (m, 4H, 2CH_2), 3.22-3.46 (m, 12H, 4CH_3N^+), 3.50-3.88 (m, 4H, 2CH_2N^+), 4.64, 4.77 (AB, 2H, $^2J_{HH}$ = 12.4 Hz, CH_2), 5.09 (s, 1H, CH), 5.09, 5.27 (AB, 2H, $^2J_{HH}$ = 12.7 Hz, CH_2), 5.10, 5.67 (AB, 2H, $^2J_{HH}$ = 16.2 Hz, CH_2), 8.58 (s, 1H, CH_{pyr}); ^{13}C NMR ($CDCl_3$, 100 MHz) δ 14.21 (CH_3), 22.76 (CH_2), 23.19 (CH_2), 24.44 (C($\underline{C}H_3)_3$), 26.42 (CH_2), 26.47 (CH_2), 29.35 (CH_2), 29.41 (CH_2), 29.48 (CH_2), 29.51 (CH_2), 29.55 (CH_2), 29.57 (CH_2), 31.92 (CH_2), 31.93 (CH_2), 35.11 ($\underline{C}(CH_3)_3$), 49.64 (CH_3N^+), 49.80 (CH_3N^+), 50.79 (CH_3N^+), 51.26 (CH_3N^+), 61.77 (CH_2), 62.19 (CH_2), 65.65 (CH_2), 65.99 (CH_2), 66.72 (CH_2), 106.26 ($\underline{CH}C(CH_3)_3$), 123.08 (C_{pyr}), 135.07 (C_{pyr}), 136.69 (C_{pyr}), 146.23 (C_{pyr}), 151.30 (C_{pyr}); HRMS-ESI [M-2Cl]$^{2+}$ 294.7772 (calculated for $C_{37}H_{71}N_3O_2$, 294.7768).

5,8-Bis((*N,N*-dimethyl-*N*-(dodecyl)ammonio)methyl)-2-(*tert*-butyl)-4*H*-[1,3]dioxino[4,5-*c*]pyridine dichloride (5g$_{12}$)

Yield 70%; white solid; mp 181-182 °C (dec.); ^{1}H NMR ($CDCl_3$, 400 MHz) δ 0.86 (t, 6H, $^3J_{HH}$ = 6.7 Hz, 2$\underline{CH_3}C_{11}H_{22}$), 1.01 (s, 9H, $C(CH_3)_3$), 1.19-1.41 (m, 36H, 18CH_2), 1.73-1.85 (m, 4H, 2CH_2), 3.28-3.45 (m, 12H, 4CH_3N^+), 3.50-3.89 (m, 4H, 2CH_2N^+), 4.67, 4.79 (AB, 2H, $^2J_{HH}$ = 12.7 Hz, CH_2), 5.11 (s, 1H, CH), 5.11, 5.32 (AB, 2H, $^2J_{HH}$ = 13.1 Hz, CH_2), 5.11, 5.71 (AB, 2H, $^2J_{HH}$ = 16.3 Hz, CH_2), 8.60 (s, 1H, CH_{pyr}); ^{13}C NMR ($CDCl_3$, 100 MHz) δ 14.25 (CH_3), 22.80 (CH_2), 23.22 (CH_2), 24.46 (C($\underline{C}H_3)_3$), 26.44 (CH_2), 26.49 (CH_2), 29.45 (CH_2), 29.50 (CH_2), 29.54 (CH_2), 29.61 (CH_2), 29.64 (CH_2), 29.71 (CH_2), 32.02 (CH_2), 35.14

($\underline{C}$(CH$_3$)$_3$), 49.67 (CH$_3$N$^+$), 49.84 (CH$_3$N$^+$), 50.85 (CH$_3$N$^+$), 51.25 (CH$_3$N$^+$), 61.69 (CH$_2$), 62.17 (CH$_2$), 65.77 (CH$_2$), 66.04 (CH$_2$), 66.82 (CH$_2$), 106.35 ($\underline{C}$HC(CH$_3$)$_3$), 123.27 (C$_{pyr}$), 135.52 (C$_{pyr}$), 136.38 (C$_{pyr}$), 145.93 (C$_{pyr}$), 151.50 (C$_{pyr}$); HRMS-ESI [M-2Cl]$^{2+}$ 322.8087 (calculated for $C_{41}H_{79}N_3O_2$, 322.8081).

5,8-Bis((*N,N*-dimethyl-*N*-(tetradecyl)ammonio)methyl)-2-(*tert*-butyl)-4*H*-[1,3]dioxino[4,5-*c*]pyridine dichloride (5g$_{14}$)

Yield 49%; white solid; mp 180-181 °C (dec.); ^{1}H NMR (CDCl$_3$, 400 MHz) δ 0.86 (t, 6H, $^3J_{HH}$ = 6.7 Hz, 2$\underline{CH_3}$C$_{13}$H$_{26}$), 1.01 (s, 9H, C(CH$_3$)$_3$), 1.22-1.41 (m, 44H, 22CH$_2$), 1.69-1.90 (m, 4H, 2CH$_2$), 3.32-3.38 (m, 12H, 4CH$_3$N$^+$), 3.52-3.86 (m, 4H, 2CH$_2$N$^+$), 4.66, 4.79 (AB, 2H, $^2J_{HH}$ = 12.5 Hz, CH$_2$), 5.11 (s, 1H, CH), 5.09, 5.33 (AB, 2H, $^2J_{HH}$ = 13.4 Hz, CH$_2$), 5.10, 5.70 (AB, 2H, $^2J_{HH}$ = 16.3 Hz, CH$_2$), 8.59 (s, 1H, CH$_{pyr}$); ^{13}C NMR (CDCl$_3$, 100 MHz) δ 14.25 (CH$_3$), 22.80 (CH$_2$), 23.20 (CH$_2$), 23.24 (CH$_2$), 24.46 (C($\underline{C}$H$_3$)$_3$), 26.45 (CH$_2$), 26.49 (CH$_2$), 29.44 (CH$_2$), 29.48 (CH$_2$), 29.51 (CH$_2$), 29.54 (CH$_2$), 29.62 (CH$_2$), 29.63 (CH$_2$), 29.73 (CH$_2$), 29.77 (CH$_2$), 29.80 (CH$_2$), 32.03 (CH$_2$), 35.14 ($\underline{C}$(CH$_3$)$_3$), 49.65 (CH$_3$N$^+$), 49.84 (CH$_3$N$^+$), 50.85 (CH$_3$N$^+$), 51.25 (CH$_3$N$^+$), 61.79 (CH$_2$), 62.21 (CH$_2$), 65.77 (CH$_2$), 66.04 (CH$_2$), 66.77 (CH$_2$), 106.31 ($\underline{CH}$C(CH$_3$)$_3$), 123.15 (C$_{pyr}$), 135.24 (C$_{pyr}$), 136.57 (C$_{pyr}$), 146.11 (C$_{pyr}$), 151.39 (C$_{pyr}$); HRMS-ESI [M-2Cl]$^{2+}$ 350.8399 (calculated for $C_{45}H_{87}N_3O_2$, 350.8394).

5,8-Bis((*N,N*-dimethyl-*N*-(hexadecyl)ammonio)methyl)-2-(*tert*-butyl)-4*H*-[1,3]dioxino[4,5-*c*]pyridine dichloride (5g$_{16}$)

Yield 50%; white solid; mp 182-183 °C (dec.); ^{1}H NMR (CDCl$_3$, 400 MHz) δ 0.86 (t, 6H, $^3J_{HH}$ = 6.7 Hz, 2$\underline{CH_3}$C$_{15}$H$_{30}$), 1.01 (s, 9H, C(CH$_3$)$_3$), 1.17-1.40 (m, 52H, 26CH$_2$), 1.68-1.94 (m, 4H, 2CH$_2$), 3.22-3.45 (m, 12H, 4CH$_3$N$^+$), 3.49-3.87 (m, 4H, 2CH$_2$N$^+$), 4.65, 4.79 (AB, 2H, $^2J_{HH}$ = 12.7 Hz, CH$_2$), 5.09 (s, 1H, CH), 5.09, 5.33 (AB, 2H, $^2J_{HH}$ = 13.3 Hz, CH$_2$), 5.10, 5.71 (AB, 2H, $^2J_{HH}$ = 16.5 Hz, CH$_2$), 8.59 (s, 1H, CH$_{pyr}$); ^{13}C NMR (CDCl$_3$, 100 MHz) δ 14.25 (CH$_3$), 22.81 (CH$_2$), 23.21 (CH$_2$), 23.23 (CH$_2$), 24.46 (C($\underline{C}$H$_3$)$_3$), 26.45 (CH$_2$), 26.49 (CH$_2$), 29.44 (CH$_2$), 29.48 (CH$_2$), 29.51 (CH$_2$), 29.54 (CH$_2$), 29.63 (CH$_2$), 29.64 (CH$_2$), 29.73 (CH$_2$), 29.78 (CH$_2$), 29.82 (CH$_2$), 32.04 (CH$_2$), 35.14 ($\underline{C}$(CH$_3$)$_3$), 49.65 (CH$_3$N$^+$), 49.84 (CH$_3$N$^+$), 50.84 (CH$_3$N$^+$), 51.25 (CH$_3$N$^+$), 61.79 (CH$_2$), 62.20 (CH$_2$), 65.78 (CH$_2$), 66.04 (CH$_2$), 66.77 (CH$_2$), 106.31 ($\underline{CH}$C(CH$_3$)$_3$), 123.14 (C$_{pyr}$), 135.23 (C$_{pyr}$), 136.58 (C$_{pyr}$), 146.12 (C$_{pyr}$), 151.38 (C$_{pyr}$); HRMS-ESI [M-2Cl]$^{2+}$ 378.8711 (calculated for $C_{49}H_{95}N_3O_2$, 378.8707).

5,8-Bis((*N,N*-dimethyl-*N*-(octadecyl)ammonio)methyl)-2-(*tert*-butyl)-4*H*-[1,3]dioxino[4,5-*c*]pyridine dichloride (5g$_{18}$)

Yield 74%; white solid; mp 184-185 °C (dec.); ^{1}H NMR ($CDCl_3$, 400 MHz) δ 0.86 (t, 6H, $^3J_{HH}$ = 6.7 Hz, 2$\underline{CH_3}C_{17}H_{34}$), 1.02 (s, 9H, $C(CH_3)_3$), 1.10-1.46 (m, 60H, 30CH_2), 1.69-1.94 (m, 4H, 2CH_2), 3.29-3.50 (m, 12H, 4CH_3N^+), 3.49-3.97 (m, 4H, 2CH_2N^+), 4.69, 4.82 (AB, 2H, $^2J_{HH}$ = 12.4 Hz, CH_2), 5.12 (s, 1H, CH), 5.11, 5.38 (AB, 2H, $^2J_{HH}$ = 13.2 Hz, CH_2), 5.13, 5.75 (AB, 2H, $^2J_{HH}$ = 16.3 Hz, CH_2), 8.61 (s, 1H, CH_{pyr}); ^{13}C NMR ($CDCl_3$, 100 MHz) δ 14.27 (CH_3), 22.83 (CH_2), 23.24 (CH_2), 23.26 (CH_2), 24.47 ($C(\underline{C}H_3)_3$), 26.46 (CH_2), 26.50 (CH_2), 29.45 (CH_2), 29.50 (CH_2), 29.55 (CH_2), 29.65 (CH_2), 29.66 (CH_2), 29.75 (CH_2), 29.80 (CH_2), 29.84 (CH_2), 32.06 (CH_2), 35.17 ($\underline{C}(CH_3)_3$), 49.67 (CH_3N^+), 49.87 (CH_3N^+), 50.92 (CH_3N^+), 51.25 (CH_3N^+), 61.62 (CH_2), 62.16 (CH_2), 65.86 (CH_2), 66.10 (CH_2), 66.93 (CH_2), 106.44 ($\underline{CH}C(CH_3)_3$), 123.48 (C_{pyr}), 136.04 (C_{pyr}), 136.21 (C_{pyr}), 145.64 (C_{pyr}), 151.70 (C_{pyr}); HRMS-ESI $[M-2Cl]^{2+}$ 406.9027 (calculated for $C_{53}H_{103}N_3O_2$, 406.9020).

5,8-Bis((*N,N*-dimethyl-*N*-(octyl)ammonio)methyl)-2-(*sec*-butyl)-4*H*-[1,3]dioxino[4,5-*c*]pyridine dichloride (a mixture of two diastereomers) (5h$_8$)

Yield 27%; white solid; mp 179-180 °C (dec.); ^{1}H NMR ($CDCl_3$, 400 MHz) δ 0.82-1.01 (m, 24H, 8CH_3), 1.12-1.90 (m, 54H, 26CH_2 + 2CH), 3.20-3.59 (m, 26H, 8CH_3N^+ + CH_2N^+), 3.61-3.75 (m, 4H, 2CH_2N^+), 3.76-3.89 (m, 2H, CH_2N^+), 4.65, 4.76 (2AB, 4H, $^2J_{HH}$ = 12.4 Hz, 2CH_2), 5.09, 5.61 (2AB, 4H, $^2J_{HH}$ = 16.1 Hz, 2CH_2), 5.14, 5.18 (2AB, 4H, $^2J_{HH}$ = 15.4 Hz, 2CH_2), 5.29 (br m, 2H, 2CH), 8.61 (s, 2H, 2CH_{pyr}); ^{13}C NMR ($CDCl_3$, 100 MHz) δ 11.46 (CH_3), 11.58 (CH_3), 13.13 (CH_3), 13.37 (CH_3), 14.16 (CH_3), 22.67 (CH_2), 23.17 (CH_2), 23.85 (CH_2), 23.97 (CH_2), 26.42 (CH_2), 26.45 (CH_2), 29.14 (CH_2), 29.16 (CH_2), 29.38 (CH_2), 29.43 (CH_2), 31.76 (CH_2), 38.86 ($CH\underline{C}H(CH_3)C_2H_5$), 38.94 ($CH\underline{C}H(CH_3)C_2H_5$), 49.61 ($CH_3N^+$), 49.75 ($CH_3N^+$), 50.87 ($CH_3N^+$), 50.98 ($CH_3N^+$), 51.34 ($CH_3N^+$), 61.55 ($CH_2$), 62.14 ($CH_2$), 65.58 ($CH_2$), 65.85 ($CH_2$), 66.51 ($CH_2$), 66.58 ($CH_2$), 103.89 ($\underline{C}HCH(CH_3)C_2H_5$), 104.00 ($\underline{C}HCH(CH_3)C_2H_5$), 123.14 ($C_{pyr}$), 135.11 ($C_{pyr}$), 136.55 ($C_{pyr}$), 146.21 ($C_{pyr}$), 151.18 ($C_{pyr}$); HRMS-ESI $[M-2Cl]^{2+}$ 266.7458 (calculated for $C_{33}H_{63}N_3O_2$, 266.7455).

5,8-Bis((*N,N*-dimethyl-*N*-(decyl)ammonio)methyl)-2-(*sec*-butyl)-4*H*-[1,3]dioxino[4,5-*c*]pyridine dichloride (a mixture of two diastereomers) (5h$_{10}$)

Yield 56%; white solid; mp 180-181 °C (dec.); ^{1}H NMR ($CDCl_3$, 400 MHz) δ 0.83 (t, 12H, $^{3}J_{HH}$ = 6.3 Hz, $4CH_3$), 0.91 (t, 6H, $^{3}J_{HH}$ = 7.3 Hz, $2CH_3$), 0.96 (d, 3H, $^{3}J_{HH}$ = 7.2 Hz, CH_3), 0.98 (d, 3H, $^{3}J_{HH}$ = 7.4 Hz, CH_3), 1.11-1.88 (m, 70H, $34CH_2$ + 2CH), 3.17-3.59 (m, 26H, $8CH_3N^+$ + CH_2N^+), 3.60-3.74 (m, 4H, $2CH_2N^+$), 3.75-3.88 (m, 2H, CH_2N^+), 4.63, 4.75 (2AB, 4H, $^{2}J_{HH}$ = 12.4 Hz, $2CH_2$), 5.07, 5.60 (2AB, 4H, $^{2}J_{HH}$ = 16.2 Hz, $2CH_2$), 5.14, 5.18 (2AB, 4H, $^{2}J_{HH}$ = 16.0 Hz, $2CH_2$), 5.26 (d, 2H, $^{3}J_{HH}$ = 3.1 Hz, 2CH), 8.60 (s, 2H, $2CH_{pyr}$); ^{13}C NMR ($CDCl_3$, 100 MHz) δ 11.44 (CH_3), 11.55 (CH_3), 13.11 (CH_3), 13.35 (CH_3), 14.17 (CH_3), 22.72 (CH_2), 23.16 (CH_2), 23.83 (CH_2), 23.94 (CH_2), 26.41 (CH_2), 26.44 (CH_2), 29.32 (CH_2), 29.43 (CH_2), 29.49 (CH_2), 29.54 (CH_2), 31.90 (CH_2), 38.83 ($CH\underline{C}H(CH_3)C_2H_5$), 38.92 ($CH\underline{C}H(CH_3)C_2H_5$), 49.55 ($CH_3N^+$), 49.68 ($CH_3N^+$), 50.82 ($CH_3N^+$), 50.93 ($CH_3N^+$), 51.31 ($CH_3N^+$), 61.57 ($CH_2$), 62.13 ($CH_2$), 65.52 ($CH_2$), 65.83 ($CH_2$), 66.44 ($CH_2$), 66.50 ($CH_2$), 103.82 ($\underline{C}HCH(CH_3)C_2H_5$), 103.93 ($\underline{C}HCH(CH_3)C_2H_5$), 123.02 ($C_{pyr}$), 134.88 ($C_{pyr}$), 136.66 ($C_{pyr}$), 146.33 ($C_{pyr}$), 151.06 ($C_{pyr}$); HRMS-ESI $[M-2Cl]^{2+}$ 294.7772 (calculated for $C_{37}H_{71}N_3O_2$, 294.7768).

5,8-Bis((*N*,*N*-dimethyl-*N*-(dodecyl)ammonio)methyl)-2-(*sec*-butyl)-4*H*-[1,3]dioxino[4,5-*c*]pyridine dichloride (a mixture of two diastereomers) (5h$_{12}$)

Yield 60%; white solid; mp 179-180 °C (dec.); ^{1}H NMR ($CDCl_3$, 400 MHz) δ 0.84 (t, 12H, $^{3}J_{HH}$ = 6.6 Hz, $4CH_3$), 0.92 (t, 6H, $^{3}J_{HH}$ = 7.4 Hz, $2CH_3$), 0.97 (d, 3H, $^{3}J_{HH}$ = 7.9 Hz, CH_3), 0.99 (d, 3H, $^{3}J_{HH}$ = 7.7 Hz, CH_3), 1.14-1.90 (m, 86H, $42CH_2$ + 2CH), 3.27 (s, 6H, $2CH_3N^+$), 3.31 (s, 12H, $4CH_3N^+$), 3.33 (s, 6H, $2CH_3N^+$), 3.44-3.57 (m, 2H, CH_2N^+), 3.58-3.73 (m, 4H, $2CH_2N^+$), 3.74-3.86 (m, 2H, CH_2N^+), 4.60, 4.71 (2AB, 4H, $^{2}J_{HH}$ = 12.5 Hz, $2CH_2$), 5.07, 5.56 (2AB, 4H, $^{2}J_{HH}$ = 16.2 Hz, $2CH_2$), 5.09, 5.13 (2AB, 4H, $^{2}J_{HH}$ = 17.7 Hz, $2CH_2$), 5.26 (d, 2H, $^{3}J_{HH}$ = 4.5 Hz, 2CH), 8.56 (s, 2H, $2CH_{pyr}$); ^{13}C NMR ($CDCl_3$, 100 MHz) δ 11.46 (CH_3), 11.57 (CH_3), 13.13 (CH_3), 13.35 (CH_3), 14.20 (CH_3), 22.75 (CH_2), 23.16 (CH_2), 23.84 (CH_2), 23.96 (CH_2), 26.43 (CH_2), 26.45 (CH_2), 29.41 (CH_2), 29.46 (CH_2), 29.51 (CH_2), 29.60 (CH_2), 29.68 (CH_2), 31.97 (CH_2), 38.85 ($CH\underline{C}H(CH_3)C_2H_5$), 38.93 ($CH\underline{C}H(CH_3)C_2H_5$), 49.56 ($CH_3N^+$), 49.69 ($CH_3N^+$), 50.76 ($CH_3N^+$), 50.86 ($CH_3N^+$), 51.33 ($CH_3N^+$), 61.88 ($CH_2$), 62.26 ($CH_2$), 65.52 ($CH_2$), 65.76 ($CH_2$), 66.37 ($CH_2$), 66.44 ($CH_2$), 103.71 ($\underline{C}HCH(CH_3)C_2H_5$), 103.80 ($\underline{C}HCH(CH_3)C_2H_5$), 122.70 ($C_{pyr}$), 134.14 ($C_{pyr}$), 137.14 ($C_{pyr}$), 146.79 ($C_{pyr}$), 150.81 ($C_{pyr}$); HRMS-ESI $[M-2Cl]^{2+}$ 322.8086 (calculated for $C_{41}H_{79}N_3O_2$, 322.8081).

5,8-Bis((*N*,*N*-dimethyl-*N*-(tetradecyl)ammonio)methyl)-2-(*sec*-butyl)-4*H*-[1,3]dioxino[4,5-*c*]pyridine dichloride (a mixture of two diastereomers) (5h$_{14}$)

Yield 66%; white solid; mp 178-180 °C (dec.); ^{1}H NMR ($CDCl_3$, 400 MHz) δ 0.87 (t, 12H, $^3J_{HH}$ = 6.3 Hz, 4CH_3), 0.96 (t, 6H, $^3J_{HH}$ = 7.3 Hz, 2CH_3), 1.02 (d, 3H, $^3J_{HH}$ = 8.1 Hz, CH_3), 1.04 (d, 3H, $^3J_{HH}$ = 7.9 Hz, CH_3), 1.14-1.95 (m, 102H, 50CH_2+2CH), 3.25-3.90 (m, 32H, 8CH_3N^+ + 4CH_2N^+), 4.65 (s, 4H, 2CH_2), 5.01-5.35 (m, 8H, 4CH_2+2CH), 5.62 (2A-part of 2AB, 2H, $^2J_{HH}$ = 16.0 Hz, 2CH_2), 8.50 (s, 2H, 2CH_{pyr}); ^{13}C NMR ($CDCl_3$, 100 MHz) δ 11.50 (CH_3), 11.60 (CH_3), 13.16 (CH_3), 13.39 (CH_3), 14.24 (CH_3), 22.79 (CH_2), 23.19 (CH_2), 23.87 (CH_2), 23.98 (CH_2), 26.45 (CH_2), 26.47 (CH_2), 29.47 (CH_2), 29.54 (CH_2), 29.64 (CH_2), 29.73 (CH_2), 29.76 (CH_2), 29.79 (CH_2), 32.02 (CH_2), 38.89 (CH$\underline{C}$H(CH_3)C_2H_5), 38.97 (CH$\underline{C}$H(CH_3)C_2H_5), 49.63 (CH_3N^+), 49.73 (CH_3N^+), 50.86 (CH_3N^+), 50.94 (CH_3N^+), 51.35 (CH_3N^+), 61.99 (CH_2), 62.30 (CH_2), 65.57 (CH_2), 65.78 (CH_2), 66.47 (CH_2), 103.75 ($\underline{C}$HCH(CH_3)C_2H_5), 103.85 ($\underline{C}$HCH(CH_3)C_2H_5), 122.69 (C_{pyr}), 134.18 (C_{pyr}), 137.15 (C_{pyr}), 146.78 (C_{pyr}), 150.88 (C_{pyr}); HRMS-ESI [M-2Cl]$^{2+}$ 350.8399 (calculated for $C_{45}H_{87}N_3O_2$, 350.8394).

5,8-Bis((*N*,*N*-dimethyl-*N*-(hexadecyl)ammonio)methyl)-2-(*sec*-butyl)-4*H*-[1,3]dioxino[4,5-*c*]pyridine dichloride (a mixture of two diastereomers) (5h$_{16}$)

Yield 64%; white solid; mp 175-178 °C (dec.); ^{1}H NMR ($CDCl_3$, 400 MHz) δ 0.84 (t, 12H, $^3J_{HH}$ = 6.7 Hz, 4CH_3), 0.92 (t, 6H, $^3J_{HH}$ = 7.4 Hz, 2CH_3), 0.97 (d, 3H, $^3J_{HH}$ = 7.0 Hz, CH_3), 1.00 (d, 3H, $^3J_{HH}$ = 7.1 Hz, CH_3), 1.11-1.46 (m, 108H, 54CH_2), 1.55-1.67 (m, 2H, 2CH), 1.68-1.89 (m, 8H, 4CH_2), 3.13-3.91 (m, 32H, 8CH_3N^+ + 4CH_2N^+), 4.65, 4.77 (2AB, 2H, $^2J_{HH}$ = 12.5 Hz, 2CH_2), 5.08, 5.61 (2AB, 4H, $^2J_{HH}$ = 16.1 Hz, 2CH_2), 5.15, 5.19 (2AB, 4H, $^2J_{HH}$ = 16.0 Hz, 2CH_2), 5.28 (d, 2H, $^3J_{HH}$ = 3.0 Hz, 2CH), 8.61 (s, 2H, 2CH_{pyr}); ^{13}C NMR ($CDCl_3$, 100 MHz) δ 11.46 (CH_3), 11.57 (CH_3), 13.13 (CH_3), 13.38 (CH_3), 14.21 (CH_3), 22.77 (CH_2), 23.18 (CH_2), 23.85 (CH_2), 23.96 (CH_2), 26.44 (CH_2), 26.47 (CH_2), 29.45 (CH_2), 29.53 (CH_2), 29.63 (CH_2), 29.75 (CH_2), 29.79 (CH_2), 32.00 (CH_2), 38.85 (CH$\underline{C}$H(CH_3)C_2H_5), 38.94 (CH$\underline{C}$H(CH_3)C_2H_5), 49.59 (CH_3N^+), 49.71 (CH_3N^+), 50.83 (CH_3N^+), 50.94 (CH_3N^+), 51.30 (CH_3N^+), 61.58 (CH_2), 62.15 (CH_2), 65.62 (CH_2), 65.84 (CH_2), 66.51 (CH_2), 66.57 (CH_2), 103.87 ($\underline{C}$HCH(CH_3)C_2H_5), 103.98 ($\underline{C}$HCH(CH_3)C_2H_5), 123.08 (C_{pyr}), 135.04 (C_{pyr}), 136.57

(C_{pyr}), 146.24 (C_{pyr}), 151.15 (C_{pyr}); HRMS-ESI $[M-2Cl]^{2+}$ 378.8704 (calculated for $C_{49}H_{95}N_3O_2$, 378.8707).

5,8-Bis((*N,N*-dimethyl-*N*-(octadecyl)ammonio)methyl)-2-(*sec*-butyl)-4*H*-[1,3]dioxino[4,5-*c*]pyridine dichloride (a mixture of two diastereomers) (5h18)

Yield 65%; white solid; mp 171-174 °C (dec.); ^1H NMR ($CDCl_3$, 400 MHz) δ 0.84 (t, 12H, $^3J_{HH}$ = 6.7 Hz, $4CH_3$), 0.92 (t, 6H, $^3J_{HH}$ = 7.3 Hz, $2CH_3$), 0.97 (d, 3H, $^3J_{HH}$ = 7.2 Hz, CH_3), 0.99 (d, 3H, $^3J_{HH}$ = 7.3 Hz, CH_3), 1.11-1.46 (m, 124H, $62CH_2$), 1.54-1.67 (m, 2H, 2CH), 1.68-1.93 (m, 8H, $4CH_2$), 3.03-3.96 (m, 32H, $8CH_3N^+ + 4CH_2N^+$), 4.64, 4.76 (2AB, 2H, $^2J_{HH}$ = 12.3 Hz, $2CH_2$), 5.07, 5.60 (2AB, 4H, $^2J_{HH}$ = 16.1 Hz, $2CH_2$), 5.15, 5.19 (2AB, 4H, $^2J_{HH}$ = 15.8 Hz, $2CH_2$), 5.27 (d, 2H, $^3J_{HH}$ = 1.9 Hz, 2CH), 8.60 (s, 2H, $2CH_{pyr}$); ^{13}C NMR ($CDCl_3$, 100 MHz) δ 11.47 (CH_3), 11.57 (CH_3), 13.13 (CH_3), 13.37 (CH_3), 14.21 (CH_3), 22.77 (CH_2), 23.18 (CH_2), 23.85 (CH_2), 23.96 (CH_2), 26.44 (CH_2), 26.46 (CH_2), 29.45 (CH_2), 29.53 (CH_2), 29.64 (CH_2), 29.75 (CH_2), 29.79 (CH_2), 32.00 (CH_2), 38.86 ($CH\underline{C}H(CH_3)C_2H_5$), 38.94 ($CH\underline{C}H(CH_3)C_2H_5$), 49.58 ($CH_3N^+$), 49.71 ($CH_3N^+$), 50.84 ($CH_3N^+$), 50.94 ($CH_3N^+$), 51.31 ($CH_3N^+$), 61.68 ($CH_2$), 62.19 ($CH_2$), 65.62 ($CH_2$), 65.84 ($CH_2$), 66.48 ($CH_2$), 66.53 ($CH_2$), 103.83 ($\underline{C}HCH(CH_3)C_2H_5$), 103.94 ($\underline{C}HCH(CH_3)C_2H_5$), 122.98 ($C_{pyr}$), 134.80 ($C_{pyr}$), 136.73 ($C_{pyr}$), 146.40 ($C_{pyr}$), 151.07 ($C_{pyr}$); HRMS-ESI $[M-2Cl]^{2+}$ 406.9024 (calculated for $C_{53}H_{103}N_3O_2$, 406.9020).

5,8-Bis((*N,N*-dimethyl-*N*-(octyl)ammonio)methyl)-2-pentyl-4*H*-[1,3]dioxino[4,5-*c*]pyridine dichloride (5i8)

Yield 55%; white solid; mp 178-180 °C (dec.); ^1H NMR ($CDCl_3$, 400 MHz) δ 0.84-0.91 (m, 9H, $2\underline{CH_3}C_7H_{14} + \underline{CH_3}C_4H_8$), 1.14-1.47 (m, 26H, $13CH_2$), 1.67-1.90 (m, 6H, $3CH_2$), 3.22-3.90 (m, 16H, $4CH_3N^+ + 2CH_2N^+$), 4.70, 4.79 (AB, 2H, $^2J_{HH}$ = 12.5 Hz, CH_2), 5.09, 5.58 (AB, 2H, $^2J_{HH}$ = 16.1 Hz, CH_2), 5.13, 5.25 (AB, 2H, $^2J_{HH}$ = 13.2 Hz, CH_2), 5.47 (br s, 1H, CH), 8.63 (s, 1H, CH_{pyr}); ^{13}C NMR ($CDCl_3$, 100 MHz) δ 14.10 (CH_3), 14.18 (CH_3), 22.66 (CH_2), 22.70 (CH_2), 23.19 (CH_2), 23.43 (CH_2), 26.47 (CH_2), 29.18 (CH_2), 29.20 (CH_2), 29.45 (CH_2), 31.65 (CH_2), 31.78 (CH_2), 34.41 (CH_2), 49.63 (CH_3N^+), 49.79 (CH_3N^+), 51.15 (CH_3N^+), 51.41 (CH_3N^+), 61.60 (CH_2), 62.20 (CH_2), 65.72 (CH_2), 66.47 (CH_2), 101.57 ($\underline{C}HC_5H_{11}$), 123.16 (C_{pyr}), 135.17 (C_{pyr}), 136.39 (C_{pyr}), 146.17 (C_{pyr}), 151.09 (C_{pyr}); HRMS-ESI $[M-2Cl]^{2+}$ 273.7533 (calculated for $C_{34}H_{65}N_3O_2$, 273.7533).

5,8-Bis((*N*,*N*-dimethyl-*N*-(decyl)ammonio)methyl)-2-pentyl-4*H*-[1,3]dioxino[4,5-*c*]pyridine dichloride ($5i_{10}$)

Yield 57%; white solid; mp 180-181 °C (dec.); ^{1}H NMR ($CDCl_3$, 400 MHz) δ 0.83-0.91 (m, 9H, 2$\underline{CH_3}C_9H_{18}$ + $\underline{CH_3}C_4H_8$), 1.13-1.50 (m, 34H, 17CH_2), 1.68-1.90 (m, 6H, 3CH_2), 3.23-3.89 (m, 16H, 4CH_3N^+ + 2CH_2N^+), 4.70, 4.78 (AB, 2H, $^2J_{HH}$ = 12.4 Hz, CH_2), 5.08, 5.57 (AB, 2H, $^2J_{HH}$ = 16.2 Hz, CH_2), 5.12, 5.25 (AB, 2H, $^2J_{HH}$ = 13.2 Hz, CH_2), 5.46 (t, 1H, $^3J_{HH}$ = 5.0 Hz, CH), 8.62 (s, 1H, CH_{pyr}); ^{13}C NMR ($CDCl_3$, 100 MHz) δ 14.09 (CH_3), 14.21 (CH_3), 22.66 (CH_2), 22.76 (CH_2), 23.20 (CH_2), 23.43 (CH_2), 26.48 (CH_2), 29.37 (CH_2), 29.53 (CH_2), 29.56 (CH_2), 31.64 (CH_2), 31.94 (CH_2), 34.40 (CH_2), 49.60 (CH_3N^+), 49.76 (CH_3N^+), 51.11 (CH_3N^+), 51.39 (CH_3N^+), 61.65 (CH_2), 62.22 (CH_2), 65.70 (CH_2), 66.44 (CH_2), 101.52 ($\underline{C}HC_5H_{11}$), 123.08 (C_{pyr}), 134.98 (C_{pyr}), 136.51 (C_{pyr}), 146.29 (C_{pyr}), 151.01 (C_{pyr}); HRMS-ESI $[M-2Cl]^{2+}$ 301.7852 (calculated for $C_{38}H_{73}N_3O_2$, 301.7846).

5,8-Bis((*N*,*N*-dimethyl-*N*-(dodecyl)ammonio)methyl)-2-pentyl-4*H*-[1,3]dioxino[4,5-*c*]pyridine dichloride ($5i_{12}$)

Yield 62%; white solid; mp 180-182 °C (dec.); ^{1}H NMR ($CDCl_3$, 400 MHz) δ 0.87 (t, 6H, $^3J_{HH}$ = 7.0 Hz, 2$\underline{CH_3}C_{11}H_{22}$), 0.91 (t, 3H, $^3J_{HH}$ = 6.6 Hz, $\underline{CH_3}C_4H_8$), 1.15-1.49 (m, 42H, 21CH_2), 1.69-1.91 (m, 6H, 3CH_2), 3.26-3.90 (m, 16H, 4CH_3N^+ + 2CH_2N^+), 4.74, 4.82 (AB, 2H, $^2J_{HH}$ = 12.6 Hz, CH_2), 5.10, 5.60 (AB, 2H, $^2J_{HH}$ = 16.2 Hz, CH_2), 5.17, 5.31 (AB, 2H, $^2J_{HH}$ = 13.1 Hz, CH_2), 5.49 (t, 1H, $^3J_{HH}$ = 4.8 Hz, CH), 8.65 (s, 1H, CH_{pyr}); ^{13}C NMR ($CDCl_3$, 100 MHz) δ 14.12 (CH_3), 14.26 (CH_3), 22.68 (CH_2), 22.81 (CH_2), 23.23 (CH_2), 23.45 (CH_2), 26.49 (CH_2), 29.46 (CH_2), 29.52 (CH_2), 29.55 (CH_2), 29.58 (CH_2), 29.64 (CH_2), 29.65 (CH_2), 29.73 (CH_2), 31.66 (CH_2), 32.03 (CH_2), 34.41 (CH_2), 49.66 (CH_3N^+), 49.84 (CH_3N^+), 51.24 (CH_3N^+), 51.37 (CH_3N^+), 61.41 (CH_2), 62.14 (CH_2), 65.78 (CH_2), 65.87 (CH_2), 66.62 (CH_2), 101.70 ($\underline{C}HC_5H_{11}$), 123.41 (C_{pyr}), 135.20 (C_{pyr}), 135.99 (C_{pyr}), 145.76 (C_{pyr}), 151.36 (C_{pyr}); HRMS-ESI $[M-2Cl]^{2+}$ 329.8160 (calculated for $C_{42}H_{81}N_3O_2$, 329.8159).

5,8-Bis((*N*,*N*-dimethyl-*N*-(tetradecyl)ammonio)methyl)-2-pentyl-4*H*-[1,3]dioxino[4,5-*c*]pyridine dichloride ($5i_{14}$)

Yield 57%; white solid; mp 177-178 °C (dec.); ^{1}H NMR ($CDCl_3$, 400 MHz) δ 0.85 (t, 6H, $^3J_{HH}$ = 6.8 Hz, 2$\underline{CH_3}C_{13}H_{26}$), 0.89 (t, 3H, $^3J_{HH}$ = 6.3 Hz, $\underline{CH_3}C_4H_8$), 1.09-1.50 (m, 50H, 25CH_2), 1.66-1.89 (m, 6H, 3CH_2), 3.20-3.90 (m, 16H, 4CH_3N^+ + 2CH_2N^+), 4.68, 4.76 (AB,

2H, $^2J_{HH}$ = 12.3 Hz, CH_2), 5.07, 5.55 (AB, 2H, $^2J_{HH}$ = 16.1 Hz, CH_2), 5.11, 5.21 (AB, 2H, $^2J_{HH}$ = 13.0 Hz, CH_2), 5.45 (br s, 1H, CH), 8.61 (s, 1H, CH_{pyr}); ^{13}C NMR ($CDCl_3$, 100 MHz) δ 14.09 (CH_3), 14.22 (CH_3), 22.65 (CH_2), 22.78 (CH_2), 23.20 (CH_2), 23.42 (CH_2), 26.48 (CH_2), 29.46 (CH_2), 29.54 (CH_2), 29.57 (CH_2), 29.64 (CH_2), 29.75 (CH_2), 29.78 (CH_2), 31.64 (CH_2), 32.01 (CH_2), 34.40 (CH_2), 49.61 (CH_3N^+), 49.74 (CH_3N^+), 51.06 (CH_3N^+), 51.39 (CH_3N^+), 61.75 (CH_2), 62.25 (CH_2), 65.67 (CH_2), 66.40 (CH_2), 101.45 ($\underline{C}HC_5H_{11}$), 122.96 (C_{pyr}), 134.70 (C_{pyr}), 136.68 (C_{pyr}), 146.46 (C_{pyr}), 150.91 (C_{pyr}); HRMS-ESI $[M-2Cl]^{2+}$ 357.8477 (calculated for $C_{46}H_{89}N_3O_2$, 357.8472).

5,8-Bis((*N,N*-dimethyl-*N*-(hexadecyl)ammonio)methyl)-2-pentyl-4*H*-[1,3]dioxino[4,5-*c*]pyridine dichloride (5i$_{16}$)

Yield 54%; white solid; mp 172-175 °C (dec.); ^1H NMR ($CDCl_3$, 400 MHz) δ 0.86 (t, 6H, $^3J_{HH}$ = 6.7 Hz, $2\underline{CH_3}C_{15}H_{30}$), 0.91 (t, 3H, $^3J_{HH}$ = 6.3 Hz, $\underline{CH_3}C_4H_8$), 1.14-1.54 (m, 58H, $29CH_2$), 1.69-1.92 (m, 6H, $3CH_2$), 3.21-3.91 (m, 16H, $4CH_3N^+$ + $2CH_2N^+$), 4.74, 4.83 (AB, 2H, $^2J_{HH}$ = 11.8 Hz, CH_2), 5.11, 5.60 (AB, 2H, $^2J_{HH}$ = 15.9 Hz, CH_2), 5.15, 5.29 (AB, 2H, $^2J_{HH}$ = 12.5 Hz, CH_2), 5.50 (br s, 1H, CH), 8.65 (s, 1H, CH_{pyr}); ^{13}C NMR ($CDCl_3$, 100 MHz) δ 14.12 (CH_3), 14.26 (CH_3), 22.68 (CH_2), 22.82 (CH_2), 23.24 (CH_2), 23.45 (CH_2), 26.50 (CH_2), 29.50 (CH_2), 29.53 (CH_2), 29.57 (CH_2), 29.59 (CH_2), 29.67 (CH_2), 29.77 (CH_2), 29.80 (CH_2), 29.83 (CH_2), 31.67 (CH_2), 32.05 (CH_2), 34.42 (CH_2), 49.69 (CH_3N^+), 49.86 (CH_3N^+), 51.20 (CH_3N^+), 51.39 (CH_3N^+), 61.47 (CH_2), 62.17 (CH_2), 65.76 (CH_2), 65.87 (CH_2), 66.64 (CH_2), 101.68 ($\underline{C}HC_5H_{11}$), 123.38 (C_{pyr}), 135.73 (C_{pyr}), 136.03 (C_{pyr}), 145.81 (C_{pyr}), 151.34 (C_{pyr}); HRMS-ESI $[M-2Cl]^{2+}$ 385.8790 (calculated for $C_{50}H_{97}N_3O_2$, 385.8785).

5,8-Bis((*N,N*-dimethyl-*N*-(octadecyl)ammonio)methyl)-2-pentyl-4*H*-[1,3]dioxino[4,5-*c*]pyridine dichloride (5i$_{18}$)

Yield 65%; white solid; mp 174-175 °C (dec.); ^1H NMR ($CDCl_3$, 400 MHz) δ 0.87 (t, 6H, $^3J_{HH}$ = 6.8 Hz, $2\underline{CH_3}C_{17}H_{34}$), 0.91 (t, 3H, $^3J_{HH}$ = 6.7 Hz, $\underline{CH_3}C_4H_8$), 1.13-1.55 (m, 66H, $33CH_2$), 1.70-1.93 (m, 6H, $3CH_2$), 3.22-4.14 (m, 16H, $4CH_3N^+$ + $2CH_2N^+$), 4.76, 4.85 (AB, 2H, $^2J_{HH}$ = 12.6 Hz, CH_2), 5.11, 5.62 (AB, 2H, $^2J_{HH}$ = 16.2 Hz, CH_2), 5.18, 5.33 (AB, 2H, $^2J_{HH}$ = 13.1 Hz, CH_2), 5.50 (t, 1H, $^3J_{HH}$ = 5.0 Hz, CH), 8.66 (s, 1H, CH_{pyr}); ^{13}C NMR ($CDCl_3$, 100 MHz) δ 14.13 (CH_3), 14.27 (CH_3), 22.68 (CH_2), 22.83 (CH_2), 23.24 (CH_2), 23.45 (CH_2), 26.50 (CH_2), 29.50 (CH_2), 29.53 (CH_2), 29.57 (CH_2), 29.60 (CH_2), 29.67 (CH_2), 29.78 (CH_2), 29.81

(CH_2), 29.85 (CH_2), 31.66 (CH_2), 32.06 (CH_2), 34.41 (CH_2), 49.67 (CH_3N^+), 49.86 (CH_3N^+), 51.24 (CH_3N^+), 51.35 (CH_3N^+), 61.37 (CH_2), 62.13 (CH_2), 65.79 (CH_2), 65.91 (CH_2), 66.66 (CH_2), 101.73 ($\underline{C}HC_5H_{11}$), 123.50 (C_{pyr}), 135.82 (C_{pyr}), 136.05 (C_{pyr}), 145.60 (C_{pyr}), 151.45 (C_{pyr}); HRMS-ESI $[M-2Cl]^{2+}$ 413.9100 (calculated for $C_{54}H_{105}N_3O_2$, 413.9098).

5,8-Bis((*N*,*N*-dimethyl-*N*-(octyl)ammonio)methyl)-2-(pentan-2-yl)-4*H*-[1,3]dioxino[4,5-*c*]pyridine dichloride (a mixture of two diastereomers) (5j$_8$)

Yield 54%; white solid; mp 177-180 °C (dec.); ^{1}H NMR ($CDCl_3$, 400 MHz) δ 0.82-1.00 (m, 24H, $8CH_3$), 1.12-1.95 (m, 58H, $28CH_2$ + 2CH), 3.16-3.59 (m, 26H, $8CH_3N^+$ + CH_2N^+), 3.61-3.75 (m, 4H, $2CH_2N^+$), 3.76-3.88 (m, 2H, CH_2N^+), 4.64, 4.76 (2AB, 4H, $^2J_{HH}$ = 12.5 Hz, $2CH_2$), 5.08, 5.61 (2AB, 4H, $^2J_{HH}$ = 16.2 Hz, $2CH_2$), 5.15, 5.19 (2AB, 4H, $^2J_{HH}$ = 16.7 Hz, $2CH_2$), 5.25 (d, 1H, $^3J_{HH}$ = 5.4 Hz, CH), 5.27 (d, 1H, $^3J_{HH}$ = 4.9 Hz, CH), 8.61 (s, 2H, $2CH_{pyr}$); ^{13}C NMR ($CDCl_3$, 100 MHz) δ 13.55 (CH_3), 13.84 (CH_3), 14.15 (CH_3), 14.37 (CH_3), 14.41 (CH_3), 20.04 (CH_2), 22.66 (CH_2), 23.15 (CH_2), 26.43 (CH_2), 29.14 (CH_2), 29.37 (CH_2), 29.42 (CH_2), 31.75 (CH_2), 33.22 (CH_2), 33.29 (CH_2), 37.07 ($CH\underline{C}H(CH_3)C_3H_7$), 49.58 ($CH_3N^+$), 49.70 ($CH_3N^+$), 50.83 ($CH_3N^+$), 50.93 ($CH_3N^+$), 51.35 ($CH_3N^+$), 61.51 ($CH_2$), 62.14 ($CH_2$), 65.56 ($CH_2$), 65.86 ($CH_2$), 66.51 ($CH_2$), 66.55 ($CH_2$), 103.98 ($\underline{C}HCH(CH_3)C_3H_7$), 104.12 ($\underline{C}HCH(CH_3)C_3H_7$), 123.08 ($C_{pyr}$), 135.00 ($C_{pyr}$), 136.60 ($C_{pyr}$), 146.28 ($C_{pyr}$), 151.11 ($C_{pyr}$), 151.16 ($C_{pyr}$); HRMS-ESI $[M-2Cl]^{2+}$ 275.7539 (calculated for $C_{34}H_{65}N_3O_2$, 275.7533).

5,8-Bis((*N*,*N*-dimethyl-*N*-(decyl)ammonio)methyl)-2-(pentan-2-yl)-4*H*-[1,3]dioxino[4,5-*c*]pyridine dichloride (a mixture of two diastereomers) (5j$_{10}$)

Yield 64%; white solid; mp 177-178 °C (dec.); ^{1}H NMR ($CDCl_3$, 400 MHz) δ 0.84 (t, 12H, $^3J_{HH}$ = 6.5 Hz, $4CH_3$), 0.88 (t, 6H, $^3J_{HH}$ = 6.9 Hz, $2CH_3$), 0.97 (d, 3H, $^3J_{HH}$ = 7.7 Hz, CH_3), 0.99 (d, 3H, $^3J_{HH}$ = 7.9 Hz, CH_3), 1.11-1.92 (m, 74H, $36CH_2$ + 2CH), 3.15-3.58 (m, 26H, $8CH_3N^+$ + CH_2N^+), 3.60-3.74 (m, 4H, $2CH_2N^+$), 3.75-3.88 (m, 2H, CH_2N^+), 4.63, 4.75 (2AB, 4H, $^2J_{HH}$ = 12.3 Hz, $2CH_2$), 5.07, 5.59 (2AB, 4H, $^2J_{HH}$ = 16.2 Hz, $2CH_2$), 5.14 (br m, 4H, $2CH_2$), 5.26 (br m, 2H, 2CH), 8.60 (s, 2H, $2CH_{pyr}$); ^{13}C NMR ($CDCl_3$, 100 MHz) δ 13.56 (CH_3), 13.83 (CH_3), 14.18 (CH_3), 14.36 (CH_3), 14.40 (CH_3), 20.03 (CH_2), 22.73 (CH_2), 23.16 (CH_2), 26.44 (CH_2), 29.33 (CH_2), 29.44 (CH_2), 29.50 (CH_2), 29.54 (CH_2), 31.90 (CH_2), 33.21 (CH_2), 33.29 (CH_2), 37.06 ($CH\underline{C}H(CH_3)C_3H_7$), 49.59 ($CH_3N^+$), 49.70 ($CH_3N^+$), 50.76 ($CH_3N^+$), 50.87 ($CH_3N^+$), 51.35 ($CH_3N^+$), 61.54 ($CH_2$), 62.16 ($CH_2$), 65.55 ($CH_2$), 65.82

(CH_2), 66.53 (CH_2), 66.56 (CH_2), 103.95 ($\underline{C}HCH(CH_3)C_3H_7$), 104.08 ($\underline{C}HCH(CH_3)C_3H_7$), 123.01 ($C_{pyr}$), 134.84 ($C_{pyr}$), 136.69 ($C_{pyr}$), 146.37 ($C_{pyr}$), 151.09 ($C_{pyr}$); HRMS-ESI $[M-2Cl]^{2+}$ 301.7853 (calculated for $C_{38}H_{73}N_3O_2$, 301.7846).

5,8-Bis((*N,N*-dimethyl-*N*-(dodecyl)ammonio)methyl)-2-(pentan-2-yl)-*4H*-[1,3]dioxino[4,5-*c*]pyridine dichloride (a mixture of two diastereomers) (5j$_{12}$)

Yield 70%; white solid; mp 168-170 °C (dec.); ^{1}H NMR ($CDCl_3$, 400 MHz) δ 0.85 (t, 12H, $^3J_{HH}$ = 6.7 Hz, $4CH_3$), 0.89 (t, 6H, $^3J_{HH}$ = 6.9 Hz, $2CH_3$), 0.98 (d, 3H, $^3J_{HH}$ = 7.3 Hz, CH_3), 1.00 (d, 3H, $^3J_{HH}$ = 7.6 Hz, CH_3), 1.11-1.92 (m, 90H, $44CH_2$ + 2CH), 3.20-3.89 (m, 32H, $8CH_3N^+$ + $4CH_2N^+$), 4.65, 4.77 (2AB, 4H, $^2J_{HH}$ = 12.2 Hz, $2CH_2$), 5.08, 5.61 (2AB, 4H, $^2J_{HH}$ = 16.1 Hz, $2CH_2$), 5.16 (br m, 4H, $2CH_2$), 5.27 (br m, 2H, 2CH), 8.61 (s, 2H, $2CH_{pyr}$); ^{13}C NMR ($CDCl_3$, 100 MHz) δ 13.57 (CH_3), 13.86 (CH_3), 14.22 (CH_3), 14.38 (CH_3), 14.43 (CH_3), 20.05 (CH_2), 22.77 (CH_2), 23.19 (CH_2), 26.46 (CH_2), 29.42 (CH_2), 29.47 (CH_2), 29.53 (CH_2), 29.61 (CH_2), 29.69 (CH_2), 31.98 (CH_2), 33.23 (CH_2), 33.30 (CH_2), 37.08 ($CH\underline{C}H(CH_3)C_3H_7$), 49.62 ($CH_3N^+$), 49.73 ($CH_3N^+$), 50.80 ($CH_3N^+$), 50.90 ($CH_3N^+$), 51.35 ($CH_3N^+$), 61.50 ($CH_2$), 62.15 ($CH_2$), 65.63 ($CH_2$), 65.85 ($CH_2$), 66.59 ($CH_2$), 66.62 ($CH_2$), 104.01 ($\underline{C}HCH(CH_3)C_3H_7$), 104.15 ($\underline{C}HCH(CH_3)C_3H_7$), 123.11 ($C_{pyr}$), 135.10 ($C_{pyr}$), 136.53 ($C_{pyr}$), 146.22 ($C_{pyr}$), 151.16 ($C_{pyr}$); HRMS-ESI $[M-2Cl]^{2+}$ 329.8165 (calculated for $C_{42}H_{81}N_3O_2$, 329.8159).

5,8-Bis((*N,N*-dimethyl-*N*-(tetradecyl)ammonio)methyl)-2-(pentan-2-yl)-4*H*-[1,3]dioxino[4,5-*c*]pyridine dichloride (a mixture of two diastereomers) (5j$_{14}$)

Yield 57%; white solid; mp 174-176 °C (dec.); ^{1}H NMR ($CDCl_3$, 400 MHz) δ 0.85 (t, 12H, $^3J_{HH}$ = 6.7 Hz, $4CH_3$), 0.90 (t, 6H, $^3J_{HH}$ = 6.9 Hz, $2CH_3$), 0.98 (d, 3H, $^3J_{HH}$ = 7.1 Hz, CH_3), 1.00 (d, 3H, $^3J_{HH}$ = 7.1 Hz, CH_3), 1.12-1.95 (m, 106H, $52CH_2$ + 2CH), 3.22-3.90 (m, 32H, $8CH_3N^+$ + $4CH_2N^+$), 4.65, 4.78 (2AB, 4H, $^2J_{HH}$ = 12.4 Hz, $2CH_2$), 5.08, 5.62 (2AB, 4H, $^2J_{HH}$ = 16.2 Hz, $2CH_2$), 5.15, 5.19 (2AB, 4H, $^2J_{HH}$ = 16.9 Hz, $2CH_2$), 5.27 (d, 1H, $^3J_{HH}$ = 5.5 Hz, CH), 5.28 (d, 1H, $^3J_{HH}$ = 4.8 Hz, CH), 8.61 (s, 2H, $2CH_{pyr}$); ^{13}C NMR ($CDCl_3$, 100 MHz) δ 13.57 (CH_3), 13.87 (CH_3), 14.23 (CH_3), 14.39 (CH_3), 14.43 (CH_3), 20.06 (CH_2), 22.78 (CH_2), 23.20 (CH_2), 26.46 (CH_2), 29.46 (CH_2), 29.54 (CH_2), 29.63 (CH_2), 29.75 (CH_2), 29.78 (CH_2), 32.01 (CH_2), 33.24 (CH_2), 33.32 (CH_2), 37.09 ($CH\underline{C}H(CH_3)C_3H_7$), 49.62 ($CH_3N^+$), 49.74 ($CH_3N^+$), 50.81 ($CH_3N^+$), 50.92 ($CH_3N^+$), 51.34 ($CH_3N^+$), 61.48 ($CH_2$), 62.15 ($CH_2$), 65.69 ($CH_2$), 65.87

(CH_2), 66.63 (CH_2), 66.66 (CH_2), 104.04 ($\underline{C}HCH(CH_3)C_3H_7$), 104.18 ($\underline{C}HCH(CH_3)C_3H_7$), 123.17 ($C_{pyr}$), 135.25 ($C_{pyr}$), 136.42 ($C_{pyr}$), 146.13 ($C_{pyr}$), 151.23 ($C_{pyr}$), 151.28 ($C_{pyr}$); HRMS-ESI $[M-2Cl]^{2+}$ 357.8477 (calculated for $C_{46}H_{89}N_3O_2$, 357.8472).

5,8-Bis((*N,N*-dimethyl-*N*-(hexadecyl)ammonio)methyl)-2-(pentan-2-yl)-4*H*-[1,3]dioxino[4,5-*c*]pyridine dichloride (a mixture of two diastereomers) (5j$_{16}$)

Yield 65%; white solid; mp 176-180 °C (dec.); ^{1}H NMR ($CDCl_3$, 400 MHz) δ 0.85 (t, 12H, $^3J_{HH}$ = 6.7 Hz, $4CH_3$), 0.90 (t, 6H, $^3J_{HH}$ = 6.9 Hz, $2CH_3$), 0.98 (d, 3H, $^3J_{HH}$ = 6.9 Hz, CH_3), 1.01 (d, 3H, $^3J_{HH}$ = 7.0 Hz, CH_3), 1.12-1.94 (m, 122H, $60CH_2$+2CH), 3.20-3.92 (m, 32H, $8CH_3N^+$ + $4CH_2N^+$), 4.66, 4.79 (2AB, 2H, $^2J_{HH}$ = 12.4 Hz, $2CH_2$), 5.08, 5.62 (2AB, 4H, $^2J_{HH}$ = 16.1 Hz, $2CH_2$), 5.18, 5.19 (2AB, 4H, $^2J_{HH}$ = 16.4 Hz, $2CH_2$), 5.27 (d, 1H, $^3J_{HH}$ = 5.9 Hz, CH), 5.28 (d, 1H, $^3J_{HH}$ = 5.4 Hz, CH), 8.62 (s, 2H, $2CH_{pyr}$); ^{13}C NMR ($CDCl_3$, 100 MHz) δ 13.57 (CH_3), 13.87 (CH_3), 14.23 (CH_3), 14.39 (CH_3), 14.43 (CH_3), 20.06 (CH_2), 22.78 (CH_2), 23.20 (CH_2), 26.46 (CH_2), 29.46 (CH_2), 29.54 (CH_2), 29.63 (CH_2), 29.76 (CH_2), 29.80 (CH_2), 32.01 (CH_2), 33.24 (CH_2), 33.31 (CH_2), 37.09 ($CH\underline{C}H(CH_3)C_3H_7$), 49.60 ($CH_3N^+$), 49.74 ($CH_3N^+$), 50.84 ($CH_3N^+$), 50.93 ($CH_3N^+$), 51.33 ($CH_3N^+$), 61.49 ($CH_2$), 62.15 ($CH_2$), 65.71 ($CH_2$), 65.88 ($CH_2$), 66.60 ($CH_2$), 104.03 ($\underline{C}HCH(CH_3)C_3H_7$), 104.19 ($\underline{C}HCH(CH_3)C_3H_7$), 123.15 ($C_{pyr}$), 135.25 ($C_{pyr}$), 136.44 ($C_{pyr}$), 146.13 ($C_{pyr}$), 151.22 ($C_{pyr}$); HRMS-ESI $[M-2Cl]^{2+}$ 385.8792 (calculated for $C_{50}H_{97}N_3O_2$, 385.8785).

5,8-Bis((*N,N*-dimethyl-*N*-(octadecyl)ammonio)methyl)-2-(pentan-2-yl)-4*H*-[1,3]dioxino[4,5-*c*]pyridine dichloride (a mixture of two diastereomers) (5j$_{18}$)

Yield 65%; white solid; mp 171-172 °C (dec.); ^{1}H NMR ($CDCl_3$, 400 MHz) δ 0.84 (t, 12H, $^3J_{HH}$ = 6.7 Hz, $4CH_3$), 0.89 (t, 6H, $^3J_{HH}$ = 6.9 Hz, $2CH_3$), 0.97 (d, 3H, $^3J_{HH}$ = 7.1 Hz, CH_3), 0.99 (d, 3H, $^3J_{HH}$ = 7.1 Hz, CH_3), 1.10-1.92 (m, 138H, $68CH_2$+2CH), 3.20-3.90 (m, 32H, $8CH_3N^+$ + $4CH_2N^+$), 4.64, 4.77 (2AB, 4H, $^2J_{HH}$ = 12.3 Hz, $2CH_2$), 5.07, 5.60 (2AB, 4H, $^2J_{HH}$ = 16.1 Hz, $2CH_2$), 5.16, 5.17 (2AB, 4H, $^2J_{HH}$ = 16.0 Hz, $2CH_2$), 5.25 (d, 1H, $^3J_{HH}$ = 5.5 Hz, CH), 5.26 (d, 1H, $^3J_{HH}$ = 5.0 Hz, CH), 8.61 (s, 2H, $2CH_{pyr}$); ^{13}C NMR ($CDCl_3$, 100 MHz) δ 13.56 (CH_3), 13.84 (CH_3), 14.21 (CH_3), 14.37 (CH_3), 14.42 (CH_3), 20.04 (CH_2), 22.76 (CH_2), 23.17 (CH_2), 26.45 (CH_2), 29.44 (CH_2), 29.53 (CH_2), 29.63 (CH_2), 29.74 (CH_2), 29.79 (CH_2), 31.99 (CH_2), 33.22 (CH_2), 33.30 (CH_2), 37.07 ($CH\underline{C}H(CH_3)C_3H_7$), 49.56 ($CH_3N^+$), 49.69 ($CH_3N^+$), 50.77 ($CH_3N^+$), 50.86 ($CH_3N^+$), 51.32 ($CH_3N^+$), 61.57 ($CH_2$), 62.17 ($CH_2$), 65.61 ($CH_2$), 65.83

(CH_2), 66.51 (CH_2), 66.55 (CH_2), 103.94 ($\underline{C}HCH(CH_3)C_3H_7$), 104.08 ($\underline{C}HCH(CH_3)C_3H_7$), 122.99 ($C_{pyr}$), 134.84 ($C_{pyr}$), 136.68 ($C_{pyr}$), 146.37 ($C_{pyr}$), 151.05 ($C_{pyr}$), 151.09 ($C_{pyr}$); HRMS-ESI [M-2Cl]$^{2+}$ 413.9107 (calculated for $C_{54}H_{105}N_3O_2$, 413.9098).

5,8-Bis((*N*,*N*-dimethyl-*N*-(octyl)ammonio)methyl)-2-(pentan-3-yl)-4*H*-[1,3]dioxino[4,5-*c*]pyridine dichloride (5k$_8$)

Yield 37%; white solid; mp 179-182 °C (dec.); ^{1}H NMR ($CDCl_3$, 400 MHz) δ 0.81-0.92 (m, 12H, 4CH_3), 1.11-2.06 (m, 29H, 14CH_2+CH), 3.18-3.88 (m, 16H, 4CH_3N^+ + 2CH_2N^+), 4.60, 4.72 (AB, 2H, $^2J_{HH}$ = 12.5 Hz, CH_2), 5.06, 5.57 (AB, 2H, $^2J_{HH}$ = 16.3 Hz, CH_2), 5.13 (br m, 2H, CH_2), 5.36 (d, 1H, $^3J_{HH}$ = 3.9 Hz, CH), 8.60 (s, 1H, CH_{pyr}); ^{13}C NMR ($CDCl_3$, 100 MHz) δ 11.45 (CH_3), 11.62 (CH_3), 14.13 (CH_3), 20.65 (CH_2), 20.80 (CH_2), 22.64 (CH_2), 23.13 (CH_2), 26.39 (CH_2), 26.42 (CH_2), 29.11 (CH_2), 29.13 (CH_2), 29.36 (CH_2), 29.40 (CH_2), 31.73 (CH_2), 44.87 (CH), 49.51 (CH_3N^+), 49.68 (CH_3N^+), 50.87 (CH_3N^+), 51.34 (CH_3N^+), 61.68 (CH_2), 62.17 (CH_2), 65.48 (CH_2), 65.85 (CH_2), 66.38 (CH_2), 103.02 ($\underline{C}HCH(C_2H_5)_2$), 122.81 ($C_{pyr}$), 134.36 ($C_{pyr}$), 137.03 ($C_{pyr}$), 146.67 ($C_{pyr}$), 150.91 ($C_{pyr}$); HRMS-ESI [M-2Cl]$^{2+}$ 273.7535 (calculated for $C_{34}H_{65}N_3O_2$, 273.7533).

5,8-Bis((*N*,*N*-dimethyl-*N*-(decyl)ammonio)methyl)-2-(pentan-3-yl)-4*H*-[1,3]dioxino[4,5-*c*]pyridine dichloride (5k$_{10}$)

Yield 44%; white solid; mp 173-174 °C (dec.); ^{1}H NMR ($CDCl_3$, 400 MHz) δ 0.85 (t, 6H, $^3J_{HH}$ = 6.5 Hz, 2CH_3), 0.92 (t, 6H, $^3J_{HH}$ = 7.3 Hz, 2CH_3), 1.14-2.05 (m, 37H, 18CH_2+CH), 3.20-3.88 (m, 16H, 4CH_3N^+ + 2CH_2N^+), 4.61, 4.72 (AB, 2H, $^2J_{HH}$ = 12.5 Hz, CH_2), 5.07, 5.57 (AB, 2H, $^2J_{HH}$ = 16.3 Hz, CH_2), 5.13 (br m, 2H, CH_2), 5.37 (d, 1H, $^3J_{HH}$ = 4.2 Hz, CH), 8.59 (s, 1H, CH_{pyr}); ^{13}C NMR ($CDCl_3$, 100 MHz) δ 11.48 (CH_3), 11.64 (CH_3), 14.20 (CH_3), 20.67 (CH_2), 20.84 (CH_2), 22.74 (CH_2), 23.17 (CH_2), 26.43 (CH_2), 26.45 (CH_2), 29.34 (CH_2), 29.49 (CH_2), 29.55 (CH_2), 31.92 (CH_2), 44.91 (CH), 49.55 (CH_3N^+), 49.73 (CH_3N^+), 50.88 (CH_3N^+), 51.36 (CH_3N^+), 61.84 (CH_2), 62.24 (CH_2), 65.59 (CH_2), 65.87 (CH_2), 66.42 (CH_2), 103.02 ($\underline{C}HCH(C_2H_5)_2$), 122.70 ($C_{pyr}$), 134.16 ($C_{pyr}$), 137.18 ($C_{pyr}$), 146.81 ($C_{pyr}$), 150.87 ($C_{pyr}$); HRMS-ESI [M-2Cl]$^{2+}$ 301.7848 (calculated for $C_{38}H_{73}N_3O_2$, 301.7846).

5,8-Bis((*N*,*N*-dimethyl-*N*-(dodecyl)ammonio)methyl)-2-(pentan-3-yl)-4*H*-[1,3]dioxino[4,5-*c*]pyridine dichloride (5k$_{12}$)

Yield 40%; white solid; mp 169-170 °C (dec.); ^{1}H NMR ($CDCl_3$, 400 MHz) δ 0.85 (t, 6H, $^3J_{HH}$ = 6.7 Hz, $2CH_3$), 0.92 (t, 6H, $^3J_{HH}$ = 7.4 Hz, $2CH_3$), 1.14-2.05 (m, 45H, $22CH_2$+CH), 3.18-3.89 (m, 16H, $4CH_3N^+$ + $2CH_2N^+$), 4.61, 4.73 (AB, 2H, $^2J_{HH}$ = 12.5 Hz, CH_2), 5.07, 5.58 (AB, 2H, $^2J_{HH}$ = 16.3 Hz, CH_2), 5.14 (br m, 2H, CH_2), 5.38 (d, 1H, $^3J_{HH}$ = 4.2 Hz, CH), 8.60 (s, 1H, CH_{pyr}); ^{13}C NMR ($CDCl_3$, 100 MHz) δ 11.47 (CH_3), 11.65 (CH_3), 14.22 (CH_3), 20.67 (CH_2), 20.84 (CH_2), 22.77 (CH_2), 23.18 (CH_2), 26.44 (CH_2), 26.46 (CH_2), 29.42 (CH_2), 29.47 (CH_2), 29.51 (CH_2), 29.61 (CH_2), 29.69 (CH_2), 31.99 (CH_2), 44.91 (CH), 49.57 (CH_3N^+), 49.73 (CH_3N^+), 50.88 (CH_3N^+), 51.35 (CH_3N^+), 61.80 (CH_2), 62.23 (CH_2), 65.64 (CH_2), 65.87 (CH_2), 66.47 (CH_2), 103.06 (<u>C</u>HCH(C_2H_5)$_2$), 122.79 (C_{pyr}), 134.36 (C_{pyr}), 137.05 (C_{pyr}), 146.69 (C_{pyr}), 150.95 (C_{pyr}); HRMS-ESI [M-2Cl]$^{2+}$ 329.8163 (calculated for $C_{42}H_{81}N_3O_2$, 329.8159).

5,8-Bis((*N*,*N*-dimethyl-*N*-(tetradecyl)ammonio)methyl)-2-(pentan-3-yl)-4*H*-[1,3]dioxino[4,5-*c*]pyridine dichloride (5k$_{14}$)

Yield 48%; white solid; mp 170-173 °C (dec.); ^{1}H NMR ($CDCl_3$, 400 MHz) δ 0.85 (t, 6H, $^3J_{HH}$ = 6.7 Hz, $2CH_3$), 0.92 (t, 6H, $^3J_{HH}$ = 7.3 Hz, $2CH_3$), 1.12-2.05 (m, 53H, $26CH_2$+CH), 3.17-3.91 (m, 16H, $4CH_3N^+$ + $2CH_2N^+$), 4.61, 4.73 (AB, 2H, $^2J_{HH}$ = 12.5 Hz, CH_2), 5.07, 5.57 (AB, 2H, $^2J_{HH}$ = 16.3 Hz, CH_2), 5.12, 5.15 (AB, 2H, $^2J_{HH}$ = 12.0 Hz, CH_2), 5.37 (d, 1H, $^3J_{HH}$ = 4.1 Hz, CH), 8.59 (s, 1H, CH_{pyr}); ^{13}C NMR ($CDCl_3$, 100 MHz) δ 11.49 (CH_3), 11.65 (CH_3), 14.22 (CH_3), 20.68 (CH_2), 20.84 (CH_2), 22.78 (CH_2), 23.18 (CH_2), 26.44 (CH_2), 26.46 (CH_2), 29.45 (CH_2), 29.52 (CH_2), 29.63 (CH_2), 29.74 (CH_2), 32.00 (CH_2), 44.92 (CH), 49.56 (CH_3N^+), 49.73 (CH_3N^+), 50.88 (CH_3N^+), 51.36 (CH_3N^+), 61.88 (CH_2), 62.26 (CH_2), 65.64 (CH_2), 65.87 (CH_2), 66.43 (CH_2), 103.02 (<u>C</u>HCH(C_2H_5)$_2$), 122.70 (C_{pyr}), 134.16 (C_{pyr}), 137.18 (C_{pyr}), 146.82 (C_{pyr}), 150.88 (C_{pyr}); HRMS-ESI [M-2Cl]$^{2+}$ 357.8476 (calculated for $C_{46}H_{89}N_3O_2$, 357.8472).

5,8-Bis((*N*,*N*-dimethyl-*N*-(hexadecyl)ammonio)methyl)-2-(pentan-3-yl)-4*H*-[1,3]dioxino[4,5-*c*]pyridine dichloride (5k$_{16}$)

Yield 45%; white solid; mp 156-157 °C (dec.); ^{1}H NMR ($CDCl_3$, 400 MHz) δ 0.85 (t, 6H, $^3J_{HH}$ = 6.7 Hz, $2CH_3$), 0.93 (t, 6H, $^3J_{HH}$ = 7.2 Hz, $2CH_3$), 1.13-2.07 (m, 61H, $30CH_2$+CH), 3.10-3.93 (m, 16H, $4CH_3N^+$ + $2CH_2N^+$), 4.63, 4.75 (AB, 2H, $^2J_{HH}$ = 12.5 Hz, CH_2), 5.08, 5.60 (AB, 2H, $^2J_{HH}$ = 16.2 Hz, CH_2), 5.15, 5.17 (AB, 2H, $^2J_{HH}$ = 15.6 Hz, CH_2),5.39 (d, 1H, $^3J_{HH}$ = 3.5 Hz, CH), 8.60 (s, 1H, CH_{pyr}); ^{13}C NMR ($CDCl_3$, 100 MHz) δ 11.49 (CH_3), 11.66 (CH_3),

14.24 (CH_3), 20.67 (CH_2), 20.85 (CH_2), 22.80 (CH_2), 23.20 (CH_2), 26.45 (CH_2), 26.47 (CH_2), 29.47 (CH_2), 29.54 (CH_2), 29.65 (CH_2), 29.77 (CH_2), 29.81 (CH_2), 32.03 (CH_2), 44.92 (CH), 49.60 (CH_3N^+), 49.78 (CH_3N^+), 50.93 (CH_3N^+), 51.35 (CH_3N^+), 61.78 (CH_2), 62.21 (CH_2), 65.72 (CH_2), 65.89 (CH_2), 66.55 (CH_2), 103.12 ($\underline{C}HCH(C_2H_5)_2$), 122.89 ($C_{pyr}$), 134.62 ($C_{pyr}$), 136.88 ($C_{pyr}$), 146.54 ($C_{pyr}$), 151.07 ($C_{pyr}$); HRMS-ESI $[M-2Cl]^{2+}$ 385.8786 (calculated for $C_{50}H_{97}N_3O_2$, 385.8785).

5,8-Bis((*N*,*N*-dimethyl-*N*-(octadecyl)ammonio)methyl)-2-(pentan-3-yl)-4*H*-[1,3]dioxino[4,5-*c*]pyridine dichloride (5k$_{18}$)

Yield 59%; white solid; mp 155-158 °C (dec.); ^{1}H NMR ($CDCl_3$, 400 MHz) δ 0.85 (t, 6H, $^3J_{HH}$ = 6.7 Hz, 2CH_3), 0.92 (t, 6H, $^3J_{HH}$ = 7.2 Hz, 2CH_3), 1.13-2.05 (m, 69H, 34CH_2+CH), 3.18-3.98 (m, 16H, 4CH_3N^+ + 2CH_2N^+), 4.62, 4.74 (AB, 2H, $^2J_{HH}$ = 12.3 Hz, CH_2), 5.08, 5.58 (AB, 2H, $^2J_{HH}$ = 16.3 Hz, CH_2), 5.14 (br m, 2H, CH_2), 5.39 (d, 1H, $^3J_{HH}$ = 3.2 Hz, CH), 8.60 (s, 1H, CH_{pyr}); ^{13}C NMR ($CDCl_3$, 100 MHz) δ 11.47 (CH_3), 11.65 (CH_3), 14.22 (CH_3), 20.67 (CH_2), 20.83 (CH_2), 22.78 (CH_2), 23.19 (CH_2), 26.45 (CH_2), 26.47 (CH_2), 29.46 (CH_2), 29.53 (CH_2), 29.64 (CH_2), 29.76 (CH_2), 29.80 (CH_2), 32.01 (CH_2), 44.90 (CH), 49.62 (CH_3N^+), 49.77 (CH_3N^+), 50.89 (CH_3N^+), 51.35 (CH_3N^+), 61.72 (CH_2), 62.20 (CH_2), 65.66 (CH_2), 65.90 (CH_2), 66.55 (CH_2), 103.11 ($\underline{C}HCH(C_2H_5)_2$), 122.92 ($C_{pyr}$), 134.63 ($C_{pyr}$), 136.87 ($C_{pyr}$), 146.53 ($C_{pyr}$), 151.07 ($C_{pyr}$); HRMS-ESI $[M-2Cl]^{2+}$ 413.9108 (calculated for $C_{54}H_{105}N_3O_2$, 413.9098).

5,8-Bis((*N*,*N*-dimethyl-*N*-(octyl)ammonio)methyl)-2-hexyl-4*H*-[1,3]dioxino[4,5-*c*]pyridine dichloride (5l$_8$)

Yield 58%; white solid; mp 186-187 °C (dec.); ^{1}H NMR ($CDCl_3$, 400 MHz) δ 0.84-0.91 (m, 9H, 2$\underline{CH_3}C_7H_{14}$ + $\underline{CH_3}C_5H_{10}$), 1.16-1.48 (m, 28H, 14$CH_2$), 1.70-1.91 (m, 6H, 3$CH_2$), 3.30-3.90 (m, 16H, 4$CH_3N^+$ + 2CH_2N^+), 4.66, 4.73 (AB, 2H, $^2J_{HH}$ = 12.8 Hz, CH_2), 5.08, 5.54 (AB, 2H, $^2J_{HH}$ = 16.1 Hz, CH_2), 5.09, 5.20 (AB, 2H, $^2J_{HH}$ = 13.5 Hz, CH_2), 5.43 (br s, 1H, CH), 8.59 (s, 1H, CH_{pyr}); ^{13}C NMR ($CDCl_3$, 100 MHz) δ 14.21 (CH_3), 22.72 (CH_2), 23.25 (CH_2), 23.78 (CH_2), 26.50 (CH_2), 29.21 (CH_2), 29.26 (CH_2), 29.47 (CH_2), 31.81 (CH_2), 31.86 (CH_2), 34.50 (CH_2), 49.84 (CH_3N^+), 50.03 (CH_3N^+), 51.40 (CH_3N^+), 51.56 (CH_3N^+), 61.36 (CH_2), 62.16 (CH_2), 65.84 (CH_2), 65.96 (CH_2), 66.77 (CH_2), 101.85 ($\underline{C}HC_6H_{13}$), 123.56 (C_{pyr}), 135.80 (C_{pyr}), 136.14 (C_{pyr}), 145.60 (C_{pyr}), 151.50 (C_{pyr}); HRMS-ESI $[M-2Cl]^{2+}$ 280.7620 (calculated for $C_{35}H_{67}N_3O_2$, 280.7611).

5,8-Bis((*N,N*-dimethyl-*N*-(decyl)ammonio)methyl)-2-hexyl-4*H*-[1,3]dioxino[4,5-*c*]pyridine dichloride (5l$_{10}$)

Yield 65%; white solid; mp 184-187 °C (dec.); ^{1}H NMR (CDCl$_3$, 400 MHz) δ 0.84-0.90 (m, 9H, 2$\underline{CH_3}$C$_9$H$_{18}$ + $\underline{CH_3}$C$_5$H$_{10}$), 1.19-1.48 (m, 36H, 18CH$_2$), 1.74-1.86 (m, 6H, 3CH$_2$), 3.31-3.84 (m, 16H, 4CH$_3$N$^+$ + 2CH$_2$N$^+$), 4.70, 4.82 (AB, 2H, $^{2}J_{HH}$ = 12.5 Hz, CH$_2$), 5.09, 5.59 (AB, 2H, $^{2}J_{HH}$ = 16.1 Hz, CH$_2$), 5.15, 5.27 (AB, 2H, $^{2}J_{HH}$ = 13.2 Hz, CH$_2$), 5.47 (br s, 1H, CH), 8.64 (s, 1H, CH$_{pyr}$); ^{13}C NMR (CDCl$_3$, 100 MHz) δ 14.18 (CH$_3$), 22.70 (CH$_2$), 23.21 (CH$_2$), 23.74 (CH$_2$), 26.48 (CH$_2$), 29.18 (CH$_2$), 29.22 (CH$_2$), 29.24 (CH$_2$), 29.45 (CH$_2$), 31.79 (CH$_2$), 31.84 (CH$_2$), 34.48 (CH$_2$), 49.66 (CH$_3$N$^+$), 49.83 (CH$_3$N$^+$), 51.18 (CH$_3$N$^+$), 51.44 (CH$_3$N$^+$), 61.54 (CH$_2$), 62.19 (CH$_2$), 65.76 (CH$_2$), 65.51 (CH$_2$), 101.60 ($\underline{C}$HC$_6$H$_{13}$), 123.21 (C$_{pyr}$), 135.30 (C$_{pyr}$), 136.32 (C$_{pyr}$), 146.10 (C$_{pyr}$), 151.14 (C$_{pyr}$); HRMS-ESI [M-2Cl]$^{2+}$ 308.7929 (calculated for C$_{39}$H$_{75}$N$_3$O$_2$, 308.7924).

5,8-Bis((*N,N*-dimethyl-*N*-(dodecyl)ammonio)methyl)-2-hexyl-4*H*-[1,3]dioxino[4,5-*c*]pyridine dichloride (5l$_{12}$)

Yield 62%; white solid; mp 179-182 °C (dec.); ^{1}H NMR (CDCl$_3$, 400 MHz) δ 0.85-0.92 (m, 9H, 2$\underline{CH_3}$C$_{11}$H$_{22}$ + $\underline{CH_3}$C$_5$H$_{10}$), 1.20-1.48 (m, 44H, 22CH$_2$), 1.73-1.81 (m, 6H, 3CH$_2$), 3.30-3.85 (m, 16H, 4CH$_3$N$^+$ + 2CH$_2$N$^+$), 4.66, 4.72 (AB, 2H, $^{2}J_{HH}$ = 12.2 Hz, CH$_2$), 5.08, 5.53 (AB, 2H, $^{2}J_{HH}$ = 16.0 Hz, CH$_2$), 5.08, 5.19 (AB, 2H, $^{2}J_{HH}$ = 13.7 Hz, CH$_2$), 5.45 (br s, 1H, CH), 8.58 (s, 1H, CH$_{pyr}$); ^{13}C NMR (CDCl$_3$, 100 MHz) δ 14.21 (CH$_3$), 14.25 (CH$_3$), 22.71 (CH$_2$), 22.81 (CH$_2$), 23.24 (CH$_2$), 23.75 (CH$_2$), 26.50 (CH$_2$), 29.25 (CH$_2$), 29.46 (CH$_2$), 29.55 (CH$_2$), 29.60 (CH$_2$), 29.64 (CH$_2$), 29.66 (CH$_2$), 29.74 (CH$_2$), 31.85 (CH$_2$), 32.02 (CH$_2$), 34.49 (CH$_2$), 49.73 (CH$_3$N$^+$), 49.89 (CH$_3$N$^+$), 51.19 (CH$_3$N$^+$), 51.47 (CH$_3$N$^+$), 61.64 (CH$_2$), 62.24 (CH$_2$), 65.73 (CH$_2$), 65.84 (CH$_2$), 66.57 (CH$_2$), 101.61 ($\underline{C}$HC$_6$H$_{13}$), 123.16 (C$_{pyr}$), 135.18 (C$_{pyr}$), 136.36 (C$_{pyr}$), 146.15 (C$_{pyr}$), 151.14 (C$_{pyr}$); HRMS-ESI [M-2Cl]$^{2+}$ 336.8241 (calculated for C$_{43}$H$_{83}$N$_3$O$_2$, 336.8237).

5,8-Bis((*N,N*-dimethyl-*N*-(tetradecyl)ammonio)methyl)-2-hexyl-4*H*-[1,3]dioxino[4,5-*c*]pyridine dichloride (5l$_{14}$)

Yield 57%; white solid; mp 184-186 °C (dec.); ^{1}H NMR (CDCl$_3$, 400 MHz) δ 0.85-0.91 (m, 9H, 2$\underline{CH_3}$C$_{13}$H$_{26}$ + $\underline{CH_3}$C$_5$H$_{10}$), 1.23-1.47 (m, 52H, 26CH$_2$), 1.74-1.84 (m, 6H, 3CH$_2$), 3.31-3.84 (m, 16H, 4CH$_3$N$^+$ + 2CH$_2$N$^+$), 4.71, 4.82 (AB, 2H, $^{2}J_{HH}$ = 12.6 Hz, CH$_2$),

5.09, 5.59 (AB, 2H, $^2J_{HH}$ = 16.2 Hz, CH_2), 5.14, 5.27 (AB, 2H, $^2J_{HH}$ = 13.5 Hz, CH_2), 5.48 (br s, 1H, CH), 8.64 (s, 1H, CH_{pyr}); ^{13}C NMR ($CDCl_3$, 100 MHz) δ 14.20 (CH_3), 14.23 (CH_3), 22.70 (CH_2), 22.78 (CH_2), 23.22 (CH_2), 23.74 (CH_2), 26.49 (CH_2), 29.24 (CH_2), 29.38 (CH_2), 29.52 (CH_2), 29.58 (CH_2), 31.85 (CH_2), 31.96 (CH_2), 34.48 (CH_2), 49.66 (CH_3N^+), 49.82 (CH_3N^+), 51.13 (CH_3N^+), 51.40 (CH_3N^+), 61.48 (CH_2), 62.18 (CH_2), 65.72 (CH_2), 65.79 (CH_2), 66.55 (CH_2), 101.60 ($\underline{C}HC_6H_{13}$), 123.26 (C_{pyr}), 135.44 (C_{pyr}), 136.20 (C_{pyr}), 146.00 (C_{pyr}), 151.20 (C_{pyr}); HRMS-ESI [M-2Cl]$^{2+}$ 364.8554 (calculated for $C_{47}H_{91}N_3O_2$, 364.8550).

5,8-Bis((*N*,*N*-dimethyl-*N*-(hexadecyl)ammonio)methyl)-2-hexyl-4*H*-[1,3]dioxino[4,5-*c*]pyridine dichloride (5l$_{16}$)

Yield 60%; white solid; mp 183-184 °C (dec.); ^1H NMR ($CDCl_3$, 400 MHz) δ 0.84-0.91 (m, 9H, 2$\underline{CH_3}C_{15}H_{30}$ + $\underline{CH_3}C_5H_{10}$), 1.23-1.47 (m, 60H, 30$CH_2$), 1.74-1.84 (m, 6H, 3$CH_2$), 3.17-3.83 (m, 16H, 4$CH_3N^+$ + 2CH_2N^+), 4.70, 4.80 (AB, 2H, $^2J_{HH}$ = 12.5 Hz, CH_2), 5.09, 5.58 (AB, 2H, $^2J_{HH}$ = 16.3 Hz, CH_2), 5.17, 5.25 (AB, 2H, $^2J_{HH}$ = 13.2 Hz, CH_2), 5.47 (br s, 1H, CH), 8.63 (s, 1H, CH_{pyr}); ^{13}C NMR ($CDCl_3$, 100 MHz) δ 14.20 (CH_3), 14.24 (CH_3), 22.70 (CH_2), 22.80 (CH_2), 23.22 (CH_2), 23.74 (CH_2), 26.50 (CH_2), 29.24 (CH_2), 29.45 (CH_2), 29.54 (CH_2), 29.59 (CH_2), 29.63 (CH_2), 29.66 (CH_2), 29.73 (CH_2), 31.85 (CH_2), 32.02 (CH_2), 34.48 (CH_2), 49.65 (CH_3N^+), 49.81 (CH_3N^+), 51.11 (CH_3N^+), 51.41 (CH_3N^+), 61.56 (CH_2), 62.21 (CH_2), 65.71 (CH_2), 65.79 (CH_2), 66.52 (CH_2), 101.56 ($\underline{C}HC_6H_{13}$), 123.17 (C_{pyr}), 135.25 (C_{pyr}), 136.32 (C_{pyr}), 146.11 (C_{pyr}), 151.13 (C_{pyr}); HRMS-ESI [M-2Cl]$^{2+}$ 392.8866 (calculated for $C_{51}H_{99}N_3O_2$, 392.8863).

5,8-Bis((*N*,*N*-dimethyl-*N*-(octadecyl)ammonio)methyl)-2-hexyl-4*H*-[1,3]dioxino[4,5-*c*]pyridine dichloride (5l$_{18}$)

Yield 67%; white solid; mp 187-189 °C (dec.); ^1H NMR ($CDCl_3$, 400 MHz) δ 0.85-0.91 (m, 9H, 2$\underline{CH_3}C_{17}H_{34}$ + $\underline{CH_3}C_5H_{10}$), 1.10-1.52 (m, 68H, 34$CH_2$), 1.71-1.87 (m, 6H, 3$CH_2$), 3.31-3.83 (m, 16H, 4$CH_3N^+$ + 2CH_2N^+), 4.71, 4.78 (AB, 2H, $^2J_{HH}$ = 12.4 Hz, CH_2), 5.12, 5.58 (AB, 2H, $^2J_{HH}$ = 16.0 Hz, CH_2), 5.12, 5.22 (AB, 2H, $^2J_{HH}$ = 13.6 Hz, CH_2), 5.50 (br s, 1H, CH), 8.61 (s, 1H, CH_{pyr}); ^{13}C NMR ($CDCl_3$, 100 MHz) δ 14.23 (CH_3), 14.27 (CH_3), 22.72 (CH_2), 22.83 (CH_2), 23.25 (CH_2), 23.74 (CH_2), 26.51 (CH_2), 29.25 (CH_2), 29.50 (CH_2), 29.58 (CH_2), 29.62 (CH_2), 29.69 (CH_2), 29.80 (CH_2), 29.85 (CH_2), 31.86 (CH_2), 32.06 (CH_2), 34.49 (CH_2), 49.75 (CH_3N^+), 49.93 (CH_3N^+), 51.21 (CH_3N^+), 51.44 (CH_3N^+), 61.66 (CH_2),

62.26 (CH_2), 65.72 (CH_2), 65.92 (CH_2), 66.66 (CH_2), 101.63 ($\underline{C}HC_6H_{13}$), 123.26 (C_{pyr}), 135.46 (C_{pyr}), 136.23 (C_{pyr}), 146.01 (C_{pyr}), 151.27 (C_{pyr}); HRMS-ESI $[M-2Cl]^{2+}$ 420.9176 (calculated for $C_{55}H_{107}N_3O_2$, 420.9176).

5,8-Bis((*N,N*-dimethyl-*N*-(octyl)ammonio)methyl)-2-heptyl-4*H*-[1,3]dioxino[4,5-*c*]pyridine dichloride (5m$_8$)

Yield 65%; white solid; mp 186-187 °C (dec.); ^{1}H NMR ($CDCl_3$, 400 MHz) δ 0.84-0.90 (m, 9H, $2\underline{CH_3}C_7H_{14}$ + $\underline{CH_3}C_6H_{12}$), 1.20-1.46 (m, 30H, $15CH_2$), 1.74-1.89 (m, 6H, $3CH_2$), 3.30-3.87 (m, 16H, $4CH_3N^+$ + $2CH_2N^+$), 4.70, 4.81 (AB, 2H, $^2J_{HH}$ = 12.5 Hz, CH_2), 5.09, 5.58 (AB, 2H, $^2J_{HH}$ = 16.1 Hz, CH_2), 5.13, 5.25 (AB, 2H, $^2J_{HH}$ = 13.4 Hz, CH_2), 5.46 (br s, 1H, CH), 8.63 (s, 1H, CH_{pyr}); ^{13}C NMR ($CDCl_3$, 100 MHz) δ 14.18 (CH_3), 14.23 (CH_3), 22.71 (CH_2), 22.77 (CH_2), 23.21 (CH_2), 23.81 (CH_2), 26.48 (CH_2), 29.18 (CH_2), 29.22 (CH_2), 29.37 (CH_2), 29.46 (CH_2), 29.55 (CH_2), 31.78 (CH_2), 31.80 (CH_2), 31.89 (CH_2), 34.49 (CH_2), 49.66 (CH_3N^+), 49.82 (CH_3N^+), 51.14 (CH_3N^+), 51.43 (CH_3N^+), 61.58 (CH_2), 62.21 (CH_2), 65.74 (CH_2), 66.49 (CH_2), 101.57 ($\underline{C}HC_7H_{15}$), 123.15 (C_{pyr}), 135.17 (C_{pyr}), 136.38 (C_{pyr}), 146.17 (C_{pyr}), 151.09 (C_{pyr}); HRMS-ESI $[M-2Cl]^{2+}$ 287.7691 (calculated for $C_{36}H_{69}N_3O_2$, 287.7689).

5,8-Bis((*N,N*-dimethyl-*N*-(decyl)ammonio)methyl)-2-heptyl-4*H*-[1,3]dioxino[4,5-*c*]pyridine dichloride (5m$_{10}$)

Yield 68%; white solid; mp 180-183 °C (dec.); ^{1}H NMR ($CDCl_3$, 400 MHz) δ 0.84-0.90 (m, 9H, $2\underline{CH_3}C_9H_{18}$ + $\underline{CH_3}C_6H_{12}$), 1.17-1.46 (m, 38H, $19CH_2$), 1.74-1.83 (m, 6H, $3CH_2$), 3.31-3.86 (m, 16H, $4CH_3N^+$ + $2CH_2N^+$), 4.71, 4.82 (AB, 2H, $^2J_{HH}$ = 12.7 Hz, CH_2), 5.08, 5.59 (AB, 2H, $^2J_{HH}$ = 16.3 Hz, CH_2), 5.15, 5.28 (AB, 2H, $^2J_{HH}$ = 13.3 Hz, CH_2), 5.46 (t, 1H, $^3J_{HH}$ = 5.4 Hz, CH), 8.64 (s, 1H, CH_{pyr}); ^{13}C NMR ($CDCl_3$, 100 MHz) δ 14.23 (CH_3), 22.77 (CH_2), 23.22 (CH_2), 23.80 (CH_2), 26.49 (CH_2), 29.38 (CH_2), 29.53 (CH_2), 29.57 (CH_2), 29.60 (CH_2), 31.90 (CH_2), 31.95 (CH_2), 34.48 (CH_2), 49.62 (CH_3N^+), 49.80 (CH_3N^+), 51.14 (CH_3N^+), 51.40 (CH_3N^+), 61.53 (CH_2), 62.18 (CH_2), 65.72 (CH_2), 65.75 (CH_2), 66.49 (CH_2), 101.57 ($\underline{C}HC_7H_{15}$), 123.18 (C_{pyr}), 135.27 (C_{pyr}), 136.30 (C_{pyr}), 146.09 (C_{pyr}), 151.12 (C_{pyr}); HRMS-ESI $[M-2Cl]^{2+}$ 315.8009 (calculated for $C_{40}H_{77}N_3O_2$, 315.8002).

5,8-Bis((*N,N*-dimethyl-*N*-(dodecyl)ammonio)methyl)-2-heptyl-4*H*-[1,3]dioxino[4,5-c]pyridine dichloride (5m$_{12}$)

Yield 78%; white solid; mp 182-184 °C (dec.); ^{1}H NMR ($CDCl_3$, 400 MHz) δ 0.84-0.90 (m, 9H, 2$\underline{CH_3}C_{11}H_{22}$ + $\underline{CH_3}C_6H_{12}$), 1.19-1.48 (m, 46H, 23$CH_2$), 1.73-1.85 (m, 6H, 3$CH_2$), 3.30-3.86 (m, 16H, 4$CH_3N^+$ + 2CH_2N^+), 4.70, 4.81 (AB, 2H, $^{2}J_{HH}$ = 12.5 Hz, CH_2), 5.09, 5.57 (AB, 2H, $^{2}J_{HH}$ = 16.1 Hz, CH_2), 5.13, 5.25 (AB, 2H, $^{2}J_{HH}$ = 13.4 Hz, CH_2), 5.47 (br s, 1H, CH), 8.62 (s, 1H, CH_{pyr}); ^{13}C NMR ($CDCl_3$, 100 MHz) δ 14.24 (CH_3), 22.78 (CH_2), 22.80 (CH_2), 23.22 (CH_2), 23.81 (CH_2), 26.50 (CH_2), 29.38 (CH_2), 29.46 (CH_2), 29.55 (CH_2), 29.60 (CH_2), 29.64 (CH_2), 29.67 (CH_2), 29.73 (CH_2), 31.90 (CH_2), 32.02 (CH_2), 34.49 (CH_2), 49.65 (CH_3N^+), 49.81 (CH_3N^+), 51.11 (CH_3N^+), 51.41 (CH_3N^+), 61.58 (CH_2), 62.21 (CH_2), 65.70 (CH_2), 65.79 (CH_2), 66.52 (CH_2), 101.56 ($\underline{C}HC_7H_{15}$), 123.15 (C_{pyr}), 135.19 (C_{pyr}), 136.35 (C_{pyr}), 146.15 (C_{pyr}), 151.10 (C_{pyr}); HRMS-ESI [M-2Cl]$^{2+}$ 343.8321 (calculated for $C_{44}H_{85}N_3O_2$, 343.8315).

5,8-Bis((*N,N*-dimethyl-*N*-(tetradecyl)ammonio)methyl)-2-heptyl-4*H*-[1,3]dioxino[4,5-*c*]pyridine dichloride (5m$_{14}$)

Yield 67%; white solid; mp 184-185 °C (dec.); ^{1}H NMR ($CDCl_3$, 400 MHz) δ 0.84-0.90 (m, 9H, 2$\underline{CH_3}C_{13}H_{26}$ + $\underline{CH_3}C_6H_{12}$), 1.23-1.46 (m, 54H, 27$CH_2$), 1.73-1.86 (m, 6H, 3$CH_2$), 3.31-3.84 (m, 16H, 4$CH_3N^+$ + 2CH_2N^+), 4.71, 4.83 (AB, 2H, $^{2}J_{HH}$ = 12.6 Hz, CH_2), 5.09, 5.59 (AB, 2H, $^{2}J_{HH}$ = 16.2 Hz, CH_2), 5.15, 5.28 (AB, 2H, $^{2}J_{HH}$ = 12.8 Hz, CH_2), 5.47 (t, 1H, $^{3}J_{HH}$ = 5.3 Hz, CH), 8.64 (s, 1H, CH_{pyr}); ^{13}C NMR ($CDCl_3$, 100 MHz) δ 14.24 (CH_3), 22.78 (CH_2), 22.81 (CH_2), 23.23 (CH_2), 23.81 (CH_2), 26.50 (CH_2), 29.38 (CH_2), 29.48 (CH_2), 29.55 (CH_2), 29.61 (CH_2), 29.65 (CH_2), 29.68 (CH_2), 29.75 (CH_2), 29.78 (CH_2), 29.81 (CH_2), 31.90 (CH_2), 32.04 (CH_2), 34.49 (CH_2), 49.65 (CH_3N^+), 49.83 (CH_3N^+), 51.14 (CH_3N^+), 51.39 (CH_3N^+), 61.49 (CH_2), 62.17 (CH_2), 65.73 (CH_2), 65.84 (CH_2), 66.56 (CH_2), 101.61 ($\underline{C}HC_7H_{15}$), 123.26 (C_{pyr}), 135.47 (C_{pyr}), 136.18 (C_{pyr}), 145.97 (C_{pyr}), 151.21 (C_{pyr}); HRMS-ESI [M-2Cl]$^{2+}$ 371.8634 (calculated for $C_{48}H_{93}N_3O_2$, 371.8628).

5,8-Bis((*N,N*-dimethyl-*N*-(hexadecyl)ammonio)methyl)-2-heptyl-4*H*-[1,3]dioxino[4,5-*c*]pyridine dichloride (5m$_{16}$)

Yield 74%; white solid; mp 185-187 °C (dec.); ^{1}H NMR ($CDCl_3$, 400 MHz) δ 0.85-0.90 (m, 9H, 2$\underline{CH_3}C_{15}H_{30}$ + $\underline{CH_3}C_6H_{12}$), 1.23-1.36 (m, 62H, 31$CH_2$), 1.76-1.82 (m, 6H, 3$CH_2$), 3.31-3.87 (m, 16H, 4$CH_3N^+$ + 2CH_2N^+), 4.73, 4.86 (AB, 2H, $^{2}J_{HH}$ = 12.7 Hz, CH_2), 5.09, 5.61 (AB, 2H, $^{2}J_{HH}$ = 16.3 Hz, CH_2), 5.17, 5.31 (AB, 2H, $^{2}J_{HH}$ = 13.3 Hz, CH_2), 5.48 (t,

1H, $^3J_{HH}$ = 5.4 Hz, CH), 8.66 (s, 1H, CH_{pyr}); ^{13}C NMR ($CDCl_3$, 100 MHz) δ 14.25 (CH_3), 22.71 (CH_2), 22.79 (CH_2), 22.82 (CH_2), 23.24 (CH_2), 23.82 (CH_2), 26.51 (CH_2), 29.38 (CH_2), 29.49 (CH_2), 29.55 (CH_2), 29.62 (CH_2), 29.66 (CH_2), 29.68 (CH_2), 29.77 (CH_2), 29.80 (CH_2), 29.84 (CH_2), 31.91 (CH_2), 32.05 (CH_2), 34.49 (CH_2), 49.67 (CH_3N^+), 49.86 (CH_3N^+), 51.19 (CH_3N^+), 51.39 (CH_3N^+), 61.41 (CH_2), 62.14 (CH_2), 65.76 (CH_2), 65.90 (CH_2), 66.61 (CH_2), 101.66 ($\underline{C}HC_7H_{15}$), 123.36 (C_{pyr}), 135.72 (C_{pyr}), 136.01 (C_{pyr}), 145.74 (C_{pyr}), 151.32 (C_{pyr}); HRMS-ESI [M-2Cl]$^{2+}$ 399.8948 (calculated for $C_{52}H_{101}N_3O_2$, 399.8941).

5,8-Bis((*N,N*-dimethyl-*N*-(octadecyl)ammonio)methyl)-2-heptyl-4*H*-[1,3]dioxino[4,5-*c*]pyridine dichloride (5m$_{18}$)

Yield 78%; white solid; mp 187-188 °C (dec.); ^{1}H NMR ($CDCl_3$, 400 MHz) δ 0.84-0.89 (m, 9H, 2$\underline{CH_3}C_{17}H_{34}$ + $\underline{CH_3}C_6H_{12}$), 1.18-1.46 (m, 70H, 35$CH_2$), 1.73-1.83 (m, 6H, 3$CH_2$), 3.29-3.85 (m, 16H, 4$CH_3N^+$ + 2CH_2N^+), 4.69, 4.81 (AB, 2H, $^2J_{HH}$ = 12.6 Hz, CH_2), 5.08, 5.57 (AB, 2H, $^2J_{HH}$ = 16.2 Hz, CH_2), 5.13, 5.25 (AB, 2H, $^2J_{HH}$ = 13.4 Hz, CH_2), 5.46 (t, 1H, $^3J_{HH}$ = 5.3 Hz, CH), 8.62 (s, 1H, CH_{pyr}); ^{13}C NMR ($CDCl_3$, 100 MHz) δ 14.24 (CH_3), 22.77 (CH_2), 22.80 (CH_2), 23.22 (CH_2), 23.80 (CH_2), 26.50 (CH_2), 29.37 (CH_2), 29.48 (CH_2), 29.55 (CH_2), 29.62 (CH_2), 29.66 (CH_2), 29.69 (CH_2), 29.77 (CH_2), 29.83 (CH_2), 31.90 (CH_2), 32.03 (CH_2), 34.48 (CH_2), 49.62 (CH_3N^+), 49.78 (CH_3N^+), 51.08 (CH_3N^+), 51.39 (CH_3N^+), 61.62 (CH_2), 62.22 (CH_2), 65.69 (CH_2), 65.77 (CH_2), 66.46 (CH_2), 101.51 ($\underline{C}HC_7H_{15}$), 123.07 (C_{pyr}), 135.03 (C_{pyr}), 136.45 (C_{pyr}), 146.24 (C_{pyr}), 151.04 (C_{pyr}); HRMS-ESI [M-2Cl]$^{2+}$ 427.9254 (calculated for $C_{56}H_{109}N_3O_2$, 427.9254).

5,8-Bis((*N,N*-dimethyl-*N*-(octyl)ammonio)methyl)-2-(heptan-3-yl)-4*H*-[1,3]dioxino[4,5-*c*]pyridine dichloride (a mixture of two diastereomers) (5n$_8$)

Yield 64%; white solid; mp 179-180 °C (dec.); ^{1}H NMR ($CDCl_3$, 400 MHz) δ 0.82-0.93 (m, 24H, 8CH_3), 1.15-1.89 (m, 66H, 32CH_2 + 2CH), 3.24-3.42 (m, 24H, 8CH_3N^+), 3.45-3.60 (m, 2H, CH_2N^+), 3.62-3.76 (m, 4H, 2CH_2N^+), 3.77-3.89 (m, 2H, CH_2N^+), 4.63, 4.79 (AB, 2H, $^2J_{HH}$ = 12.7 Hz, CH_2), 4.64, 4.80 (AB, 2H, $^2J_{HH}$ = 12.3 Hz, CH_2), 5.07, 5.62 (AB, 2H, $^2J_{HH}$ = 16.3 Hz, CH_2), 5.07, 5.63 (AB, 2H, $^2J_{HH}$ = 16.3 Hz, CH_2), 5.19, 5.20 (2AB, 4H, $^2J_{HH}$ = 17.6 Hz, 2CH_2), 5.36 (d, 1H, $^3J_{HH}$ = 3.8 Hz, CH), 5.37 (d, 1H, $^3J_{HH}$ = 3.0 Hz, CH), 8.65 (s, 2H, 2CH_{pyr}); ^{13}C NMR ($CDCl_3$, 100 MHz) δ 11.44 (CH_3), 11.68 (CH_3), 14.15 (CH_3), 21.25 (CH_2), 21.32 (CH_2), 22.66 (CH_2), 23.12 (CH_2), 23.17 (CH_2), 23.23 (CH_2), 26.44 (CH_2), 27.58 (CH_2), 27.98

(CH_2), 29.15 (CH_2), 29.25 (CH_2), 29.38 (CH_2), 29.42 (CH_2), 31.75 (CH_2), 43.36 ($CH\underline{C}H(C_2H_5)C_4H_9$), 43.57 ($CH\underline{C}H(C_2H_5)C_4H_9$), 49.58 ($CH_3N^+$), 49.73 ($CH_3N^+$), 50.92 ($CH_3N^+$), 51.36 ($CH_3N^+$), 51.38 ($CH_3N^+$), 61.39 ($CH_2$), 61.46 ($CH_2$), 62.13 ($CH_2$), 65.62 ($CH_2$), 65.93 ($CH_2$), 66.58 ($CH_2$), 103.33 ($\underline{C}HCH(C_2H_5)C_4H_9$), 123.12 ($C_{pyr}$), 135.04 ($C_{pyr}$), 136.57 ($C_{pyr}$), 146.28 ($C_{pyr}$), 151.19 ($C_{pyr}$); HRMS-ESI $[M-2Cl]^{2+}$ 287.7695 (calculated for $C_{36}H_{69}N_3O_2$, 287.7689).

5,8-Bis((*N*,*N*-dimethyl-*N*-(decyl)ammonio)methyl)-2-(heptan-3-yl)-4*H*-[1,3]dioxino[4,5-*c*]pyridine dichloride (a mixture of two diastereomers) (5n$_{10}$)

Yield 71%; white solid; mp 178-180 °C (dec.); ^{1}H NMR ($CDCl_3$, 400 MHz) δ 0.84-0.95 (m, 24H, $8CH_3$), 1.16-1.91 (m, 82H, $40CH_2 + 2CH$), 3.21-3.45 (m, 24H, $8CH_3N^+$), 3.48-3.61 (m, 2H, CH_2N^+), 3.62-3.76 (m, 4H, $2CH_2N^+$), 3.78-3.90 (m, 2H, CH_2N^+), 4.65, 4.83 (AB, 2H, $^2J_{HH}$ = 12.3 Hz, CH_2), 4.66, 4.83 (AB, 2H, $^2J_{HH}$ = 12.2 Hz, CH_2), 5.08, 5.66 (AB, 2H, $^2J_{HH}$ = 16.2 Hz, CH_2), 5.08, 5.67 (AB, 2H, $^2J_{HH}$ = 16.2 Hz, CH_2), 5.21, 5.26 (2AB, 4H, $^2J_{HH}$ = 18.5 Hz, $2CH_2$), 5.38 (d, 1H, $^3J_{HH}$ = 4.2 Hz, CH), 5.40 (d, 1H, $^3J_{HH}$ = 3.8 Hz, CH), 8.67 (s, 2H, $2CH_{pyr}$); ^{13}C NMR ($CDCl_3$, 100 MHz) δ 11.44 (CH_3), 11.70 (CH_3), 14.19 (CH_3), 14.21 (CH_3), 21.24 (CH_2), 21.33 (CH_2), 22.77 (CH_2), 23.15 (CH_2), 23.21 (CH_2), 23.27 (CH_2), 26.48 (CH_2), 27.58 (CH_2), 28.01 (CH_2), 29.26 (CH_2), 29.36 (CH_2), 29.47 (CH_2), 29.52 (CH_2), 29.57 (CH_2), 31.94 (CH_2), 43.38 ($CH\underline{C}H(C_2H_5)C_4H_9$), 43.59 ($CH\underline{C}H(C_2H_5)C_4H_9$), 49.63 ($CH_3N^+$), 49.80 ($CH_3N^+$), 51.00 ($CH_3N^+$), 51.37 ($CH_3N^+$), 51.38 ($CH_3N^+$), 61.28 ($CH_2$), 61.34 ($CH_2$), 62.10 ($CH_2$), 65.81 ($CH_2$), 66.00 ($CH_2$), 66.76 ($CH_2$), 103.47 ($\underline{C}HCH(C_2H_5)C_4H_9$), 123.37 ($C_{pyr}$), 135.64 ($C_{pyr}$), 136.16 ($C_{pyr}$), 145.91 ($C_{pyr}$), 151.46 ($C_{pyr}$); HRMS-ESI $[M-2Cl]^{2+}$ 315.8009 (calculated for $C_{40}H_{77}N_3O_2$, 315.8002).

5,8-Bis((*N*,*N*-dimethyl-*N*-(dodecyl)ammonio)methyl)-2-(heptan-3-yl)-4*H*-[1,3]dioxino[4,5-*c*]pyridine dichloride (a mixture of two diastereomers) (5n$_{12}$)

Yield 62%; white solid; mp 180-181 °C (dec.); ^{1}H NMR ($CDCl_3$, 400 MHz) δ 0.83-0.93 (m, 24H, $8CH_3$), 1.11-1.90 (m, 98H, $48CH_2 + 2CH$), 3.18-3.42 (m, 24H, $8CH_3N^+$), 3.47-3.60 (m, 2H, CH_2N^+), 3.61-3.74 (m, 4H, $2CH_2N^+$), 3.76-3.89 (m, 2H, CH_2N^+), 4.62, 4.78 (AB, 2H, $^2J_{HH}$ = 12.6 Hz, CH_2), 4.63, 4.78 (AB, 2H, $^2J_{HH}$ = 12.1 Hz, CH_2), 5.06, 5.61 (AB, 2H, $^2J_{HH}$ = 16.2 Hz, CH_2), 5.06, 5.62 (AB, 2H, $^2J_{HH}$ = 16.2 Hz, CH_2), 5.17, 5.19 (2AB, 4H, $^2J_{HH}$ = 15.6 Hz, $2CH_2$), 5.35 (d, 1H, $^3J_{HH}$ = 3.6 Hz, CH), 5.37 (d, 1H, $^3J_{HH}$ = 3.5 Hz, CH), 8.64 (s, 2H, $2CH_{pyr}$);

^{13}C NMR ($CDCl_3$, 100 MHz) δ 11.42 (CH_3), 11.66 (CH_3), 14.15 (CH_3), 14.19 (CH_3), 21.23 (CH_2), 21.30 (CH_2), 22.75 (CH_2), 23.11 (CH_2), 23.18 (CH_2), 23.23 (CH_2), 26.46 (CH_2), 27.56 (CH_2), 27.97 (CH_2), 29.23 (CH_2), 29.34 (CH_2), 29.41 (CH_2), 29.46 (CH_2), 29.51 (CH_2), 29.60 (CH_2), 29.68 (CH_2), 31.97 (CH_2), 43.34 (CH<u>C</u>H(C_2H_5)C_4H_9), 43.55 (CH<u>C</u>H(C_2H_5)C_4H_9), 49.57 (CH_3N^+), 49.71 (CH_3N^+), 50.86 (CH_3N^+), 51.33 (CH_3N^+), 51.36 (CH_3N^+), 61.39 (CH_2), 61.46 (CH_2), 62.12 (CH_2), 65.65 (CH_2), 65.91 (CH_2), 66.60 (CH_2), 103.30 (<u>C</u>HCH(C_2H_5)C_4H_9), 123.09 (C_{pyr}), 135.00 (C_{pyr}), 136.57 (C_{pyr}), 146.28 (C_{pyr}), 151.18 (C_{pyr}); HRMS-ESI [M-2Cl]$^{2+}$ 343.8321 (calculated for $C_{44}H_{85}N_3O_2$, 343.8315).

5,8-Bis((*N*,*N*-dimethyl-*N*-(tetradecyl)ammonio)methyl)-2-(heptan-3-yl)-4*H*-[1,3]dioxino[4,5-*c*]pyridine dichloride (a mixture of two diastereomers) (5n$_{14}$)

Yield 67%; white solid; mp 182-183 °C (dec.); ^{1}H NMR ($CDCl_3$, 400 MHz) δ 0.83-0.94 (m, 24H, 8CH_3), 1.10-1.90 (m, 114H, 56CH_2 + 2CH), 3.20-3.92 (m, 32H, 8CH_3N^+ + 4CH_2N^+), 4.63, 4.80 (2AB, 4H, $^2J_{HH}$ = 11.5 Hz, 2CH_2), 5.07, 5.63 (2AB, 4H, $^2J_{HH}$ = 16.1 Hz, 2CH_2), 5.19, 5.21 (2AB, 4H, $^2J_{HH}$ = 17.4 Hz, 2CH_2), 5.37 (br s, 2H, 2CH), 8.65 (s, 2H, 2CH_{pyr}); ^{13}C NMR ($CDCl_3$, 100 MHz) δ 11.44 (CH_3), 11.68 (CH_3), 14.17 (CH_3), 14.21 (CH_3), 21.24 (CH_2), 21.32 (CH_2), 22.77 (CH_2), 23.13 (CH_2), 23.20 (CH_2), 23.24 (CH_2), 26.47 (CH_2), 27.57 (CH_2), 27.99 (CH_2), 29.24 (CH_2), 29.35 (CH_2), 29.45 (CH_2), 29.48 (CH_2), 29.53 (CH_2), 29.63 (CH_2), 29.74 (CH_2), 32.00 (CH_2), 43.36 (CH<u>C</u>H(C_2H_5)C_4H_9), 43.57 (CH<u>C</u>H(C_2H_5)C_4H_9), 49.60 (CH_3N^+), 49.76 (CH_3N^+), 50.92 (CH_3N^+), 51.34 (CH_3N^+), 51.37 (CH_3N^+), 61.39 (CH_2), 61.44 (CH_2), 62.13 (CH_2), 65.72 (CH_2), 65.95 (CH_2), 66.66 (CH_2), 103.37 (<u>C</u>HCH(C_2H_5)C_4H_9), 123.17 (C_{pyr}), 135.19 (C_{pyr}), 136.46 (C_{pyr}), 146.19 (C_{pyr}), 151.26 (C_{pyr}); HRMS-ESI [M-2Cl]$^{2+}$ 371.8626 (calculated for $C_{48}H_{93}N_3O_2$, 371.8628).

5,8-Bis((*N*,*N*-dimethyl-*N*-(hexadecyl)ammonio)methyl)-2-(heptan-3-yl)-4*H*-[1,3]dioxino[4,5-*c*]pyridine dichloride (a mixture of two diastereomers) (5n$_{16}$)

Yield 72%; white solid; mp 182-183 °C (dec.); ^{1}H NMR ($CDCl_3$, 400 MHz) δ 0.83-0.94 (m, 24H, 8CH_3), 1.11-1.89 (m, 130H, 64CH_2 + 2CH), 3.20-3.40 (m, 24H, 8CH_3N^+), 3.41-3.59 (m, 2H, CH_2N^+), 3.60-3.75 (m, 4H, 2CH_2N^+), 3.76-3.90 (m, 2H, CH_2N^+), 4.62, 4.79 (AB, 2H, $^2J_{HH}$ = 12.5 Hz, CH_2), 4.63, 4.79 (AB, 2H, $^2J_{HH}$ = 12.5 Hz, CH_2), 5.07, 5.61 (AB, 2H, $^2J_{HH}$ = 16.2 Hz, CH_2), 5.07, 5.62 (AB, 2H, $^2J_{HH}$ = 16.2 Hz, CH_2), 5.17, 5.18 (2AB, 4H, $^2J_{HH}$ = 16.4 Hz, 2CH_2), 5.34-5.41 (br m, 2H, 2CH), 8.64 (s, 2H, 2CH_{pyr}); ^{13}C NMR ($CDCl_3$, 100 MHz) δ

11.43 (CH_3), 11.67 (CH_3), 14.17 (CH_3), 14.21 (CH_3), 21.23 (CH_2), 21.31 (CH_2), 22.77 (CH_2), 23.13 (CH_2), 23.19 (CH_2), 23.24 (CH_2), 26.47 (CH_2), 27.57 (CH_2), 27.98 (CH_2), 29.24 (CH_2), 29.34 (CH_2), 29.45 (CH_2), 29.48 (CH_2), 29.54 (CH_2), 29.64 (CH_2), 29.75 (CH_2), 29.79 (CH_2), 32.01 (CH_2), 43.36 (CH$\underline{C}$H(C_2H_5)C_4H_9), 43.57 (CH$\underline{C}$H(C_2H_5)C_4H_9), 49.61 (CH_3N^+), 49.73 (CH_3N^+), 50.86 (CH_3N^+), 51.33 (CH_3N^+), 51.36 (CH_3N^+), 61.42 (CH_2), 61.48 (CH_2), 62.14 (CH_2), 65.70 (CH_2), 65.91 (CH_2), 66.65 (CH_2), 103.32 ($\underline{C}$HCH(C_2H_5)C_4H_9), 123.12 (C_{pyr}), 135.07 (C_{pyr}), 136.53 (C_{pyr}), 146.26 (C_{pyr}), 151.22 (C_{pyr}); HRMS-ESI [M-2Cl]$^{2+}$ 399.8946 (calculated for $C_{52}H_{101}N_3O_2$, 399.8941).

5,8-Bis((*N*,*N*-dimethyl-*N*-(octadecyl)ammonio)methyl)-2-(heptan-3-yl)-4*H*-[1,3]dioxino[4,5-*c*]pyridine dichloride (a mixture of two diastereomers) (5n$_{18}$)

Yield 67%; white solid; mp 180-182 °C (dec.); ^{1}H NMR ($CDCl_3$, 400 MHz) δ 0.83-0.93 (m, 24H, 8CH_3), 1.01-1.91 (m, 146H, 72CH_2 + 2CH), 3.20-3.42 (m, 24H, 8CH_3N^+), 3.45-3.59 (m, 2H, CH_2N^+), 3.62-3.75 (m, 4H, 2CH_2N^+), 3.76-3.89 (m, 2H, CH_2N^+), 4.62, 4.78 (AB, 2H, $^2J_{HH}$ = 12.5 Hz, CH_2), 4.63, 4.78 (AB, 2H, $^2J_{HH}$ = 11.9 Hz, CH_2), 5.06, 5.61 (AB, 2H, $^2J_{HH}$ = 16.2 Hz, CH_2), 5.06, 5.62 (AB, 2H, $^2J_{HH}$ = 16.2 Hz, CH_2), 5.18, 5.19 (2AB, 4H, $^2J_{HH}$ = 17.7 Hz, 2CH_2), 5.33-5.38 (br m, 2H, 2CH), 8.64 (s, 2H, 2CH_{pyr}); ^{13}C NMR ($CDCl_3$, 100 MHz) δ 11.44 (CH_3), 11.67 (CH_3), 14.16 (CH_3), 14.21 (CH_3), 21.24 (CH_2), 21.32 (CH_2), 22.77 (CH_2), 23.12 (CH_2), 23.19 (CH_2), 23.24 (CH_2), 26.47 (CH_2), 27.57 (CH_2), 27.98 (CH_2), 29.24 (CH_2), 29.35 (CH_2), 29.44 (CH_2), 29.48 (CH_2), 29.53 (CH_2), 29.64 (CH_2), 29.75 (CH_2), 29.79 (CH_2), 32.00 (CH_2), 43.36 (CH$\underline{C}$H(C_2H_5)C_4H_9), 43.56 (CH$\underline{C}$H(C_2H_5)C_4H_9), 49.58 (CH_3N^+), 49.73 (CH_3N^+), 50.86 (CH_3N^+), 51.33 (CH_3N^+), 51.36 (CH_3N^+), 61.48 (CH_2), 61.53 (CH_2), 62.16 (CH_2), 65.70 (CH_2), 65.91 (CH_2), 66.59 (CH_2), 103.29 ($\underline{C}$HCH(C_2H_5)C_4H_9), 123.02 (C_{pyr}), 134.86 (C_{pyr}), 136.67 (C_{pyr}), 146.39 (C_{pyr}), 151.13 (C_{pyr}); HRMS-ESI [M-2Cl]$^{2+}$ 427.9262 (calculated for $C_{56}H_{109}N_3O_2$, 427.9254).

5,8-Bis((*N*,*N*-dimethyl-*N*-(octyl)ammonio)methyl)-2-octyl-4*H*-[1,3]dioxino[4,5-*c*]pyridine dichloride (5o$_8$)

Yield 53%; white solid; mp 187-188 °C (dec.); ^{1}H NMR ($CDCl_3$, 400 MHz) δ 0.83-0.88 (m, 9H, 2$\underline{CH_3}C_7H_{14}$ + $\underline{CH_3}C_7H_{14}$), 1.16-1.46 (m, 30H, 15$CH_2$), 1.69-1.84 (m, 6H, 3$CH_2$), 2.11-2.61 (m, 2H, CH_2), 3.28-3.83 (m, 16H, 4CH_3N^+ + 2CH_2N^+), 4.63, 4.73 (AB, 2H, $^2J_{HH}$ = 12.9 Hz, CH_2), 5.07, 5.52 (AB, 2H, $^2J_{HH}$ = 16.0 Hz, CH_2), 5.08, 5.16 (AB, 2H, $^2J_{HH}$ = 13.4 Hz,

CH_2), 5.44 (br s, 1H, CH), 8.57 (s, 1H, CH_{pyr}); ^{13}C NMR ($CDCl_3$, 100 MHz) δ 14.18 (CH_3), 14.23 (CH_3), 22.71 (CH_2), 22.80 (CH_2), 23.19 (CH_2), 23.81 (CH_2), 26.47 (CH_2), 29.18 (CH_2), 29.23 (CH_2), 29.39 (CH_2), 29.46 (CH_2), 29.61 (CH_2), 29.68 (CH_2), 31.78 (CH_2), 31.81 (CH_2), 31.98 (CH_2), 34.51 (CH_2), 49.62 (CH_3N^+), 49.76 (CH_3N^+), 51.02 (CH_3N^+), 51.46 (CH_3N^+), 62.02 (CH_2), 62.37 (CH_2), 65.62 (CH_2), 65.72 (CH_2), 66.32 (CH_2), 101.35 ($\underline{C}HC_8H_{17}$), 122.68 (C_{pyr}), 134.04 (C_{pyr}), 137.10 (C_{pyr}), 146.87 (C_{pyr}), 150.68 (C_{pyr}); HRMS-ESI [M-2Cl]$^{2+}$ 294.7774 (calculated for $C_{37}H_{71}N_3O_2$, 294.7768).

5,8-Bis((*N*,*N*-dimethyl-*N*-(decyl)ammonio)methyl)-2-octyl-4*H*-[1,3]dioxino[4,5-*c*]pyridine dichloride (5o$_{10}$)

Yield 63%; white solid; mp 182-183 °C (dec.); ^1H NMR ($CDCl_3$, 400 MHz) δ 0.83-0.90 (m, 9H, 2$\underline{CH_3}C_9H_{18}$ + $\underline{CH_3}C_7H_{14}$), 1.24-1.45 (m, 38H, 19$CH_2$), 1.73-1.84 (m, 6H, 3$CH_2$), 2.15-2.23 (m, 2H, CH_2), 3.29-3.83 (m, 16H, 4CH_3N^+ + 2CH_2N^+), 4.65, 4.72 (AB, 2H, $^2J_{HH}$ = 12.6 Hz, CH_2), 5.08, 5.52 (AB, 2H, $^2J_{HH}$ = 16.2 Hz, CH_2), 5.08, 5.16 (AB, 2H, $^2J_{HH}$ = 13.4 Hz, CH_2), 5.45 (t, 1H, $^3J_{HH}$ = 5.3 Hz, CH), 8.56 (s, 1H, CH_{pyr}); ^{13}C NMR ($CDCl_3$, 100 MHz) δ 14.24 (CH_3), 22.79 (CH_2), 22.81 (CH_2), 23.23 (CH_2), 23.82 (CH_2), 26.50 (CH_2), 29.40 (CH_2), 29.54 (CH_2), 29.59 (CH_2), 29.62 (CH_2), 29.70 (CH_2), 31.97 (CH_2), 32.00 (CH_2), 34.53 (CH_2), 49.68 (CH_3N^+), 49.82 (CH_3N^+), 51.07 (CH_3N^+), 51.47 (CH_3N^+), 62.12 (CH_2), 62.41 (CH_2), 65.64 (CH_2), 65.76 (CH_2), 66.40 (CH_2), 101.38 ($\underline{C}HC_8H_{17}$), 122.68 (C_{pyr}), 134.09 (C_{pyr}), 137.10 (C_{pyr}), 146.86 (C_{pyr}), 150.74 (C_{pyr}); HRMS-ESI [M-2Cl]$^{2+}$ 322.8089 (calculated for $C_{41}H_{79}N_3O_2$, 322.8081).

5,8-Bis((*N*,*N*-dimethyl-*N*-(dodecyl)ammonio)methyl)-2-octyl-4*H*-[1,3]dioxino[4,5-*c*]pyridine dichloride (5o$_{12}$)

Yield 57%; white solid; mp 182-183 °C (dec.); ^1H NMR ($CDCl_3$, 400 MHz) δ 0.84-0.89 (m, 9H, 2$\underline{CH_3}C_{11}H_{22}$ + $\underline{CH_3}C_7H_{14}$), 1.19-1.44 (m, 46H, 23$CH_2$), 1.72-1.85 (m, 6H, 3$CH_2$), 2.34-2.43 (m, 2H, CH_2), 3.28-3.83 (m, 16H, 4CH_3N^+ + 2CH_2N^+), 4.65, 4.72 (AB, 2H, $^2J_{HH}$ = 12.7 Hz, CH_2), 5.07, 5.52 (AB, 2H, $^2J_{HH}$ = 16.2 Hz, CH_2), 5.07, 5.16 (AB, 2H, $^2J_{HH}$ = 13.4 Hz, CH_2), 5.44 (t, 1H, $^3J_{HH}$ = 5.4 Hz, CH), 8.56 (s, 1H, CH_{pyr}); ^{13}C NMR ($CDCl_3$, 100 MHz) δ 14.24 (CH_3), 22.80 (CH_2), 23.22 (CH_2), 23.82 (CH_2), 26.50 (CH_2), 29.40 (CH_2), 29.46 (CH_2), 29.55 (CH_2), 29.61 (CH_2), 29.64 (CH_2), 29.68 (CH_2), 29.73 (CH_2), 31.99 (CH_2), 32.02 (CH_2), 34.52 (CH_2), 49.63 (CH_3N^+), 49.76 (CH_3N^+), 51.01 (CH_3N^+), 51.45 (CH_3N^+), 62.03

(CH_2), 62.38 (CH_2), 65.62 (CH_2), 65.69 (CH_2), 66.35 (CH_2), 101.35 ($\underline{C}HC_8H_{17}$), 122.67 (C_{pyr}), 134.04 (C_{pyr}), 137.09 (C_{pyr}), 146.87 (C_{pyr}), 150.69 (C_{pyr}); HRMS-ESI $[M-2Cl]^{2+}$ 350.8403 (calculated for $C_{45}H_{87}N_3O_2$, 350.8394).

5,8-Bis((*N*,*N*-dimethyl-*N*-(tetradecyl)ammonio)methyl)-2-octyl-4*H*-[1,3]dioxino[4,5-*c*]pyridine dichloride ($5o_{14}$)

Yield 57%; white solid; mp 180-182 °C (dec.); ^1H NMR $(CDCl_3$, 400 MHz) δ 0.85-0.90 (m, 9H, $2\underline{CH_3}C_{13}H_{26}$ + $\underline{CH_3}C_7H_{14}$), 1.24-1.49 (m, 54H, $27CH_2$), 1.74-1.86 (m, 6H, $3CH_2$), 2.04-2.07 (m, 2H, CH_2), 3.29-3.84 (m, 16H, $4CH_3N^+ + 2CH_2N^+$), 4.66, 4.72 (AB, 2H, $^2J_{HH} = 12.7$ Hz, CH_2), 5.08, 5.52 (AB, 2H, $^2J_{HH} = 16.0$ Hz, CH_2), 5.09, 5.17 (AB, 2H, $^2J_{HH} = 13.3$ Hz, CH_2), 5.45 (t, 1H, $^3J_{HH} = 5.3$ Hz, CH), 8.55 (s, 1H, CH_{pyr}); ^{13}C NMR $(CDCl_3$, 100 MHz) δ 14.26 (CH_3), 22.82 (CH_2), 23.24 (CH_2), 23.81 (CH_2), 26.51 (CH_2), 29.41 (CH_2), 29.50 (CH_2), 29.56 (CH_2), 29.62 (CH_2), 29.66 (CH_2), 29.70 (CH_2), 29.79 (CH_2), 29.83 (CH_2), 32.01 (CH_2), 32.05 (CH_2), 34.54 (CH_2), 49.70 (CH_3N^+), 49.86 (CH_3N^+), 51.13 (CH_3N^+), 51.48 (CH_3N^+), 62.20 (CH_2), 62.43 (CH_2), 65.65 (CH_2), 65.84 (CH_2), 66.43 (CH_2), 101.39 ($\underline{C}HC_8H_{17}$), 122.67 (C_{pyr}), 134.11 (C_{pyr}), 137.10 (C_{pyr}), 146.85 (C_{pyr}), 150.78 (C_{pyr}); HRMS-ESI $[M-2Cl]^{2+}$ 378.8712 (calculated for $C_{49}H_{95}N_3O_2$, 378.8707).

5,8-Bis((*N*,*N*-dimethyl-*N*-(hexadecyl)ammonio)methyl)-2-octyl-4*H*-[1,3]dioxino[4,5-*c*]pyridine dichloride ($5o_{16}$)

Yield 67%; white solid; mp 183-184 °C (dec.); ^1H NMR $(CDCl_3$, 400 MHz) δ 0.85-0.90 (m, 9H, $2\underline{CH_3}C_{15}H_{30}$ + $\underline{CH_3}C_7H_{14}$), 1.24-1.47 (m, 62H, $31CH_2$), 1.74-1.80 (m, 6H, $3CH_2$), 1.96 (br s, 2H, CH_2), 3.30-3.84 (m, 16H, $4CH_3N^+ + 2CH_2N^+$), 4.66, 4.71 (AB, 2H, $^2J_{HH} = 12.7$ Hz, CH_2), 5.09, 5.52 (AB, 2H, $^2J_{HH} = 16.1$ Hz, CH_2), 5.09, 5.17 (AB, 2H, $^2J_{HH} = 13.5$ Hz, CH_2), 5.45 (t, 1H, $^3J_{HH} = 5.3$ Hz, CH), 8.55 (s, 1H, CH_{pyr}); ^{13}C NMR $(CDCl_3$, 100 MHz) δ 14.27 (CH_3), 22.83 (CH_2), 23.25 (CH_2), 23.81 (CH_2), 26.51 (CH_2), 29.41 (CH_2), 29.51 (CH_2), 29.56 (CH_2), 29.63 (CH_2), 29.67 (CH_2), 29.70 (CH_2), 29.80 (CH_2), 29.85 (CH_2), 32.02 (CH_2), 32.06 (CH_2), 34.54 (CH_2), 49.72 (CH_3N^+), 49.90 (CH_3N^+), 51.17 (CH_3N^+), 51.50 (CH_3N^+), 62.22 (CH_2), 62.44 (CH_2), 65.66 (CH_2), 65.87 (CH_2), 66.45 (CH_2), 101.41 ($\underline{C}HC_8H_{17}$), 122.67 (C_{pyr}), 134.13 (C_{pyr}), 137.09 (C_{pyr}), 146.84 (C_{pyr}), 150.80 (C_{pyr}); HRMS-ESI $[M-2Cl]^{2+}$ 406.9023 (calculated for $C_{53}H_{103}N_3O_2$, 406.9020).

5,8-Bis((*N,N*-dimethyl-*N*-(octadecyl)ammonio)methyl)-2-octyl-4*H*-[1,3]dioxino[4,5-*c*]pyridine dichloride (5o18)

Yield 51%; white solid; mp 185-187 °C (dec.); ^{1}H NMR ($CDCl_3$, 400 MHz) δ 0.85-0.90 (m, 9H, 2$\underline{CH_3}C_{17}H_{34}$ + $\underline{CH_3}C_7H_{14}$), 1.25-1.47 (m, 70H, 35$CH_2$), 1.76-1.88 (m, 8H, 4$CH_2$), 3.31-3.85 (m, 16H, 4$CH_3N^+$ + 2CH_2N^+), 4.68 (s, 2H, CH_2), 5.10, 5.53 (AB, 2H, $^2J_{HH}$ = 16.1 Hz, CH_2), 5.10, 5.17 (AB, 2H, $^2J_{HH}$ = 13.4 Hz, CH_2), 5.46 (t, 1H, $^3J_{HH}$ = 5.1 Hz, CH), 8.55 (s, 1H, CH_{pyr}); ^{13}C NMR ($CDCl_3$, 100 MHz) δ 14.28 (CH_3), 22.83 (CH_2), 23.24 (CH_2), 23.80 (CH_2), 26.50 (CH_2), 29.41 (CH_2), 29.51 (CH_2), 29.56 (CH_2), 29.62 (CH_2), 29.67 (CH_2), 29.70 (CH_2), 29.77 (CH_2), 29.81 (CH_2), 29.86 (CH_2), 32.02 (CH_2), 32.06 (CH_2), 34.53 (CH_2), 49.71 (CH_3N^+), 49.92 (CH_3N^+), 51.22 (CH_3N^+), 51.50 (CH_3N^+), 62.26 (CH_2), 62.44 (CH_2), 65.67 (CH_2), 65.93 (CH_2), 66.44 (CH_2), 101.42 ($\underline{C}HC_8H_{17}$), 122.66 (C_{pyr}), 134.14 (C_{pyr}), 137.07 (C_{pyr}), 146.82 (C_{pyr}), 150.81 (C_{pyr}); HRMS-ESI [M-2Cl]$^{2+}$ 434.9342 (calculated for $C_{57}H_{111}N_3O_2$, 434.9333).

5,8-Bis((*N,N*-dimethyl-*N*-(octyl)ammonio)methyl)-2-(undecan-2-yl)-4*H*-[1,3]dioxino[4,5-*c*]pyridine dichloride (a mixture of two diastereomers) (5p8)

Yield 39%; white solid; mp 168-170 °C (dec.); ^{1}H NMR ($CDCl_3$, 400 MHz) δ 0.85-0.90 (m, 18H, 2CH_3 + 4$\underline{CH_3}C_7H_{14}$), 1.01 (d, 3H, $^3J_{HH}$ = 8.5 Hz, CH_3), 1.03 (d, 3H, $^3J_{HH}$ = 8.2 Hz, CH_3), 1.27-1.42 (m, 64H, 32CH_2), 1.58-1.79 (m, 18H, 8CH_2 +2CH), 3.32-3.87 (m, 32H, 8CH_3N^+ + 4CH_2N^+), 4.65 (br s, 4H, 2CH_2), 5.03-5.29 (m, 8H, 4CH_2 + 2CH), 5.59-5.65 (m, 2H, 2CH_2), 8.51 (s, 2H, 2CH); ^{13}C NMR ($CDCl_3$, 100 MHz) δ 13.59 (CH_3), 13.86 (CH_3), 14.19 (CH_3), 14.25 (CH_3), 22.71 (CH_2), 22.81 (CH_2), 23.20 (CH_2), 26.47 (CH_2), 27.05 (CH_2), 27.11 (CH_2), 29.18 (CH_2), 29.21 (CH_2), 29.45 (CH_2), 29.49 (CH_2), 29.79 (CH_2), 29.81 (CH_2), 30.06 (CH_2), 30.15 (CH_2), 31.17 (CH_2), 31.80 (CH_2), 32.03 (CH_2), 37.44 ($CH\underline{C}H(CH_3)C_9H_{19}$), 37.45 ($CH\underline{C}H(CH_3)C_9H_{19}$), 49.68 ($CH_3N^+$), 49.83 ($CH_3N^+$), 50.85 ($CH_3N^+$), 50.94 ($CH_3N^+$), 51.44 ($CH_3N^+$), 62.02 ($CH_2$), 62.38 ($CH_2$), 65.77 ($CH_2$), 65.81 ($CH_2$), 66.55 ($CH_2$), 103.94 ($\underline{C}HCH(CH_3)C_9H_{19}$), 104.10 ($\underline{C}HCH(CH_3)C_9H_{19}$), 122.69 ($C_{pyr}$), 134.17 ($C_{pyr}$), 137.17 ($C_{pyr}$), 137.18 ($C_{pyr}$), 146.81 ($C_{pyr}$), 150.88 ($C_{pyr}$); HRMS-ESI [M-2Cl]$^{2+}$ 315.8008 (calculated for $C_{40}H_{77}N_3O_2$, 315.8002).

5,8-Bis((*N,N*-dimethyl-*N*-(decyl)ammonio)methyl)-2-(undecan-2-yl)-4*H*-[1,3]dioxino[4,5-*c*]pyridine dichloride (a mixture of two diastereomers) (5p10)

Yield 60%; white solid; mp 172-173 °C (dec.); ^{1}H NMR ($CDCl_3$, 400 MHz) δ 0.86-0.89 (m, 18H, $2CH_3$ + $4\underline{CH_3}C_9H_{18}$), 1.00-1.06 (m, 6H, $2CH_3$), 1.25-1.42 (m, 80H, $40CH_2$), 1.54-1.95 (m, 18H, $8CH_2$ +2CH), 3.33-3.87 (m, 32H, $8CH_3N^+$ + $4CH_2N^+$), 4.64 (br s, 4H, $2CH_2$), 5.00-5.35 (m, 8H, $4CH_2$ + 2CH), 5.59-5.69 (m, 2H, $2CH_2$), 8.50 (s, 2H, 2CH); ^{13}C NMR ($CDCl_3$, 100 MHz) δ 13.58 (CH_3), 13.83 (CH_3), 14.21 (CH_3), 22.76 (CH_2), 22.79 (CH_2), 23.20 (CH_2), 26.48 (CH_2), 27.04 (CH_2), 27.10 (CH_2), 29.37 (CH_2), 29.49 (CH_2), 29.52 (CH_2), 29.58 (CH_2), 29.79 (CH_2), 30.05 (CH_2), 30.14 (CH_2), 31.16 (CH_2), 31.94 (CH_2), 32.01 (CH_2), 37.43 ($CH\underline{C}H(CH_3)C_9H_{19}$), 37.45 ($CH\underline{C}H(CH_3)C_9H_{19}$), 49.64 ($CH_3N^+$), 49.78 ($CH_3N^+$), 50.78 ($CH_3N^+$), 50.87 ($CH_3N^+$), 51.41 ($CH_3N^+$), 61.94 ($CH_2$), 62.35 ($CH_2$), 65.68 ($CH_2$), 65.80 ($CH_2$), 66.53 ($CH_2$), 103.92 ($\underline{C}HCH(CH_3)C_9H_{19}$), 104.07 ($\underline{C}HCH(CH_3)C_9H_{19}$), 122.70 ($C_{pyr}$), 134.13 ($C_{pyr}$), 137.17 ($C_{pyr}$), 146.84 ($C_{pyr}$), 150.86 ($C_{pyr}$); HRMS-ESI [M-2Cl]$^{2+}$ 343.8321 (calculated for $C_{44}H_{85}N_3O_2$, 343.8315).

5,8-Bis((*N*,*N*-dimethyl-*N*-(dodecyl)ammonio)methyl)-2-(undecan-2-yl)-4*H*-[1,3]dioxino[4,5-*c*]pyridine dichloride (a mixture of two diastereomers) (5p$_{12}$)

Yield 58%; white solid; mp 172-173 °C (dec.); ^{1}H NMR ($CDCl_3$, 400 MHz) δ 0.86-0.90 (m, 18H, $2CH_3$ + $4\underline{CH_3}C_{11}H_{22}$), 1.00-1.06 (m, 6H, $2CH_3$), 1.17-1.42 (m, 96H, $48CH_2$), 1.53-1.88 (m, 18H, $8CH_2$ +2CH), 3.33-3.88 (m, 32H, $8CH_3N^+$ + $4CH_2N^+$), 4.64 (br s, 4H, $2CH_2$), 5.04-5.30 (m, 8H, $4CH_2$ + 2CH), 5.60-5.65 (m, 2H, $2CH_2$), 8.48 (s, 2H, 2CH); ^{13}C NMR ($CDCl_3$, 100 MHz) δ 13.57 (CH_3), 13.81 (CH_3), 14.21 (CH_3), 22.77 (CH_2), 23.18 (CH_2), 26.46 (CH_2), 27.03 (CH_2), 27.08 (CH_2), 29.43 (CH_2), 29.49 (CH_2), 29.53 (CH_2), 29.57 (CH_2), 29.63 (CH_2), 29.70 (CH_2), 29.77 (CH_2), 29.80 (CH_2), 30.05 (CH_2), 30.14 (CH_2), 31.14 (CH_2), 31.99 (CH_2), 37.40 ($CH\underline{C}H(CH_3)C_9H_{19}$), 37.42 ($CH\underline{C}H(CH_3)C_9H_{19}$), 49.62 ($CH_3N^+$), 49.71 ($CH_3N^+$), 50.67 ($CH_3N^+$), 50.76 ($CH_3N^+$), 51.38 ($CH_3N^+$), 61.85 ($CH_2$), 62.30 ($CH_2$), 65.61 ($CH_2$), 65.75 ($CH_2$), 66.49 ($CH_2$), 103.88 ($\underline{C}HCH(CH_3)C_9H_{19}$), 104.02 ($\underline{C}HCH(CH_3)C_9H_{19}$), 122.69 ($C_{pyr}$), 134.09 ($C_{pyr}$), 137.13 ($C_{pyr}$), 137.15 ($C_{pyr}$), 146.81 ($C_{pyr}$), 150.80 ($C_{pyr}$), 150.83 ($C_{pyr}$); HRMS-ESI [M-2Cl]$^{2+}$ 371.8634 (calculated for $C_{48}H_{93}N_3O_2$, 371.8628).

5,8-Bis((*N*,*N*-dimethyl-*N*-(tetradecyl)ammonio)methyl)-2-(undecan-2-yl)-4*H*-[1,3]dioxino[4,5-*c*]pyridine dichloride (a mixture of two diastereomers) (5p$_{14}$)

Yield 49%; white solid; mp 172-174 °C (dec.); ^{1}H NMR ($CDCl_3$, 400 MHz) δ 0.86-0.90 (m, 18H, $2CH_3$ + $4\underline{CH_3}C_{13}H_{26}$), 1.01-1.05 (m, 6H, $2CH_3$), 1.25-1.45 (m, 112H, $56CH_2$),

1.51-1.88 (m, 18H, 8CH_2 +2CH), 3.23-3.92 (m, 32H, 8CH_3N^+ + 4CH_2N^+), 4.63 (br m, 4H, 2CH_2), 5.00-5.29 (m, 8H, 4CH_2 + 2CH), 5.60-5.66 (m, 2H, 2CH_2), 8.48 (s, 2H, 2CH); ^{13}C NMR ($CDCl_3$, 100 MHz) δ 13.57 (CH_3), 13.81 (CH_3), 14.21 (CH_3), 22.77 (CH_2), 23.18 (CH_2), 26.46 (CH_2), 27.02 (CH_2), 27.08 (CH_2), 29.45 (CH_2), 29.54 (CH_2), 29.58 (CH_2), 29.64 (CH_2), 29.75 (CH_2), 29.77 (CH_2), 30.05 (CH_2), 30.14 (CH_2), 31.14 (CH_2), 32.00 (CH_2), 37.39 ($CH\underline{C}H(CH_3)C_9H_{19}$), 37.42 ($CH\underline{C}H(CH_3)C_9H_{19}$), 49.62 ($CH_3N^+$), 49.69 ($CH_3N^+$), 50.65 ($CH_3N^+$), 50.75 ($CH_3N^+$), 51.37 ($CH_3N^+$), 61.85 ($CH_2$), 62.30 ($CH_2$), 65.60 ($CH_2$), 65.75 ($CH_2$), 66.49 ($CH_2$), 103.88 ($\underline{C}HCH(CH_3)C_9H_{19}$), 104.02 ($\underline{C}HCH(CH_3)C_9H_{19}$), 122.69 ($C_{pyr}$), 134.09 ($C_{pyr}$), 137.12 ($C_{pyr}$), 137.15 ($C_{pyr}$), 146.82 ($C_{pyr}$), 150.79 ($C_{pyr}$), 150.83 ($C_{pyr}$); HRMS-ESI $[M-2Cl]^{2+}$ 399.8947 (calculated for $C_{52}H_{101}N_3O_2$, 399.8941).

5,8-Bis((*N,N*-dimethyl-*N*-(hexadecyl)ammonio)methyl)-2-(undecan-2-yl)-4*H*-[1,3]dioxino[4,5-*c*]pyridine dichloride (a mixture of two diastereomers) (5p$_{16}$)

Yield 56%; white solid; mp 168-170 °C (dec.); ^{1}H NMR ($CDCl_3$, 400 MHz) δ 0.86-0.90 (m, 18H, 2CH_3 + 4$\underline{CH_3}C_{15}H_{30}$), 1.00-1.04 (m, 6H, 2$CH_3$), 1.16-1.45 (m, 128H, 64CH_2), 1.53-1.93 (m, 18H, 8CH_2 +2CH), 3.32-3.90 (m, 32H, 8CH_3N^+ + 4CH_2N^+), 4.64 (br s, 4H, 2CH_2), 5.04-5.29 (m, 8H, 4CH_2 + 2CH), 5.59-5.65 (m, 2H, 2CH_2), 8.49 (s, 2H, 2CH); ^{13}C NMR ($CDCl_3$, 100 MHz) δ 13.57 (CH_3), 13.81 (CH_3), 14.22 (CH_3), 22.78 (CH_2), 23.19 (CH_2), 26.47 (CH_2), 27.03 (CH_2), 27.08 (CH_2), 29.46 (CH_2), 29.54 (CH_2), 29.59 (CH_2), 29.66 (CH_2), 29.76 (CH_2), 29.78 (CH_2), 29.80 (CH_2), 30.05 (CH_2), 30.14 (CH_2), 31.14 (CH_2), 32.01 (CH_2), 37.40 ($CH\underline{C}H(CH_3)C_9H_{19}$), 37.43 ($CH\underline{C}H(CH_3)C_9H_{19}$), 49.62 ($CH_3N^+$), 49.72 ($CH_3N^+$), 50.66 ($CH_3N^+$), 50.76 ($CH_3N^+$), 51.37 ($CH_3N^+$), 61.86 ($CH_2$), 62.30 ($CH_2$), 65.64 ($CH_2$), 65.75 ($CH_2$), 66.50 ($CH_2$), 66.52 ($CH_2$), 103.88 ($\underline{C}HCH(CH_3)C_9H_{19}$), 104.02 ($\underline{C}HCH(CH_3)C_9H_{19}$), 122.69 ($C_{pyr}$), 134.09 ($C_{pyr}$), 137.13 ($C_{pyr}$), 137.15 ($C_{pyr}$), 146.81 ($C_{pyr}$), 146.82 ($C_{pyr}$), 150.80 ($C_{pyr}$), 150.83 ($C_{pyr}$); HRMS-ESI $[M-2Cl]^{2+}$ 427.9259 (calculated for $C_{56}H_{109}N_3O_2$, 427.9254).

5,8-Bis((*N,N*-dimethyl-*N*-(octadecyl)ammonio)methyl)-2-(undecan-2-yl)-4*H*-[1,3]dioxino[4,5-*c*]pyridine dichloride (a mixture of two diastereomers) (5p$_{18}$)

Yield 52%; white solid; mp 166-169 °C (dec.); ^{1}H NMR ($CDCl_3$, 400 MHz) δ 0.86-0.90 (m, 18H, 2CH_3 + 4$\underline{CH_3}C_{17}H_{34}$), 1.00-1.05 (m, 6H, 2$CH_3$), 1.18-1.45 (m, 144H, 72CH_2), 1.52-1.95 (m, 18H, 8CH_2 +2CH), 3.32-3.86 (m, 32H, 8CH_3N^+ + 4CH_2N^+), 4.63 (br m, 4H, 2CH_2), 5.04-5.30 (m, 8H, 4CH_2 + 2CH), 5.60-5.65 (m, 2H, 2CH_2), 8.48 (s, 2H, 2CH); ^{13}C

NMR ($CDCl_3$, 100 MHz) δ 13.58 (CH_3), 13.83 (CH_3), 14.22 (CH_3), 22.79 (CH_2), 23.20 (CH_2), 26.48 (CH_2), 27.04 (CH_2), 27.09 (CH_2), 29.46 (CH_2), 29.49 (CH_2), 29.55 (CH_2), 29.60 (CH_2), 29.67 (CH_2), 29.76 (CH_2), 29.82 (CH_2), 30.06 (CH_2), 30.15 (CH_2), 31.16 (CH_2), 32.02 (CH_2), 37.41 ($CH\underline{C}H(CH_3)C_9H_{19}$), 37.44 ($CH\underline{C}H(CH_3)C_9H_{19}$), 49.65 ($CH_3N^+$), 49.75 ($CH_3N^+$), 50.69 ($CH_3N^+$), 50.79 ($CH_3N^+$), 51.39 ($CH_3N^+$), 61.92 ($CH_2$), 62.23 ($CH_2$), 65.68 ($CH_2$), 65.76 ($CH_2$), 66.53 ($CH_2$), 103.90 ($\underline{C}HCH(CH_3)C_9H_{19}$), 104.04 ($\underline{C}HCH(CH_3)C_9H_{19}$), 122.69 ($C_{pyr}$), 134.11 ($C_{pyr}$), 137.14 ($C_{pyr}$), 137.17 ($C_{pyr}$), 146.83 ($C_{pyr}$), 150.83 ($C_{pyr}$), 150.86 ($C_{pyr}$); HRMS-ESI $[M\text{-}2Cl]^{2+}$ 455.9571 (calculated for $C_{60}H_{117}N_3O_2$, 455.9567).

5,8-Bis((*N,N*-dimethyl-*N*-(octyl)ammonio)methyl)-2-methyl-2-octyl-4*H*-[1,3]dioxino[4,5-*c*]pyridine dichloride (5q8)

Yield 29%; white solid; mp 156-160 °C (dec.); ^1H NMR ($CDCl_3$, 400 MHz) δ 0.82-0.87 (m, 9H, $3\underline{CH_3}C_7H_{14}$), 1.18-1.44 (m, 32H, $16CH_2$), 1.56 (s, 3H, CH_3), 1.71-1.93 (m, 6H, $3CH_2$), 3.31-3.38 (m, 12H, $4CH_3N^+$), 3.52-3.79 (m, 4H, $2CH_2N^+$), 4.67, 4.74 (AB, 2H, $^2J_{HH}$ = 12.6 Hz, CH_2), 5.08, 5.32 (AB, 2H, $^2J_{HH}$ = 16.8 Hz, CH_2), 5.18, 5.22 (AB, 2H, $^2J_{HH}$ = 14.7 Hz, CH_2), 8.59 (s, 1H, CH_{pyr}); ^{13}C NMR ($CDCl_3$, 100 MHz) δ 14.17 (CH_3), 14.21 (CH_3), 22.03 (CH_2), 22.69 (CH_3), 22.77 (CH_2), 23.15 (CH_2), 23.50 (CH_2), 26.46 (CH_2), 29.19 (CH_2), 29.40 (CH_2), 29.44 (CH_2), 29.74 (CH_2), 29.85 (CH_2), 31.79 (CH_2), 31.97 (CH_2), 39.57 (CH_2), 49.55 (CH_3N^+), 49.69 (CH_3N^+), 50.91 (CH_3N^+), 51.26 (CH_3N^+), 59.36 (CH_2), 61.79 (CH_2), 62.31 (CH_2), 65.33 (CH_2), 66.26 (CH_2), 104.01 ($\underline{C}(CH_3)C_8H_{17}$), 123.09 ($C_{pyr}$), 133.18 ($C_{pyr}$), 137.02 ($C_{pyr}$), 145.66 ($C_{pyr}$), 149.68 ($C_{pyr}$); HRMS-ESI $[M\text{-}2Cl]^{2+}$ 301.7851 (calculated for $C_{38}H_{73}N_3O_2$, 301.7846).

5,8-Bis((*N,N*-dimethyl-*N*-(decyl)ammonio)methyl)-2-methyl-2-octyl-4*H*-[1,3]dioxino[4,5-*c*]pyridine dichloride (5q10)

Yield 40%; white solid; mp 164-165 °C (dec.); ^1H NMR ($CDCl_3$, 400 MHz) δ 0.83-0.88 (m, 9H, $2\underline{CH_3}C_9H_{18}$ + $\underline{CH_3}C_7H_{14}$), 1.18-1.46 (m, 40H, $20CH_2$), 1.56 (s, 3H, CH_3), 1.71-1.90 (m, 6H, $3CH_2$), 3.16-3.85 (m, 16H, $4CH_3N^+$ + $2CH_2N^+$), 4.67, 4.74 (AB, 2H, $^2J_{HH}$ = 12.7 Hz, CH_2), 5.08, 5.32 (AB, 2H, $^2J_{HH}$ = 16.9 Hz, CH_2), 5.18, 5.22 (AB, 2H, $^2J_{HH}$ = 14.8 Hz, CH_2), 8.59 (s, 1H, CH_{pyr}); ^{13}C NMR ($CDCl_3$, 100 MHz) δ 14.22 (CH_3), 22.05 (CH_2), 22.77 (CH_3), 23.15 (CH_2), 23.51 (CH_2), 26.47 (CH_2), 29.37 (CH_2), 29.41 (CH_2), 29.51 (CH_2), 29.55 (CH_2), 29.59 (CH_2), 29.75 (CH_2), 29.85 (CH_2), 31.94 (CH_2), 31.98 (CH_2), 39.57 (CH_2), 49.56

(CH_3N^+), 49.70 (CH_3N^+), 50.91 (CH_3N^+), 51.26 (CH_3N^+), 59.36 (CH_2), 61.81 (CH_2), 62.32 (CH_2), 65.36 (CH_2), 66.28 (CH_2), 104.02 ($\underline{C}(CH_3)C_8H_{17}$), 123.09 ($C_{pyr}$), 133.19 ($C_{pyr}$), 137.00 ($C_{pyr}$), 145.65 ($C_{pyr}$), 149.69 ($C_{pyr}$); HRMS-ESI $[M-2Cl]^{2+}$ 329.8164 (calculated for $C_{42}H_{81}N_3O_2$, 329.8159).

5,8-Bis((*N*,*N*-dimethyl-*N*-(dodecyl)ammonio)methyl)-2-methyl-2-octyl-4*H*-[1,3]dioxino[4,5-*c*]pyridine dichloride (5q$_{12}$)

Yield 46%; white solid; mp 153-157 °C (dec.); ^{1}H NMR ($CDCl_3$, 400 MHz) δ 0.84-0.88 (m, 9H, $2\underline{CH_3}C_{11}H_{22}$ + $\underline{CH_3}C_7H_{14}$), 1.19-1.42 (m, 48H, $24CH_2$), 1.57 (s, 3H, CH_3), 1.72-1.94 (m, 6H, $3CH_2$), 3.33-3.39 (m, 12H, $4CH_3N^+$), 3.51-3.80 (m, 4H, $2CH_2N^+$), 4.70, 4.77 (AB, 2H, $^2J_{HH}$ = 12.6 Hz, CH_2), 5.10, 5.34 (AB, 2H, $^2J_{HH}$ = 17.0 Hz, CH_2), 5.21, 5.26 (AB, 2H, $^2J_{HH}$ = 13.4 Hz, CH_2), 8.60 (s, 1H, CH_{pyr}); ^{13}C NMR ($CDCl_3$, 100 MHz) δ 14.24 (CH_3), 22.11 (CH_2), 22.80 (CH_3), 23.17 (CH_2), 23.52 (CH_2), 26.48 (CH_2), 29.42 (CH_2), 29.46 (CH_2), 29.53 (CH_2), 29.57 (CH_2), 29.65 (CH_2), 29.73 (CH_2), 29.77 (CH_2), 29.86 (CH_2), 31.99 (CH_2), 32.02 (CH_2), 39.56 (CH_2), 49.56 (CH_3N^+), 49.71 (CH_3N^+), 50.93 (CH_3N^+), 51.25 (CH_3N^+), 59.36 (CH_2), 61.70 (CH_2), 62.28 (CH_2), 65.43 (CH_2), 66.34 (CH_2), 104.17 ($\underline{C}(CH_3)C_8H_{17}$), 123.29 ($C_{pyr}$), 133.63 ($C_{pyr}$), 136.72 ($C_{pyr}$), 145.36 ($C_{pyr}$), 149.88 ($C_{pyr}$); HRMS-ESI $[M-2Cl]^{2+}$ 357.8477 (calculated for $C_{46}H_{89}N_3O_2$, 357.8472).

5,8-Bis((*N*,*N*-dimethyl-*N*-(tetradecyl)ammonio)methyl)-2-methyl-2-octyl-4*H*-[1,3]dioxino[4,5-c]pyridine dichloride (5q$_{14}$)

Yield 59%; white solid; mp 162-163 °C (dec.); ^{1}H NMR ($CDCl_3$, 400 MHz) δ 0.83-0.88 (m, 9H, $2\underline{CH_3}C_{13}H_{26}$ + $\underline{CH_3}C_7H_{14}$), 1.13-1.44 (m, 56H, $28CH_2$), 1.56 (s, 3H, CH_3), 1.69-1.91 (m, 6H, $3CH_2$), 3.30-3.39 (m, 12H, $4CH_3N^+$), 3.52-3.78 (m, 4H, $2CH_2N^+$), 4.68, 4.75 (AB, 2H, $^2J_{HH}$ = 12.6 Hz, CH_2), 5.08, 5.32 (AB, 2H, $^2J_{HH}$ = 16.9 Hz, CH_2), 5.21, 5.25 (AB, 2H, $^2J_{HH}$ = 13.4 Hz, CH_2), 8.60 (s, 1H, CH_{pyr}); ^{13}C NMR ($CDCl_3$, 100 MHz) δ 14.23 (CH_3), 22.07 (CH_2), 22.78 (CH_3), 23.16 (CH_2), 23.49 (CH_2), 26.47 (CH_2), 29.40 (CH_2), 29.46 (CH_2), 29.52 (CH_2), 29.56 (CH_2), 29.65 (CH_2), 29.73 (CH_2), 29.76 (CH_2), 29.79 (CH_2), 29.85 (CH_2), 31.98 (CH_2), 32.01 (CH_2), 39.55 (CH_2), 49.51 (CH_3N^+), 49.66 (CH_3N^+), 50.93 (CH_3N^+), 51.25 (CH_3N^+), 59.36 (CH_2), 61.86 (CH_2), 62.31 (CH_2), 65.36 (CH_2), 66.22 (CH_2), 103.99 ($\underline{C}(CH_3)C_8H_{17}$), 123.02 ($C_{pyr}$), 133.09 ($C_{pyr}$), 137.08 ($C_{pyr}$), 145.73 ($C_{pyr}$), 149.67 ($C_{pyr}$); HRMS-ESI $[M-2Cl]^{2+}$ 385.8790 (calculated for $C_{50}H_{97}N_3O_2$, 385.8785).

5,8-Bis((*N,N*-dimethyl-*N*-(hexadecyl)ammonio)methyl)-2-methyl-2-octyl-4*H*-[1,3]dioxino[4,5-*c*]pyridine dichloride (5q$_{16}$)

Yield 63%; white solid; mp 162-163 °C (dec.); ^{1}H NMR ($CDCl_3$, 400 MHz) δ 0.83-0.87 (m, 9H, 2$\underline{CH_3}C_{15}H_{30}$ + $\underline{CH_3}C_7H_{14}$), 1.20-1.41 (m, 64H, 32$CH_2$), 1.55 (s, 3H, CH_3), 1.70-1.90 (m, 6H, 3CH_2), 3.31-3.37 (m, 12H, 4CH_3N^+), 3.51-3.81 (m, 4H, 2CH_2N^+), 4.66, 4.73 (AB, 2H, $^2J_{HH}$ = 12.6 Hz, CH_2), 5.07, 5.30 (AB, 2H, $^2J_{HH}$ = 16.8 Hz, CH_2), 5.17, 5.21 (AB, 2H, $^2J_{HH}$ = 14.3 Hz, CH_2), 8.58 (s, 1H, CH_{pyr}); ^{13}C NMR ($CDCl_3$, 100 MHz) δ 14.22 (CH_3), 22.02 (CH_2), 22.77 (CH_3), 23.15 (CH_2), 23.50 (CH_2), 26.47 (CH_2), 29.40 (CH_2), 29.45 (CH_2), 29.53 (CH_2), 29.57 (CH_2), 29.66 (CH_2), 29.75 (CH_2), 29.80 (CH_2), 29.84 (CH_2), 31.97 (CH_2), 32.01 (CH_2), 39.55 (CH_2), 49.52 (CH_3N^+), 49.65 (CH_3N^+), 50.87 (CH_3N^+), 51.22 (CH_3N^+), 59.33 (CH_2), 61.94 (CH_2), 62.34 (CH_2), 65.31 (CH_2), 66.19 (CH_2), 103.88 ($\underline{C}(CH_3)C_8H_{17}$), 122.90 ($C_{pyr}$), 132.80 ($C_{pyr}$), 137.26 ($C_{pyr}$), 145.90 ($C_{pyr}$), 149.53 ($C_{pyr}$); HRMS-ESI [M-2Cl]$^{2+}$ 413.9101 (calculated for $C_{54}H_{105}N_3O_2$, 413.9098).

5,8-Bis((*N,N*-dimethyl-*N*-(octadecyl)ammonio)methyl)-2-methyl-2-octyl-4*H*-[1,3]dioxino[4,5-*c*]pyridine dichloride (5q$_{18}$)

Yield 67%; white solid; mp 162-164 °C (dec.); ^{1}H NMR ($CDCl_3$, 400 MHz) δ 0.83-0.87 (m, 9H, 2$\underline{CH_3}C_{17}H_{34}$ + $\underline{CH_3}C_7H_{14}$), 1.21-1.41 (m, 72H, 36$CH_2$), 1.55 (s, 3H, CH_3), 1.70-1.91 (m, 6H, 3CH_2), 3.31-3.41 (m, 12H, 4CH_3N^+), 3.48-3.82 (m, 4H, 2CH_2N^+), 4.66, 4.72 (AB, 2H, $^2J_{HH}$ = 12.7 Hz, CH_2), 5.06, 5.30 (AB, 2H, $^2J_{HH}$ = 16.8 Hz, CH_2), 5.17, 5.21 (AB, 2H, $^2J_{HH}$ = 13.4 Hz, CH_2), 8.57 (s, 1H, CH_{pyr}); ^{13}C NMR ($CDCl_3$, 100 MHz) δ 14.22 (CH_3), 22.02 (CH_2), 22.78 (CH_3), 23.15 (CH_2), 23.49 (CH_2), 26.47 (CH_2), 29.40 (CH_2), 29.46 (CH_2), 29.53 (CH_2), 29.57 (CH_2), 29.66 (CH_2), 29.75 (CH_2), 29.81 (CH_2), 31.97 (CH_2), 32.01 (CH_2), 39.55 (CH_2), 49.51 (CH_3N^+), 49.65 (CH_3N^+), 50.89 (CH_3N^+), 51.24 (CH_3N^+), 59.33 (CH_2), 62.03 (CH_2), 62.37 (CH_2), 65.33 (CH_2), 66.15 (CH_2), 103.83 ($\underline{C}(CH_3)C_8H_{17}$), 122.79 ($C_{pyr}$), 132.61 ($C_{pyr}$), 137.39 ($C_{pyr}$), 146.03 ($C_{pyr}$), 149.47 ($C_{pyr}$); HRMS-ESI [M-2Cl]$^{2+}$ 441.9416 (calculated for $C_{58}H_{113}N_3O_2$, 441.9411).

5′,8′-Bis((*N,N*-dimethyl-*N*-(octyl)ammonio)methyl)-4'*H*-spiro[cyclohexane-1,2'-[1,3]dioxino[4,5-*c*]pyridine] dichloride (5r$_8$)

Yield 47%; white solid; mp 161-163 °C (dec.); ^{1}H NMR ($CDCl_3$, 400 MHz) δ 0.82-0.87 (m, 6H, 2$\underline{CH_3}C_7H_{14}$), 1.21-1.42 (m, 20H, 10$CH_2$), 1.50-1.85 (m, 12H, 6$CH_2$), 1.97-2.00

(m, 2H, CH_2), 3.34-3.77 (m, 16H, $4CH_3N^+ + 2CH_2N^+$), 4.75 (s, 2H, CH_2), 5.18 (s, 2H, CH_2), 5.24 (s, 2H, CH_2), 8.58 (s, 1H, CH_{pyr}); ^{13}C NMR ($CDCl_3$, 100 MHz) δ 14.17 (CH_3), 22.44 (CH_2), 22.68 (CH_2), 23.09 (CH_2), 23.15 (CH_2), 24.97 (CH_2), 26.43 (CH_2), 29.17 (CH_2), 29.40 (CH_2), 31.76 (CH_2), 34.10 (CH_2), 49.58 (CH_3N^+), 51.14 (CH_3N^+), 58.64 (CH_2), 61.59 (CH_2), 62.15 (CH_2), 65.33 (CH_2), 66.23 (CH_2), 103.01 (C_{spiro}), 123.09 (C_{pyr}), 133.53 (C_{pyr}), 136.91 (C_{pyr}), 145.44 (C_{pyr}), 149.68 (C_{pyr}); HRMS-ESI $[M\text{-}2Cl]^{2+}$ 272.7460 (calculated for $C_{34}H_{63}N_3O_2$, 272.7455).

5′,8′-Bis((*N,N*-dimethyl-*N*-(decyl)ammonio)methyl)-4'*H*-spiro[cyclohexane-1,2'-[1,3]dioxino[4,5-*c*]pyridine] dichloride (5r$_{10}$)

Yield 51%; white solid; mp 181-182 °C (dec.); ^1H NMR ($CDCl_3$, 400 MHz) δ 0.82-0.87 (m, 6H, $2\underline{CH_3}C_9H_{18}$), 1.15-1.41 (m, 28H, $14CH_2$), 1.53-1.82 (m, 12H, $6CH_2$), 1.97-2.01 (m, 2H, CH_2), 3.34-3.77 (m, 16H, $4CH_3N^+ + 2CH_2N^+$), 4.75 (s, 2H, CH_2), 5.18 (s, 2H, CH_2), 5.24 (s, 2H, CH_2), 8.58 (s, 1H, CH_{pyr}); ^{13}C NMR ($CDCl_3$, 100 MHz) δ 14.21 (CH_3), 22.44 (CH_2), 22.75 (CH_2), 23.10 (CH_2), 23.16 (CH_2), 24.97 (CH_2), 26.44 (CH_2), 29.35 (CH_2), 29.47 (CH_2), 29.52 (CH_2), 29.55 (CH_2), 31.92 (CH_2), 34.10 (CH_2), 49.58 (CH_3N^+), 51.12 (CH_3N^+), 58.63 (CH_2), 61.61 (CH_2), 62.16 (CH_2), 65.35 (CH_2), 66.24 (CH_2), 103.01 (C_{spiro}), 123.07 (C_{pyr}), 133.52 (C_{pyr}), 136.90 (C_{pyr}), 145.44 (C_{pyr}), 149.68 (C_{pyr}); HRMS-ESI $[M\text{-}2Cl]^{2+}$ 300.7767 (calculated for $C_{38}H_{71}N_3O_2$, 300.7768).

5′,8′-Bis((*N,N*-dimethyl-*N*-(dodecyl)ammonio)methyl)-4'*H*-spiro[cyclohexane-1,2'-[1,3]dioxino[4,5-*c*]pyridine] dichloride (5r$_{12}$)

Yield 53%; white solid; mp 177-179 °C (dec.); ^1H NMR ($CDCl_3$, 400 MHz) δ 0.85 (t, 6H, $^3J_{HH}$ = 6.7 Hz, $2\underline{CH_3}C_{11}H_{22}$), 1.14-1.42 (m, 36H, $18CH_2$), 1.53-1.82 (m, 12H, $6CH_2$), 1.98-2.02 (m, 2H, CH_2), 3.34-3.76 (m, 16H, $4CH_3N^+ + 2CH_2N^+$), 4.74 (s, 2H, CH_2), 5.18 (s, 2H, CH_2), 5.23 (s, 2H, CH_2), 8.57 (s, 1H, CH_{pyr}); ^{13}C NMR ($CDCl_3$, 100 MHz) δ 14.22 (CH_3), 22.44 (CH_2), 22.77 (CH_2), 23.16 (CH_2), 24.98 (CH_2), 26.45 (CH_2), 29.42 (CH_2), 29.48 (CH_2), 29.54 (CH_2), 29.61 (CH_2), 29.70 (CH_2), 31.99 (CH_2), 34.11 (CH_2), 49.60 (CH_3N^+), 51.13 (CH_3N^+), 58.63 (CH_2), 61.65 (CH_2), 62.18 (CH_2), 65.37 (CH_2), 66.27 (CH_2), 102.99 (C_{spiro}), 123.05 (C_{pyr}), 133.46 (C_{pyr}), 136.94 (C_{pyr}), 145.49 (C_{pyr}), 149.67 (C_{pyr}); HRMS-ESI $[M\text{-}2Cl]^{2+}$ 328.8089 (calculated for $C_{42}H_{79}N_3O_2$, 328.8081).

5′,8′-Bis((*N,N*-dimethyl-*N*-(tetradecyl)ammonio)methyl)-4'*H*-spiro[cyclohexane-1,2'-[1,3]dioxino[4,5-*c*]pyridine] dichloride (5r14)

Yield 60%; white solid; mp 177-178 °C (dec.); ^{1}H NMR ($CDCl_3$, 400 MHz) δ 0.84 (t, 6H, $^3J_{HH}$ = 6.7 Hz, 2$\underline{CH_3}C_{13}H_{26}$), 1.15-1.40 (m, 44H, 22$CH_2$), 1.55-1.80 (m, 12H, 6$CH_2$), 1.96-1.99 (m, 2H, CH_2), 3.33-3.75 (m, 16H, 4CH_3N^+ + 2CH_2N^+), 4.72 (s, 2H, CH_2), 5.16 (s, 2H, CH_2), 5.22 (s, 2H, CH_2), 8.57 (s, 1H, CH_{pyr}); ^{13}C NMR ($CDCl_3$, 100 MHz) δ 14.20 (CH_3), 22.42 (CH_2), 22.75 (CH_2), 23.13 (CH_2), 24.96 (CH_2), 26.44 (CH_2), 29.43 (CH_2), 29.47 (CH_2), 29.53 (CH_2), 29.61 (CH_2), 29.70 (CH_2), 29.72 (CH_2), 29.75 (CH_2), 31.98 (CH_2), 34.08 (CH_2), 49.56 (CH_3N^+), 51.12 (CH_3N^+), 58.60 (CH_2), 61.70 (CH_2), 62.18 (CH_2), 65.30 (CH_2), 66.19 (CH_2), 102.88 (C_{spiro}), 122.92 (C_{pyr}), 133.19 (C_{pyr}), 137.11 (C_{pyr}), 145.66 (C_{pyr}), 149.53 (C_{pyr}); HRMS-ESI [M-2Cl]$^{2+}$ 356.8397 (calculated for $C_{46}H_{87}N_3O_2$, 356.8394).

5′,8′-Bis((*N,N*-dimethyl-*N*-(hexadecyl)ammonio)methyl)-4'*H*-spiro[cyclohexane-1,2'-[1,3]dioxino[4,5-*c*]pyridine] dichloride (5r16)

Yield 64%; white solid; mp 178-180 °C (dec.); ^{1}H NMR ($CDCl_3$, 400 MHz) δ 0.85 (t, 6H, $^3J_{HH}$ = 6.7 Hz, 2$\underline{CH_3}C_{15}H_{30}$), 1.14-1.40 (m, 52H, 26$CH_2$), 1.48-1.82 (m, 12H, 6$CH_2$), 1.98-2.02 (m, 2H, CH_2), 3.34-3.77 (m, 16H, 4CH_3N^+ + 2CH_2N^+), 4.75 (s, 2H, CH_2), 5.18 (s, 2H, CH_2), 5.24 (s, 2H, CH_2), 8.58 (s, 1H, CH_{pyr}); ^{13}C NMR ($CDCl_3$, 100 MHz) δ 14.23 (CH_3), 22.44 (CH_2), 22.79 (CH_2), 23.17 (CH_2), 24.98 (CH_2), 26.46 (CH_2), 29.46 (CH_2), 29.50 (CH_2), 29.56 (CH_2), 29.64 (CH_2), 29.73 (CH_2), 29.76 (CH_2), 29.80 (CH_2), 32.01 (CH_2), 34.13 (CH_2), 49.60 (CH_3N^+), 51.12 (CH_3N^+), 58.63 (CH_2), 61.63 (CH_2), 62.17 (CH_2), 65.42 (CH_2), 66.29 (CH_2), 103.04 (C_{spiro}), 123.10 (C_{pyr}), 133.58 (C_{pyr}), 136.85 (C_{pyr}), 145.40 (C_{pyr}), 149.73 (C_{pyr}); HRMS-ESI [M-2Cl]$^{2+}$ 384.8713 (calculated for $C_{50}H_{95}N_3O_2$, 384.8707).

5′,8′-Bis((*N,N*-dimethyl-*N*-(octadecyl)ammonio)methyl)-4'*H*-spiro[cyclohexane-1,2'-[1,3]dioxino[4,5-*c*]pyridine] dichloride (5r18)

Yield 52%; white solid; mp 174-176 °C (dec.); ^{1}H NMR ($CDCl_3$, 400 MHz) δ 0.85 (t, 6H, $^3J_{HH}$ = 6.6 Hz, 2$\underline{CH_3}C_{17}H_{34}$), 1.12-1.41 (m, 60H, 30$CH_2$), 1.48-1.87 (m, 12H, 6$CH_2$), 1.99-2.02 (m, 2H, CH_2), 3.34-3.77 (m, 16H, 4CH_3N^+ + 2CH_2N^+), 4.76 (s, 2H, CH_2), 5.19 (s, 2H, CH_2), 5.25 (s, 2H, CH_2), 8.58 (s, 1H, CH_{pyr}); ^{13}C NMR ($CDCl_3$, 100 MHz) δ 14.24 (CH_3), 22.45 (CH_2), 22.79 (CH_2), 23.17 (CH_2), 24.99 (CH_2), 26.47 (CH_2), 29.47 (CH_2), 29.50 (CH_2), 29.56 (CH_2), 29.65 (CH_2), 29.74 (CH_2), 29.77 (CH_2), 29.81 (CH_2), 32.02 (CH_2), 34.15 (CH_2),

49.62 (CH_3N^+), 51.15 (CH_3N^+), 58.64 (CH_2), 61.65 (CH_2), 62.18 (CH_2), 65.46 (CH_2), 66.30 (CH_2), 103.06 (C_{spiro}), 123.11 (C_{pyr}), 133.62 (C_{pyr}), 136.83 (C_{pyr}), 145.39 (C_{pyr}), 149.75 (C_{pyr}); HRMS-ESI $[M-2Cl]^{2+}$ 412.9026 (calculated for $C_{54}H_{103}N_3O_2$, 412.9020).

References

[1] Gottlieb, H.E.; Kotlyar, V.; Nudelman, A. *J. Org. Chem.* **1997**, *62*, 7512–7515.

[2] Fulmer, G.R.; Miller, A.J.M.; Sherden, N.H.; Gottlieb, H.E.; Nudelman, A.; Stoltz, B. M.; Bercaw, J.E.; Goldberg, K.I. *Organometallics* **2010**, *29*, 2176–2179.

[3] Sapozhnikov, S.V.; Shtyrlin, N.V.; Kayumov, A.R.; Zamaldinova, A.E.; Iksanova, A.G.; Nikitina, E.V.; Krylova, E.S.; Grishaev, D.Yu.; Balakin, K.V.; Shtyrlin, Yu.G. *Med. Chem. Res.* **2017**, *26*, 3188-3202.

[4] Arustamova, I.S.; Kulnevich, V.G. *Russ. J. Org. Chem* **1985**, *21*, 2433-2438.

[5] Procédé de préparation d'un dérivé sulfuré de la vitamine B6. Patent BE659401 (A) from 08.09.1965.

[6] Grigor'ev, A.A.; Shtyrlin, N.V.; Gabbasova, R.R.; Zeldi, M.I.; Grishaev, D. Yu.; Gnezdilov, O.I.; Balakin, K.V; Nasakin, O.E.; Shtyrlin, Y.G. *Syn. Comm.* **2018**, *48*, 2288-2304.

[7] Pavelyev, R.S.; Bondar, O.V.; Nguyen, T.N.T.; Ziganshina, A.A.; Al Farroukh, M.; Karwt, R.; Alekbaeva, G.D.; Pugachev, M.V.; Yamaleeva, Z.R.; Kataeva, O.N.; Balakin, K.V.; Shtyrlin, Y.G. *Bioorg. Med. Chem.* **2018**, *26*, 5824-5837.

[8] Shtyrlin, Y.G.; Shtyrlin, N.V.; Sapozhnikov, S.V.; Pavelyev, R.S; Method of producing 5,8-(bis(methylene(N,N-dimethyl-N-dodecylammonium))-2-ethyl-4H-[1,3]dioxino[4,5-c]pyridinium dichloride. Patent RU №2697848 C1 from 21.08.2019, priority date 06.06.2019.

NMR Spectra

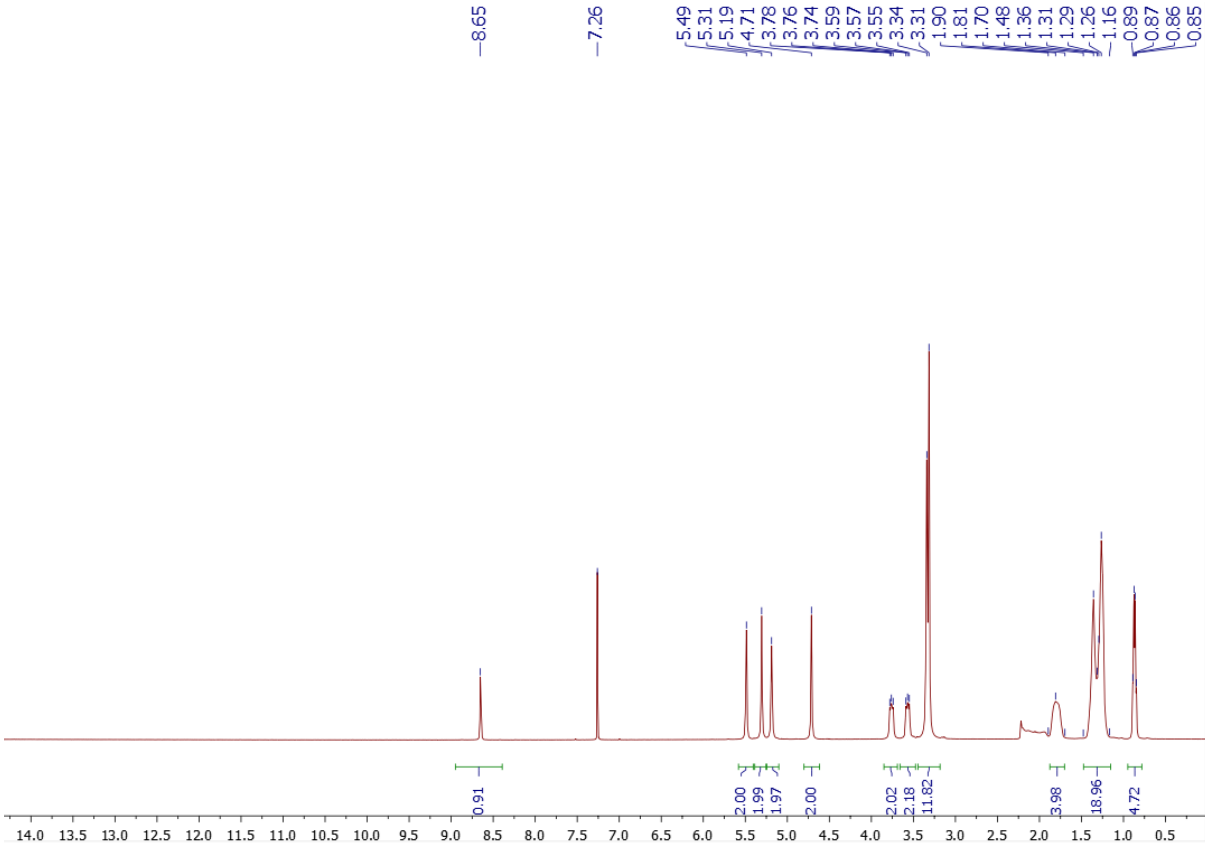

^{1}H NMR spectrum of compound **5a$_8$**

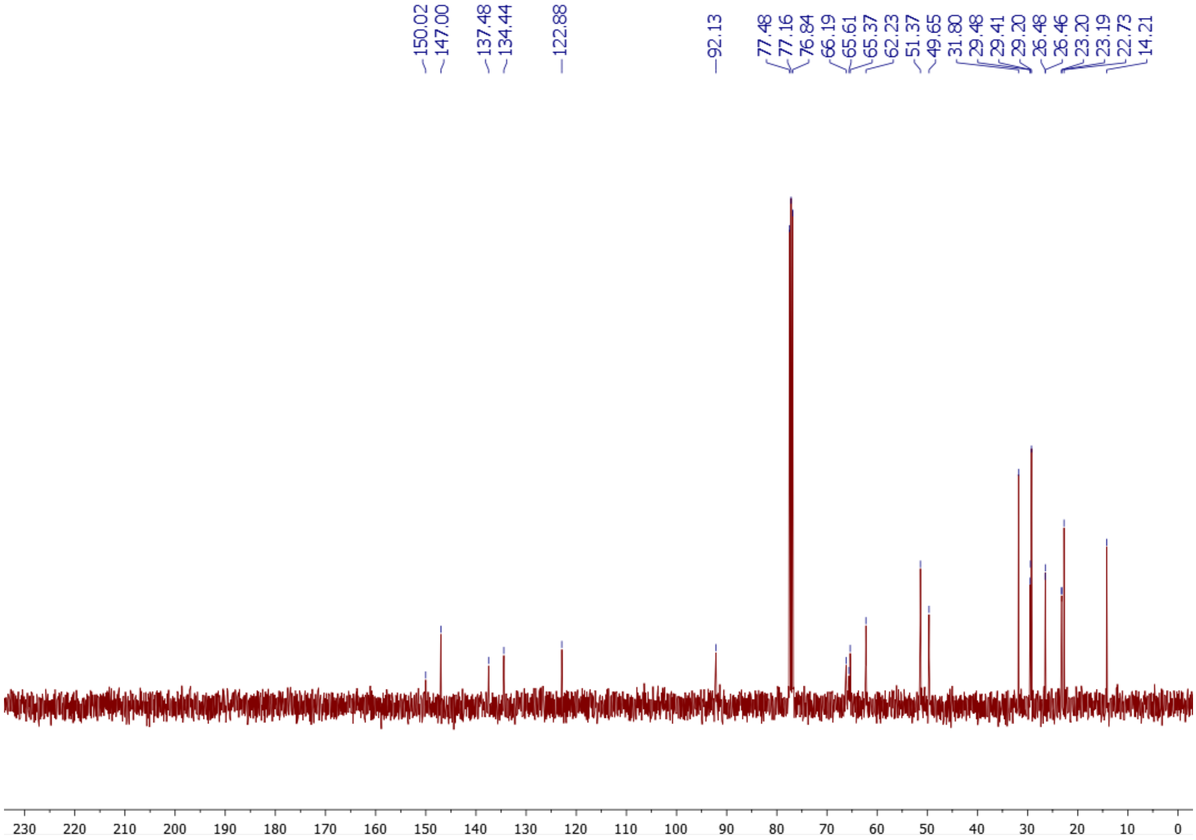

^{13}C{H} NMR spectrum of compound **5a$_8$**

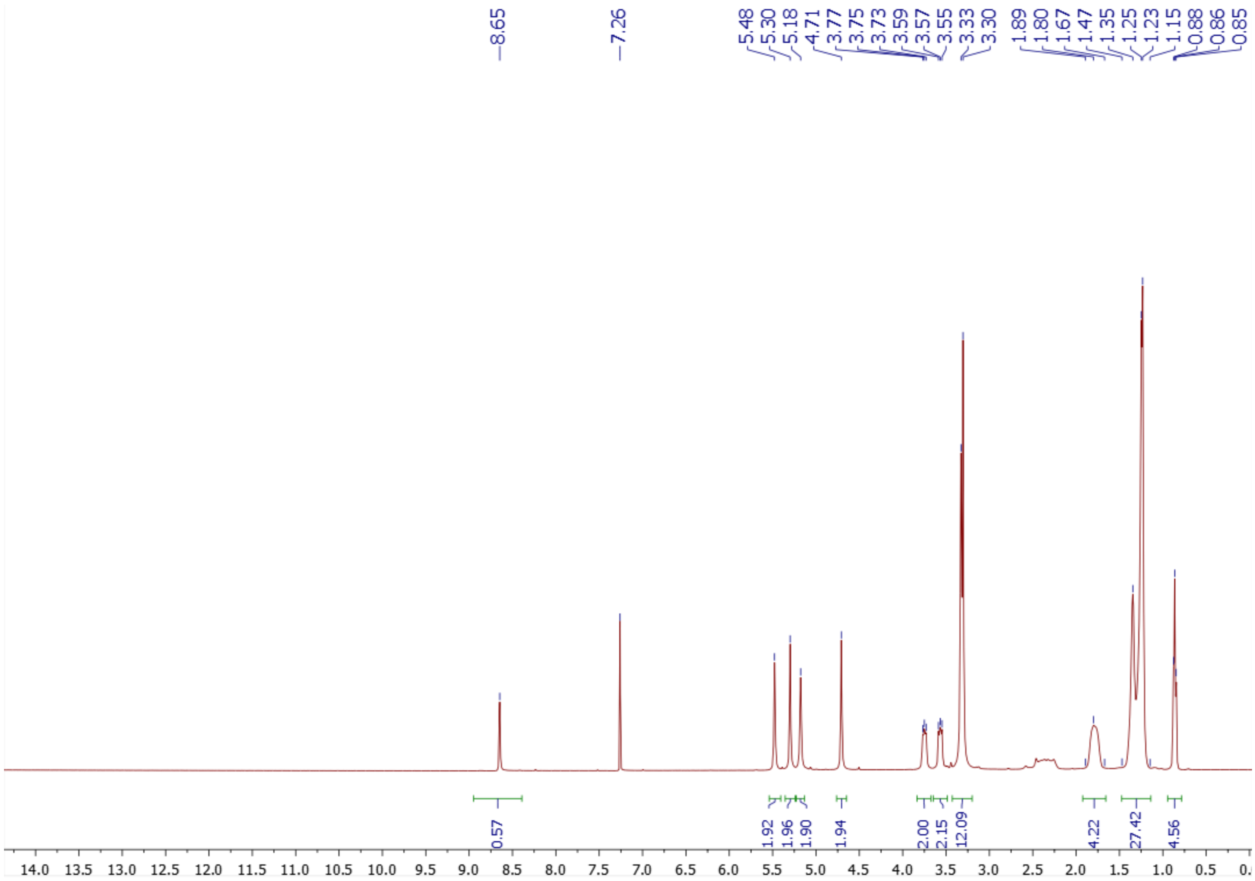

^{1}H NMR spectrum of compound **5a**$_{10}$

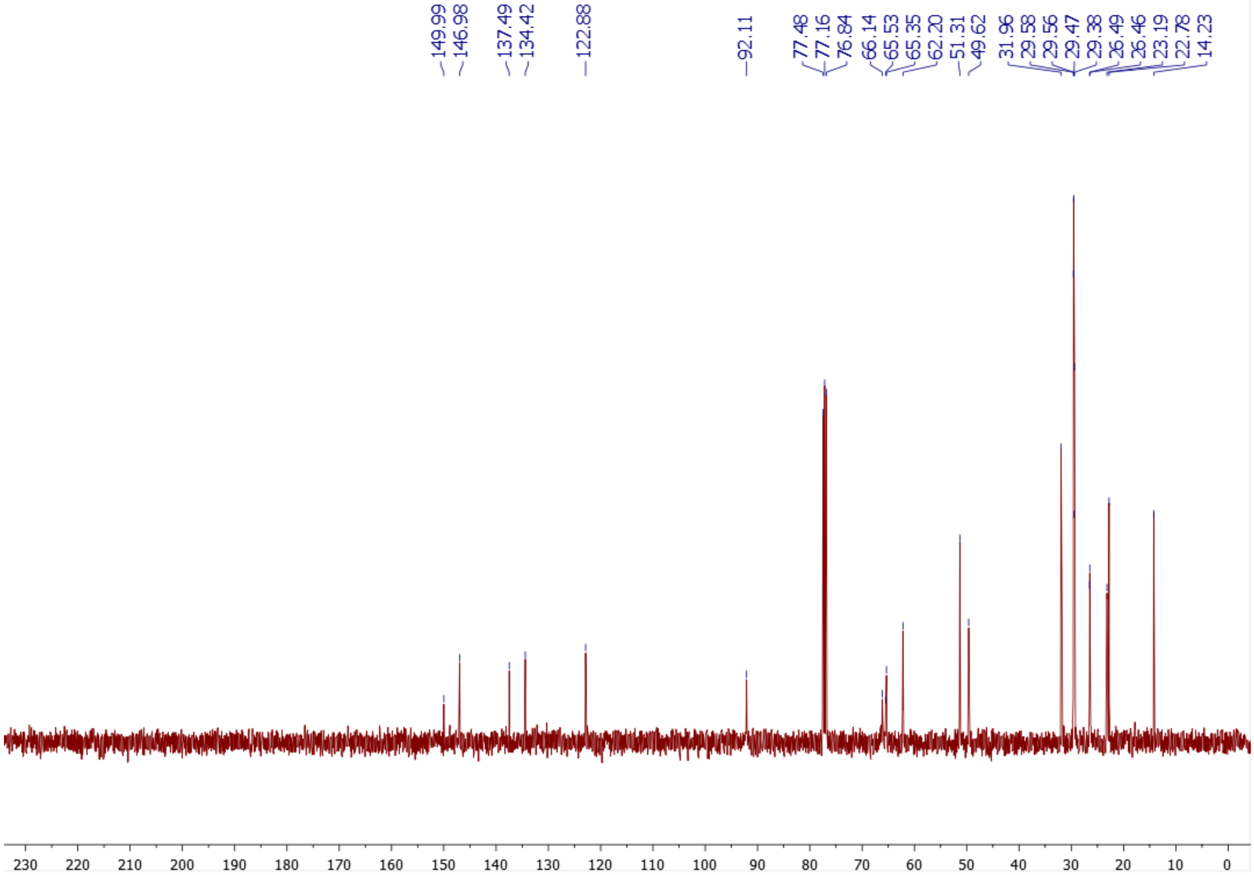

$^{13}C\{H\}$ NMR spectrum of compound **5a**$_{10}$

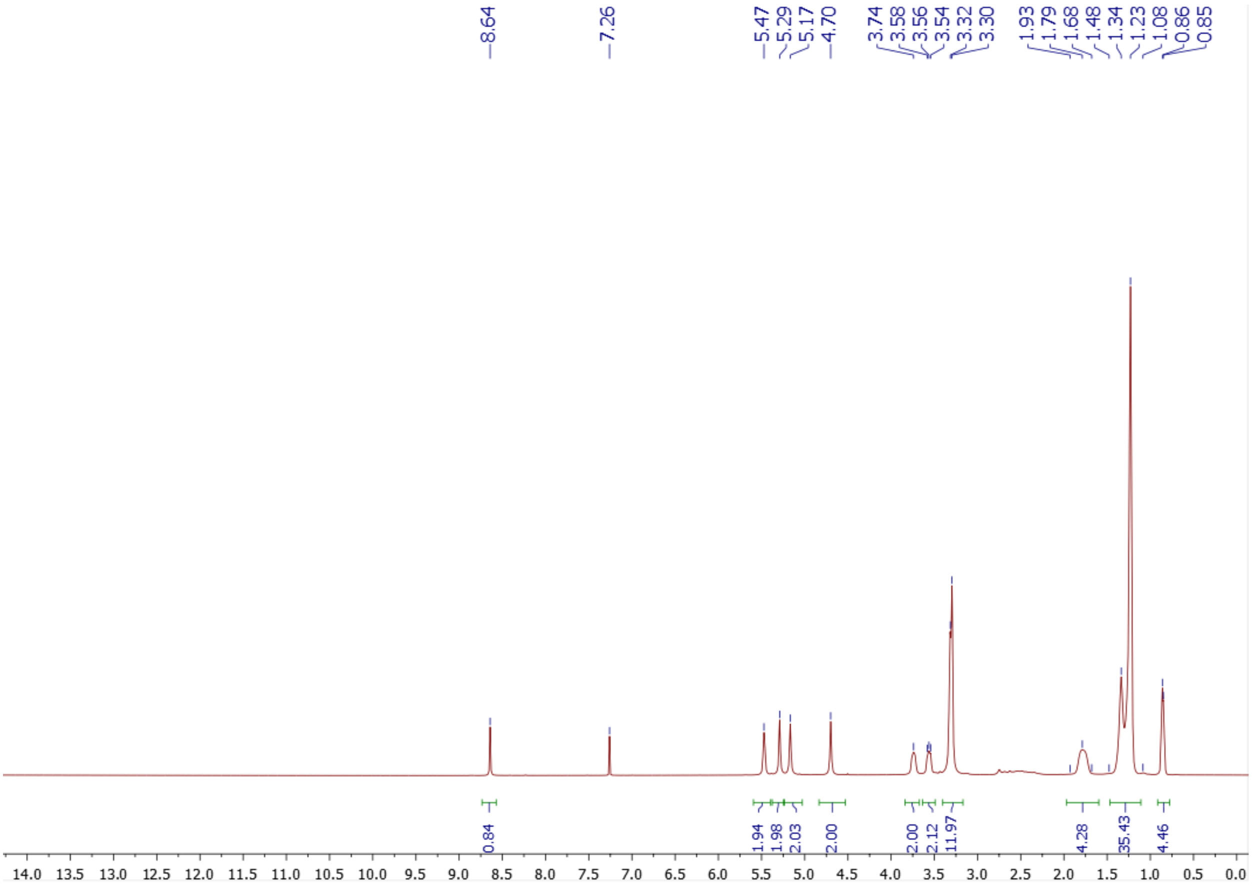

^{1}H NMR spectrum of compound **5a**12

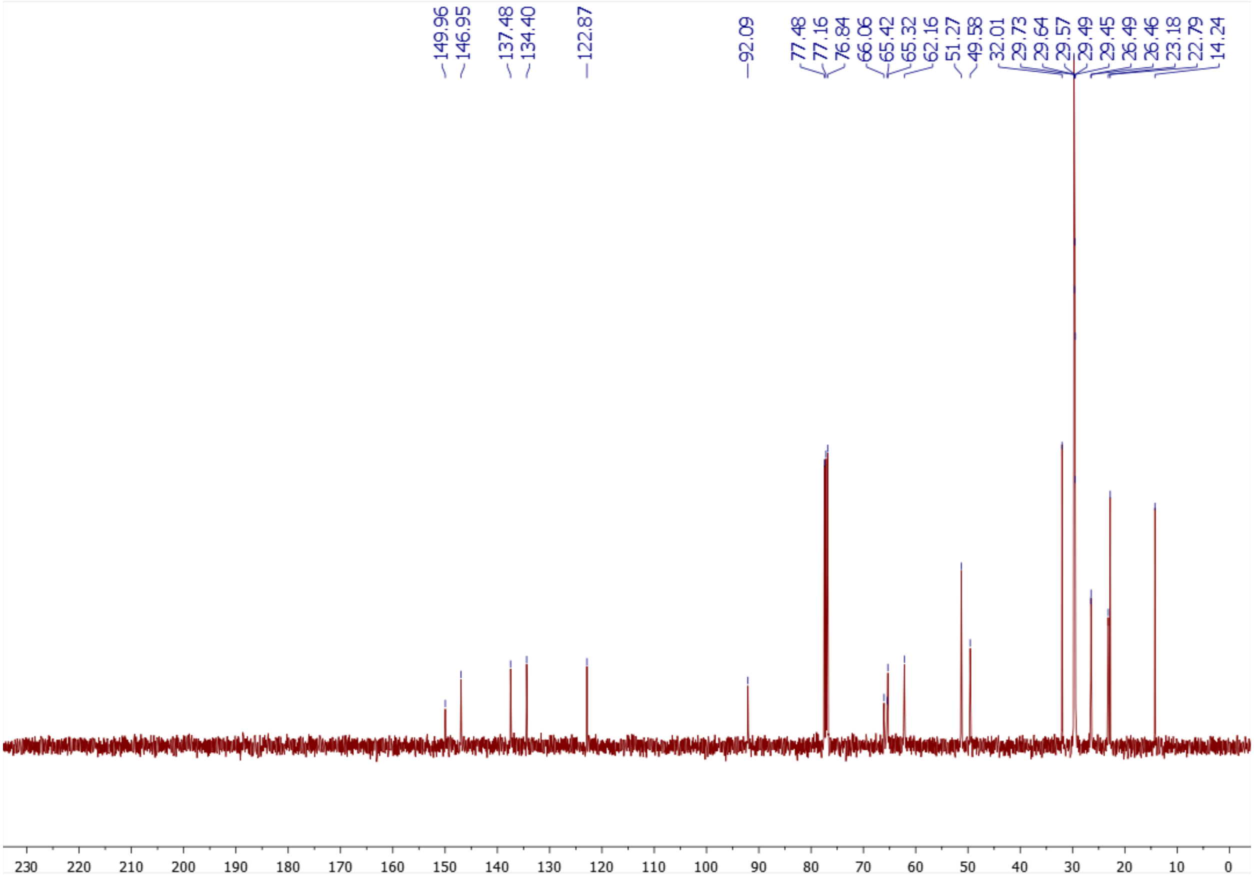

^{13}C{H} NMR spectrum of compound **5a**12

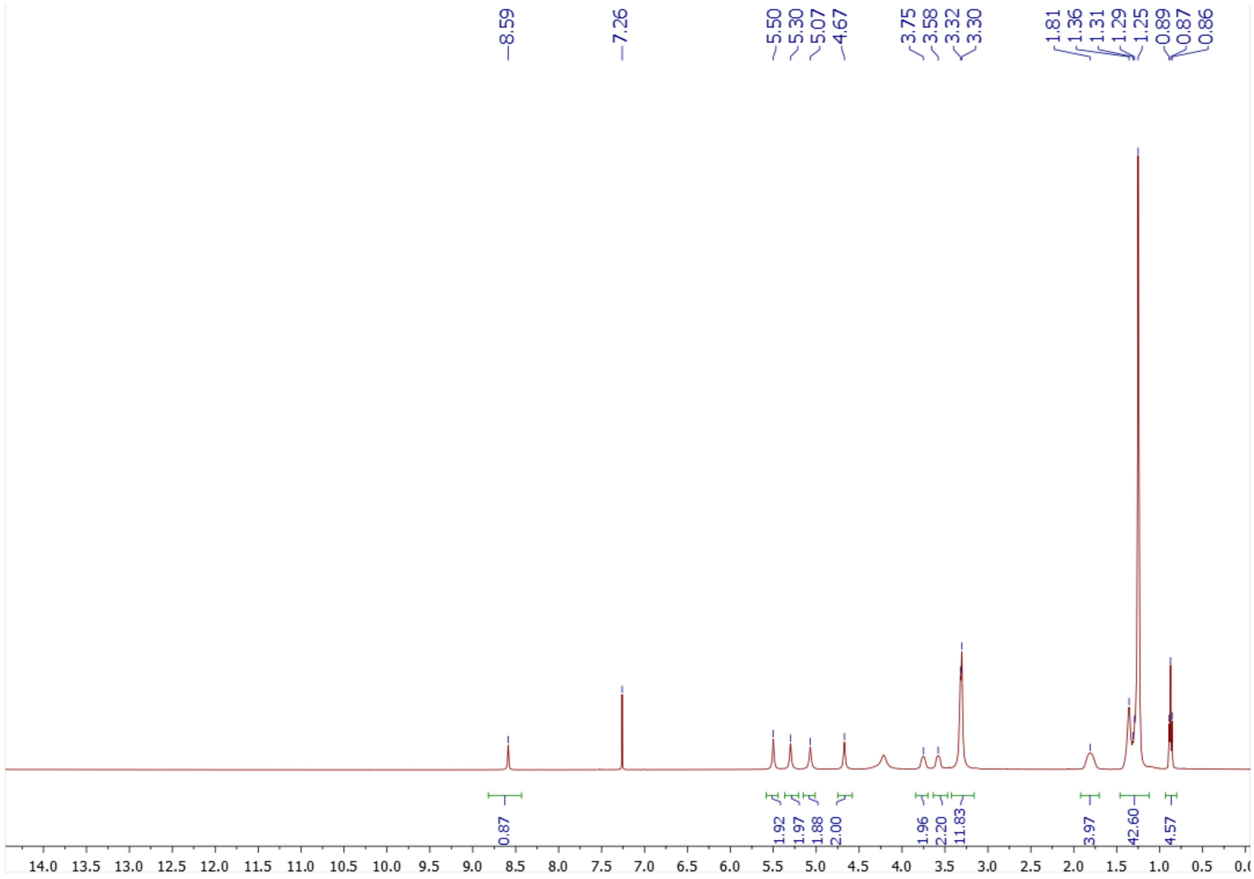

^{1}H NMR spectrum of compound **5a**$_{14}$

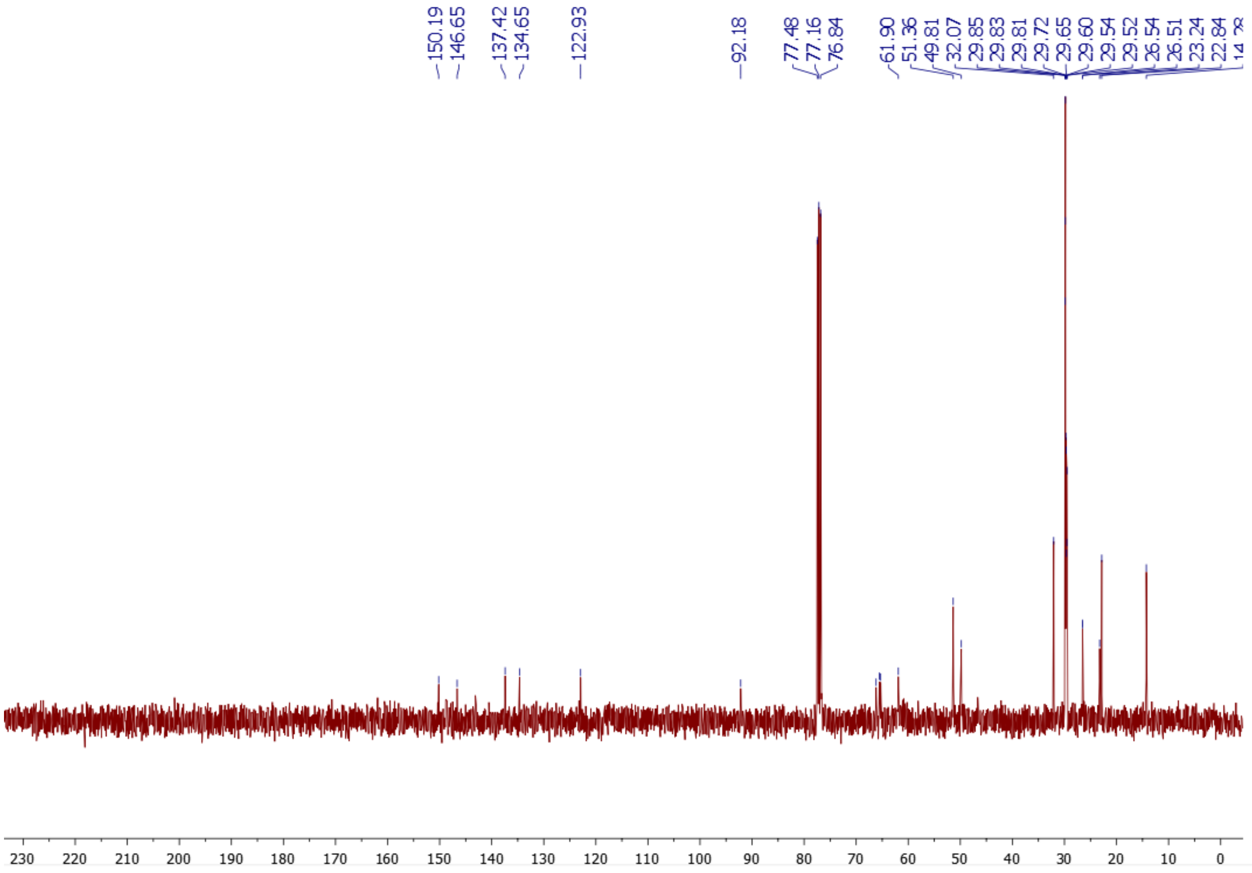

$^{13}C\{H\}$ NMR spectrum of compound **5a**$_{14}$

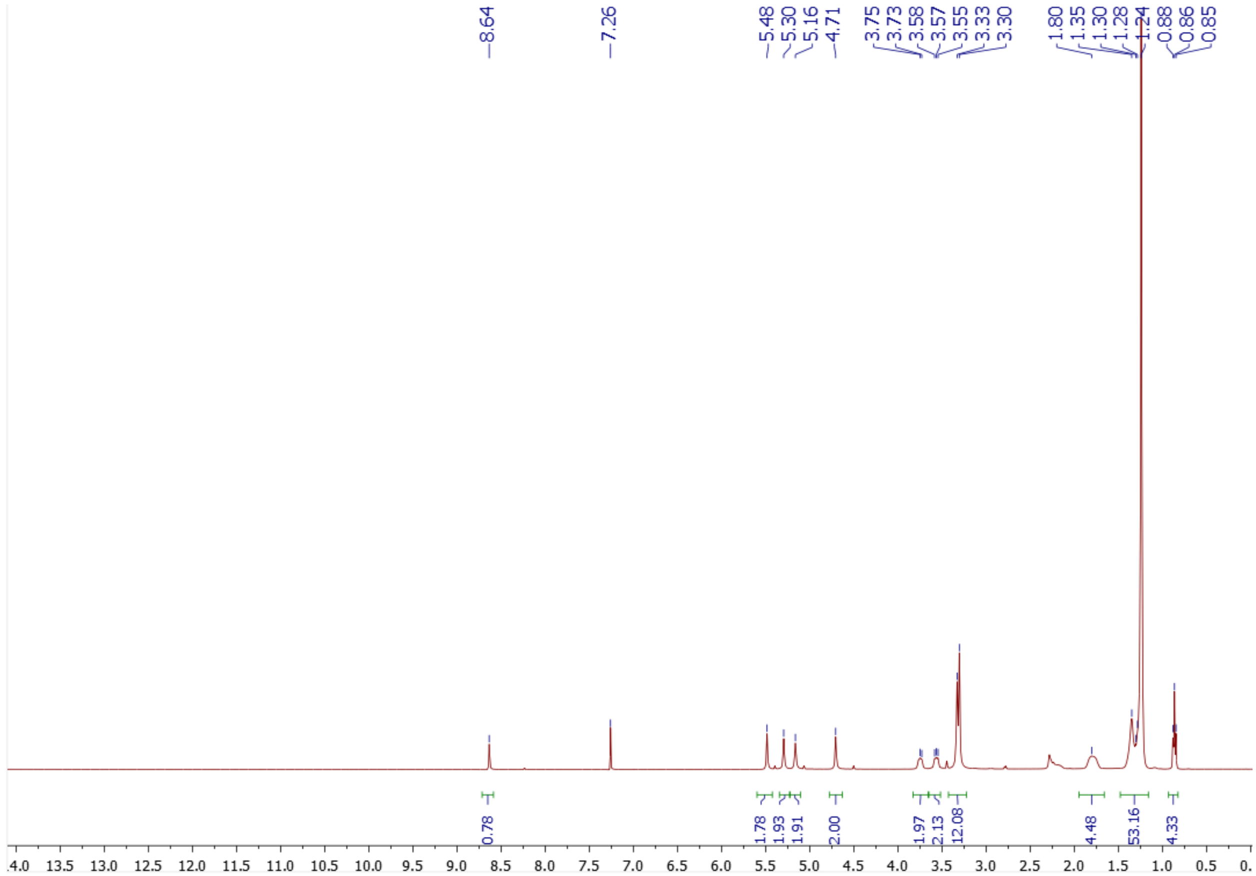

^{1}H NMR spectrum of compound **5a**16

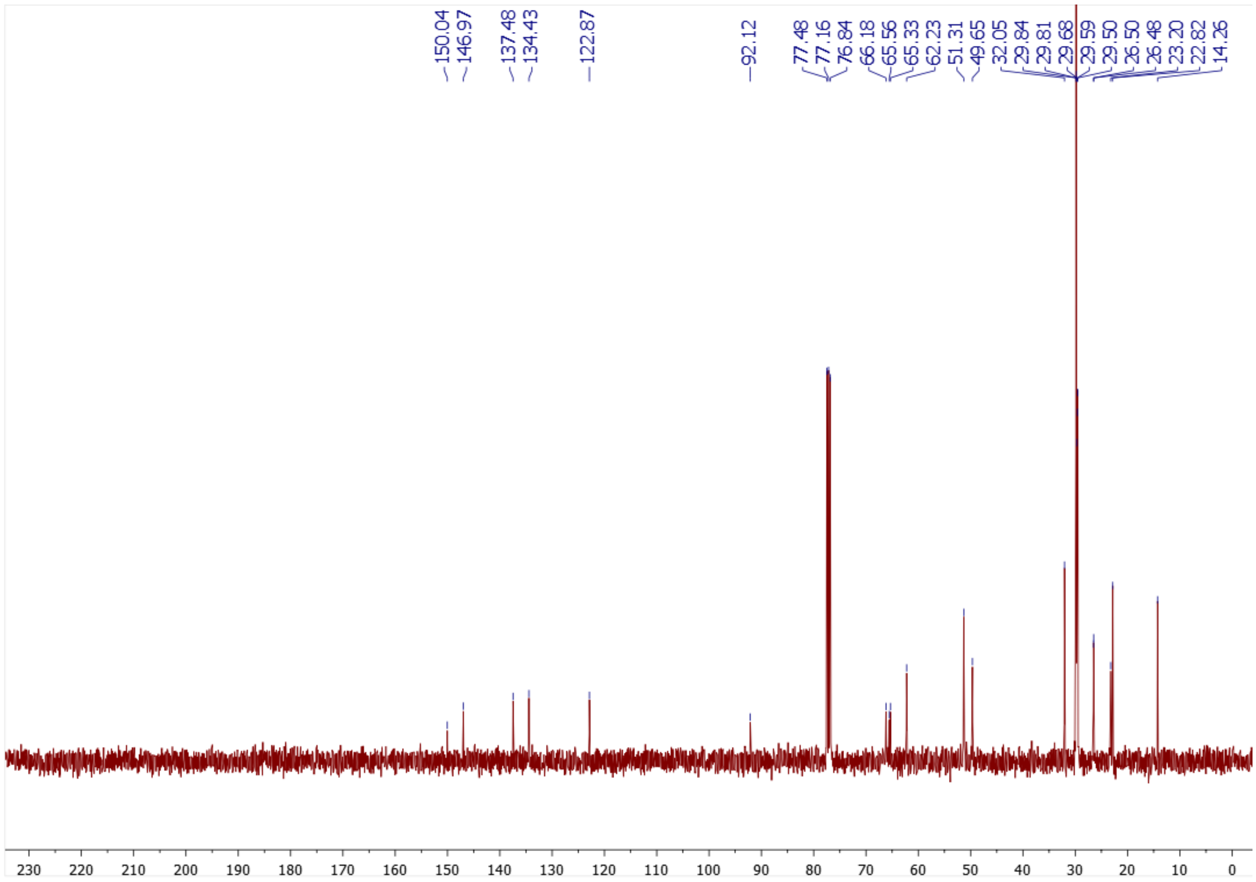

^{13}C{H} NMR spectrum of compound **5a**16

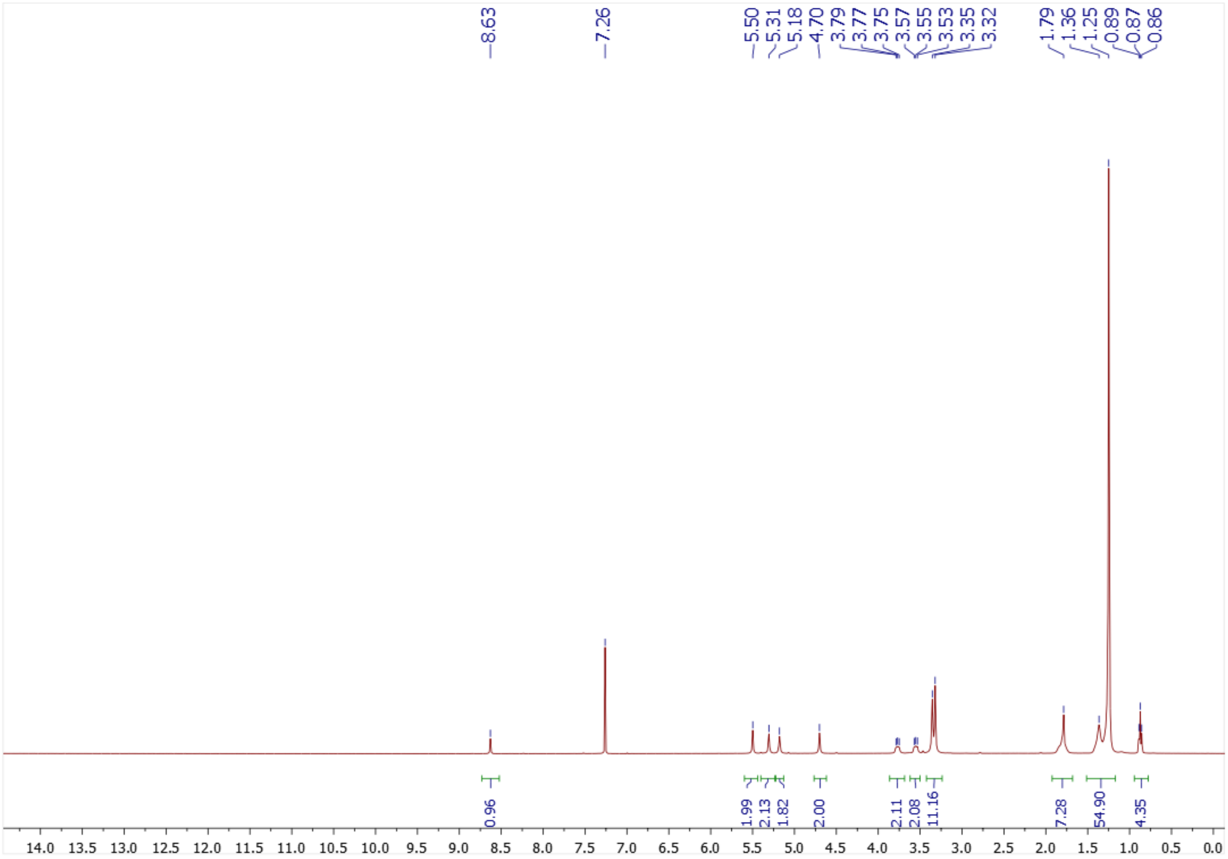

^{1}H NMR spectrum of compound **5a**18

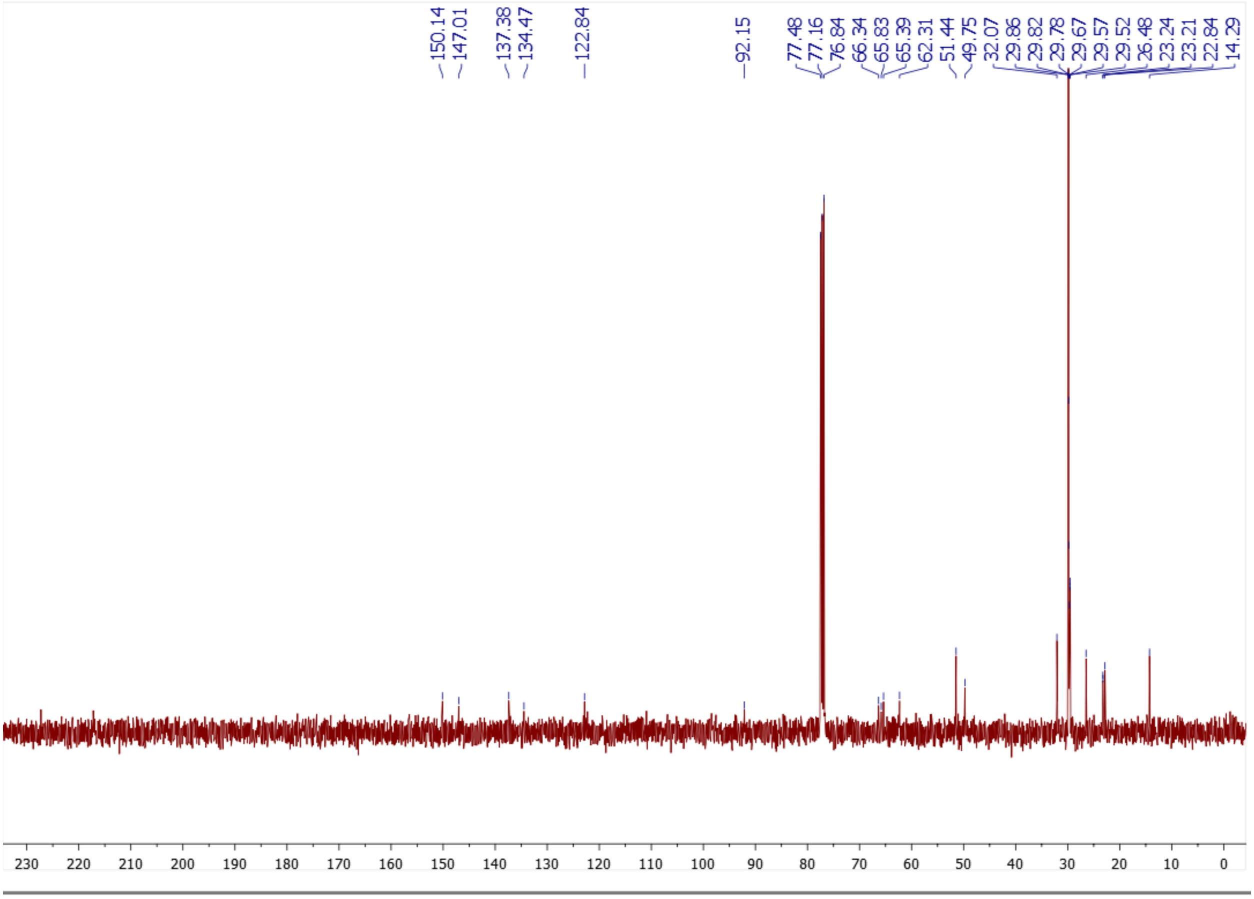

^{13}C{H} NMR spectrum of compound **5a**18

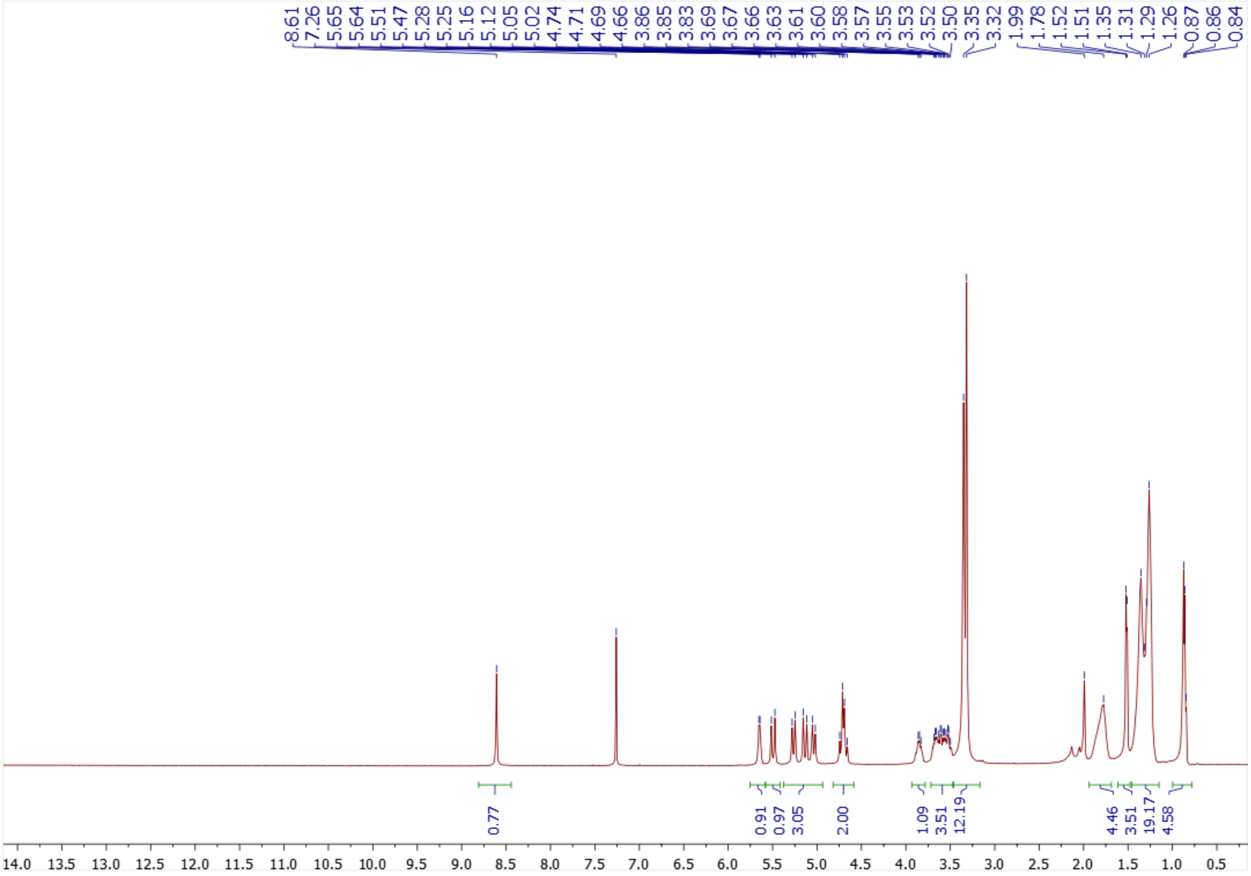

^{1}H NMR spectrum of compound **5b**$_{8}$

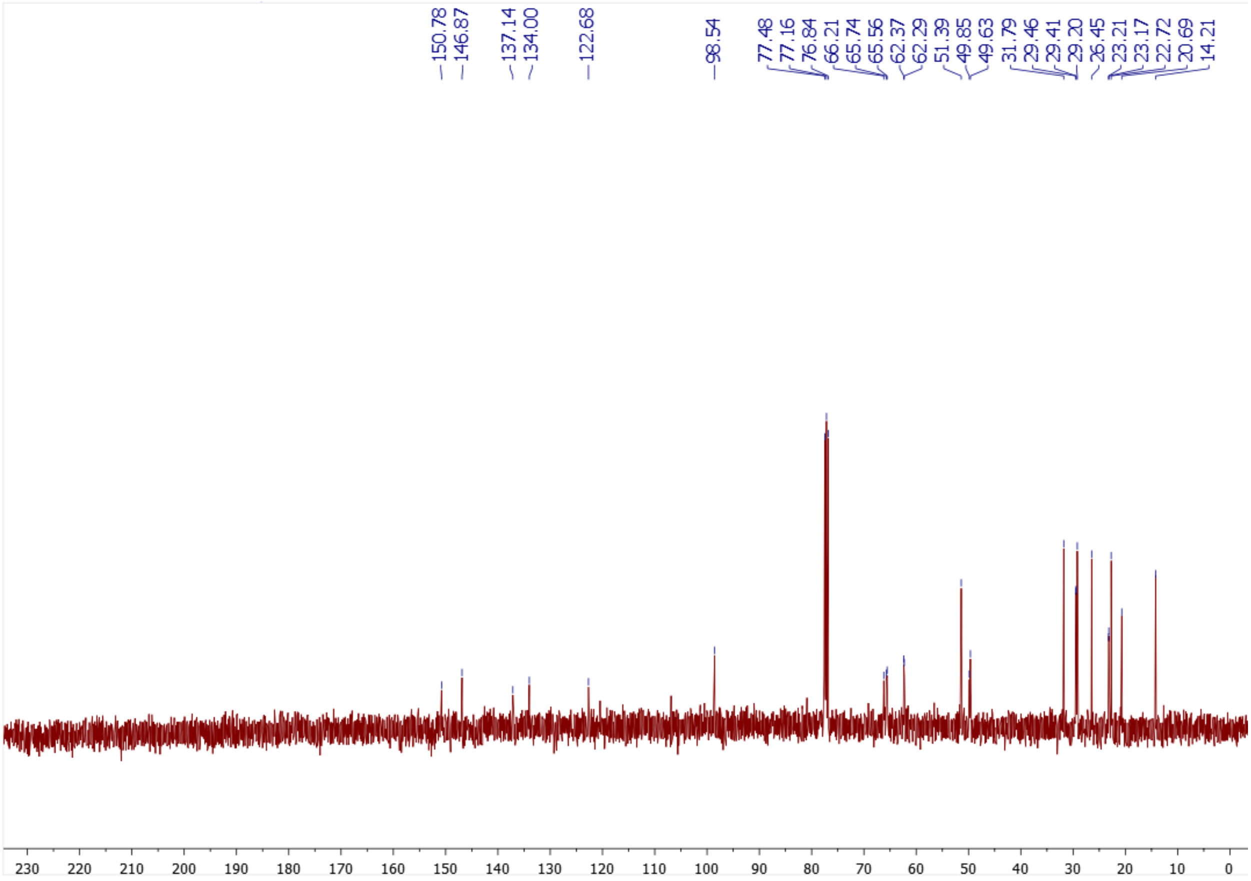

^{13}C{H} NMR spectrum of compound **5b**$_{8}$

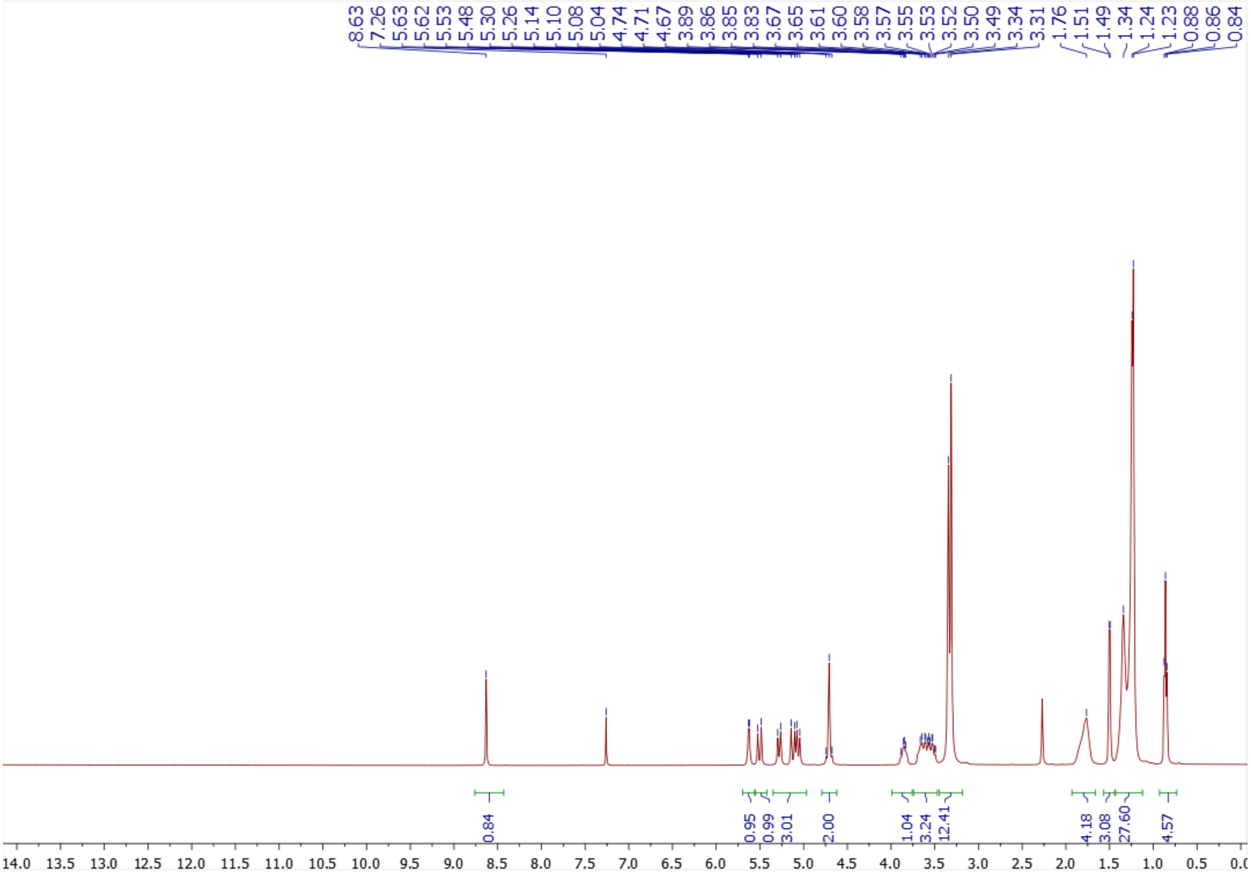

^{1}H NMR spectrum of compound **5b**10

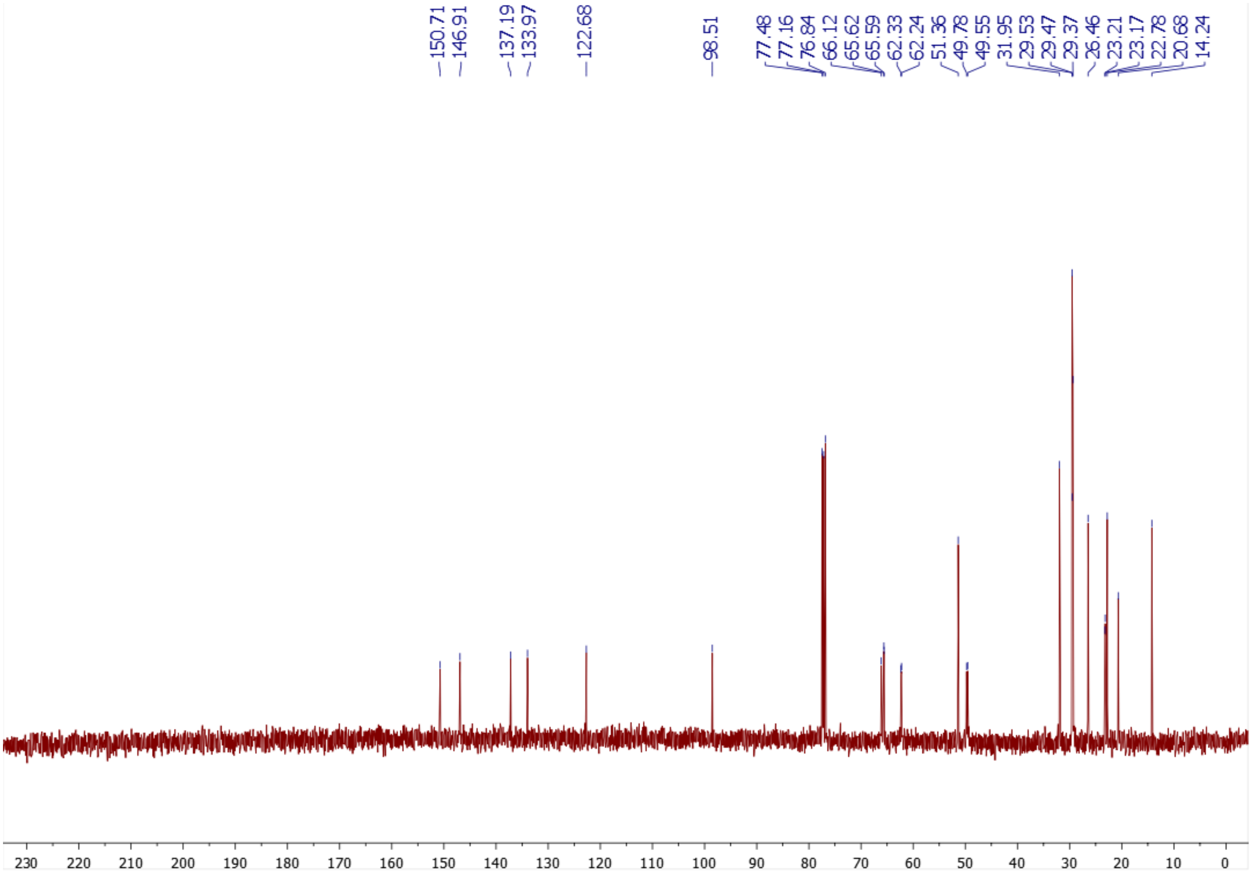

^{13}C{H} NMR spectrum of compound **5b**10

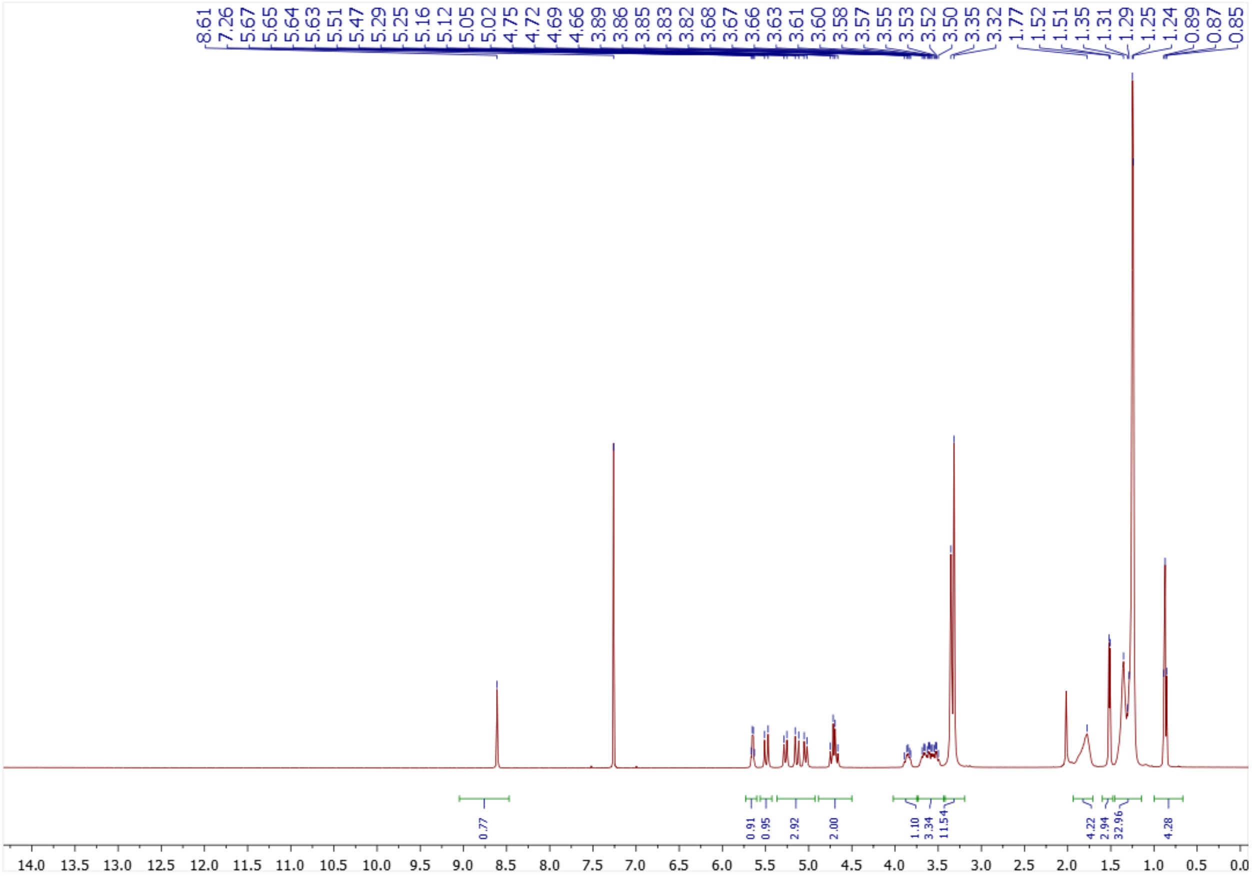

^{1}H NMR spectrum of compound **5b**$_{12}$

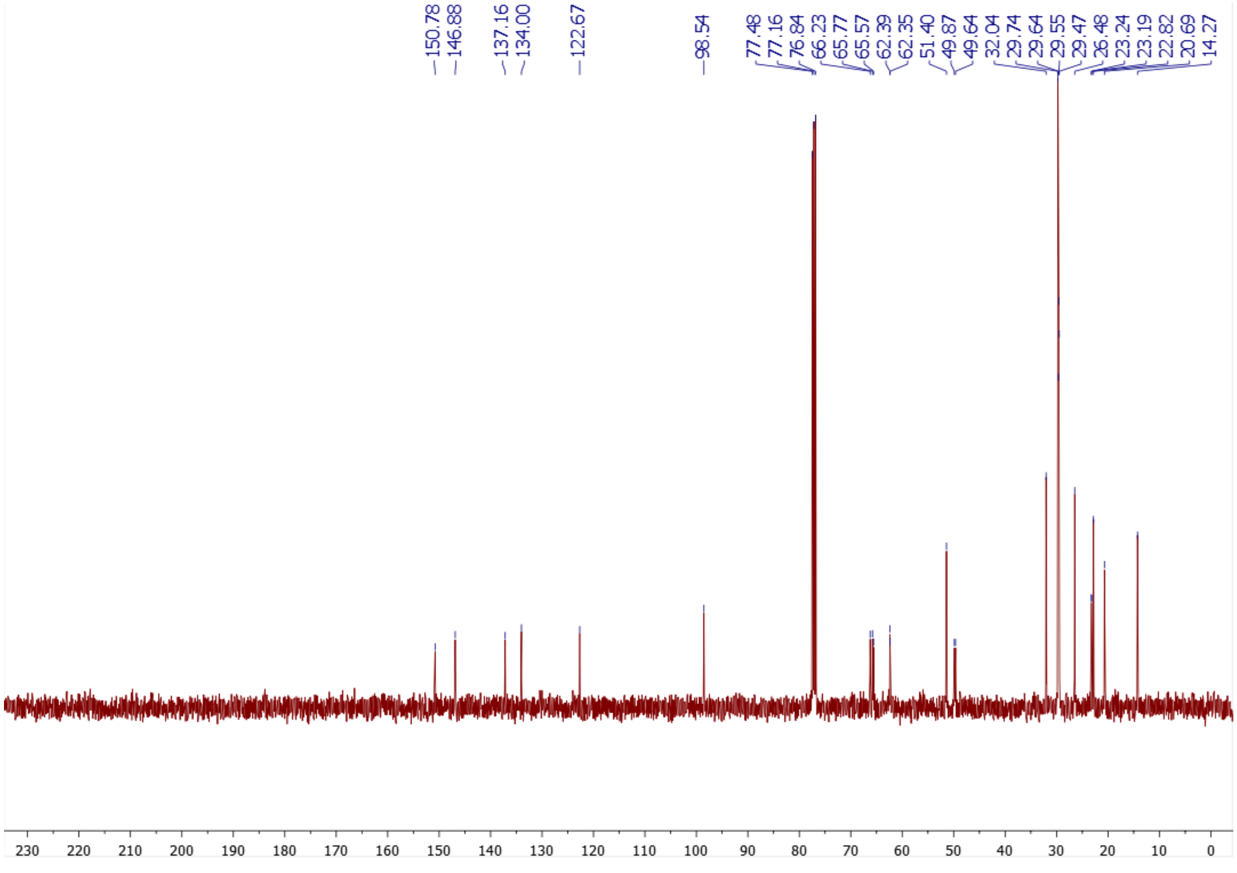

^{13}C{H} NMR spectrum of compound **5b**$_{12}$

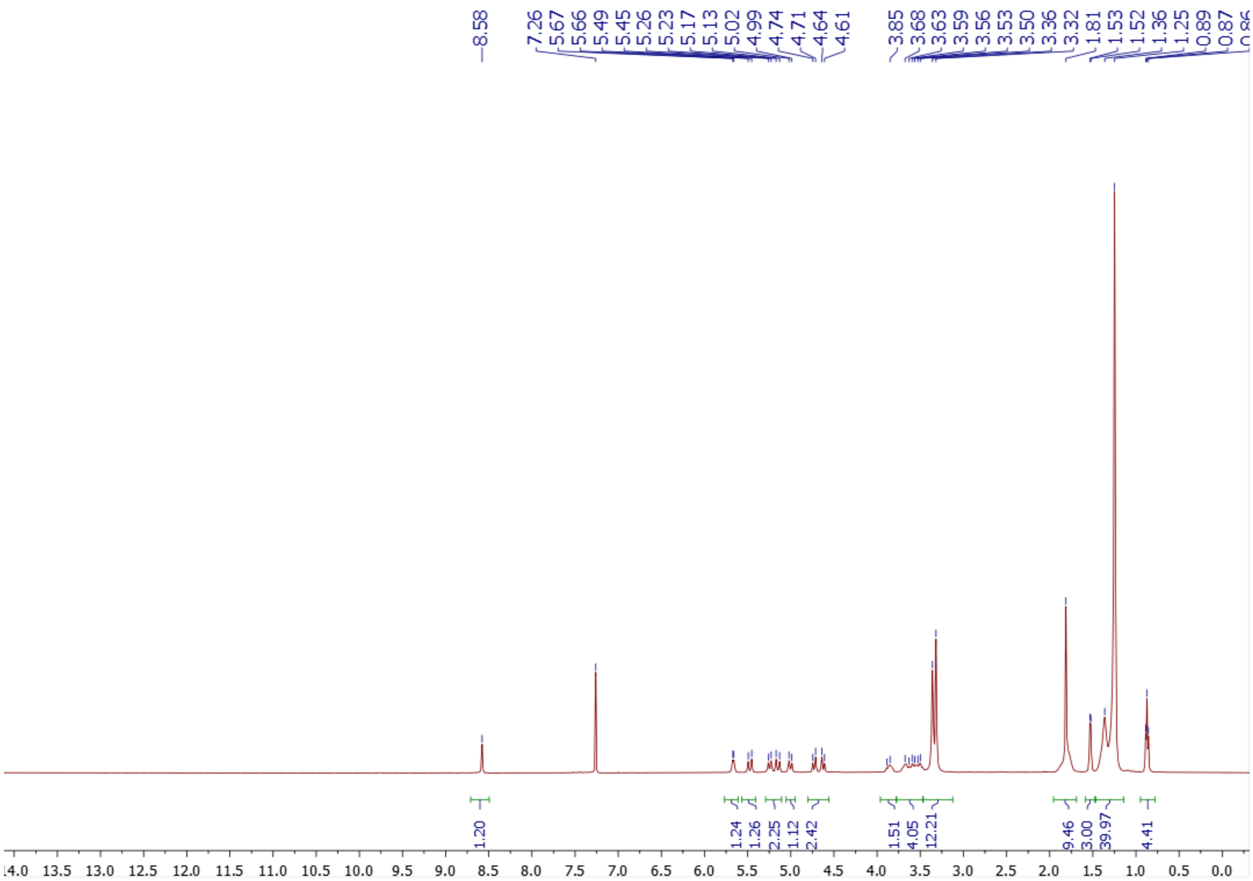

^{1}H NMR spectrum of compound **5b**₁₄

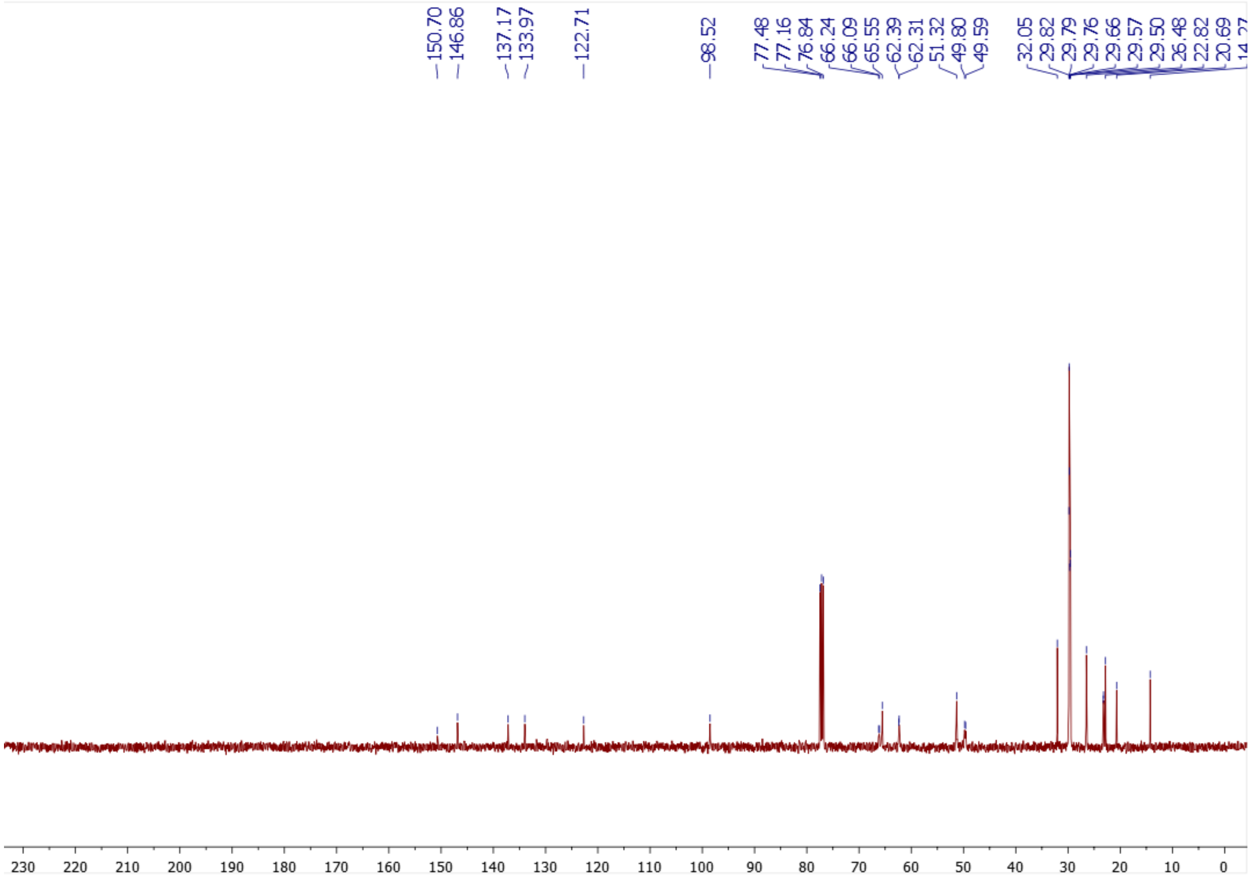

^{13}C{H} NMR spectrum of compound **5b**₁₄

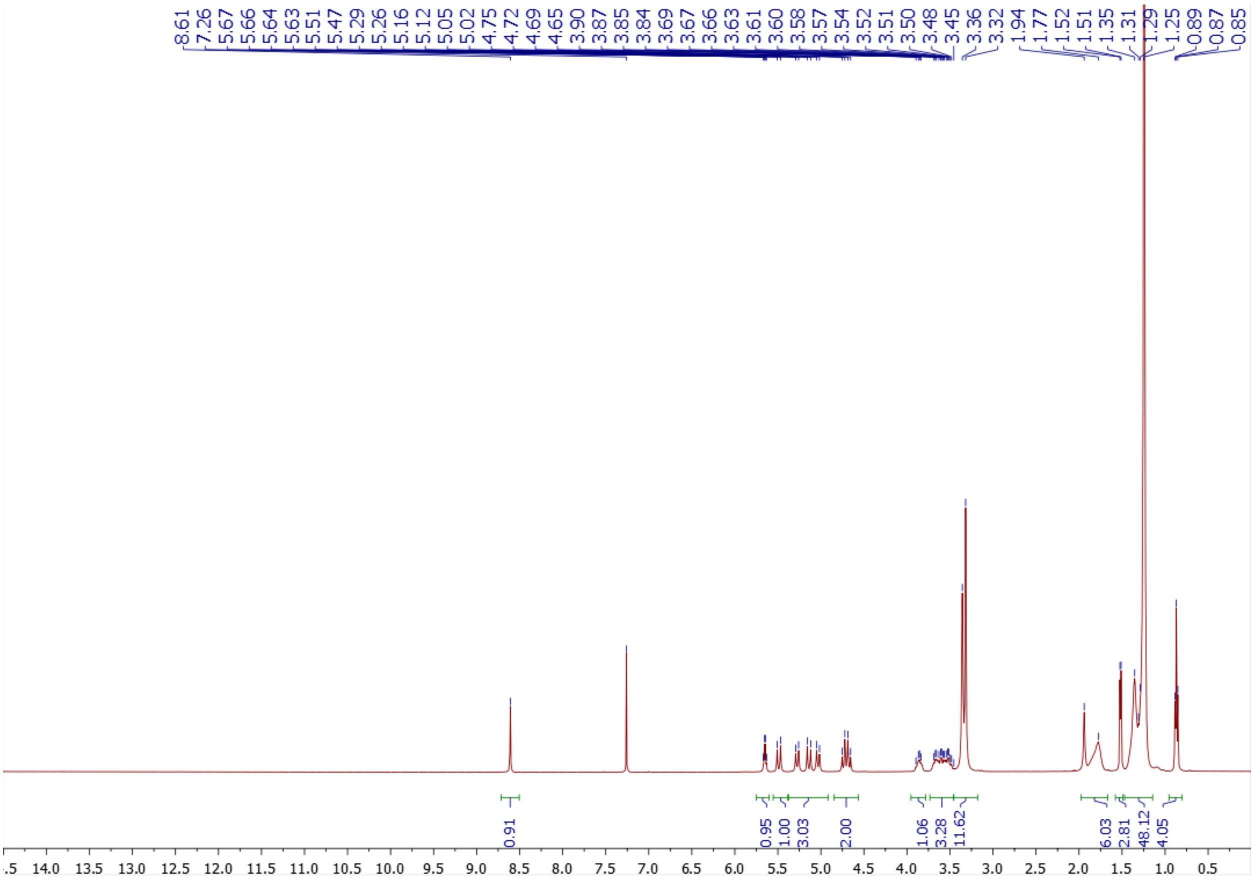

^{1}H NMR spectrum of compound **5b**$_{16}$

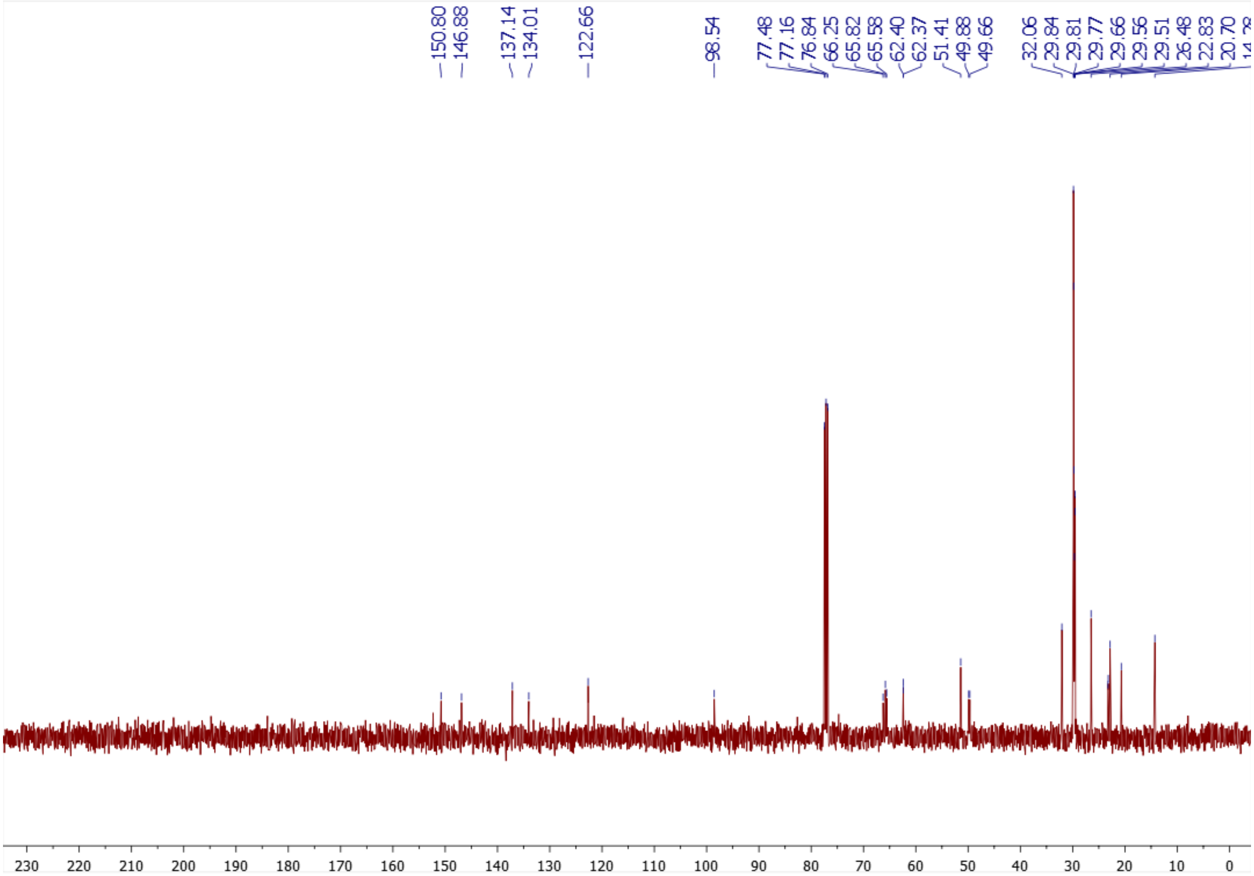

^{13}C{H} NMR spectrum of compound **5b**$_{16}$

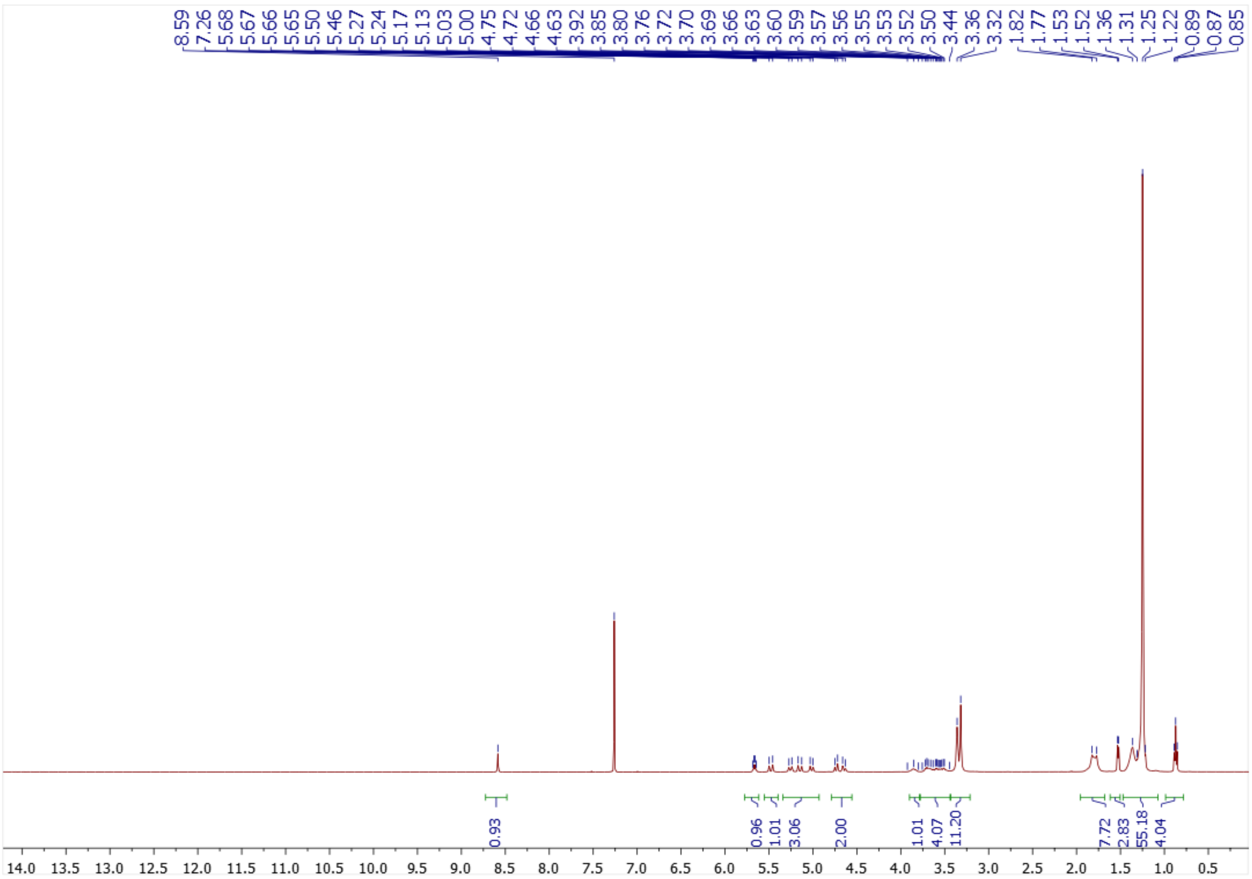

^{1}H NMR spectrum of compound **5b**$_{18}$

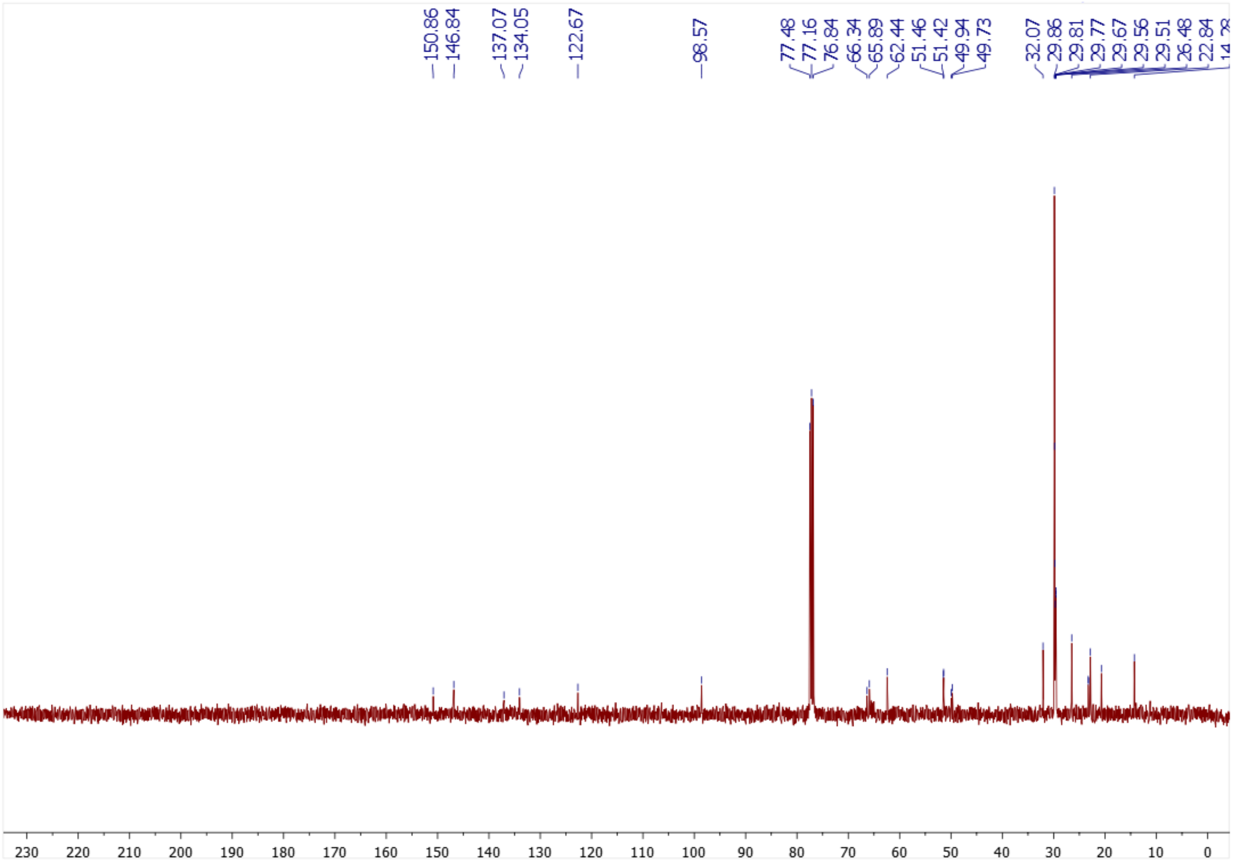

^{13}C{H} NMR spectrum of compound **5b**$_{18}$

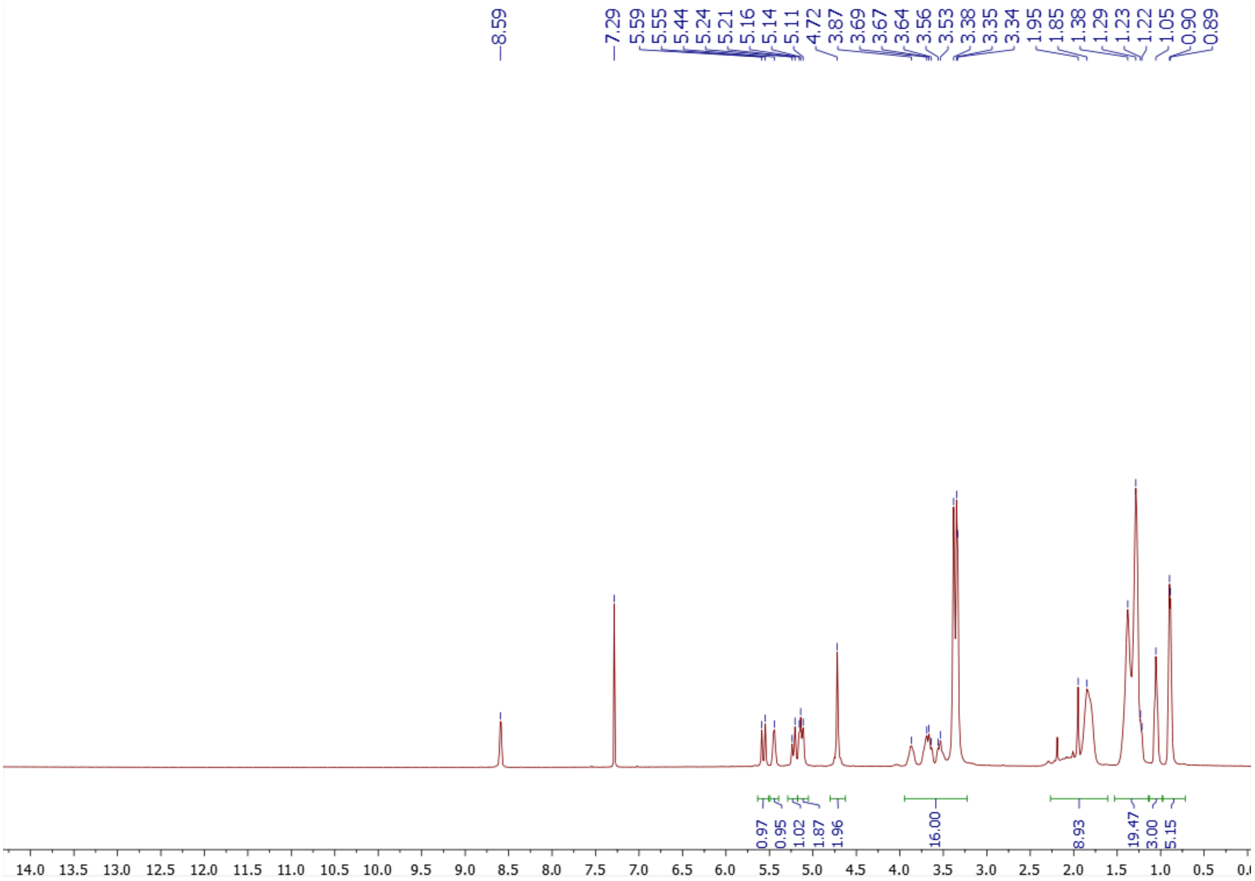

^{1}H NMR spectrum of compound **5c**8

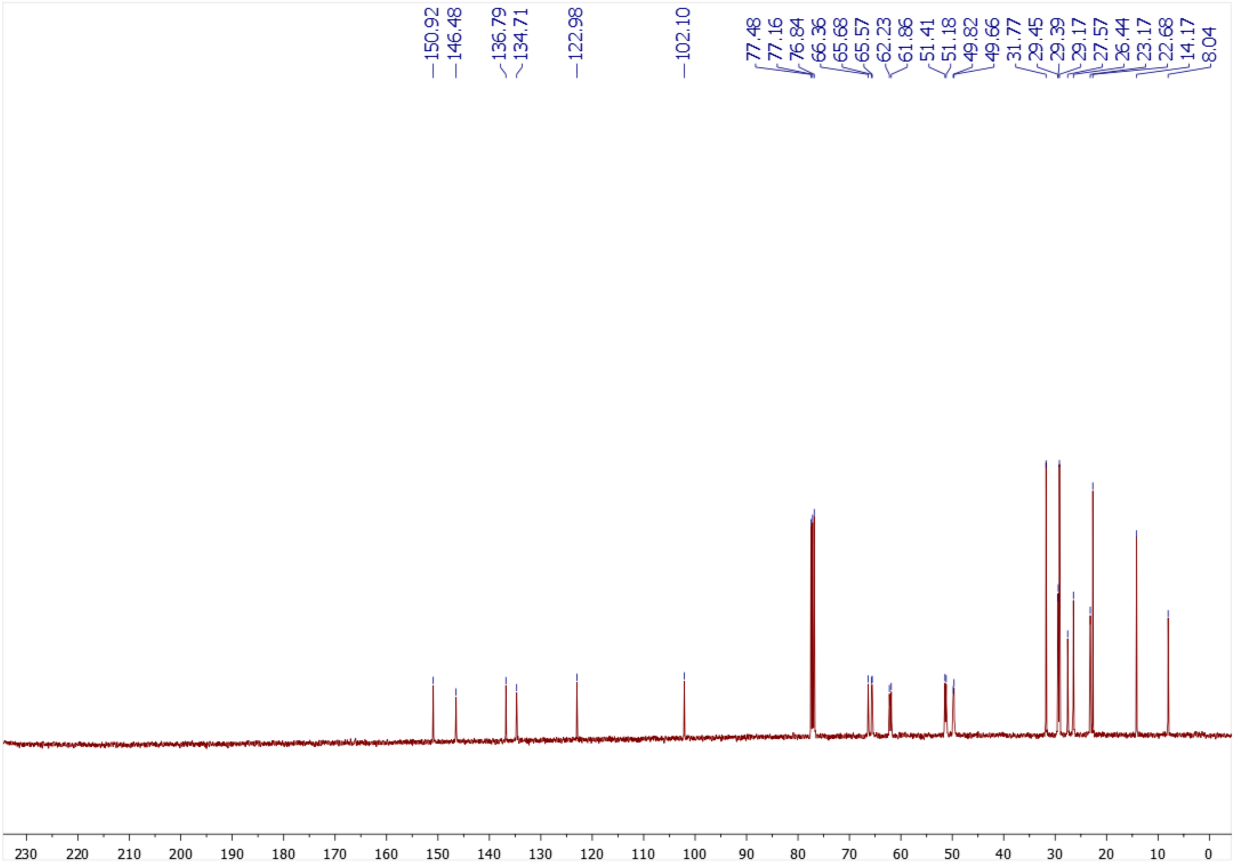

^{13}C{H} NMR spectrum of compound **5c**8

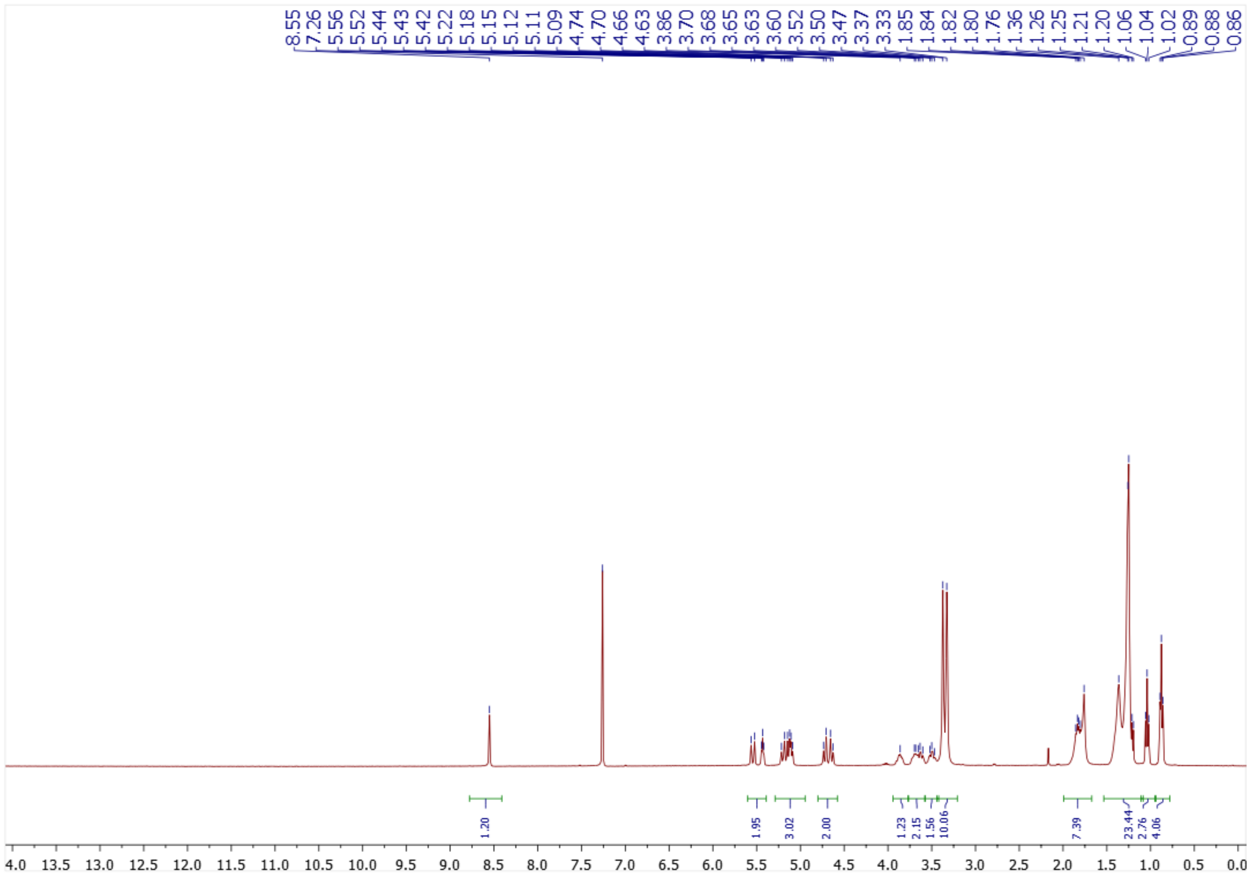

^{1}H NMR spectrum of compound **5c10**

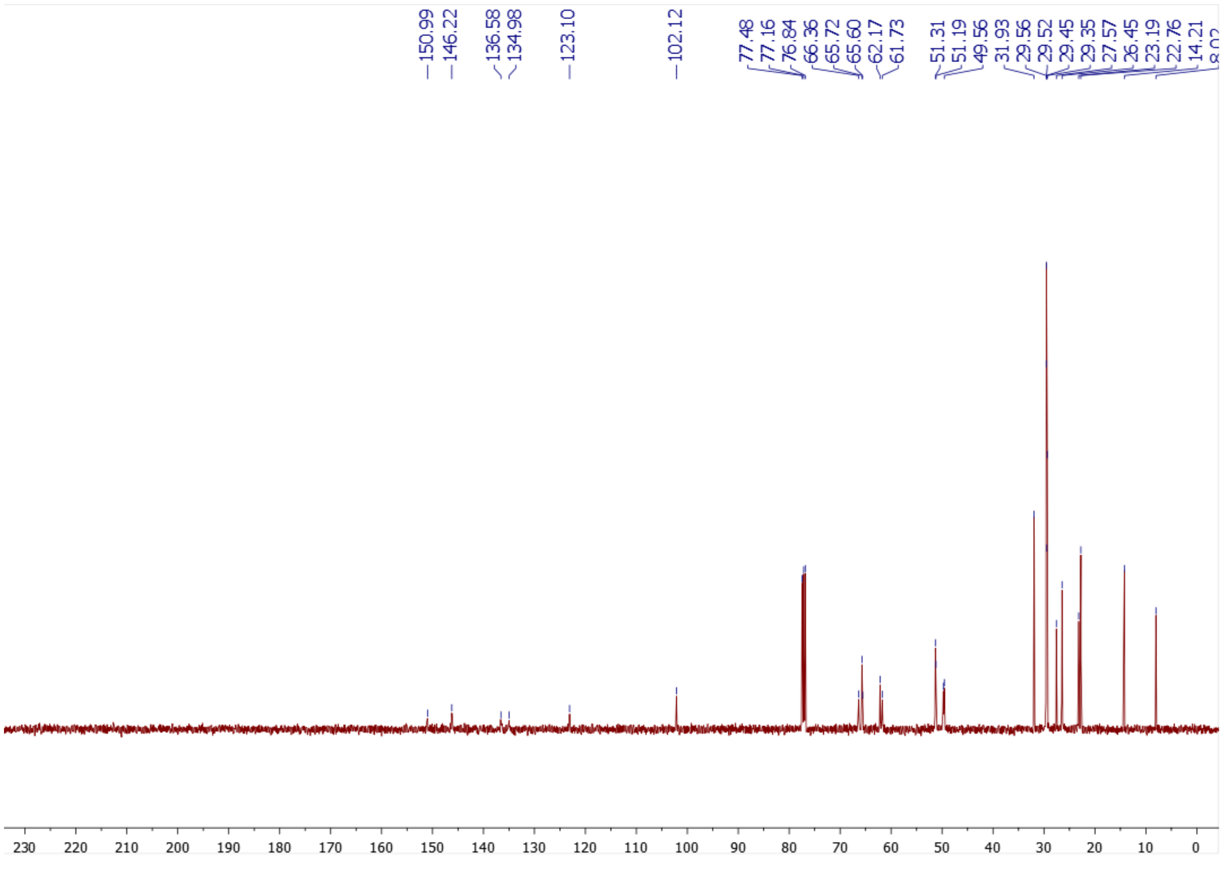

^{13}C{H} NMR spectrum of compound **5c10**

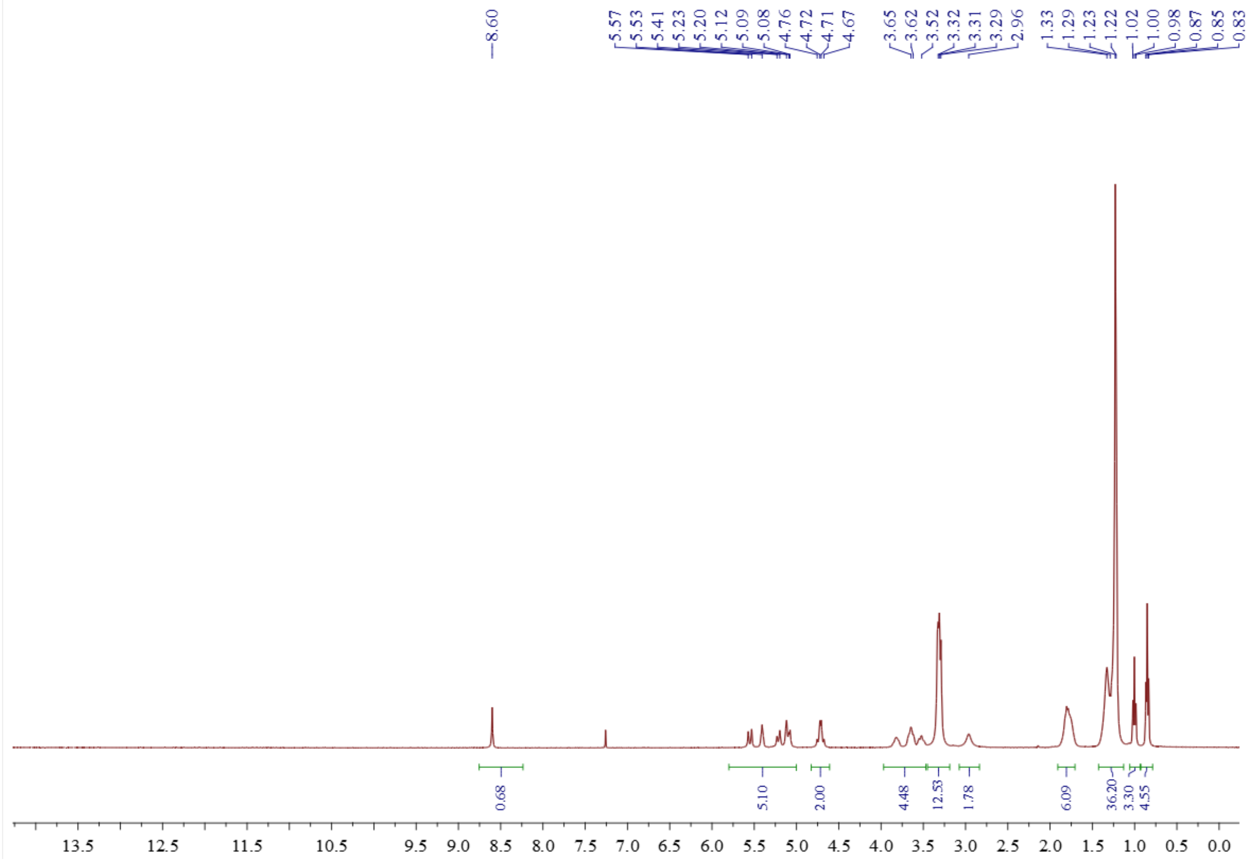

^{1}H NMR spectrum of compound **5c**$_{12}$

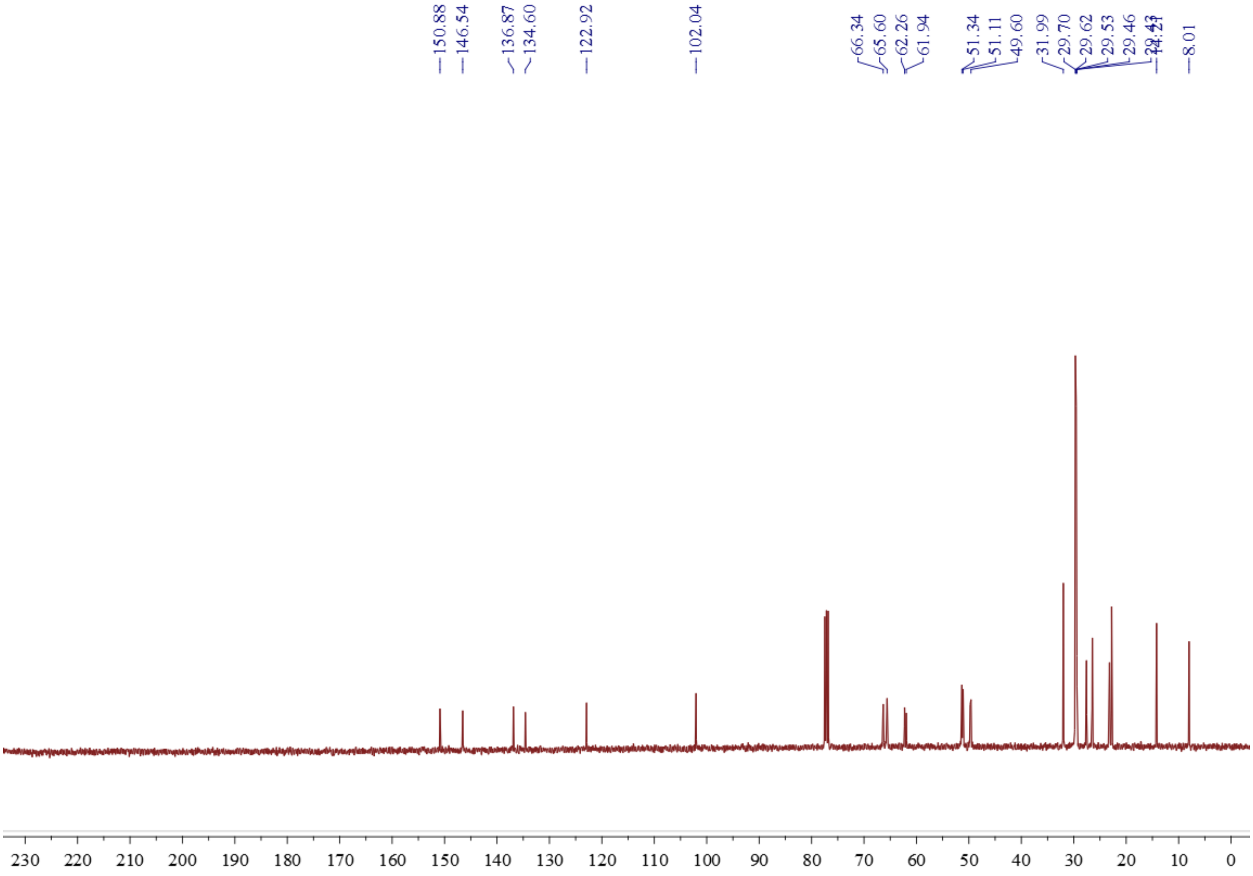

^{13}C{H} NMR spectrum of compound **5c**$_{12}$

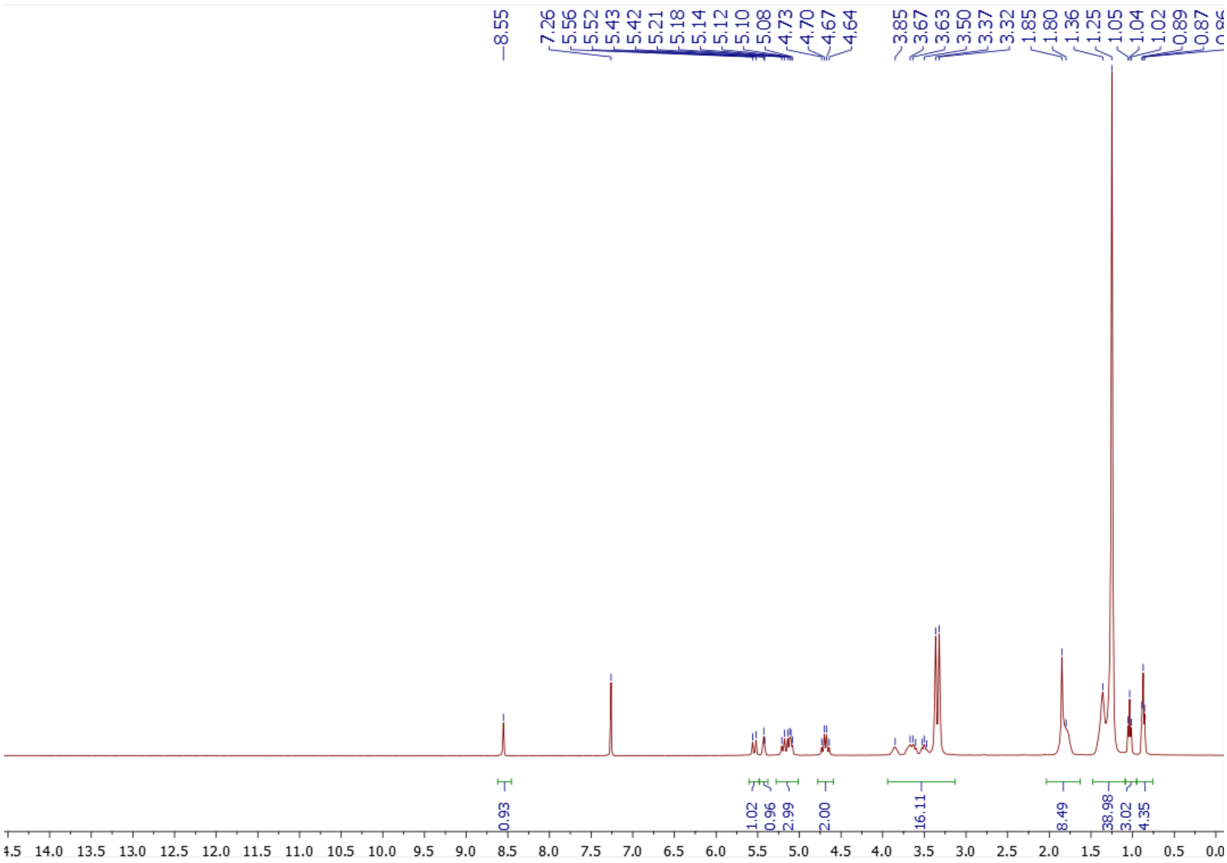

^{1}H NMR spectrum of compound **5c**$_{14}$

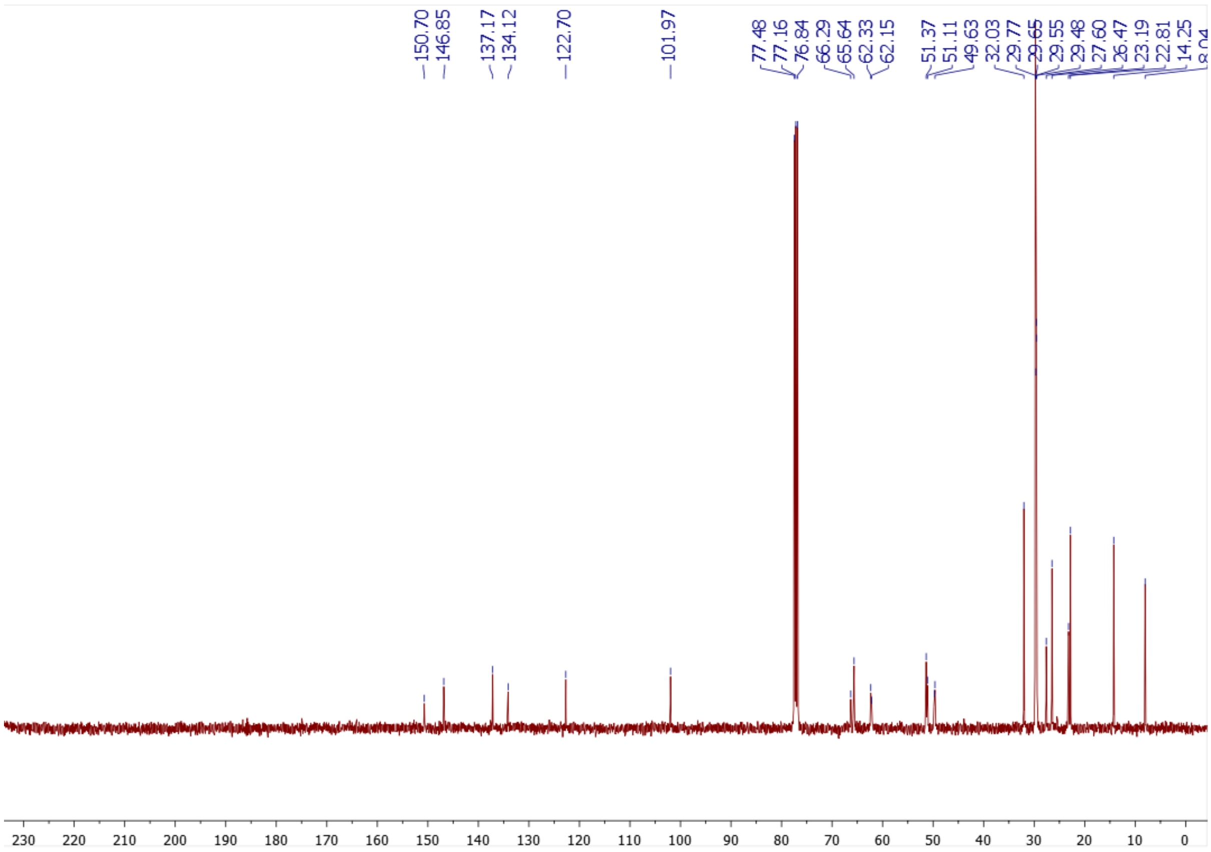

^{13}C{H} NMR spectrum of compound **5c**$_{14}$

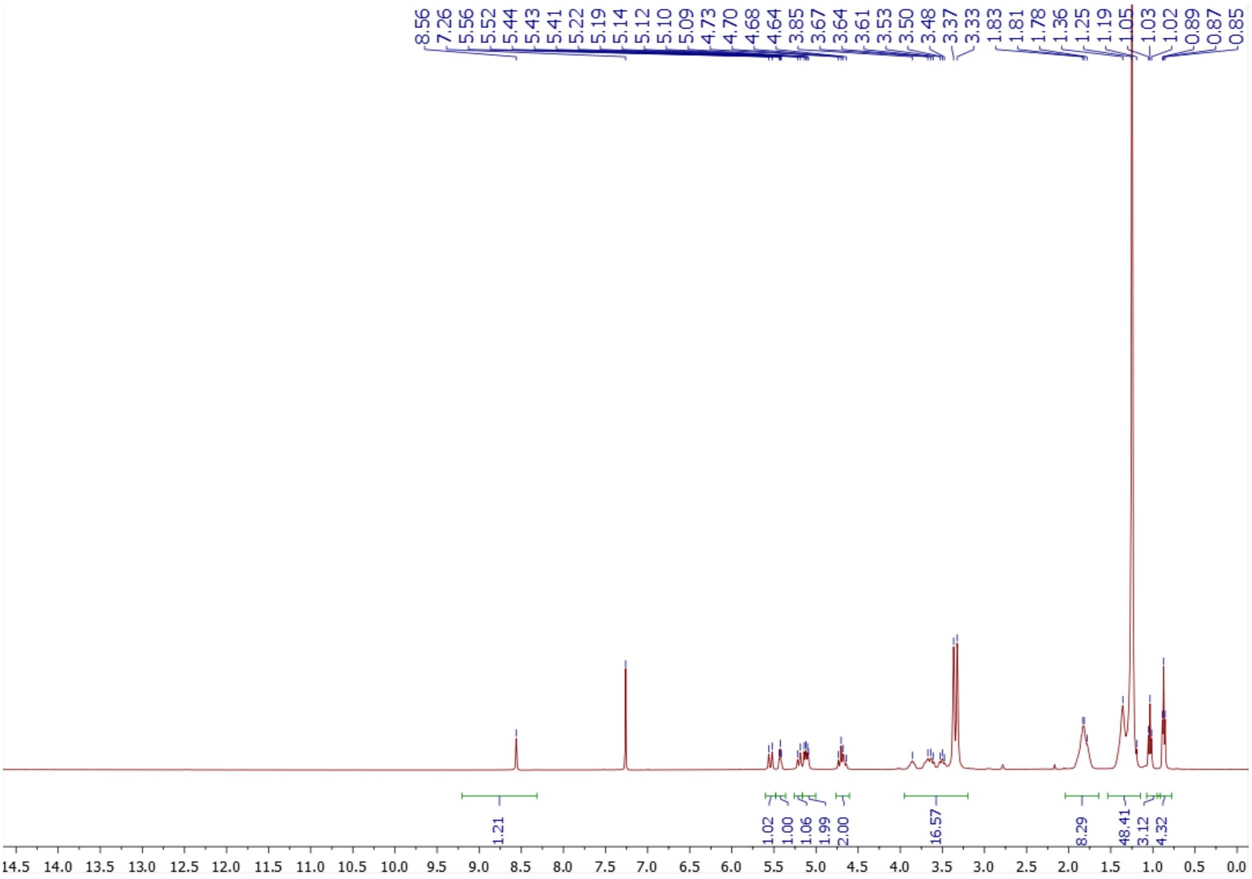

^{1}H NMR spectrum of compound **5c$_{16}$**

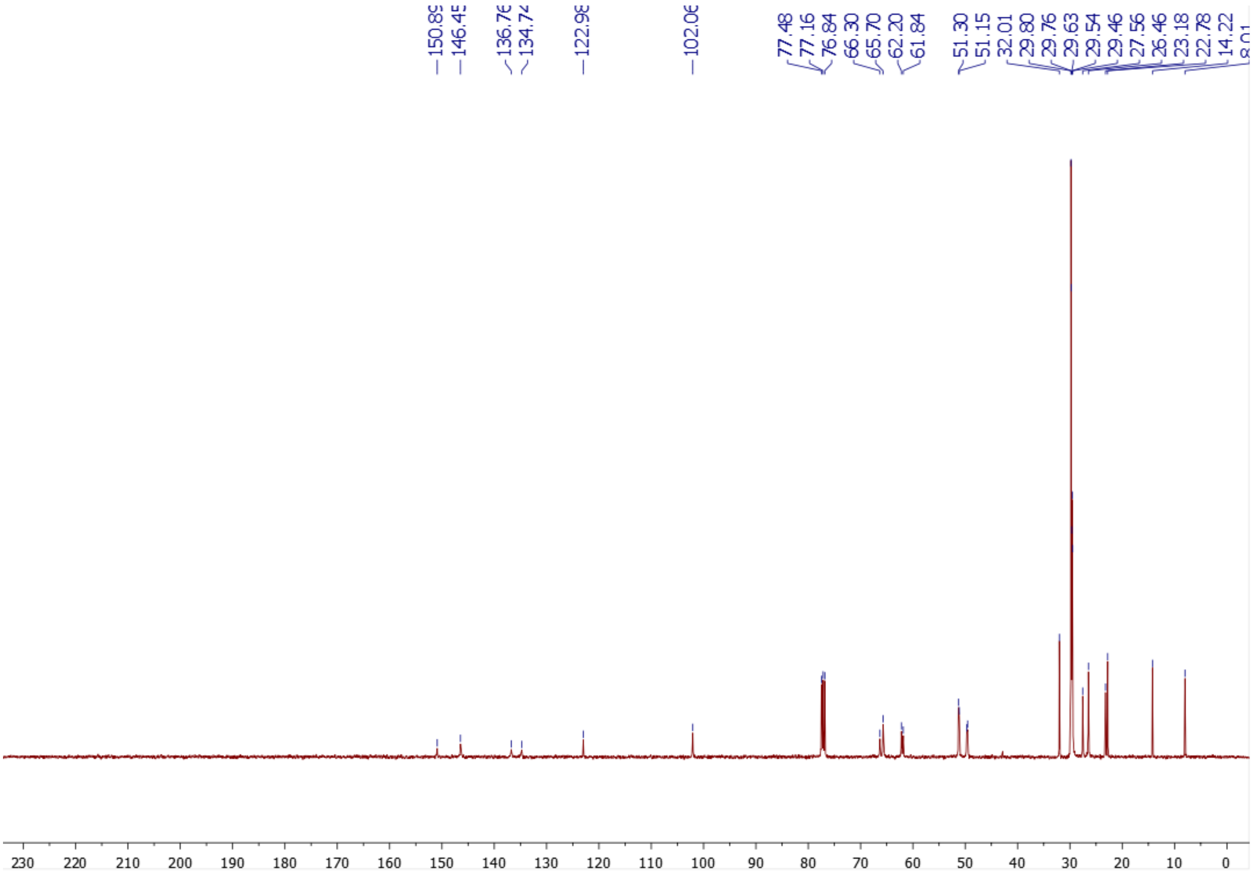

^{13}C{H} NMR spectrum of compound **5c$_{16}$**

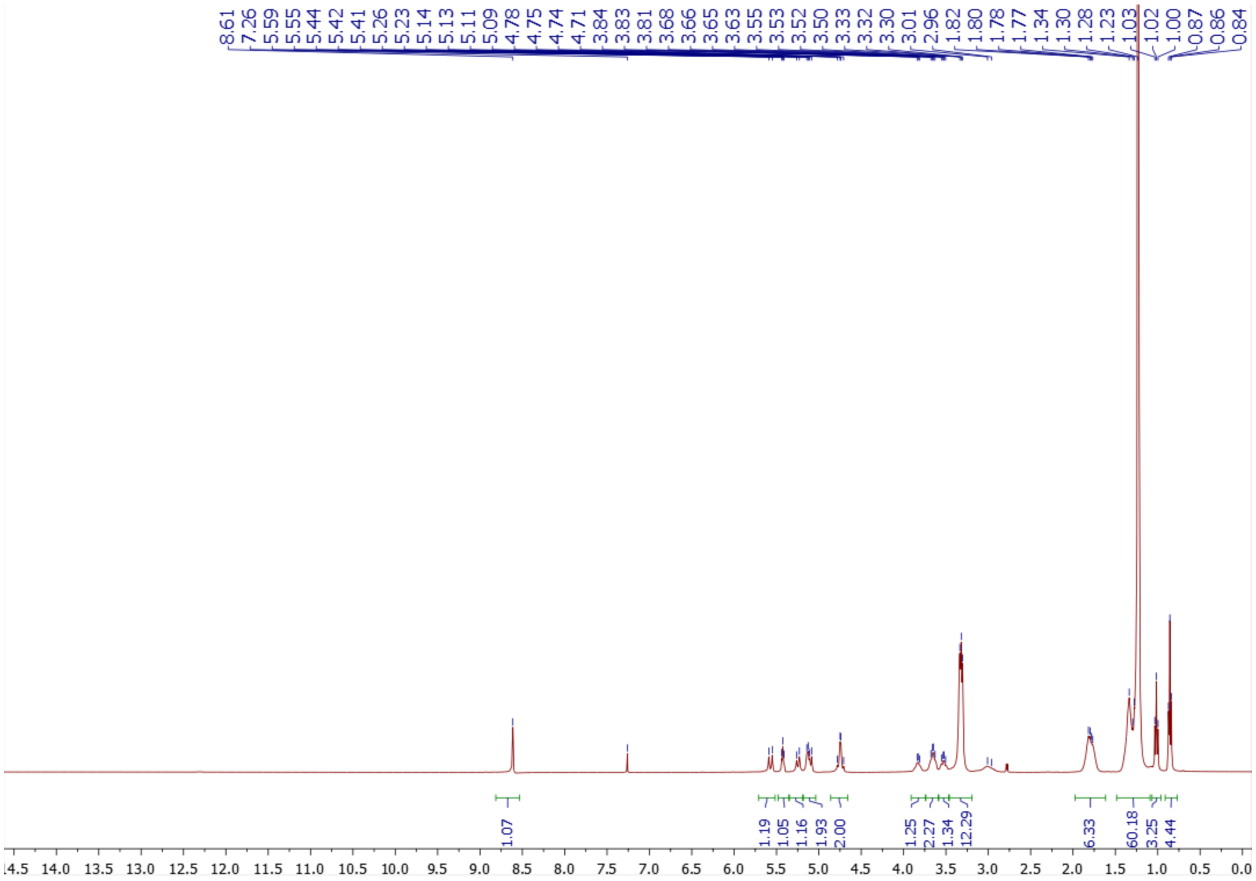

^{1}H NMR spectrum of compound **5c18**

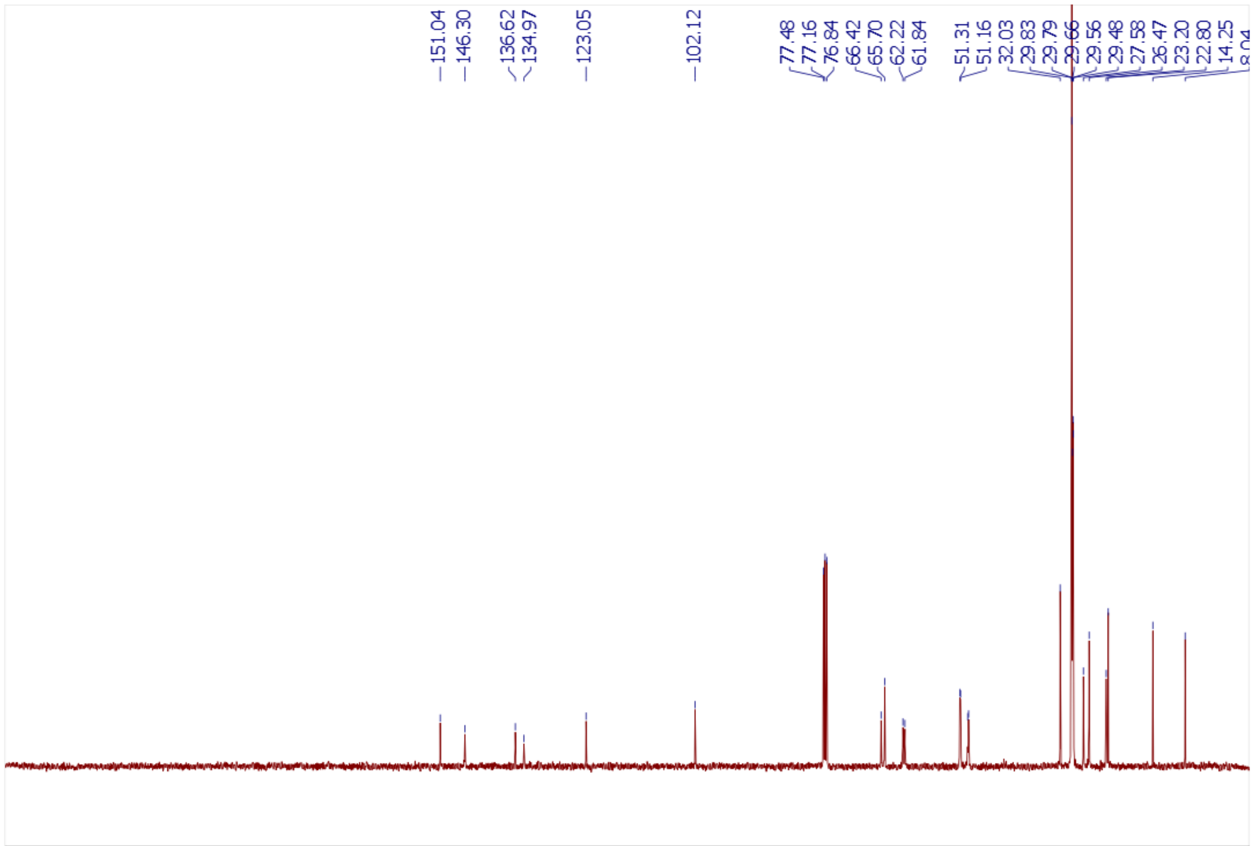

^{13}C{H} NMR spectrum of compound **5c18**

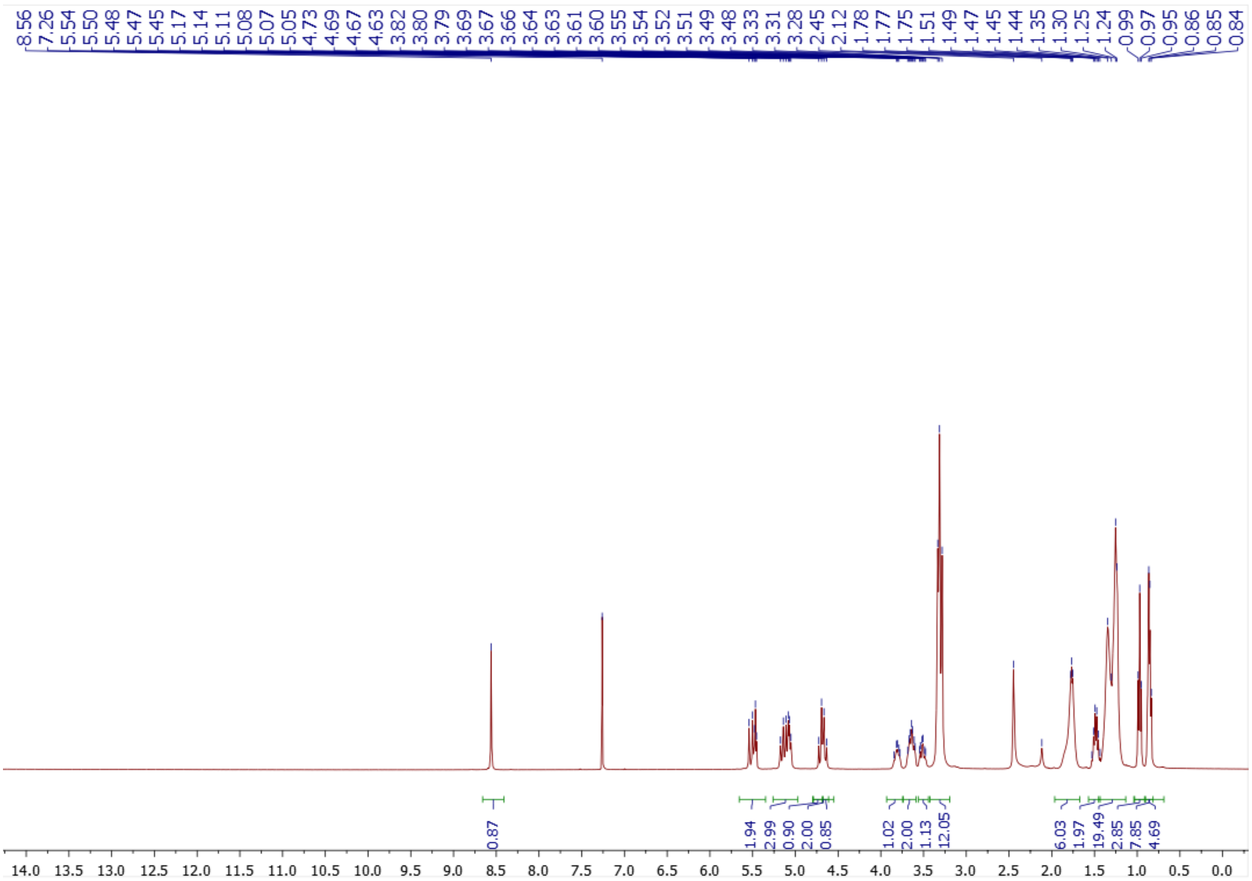

^{1}H NMR spectrum of compound **5d**8

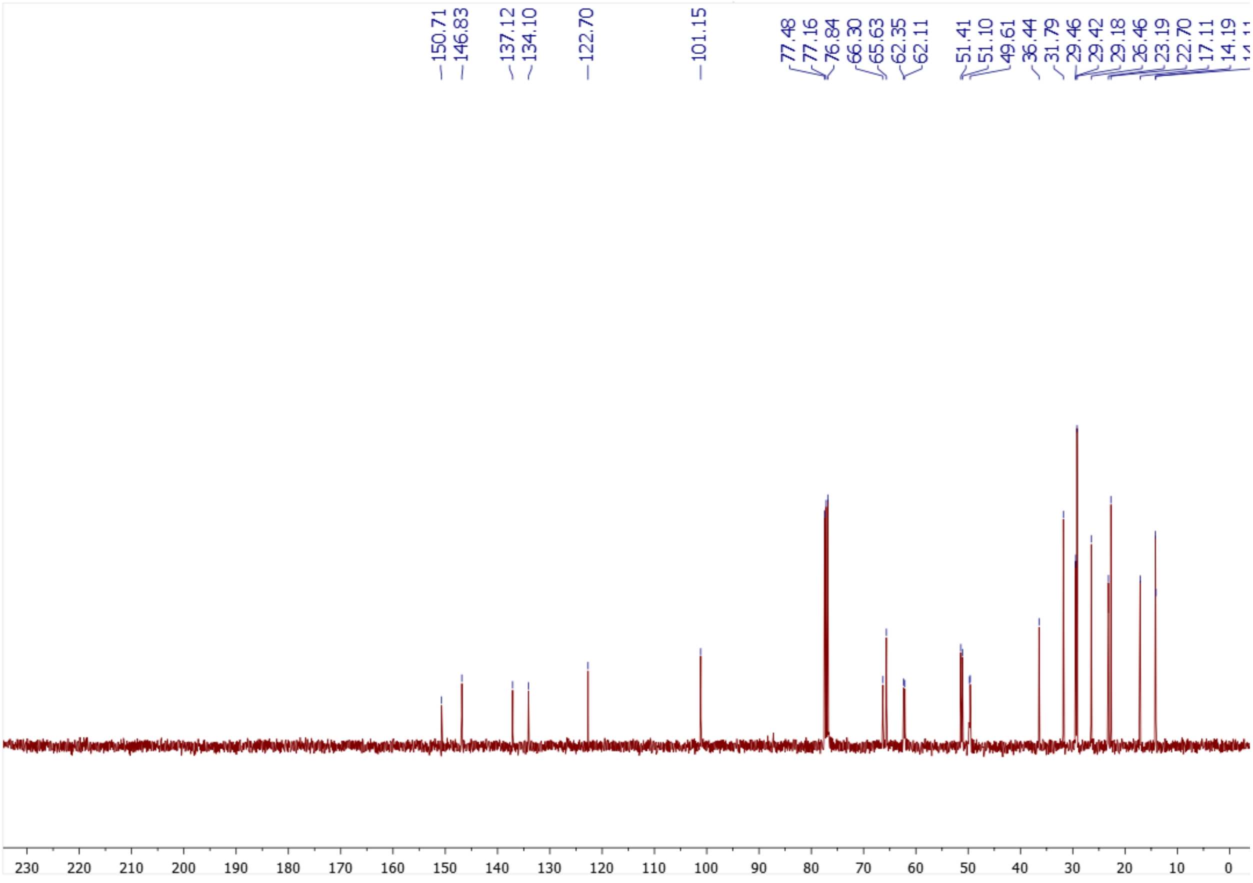

^{13}C{H} NMR spectrum of compound **5d**8

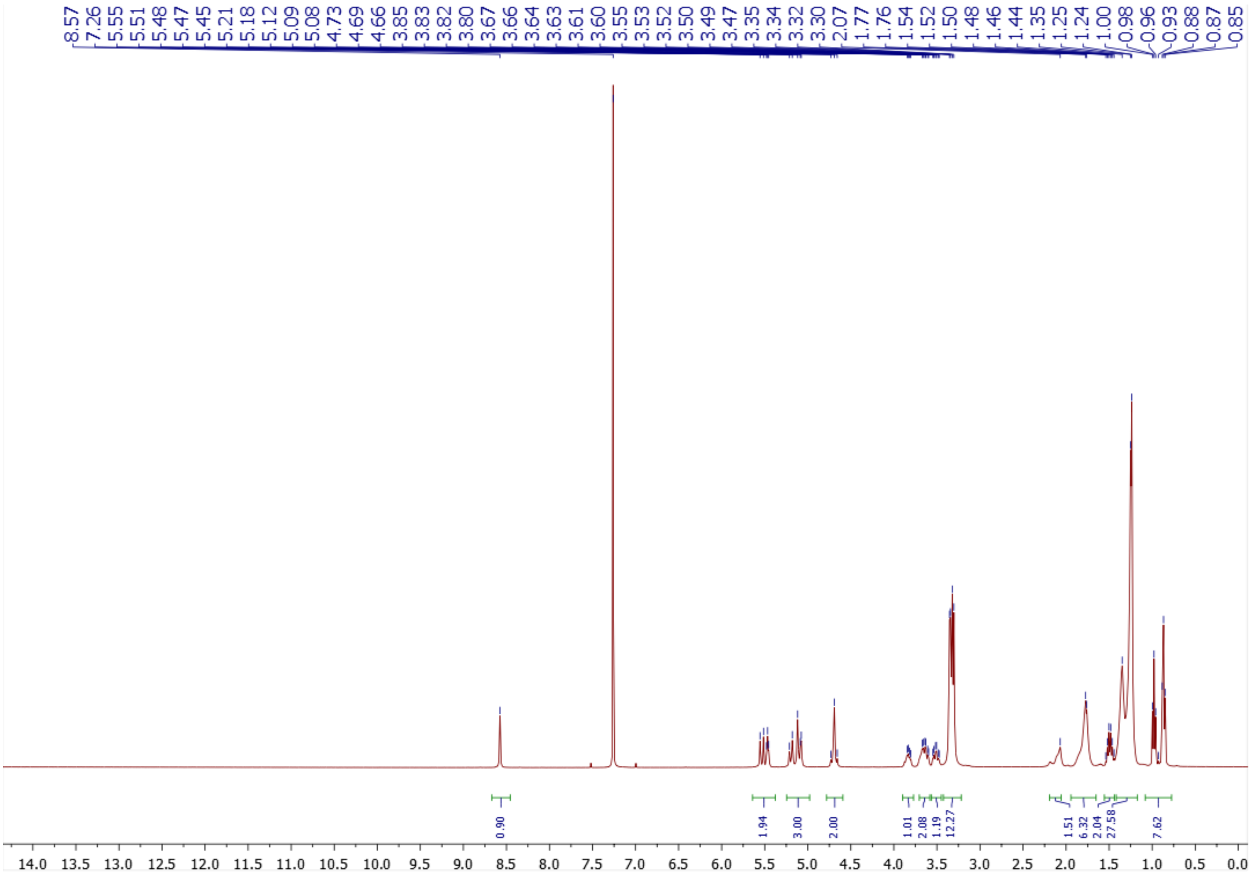

^{1}H NMR spectrum of compound **5d$_{10}$**

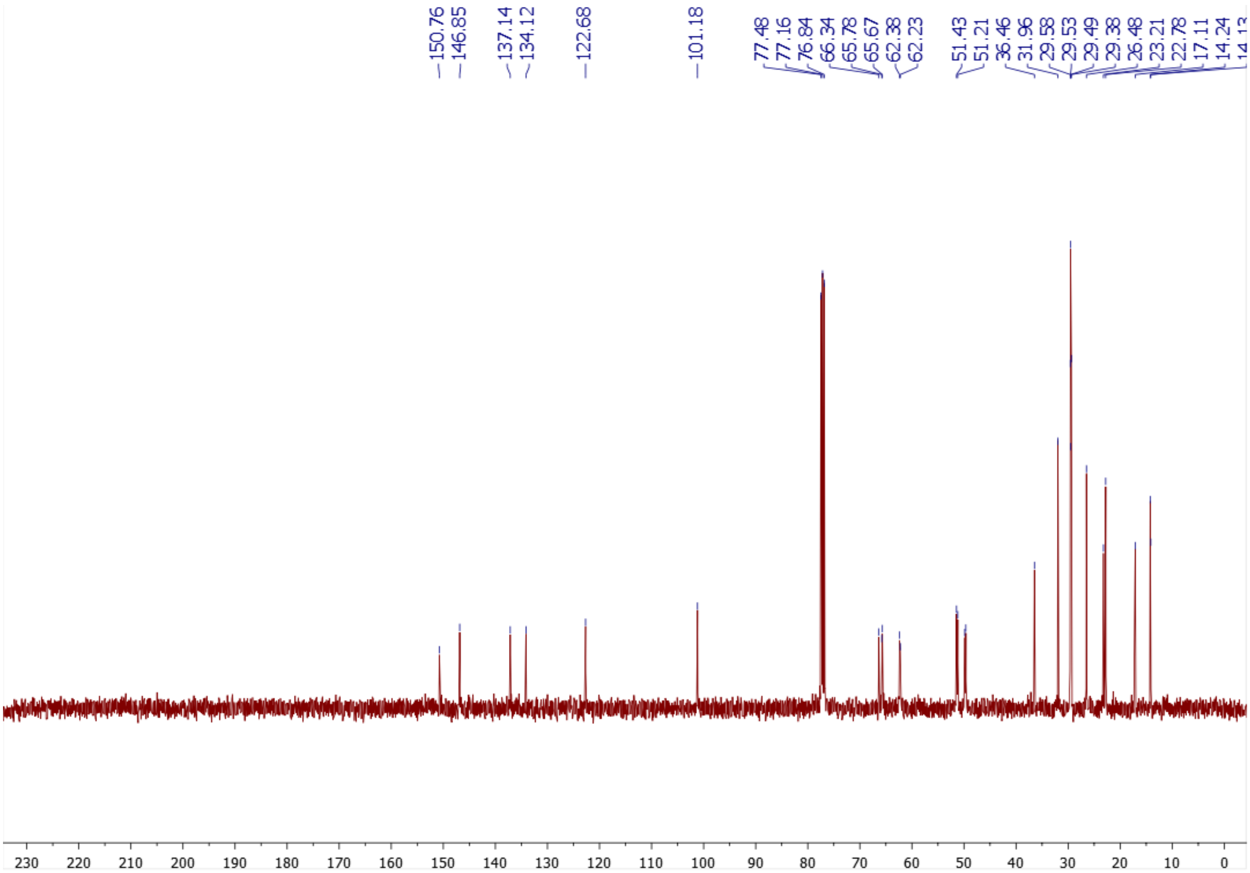

^{13}C{H} NMR spectrum of compound **5d$_{10}$**

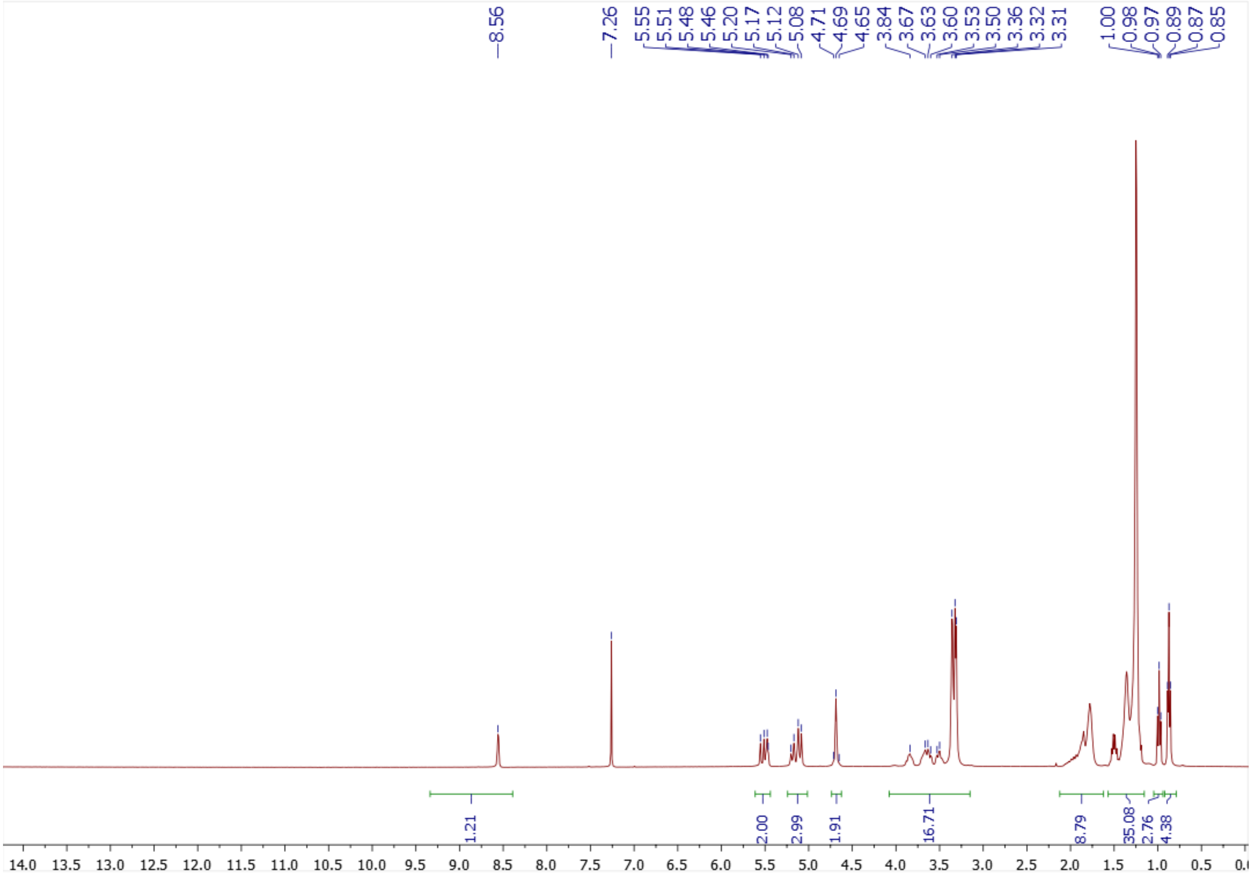

^{1}H NMR spectrum of compound **5d**$_{12}$

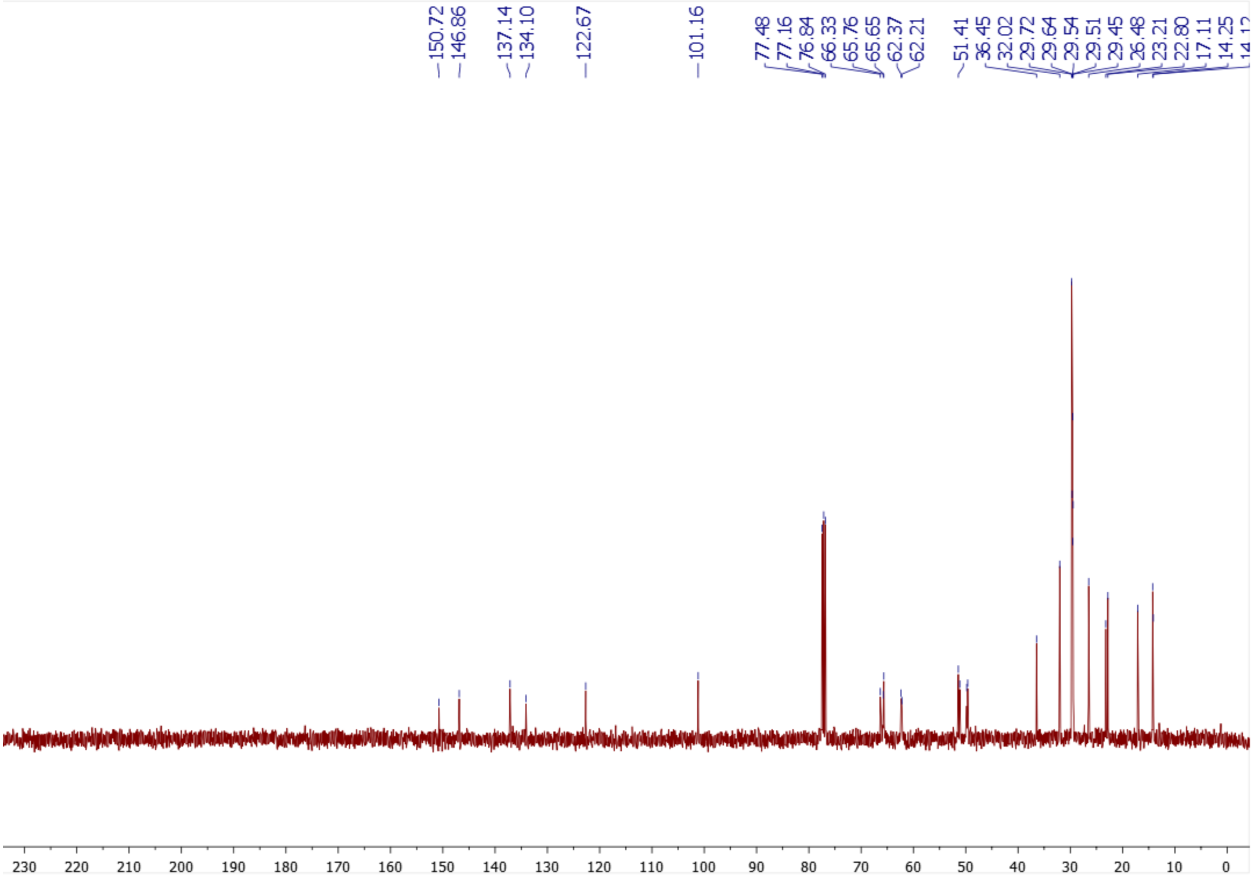

^{13}C{H} NMR spectrum of compound **5d**$_{12}$

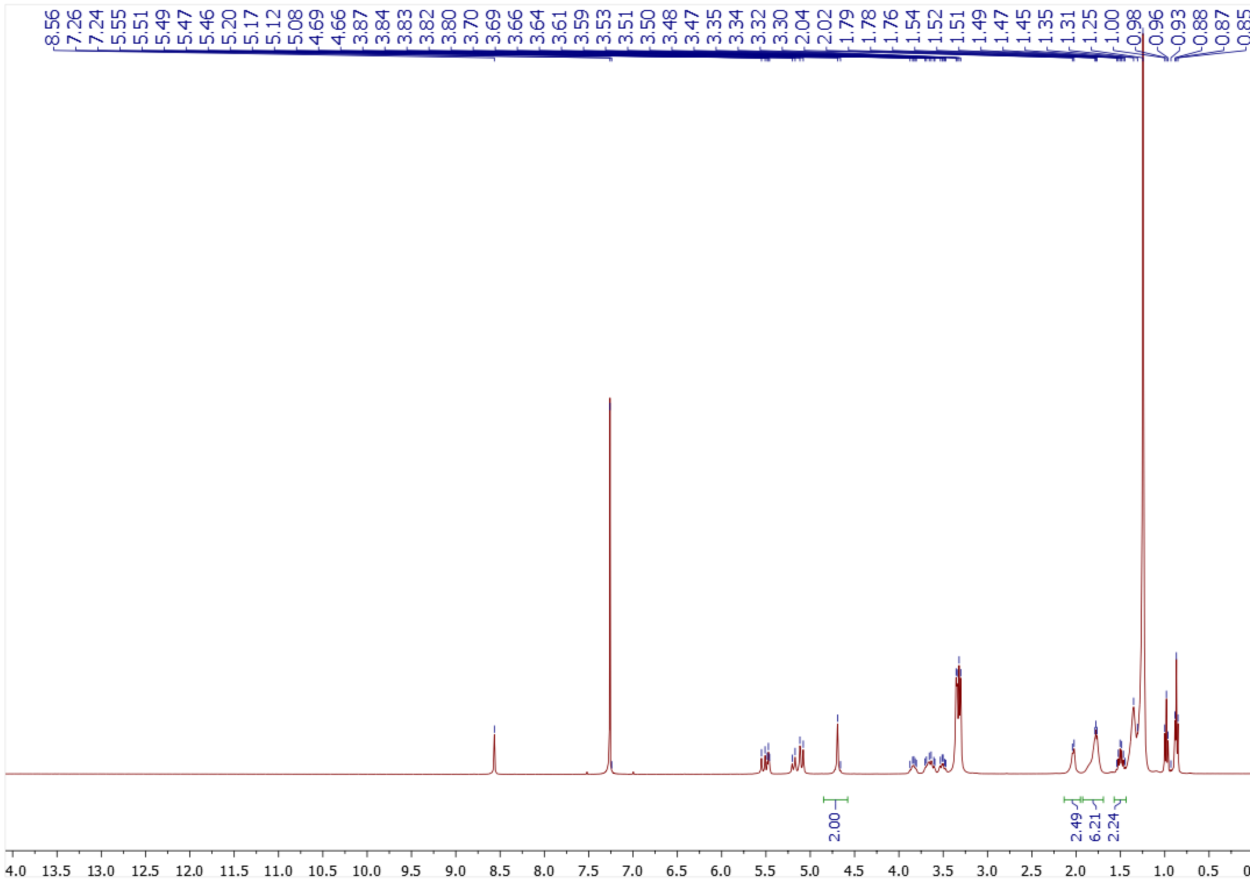

^{1}H NMR spectrum of compound **5d**$_{14}$

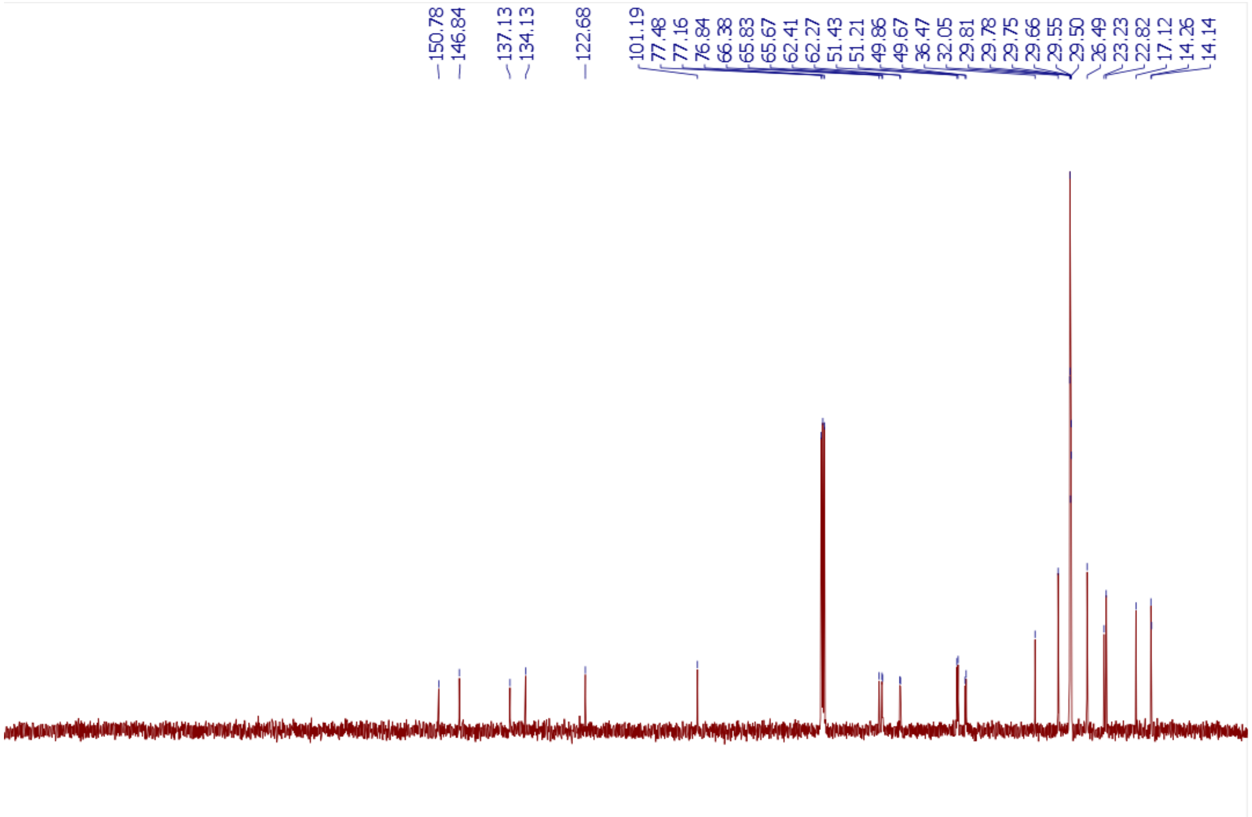

$^{13}C\{H\}$ NMR spectrum of compound **5d**$_{14}$

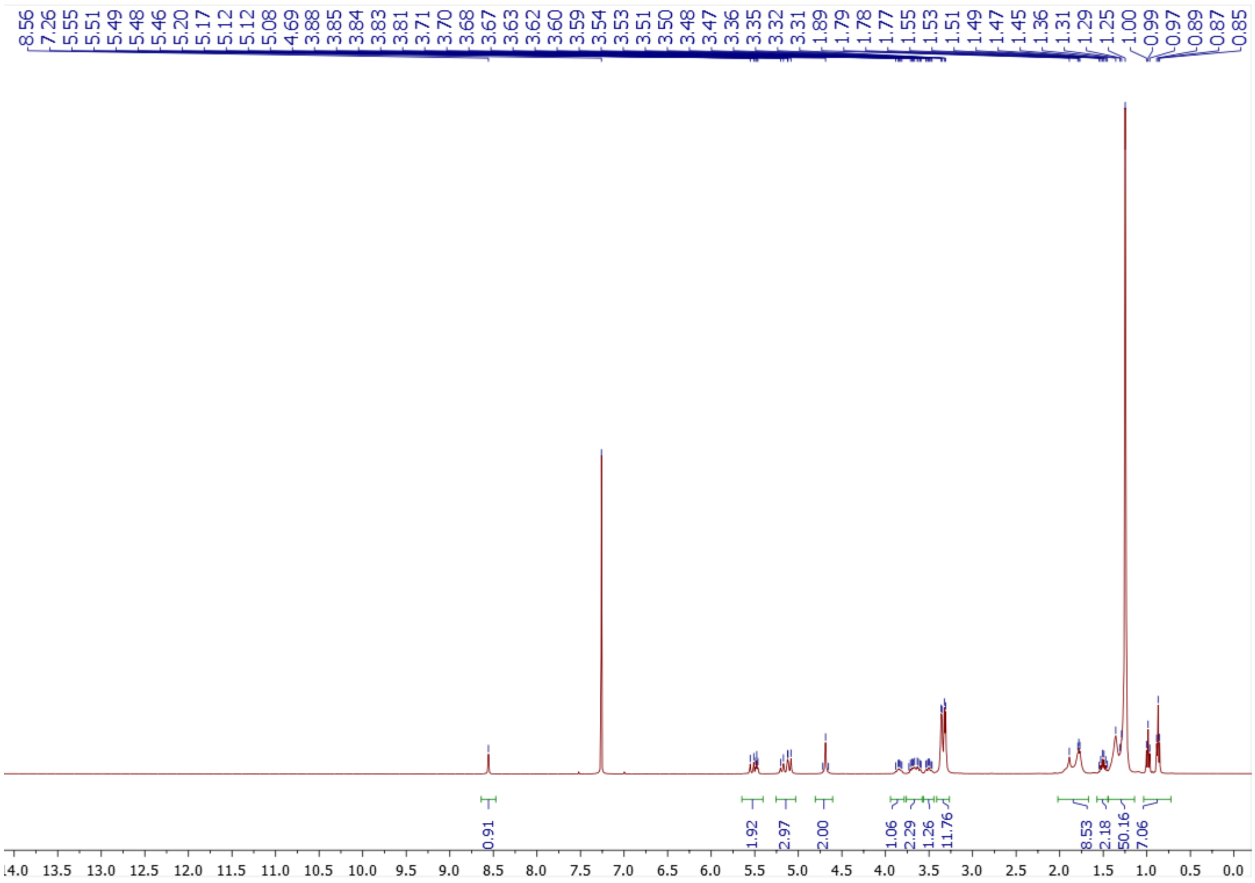

^{1}H NMR spectrum of compound **5d$_{16}$**

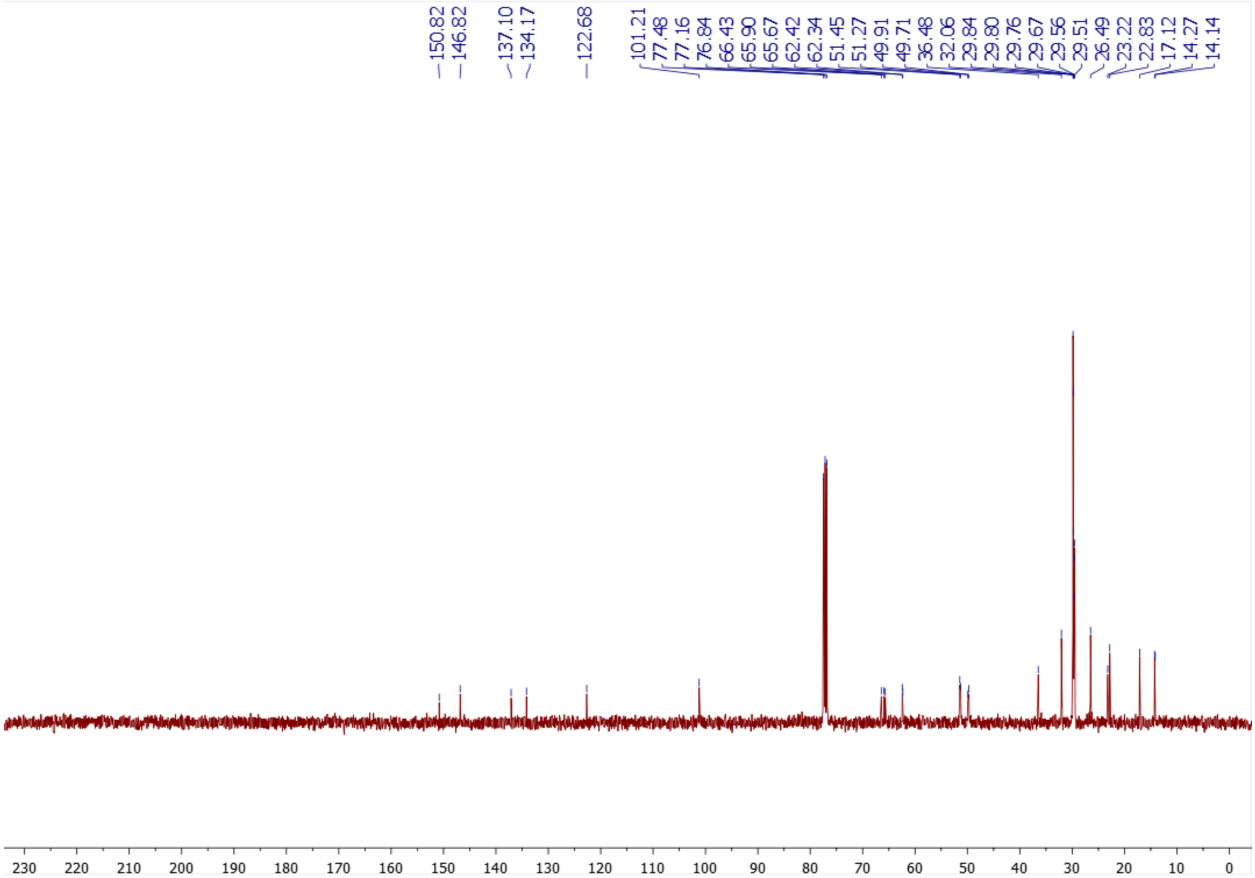

^{13}C{H} NMR spectrum of compound **5d$_{16}$**

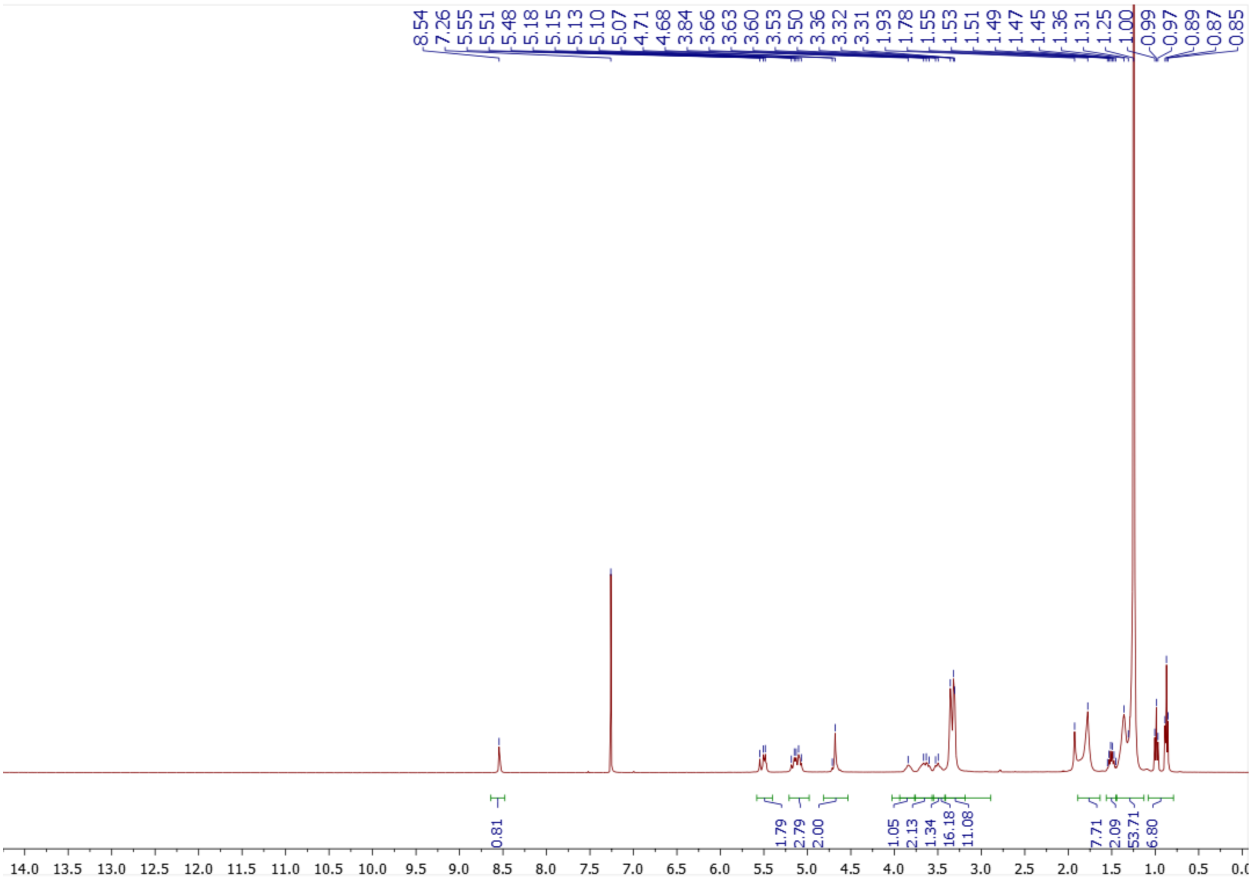

^{1}H NMR spectrum of compound **5d₁₈**

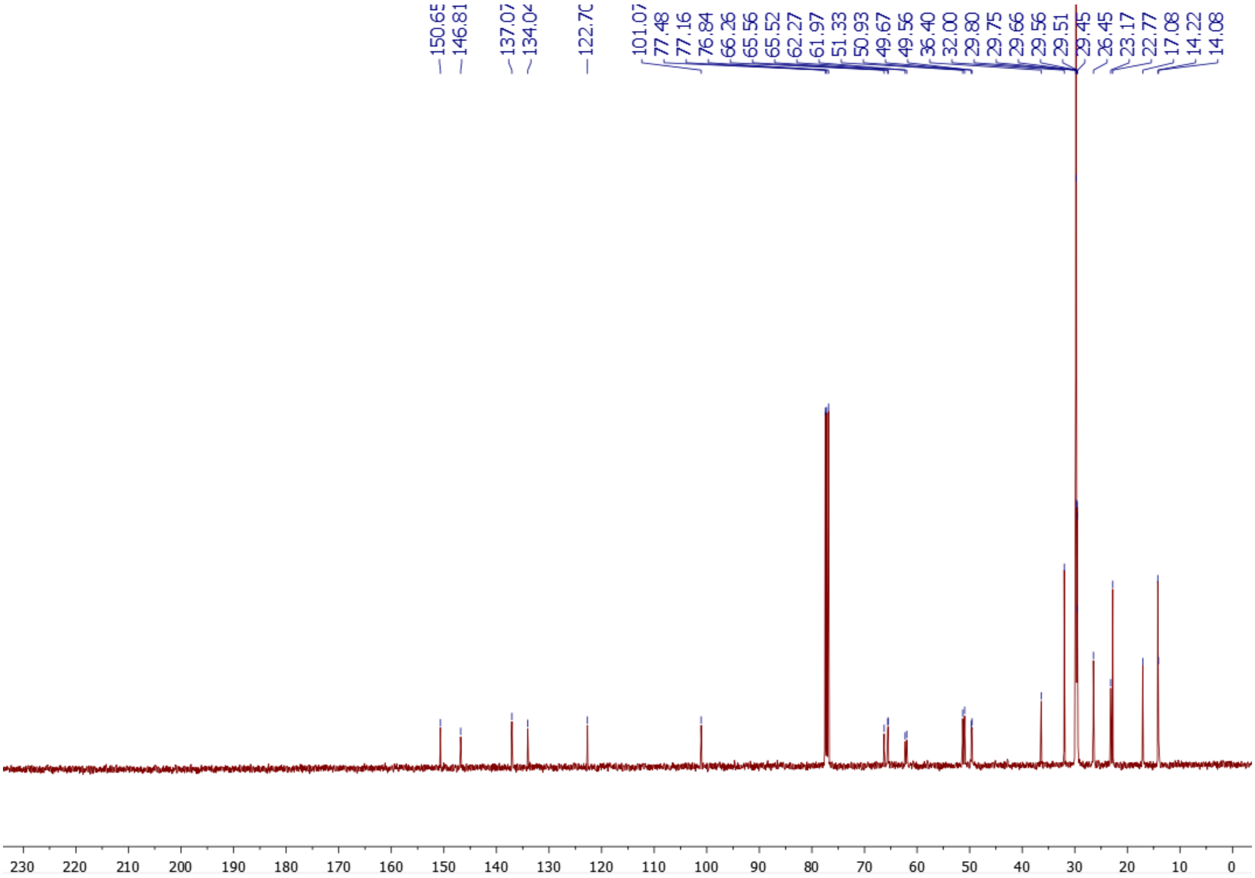

^{13}C{H} NMR spectrum of compound **5d₁₈**

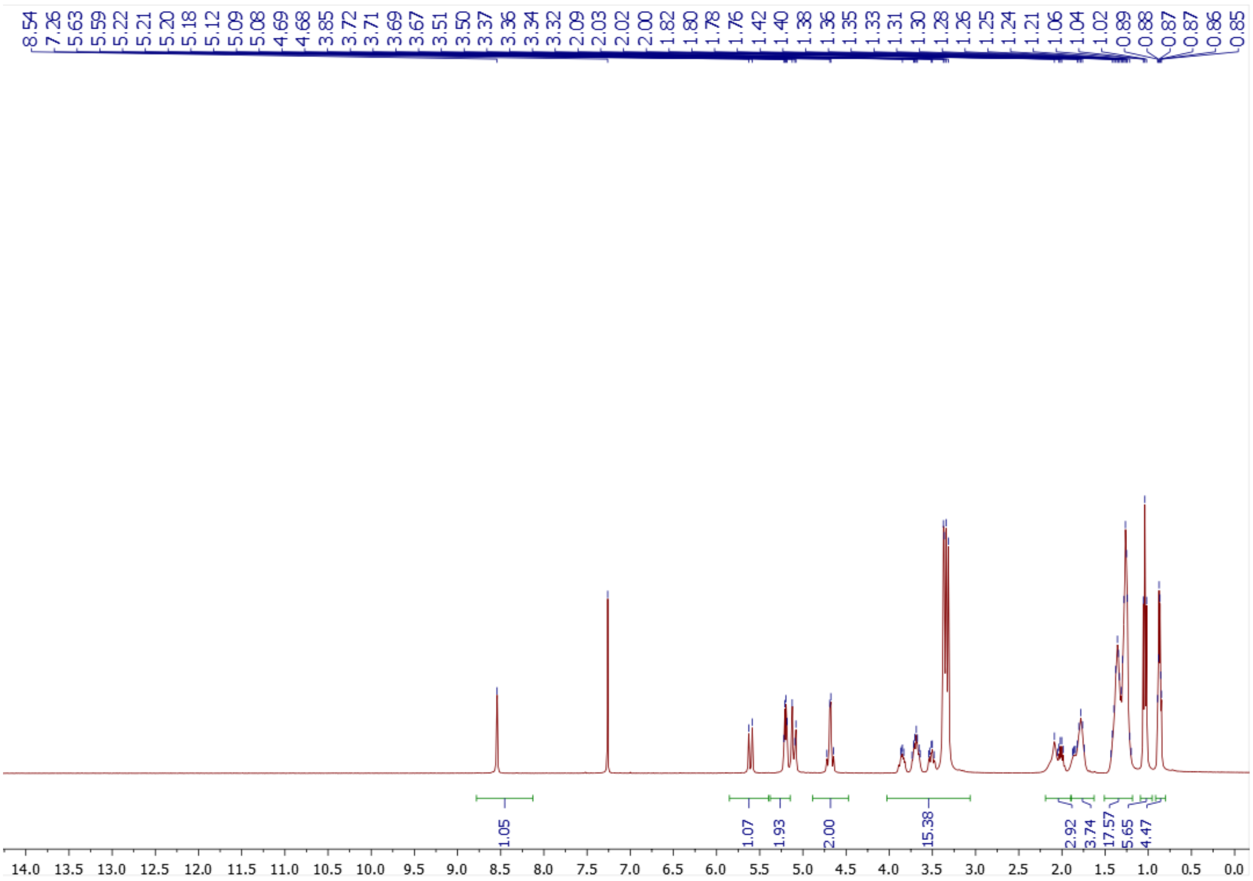

^{1}H NMR spectrum of compound **5e**8

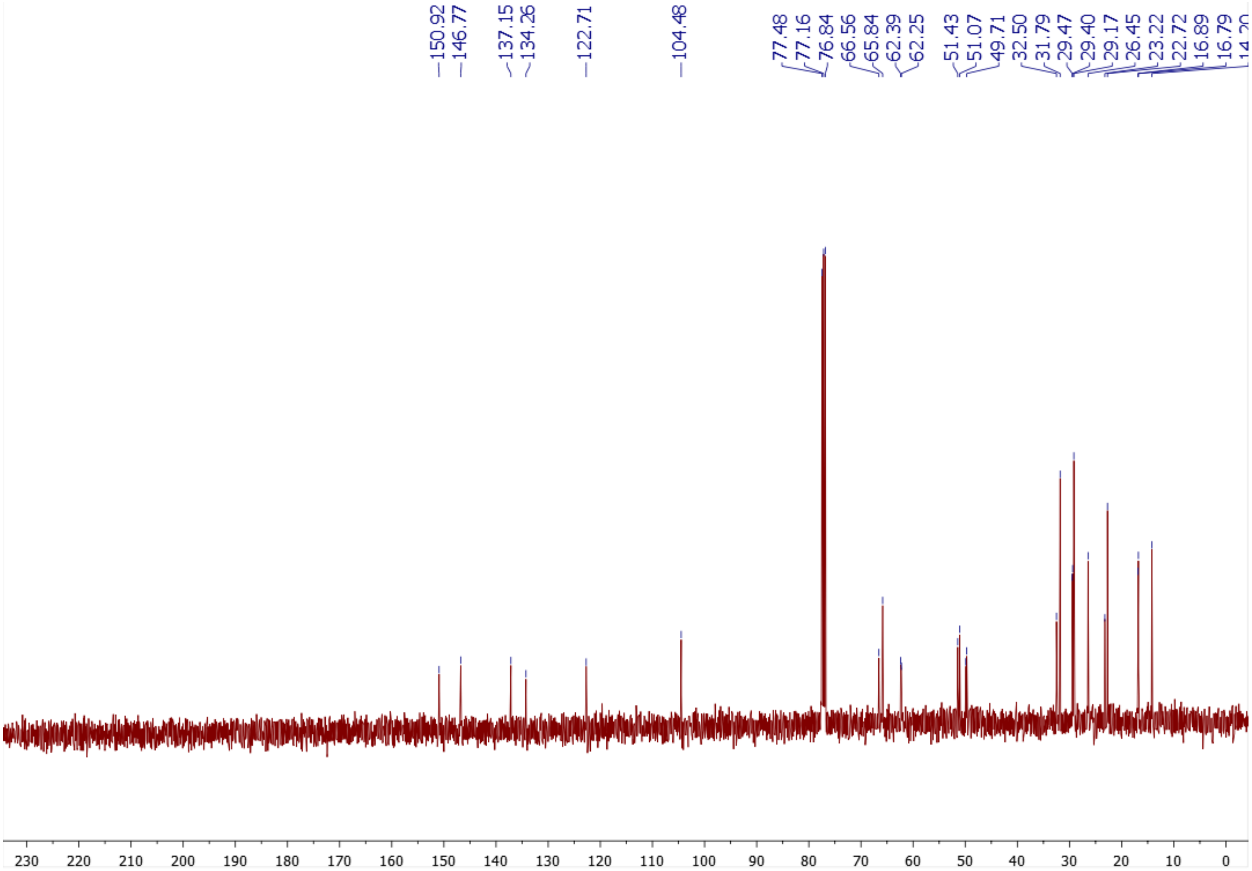

^{13}C{H} NMR spectrum of compound **5e**8

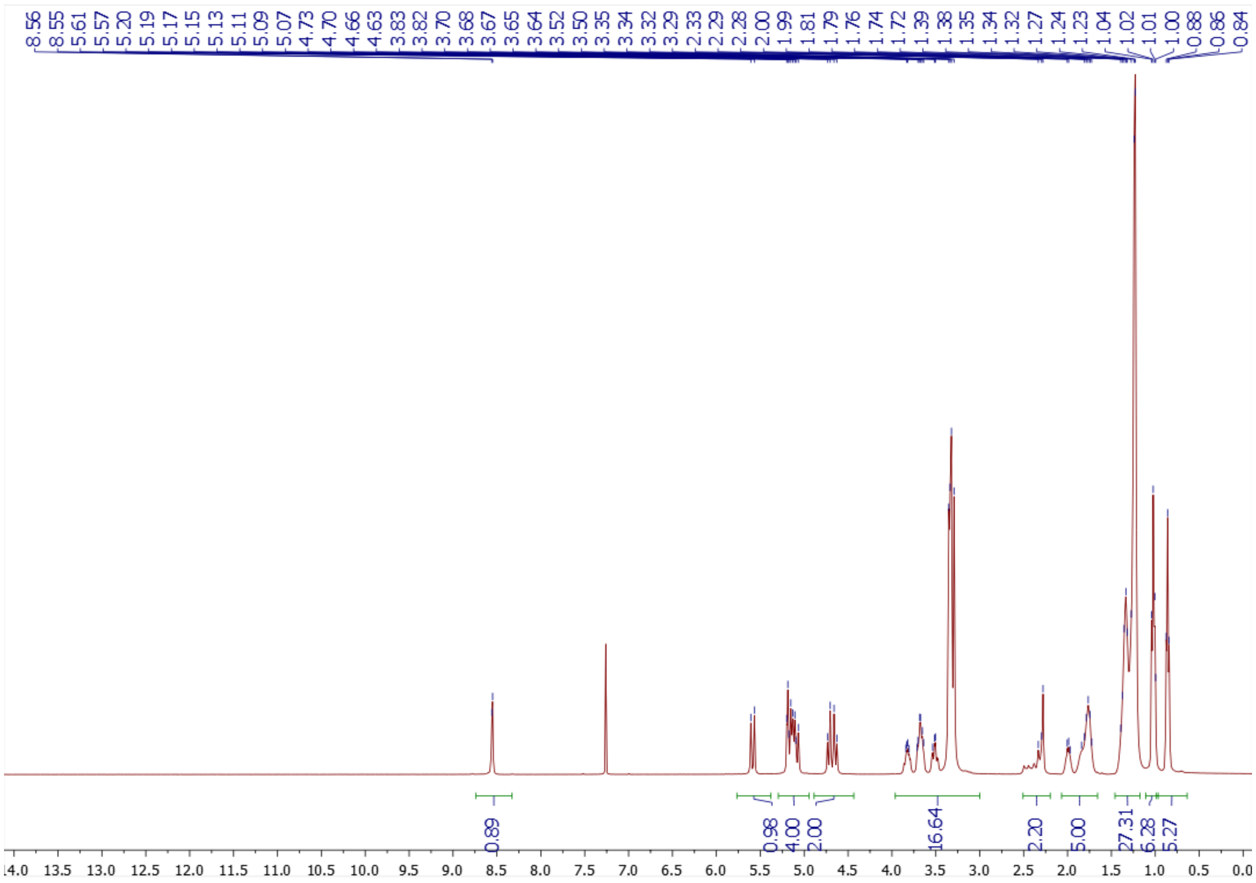

^{1}H NMR spectrum of compound **5e**$_{10}$

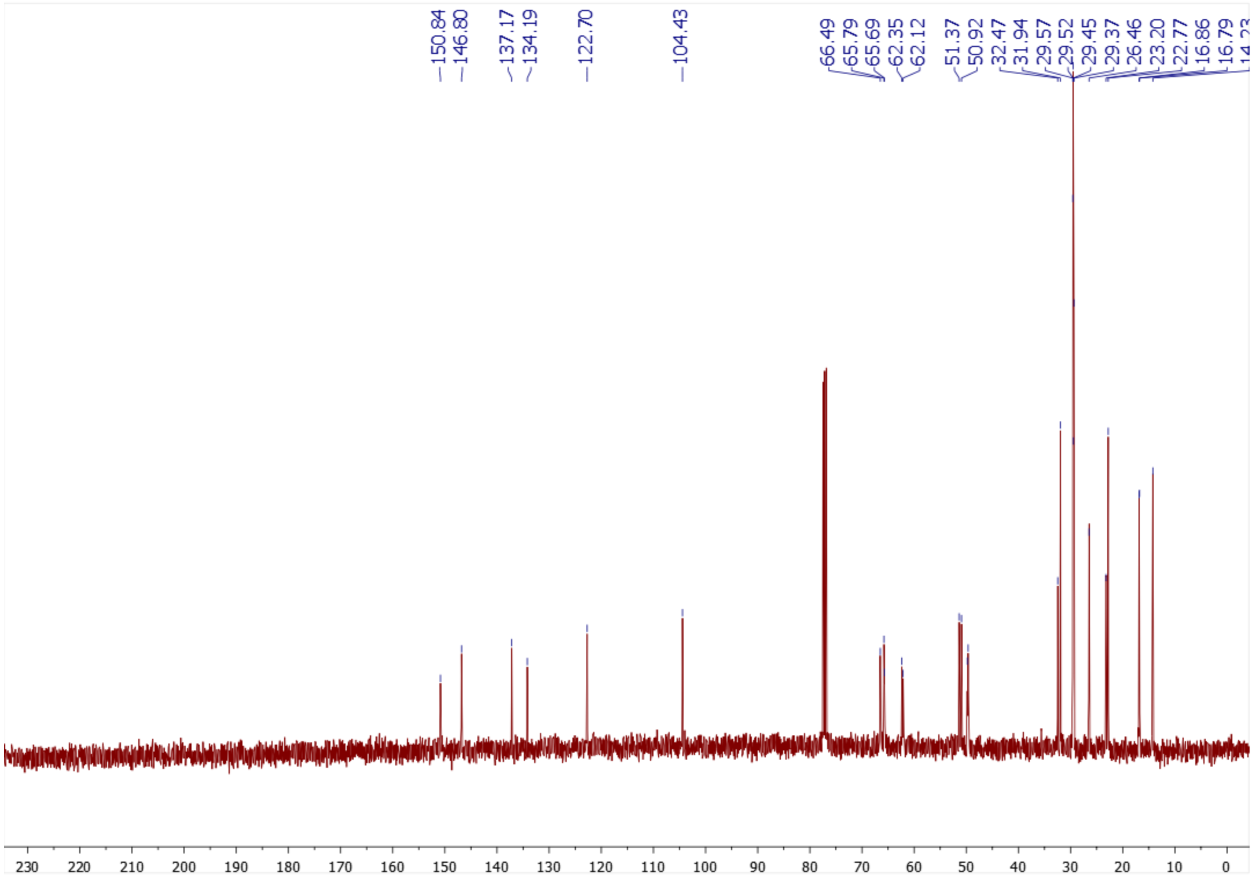

^{13}C{H} NMR spectrum of compound **5e**$_{10}$

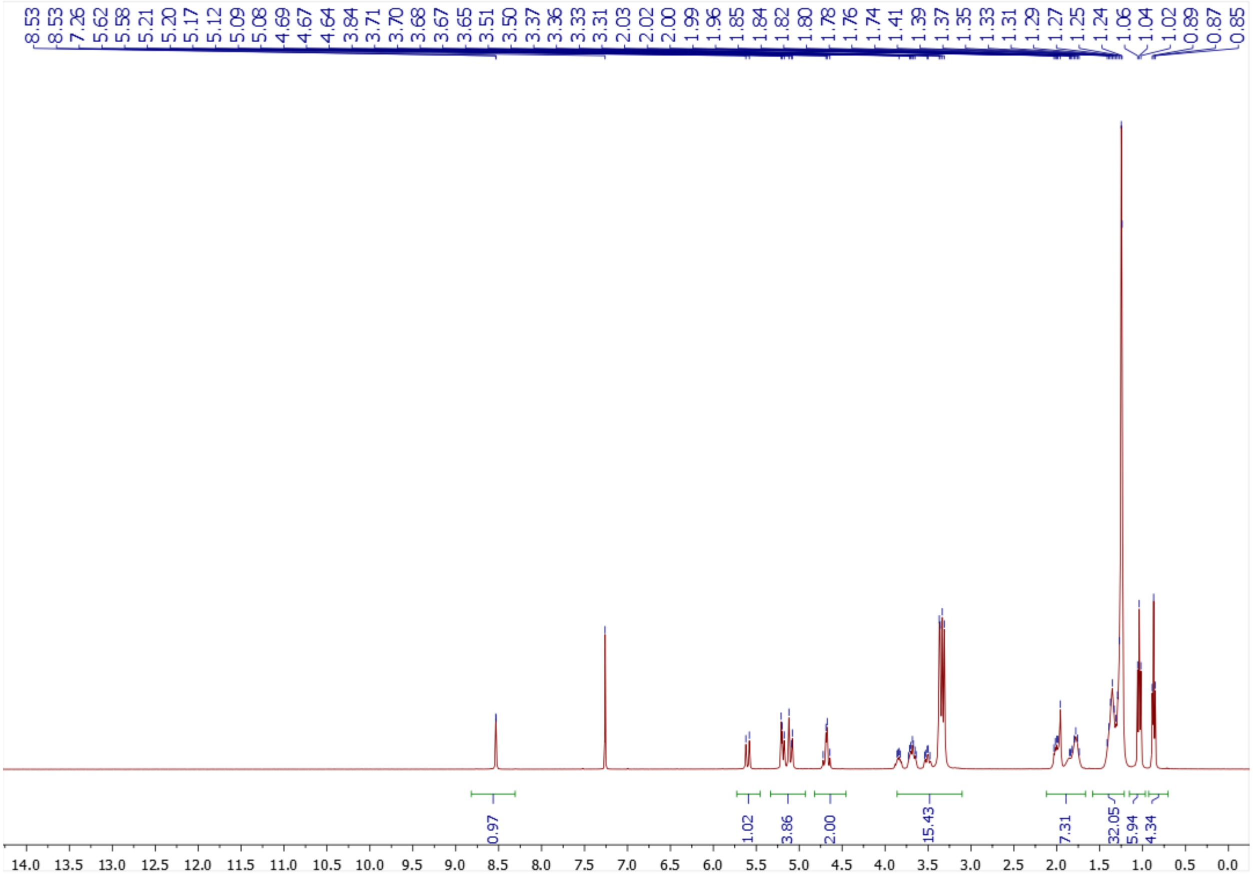

^{1}H NMR spectrum of compound **5e**$_{12}$

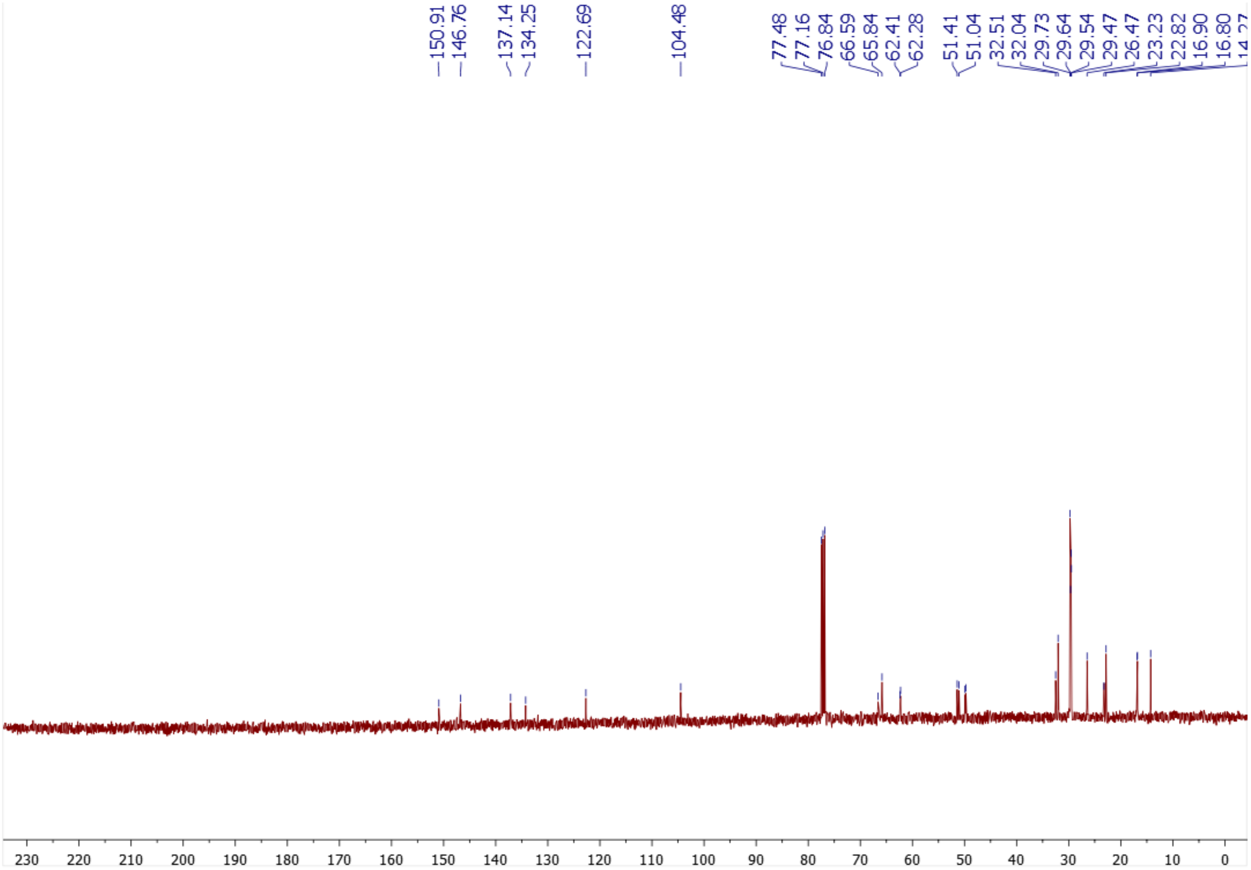

^{13}C{H} NMR spectrum of compound **5e**$_{12}$

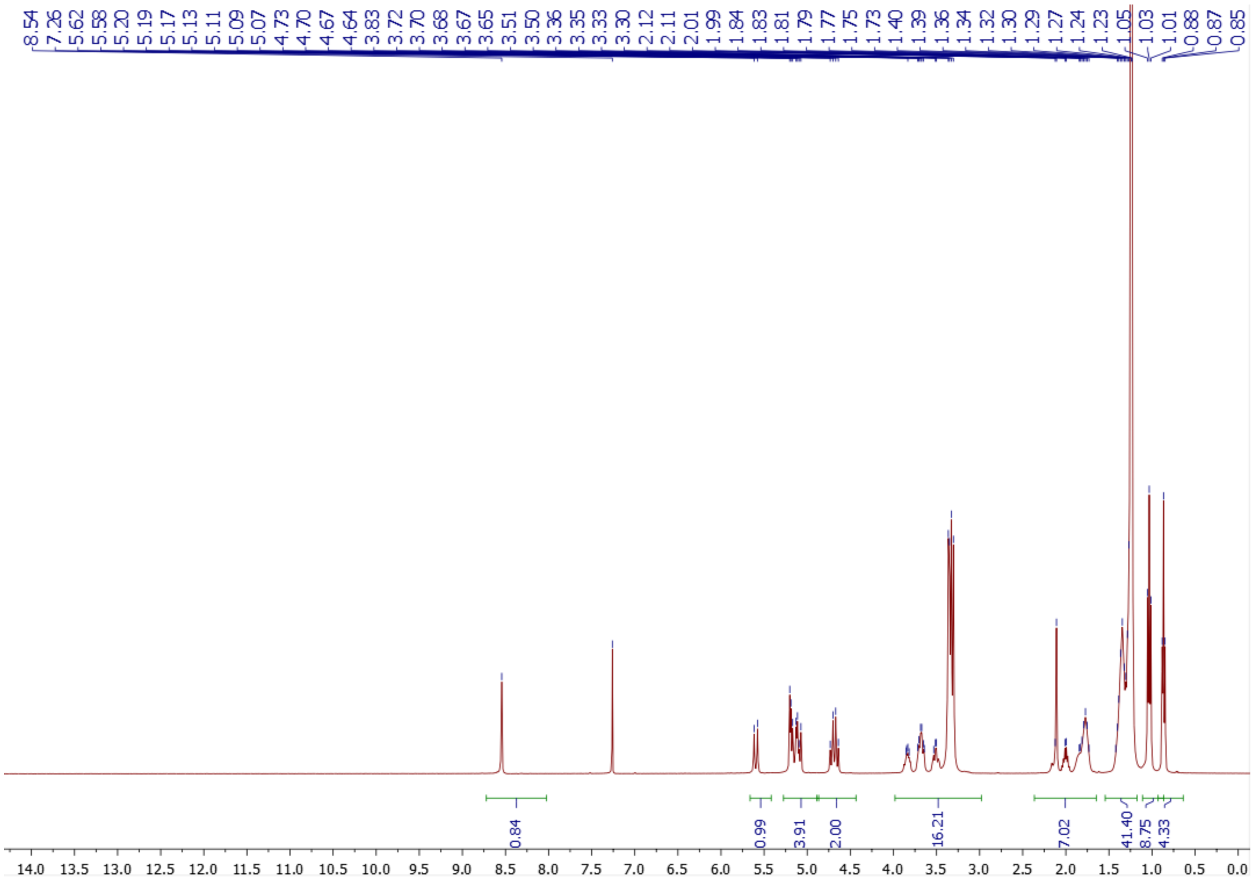

^{1}H NMR spectrum of compound **5e14**

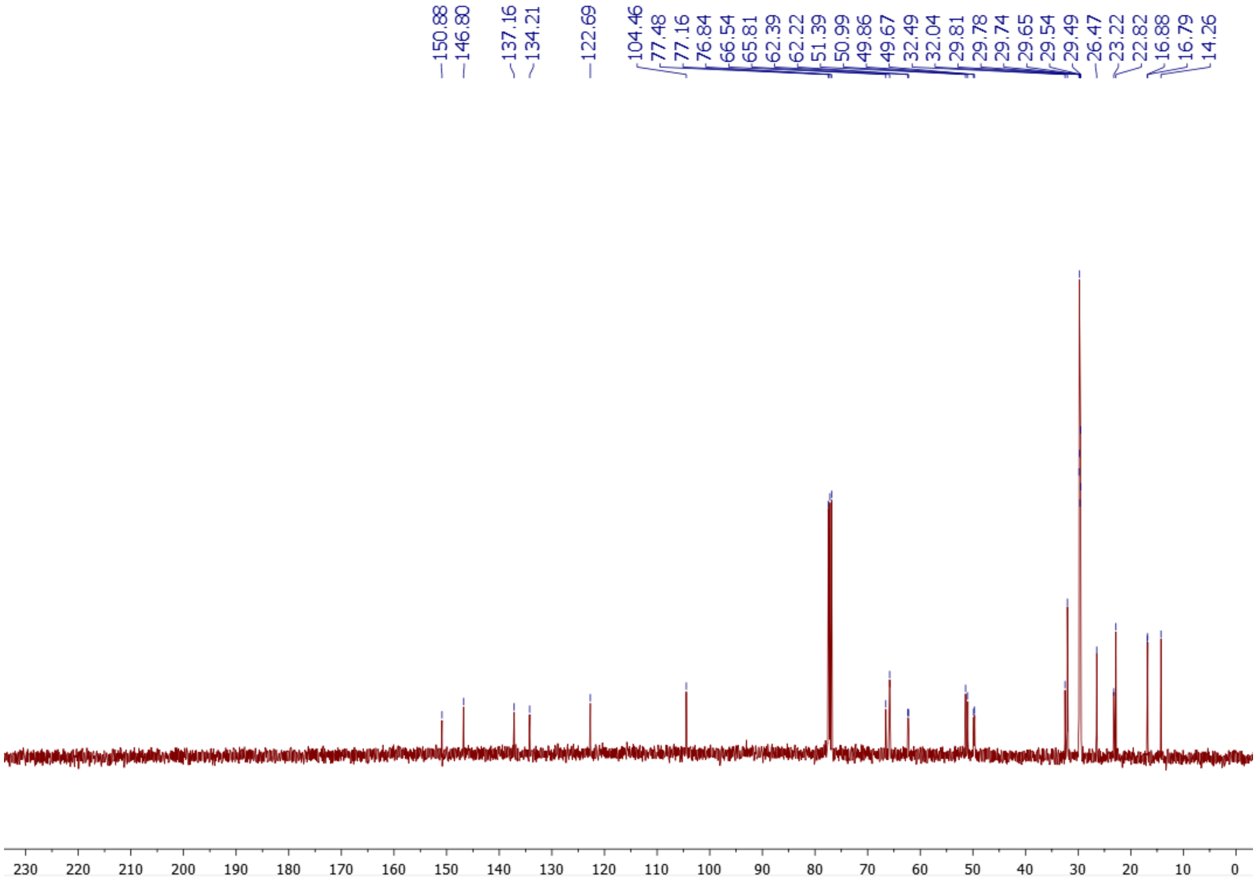

^{13}C{H} NMR spectrum of compound **5e14**

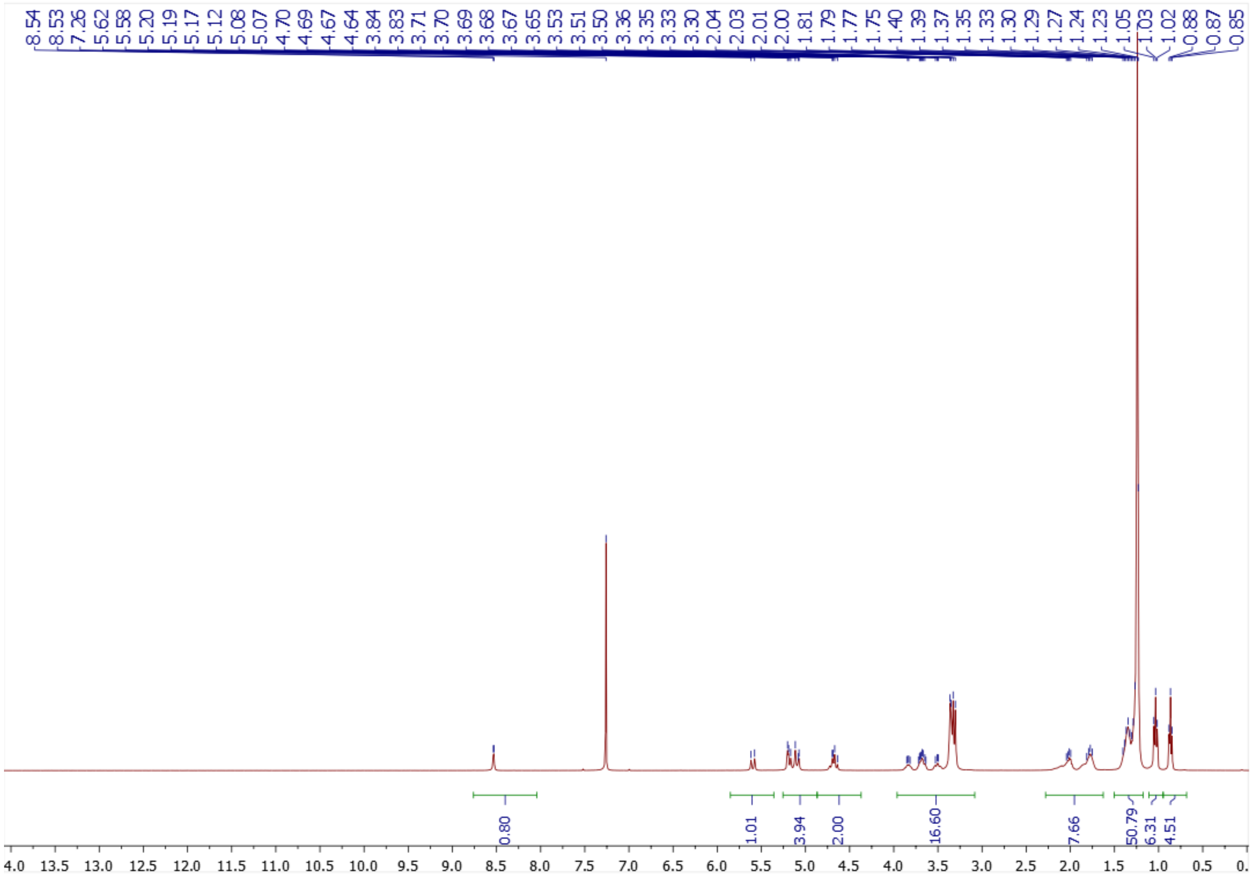

^{1}H NMR spectrum of compound **5e**16

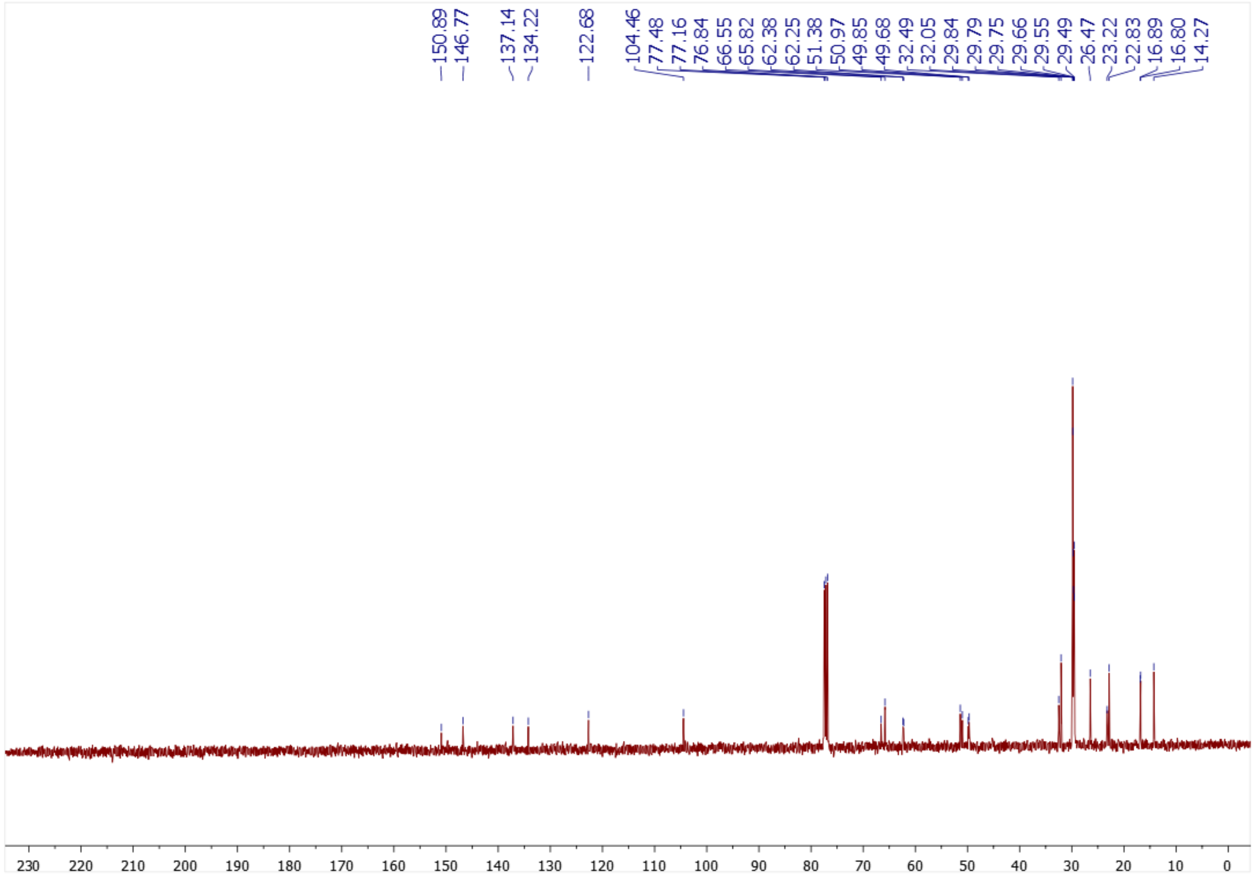

^{13}C{H} NMR spectrum of compound **5e**16

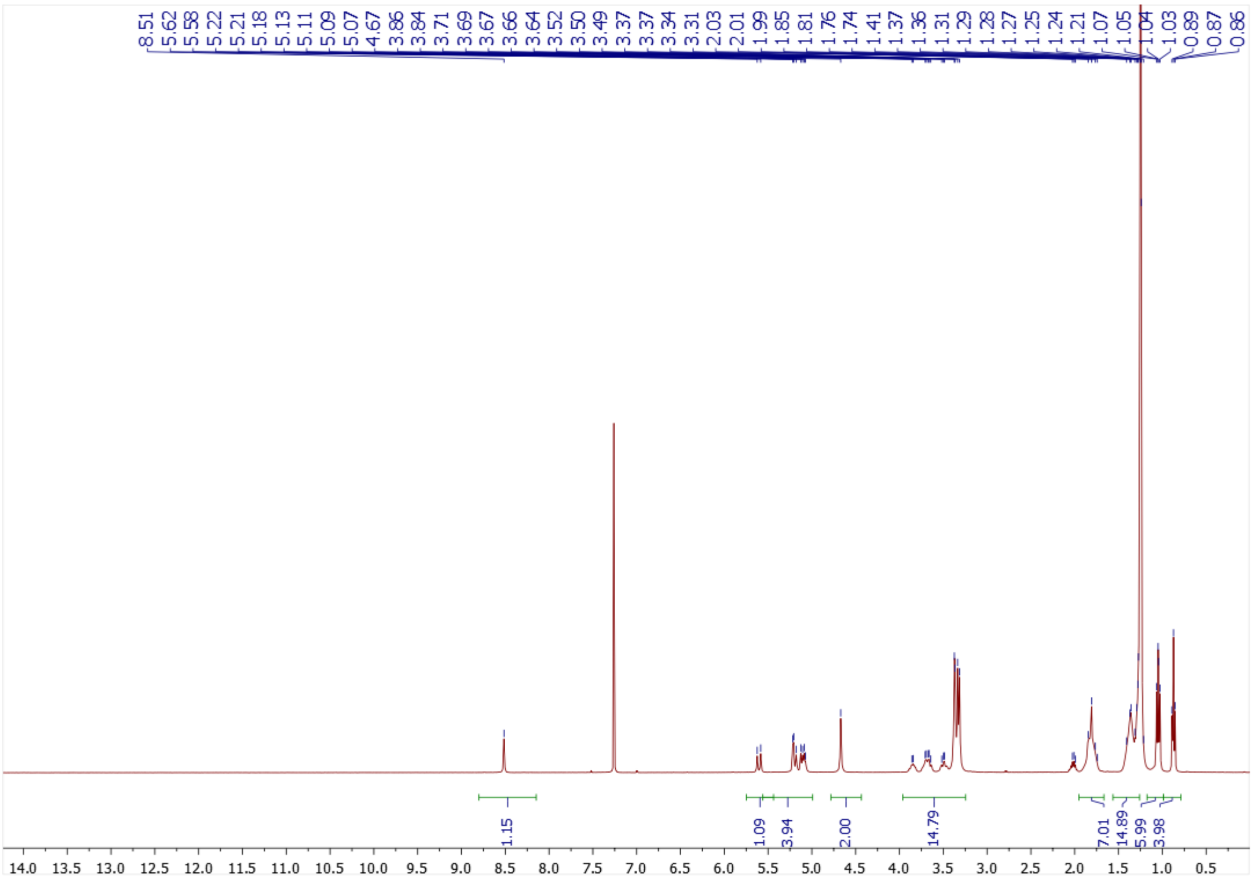

^{1}H NMR spectrum of compound **5e**18

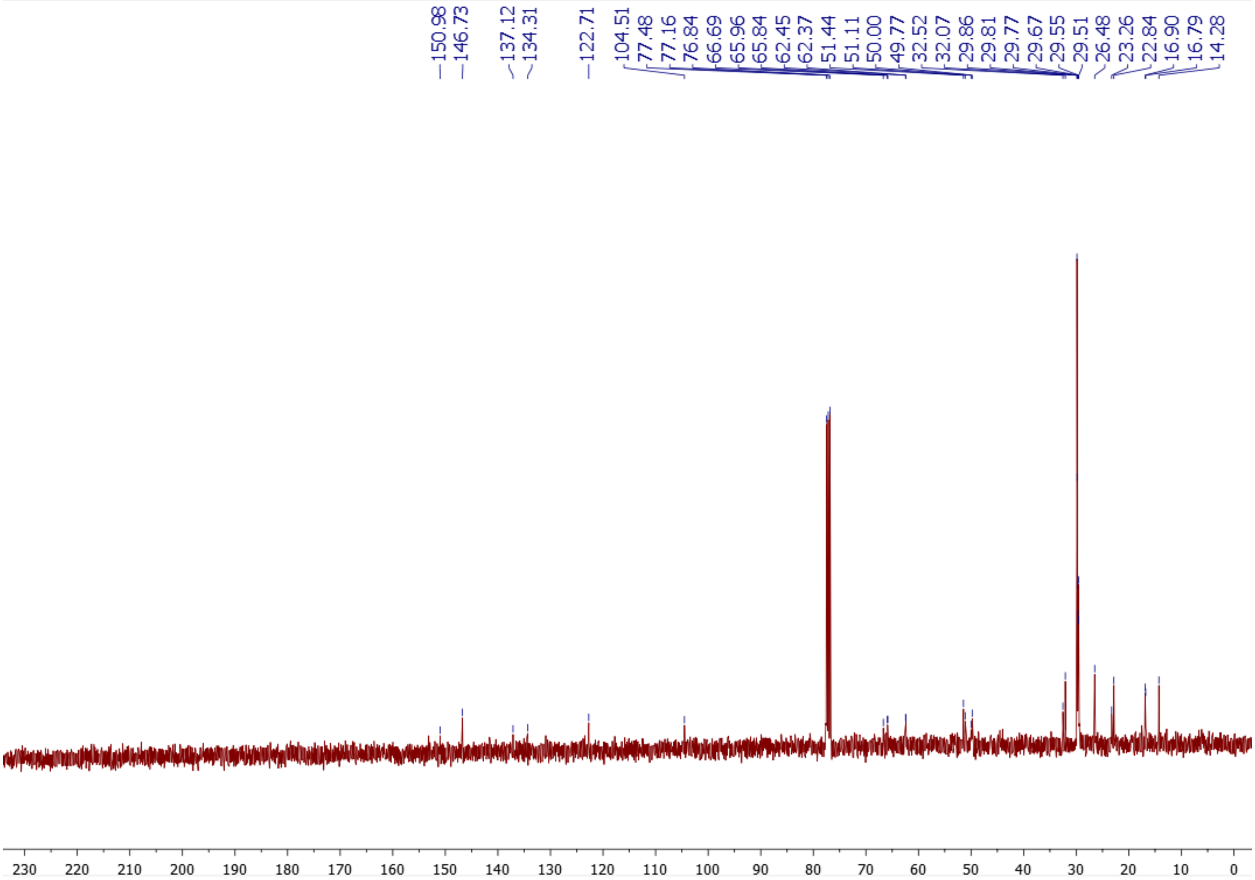

^{13}C{H} NMR spectrum of compound **5e**18

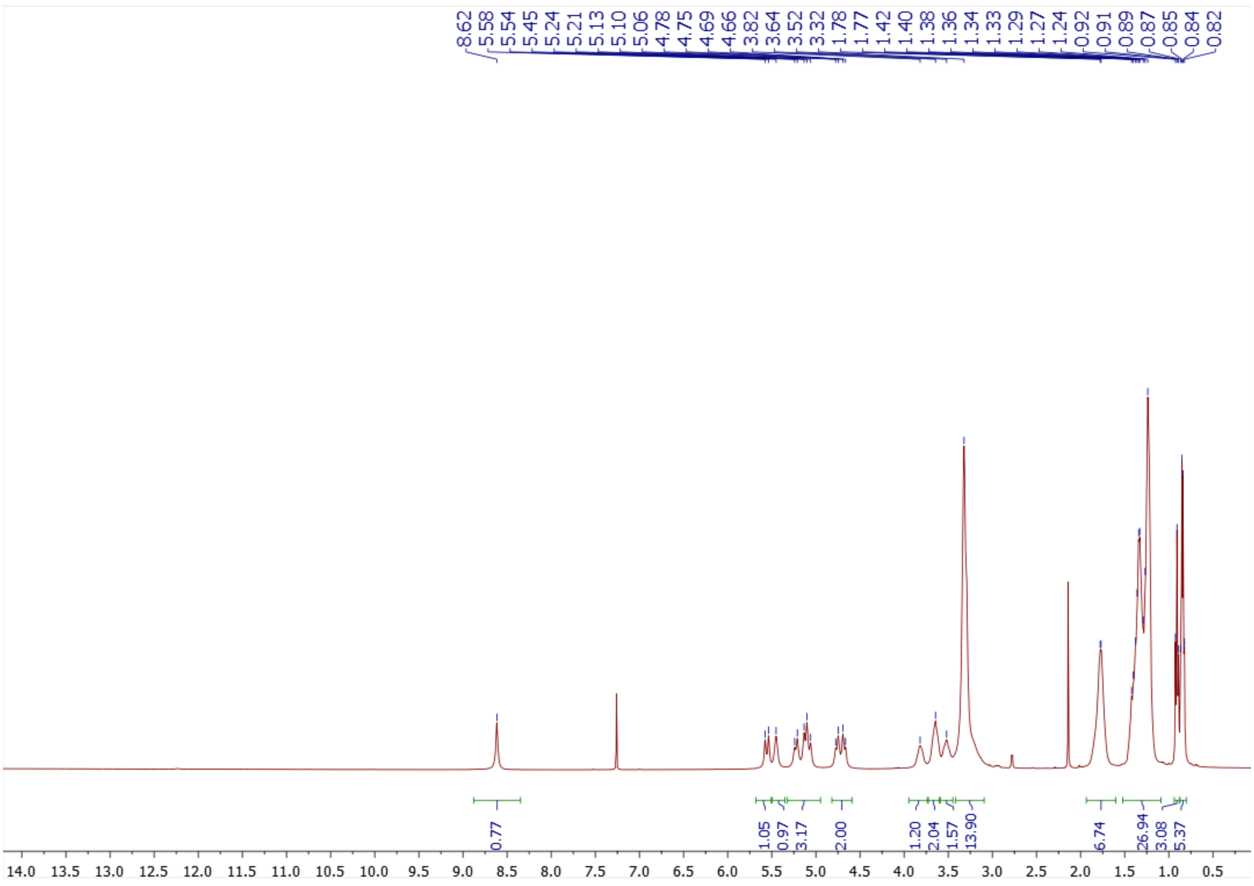

^{1}H NMR spectrum of compound **5f$_8$**

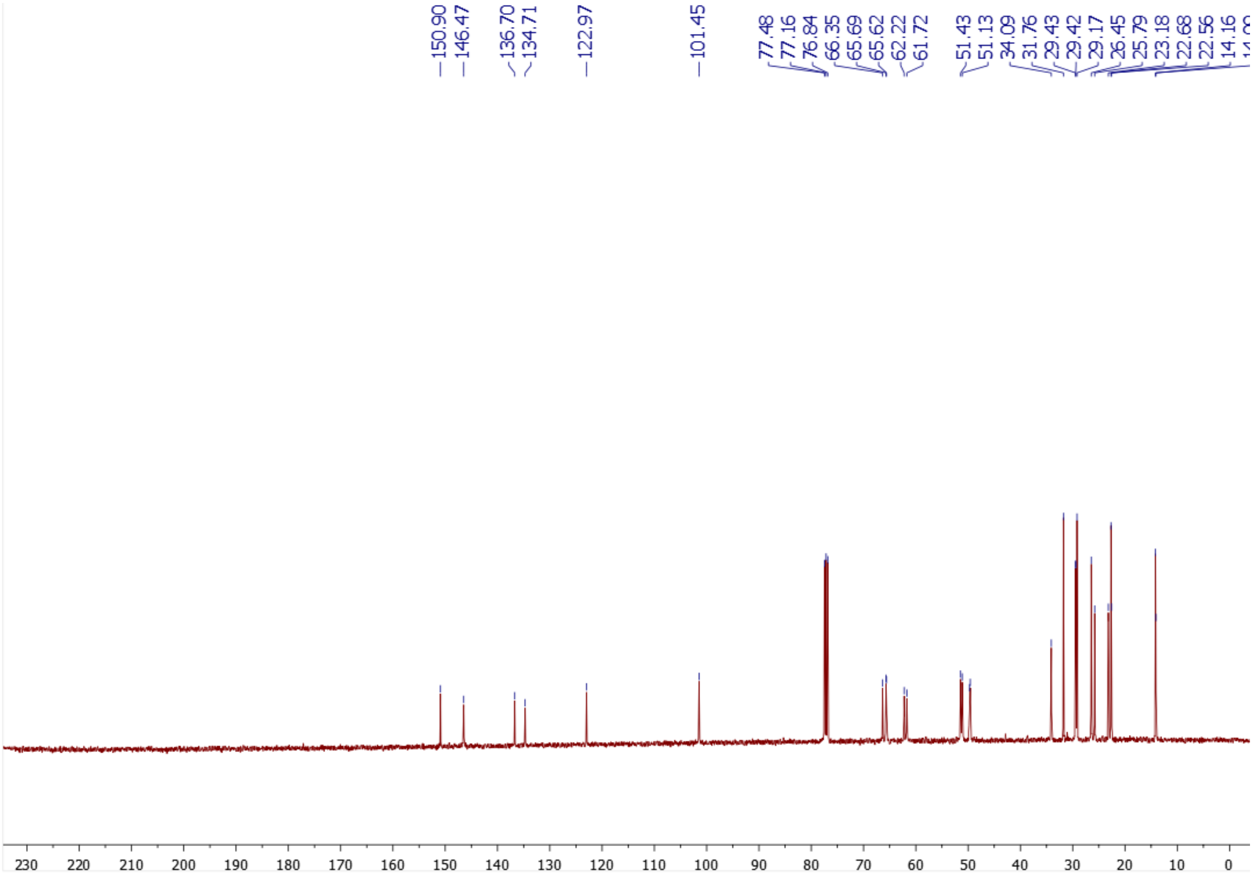

^{13}C{H} NMR spectrum of compound **5f$_8$**

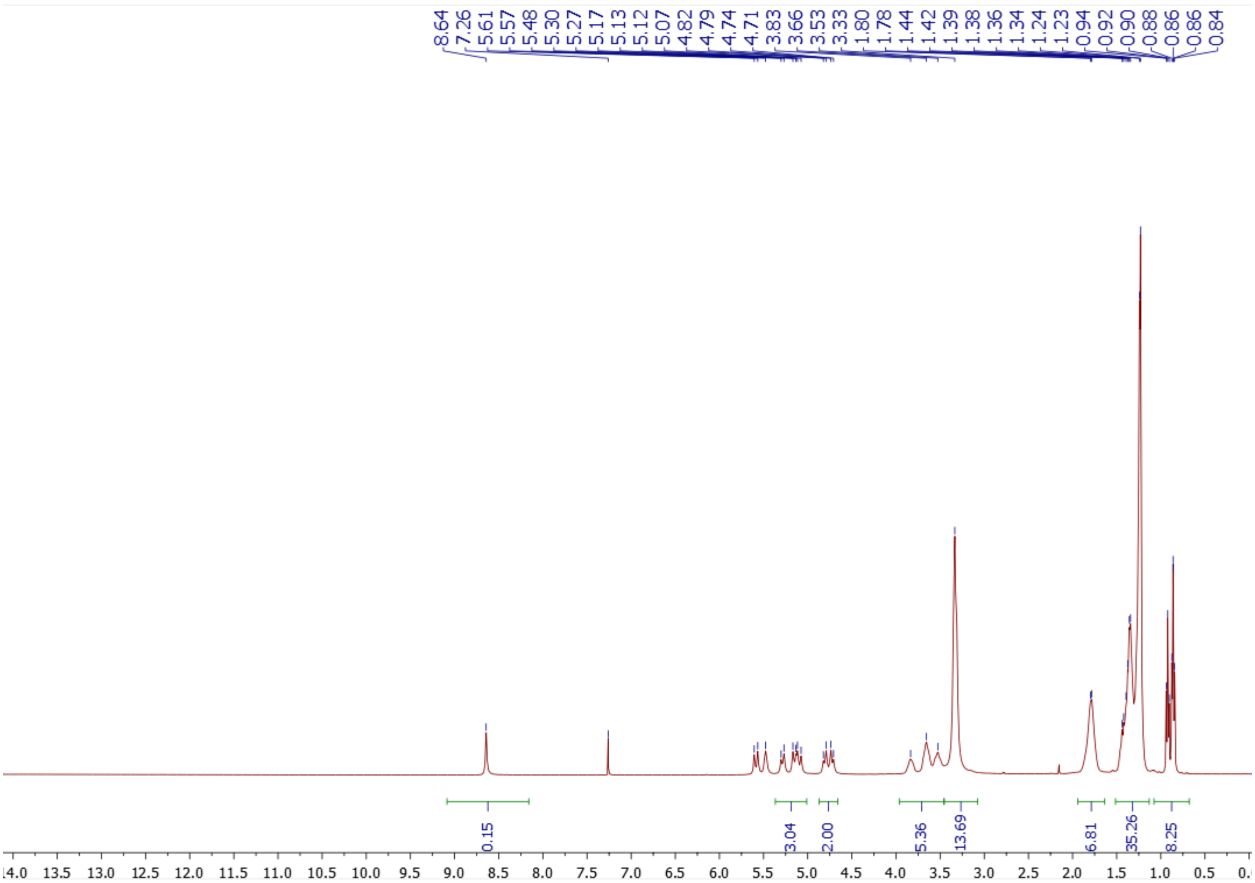

^{1}H NMR spectrum of compound **5f10**

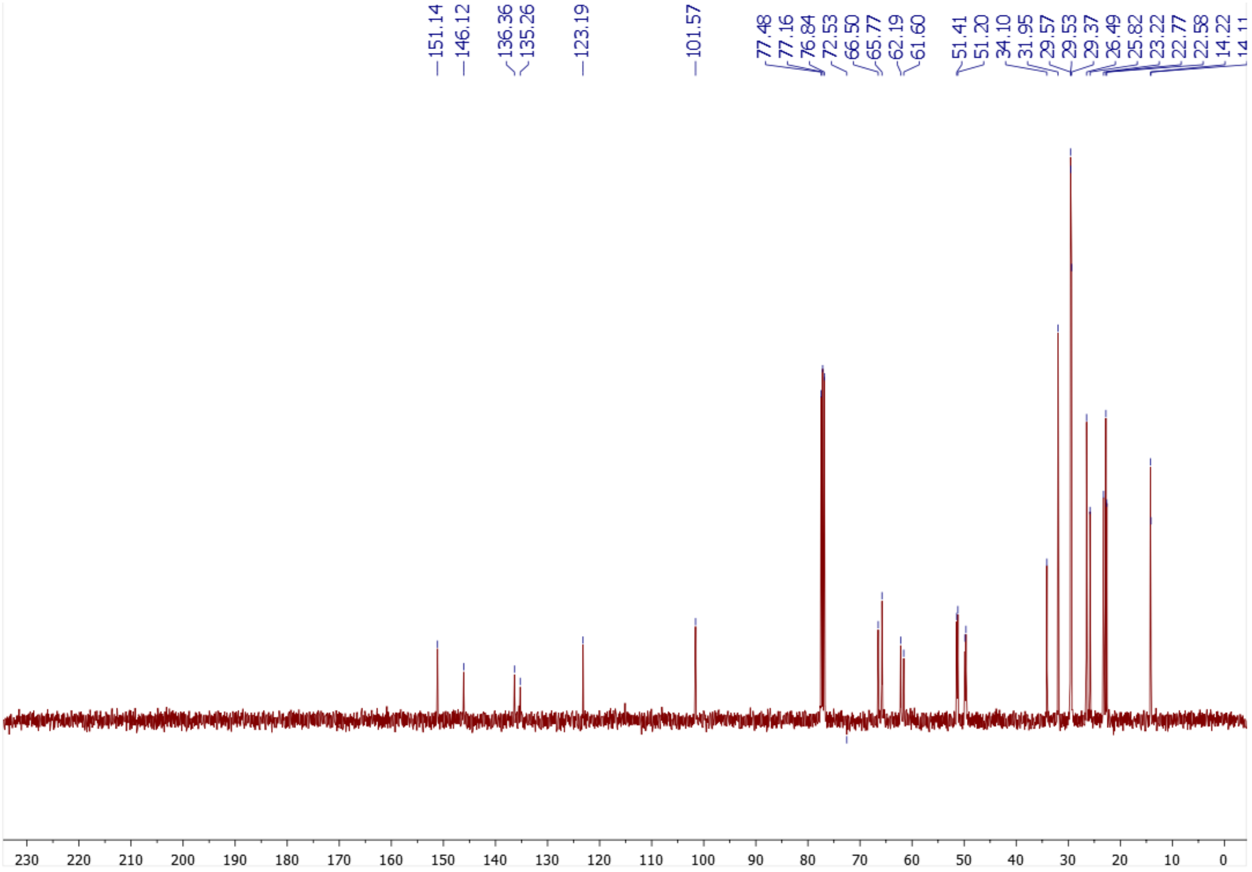

^{13}C{H} NMR spectrum of compound **5f10**

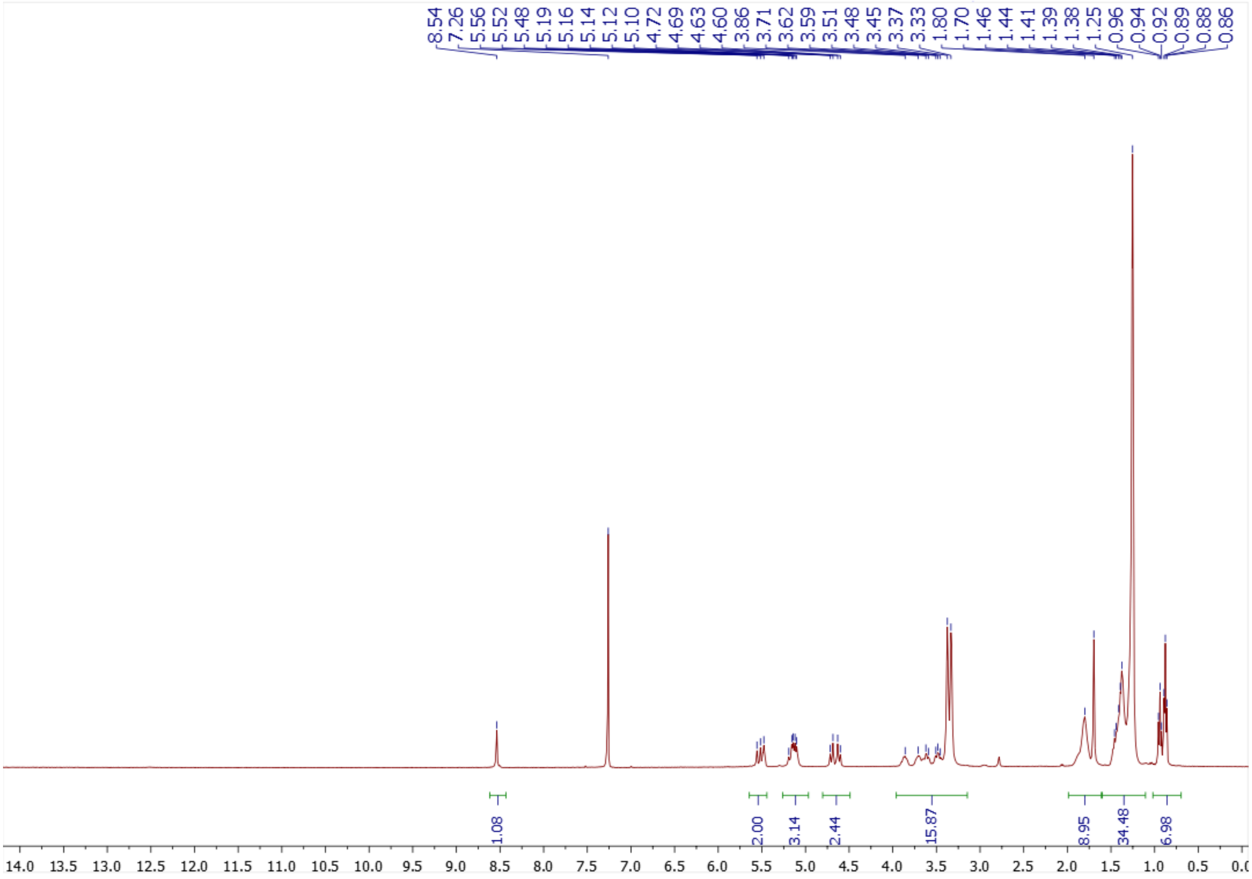

^{1}H NMR spectrum of compound **5f$_{12}$**

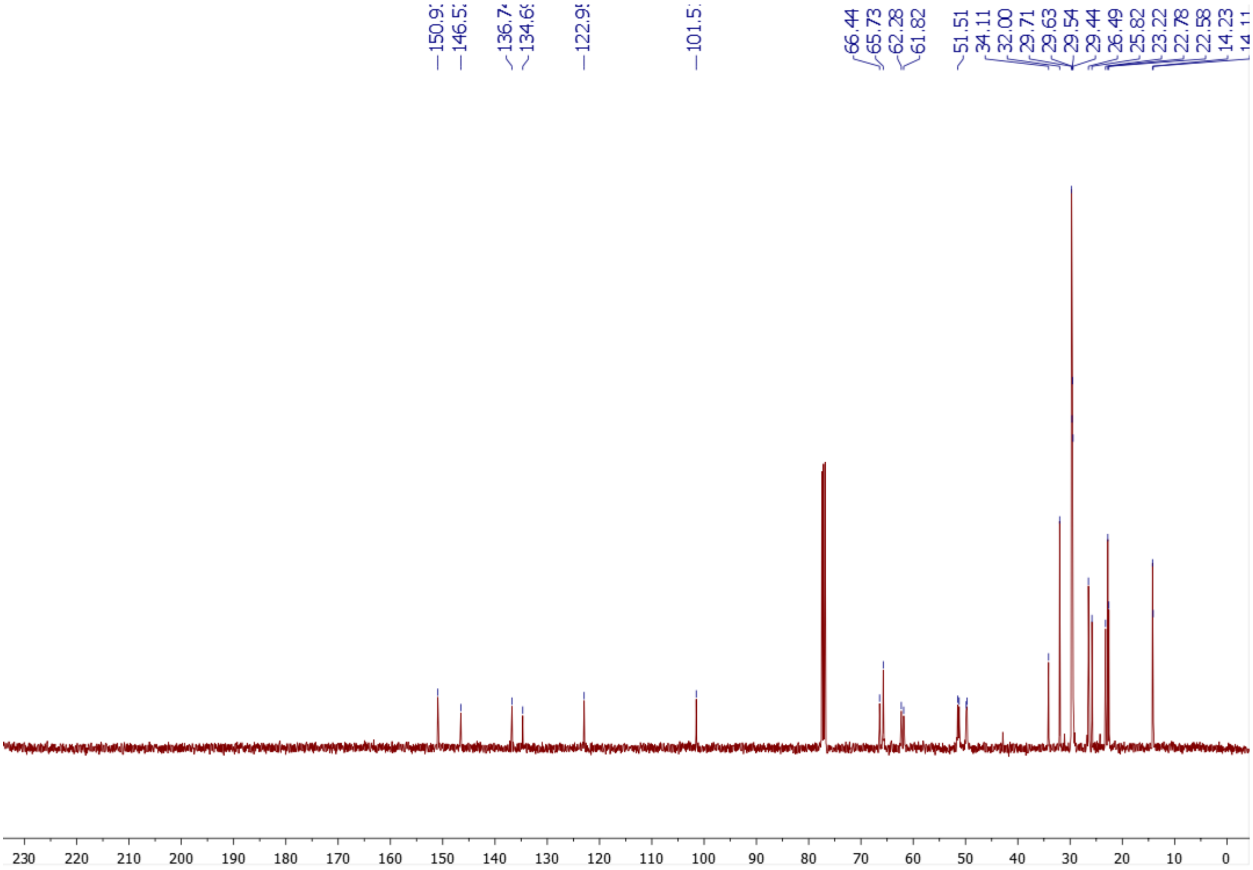

^{13}C{H} NMR spectrum of compound **5f$_{12}$**

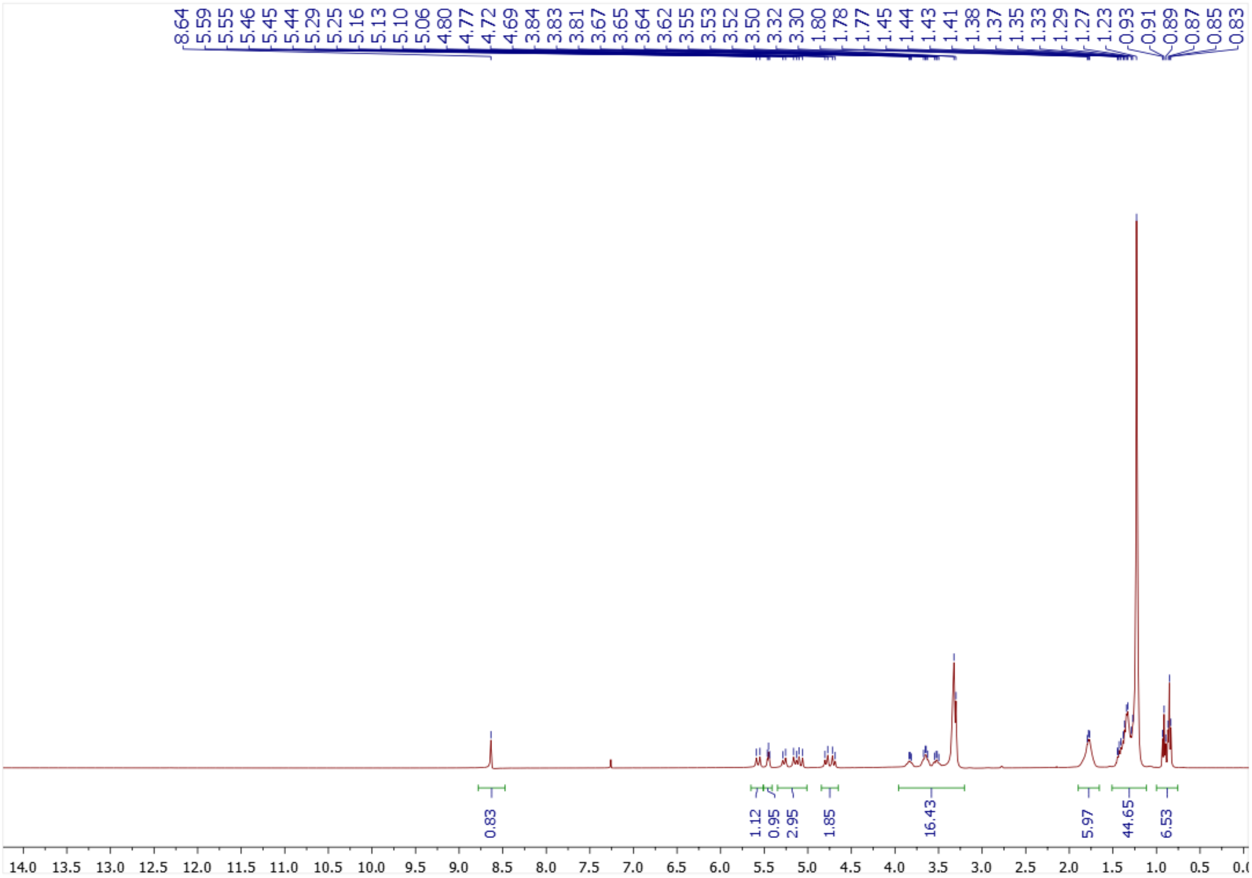

^{1}H NMR spectrum of compound **5f14**

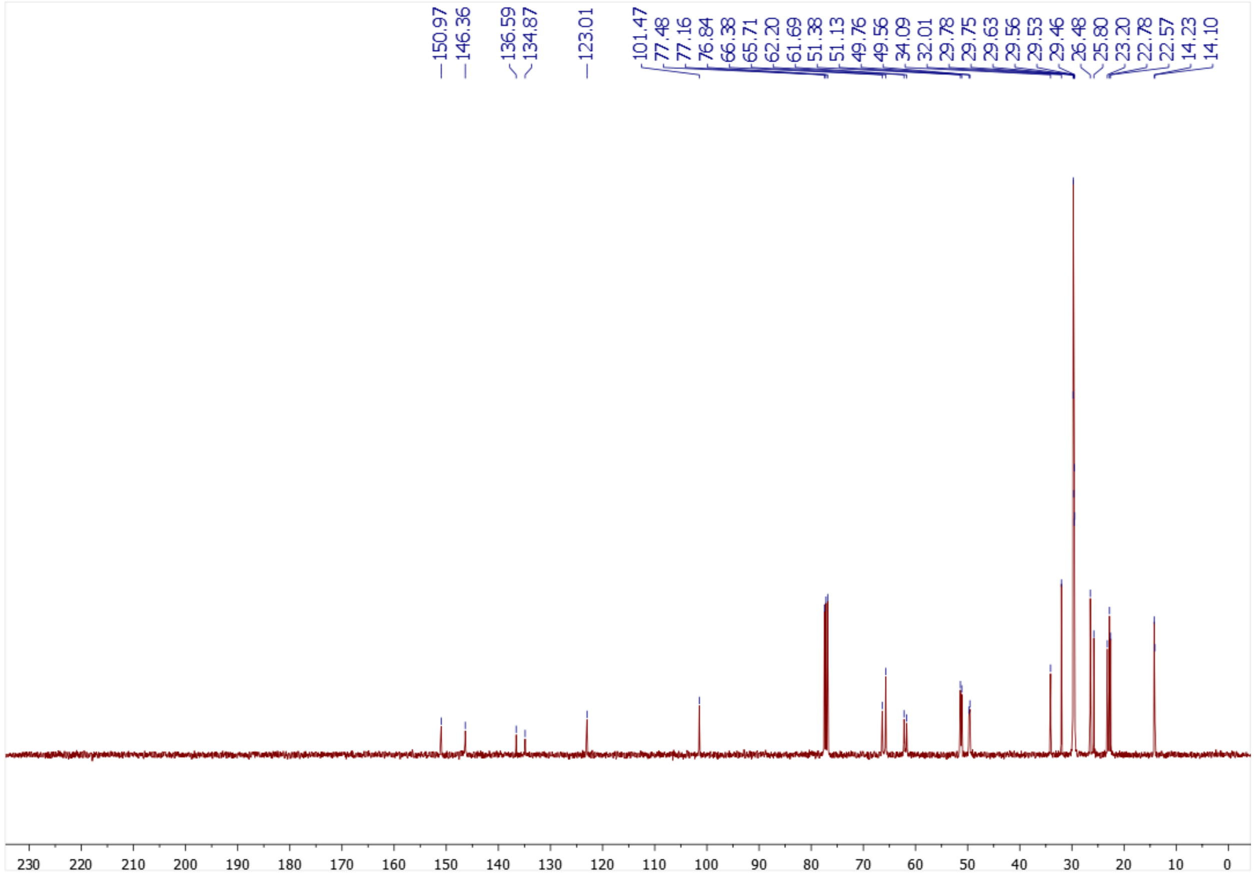

^{13}C{H} NMR spectrum of compound **5f14**

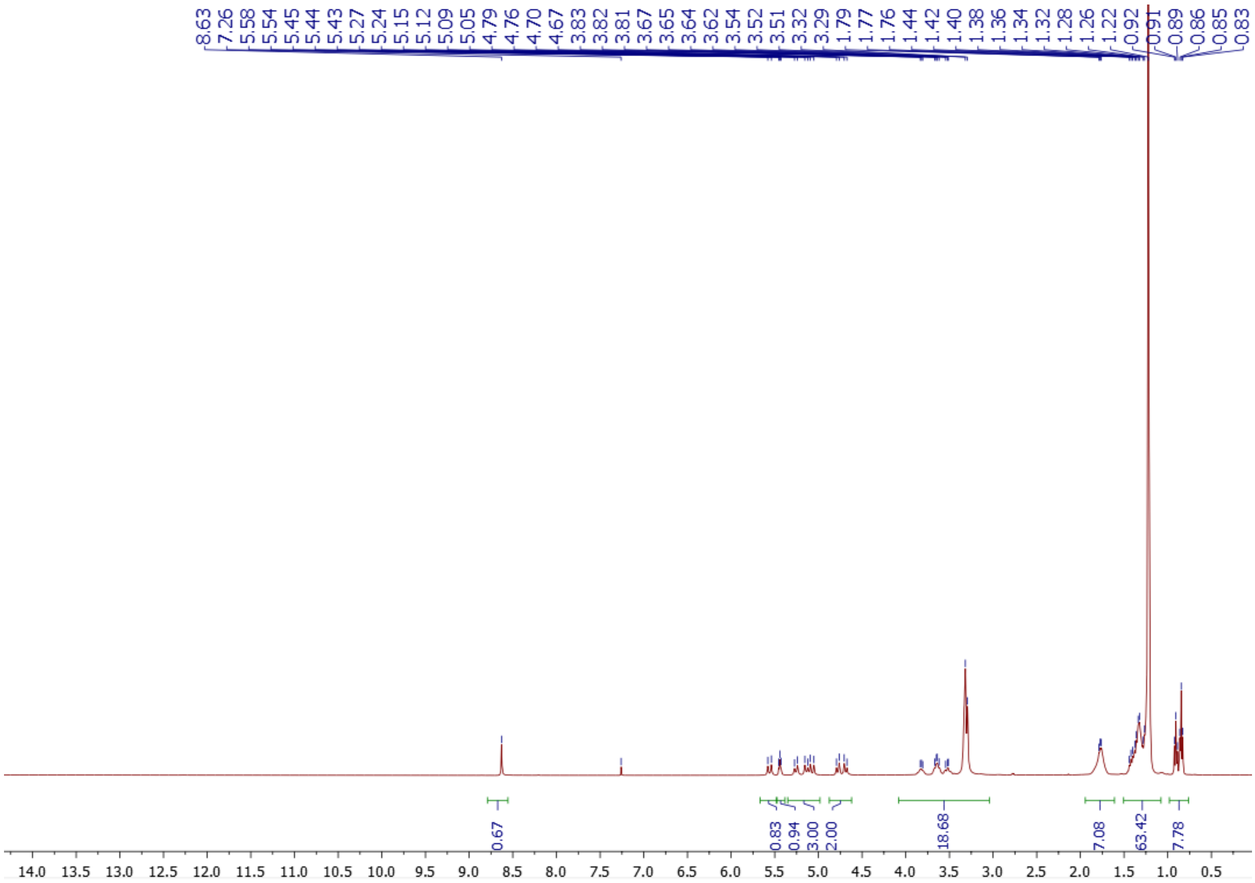

^{1}H NMR spectrum of compound **5f16**

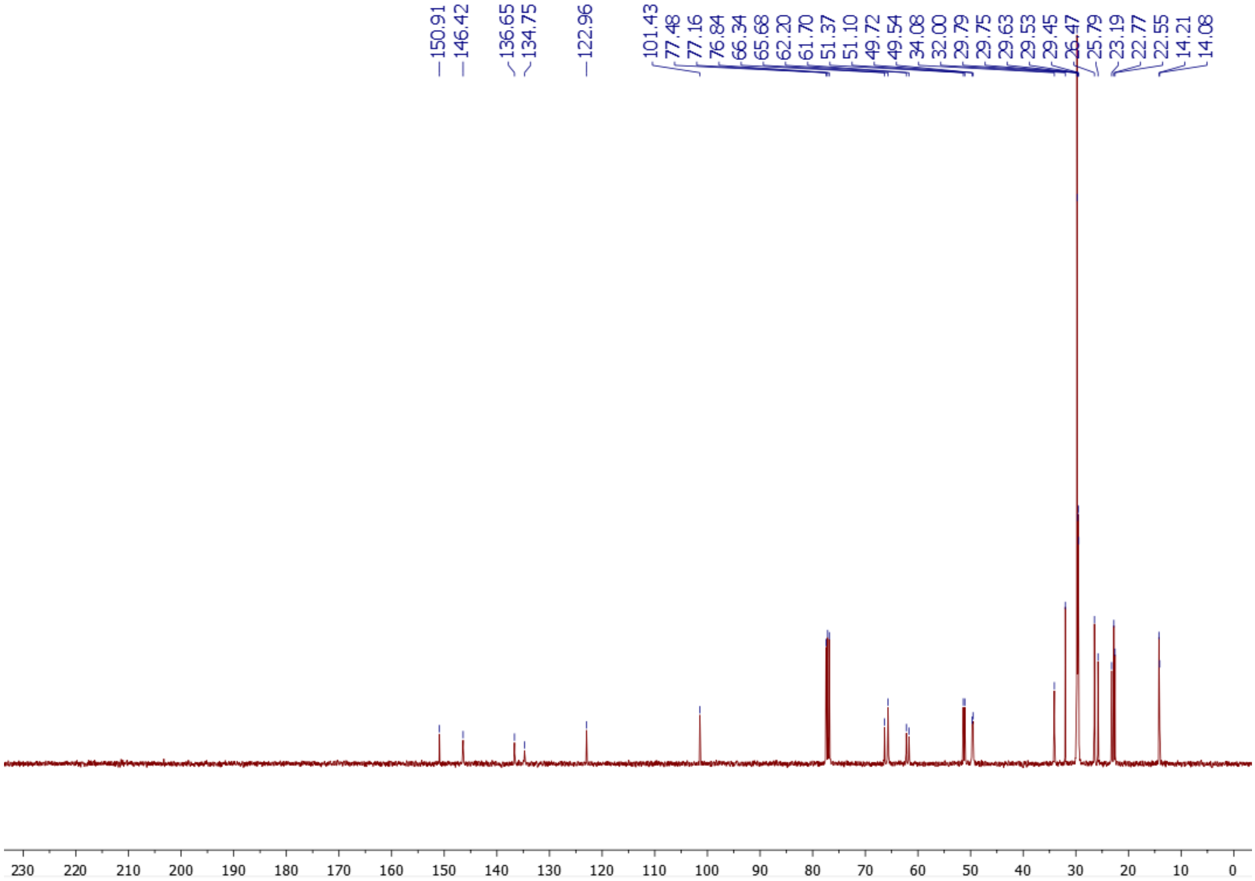

^{13}C{H} NMR spectrum of compound **5f16**

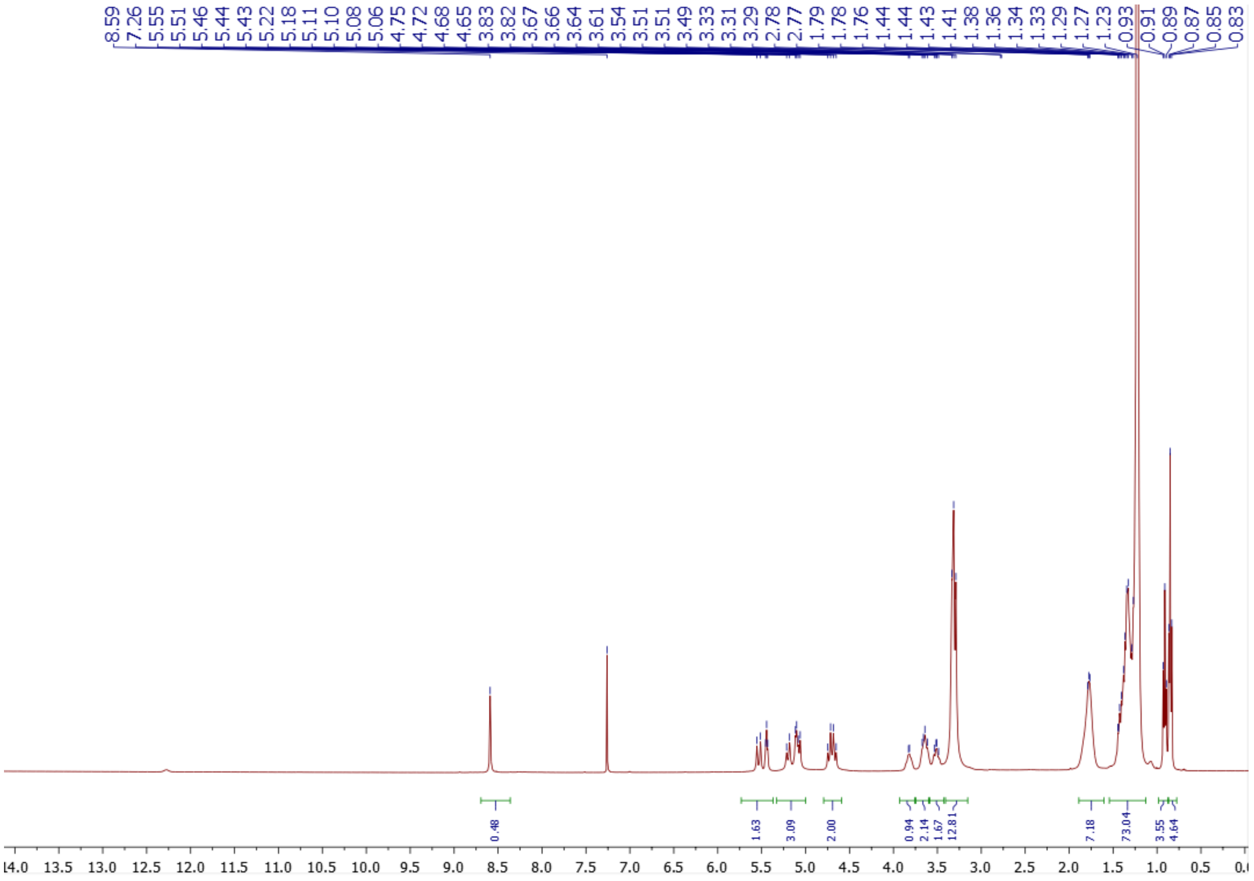

^{1}H NMR spectrum of compound **5f**18

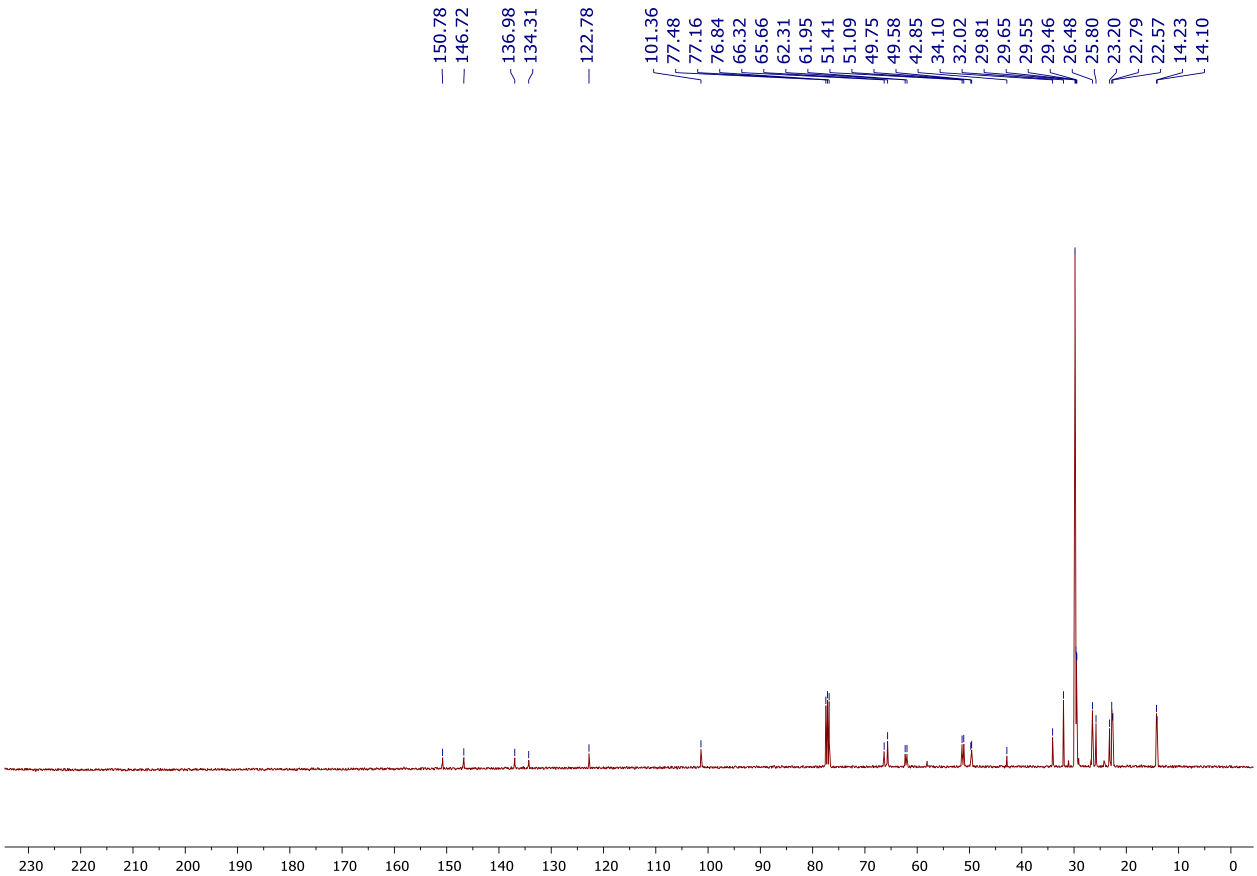

^{13}C{H} NMR spectrum of compound **5f**18

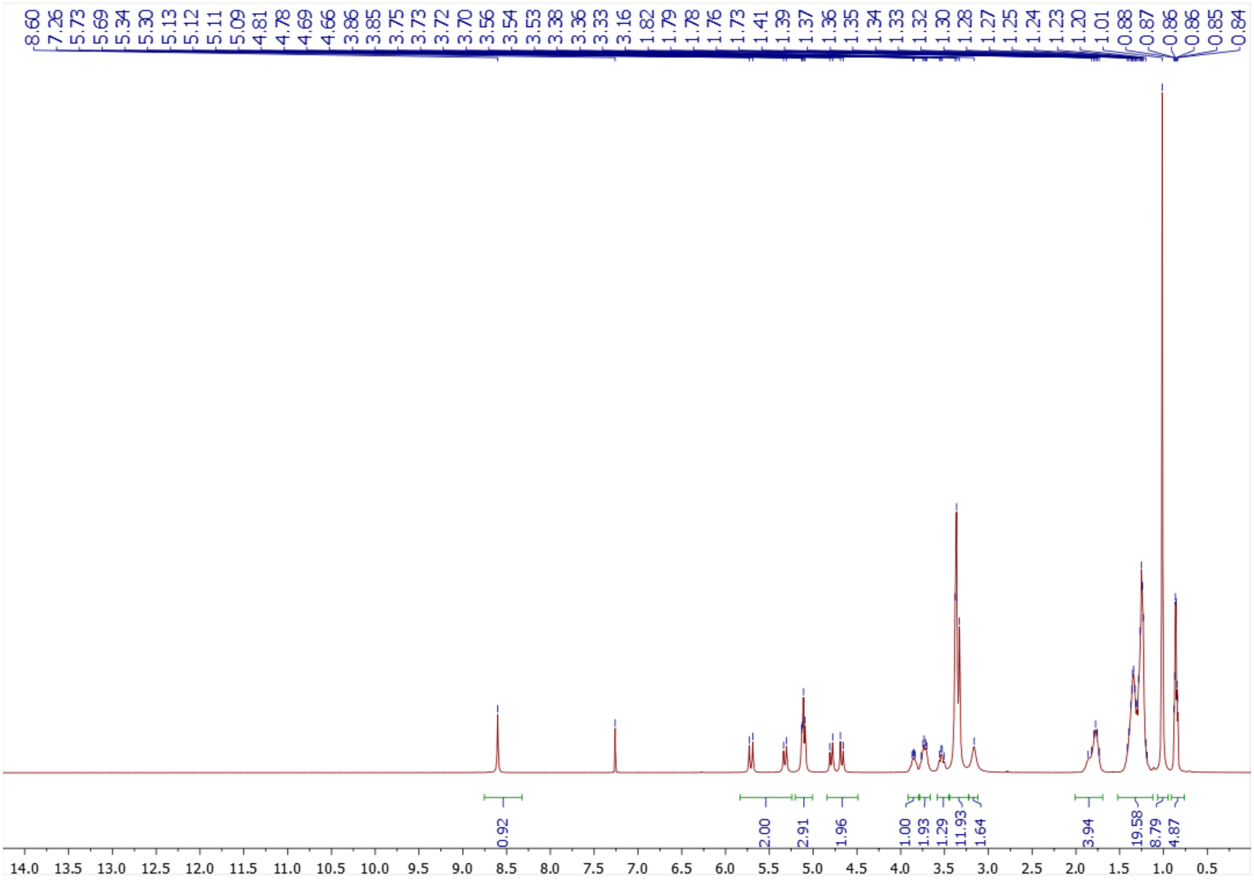

^{1}H NMR spectrum of compound **5g**$_8$

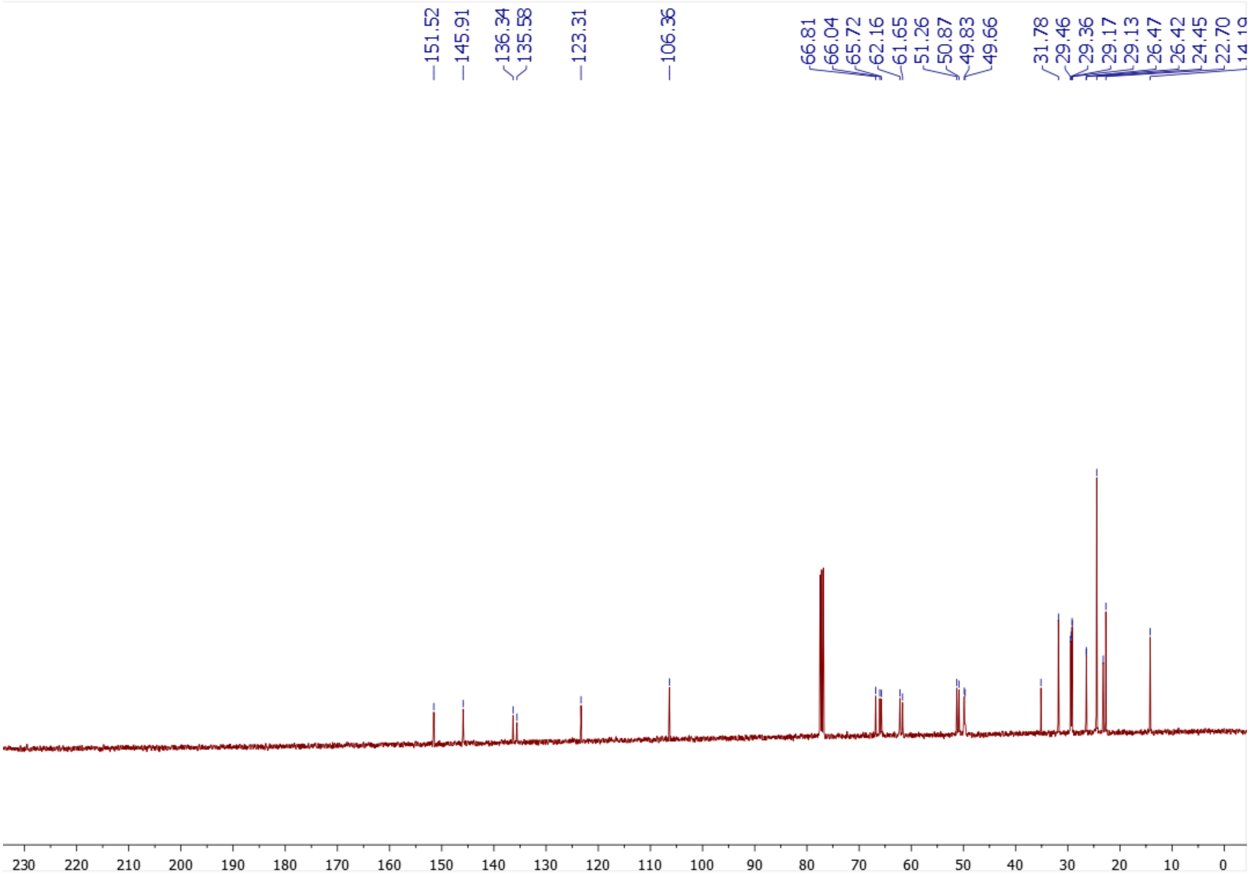

^{13}C{H} NMR spectrum of compound **5g**$_8$

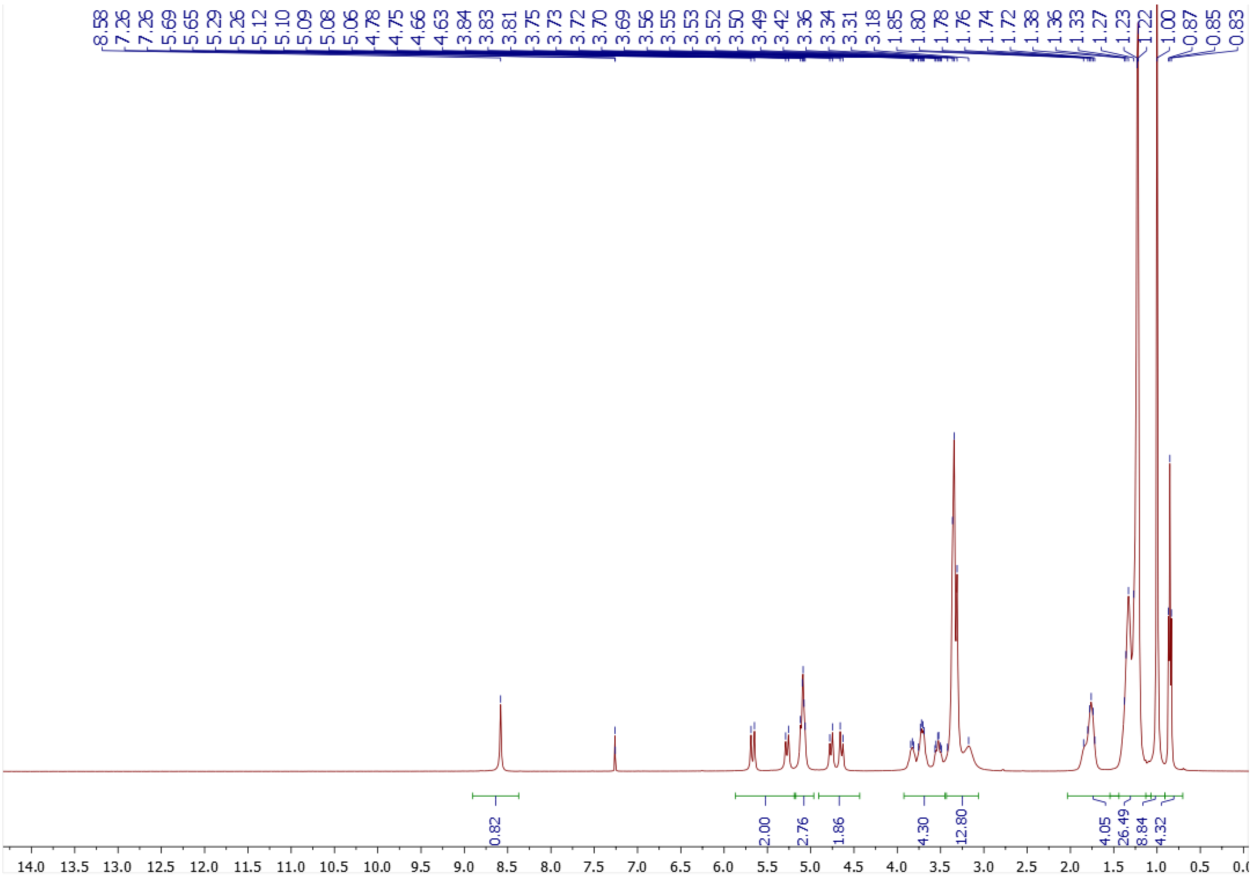

^{1}H NMR spectrum of compound **5g$_{10}$**

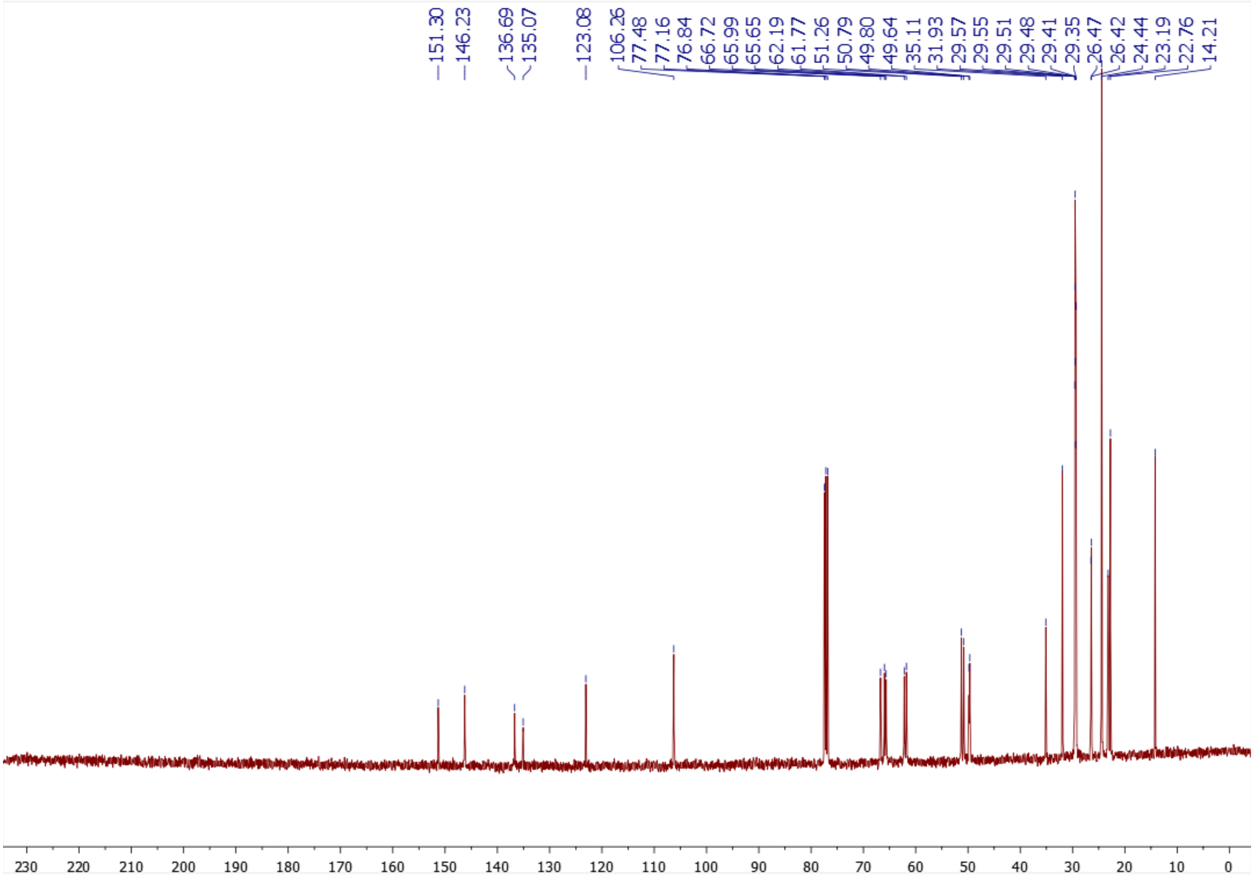

^{13}C{H} NMR spectrum of compound **5g$_{10}$**

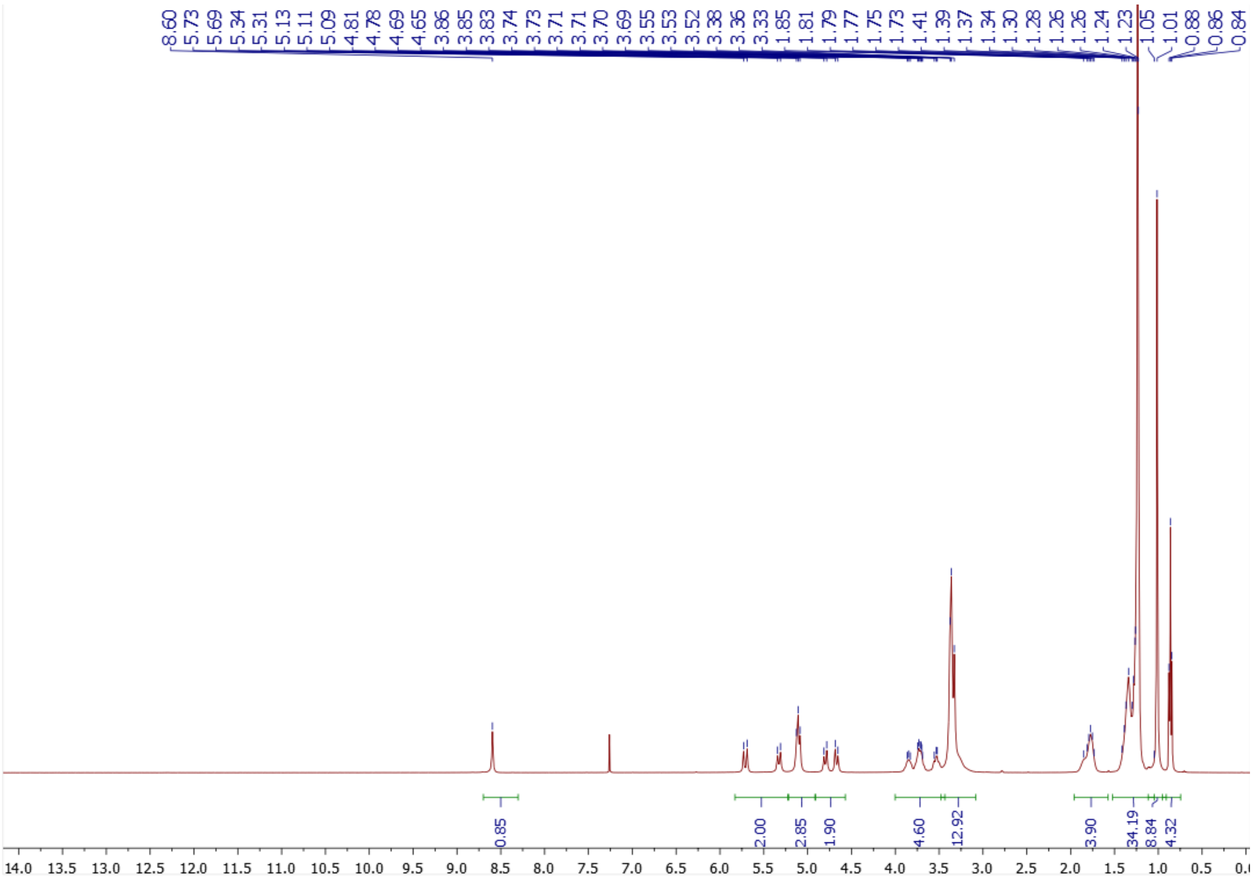

^{1}H NMR spectrum of compound **5g**$_{12}$

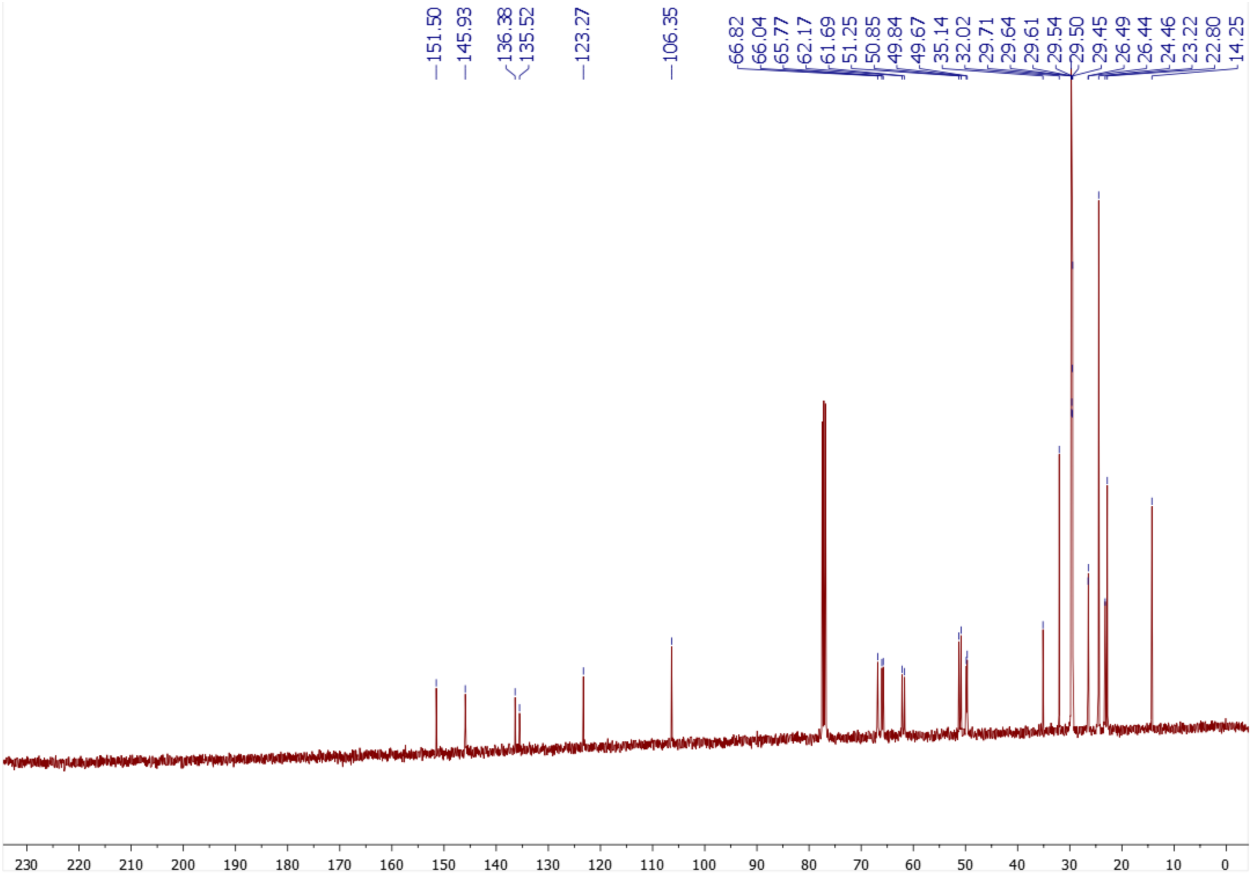

^{13}C{H} NMR spectrum of compound **5g**$_{12}$

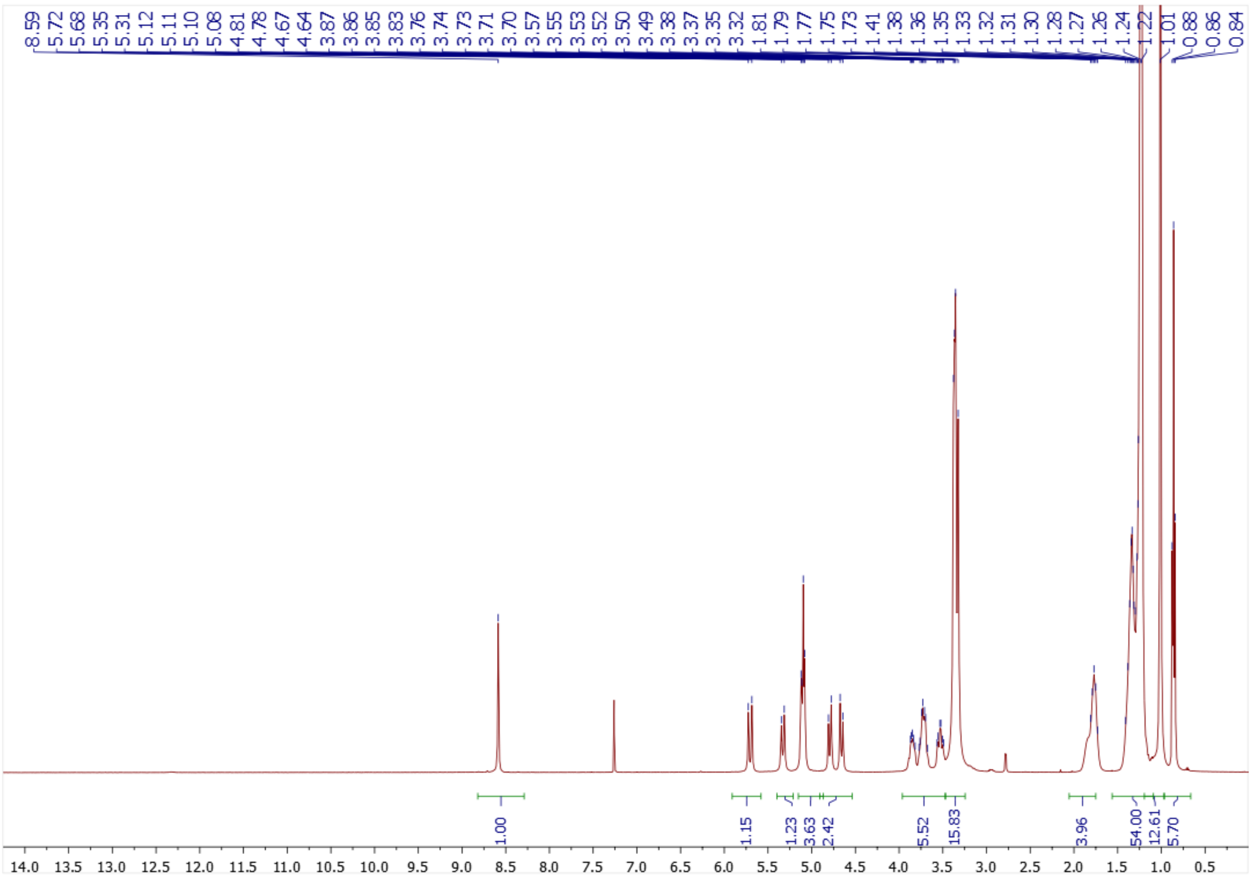

^{1}H NMR spectrum of compound **5g14**

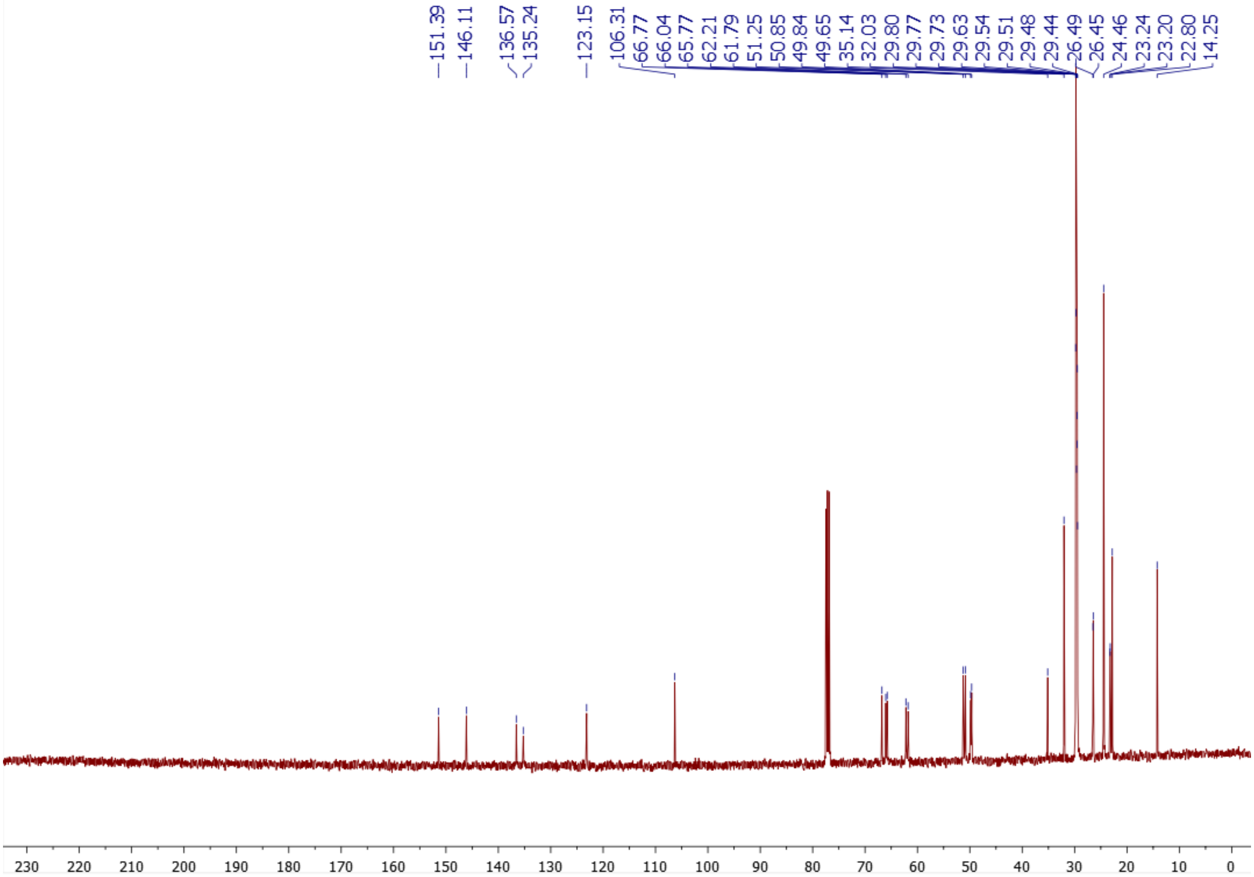

$^{13}C\{H\}$ NMR spectrum of compound **5g14**

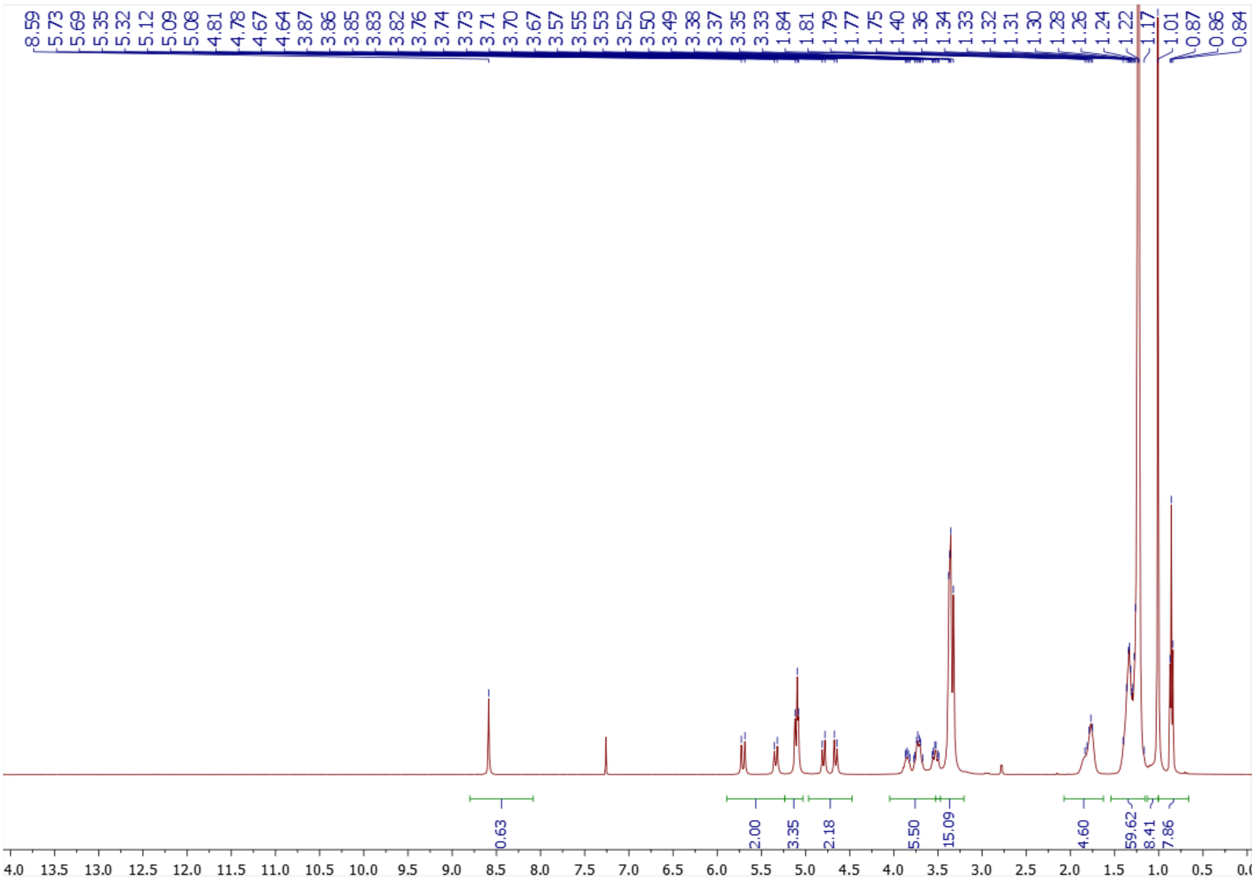

^{1}H NMR spectrum of compound **5g**$_{16}$

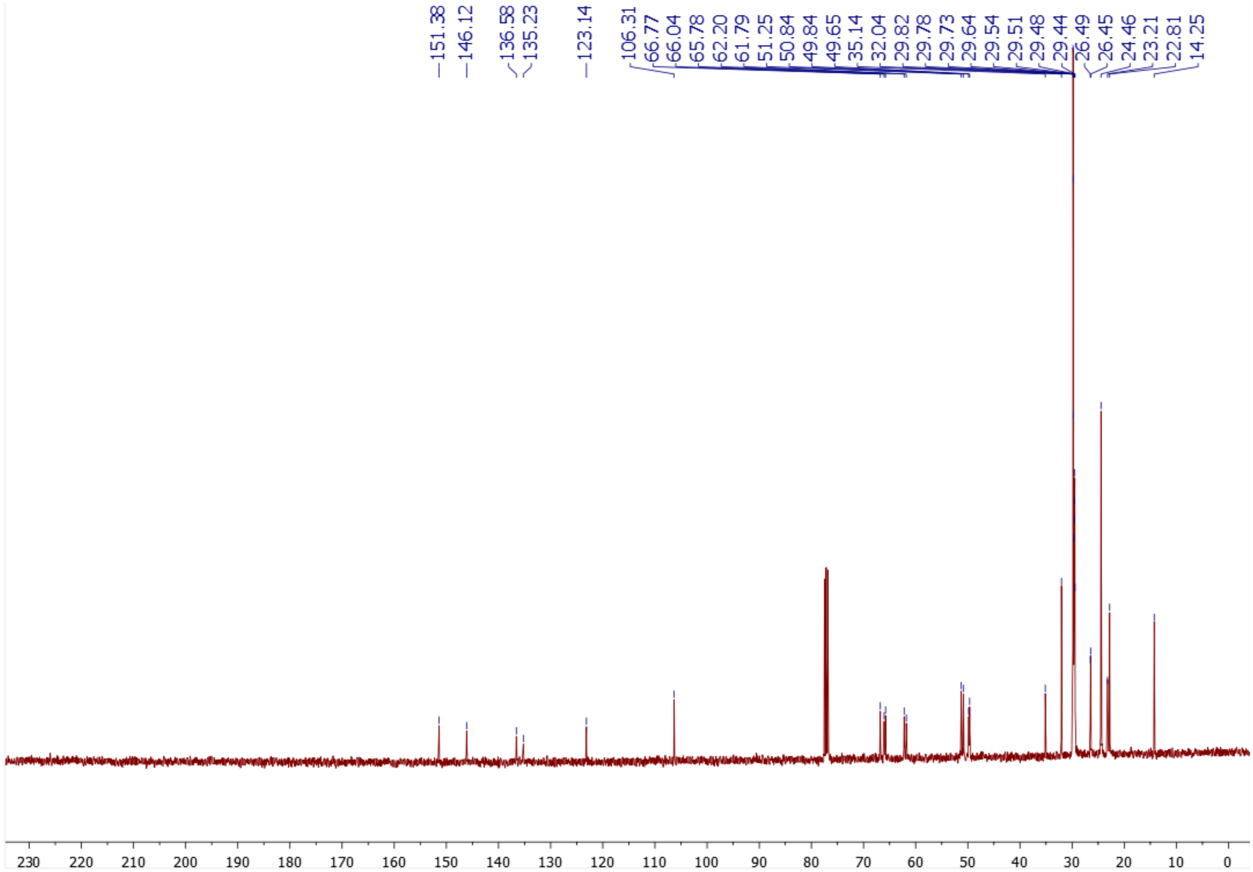

^{13}C{H} NMR spectrum of compound **5g**$_{16}$

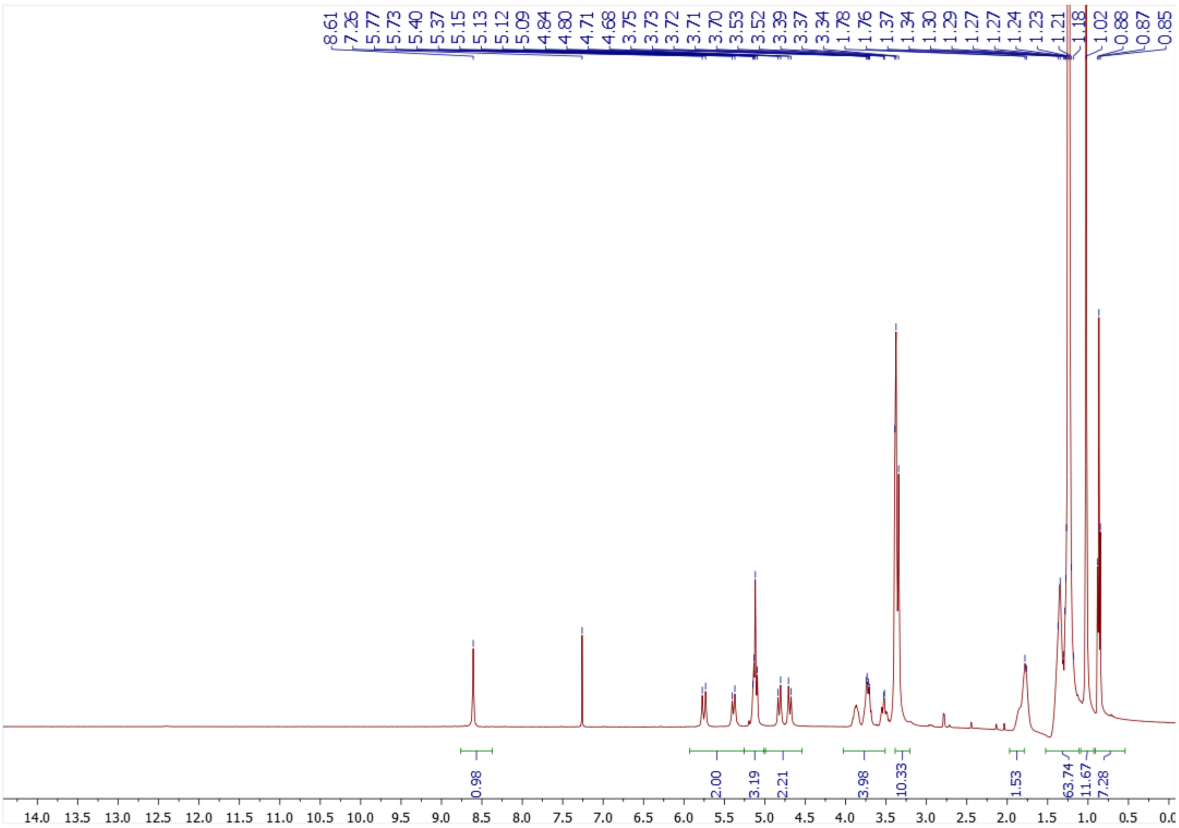

^{1}H NMR spectrum of compound **5g**18

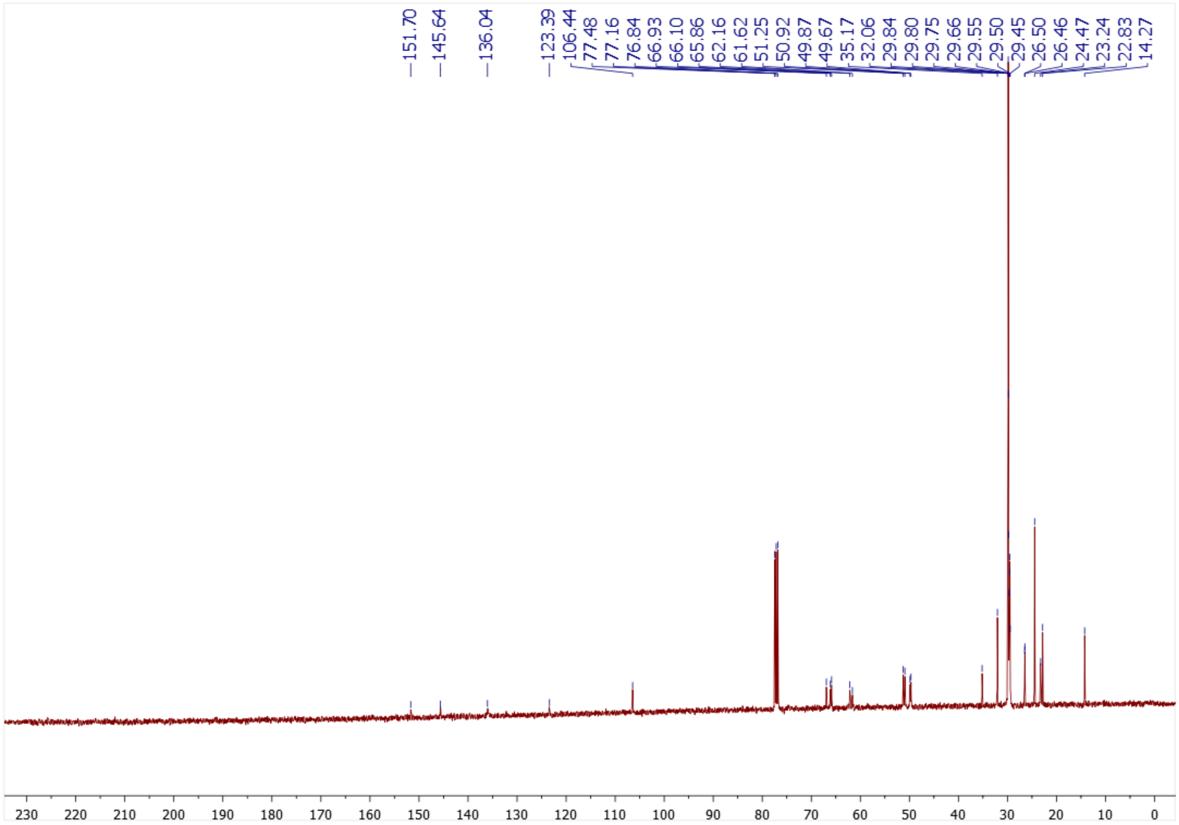

^{13}C{H} NMR spectrum of compound **5g**18

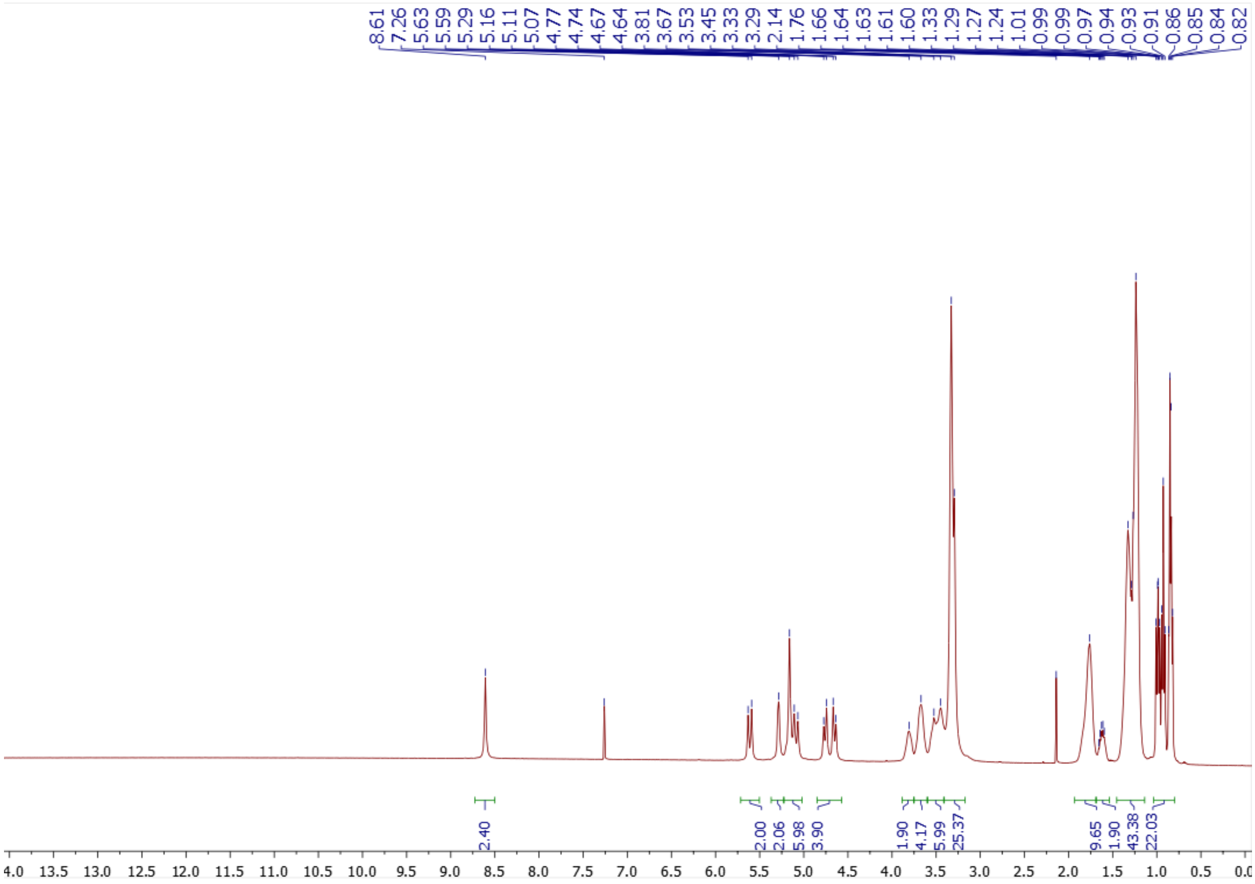

^{1}H NMR spectrum of compound **5h**$_8$

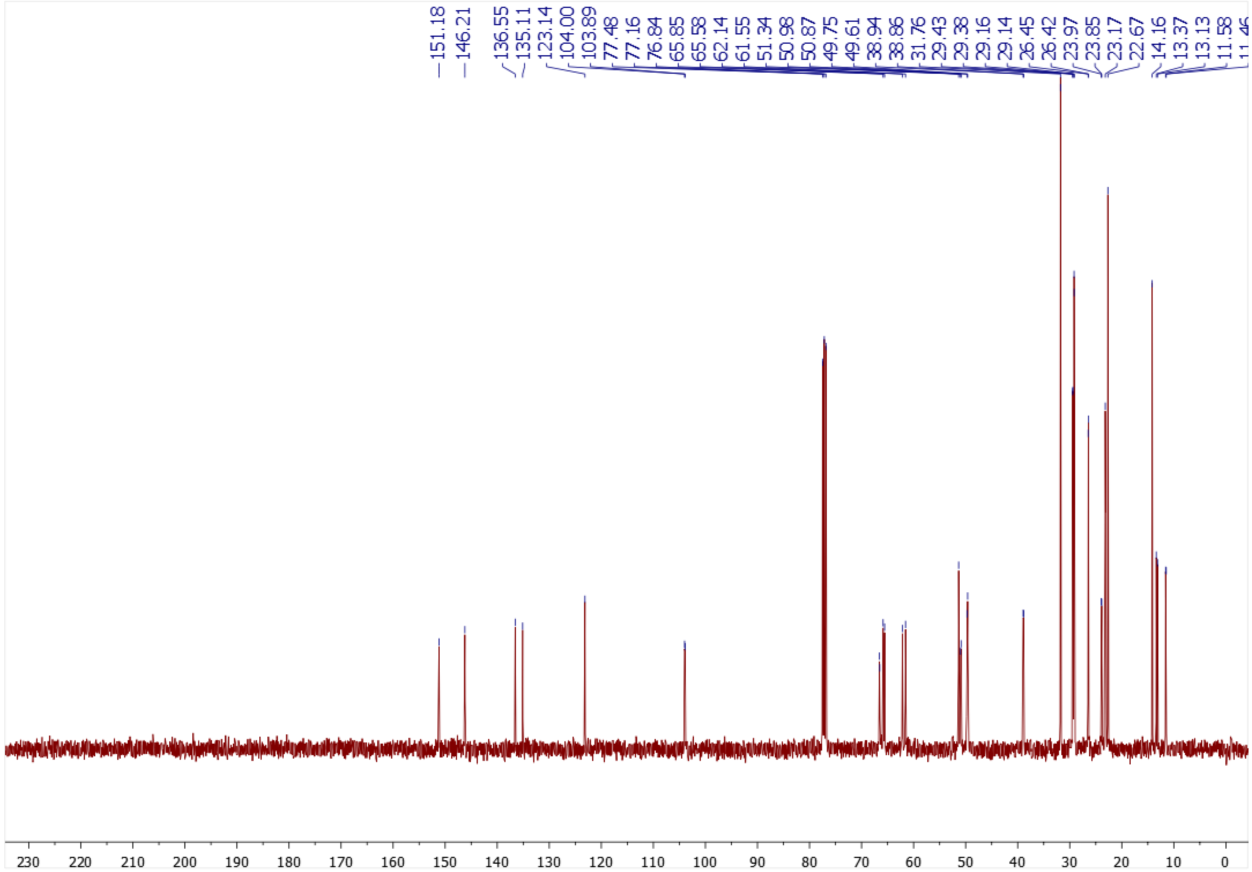

^{13}C{H} NMR spectrum of compound **5h**$_8$

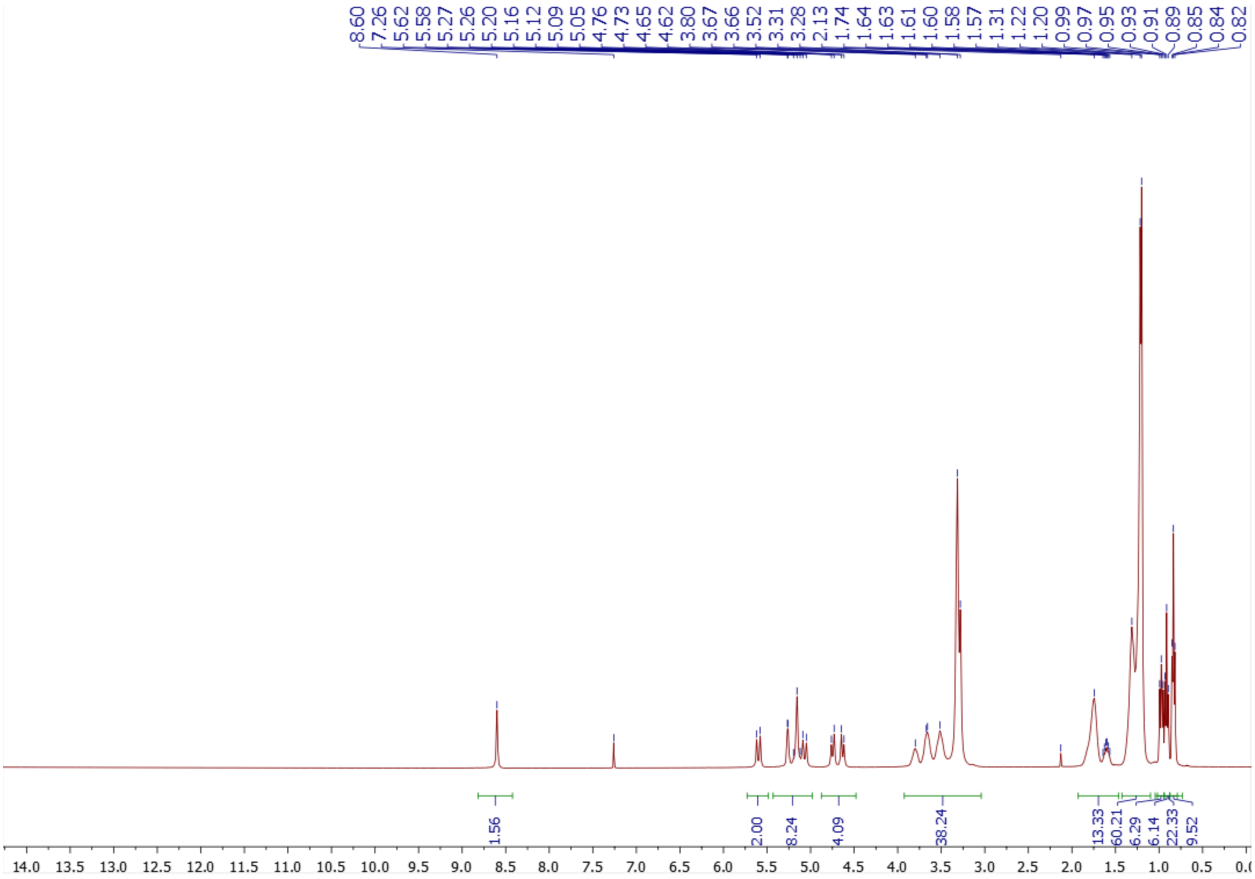

^{1}H NMR spectrum of compound **5h**$_{10}$

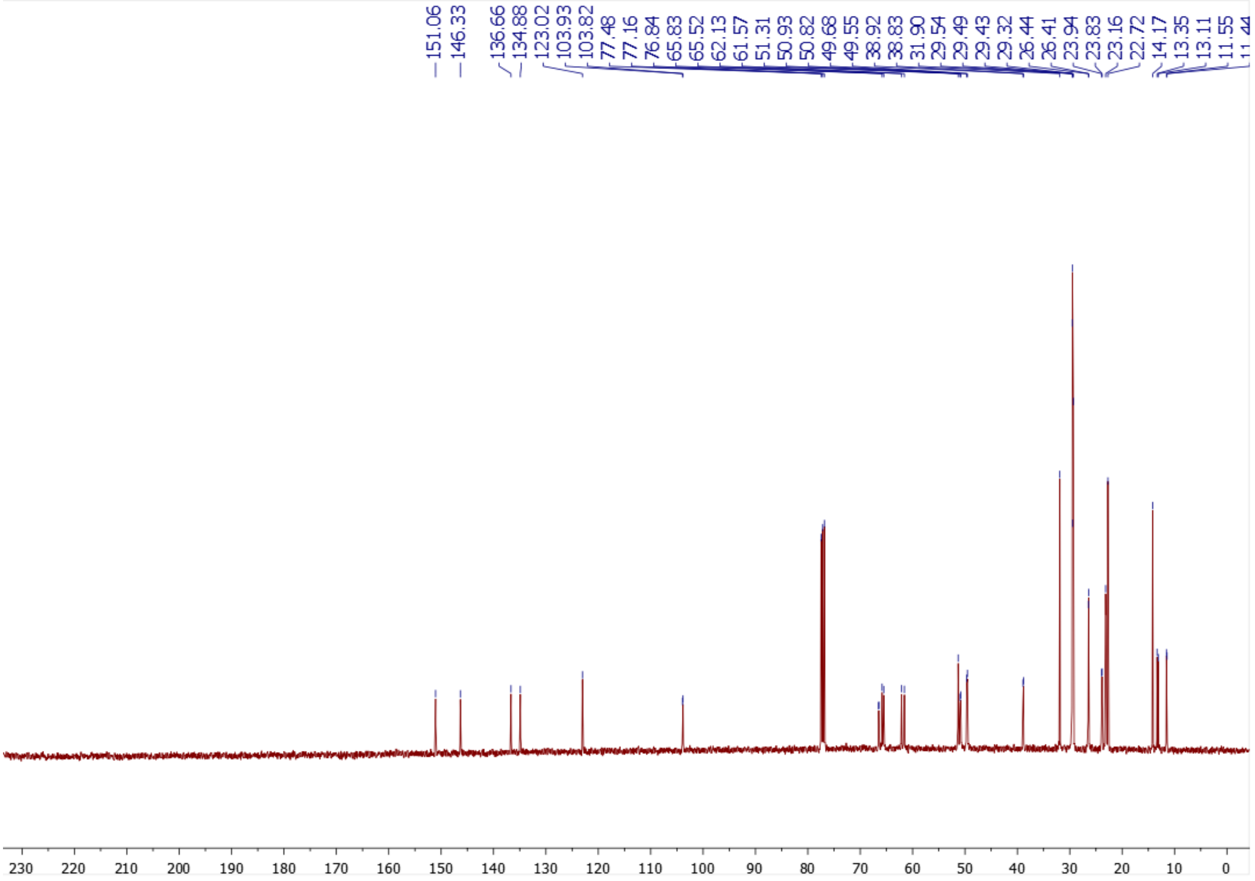

$^{13}C\{H\}$ NMR spectrum of compound **5h**$_{10}$

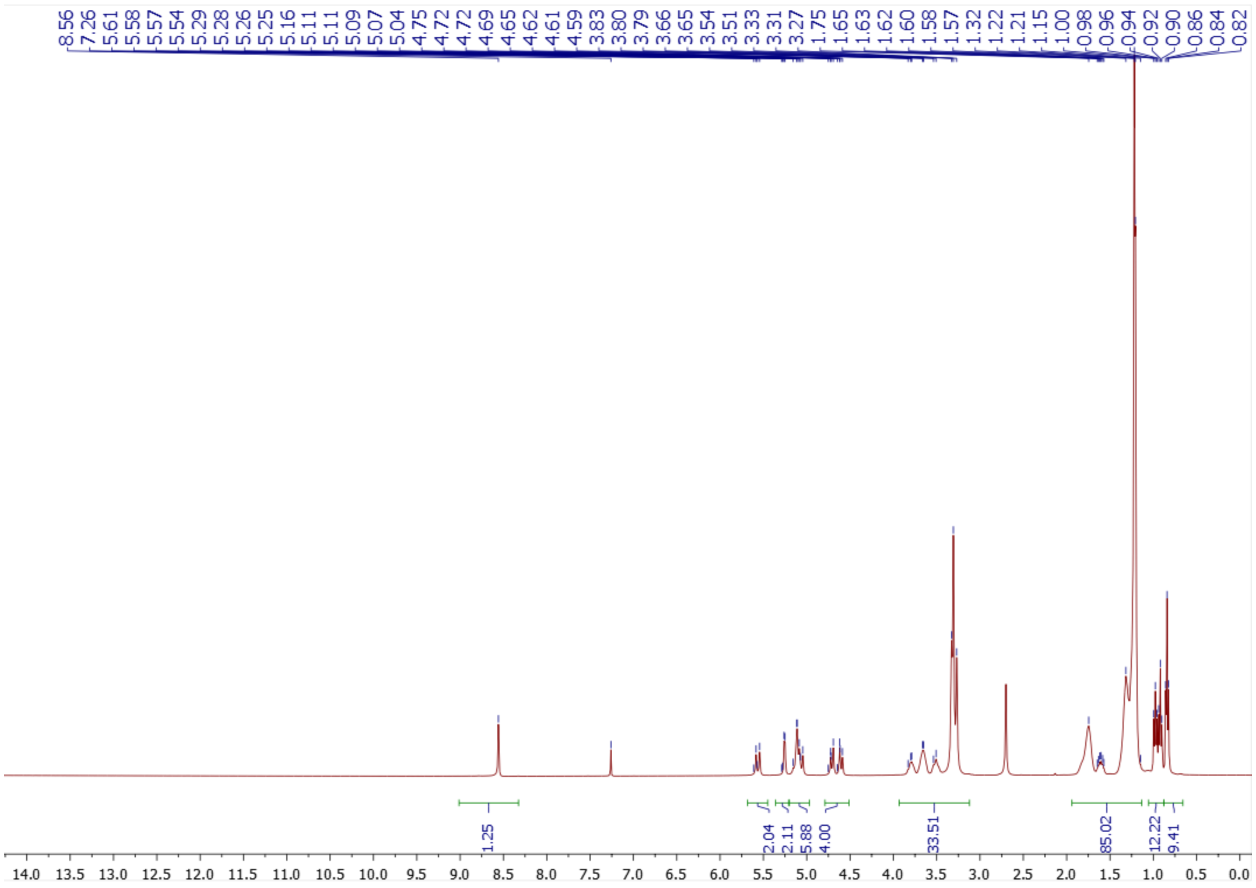

^{1}H NMR spectrum of compound **5h$_{12}$**

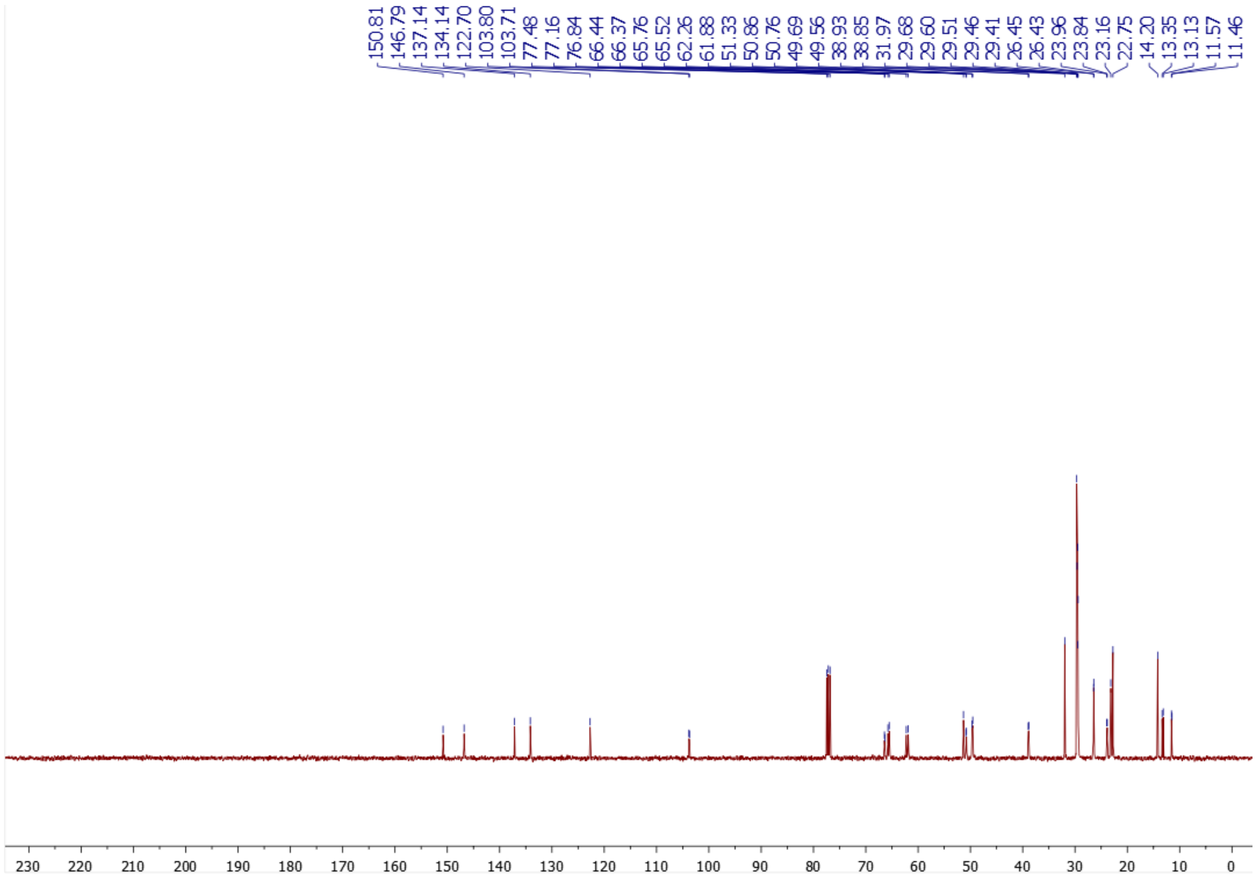

$^{13}C\{H\}$ NMR spectrum of compound **5h$_{12}$**

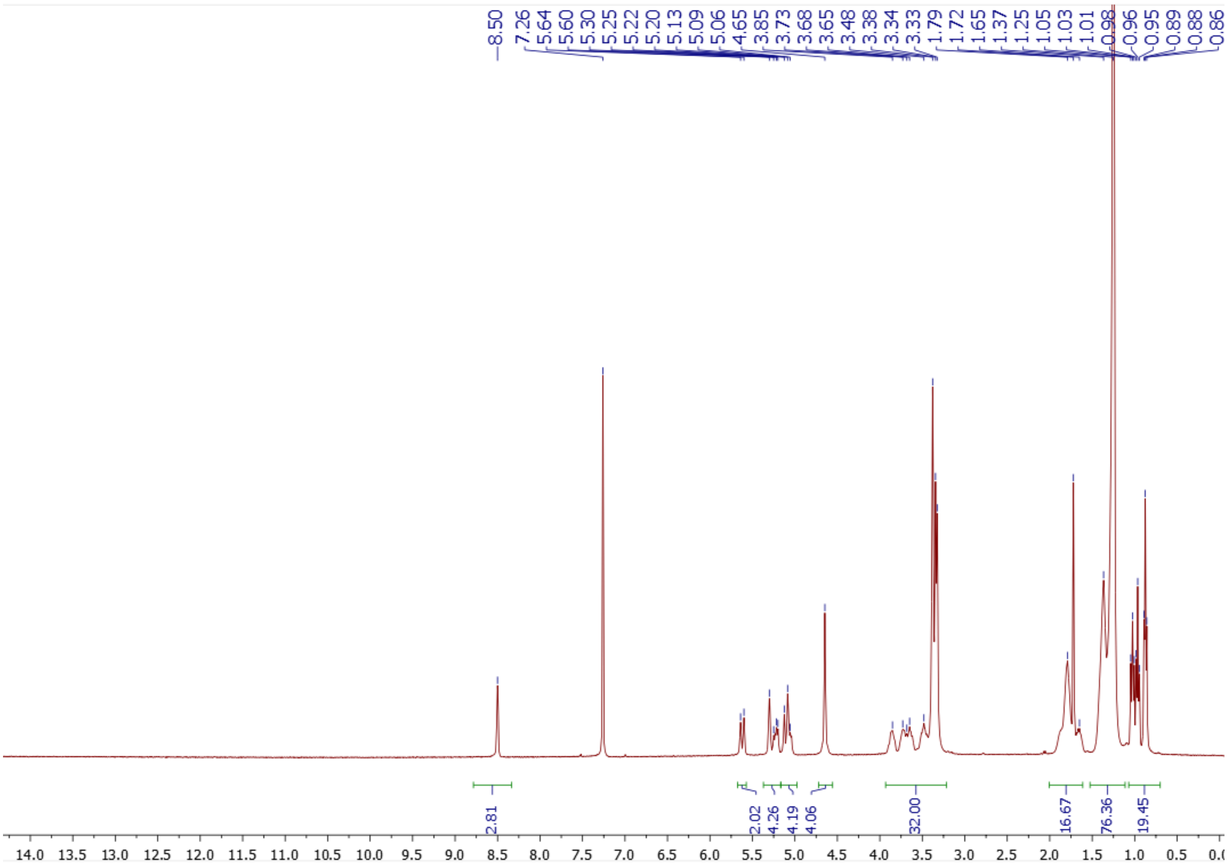

^{1}H NMR spectrum of compound **5h₁₄**

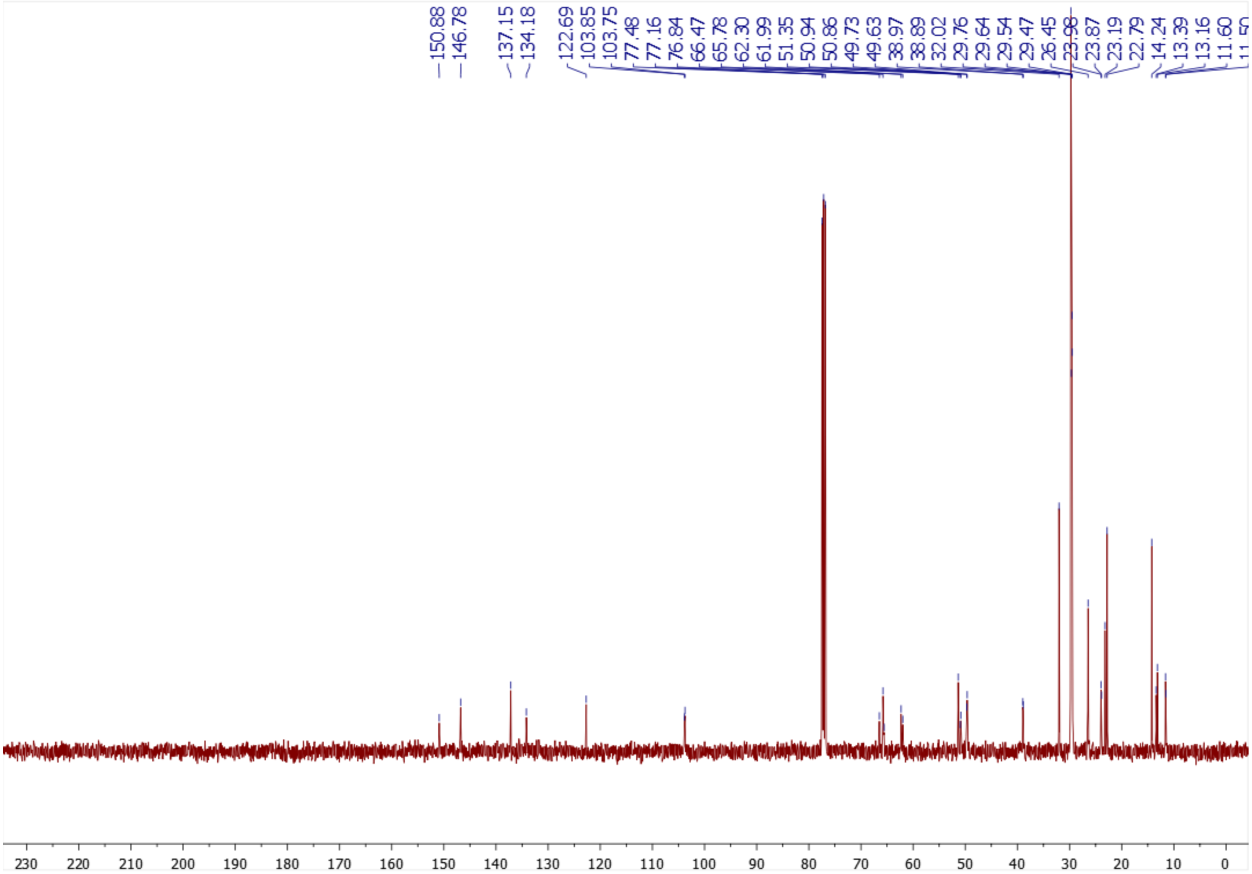

^{13}C{H} NMR spectrum of compound **5h$_{14}$**

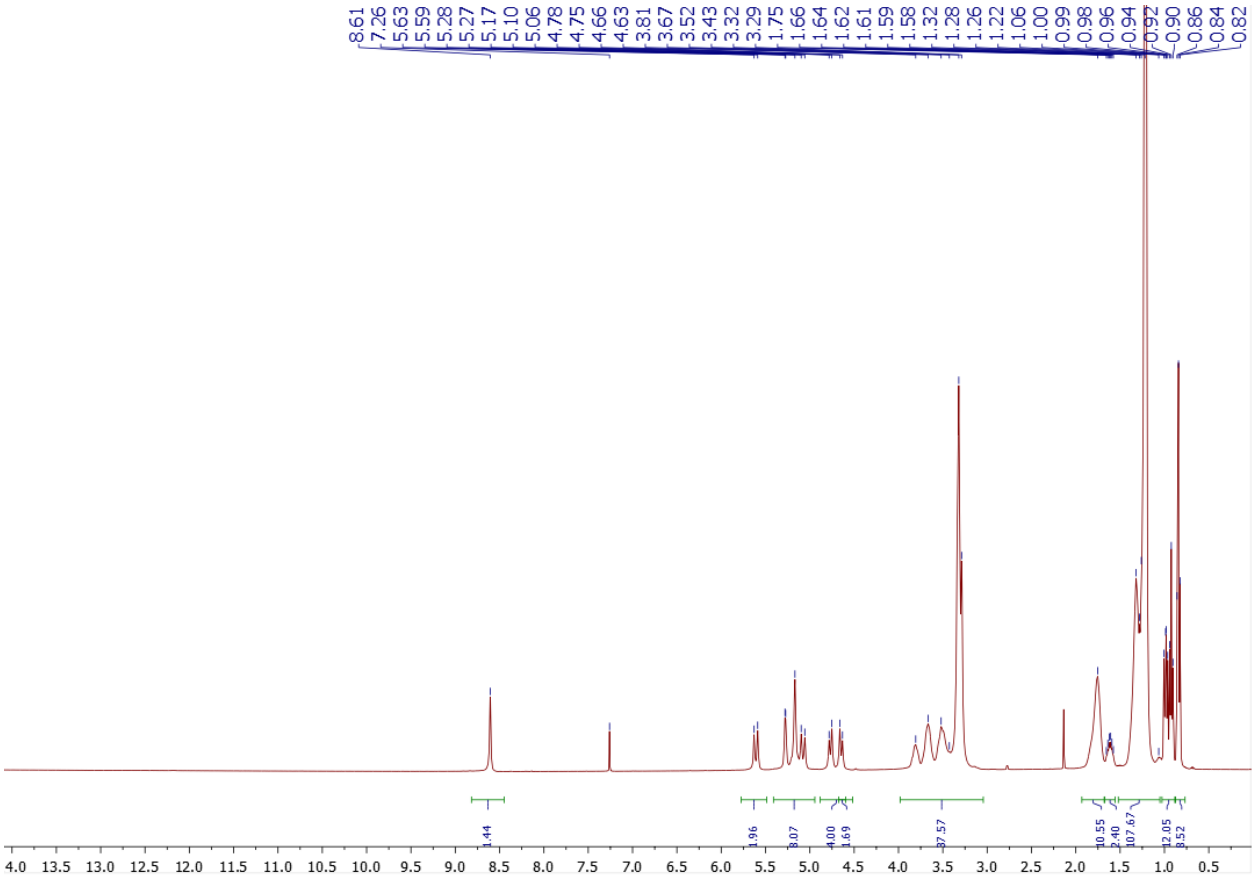

^{1}H NMR spectrum of compound **5h**$_{16}$

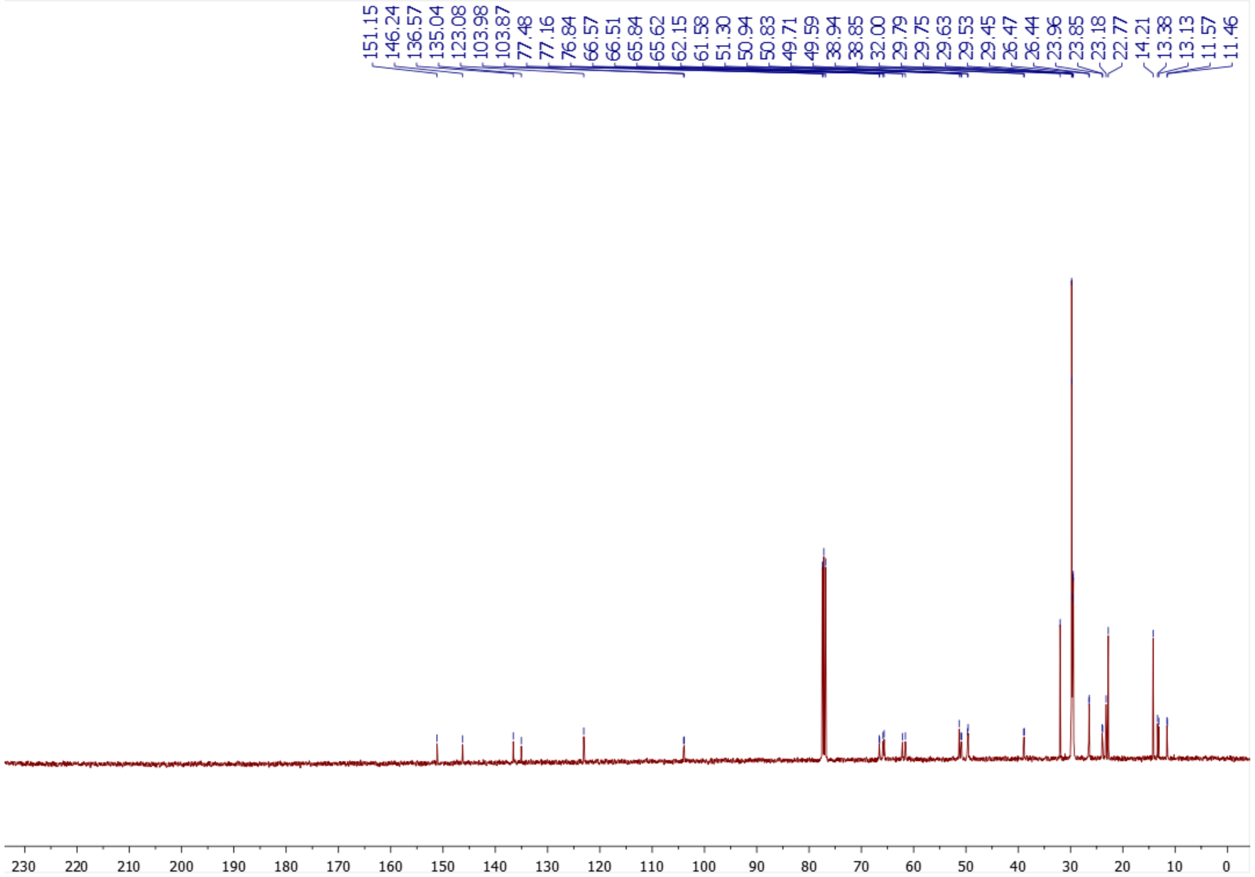

^{13}C{H} NMR spectrum of compound **5h**$_{16}$

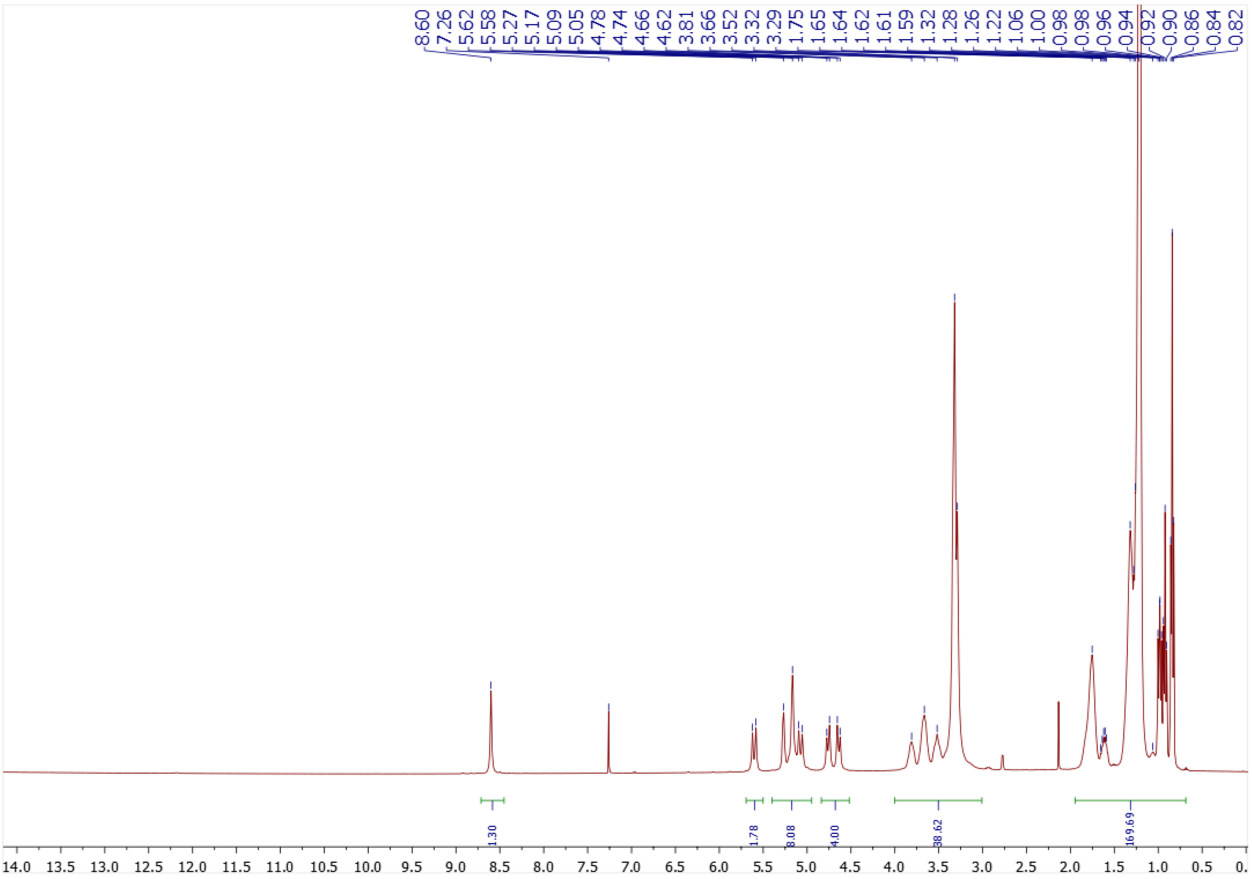

^{1}H NMR spectrum of compound **5h**$_{18}$

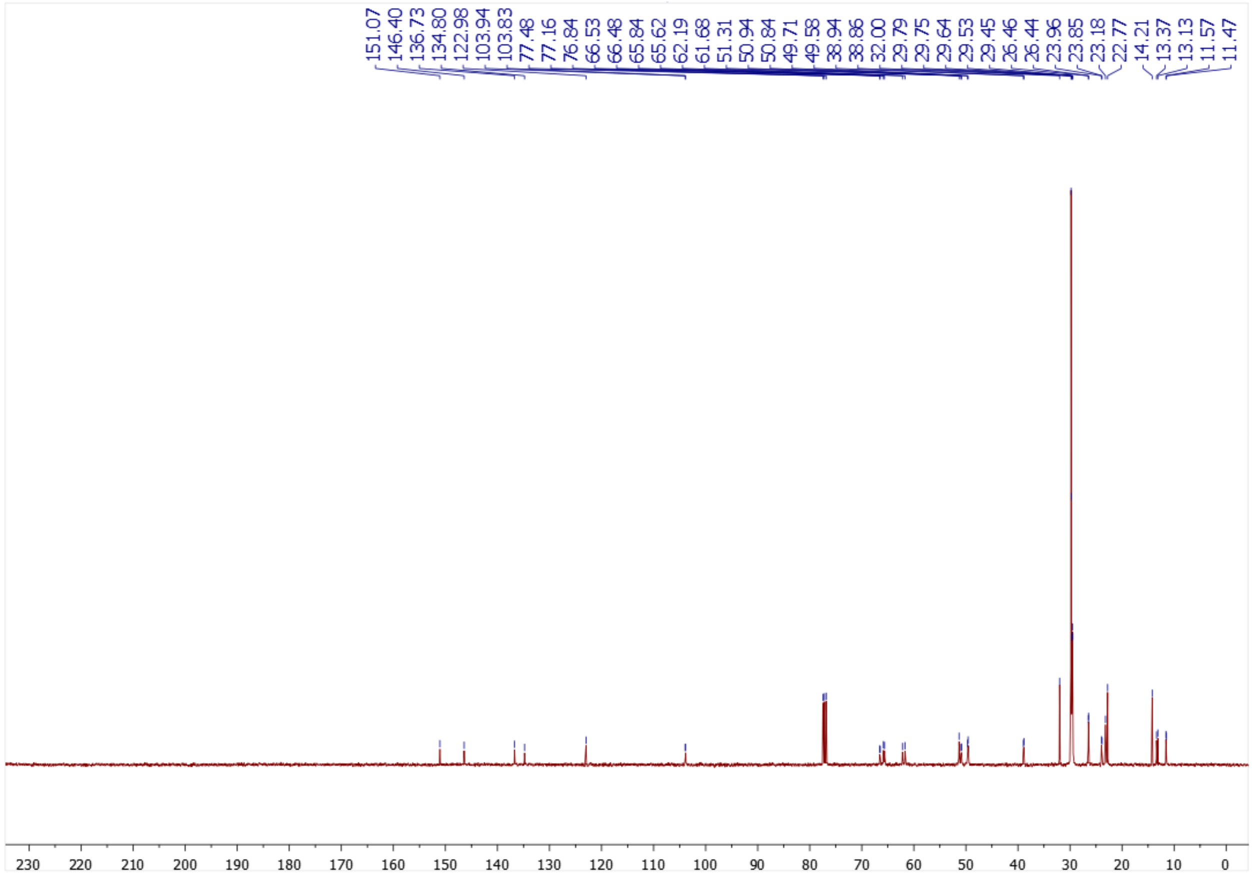

^{13}C{H} NMR spectrum of compound **5h**$_{18}$

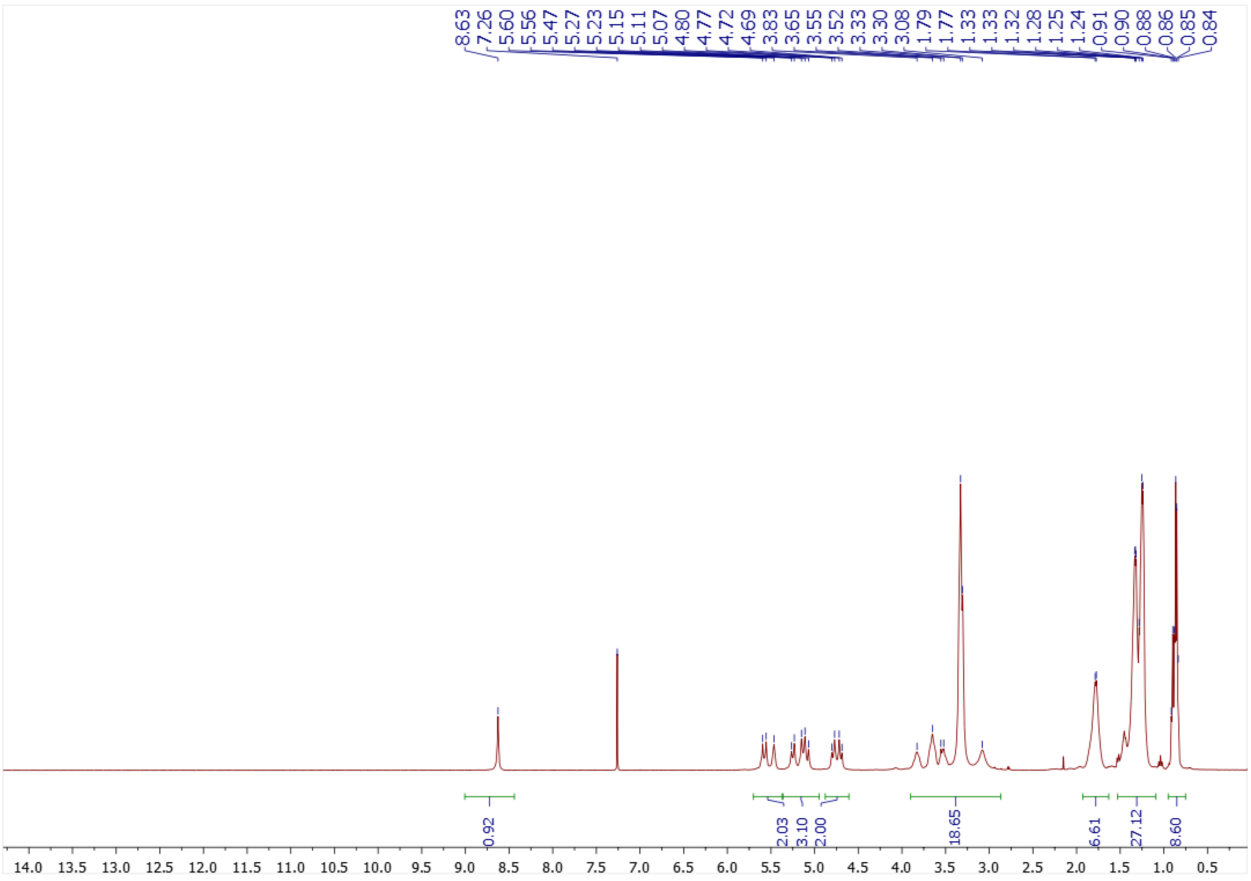

^{1}H NMR spectrum of compound **5i₈**

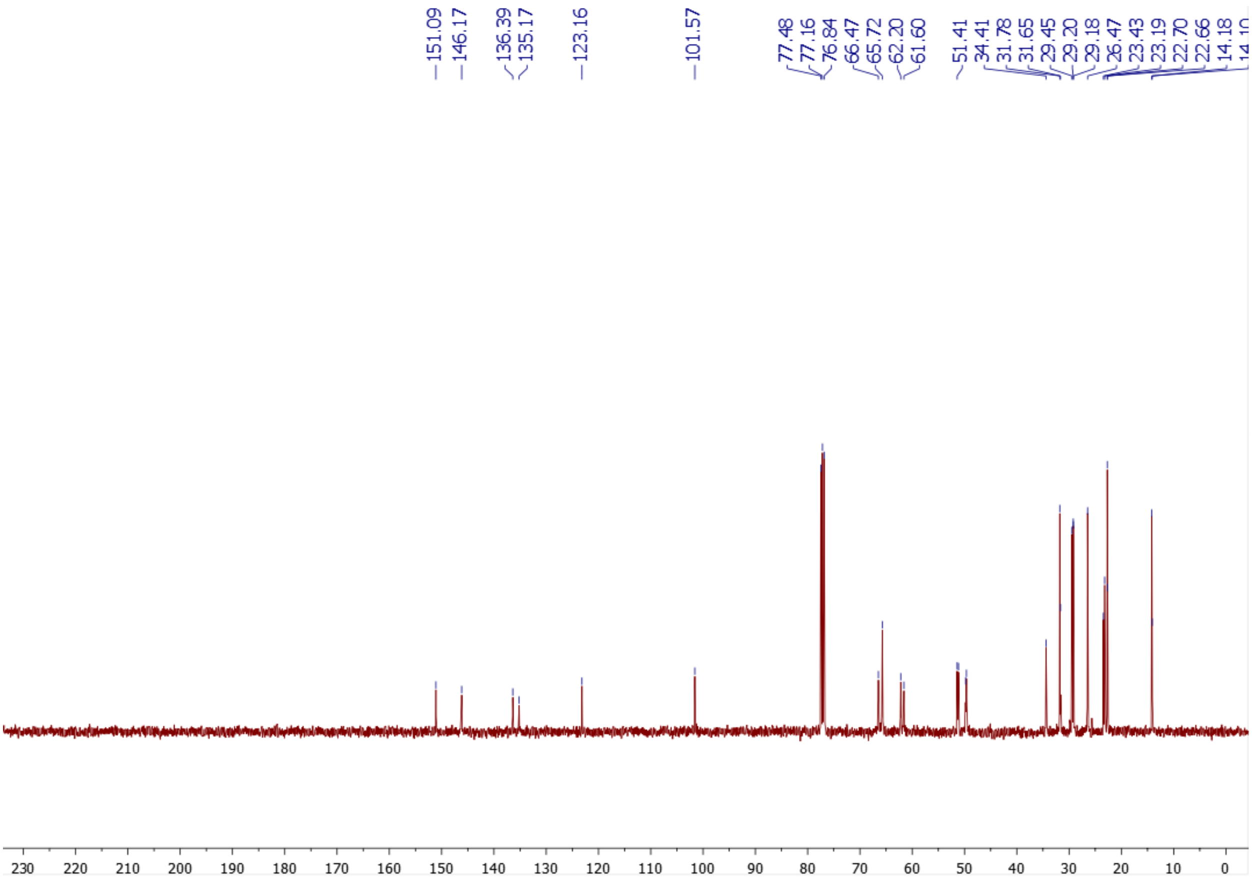

$^{13}C\{H\}$ NMR spectrum of compound **5i₈**

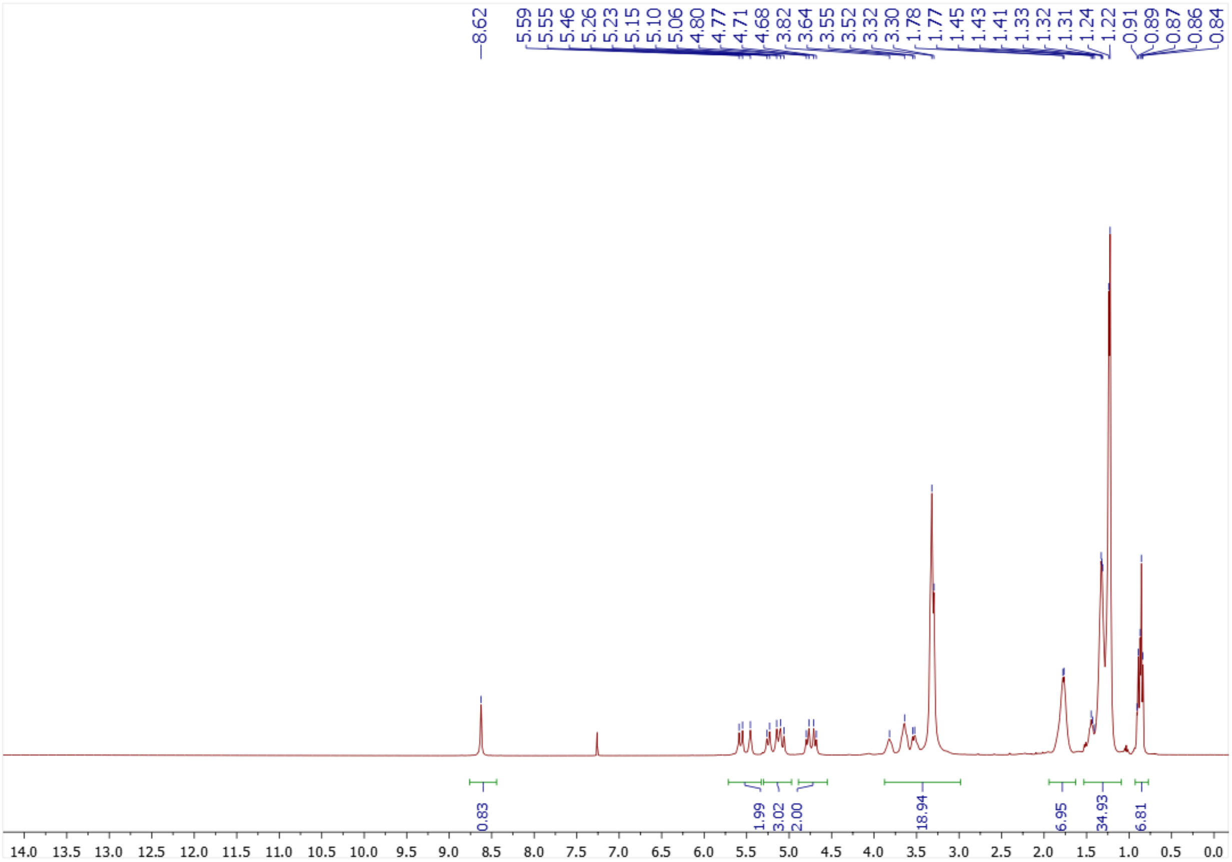

^{1}H NMR spectrum of compound **5i**$_{10}$

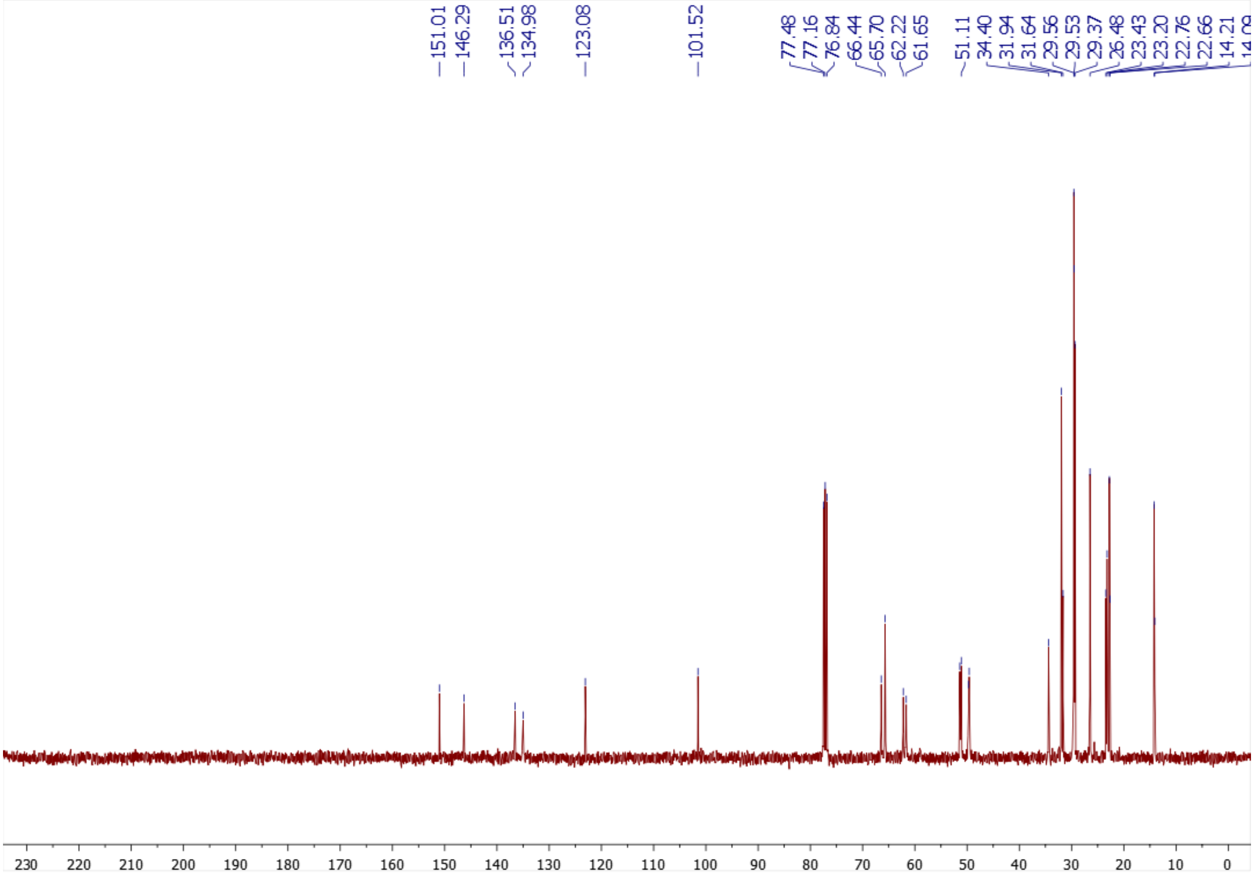

^{13}C{H} NMR spectrum of compound **5i**$_{10}$

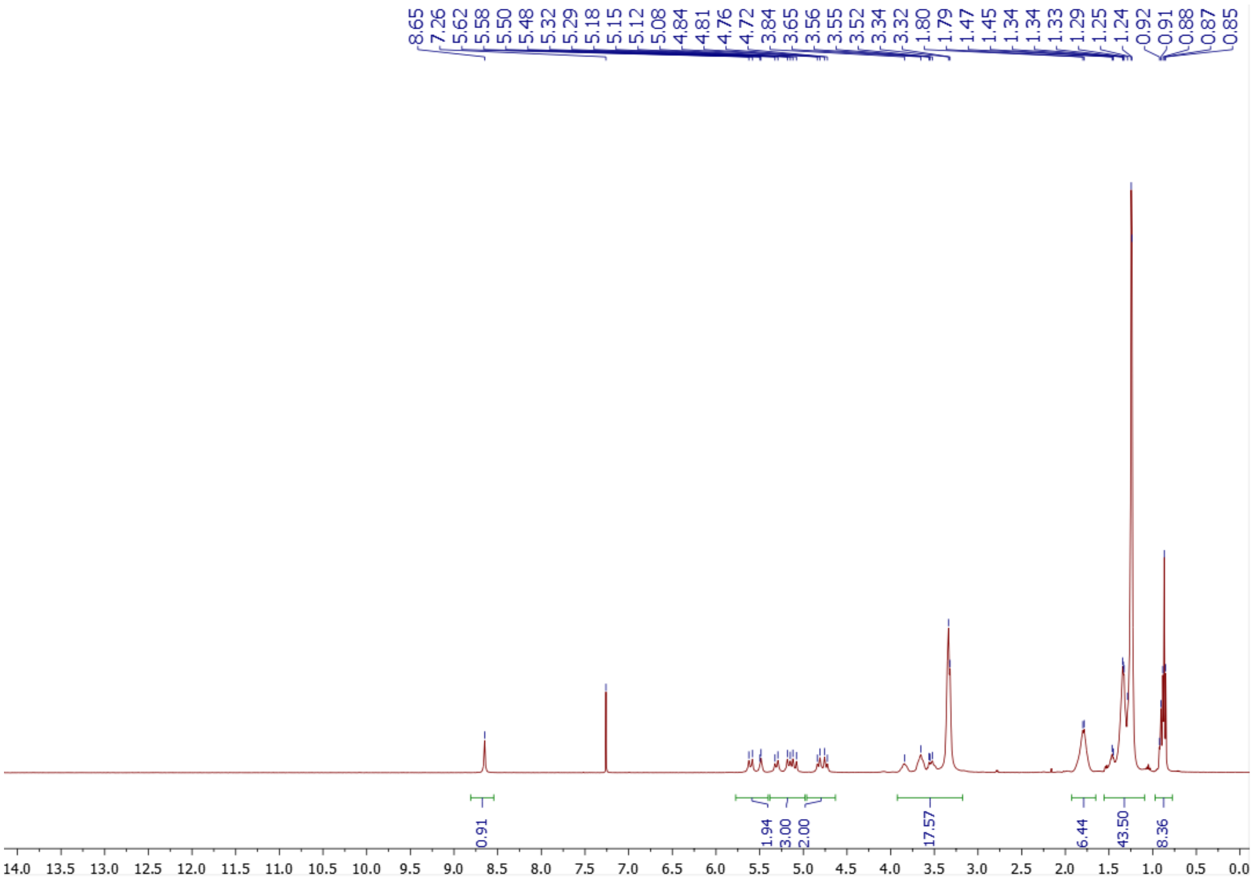

^{1}H NMR spectrum of compound **5i**12

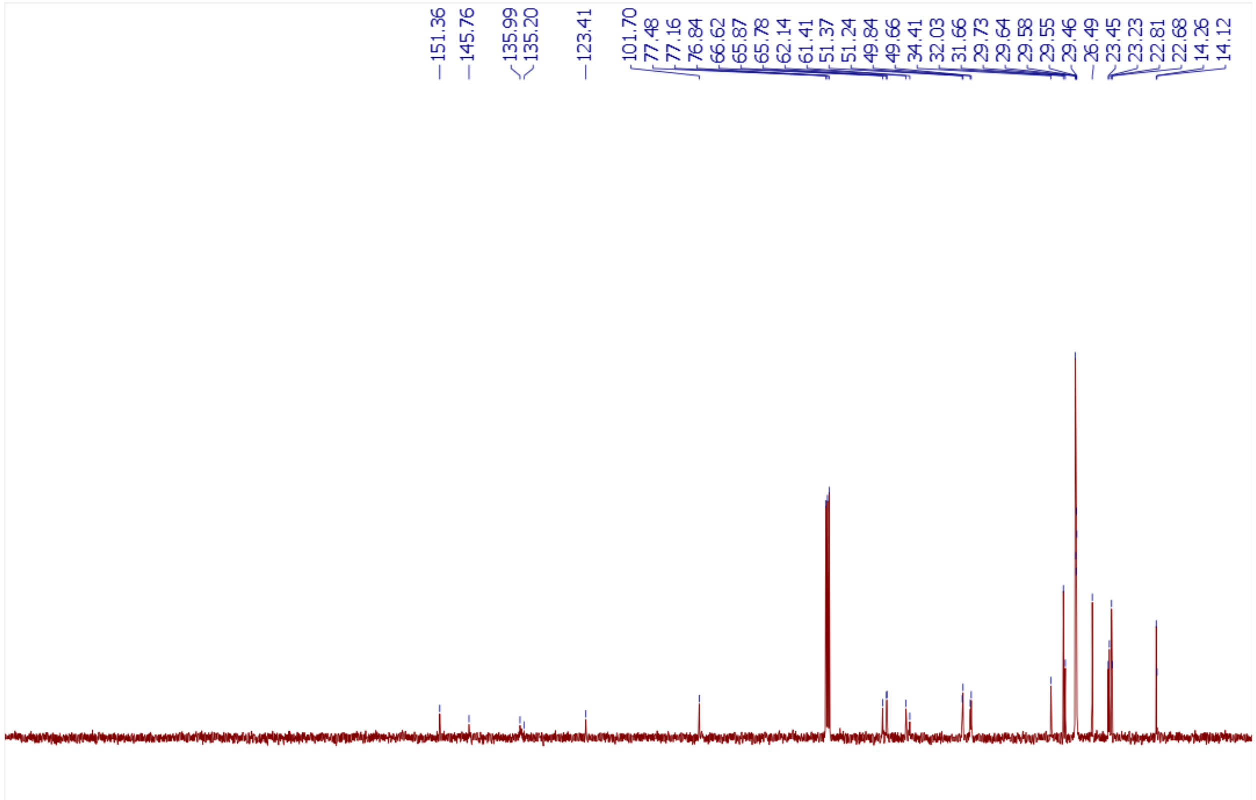

^{13}C{H} NMR spectrum of compound **5i**12

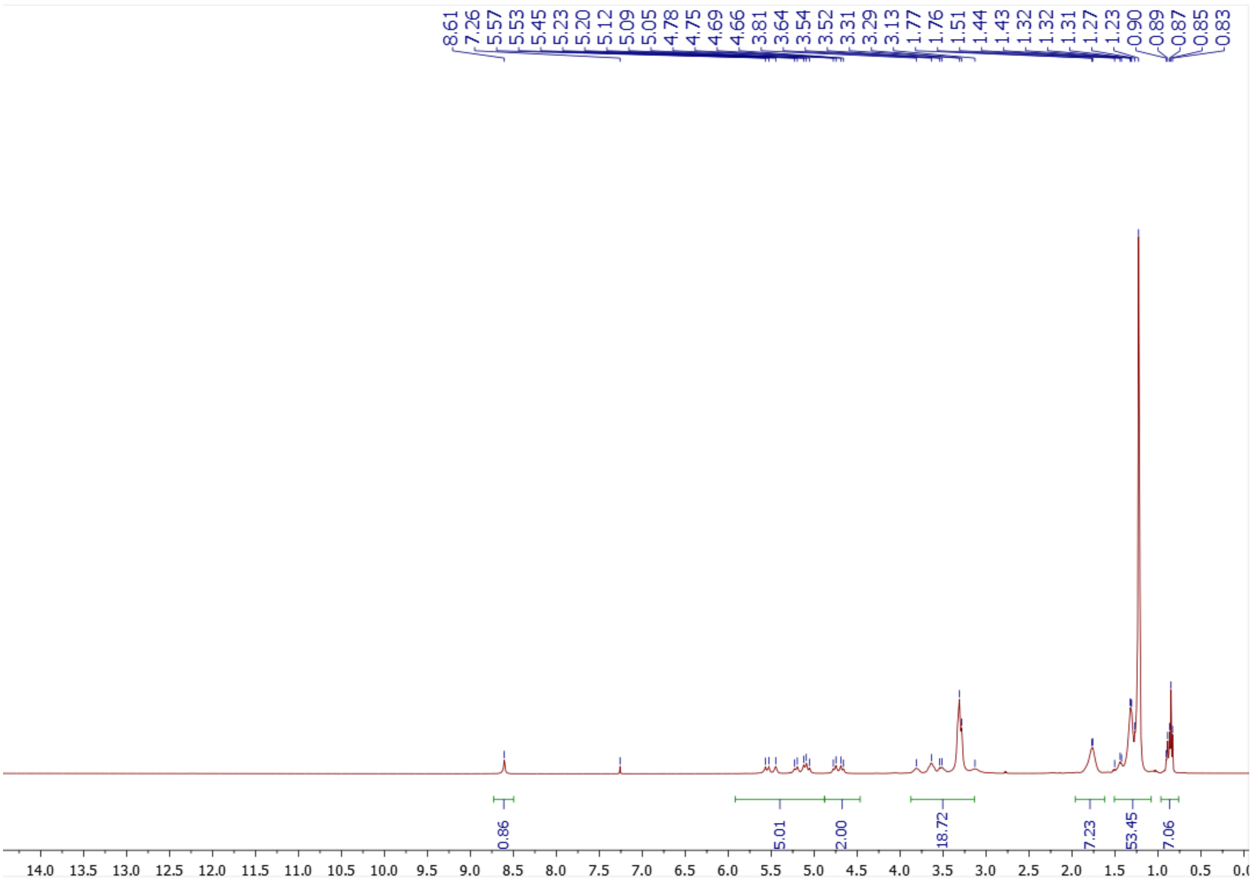

^{1}H NMR spectrum of compound **5i14**

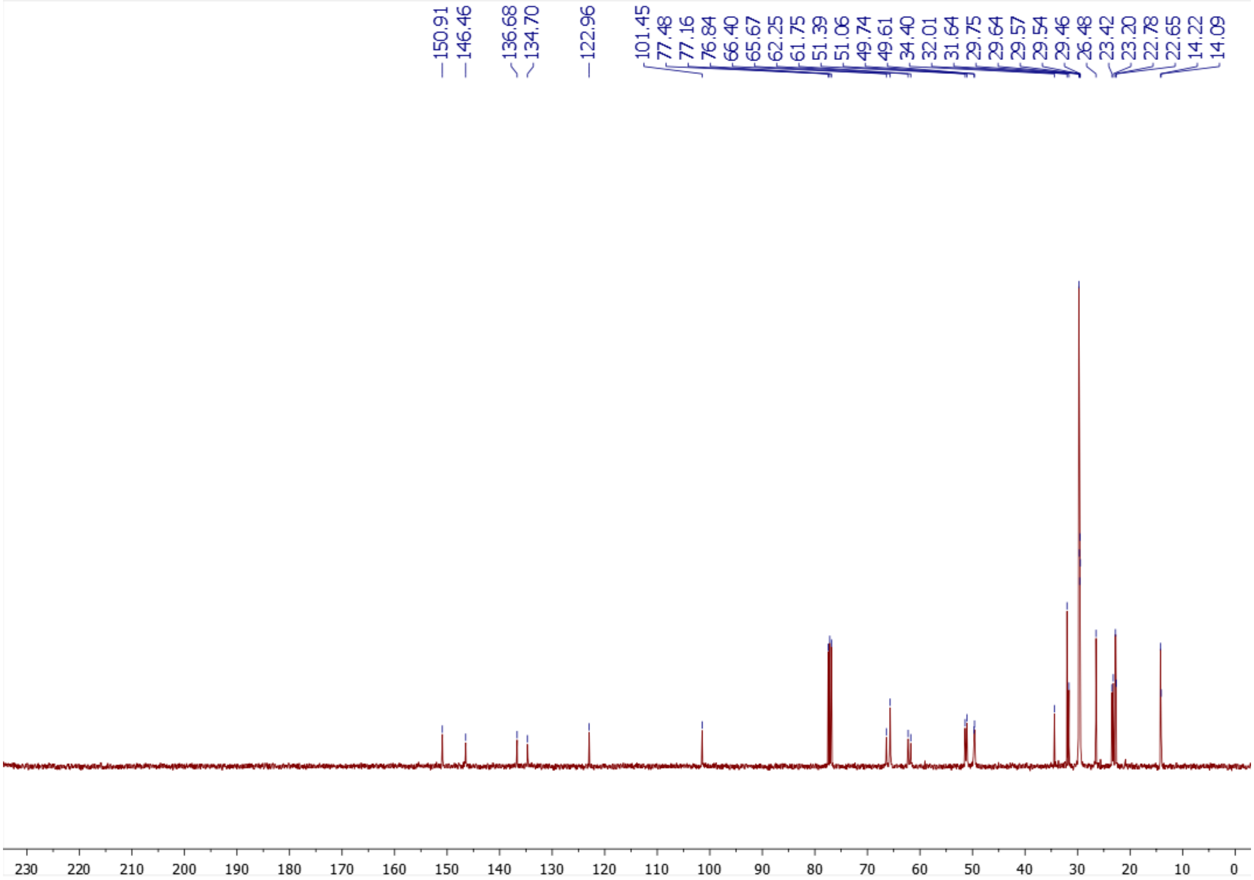

^{13}C{H} NMR spectrum of compound **5i14**

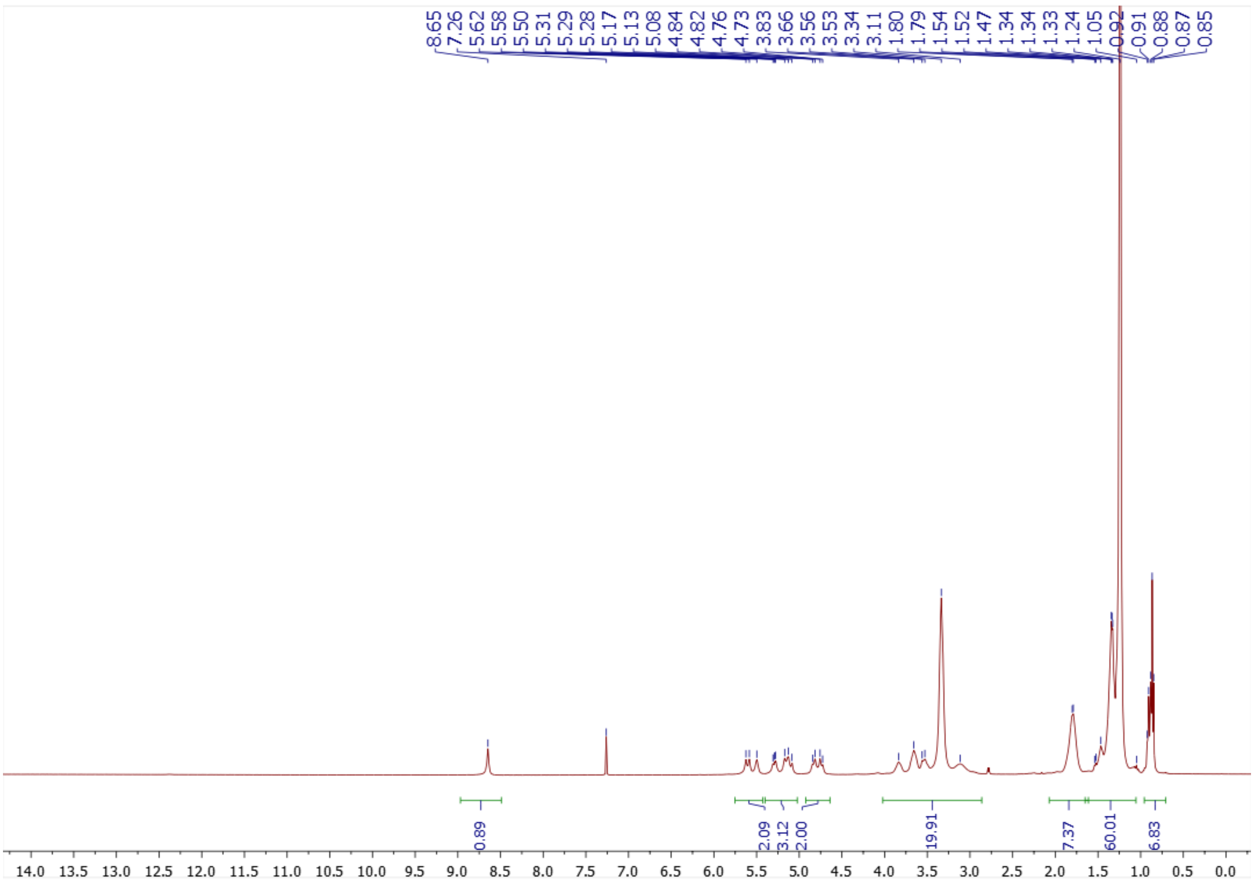

^{1}H NMR spectrum of compound **5i16**

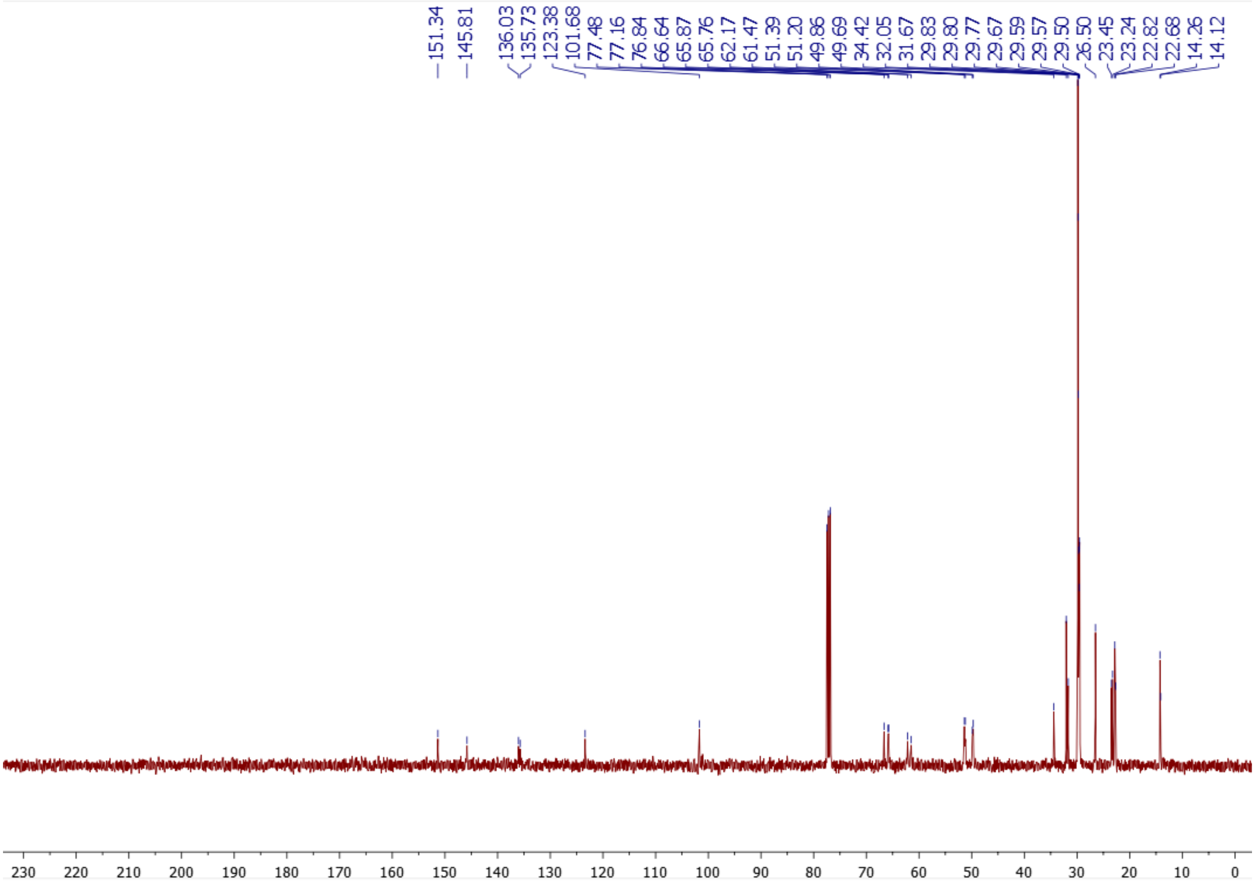

^{13}C{H} NMR spectrum of compound **5i16**

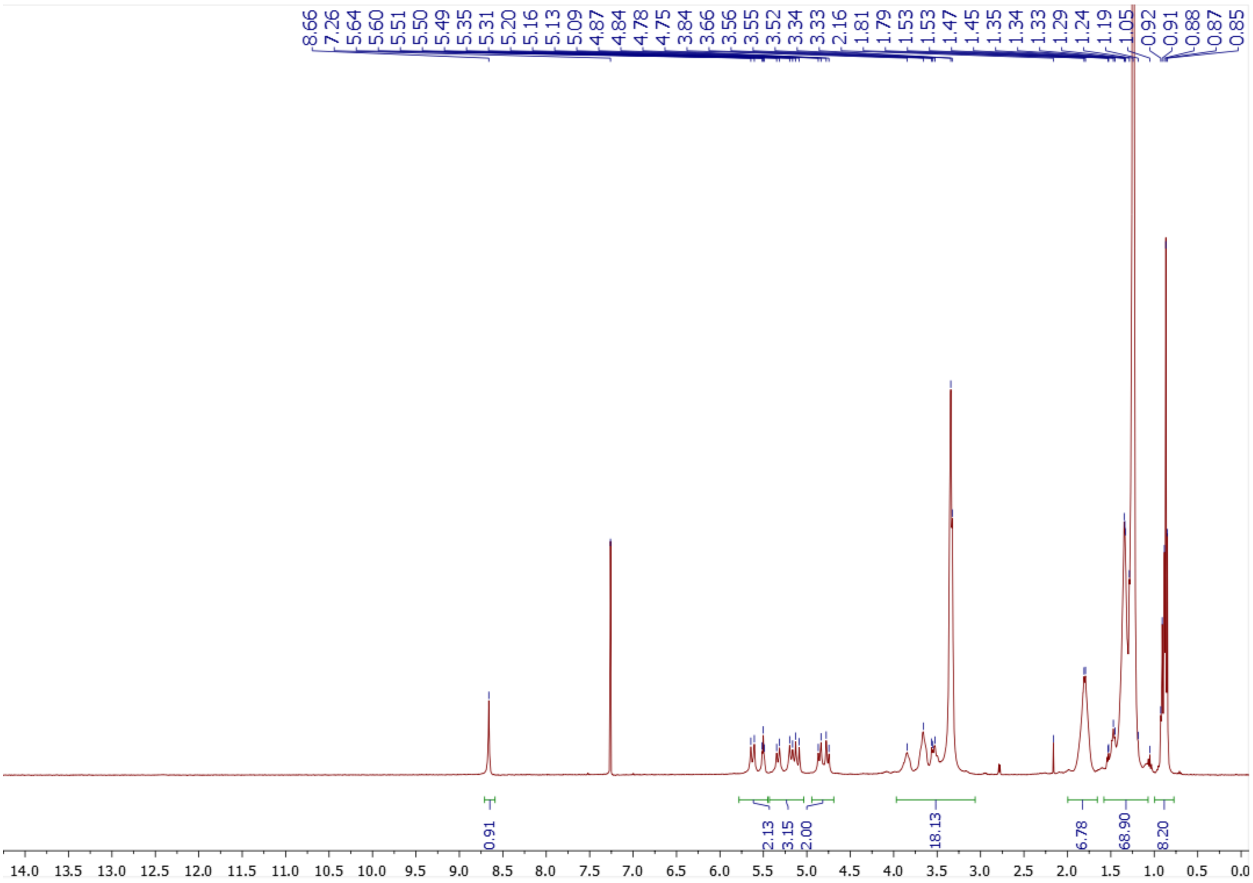

^{1}H NMR spectrum of compound **5i**$_{18}$

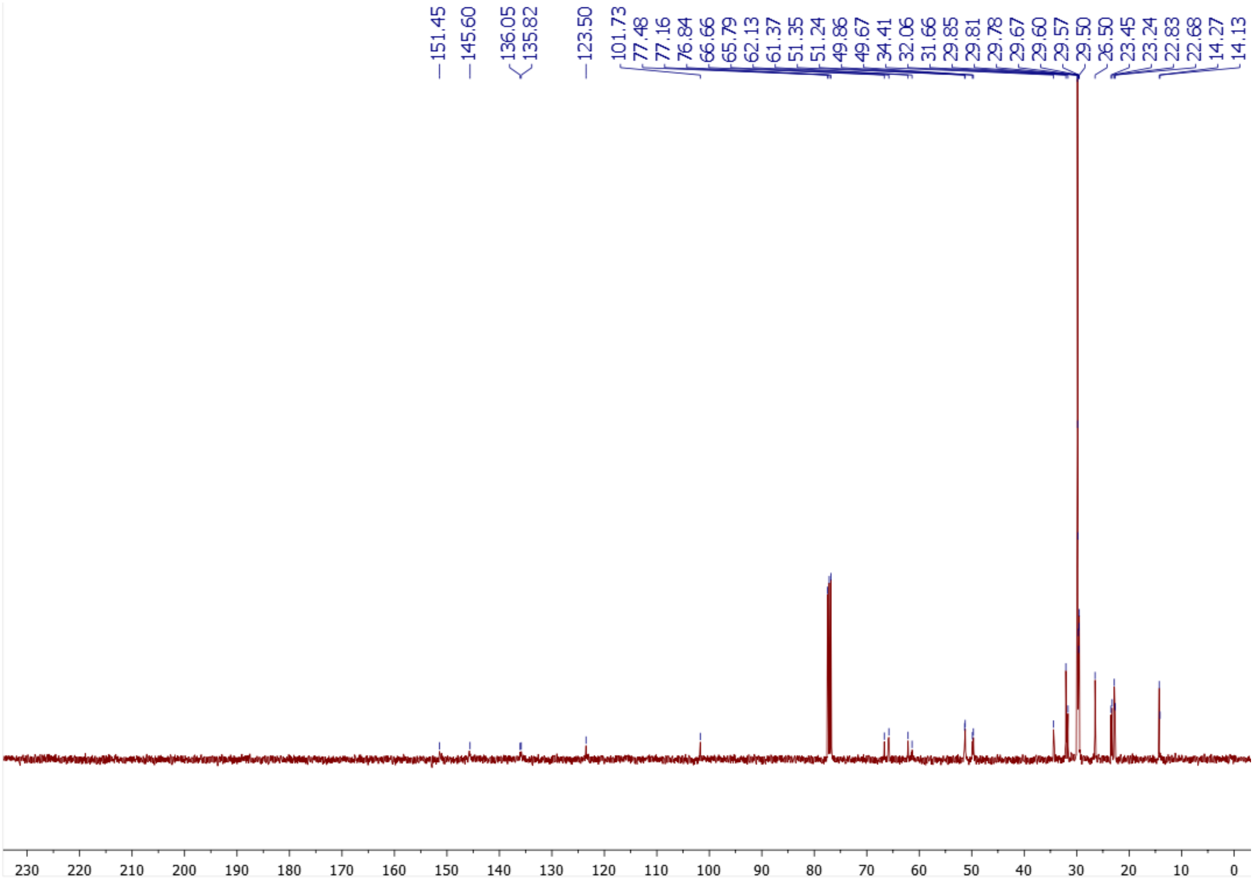

^{13}C{H} NMR spectrum of compound **5i**$_{18}$

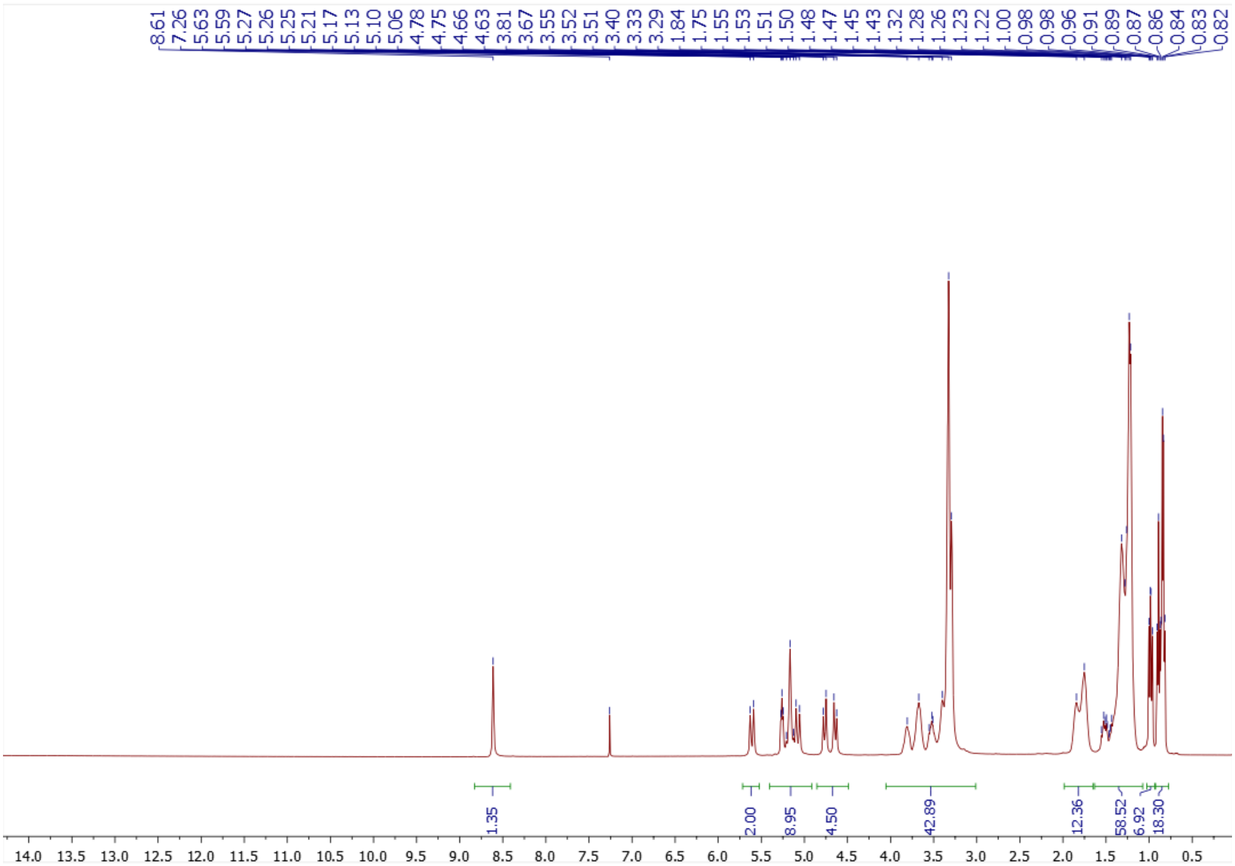

^{1}H NMR spectrum of compound **5j8**

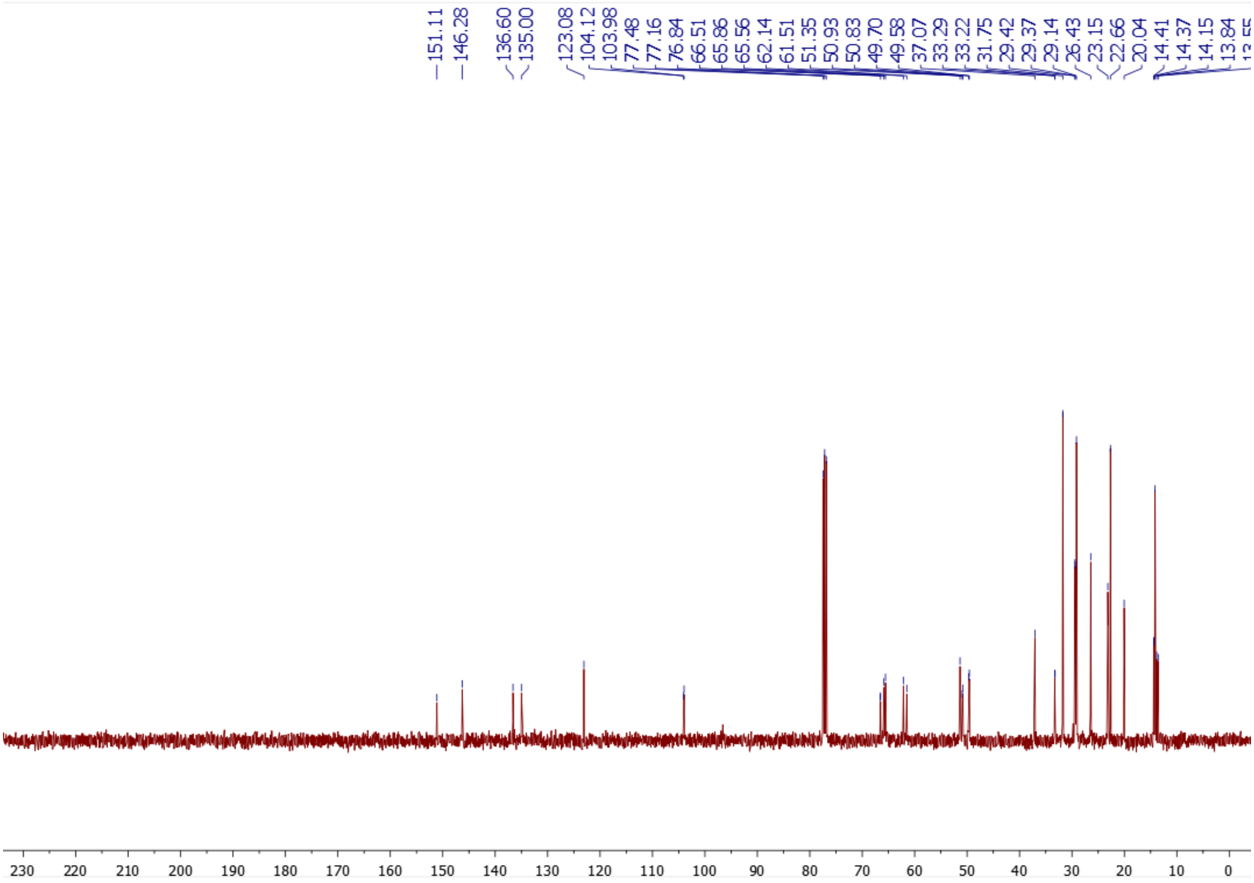

^{13}C{H} NMR spectrum of compound **5j8**

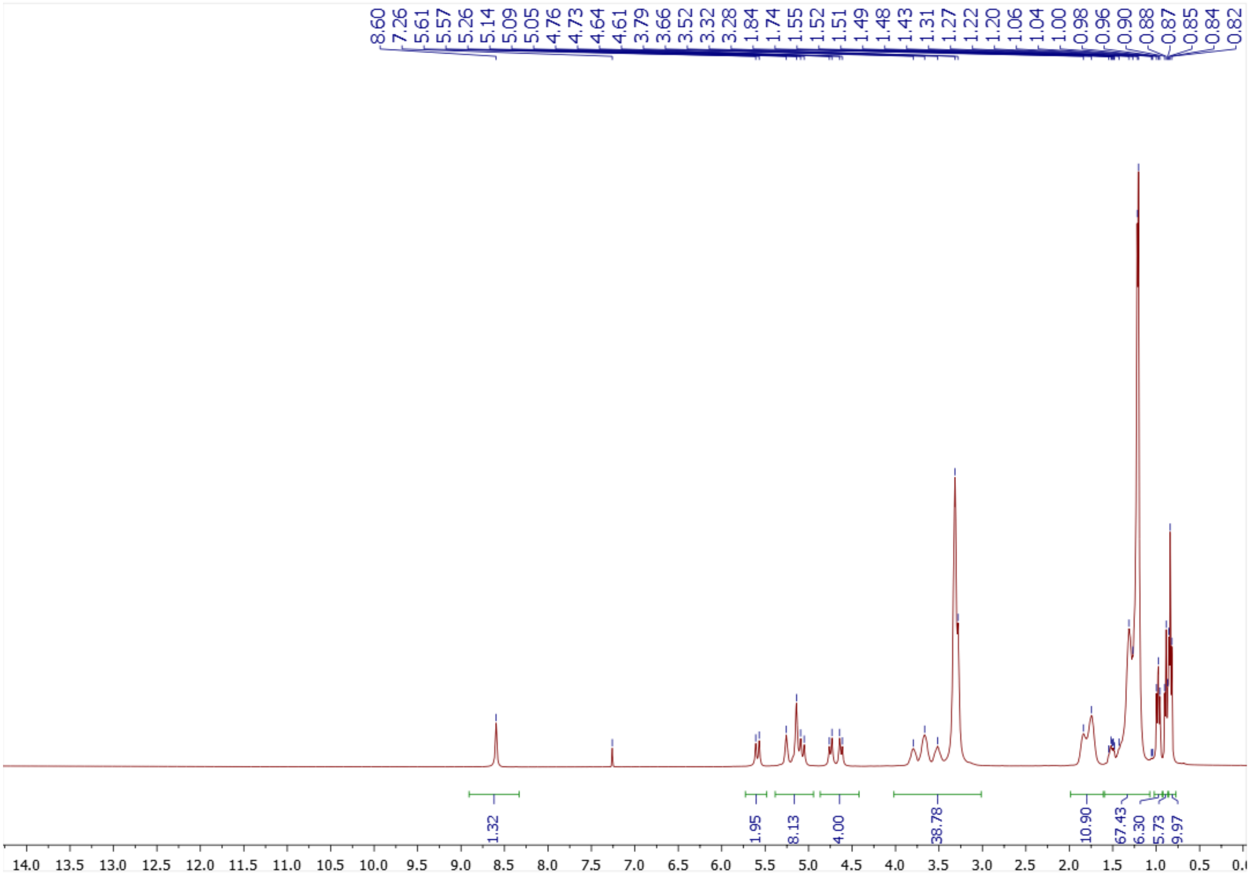

¹H NMR spectrum of compound **5j₁₀**

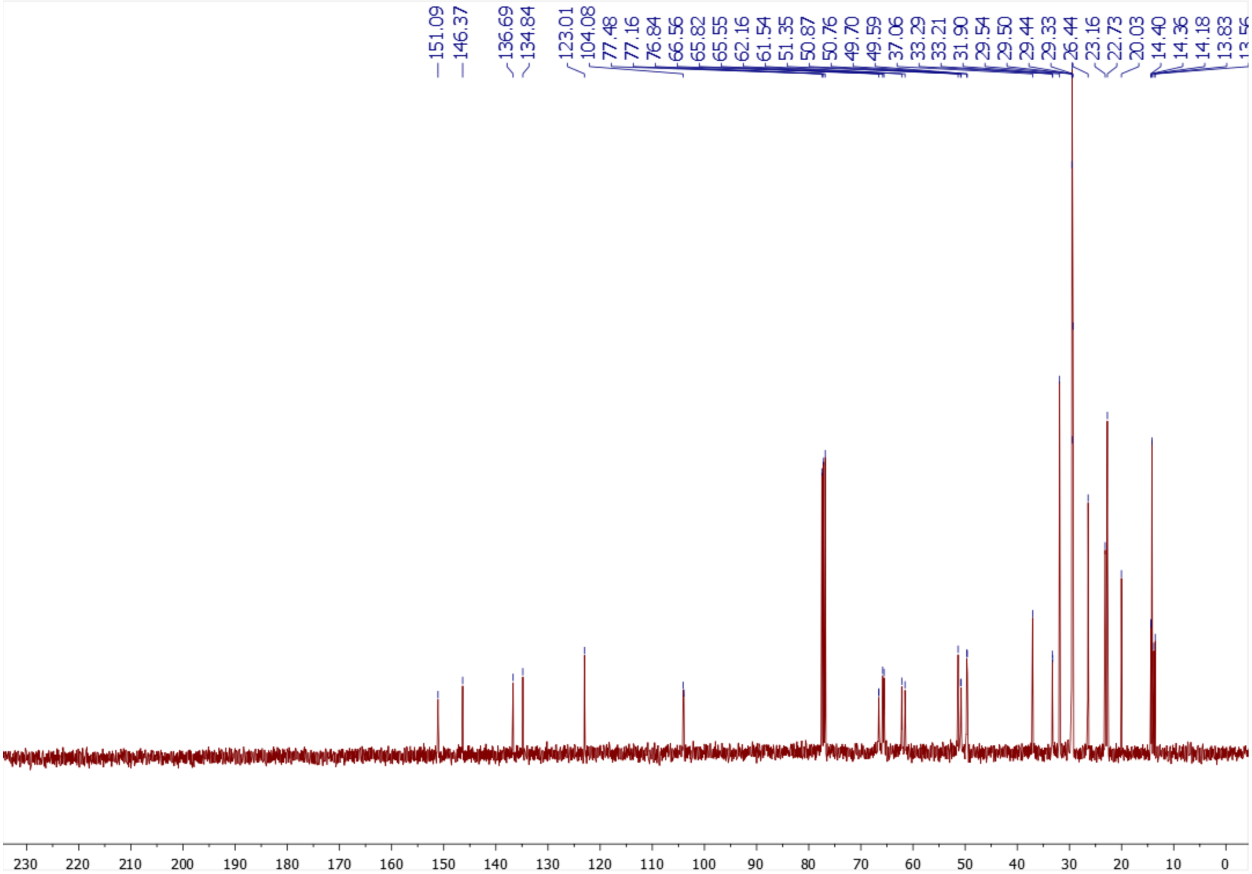

$^{13}C\{H\}$ NMR spectrum of compound **5j₁₀**

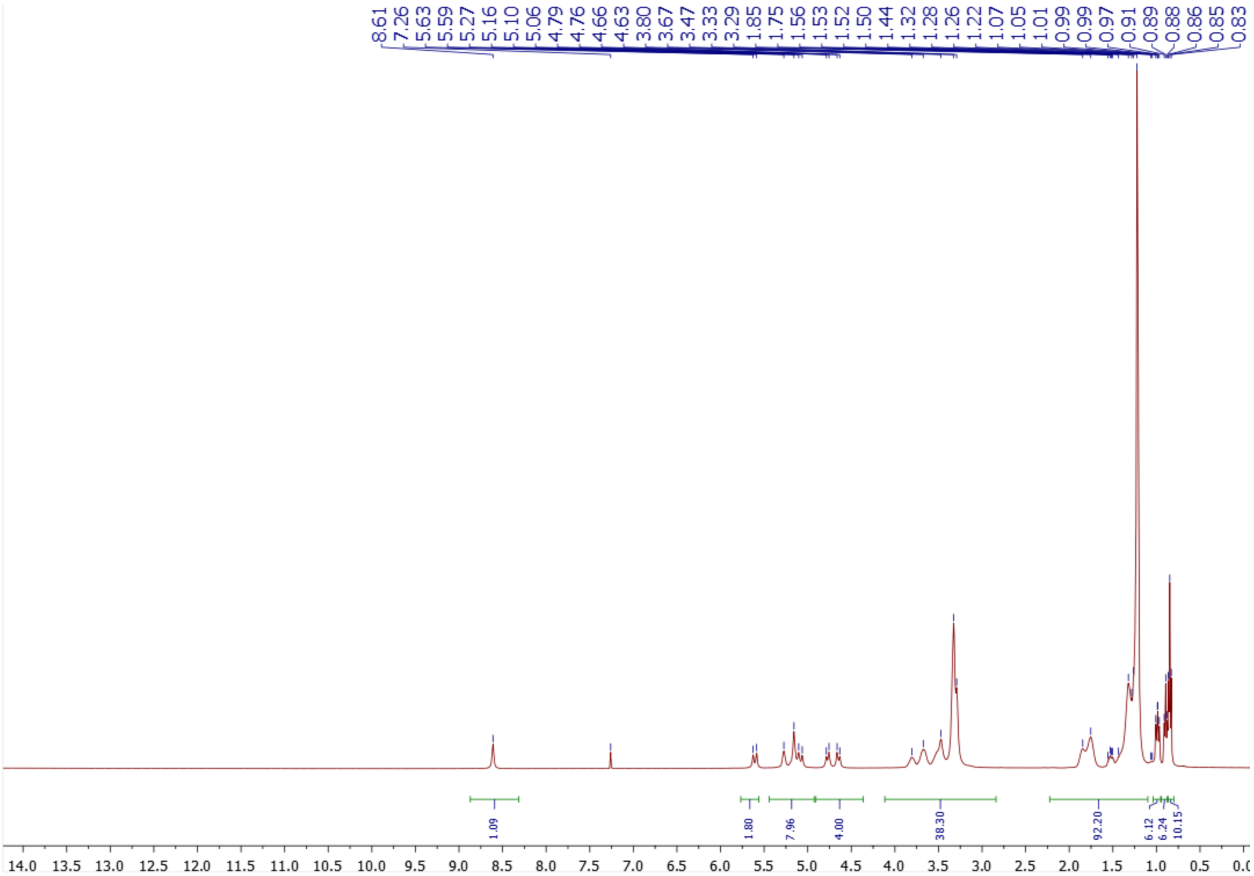

^{1}H NMR spectrum of compound **5j**12

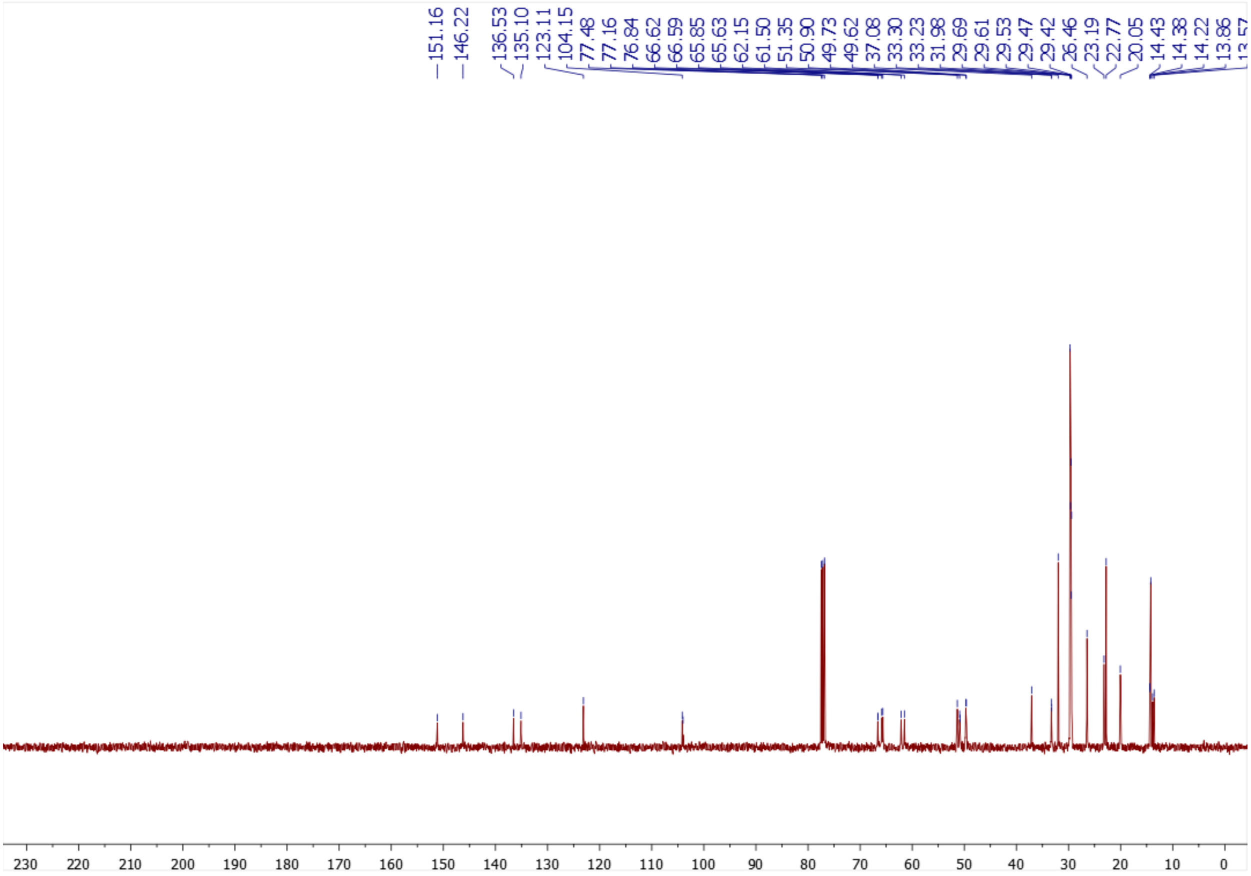

^{13}C{H} NMR spectrum of compound **5j**12

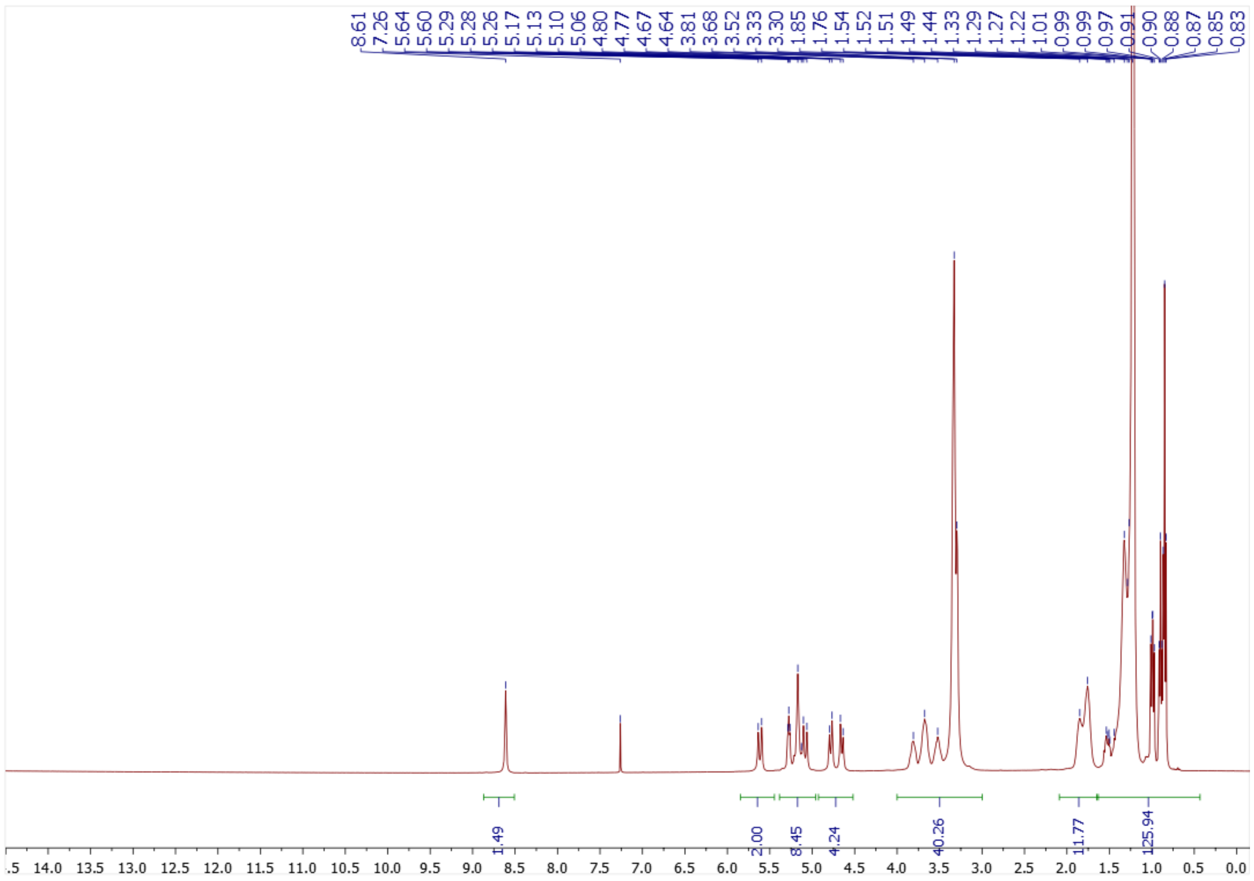

^{1}H NMR spectrum of compound **5j**$_{14}$

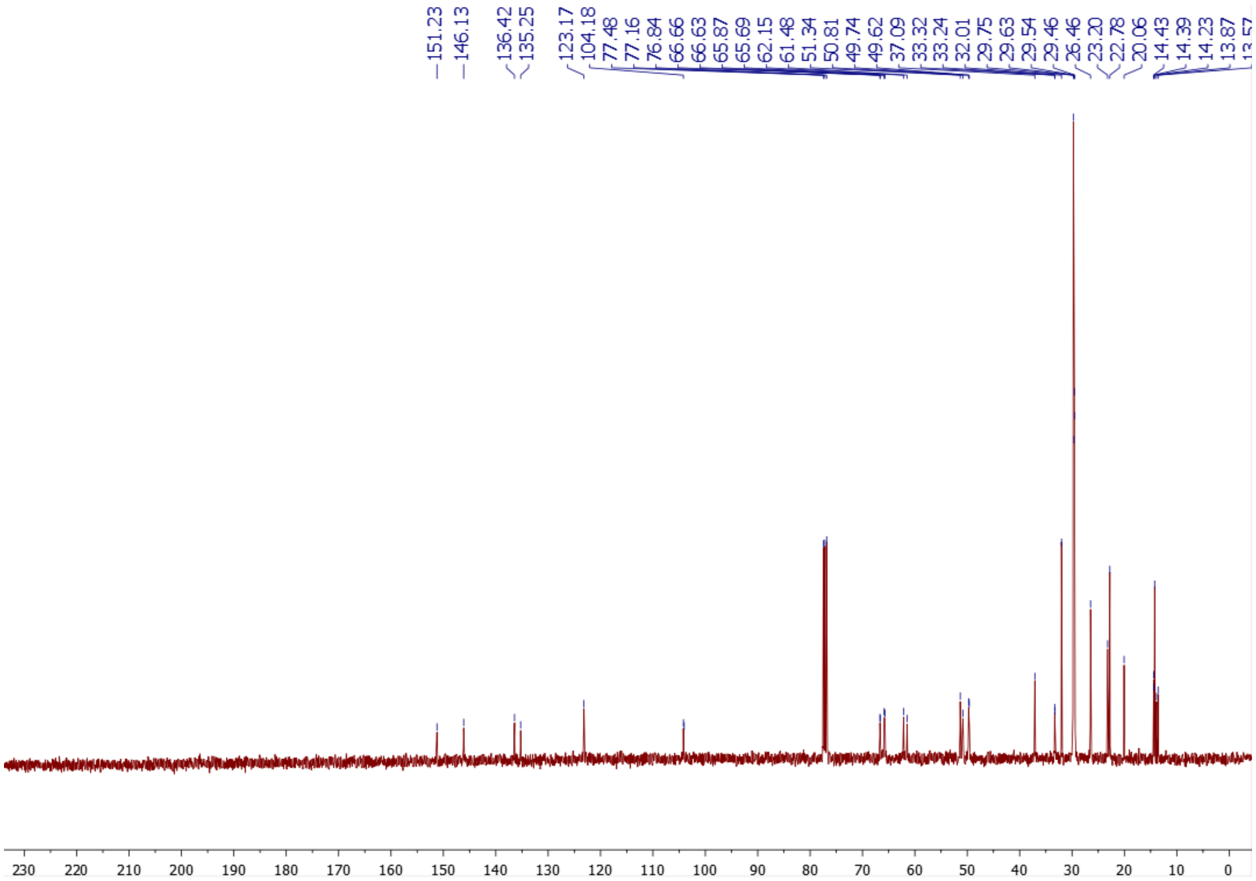

$^{13}C\{H\}$ NMR spectrum of compound **5j**$_{14}$

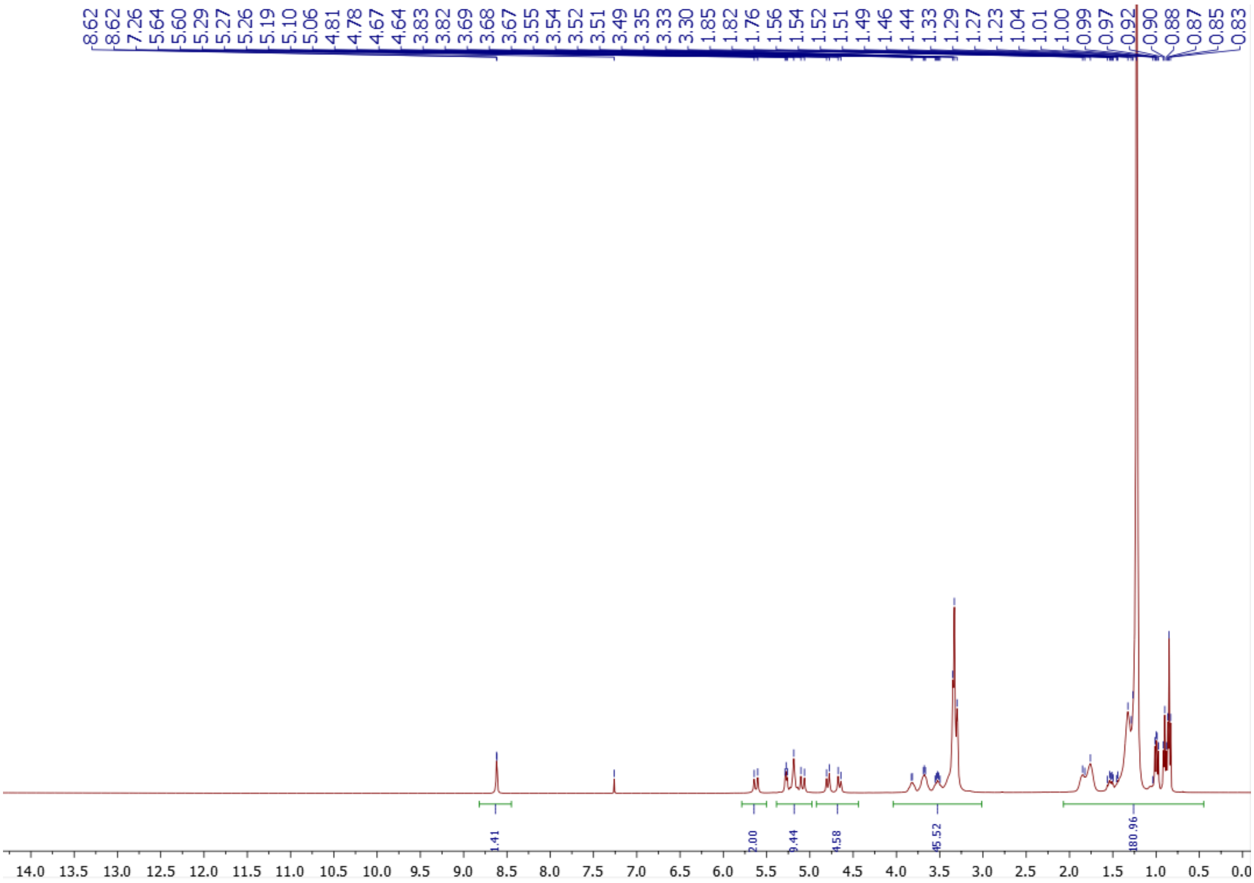

^{1}H NMR spectrum of compound **5j**16

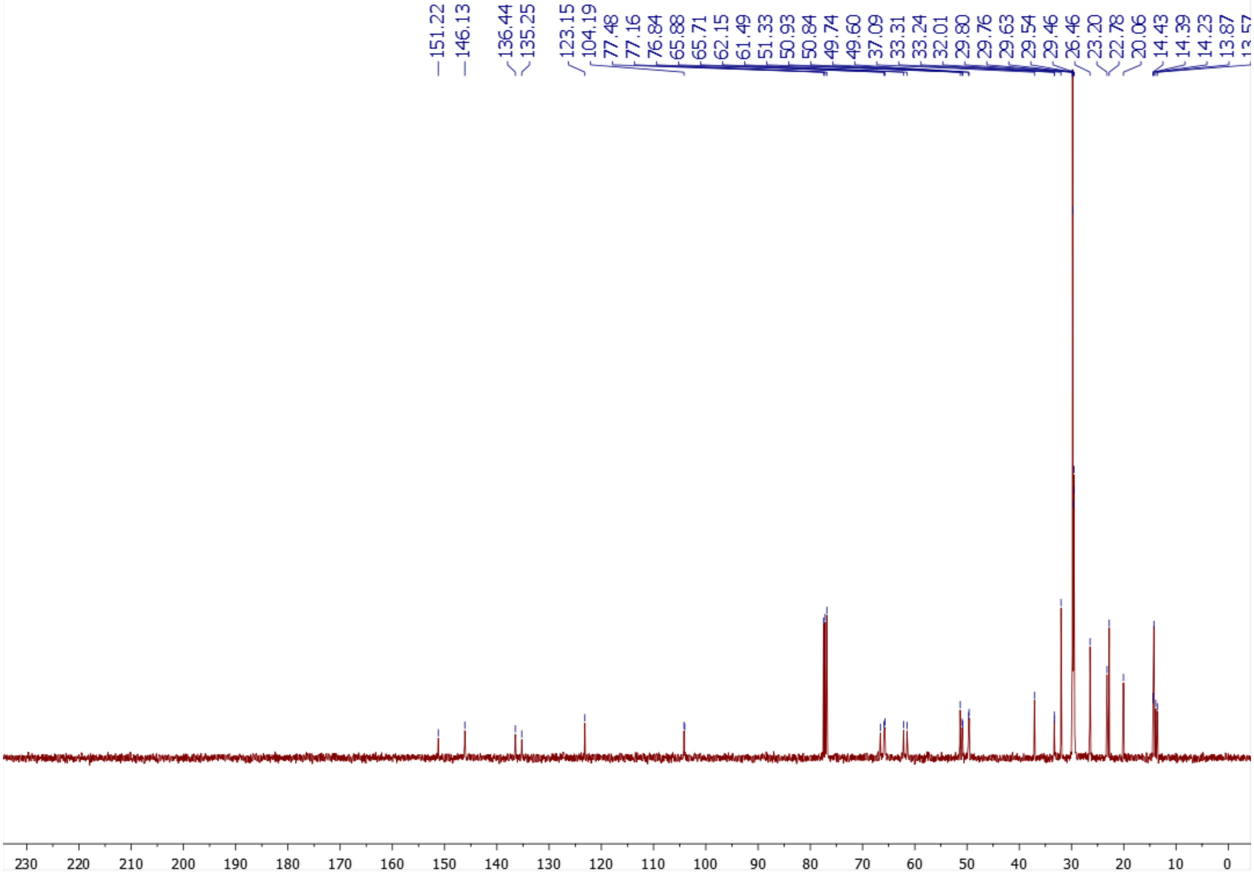

^{13}C{H} NMR spectrum of compound **5j**16

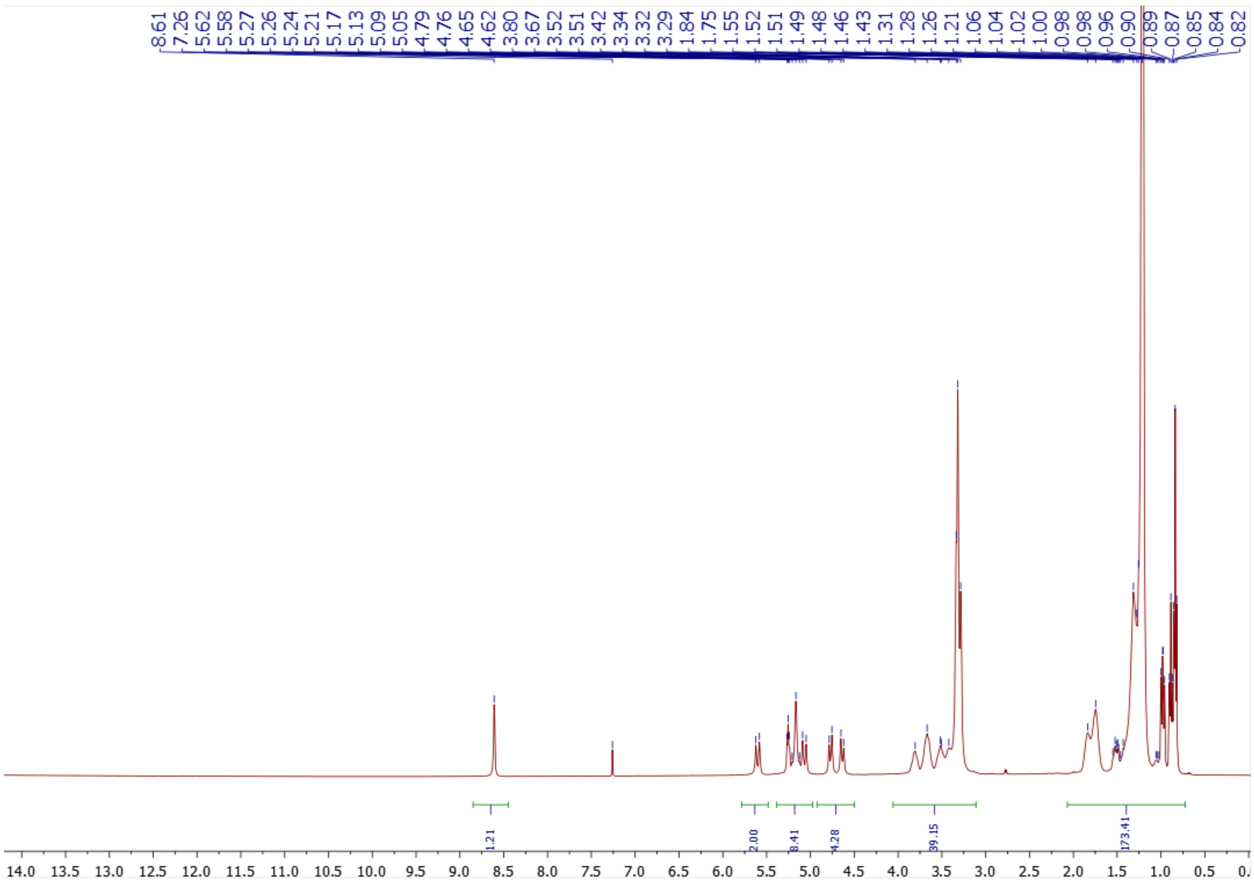

^{1}H NMR spectrum of compound **5j**18

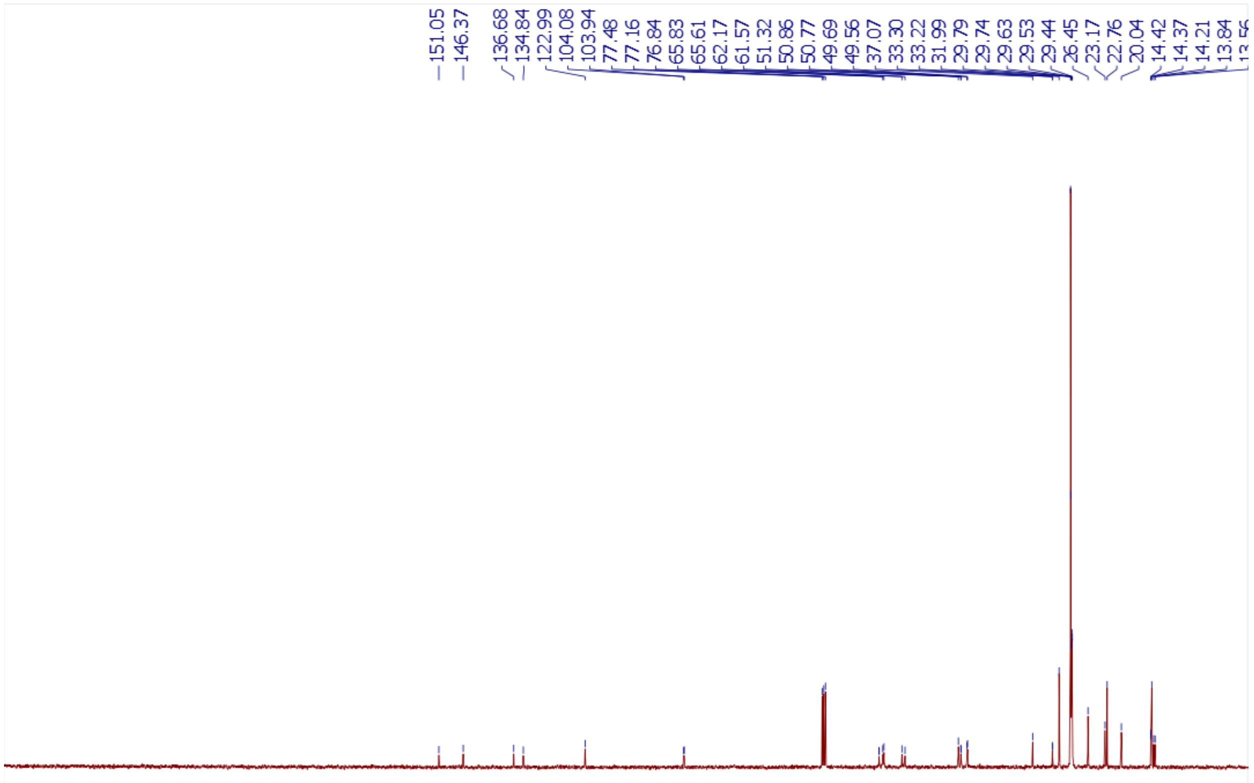

^{13}C{H} NMR spectrum of compound **5j**18

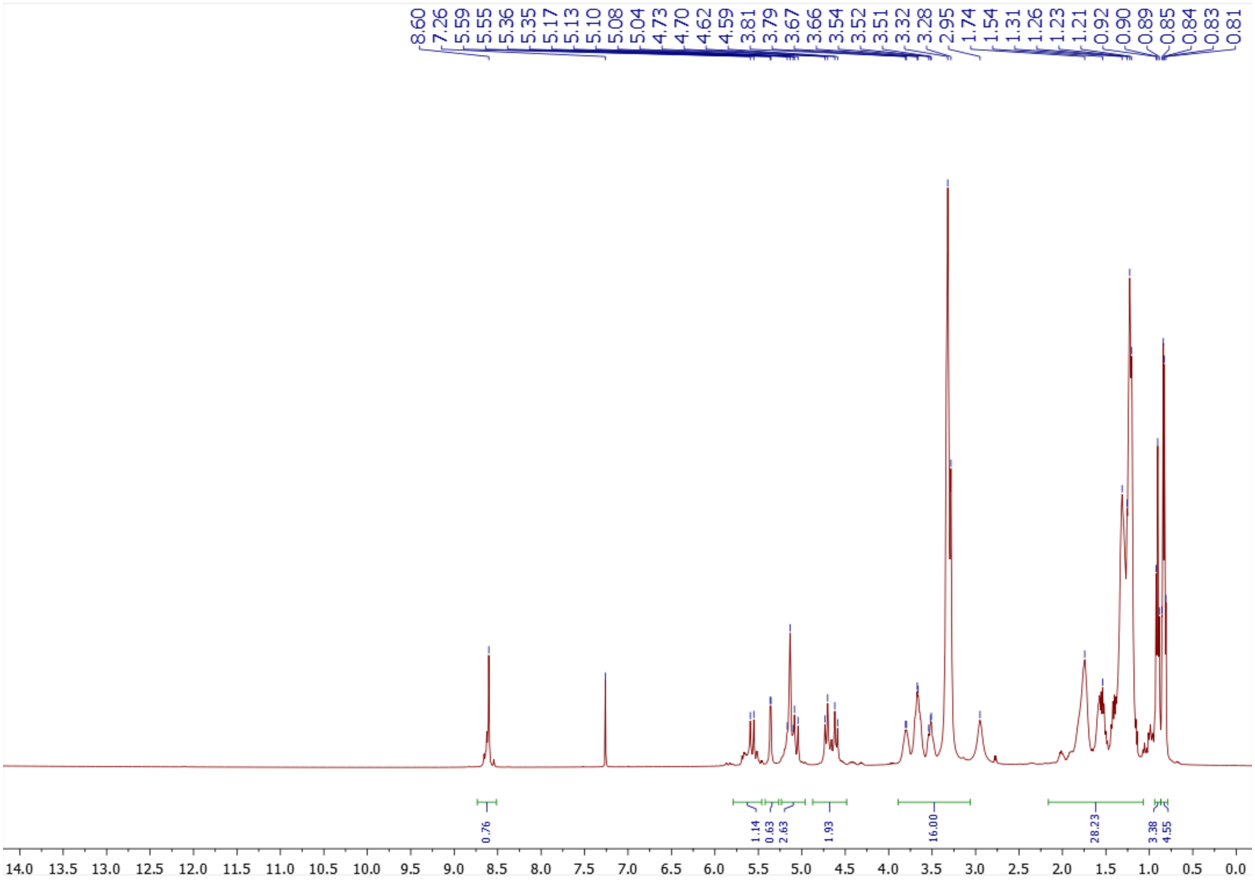

^{1}H NMR spectrum of compound **5k**$_8$

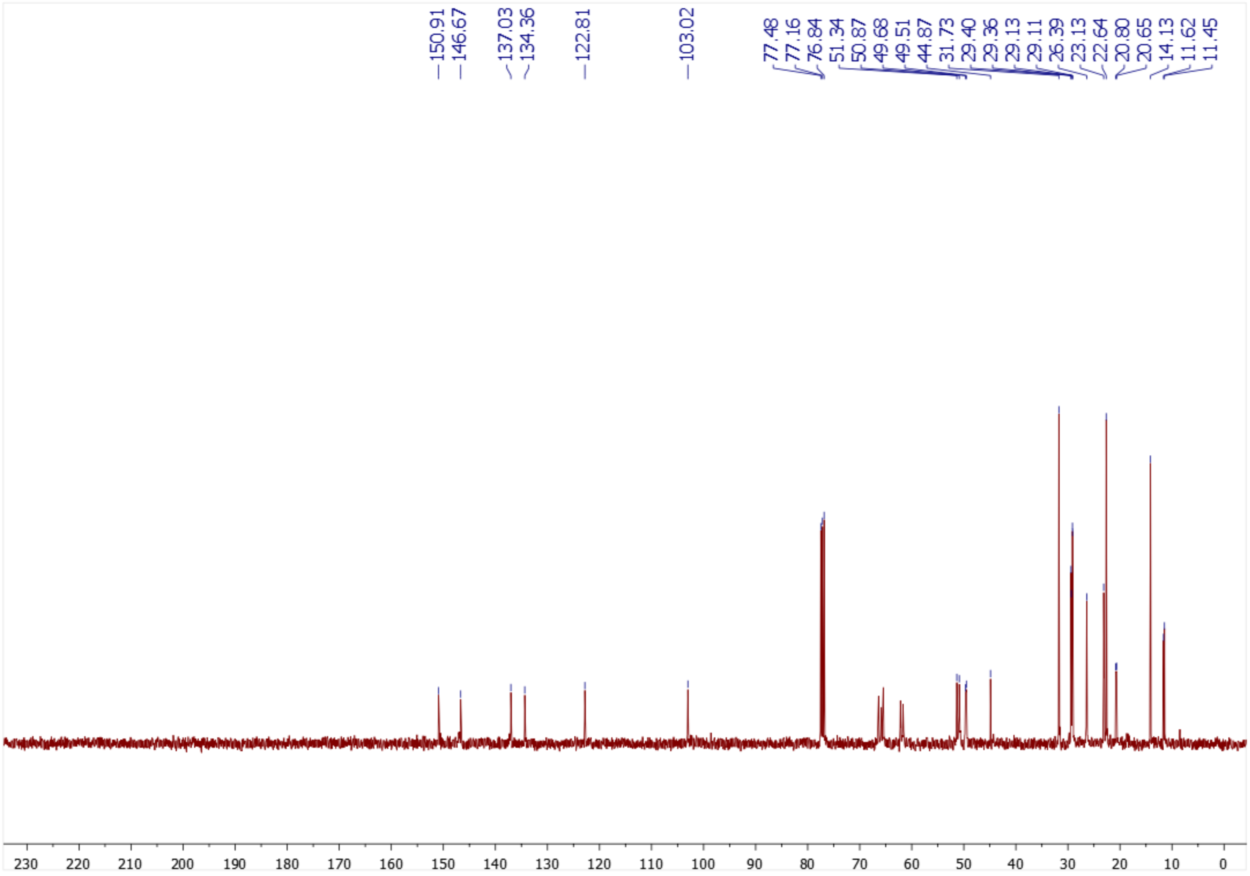

^{13}C{H} NMR spectrum of compound **5k**$_8$

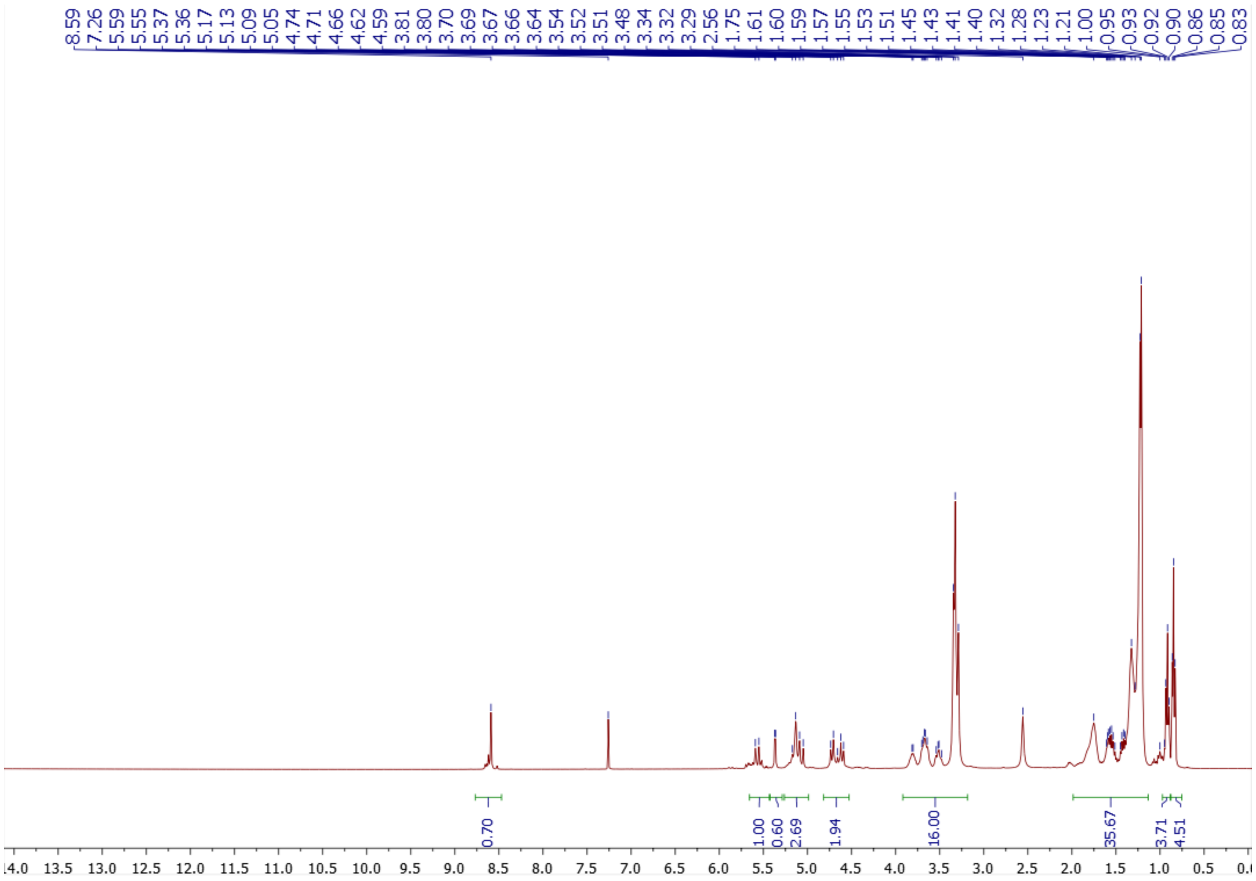

^{1}H NMR spectrum of compound **5k**$_{10}$

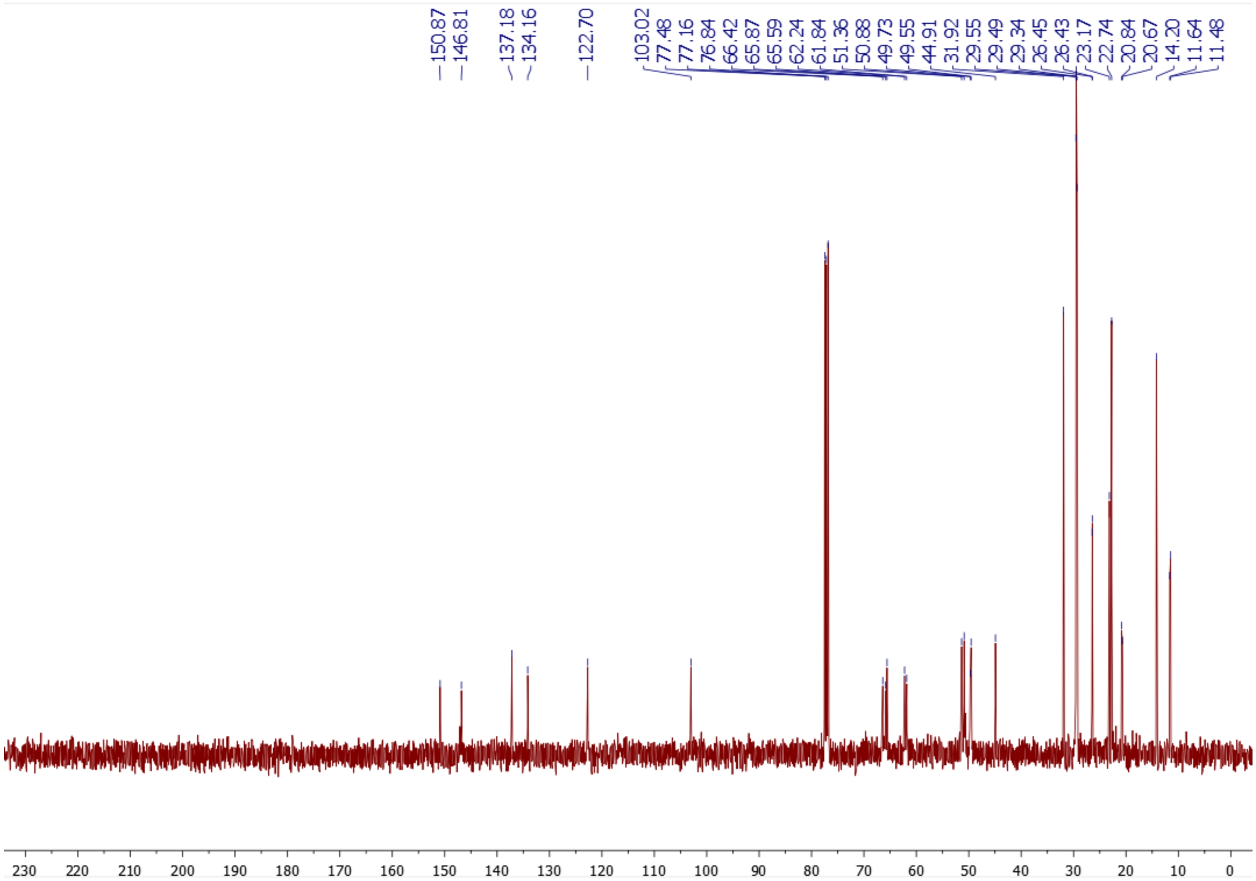

^{13}C{H} NMR spectrum of compound **5k**$_{10}$

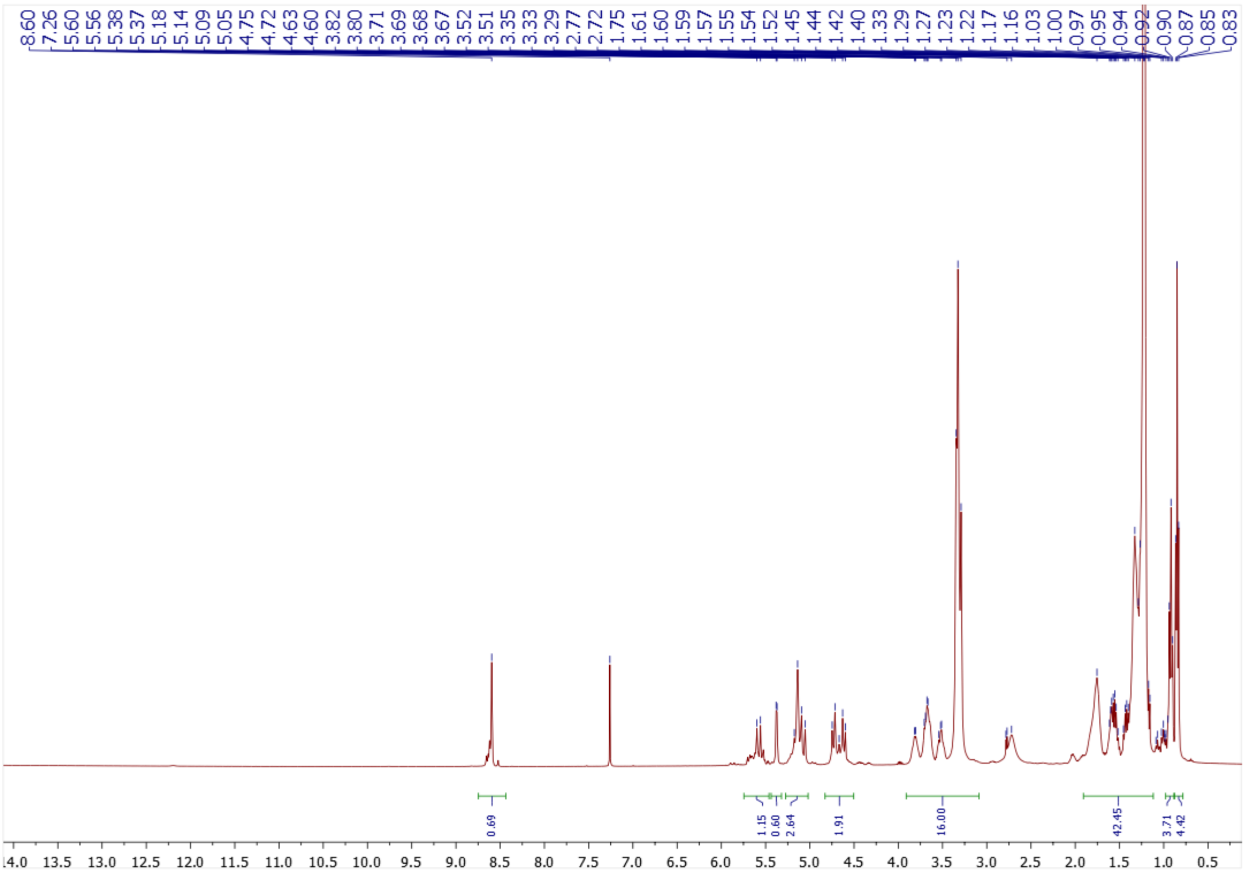

^{1}H NMR spectrum of compound **5k**$_{12}$

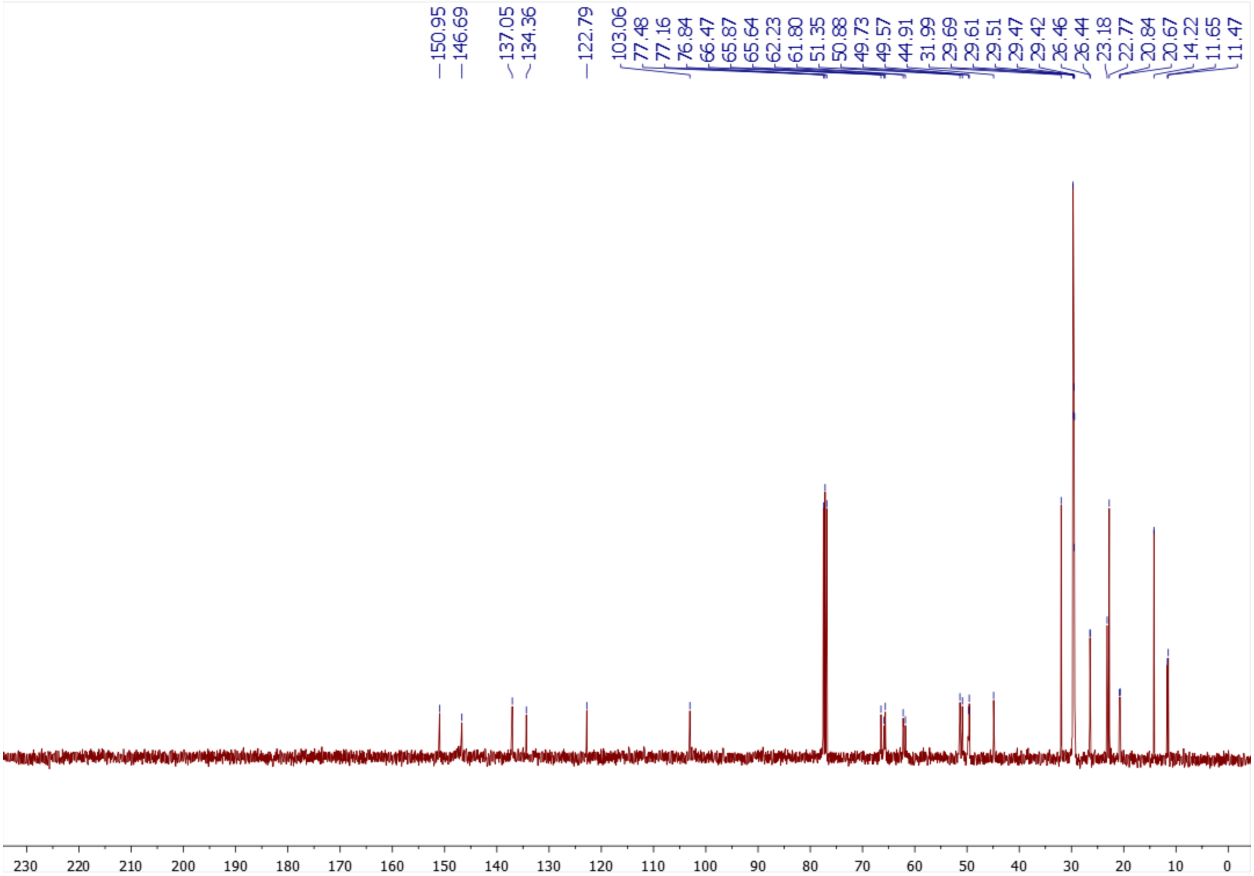

^{13}C{H} NMR spectrum of compound **5k**$_{12}$

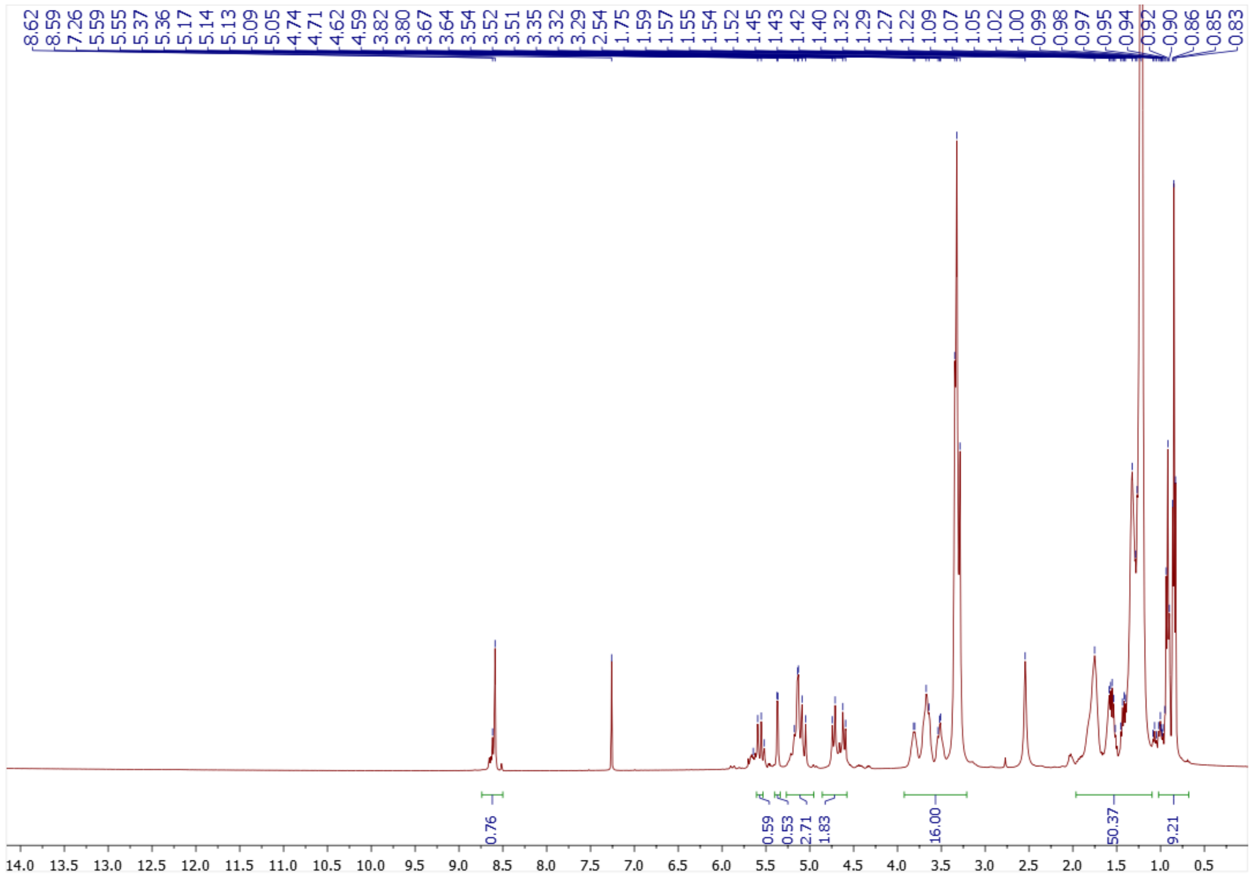

^{1}H NMR spectrum of compound **5k$_{14}$**

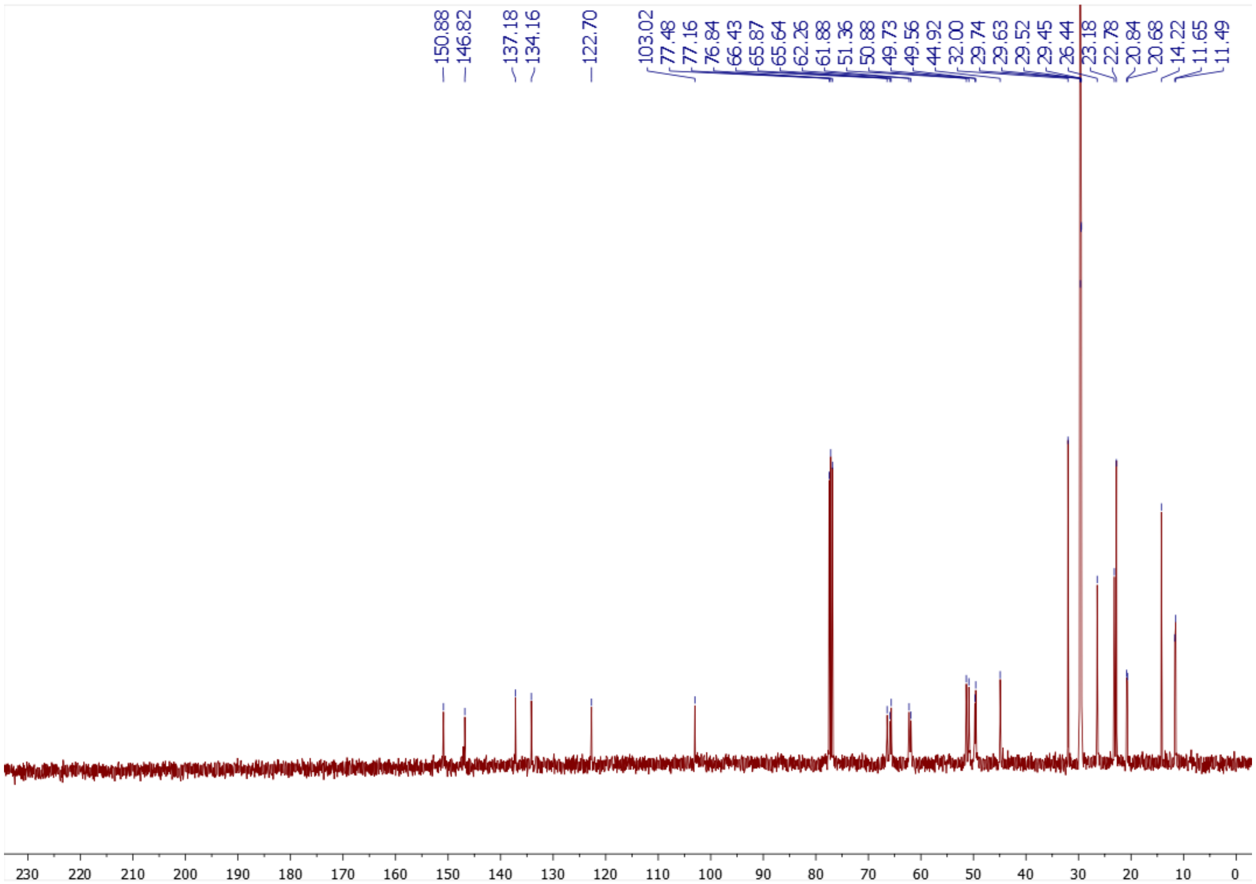

^{13}C{H} NMR spectrum of compound **5k$_{14}$**

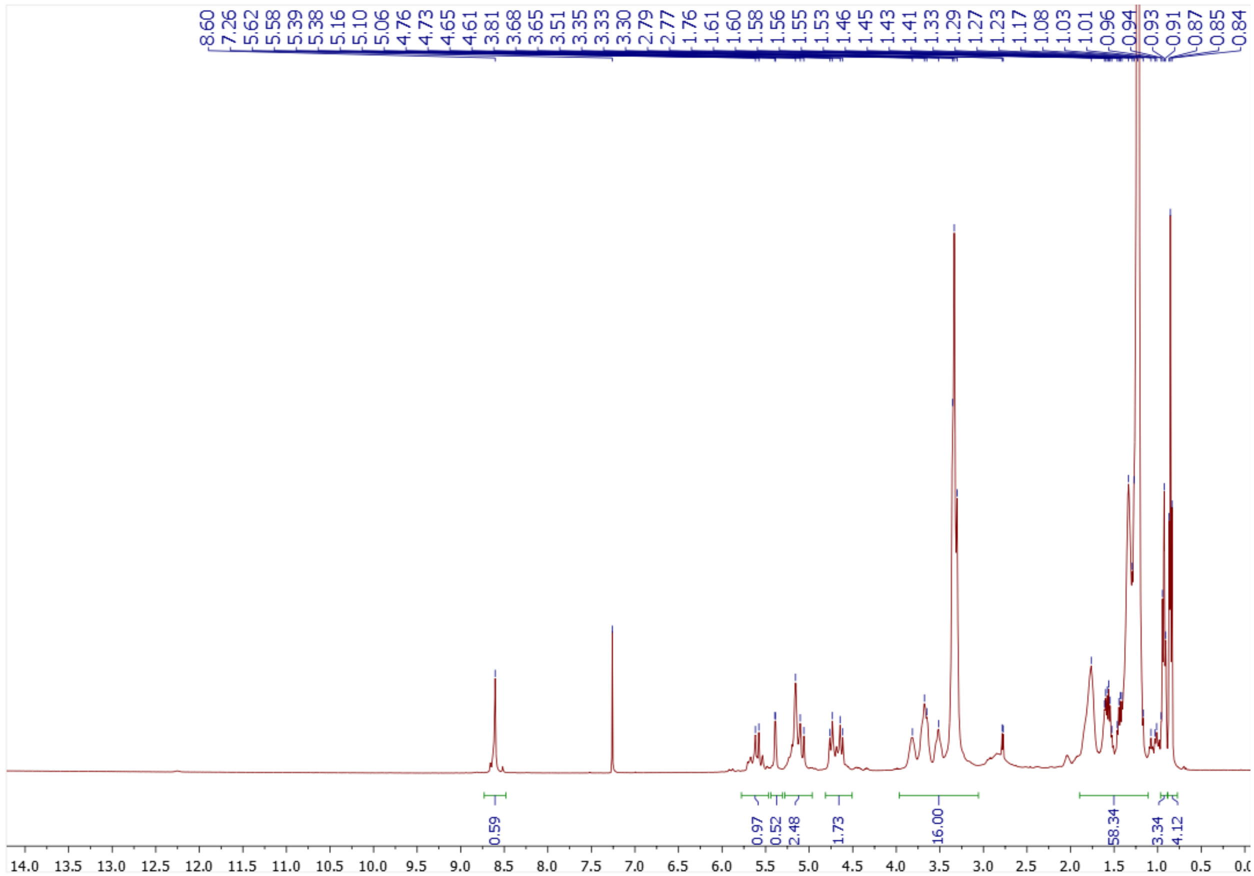

^{1}H NMR spectrum of compound **5k16**

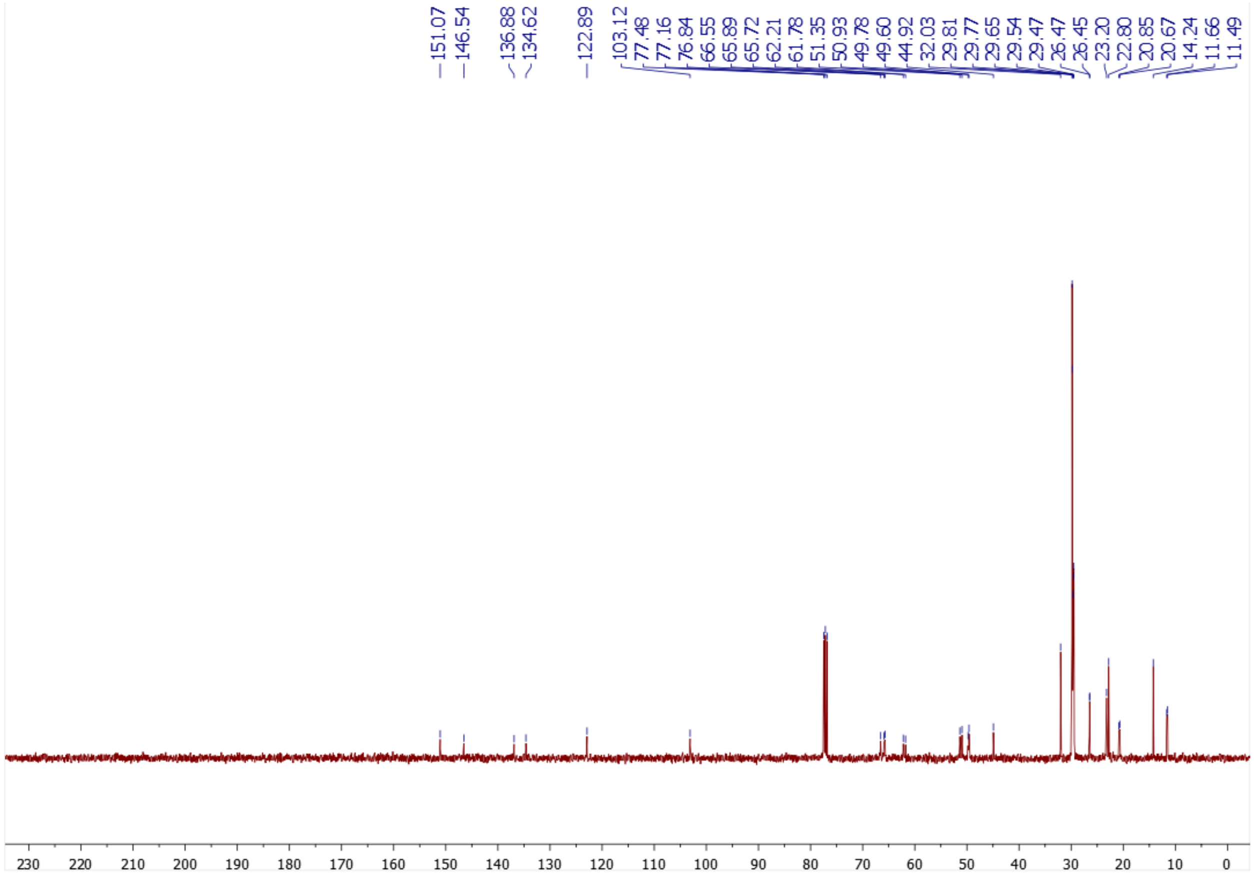

^{13}C{H} NMR spectrum of compound **5k16**

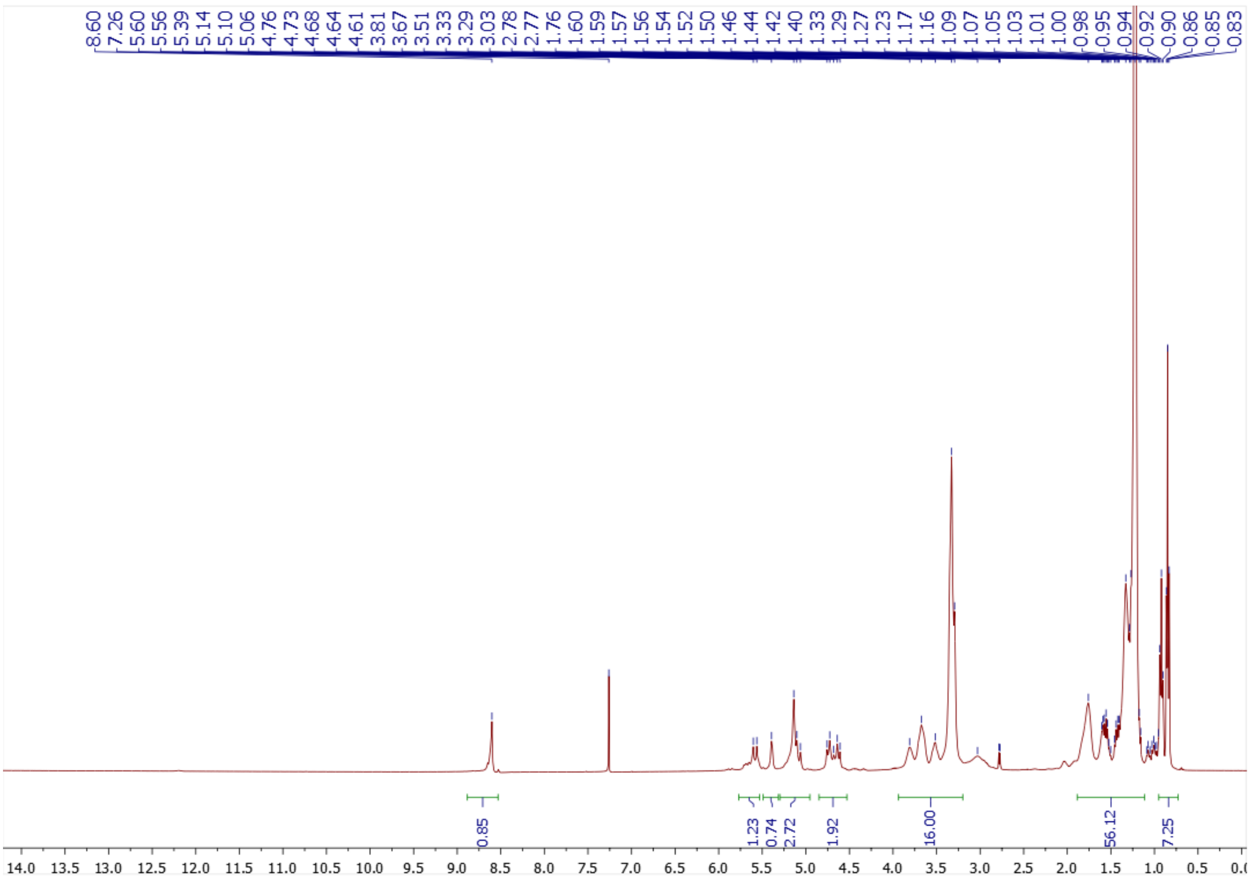

^{1}H NMR spectrum of compound **5k**$_{18}$

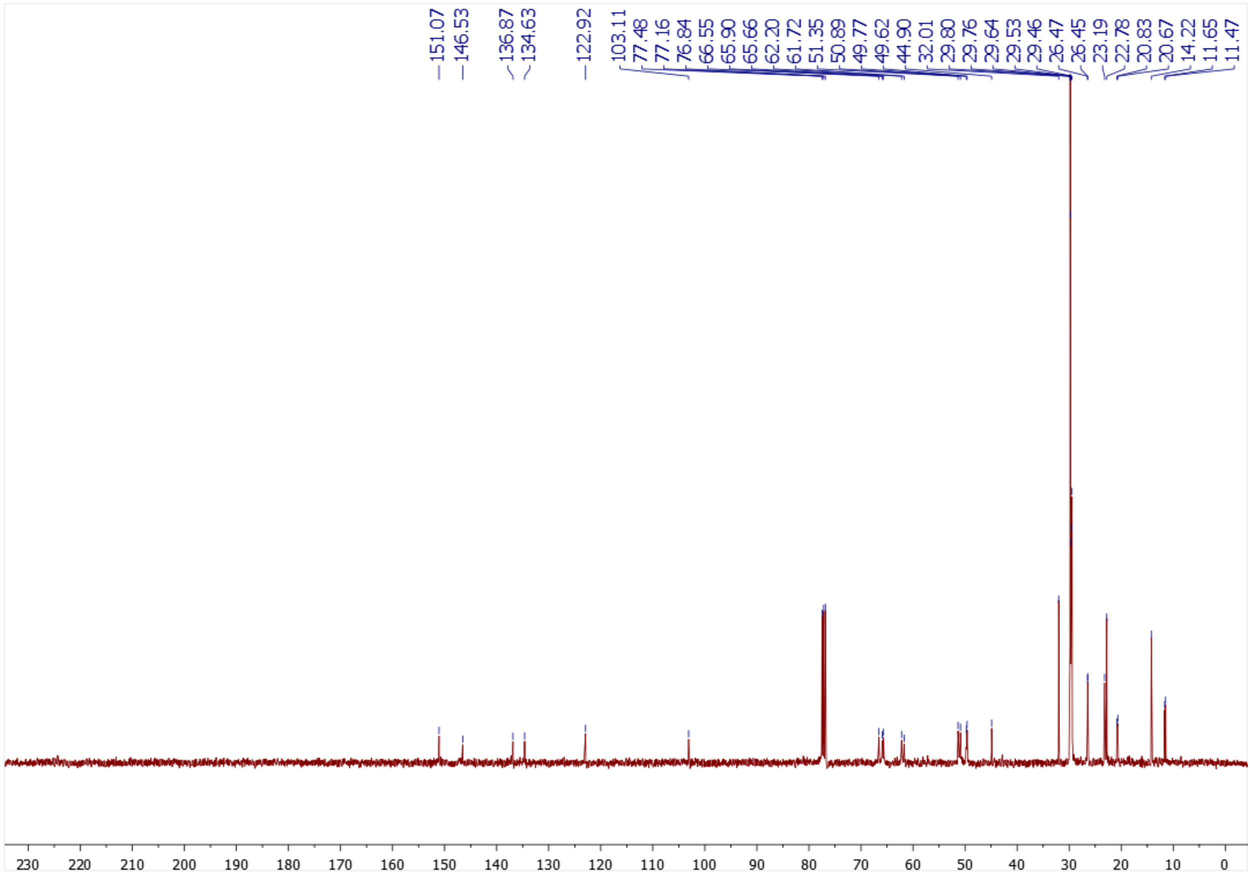

^{13}C{H} NMR spectrum of compound **5k**$_{18}$

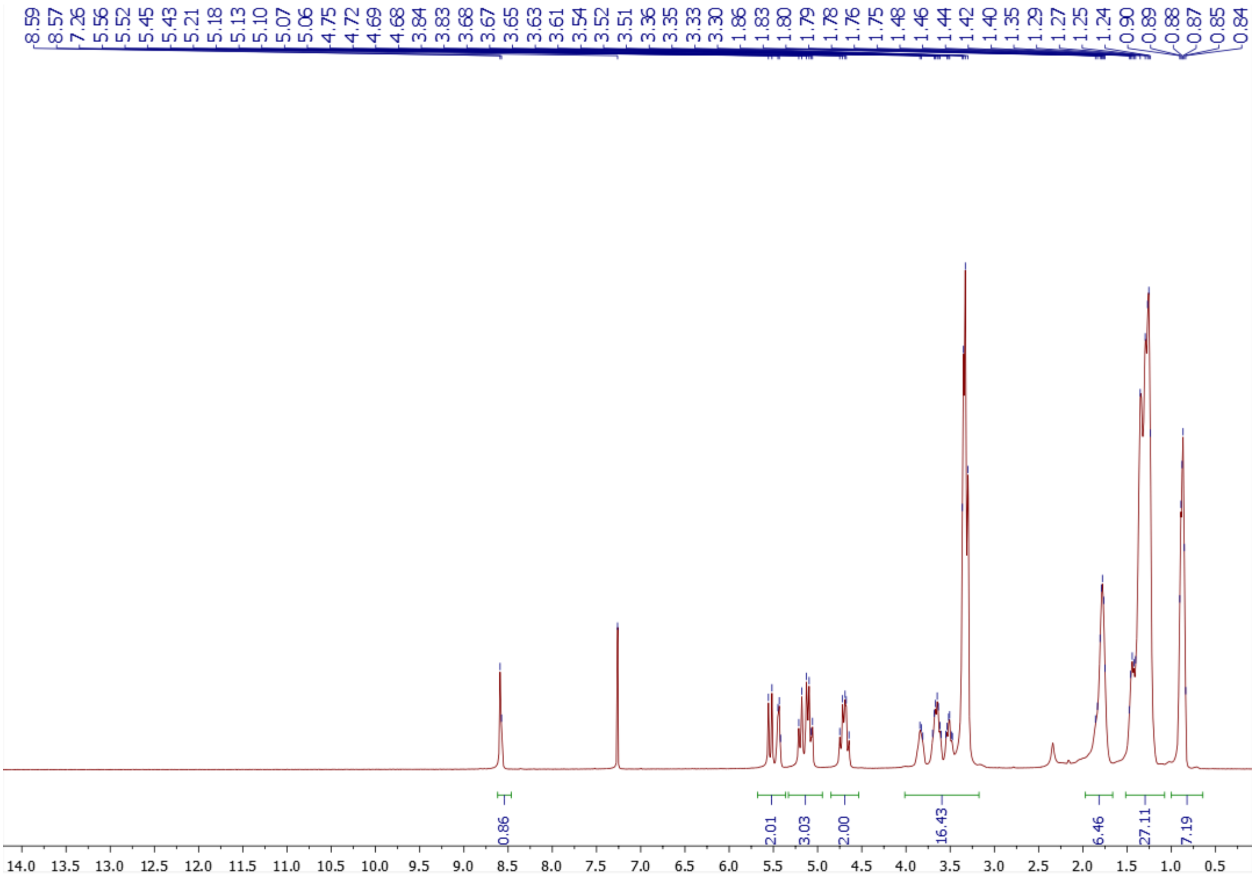

^{1}H NMR spectrum of compound **5l**$_8$

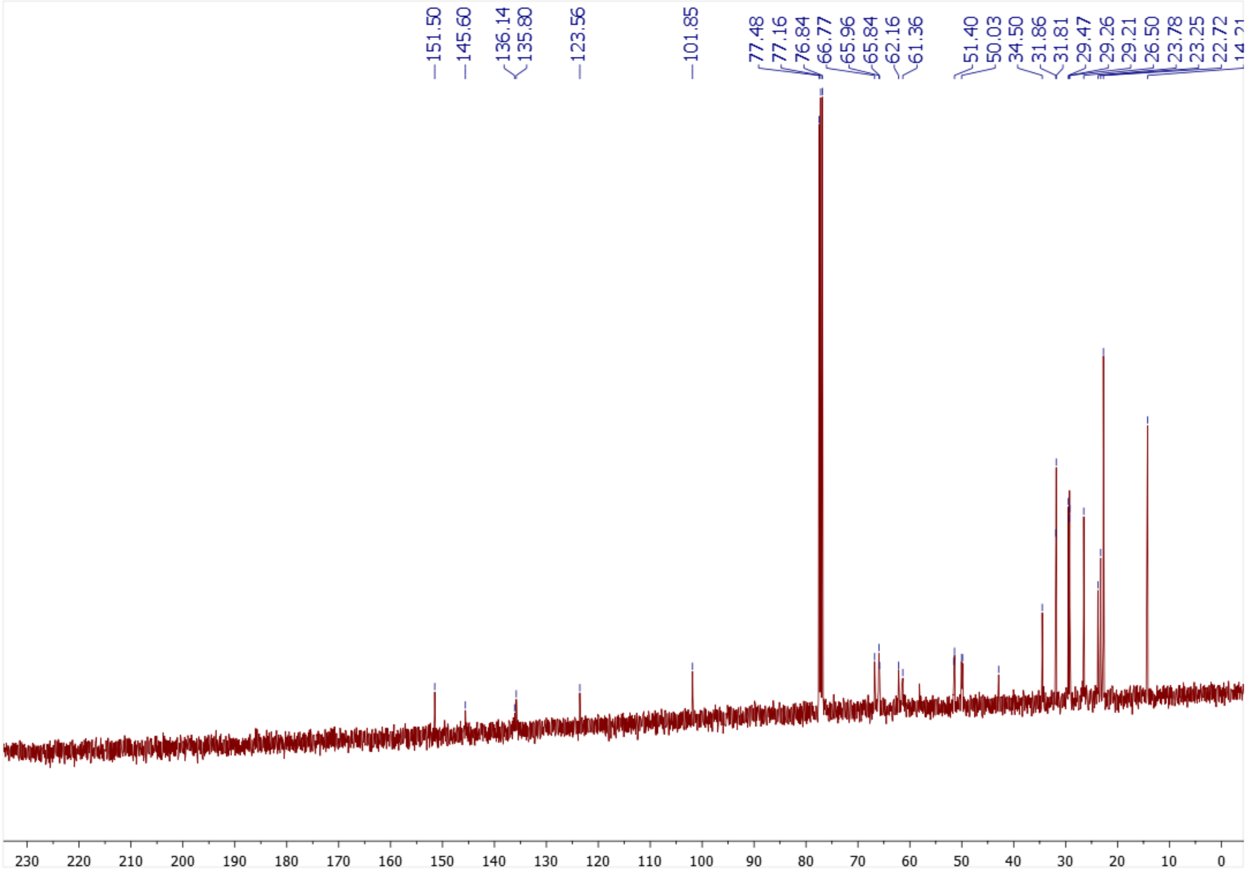

^{13}C{H} NMR spectrum of compound **5l**$_8$

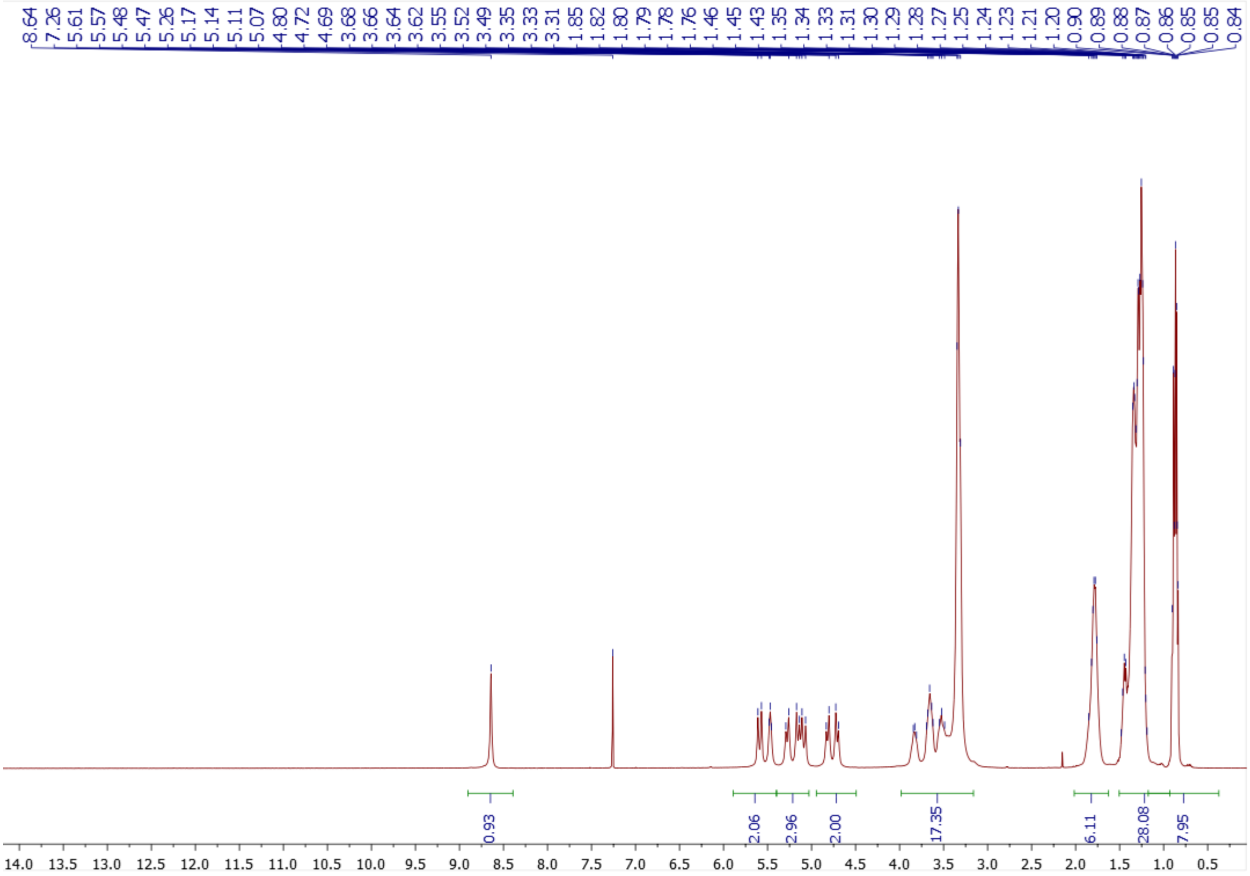

^{1}H NMR spectrum of compound **5l**$_{10}$

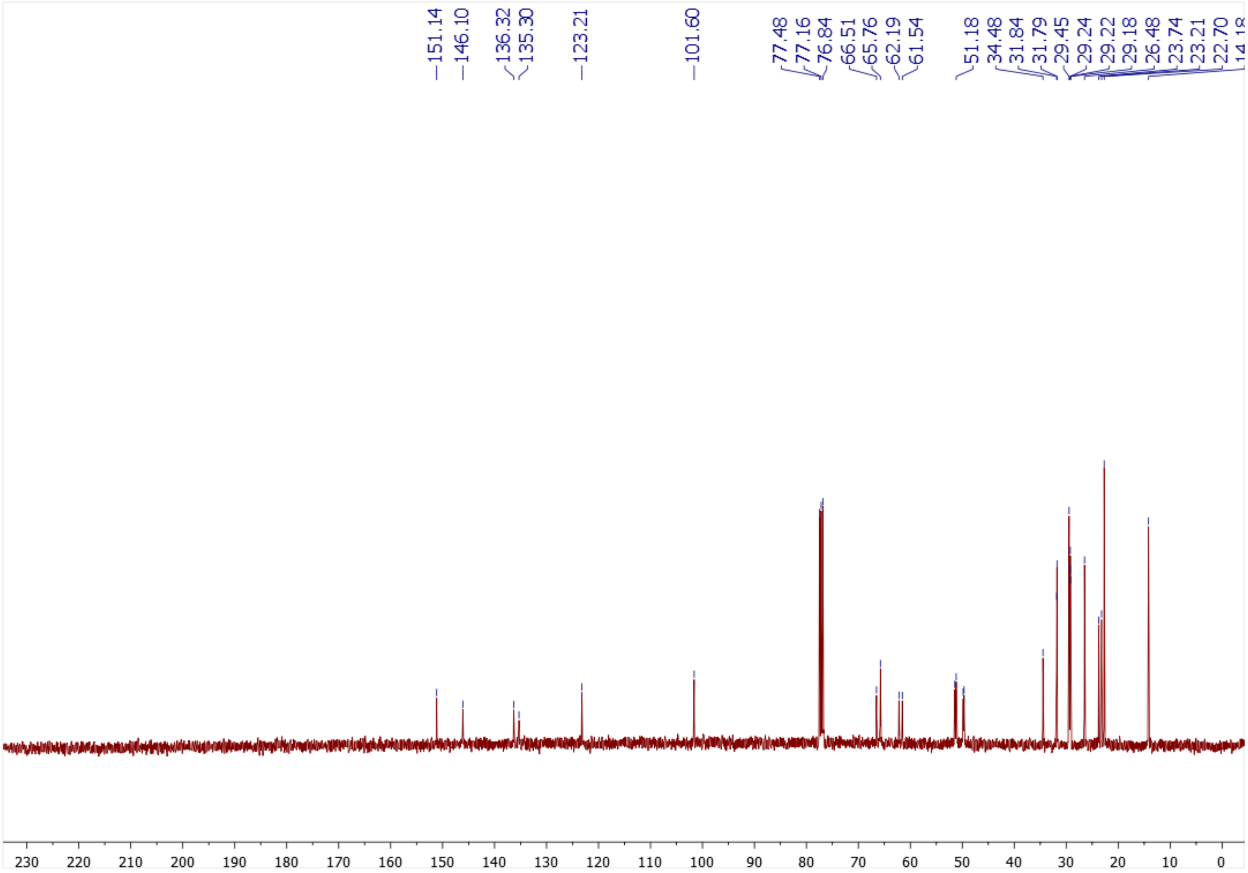

^{13}C{H} NMR spectrum of compound **5l**$_{10}$

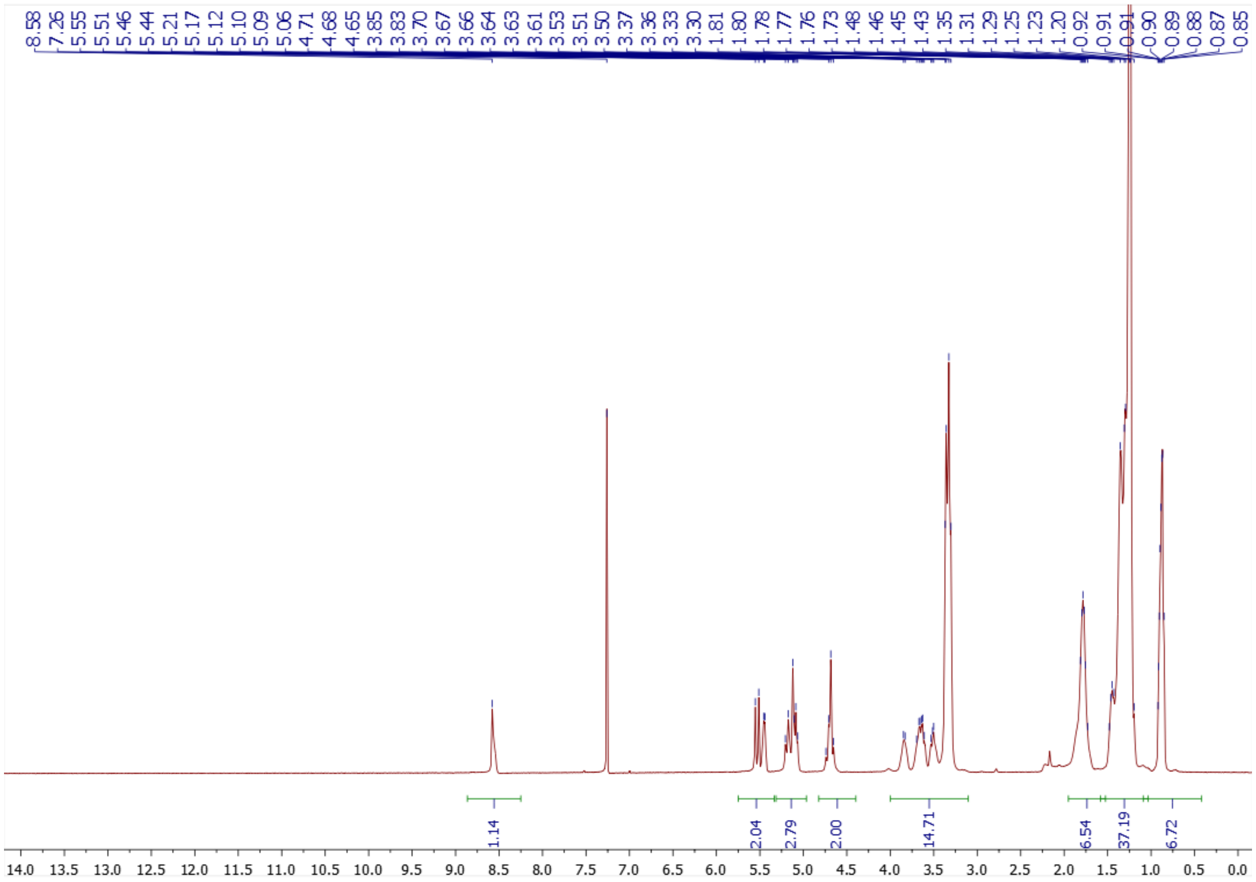

^{1}H NMR spectrum of compound **5l**$_{12}$

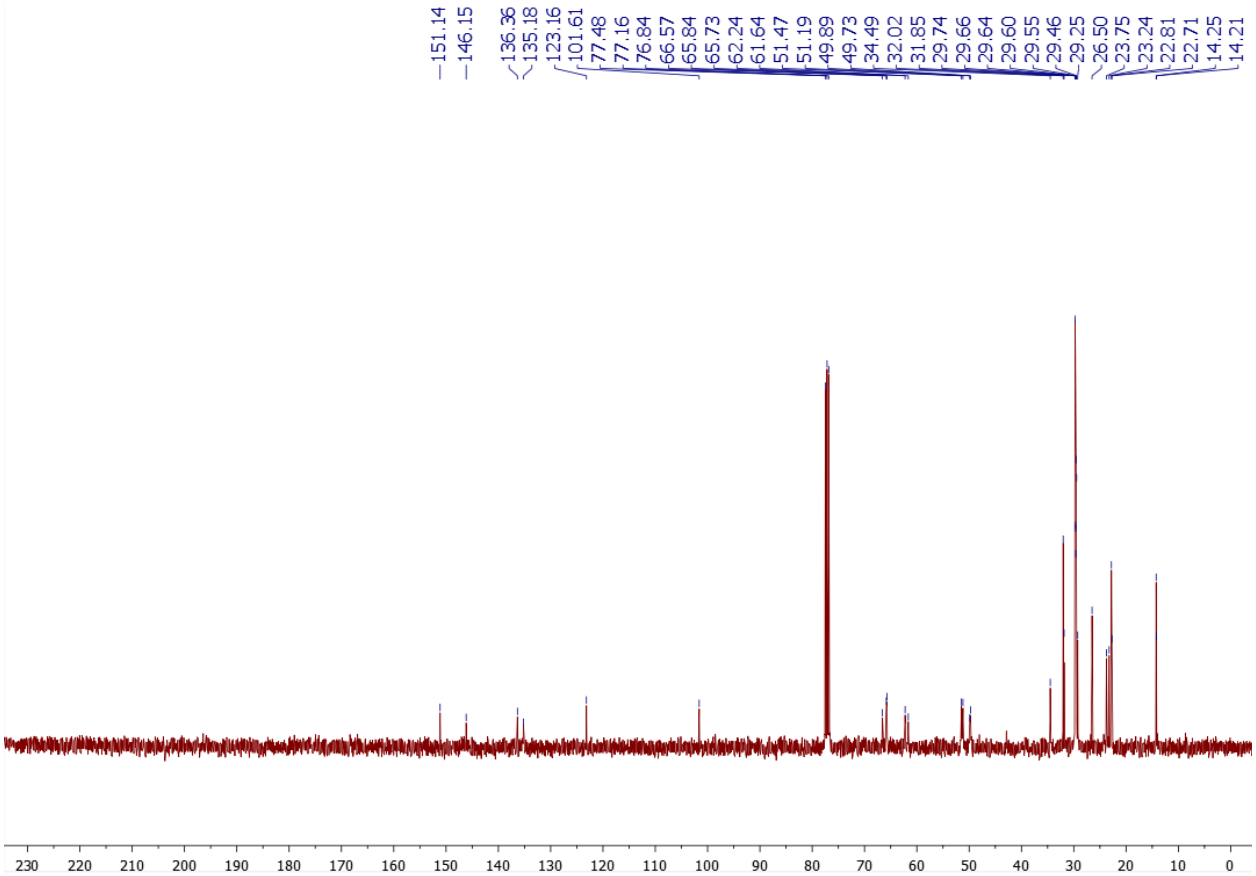

^{13}C{H} NMR spectrum of compound **5l**$_{12}$

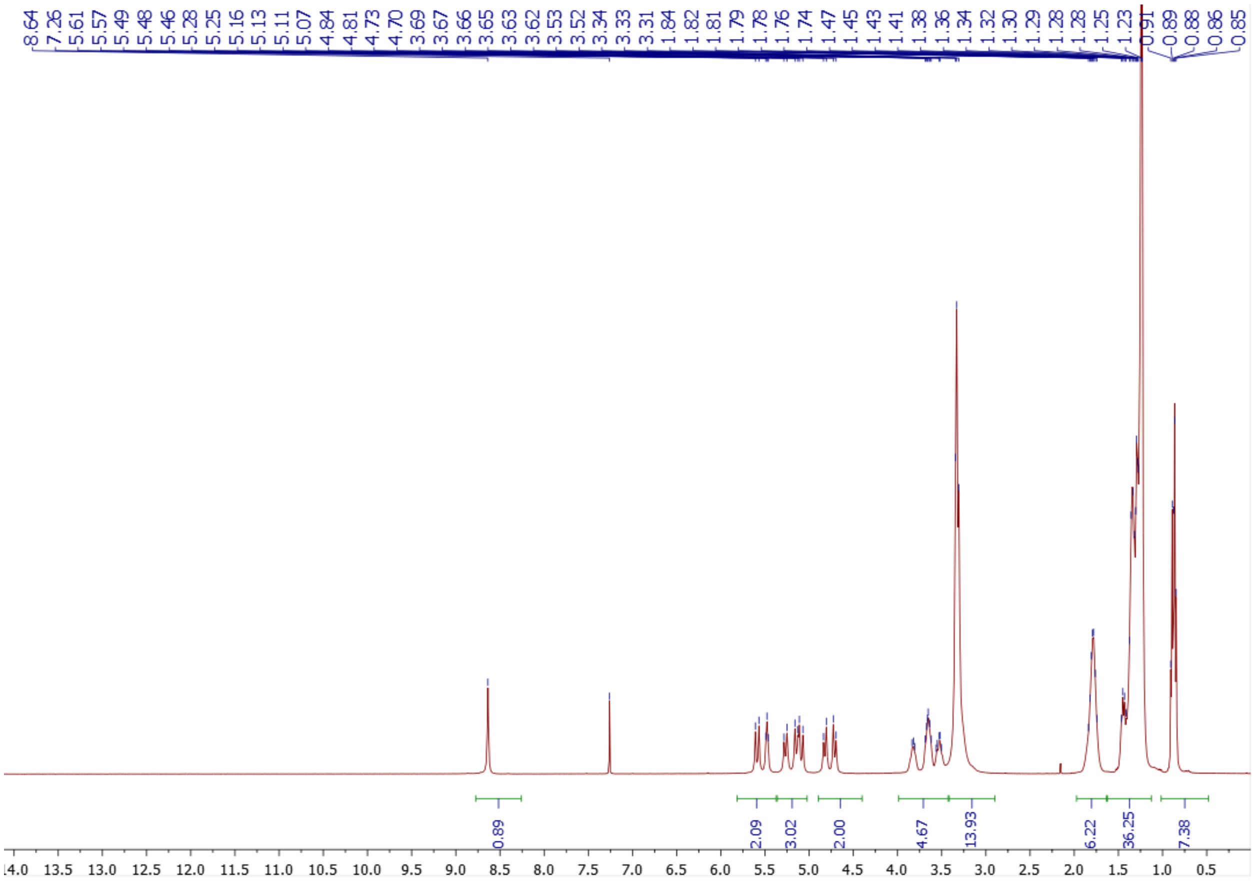

^{1}H NMR spectrum of compound **5l**$_{14}$

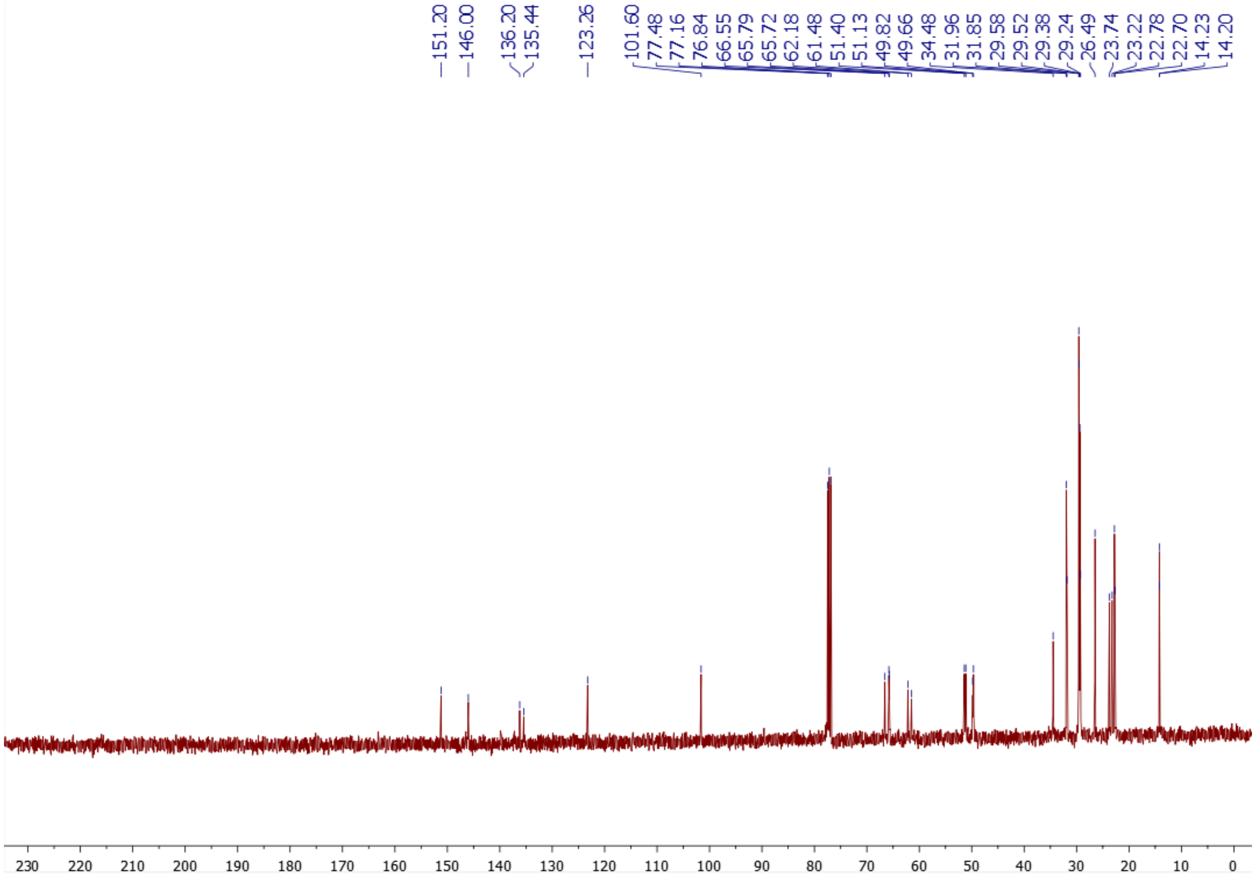

^{13}C{H} NMR spectrum of compound **5l**$_{14}$

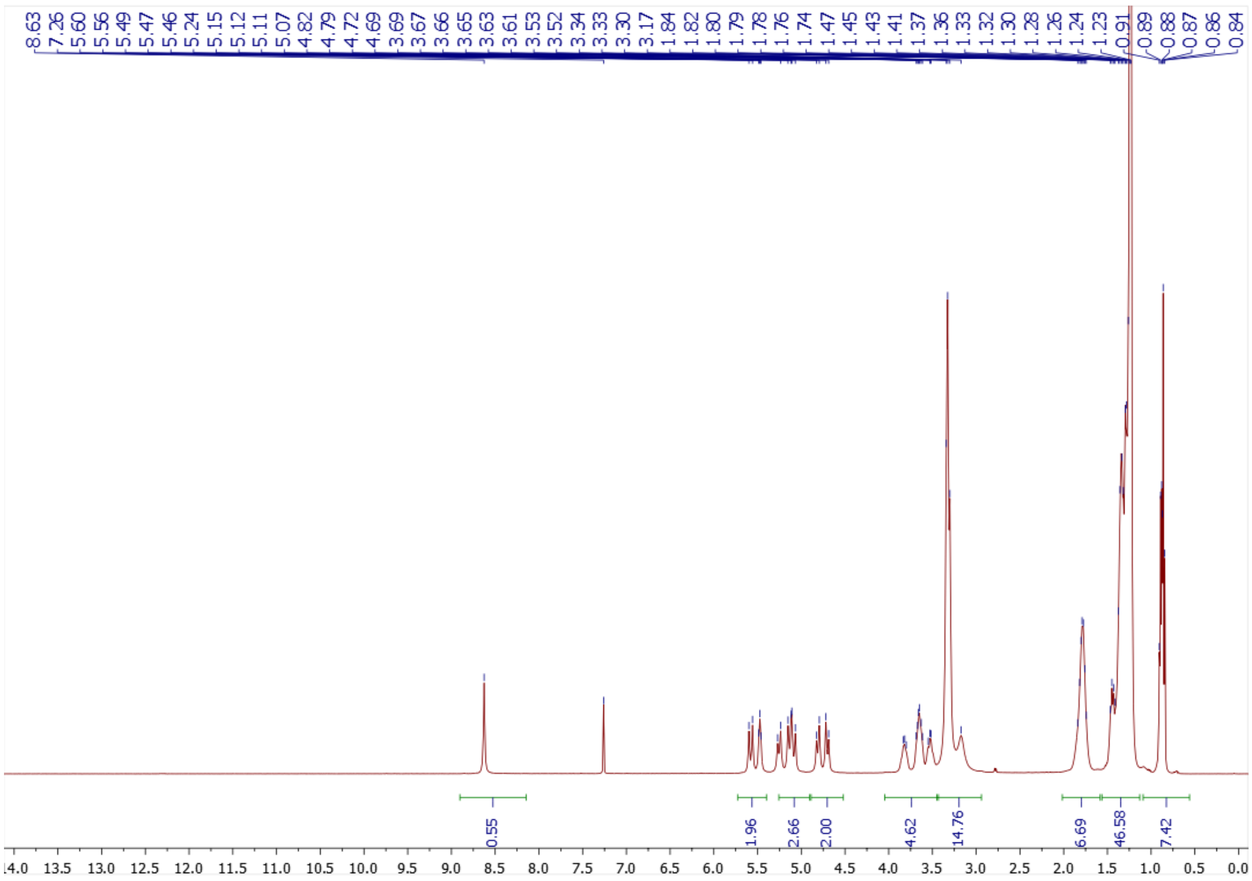

^{1}H NMR spectrum of compound **5l16**

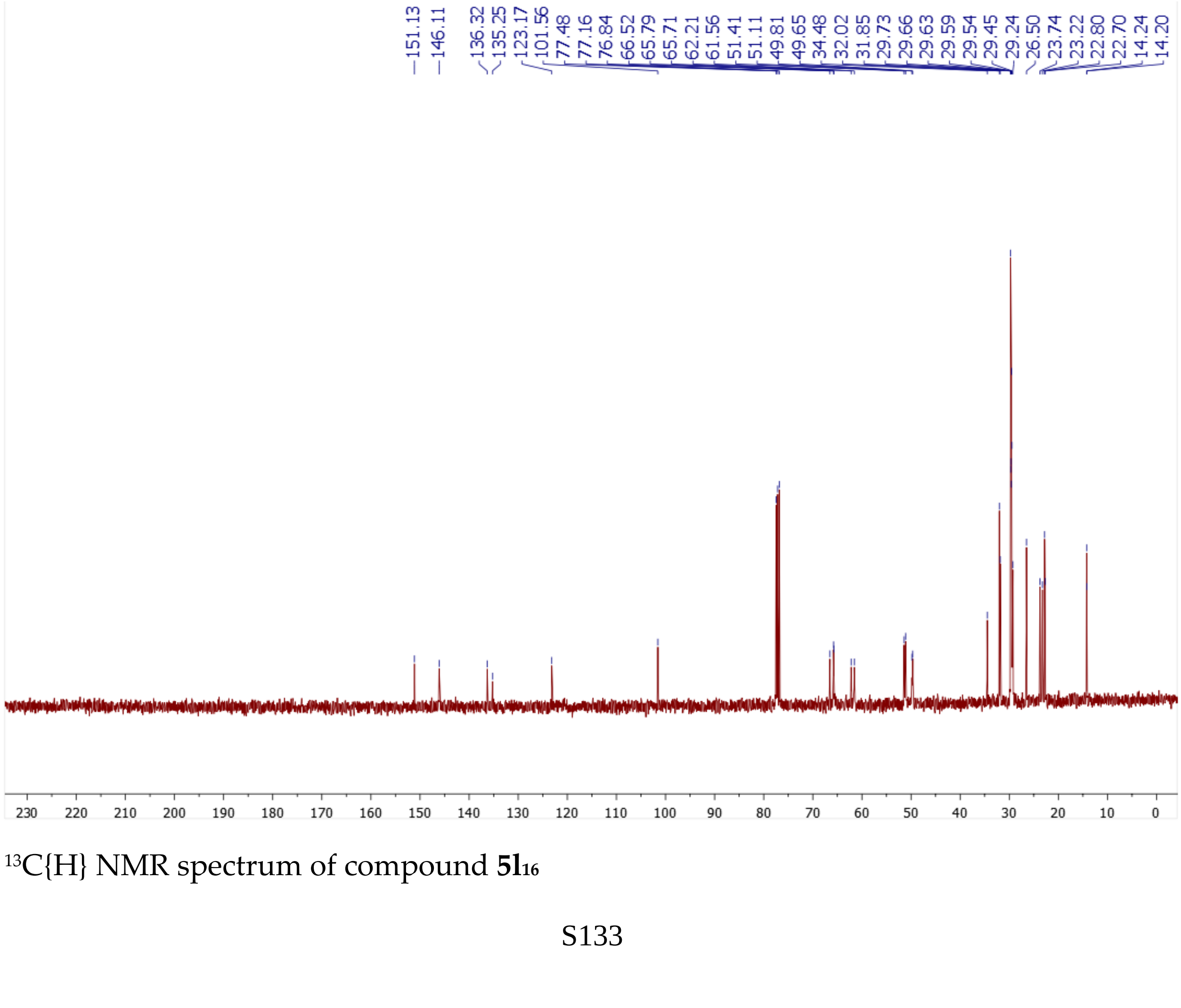

^{13}C{H} NMR spectrum of compound **5l16**

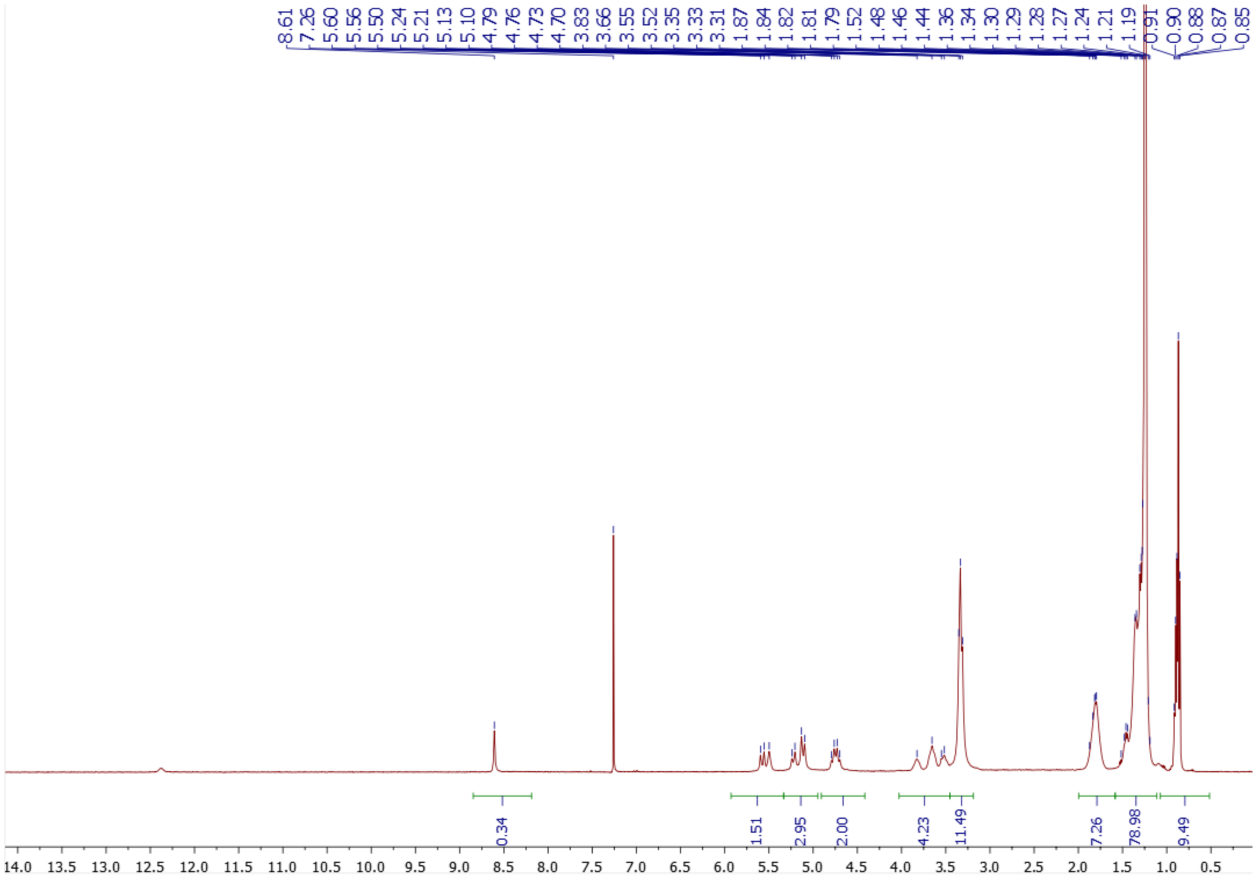

^{1}H NMR spectrum of compound **5l$_{18}$**

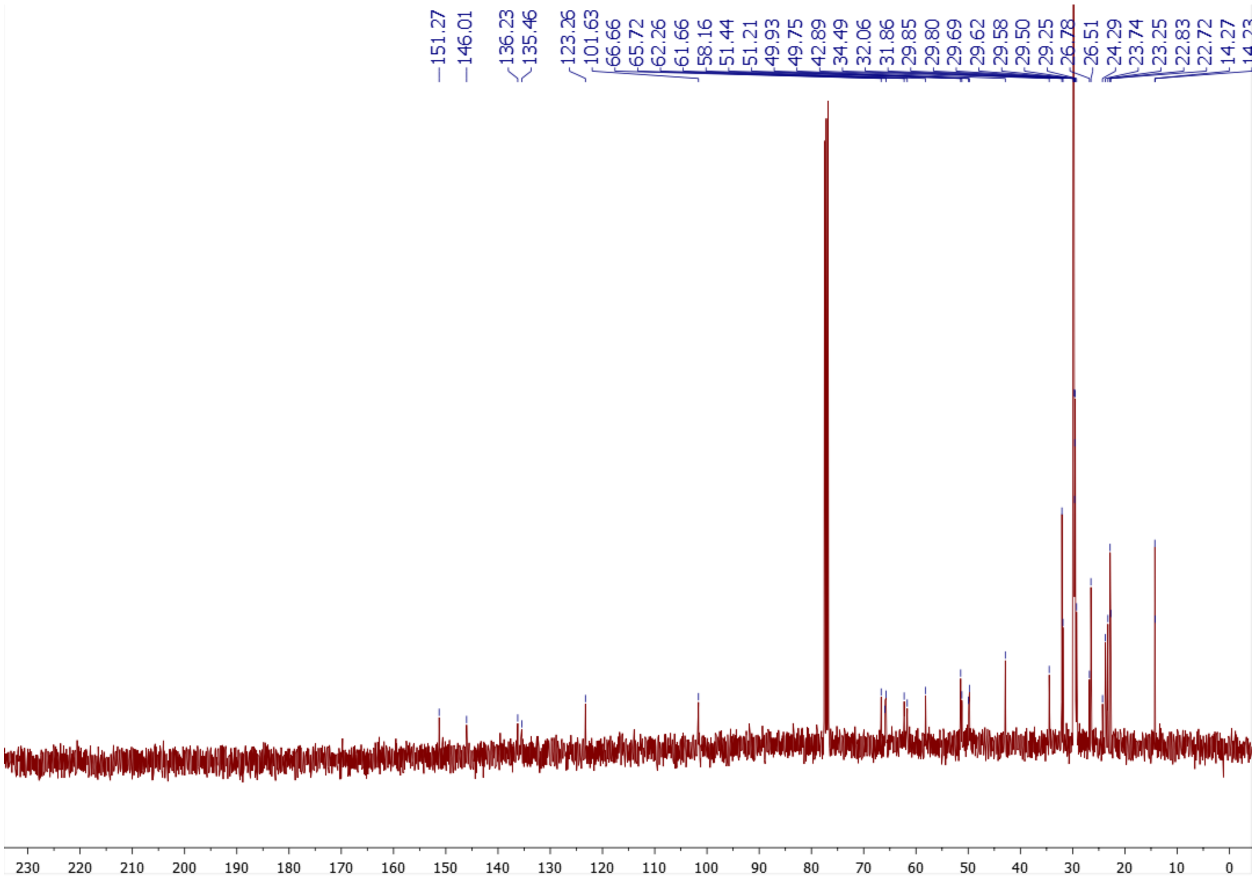

^{13}C{H} NMR spectrum of compound **5l$_{18}$**

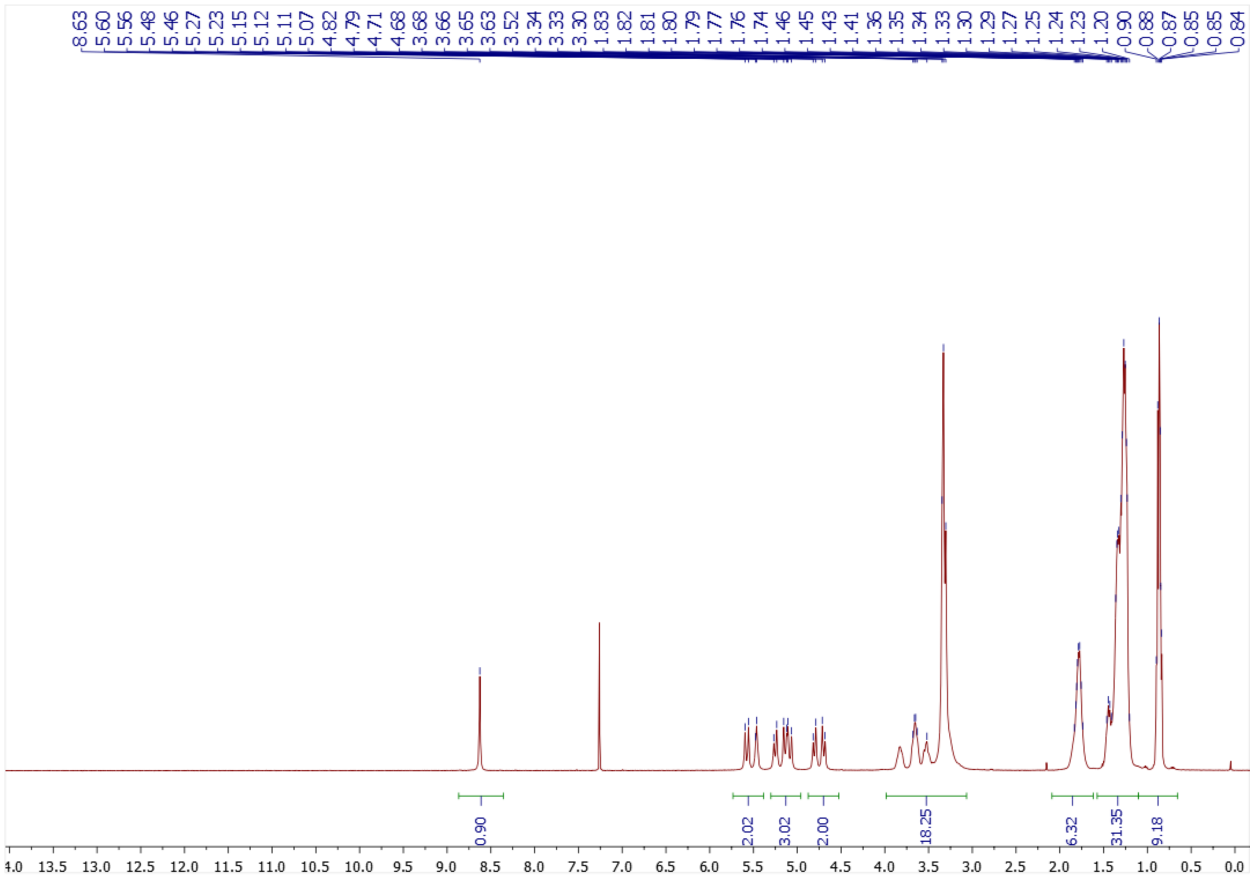

^{1}H NMR spectrum of compound **5m**8

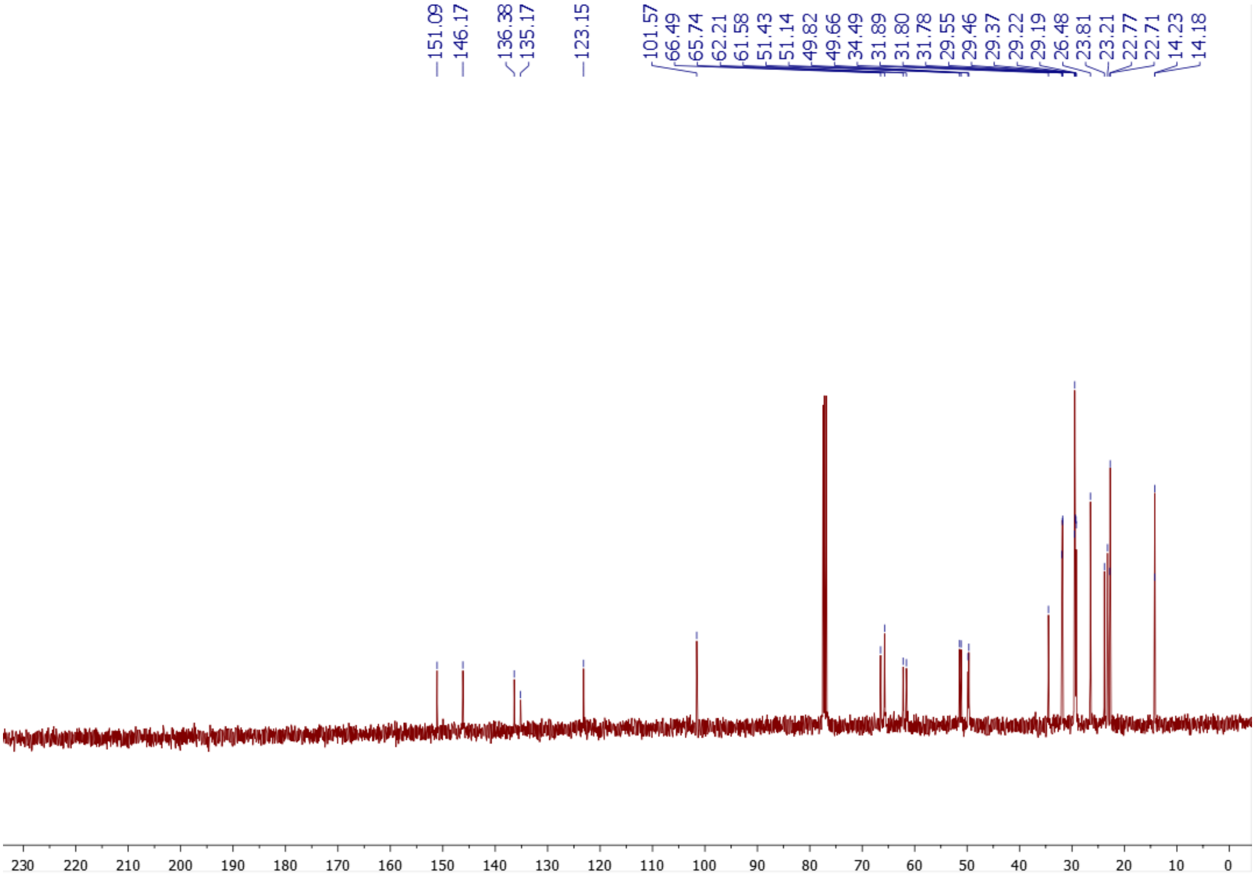

^{13}C{H} NMR spectrum of compound **5m**8

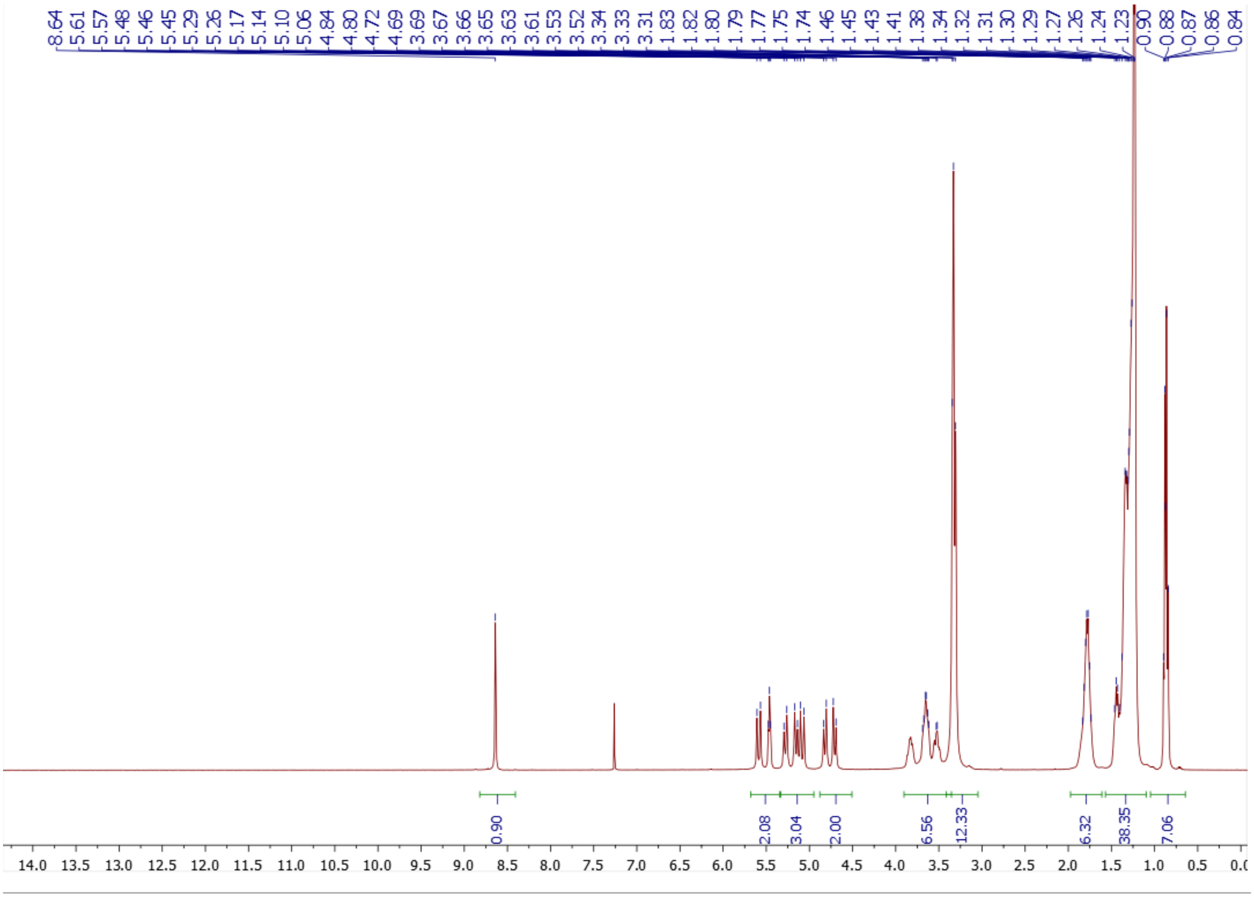

^{1}H NMR spectrum of compound **5m$_{10}$**

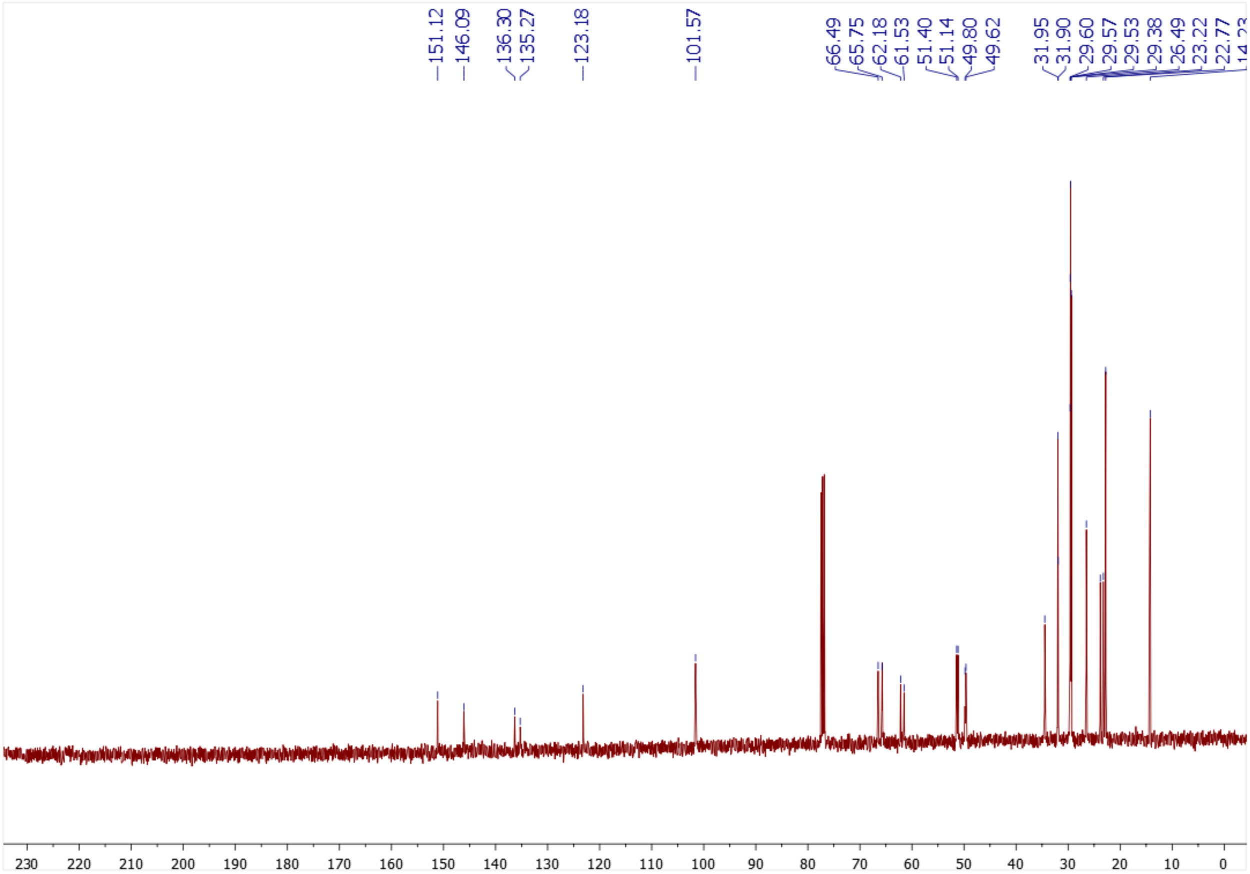

^{13}C{H} NMR spectrum of compound **5m$_{10}$**

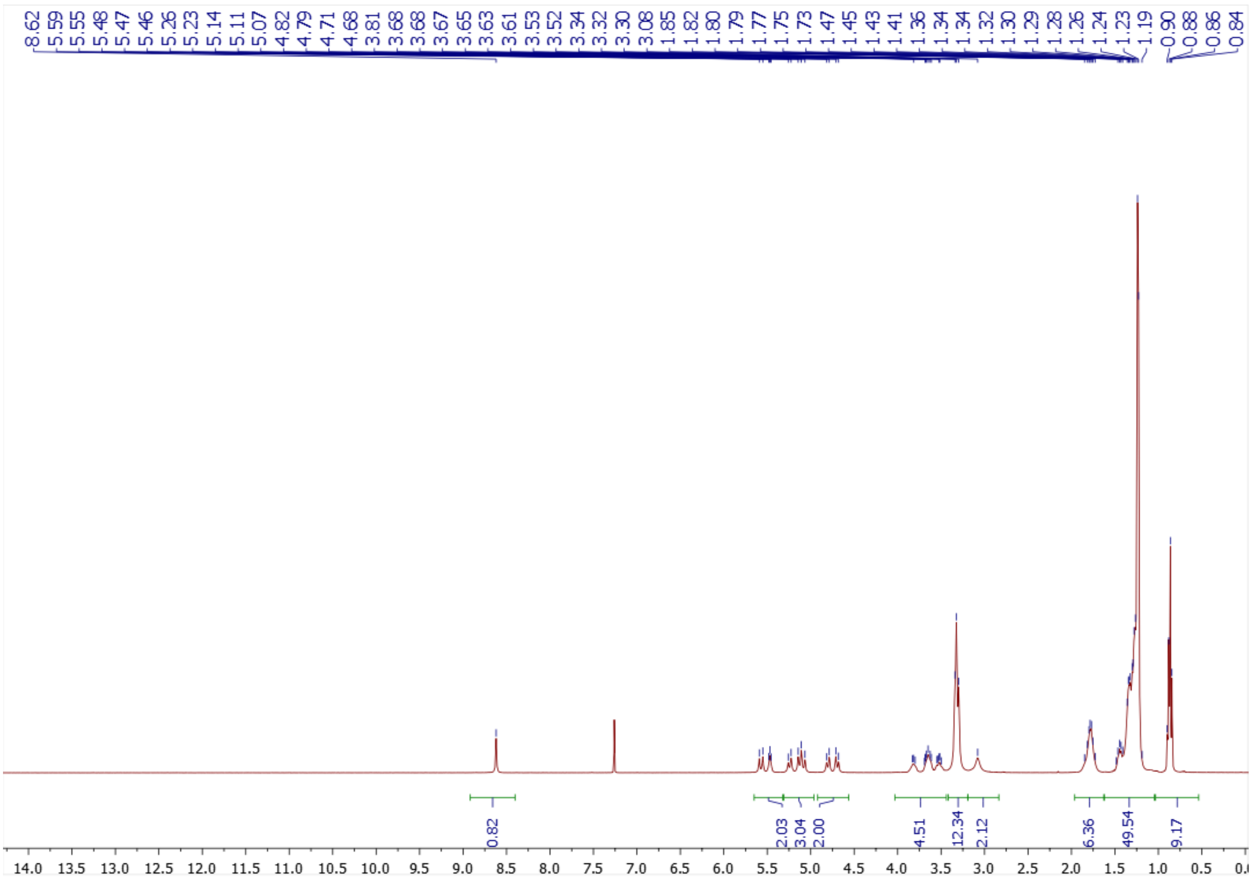

^{1}H NMR spectrum of compound **5m12**

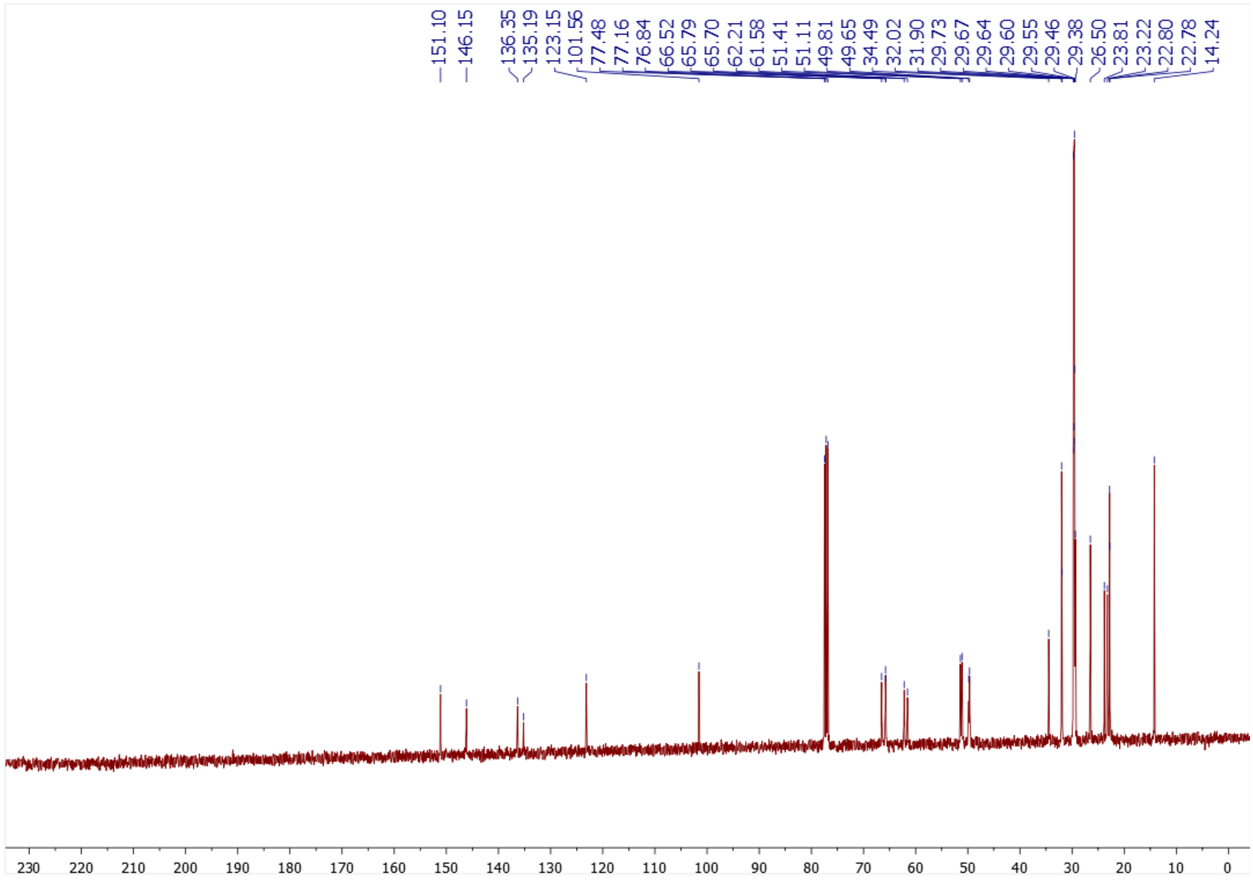

^{13}C{H} NMR spectrum of compound **5m12**

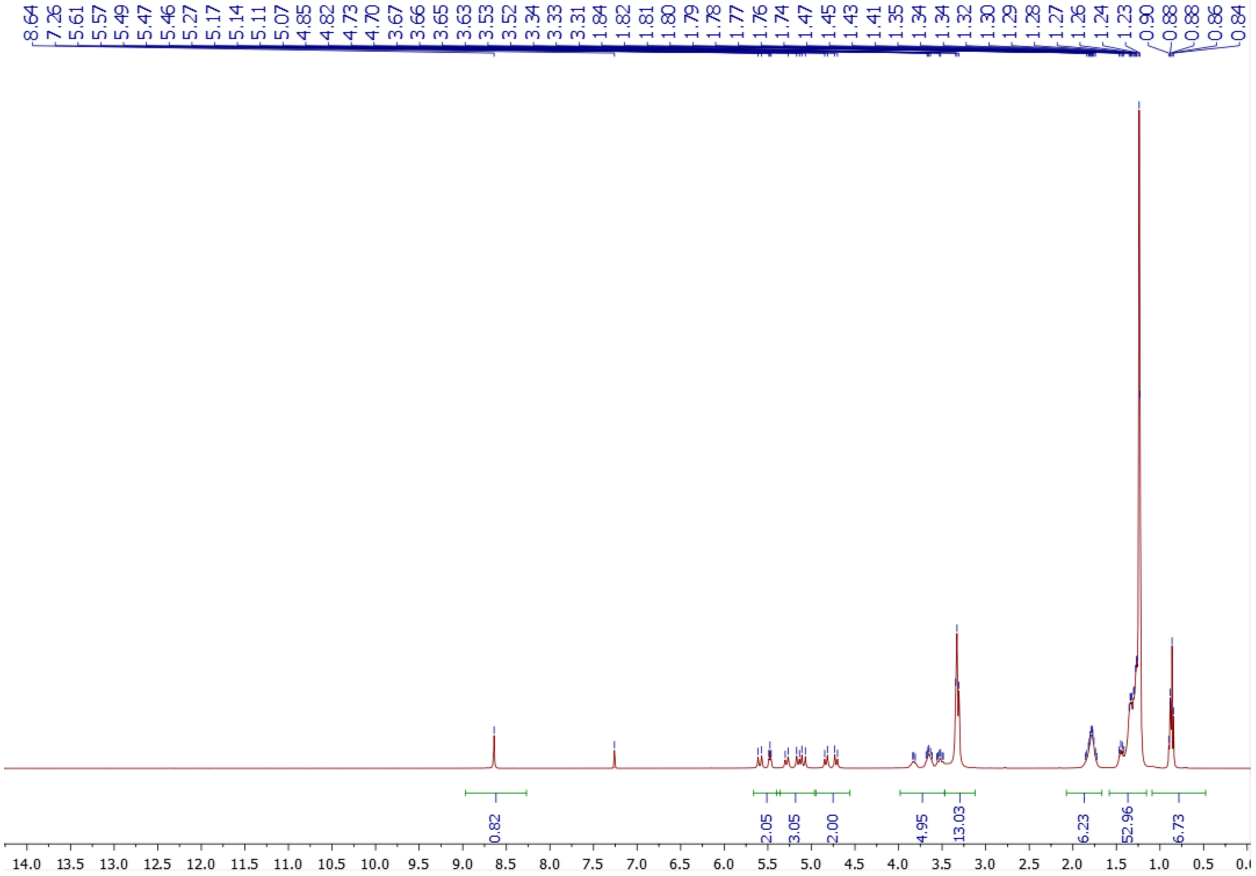

^{1}H NMR spectrum of compound **5m**14

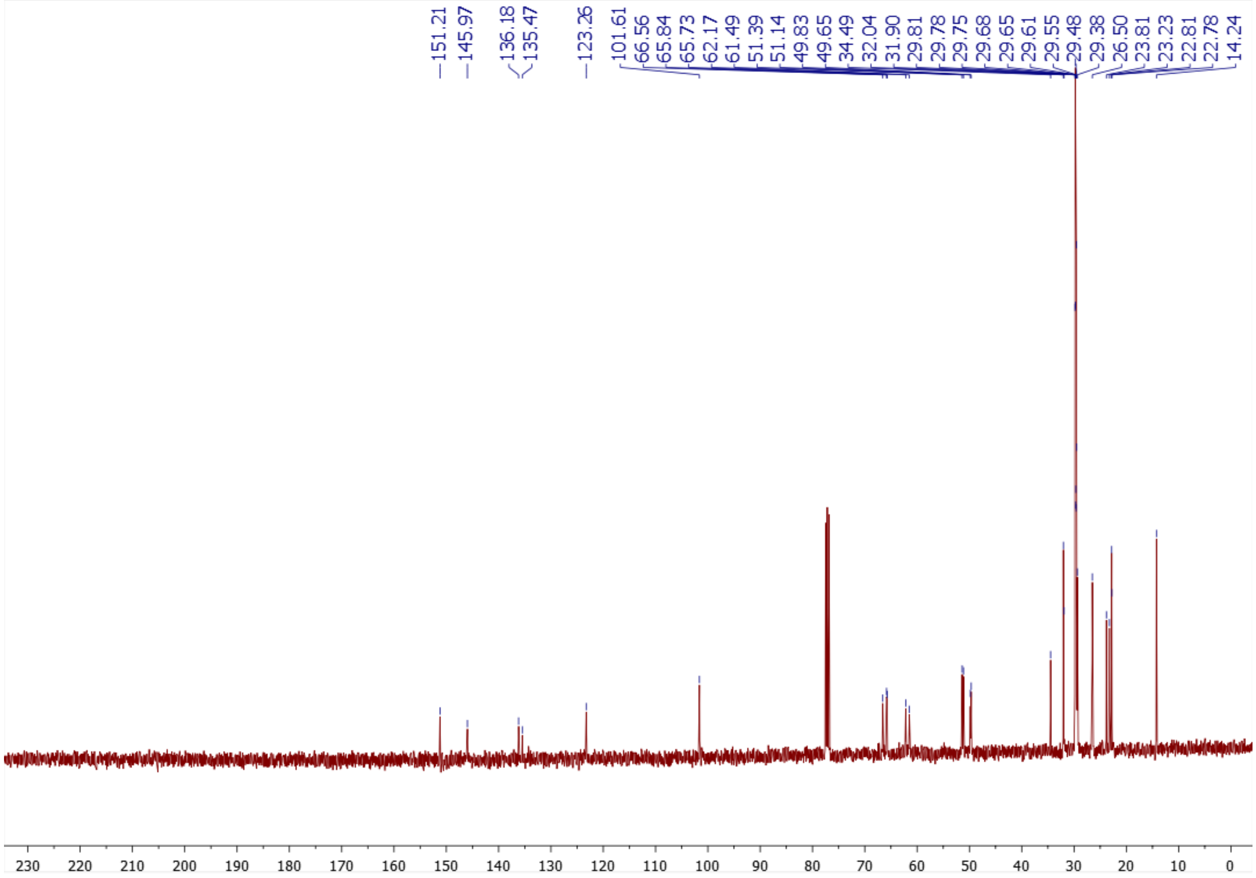

^{13}C{H} NMR spectrum of compound **5m**14

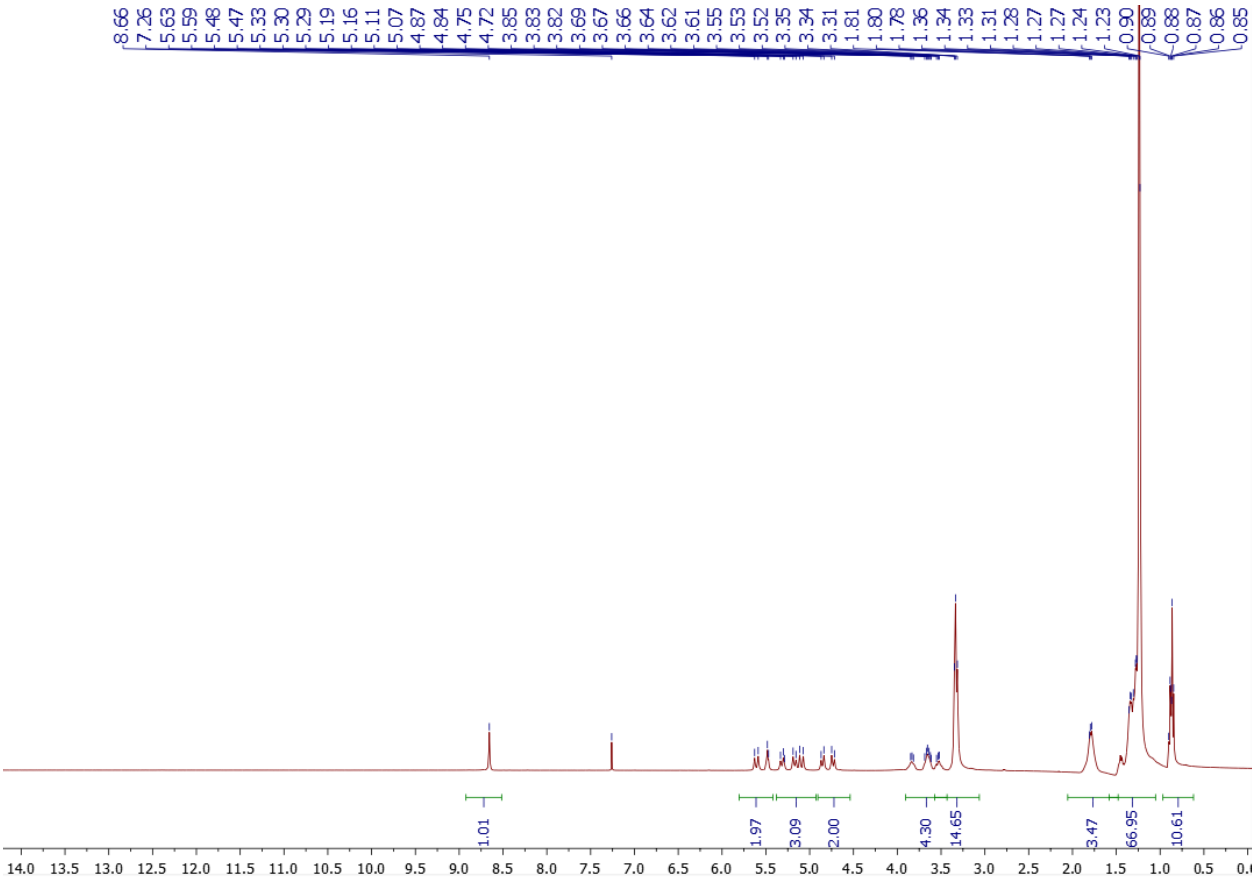

^{1}H NMR spectrum of compound **5m16**

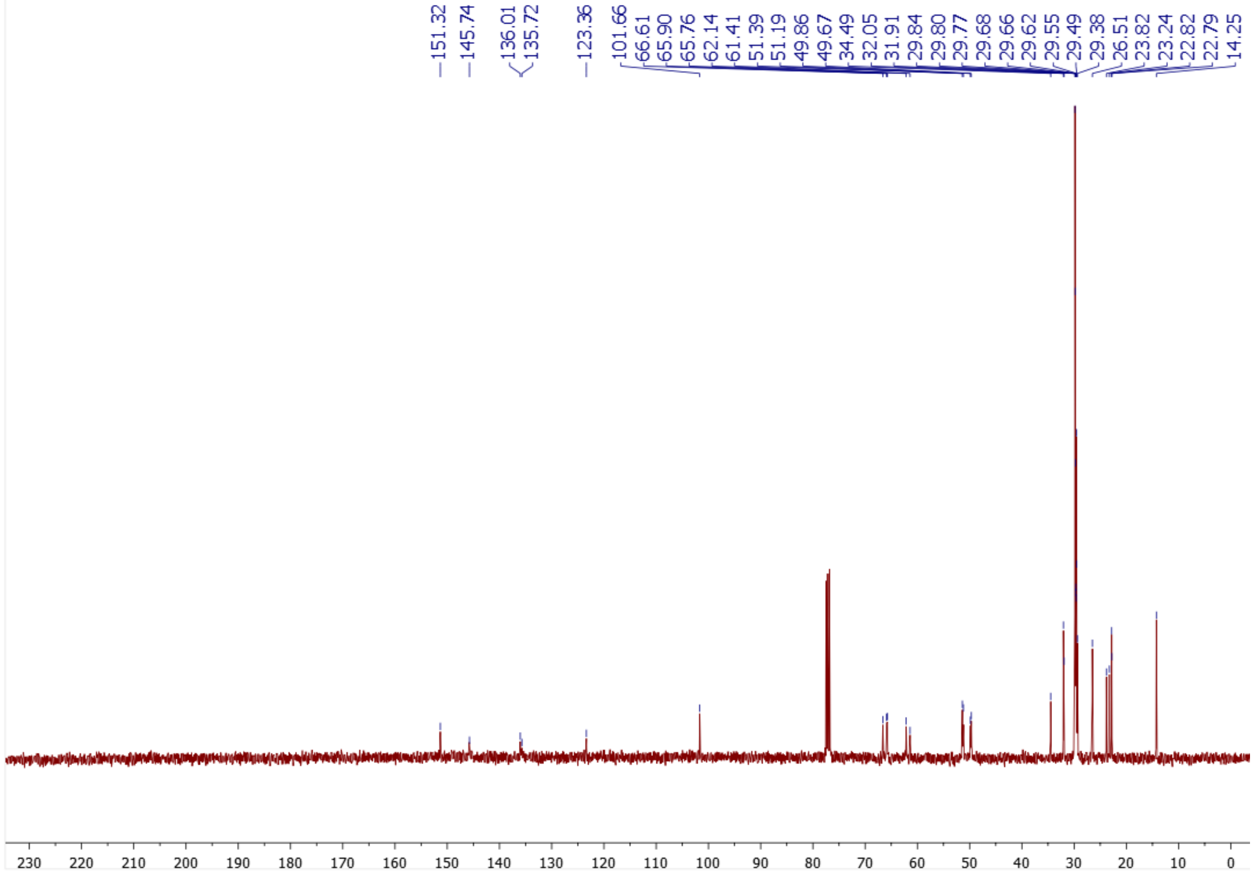

^{13}C{H} NMR spectrum of compound **5m16**

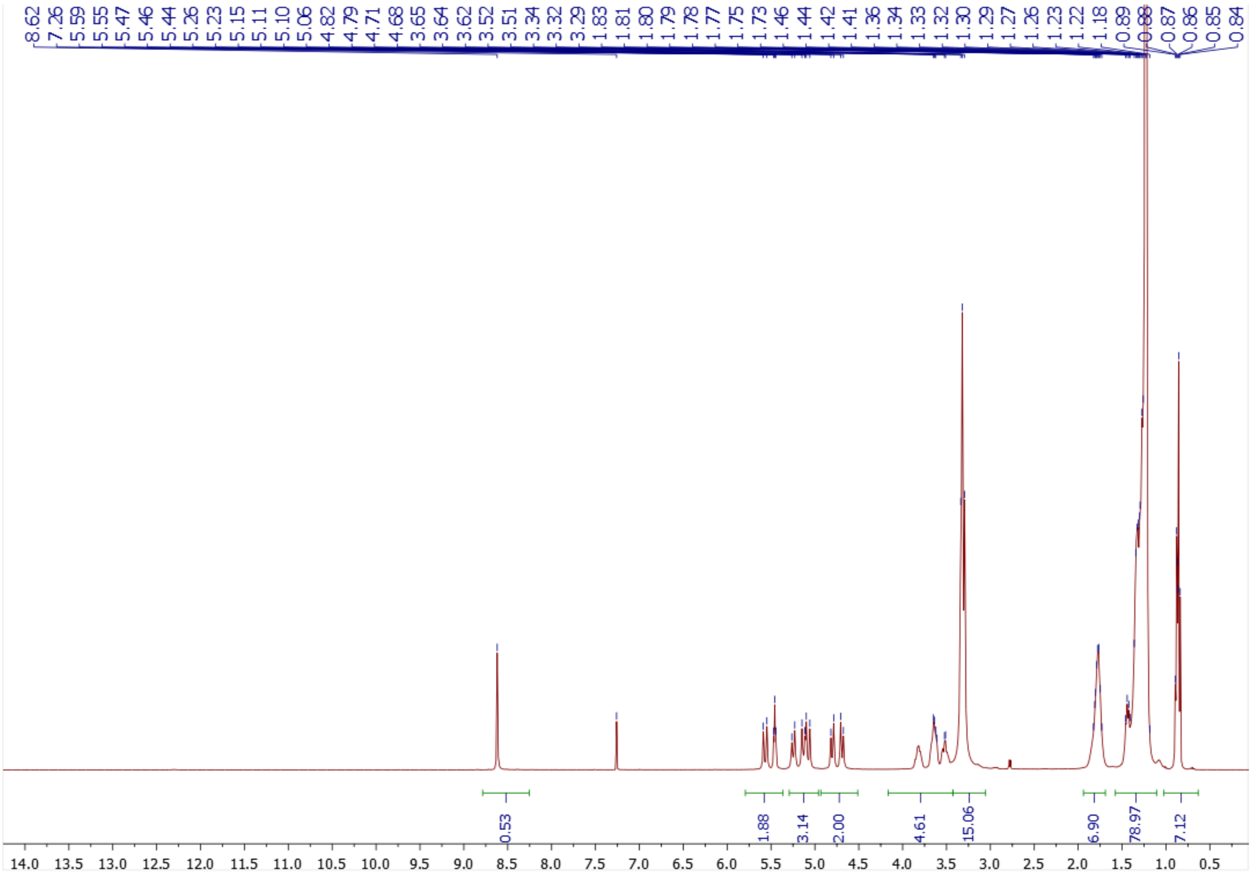

^{1}H NMR spectrum of compound **5m**$_{18}$

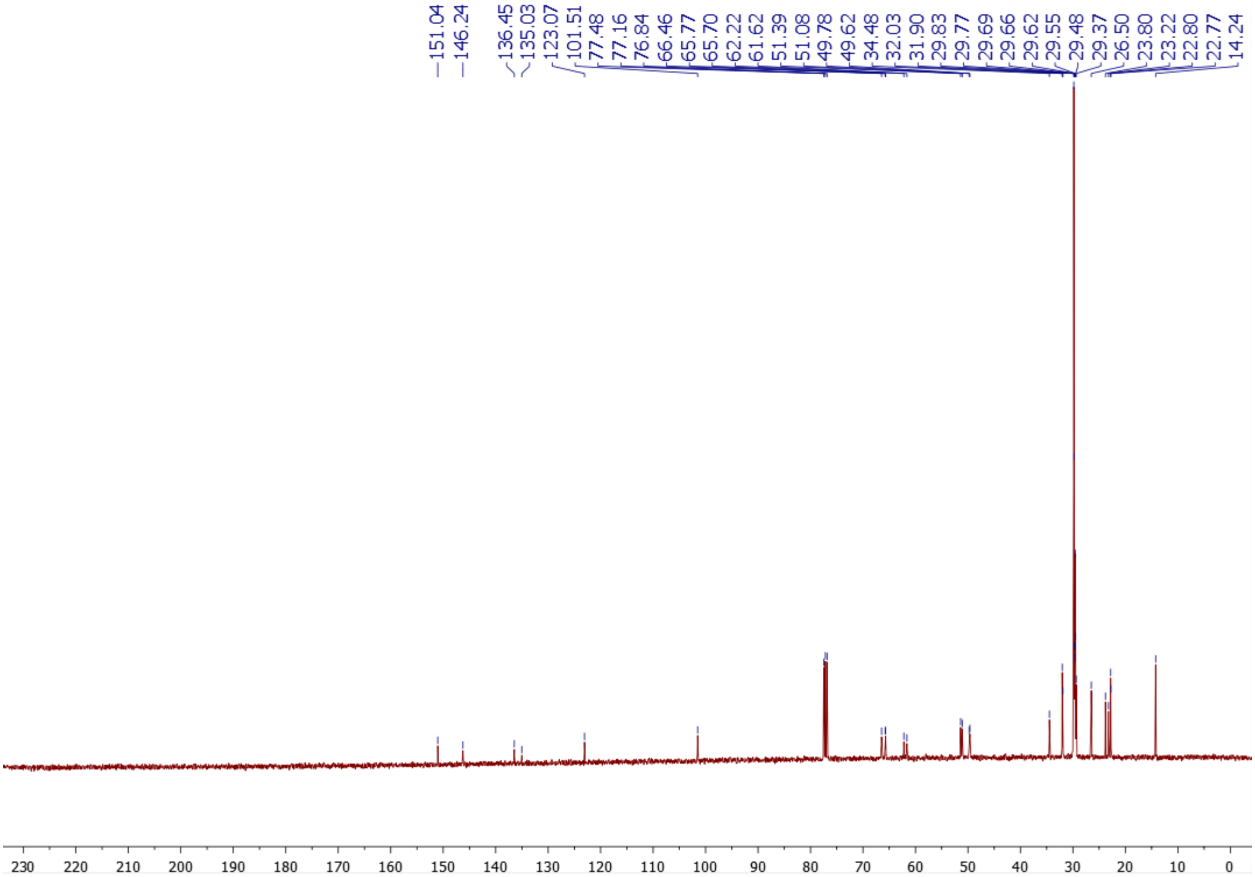

^{13}C{H} NMR spectrum of compound **5m**$_{18}$

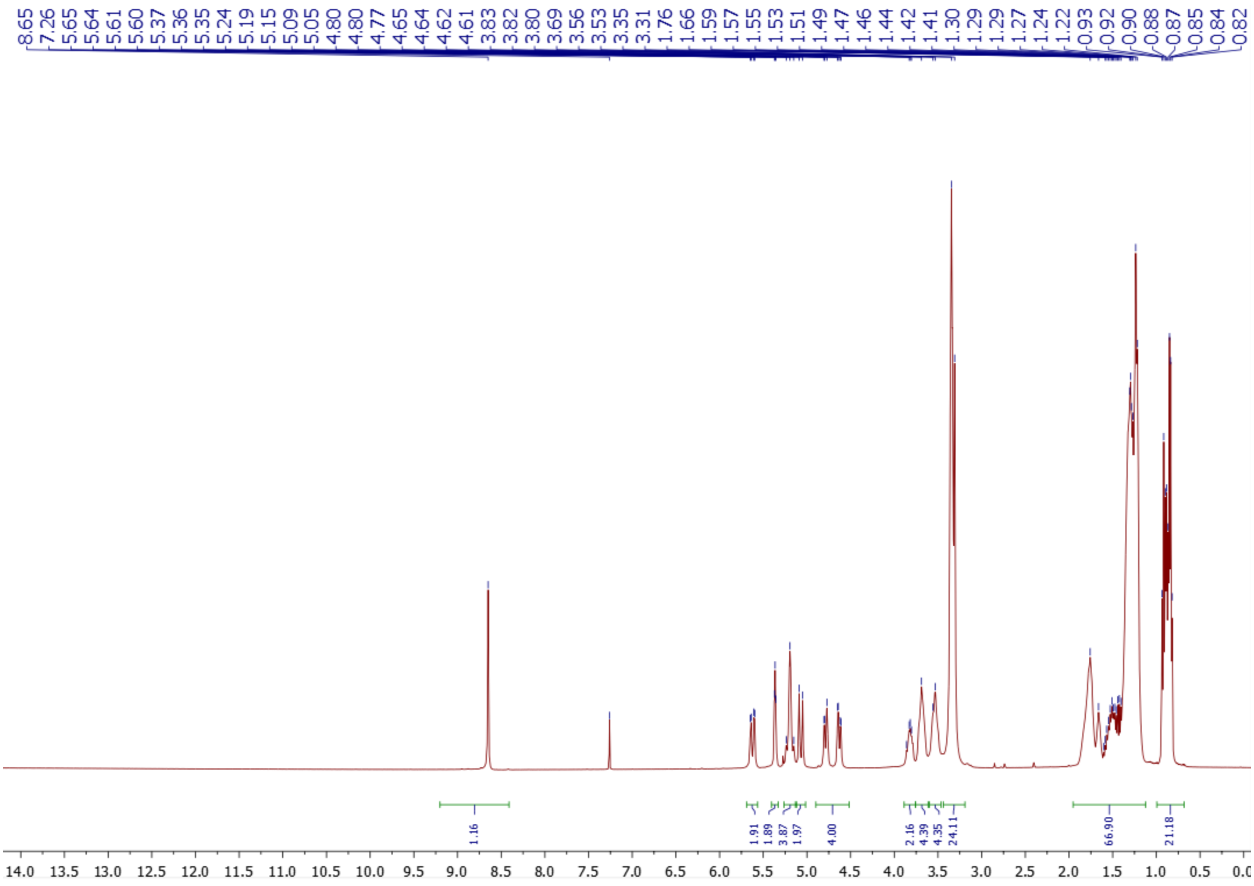

^{1}H NMR spectrum of compound **5n**8

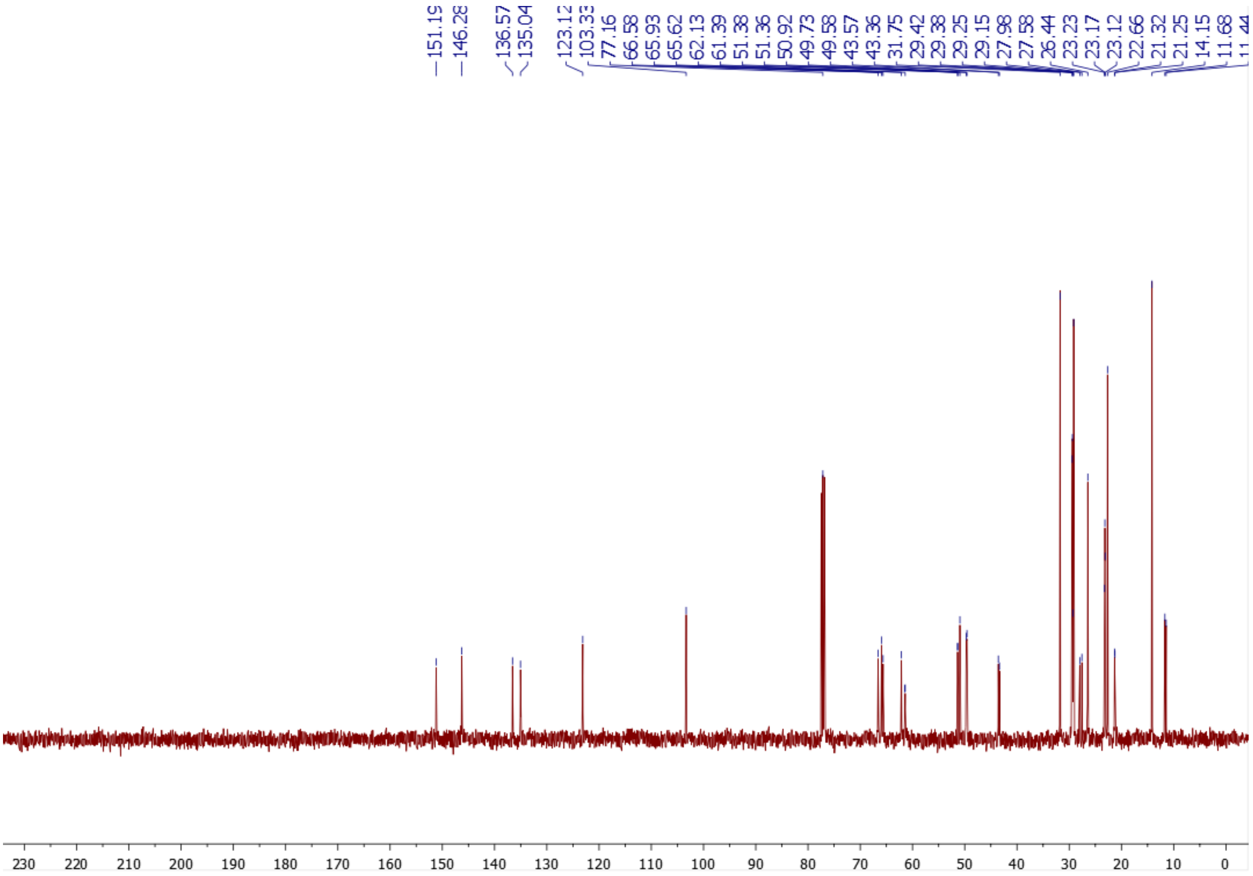

^{13}C{H} NMR spectrum of compound **5n**8

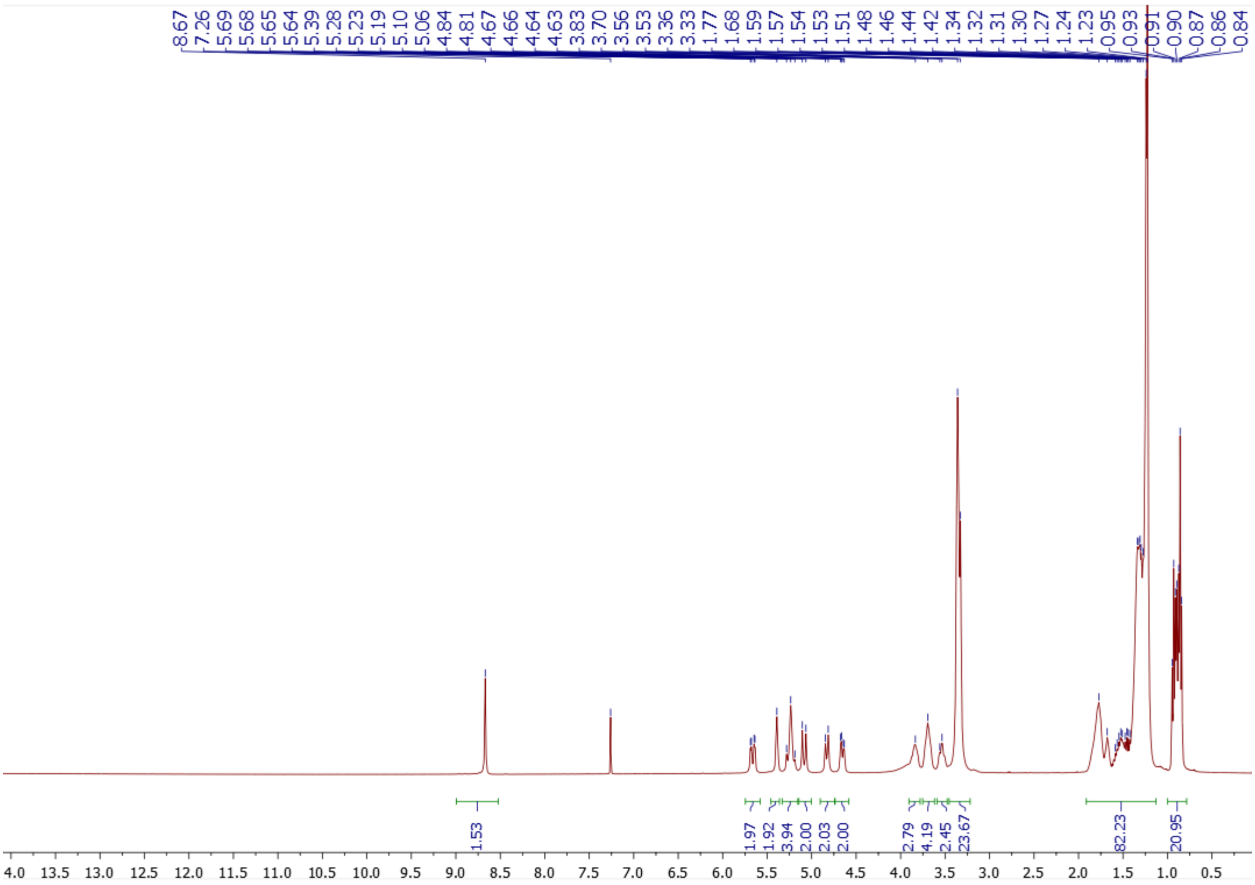

^{1}H NMR spectrum of compound **5n$_{10}$**

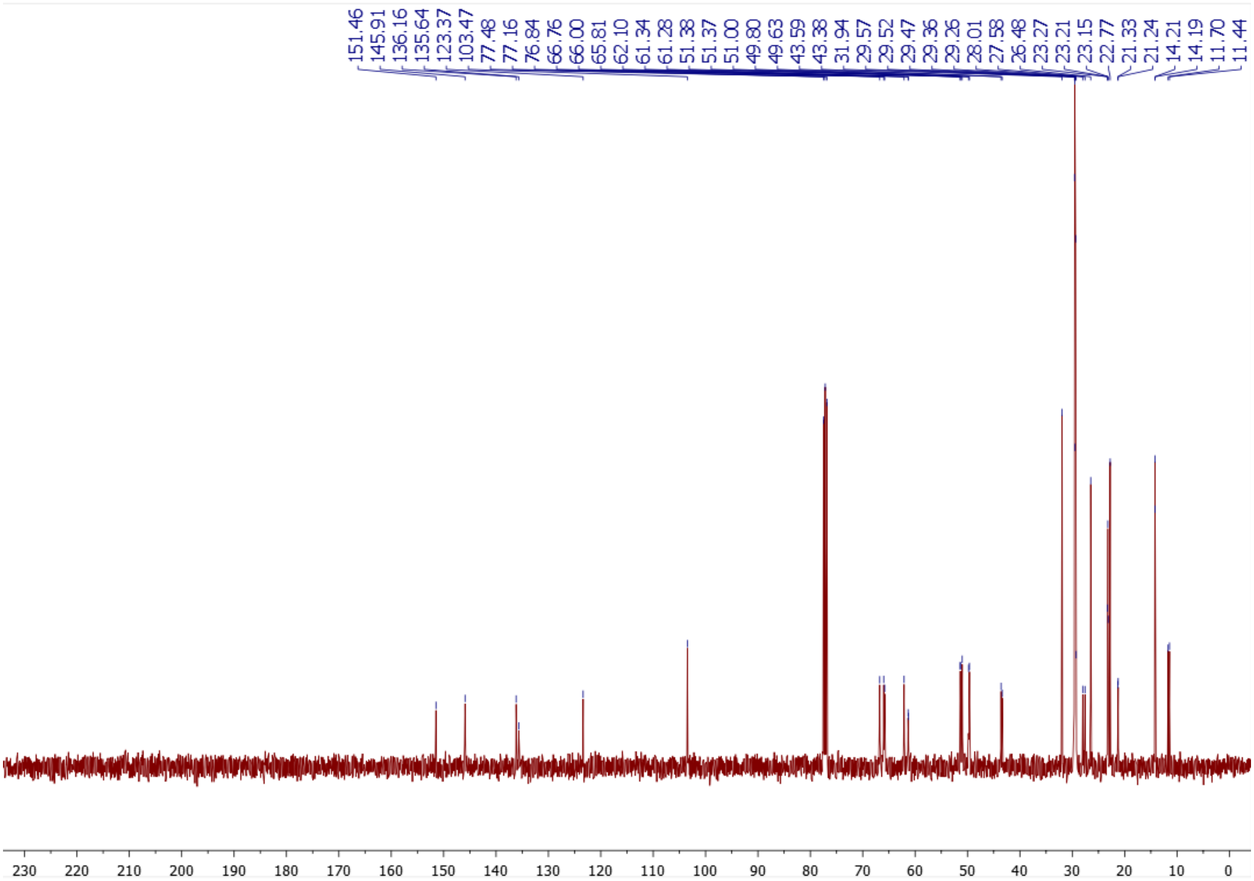

^{13}C{H} NMR spectrum of compound **5n$_{10}$**

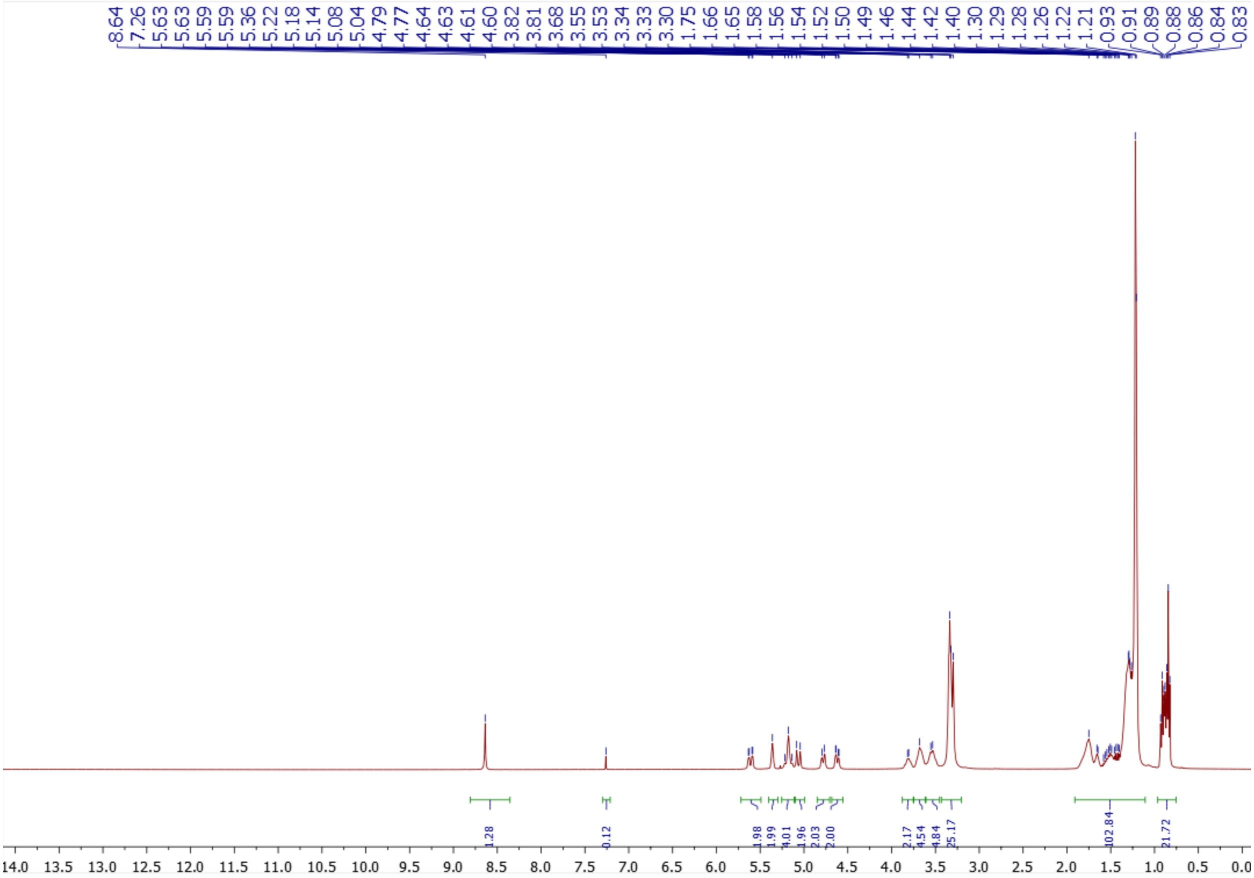

^{1}H NMR spectrum of compound **5n**12

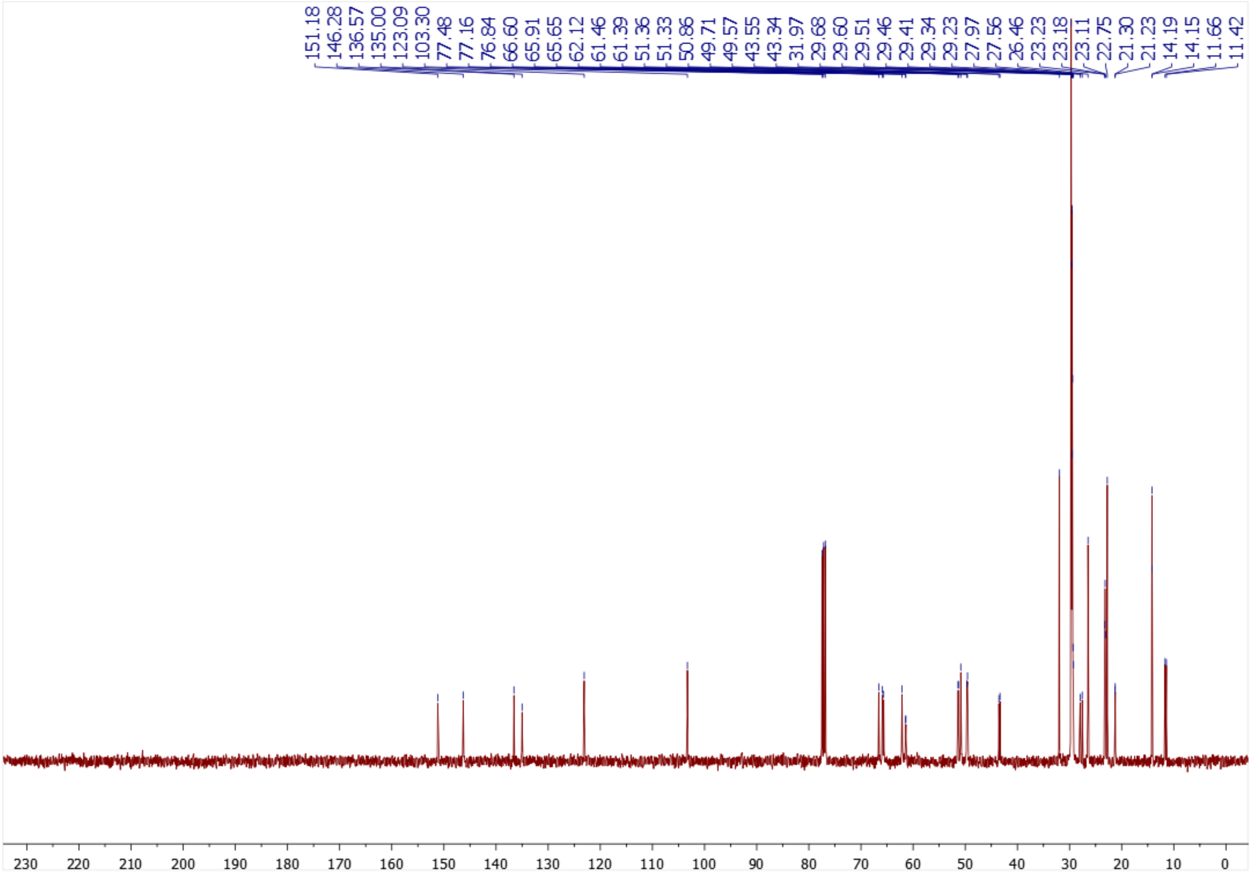

^{13}C{H} NMR spectrum of compound **5n**12

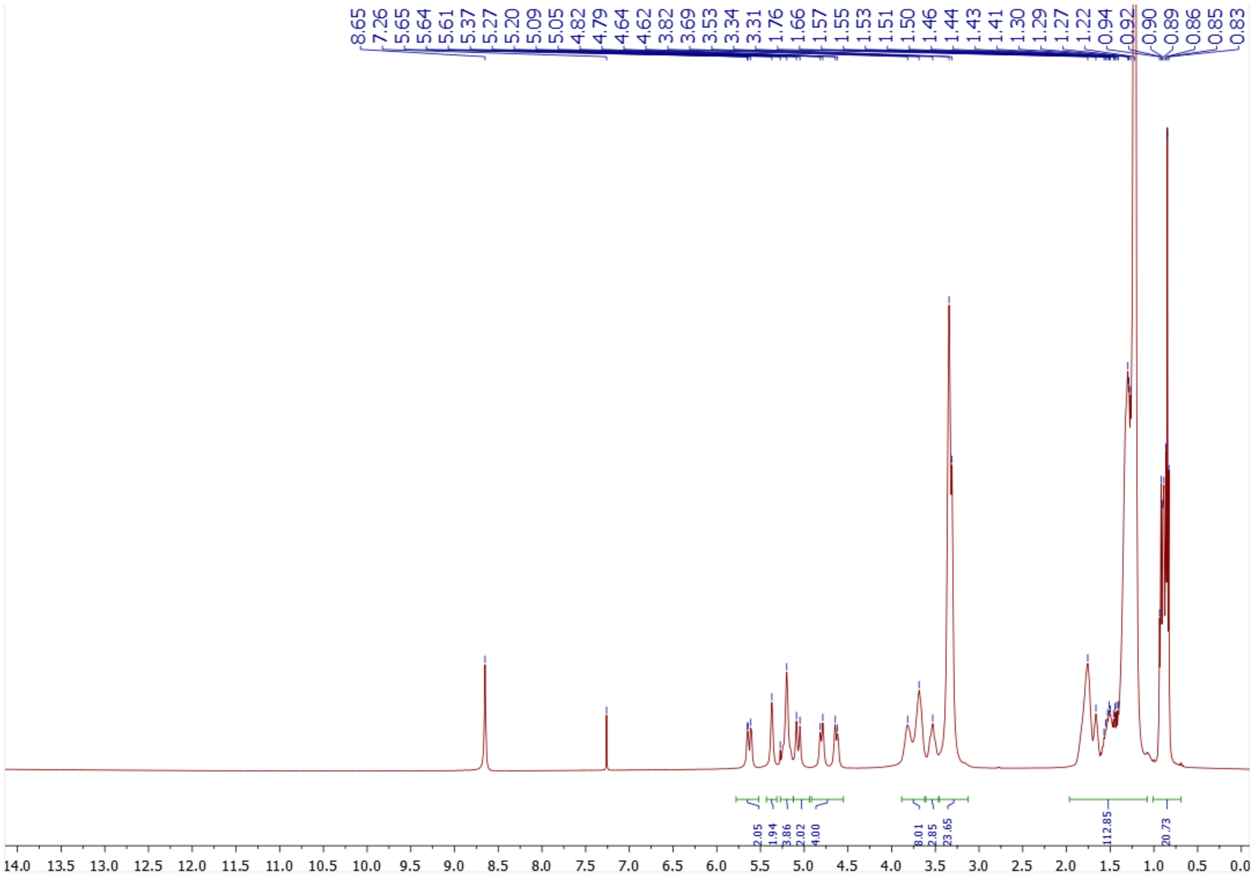

^{1}H NMR spectrum of compound **5n**$_{14}$

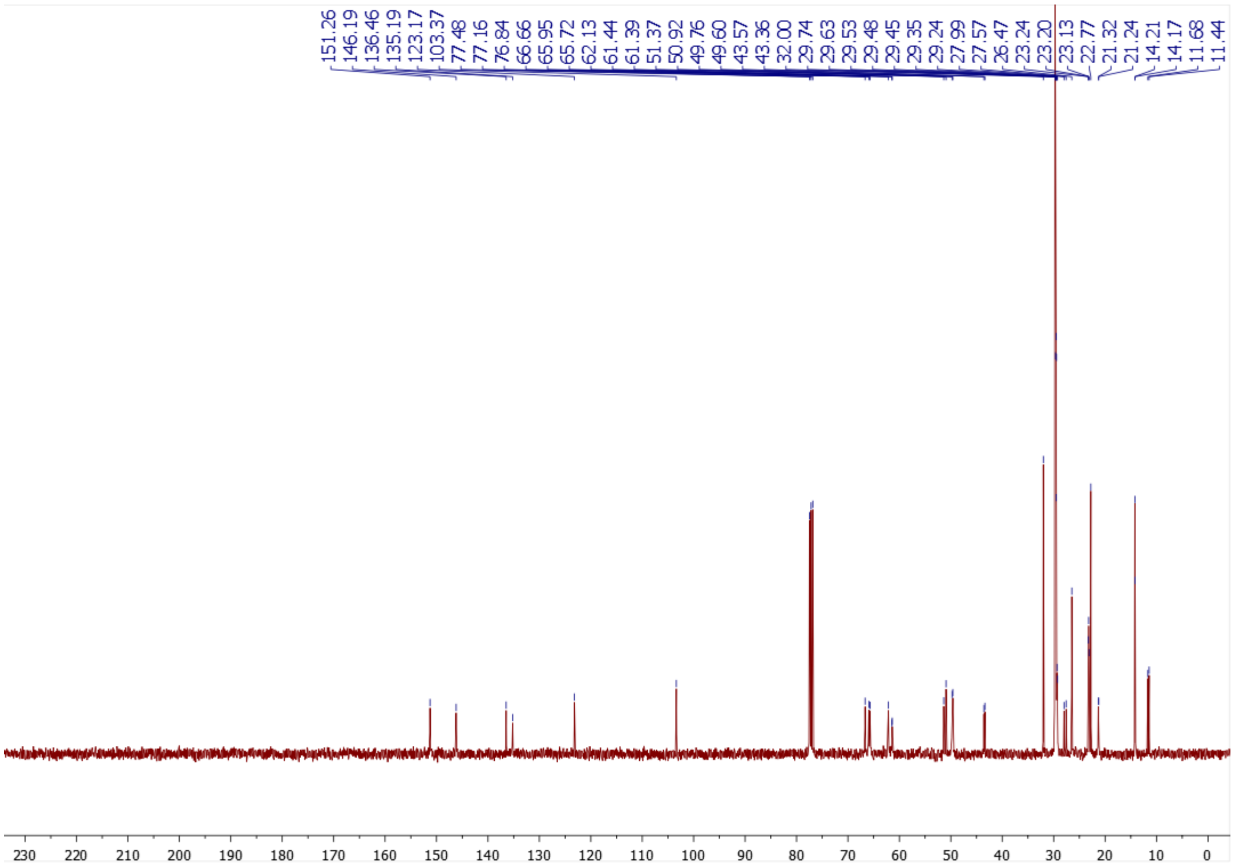

^{13}C{H} NMR spectrum of compound **5n**$_{14}$

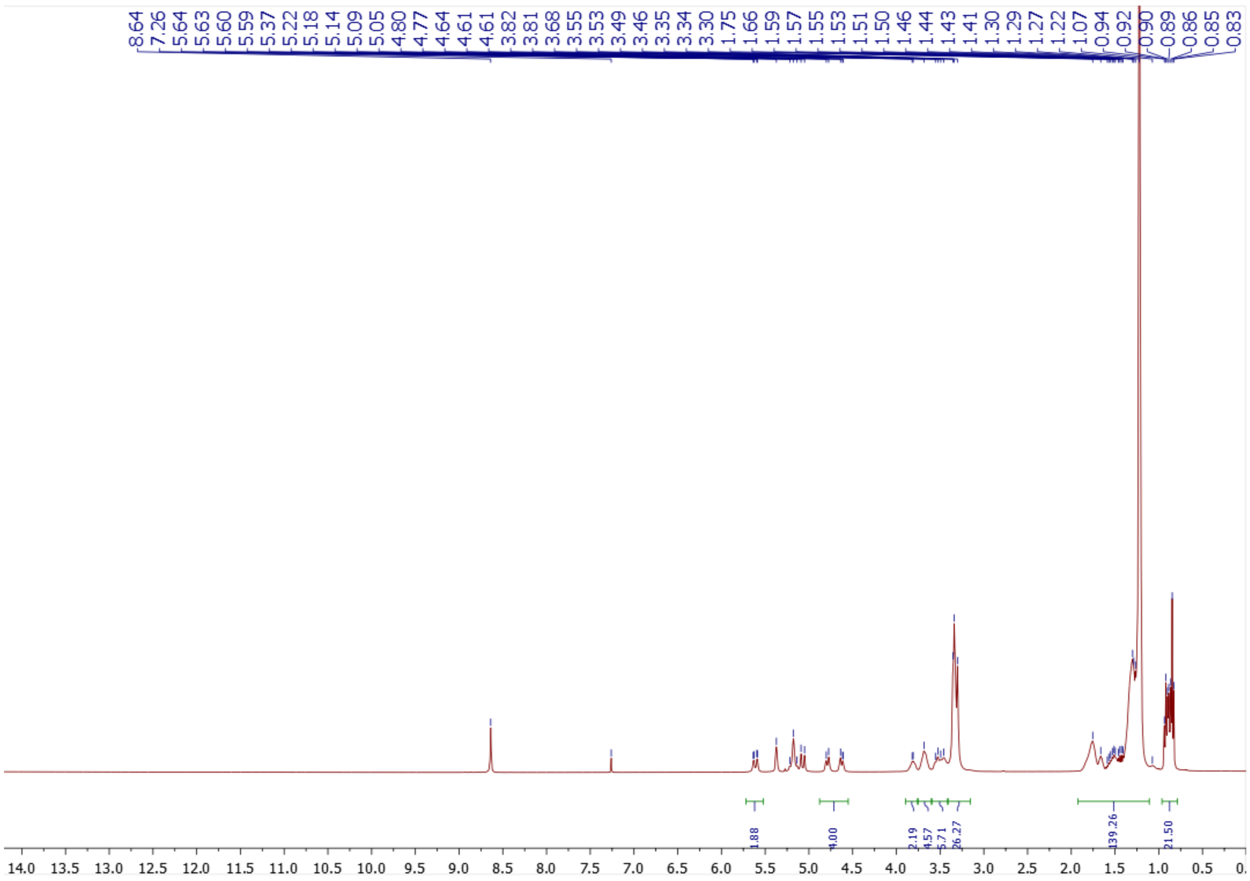

^{1}H NMR spectrum of compound **5n**16

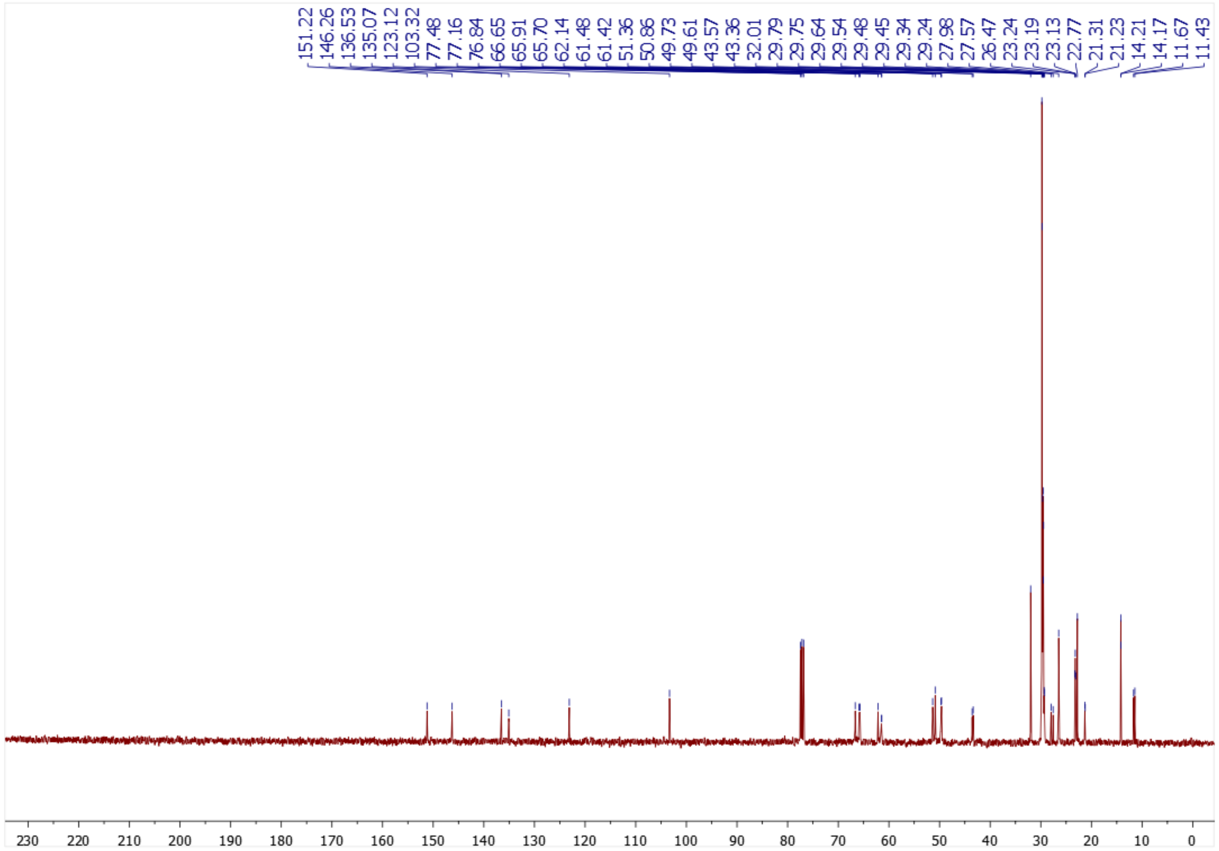

^{13}C{H} NMR spectrum of compound **5n**16

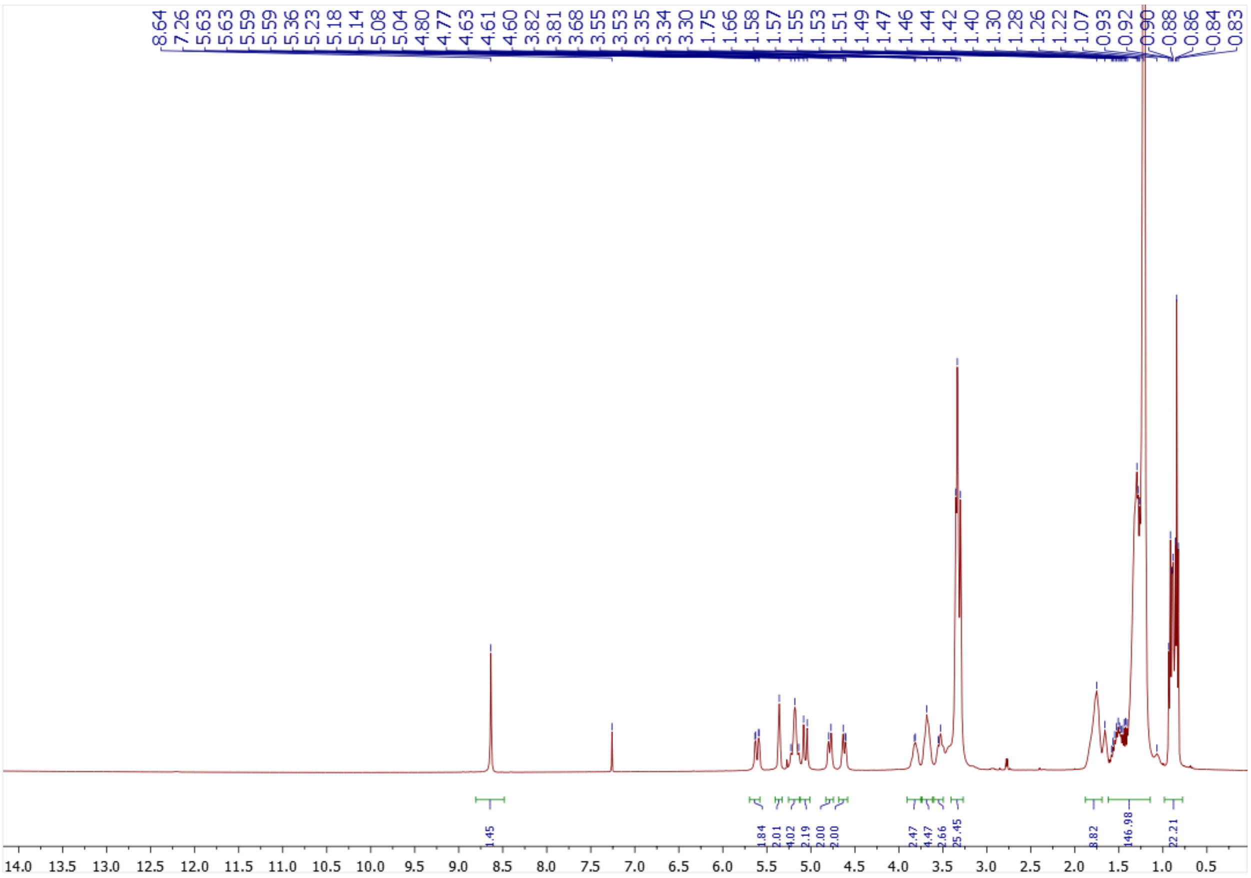

^{1}H NMR spectrum of compound **5n**$_{18}$

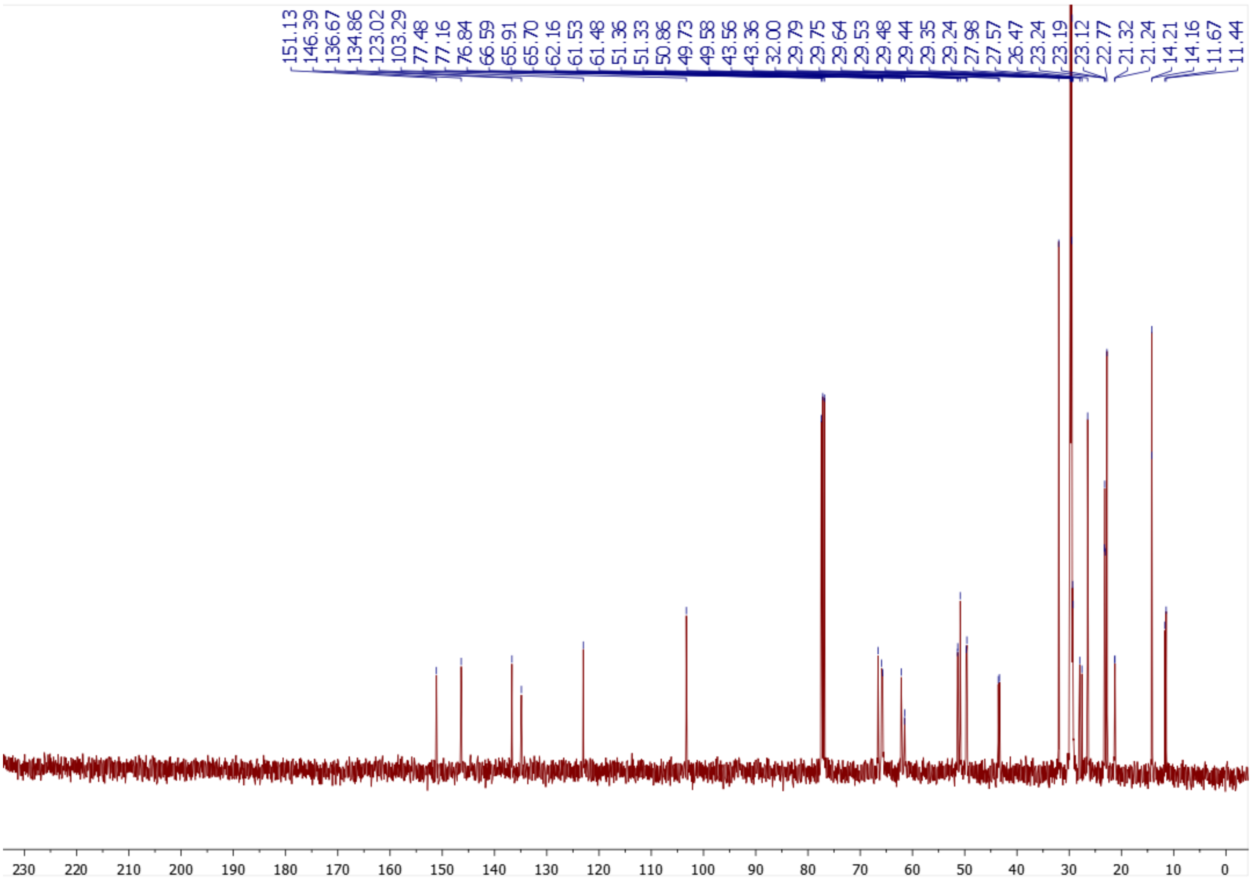

^{13}C{H} NMR spectrum of compound **5n**$_{18}$

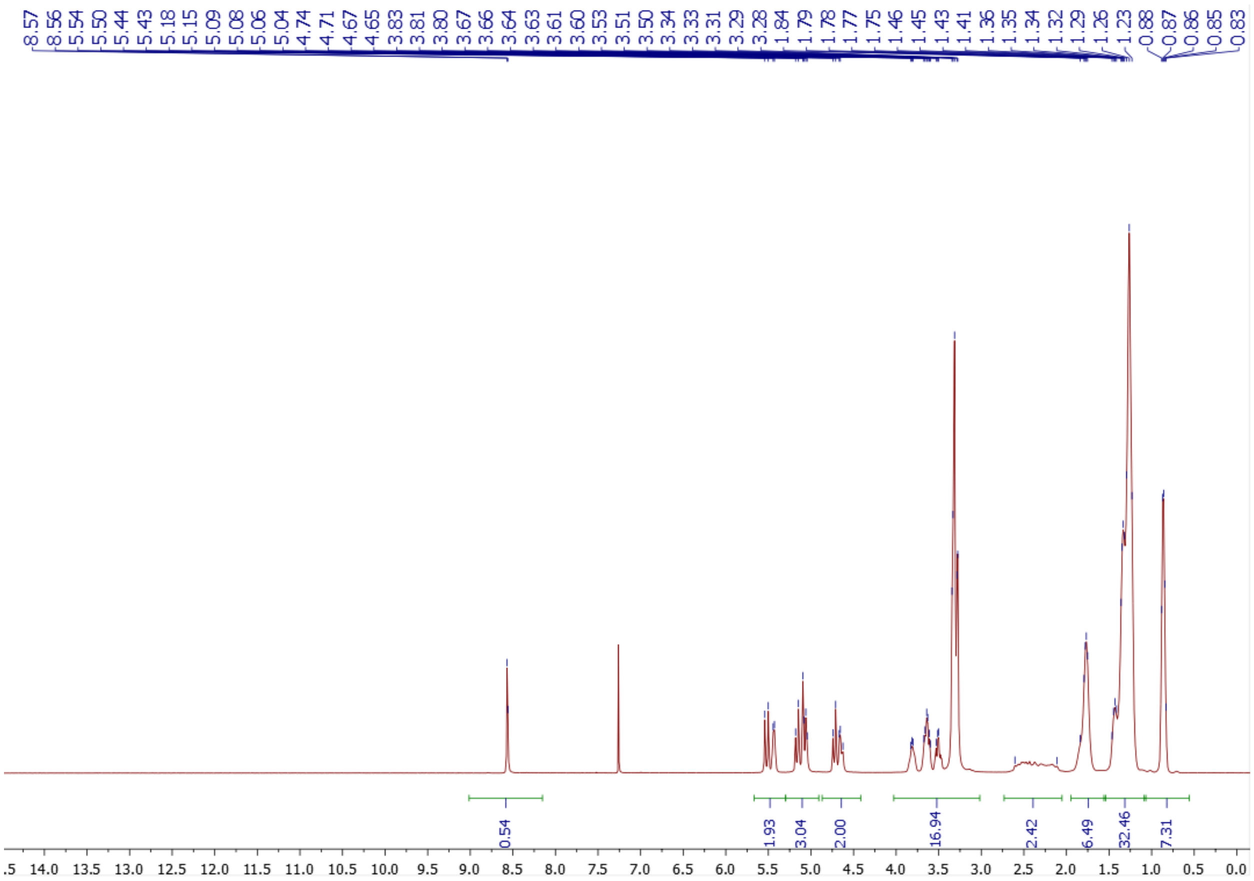

^{1}H NMR spectrum of compound **5o**8

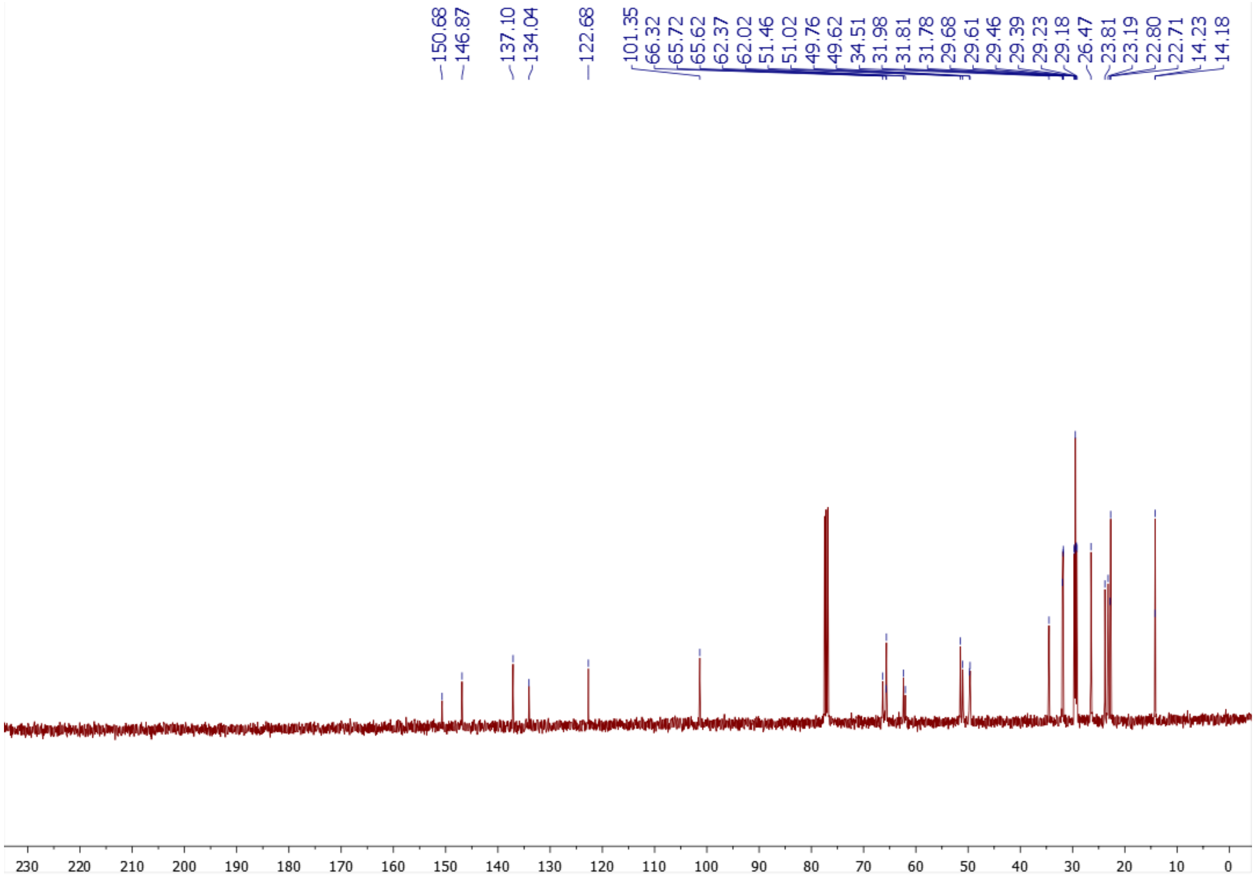

^{13}C{H} NMR spectrum of compound **5o**8

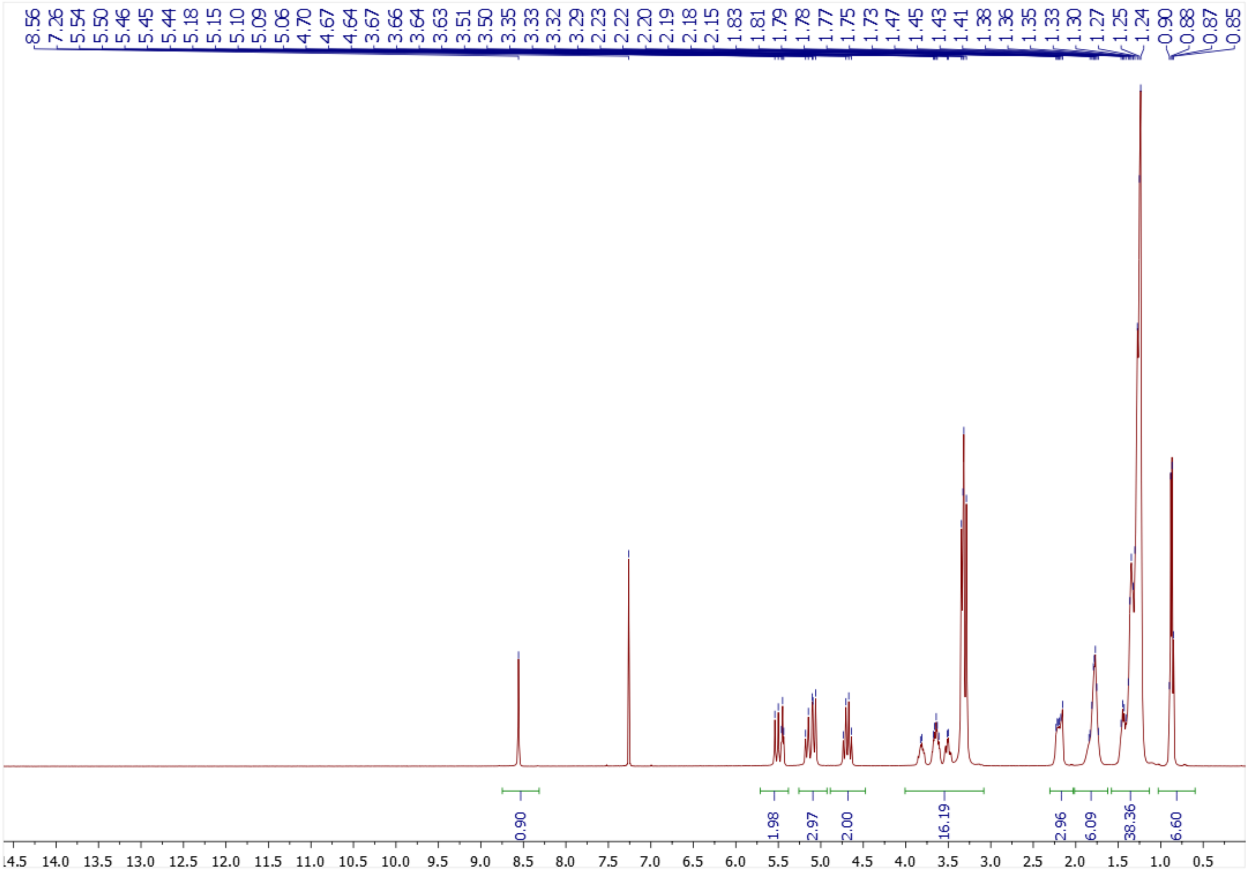

^{1}H NMR spectrum of compound **5o**$_{10}$

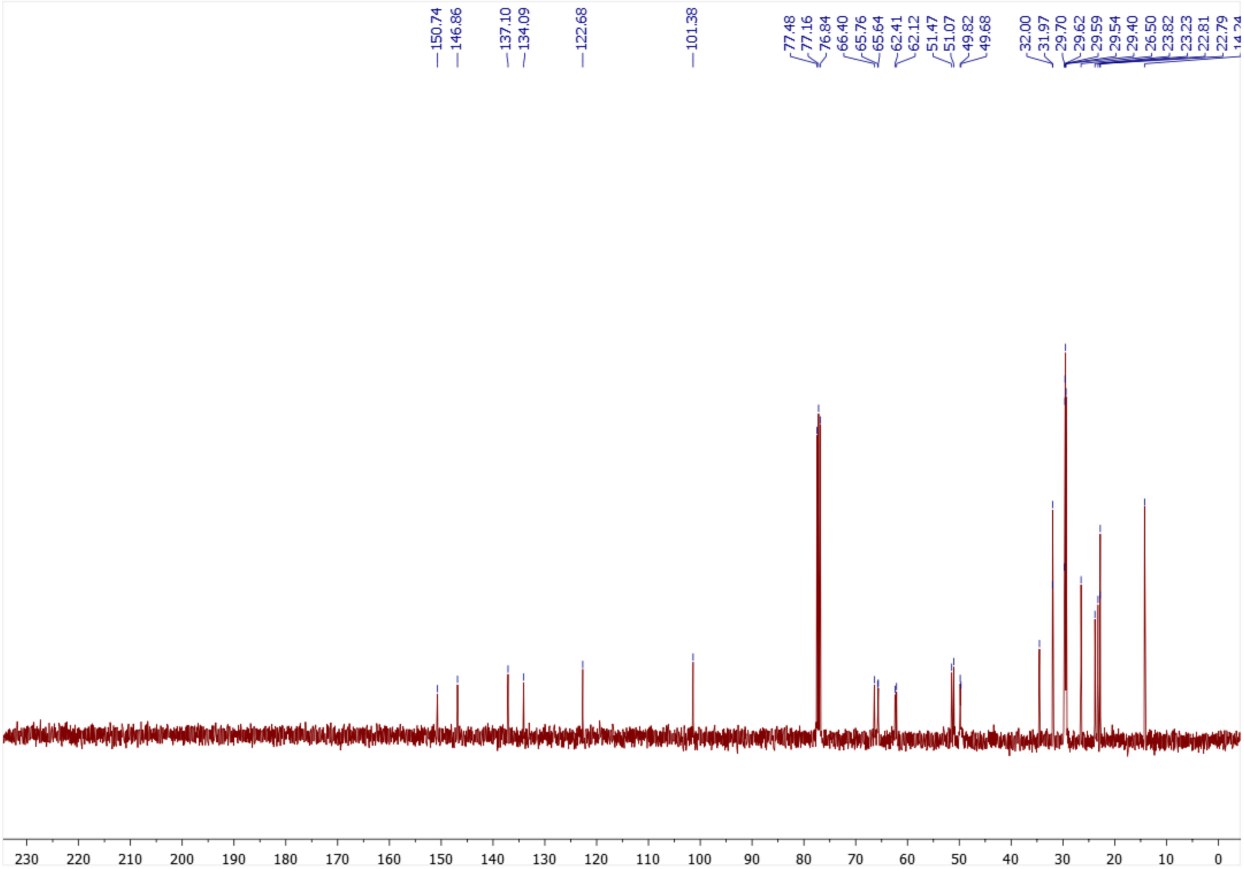

^{13}C{H} NMR spectrum of compound **5o**$_{10}$

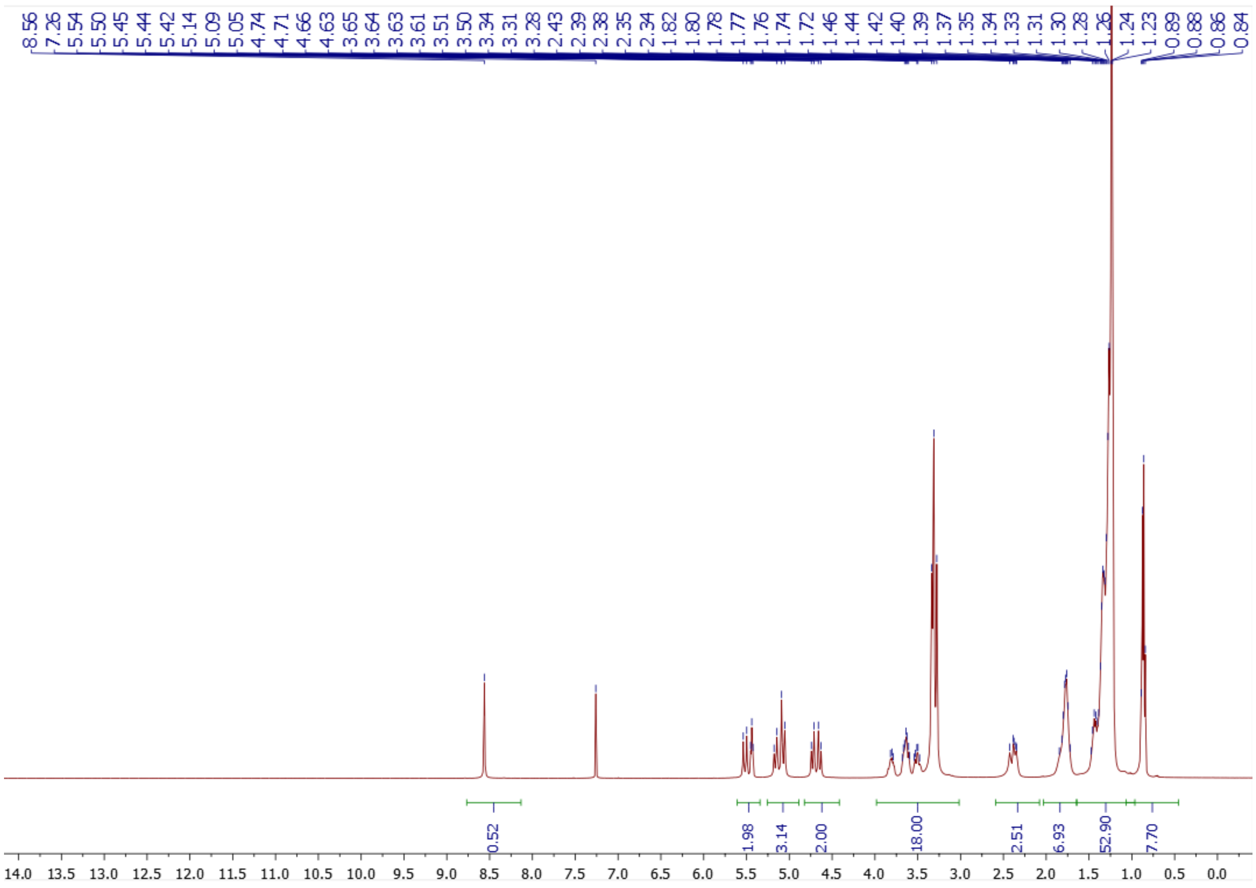

^{1}H NMR spectrum of compound **5o**12

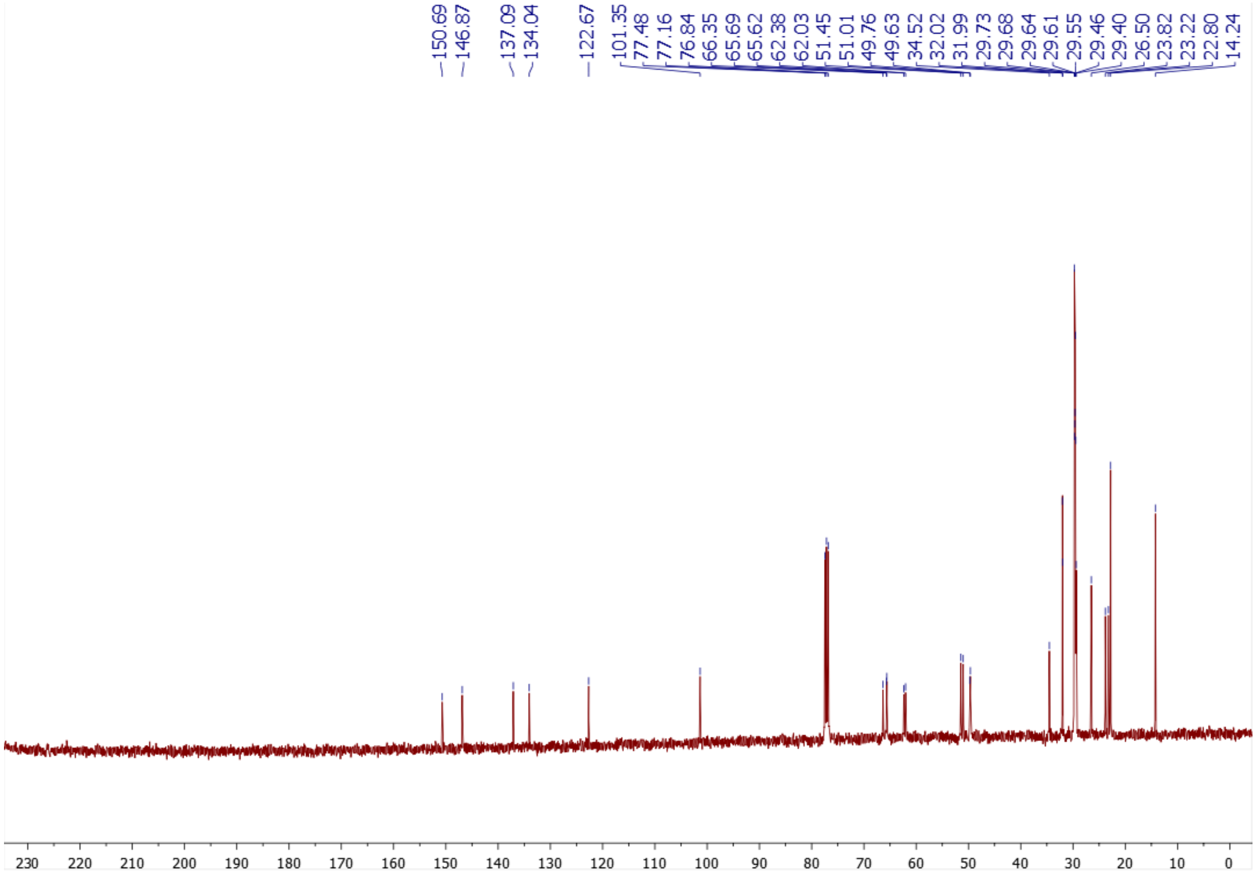

^{13}C{H} NMR spectrum of compound **5o**12

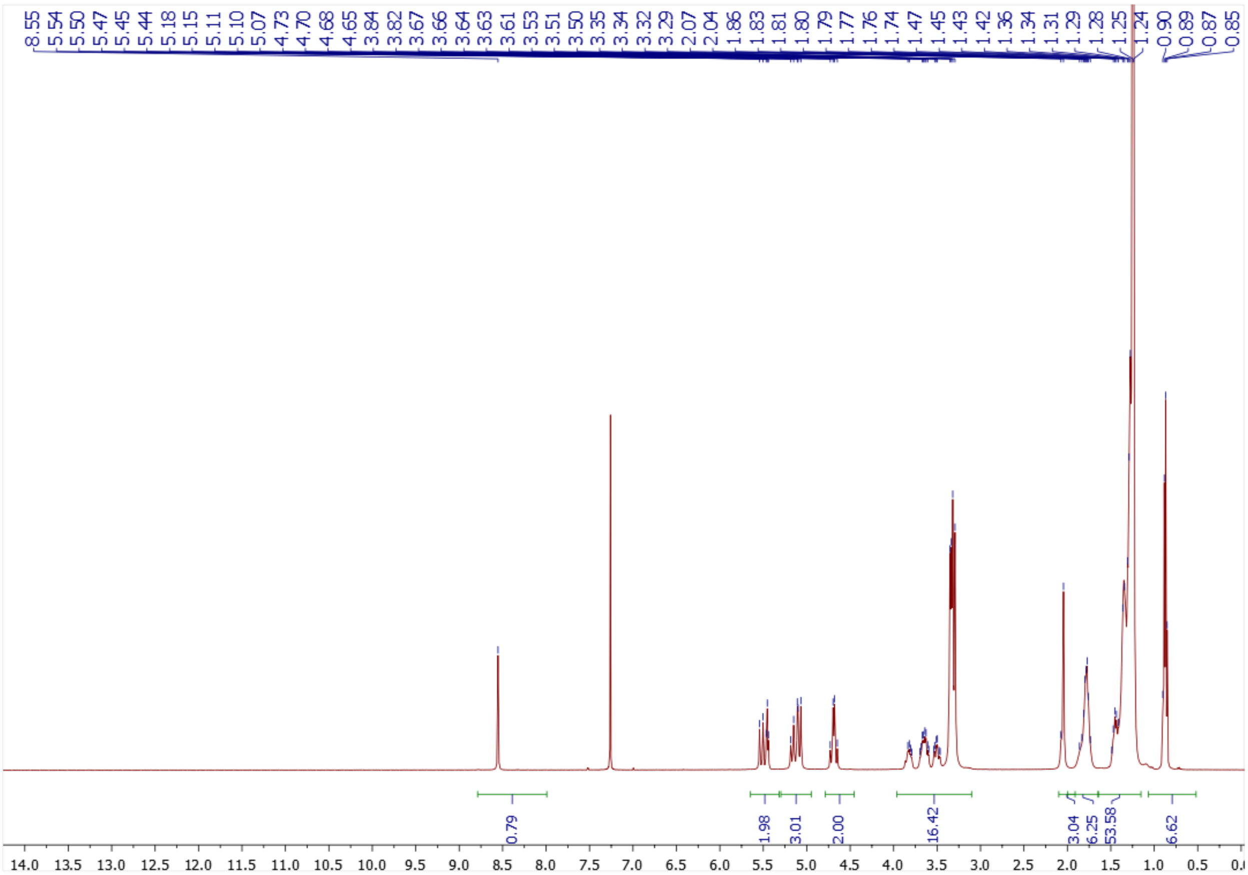

^{1}H NMR spectrum of compound **5o**$_{14}$

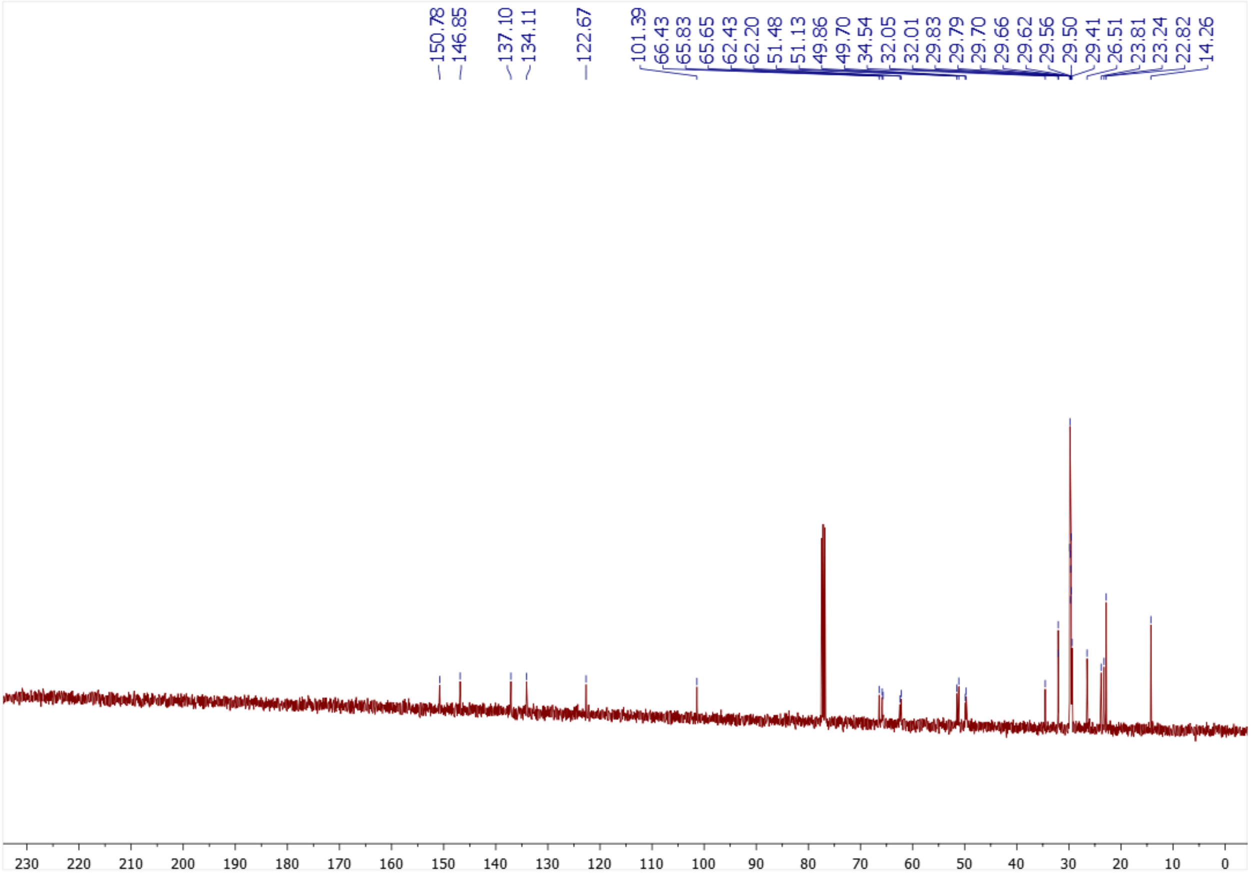

^{13}C{H} NMR spectrum of compound **5o**$_{14}$

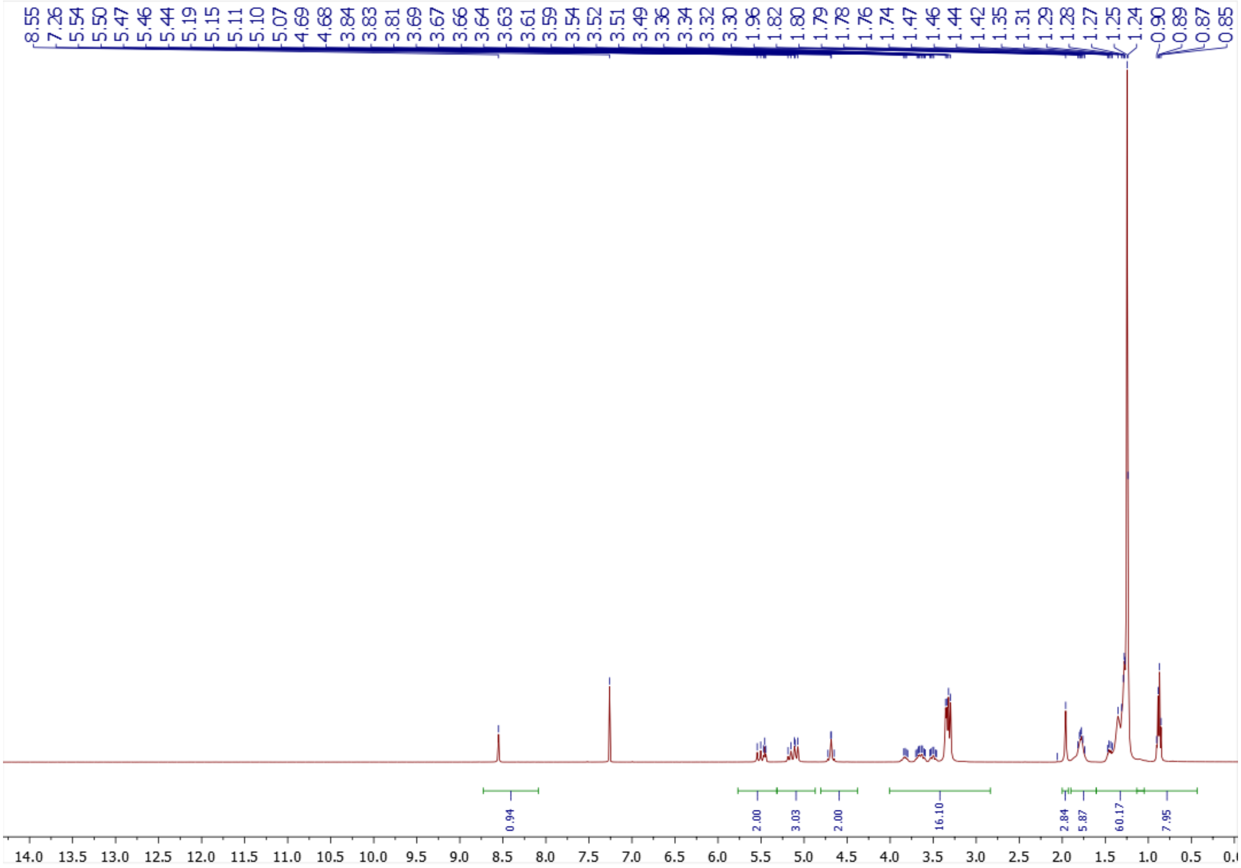

^{1}H NMR spectrum of compound **5o16**

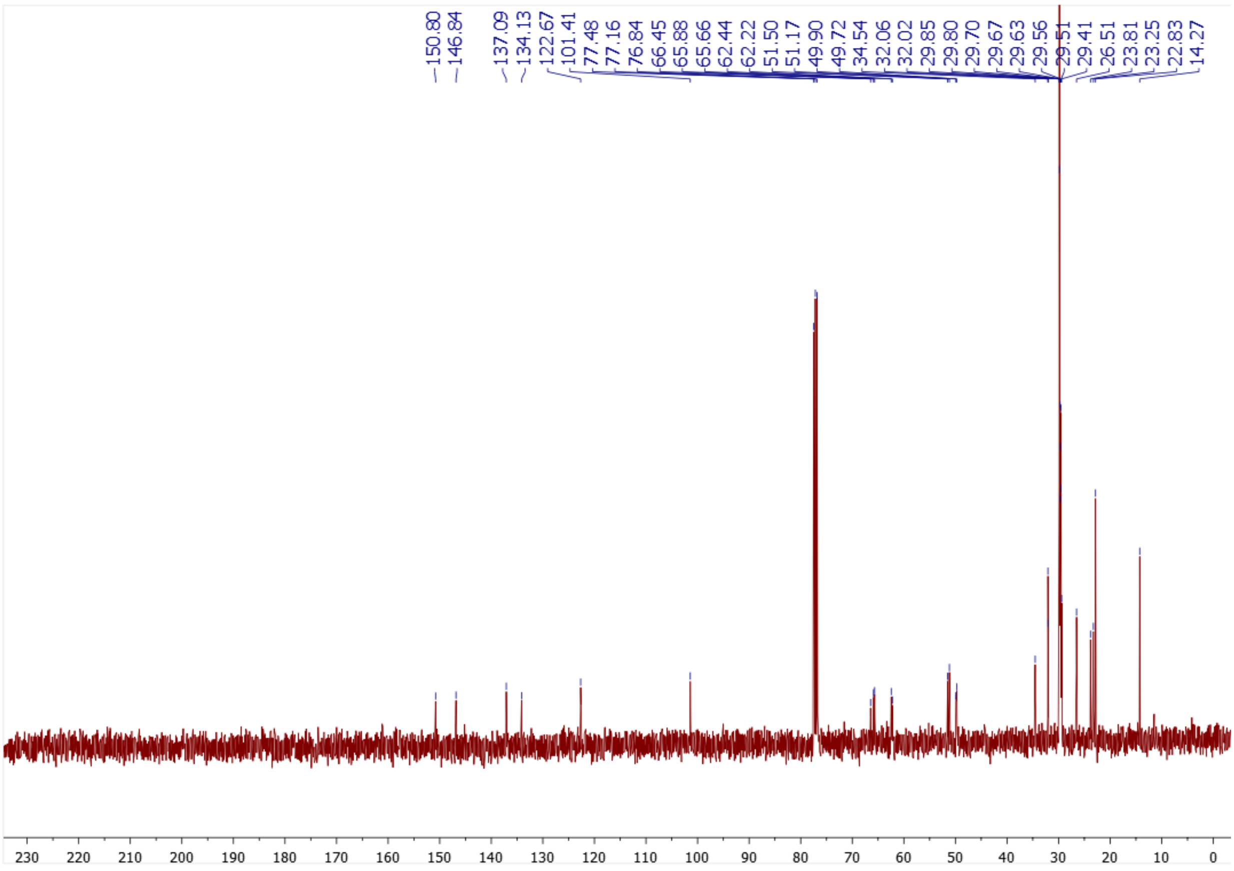

^{13}C{H} NMR spectrum of compound **5o16**

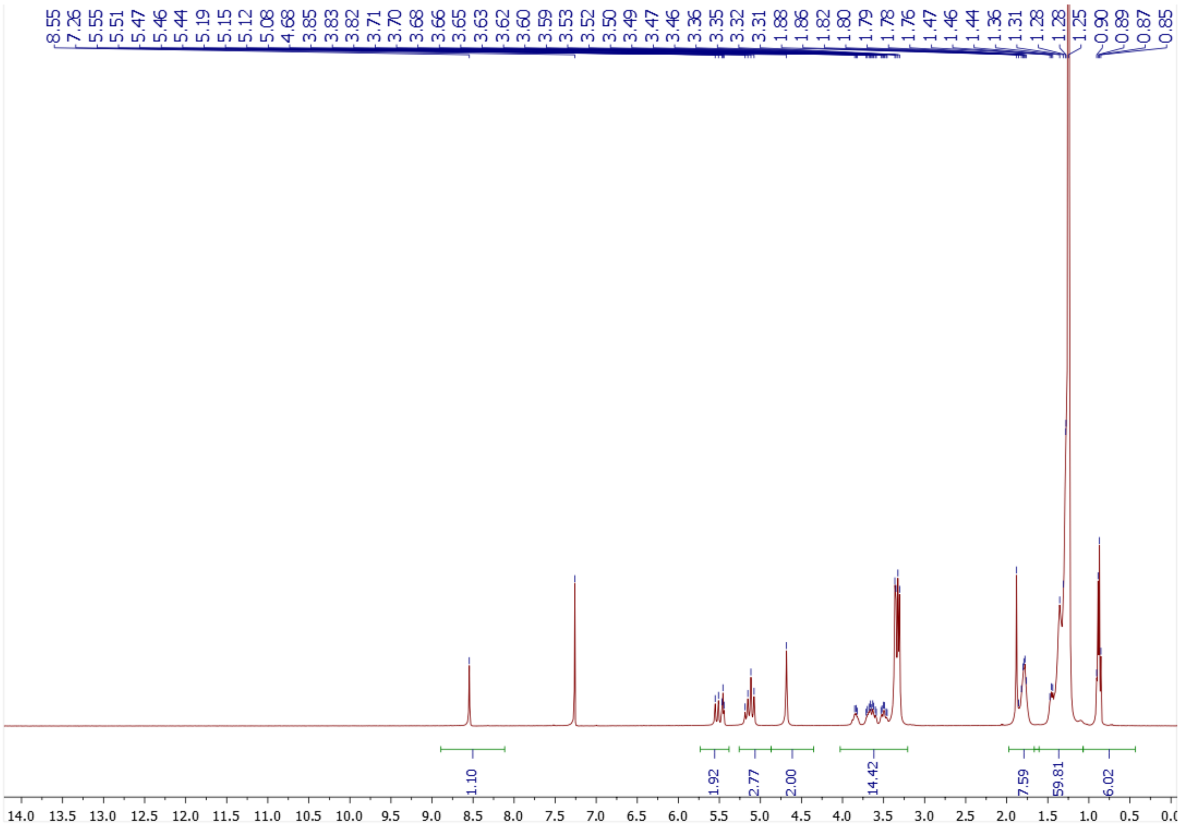

^{1}H NMR spectrum of compound **5o18**

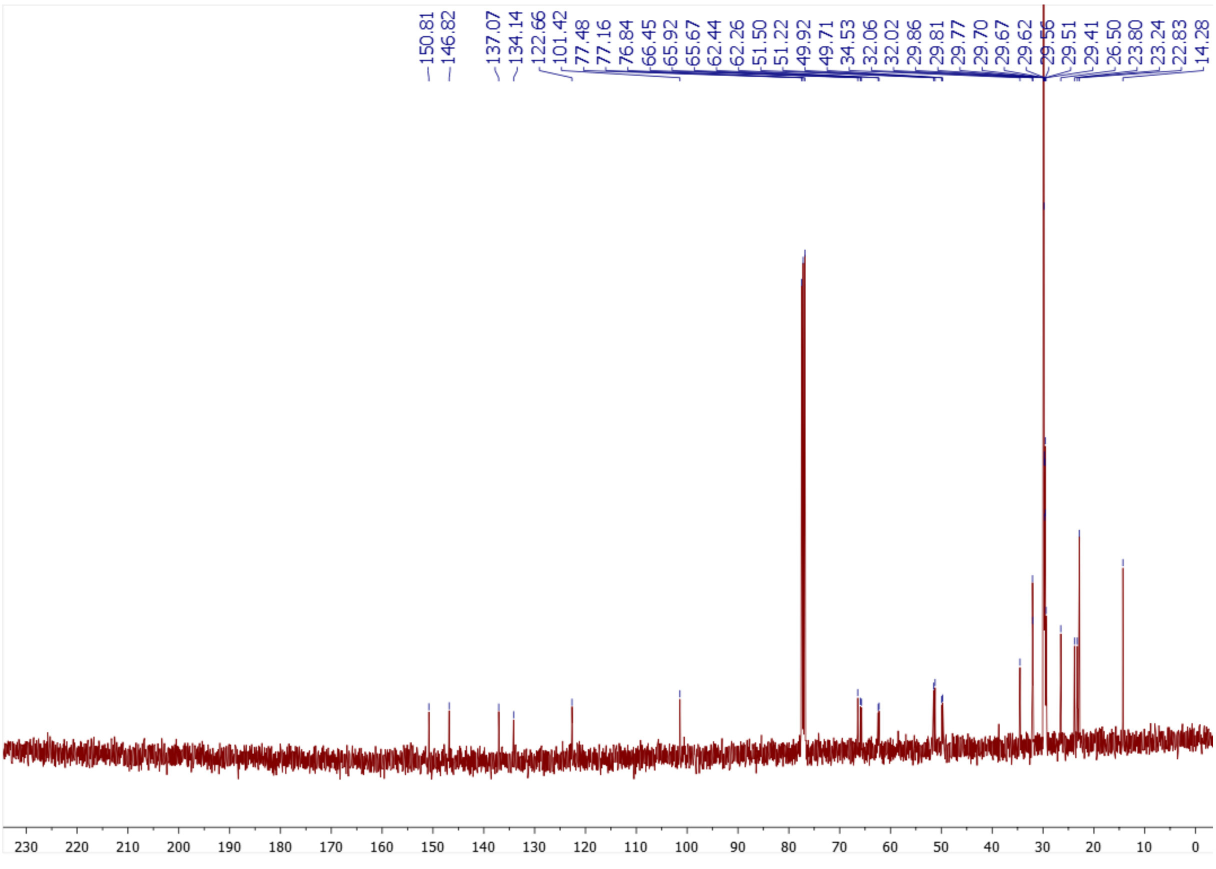

^{13}C{H} NMR spectrum of compound **5o18**

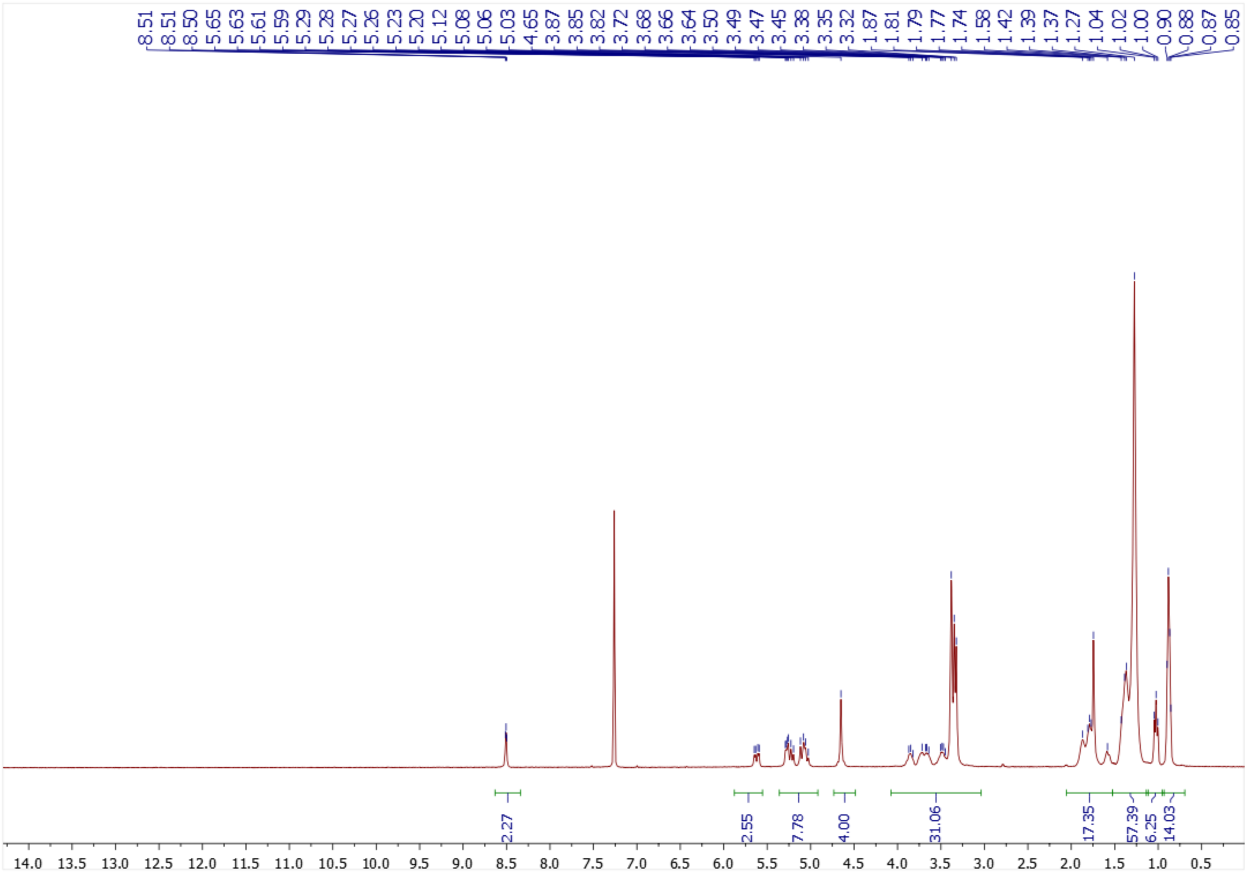

^{1}H NMR spectrum of compound **5p$_8$**

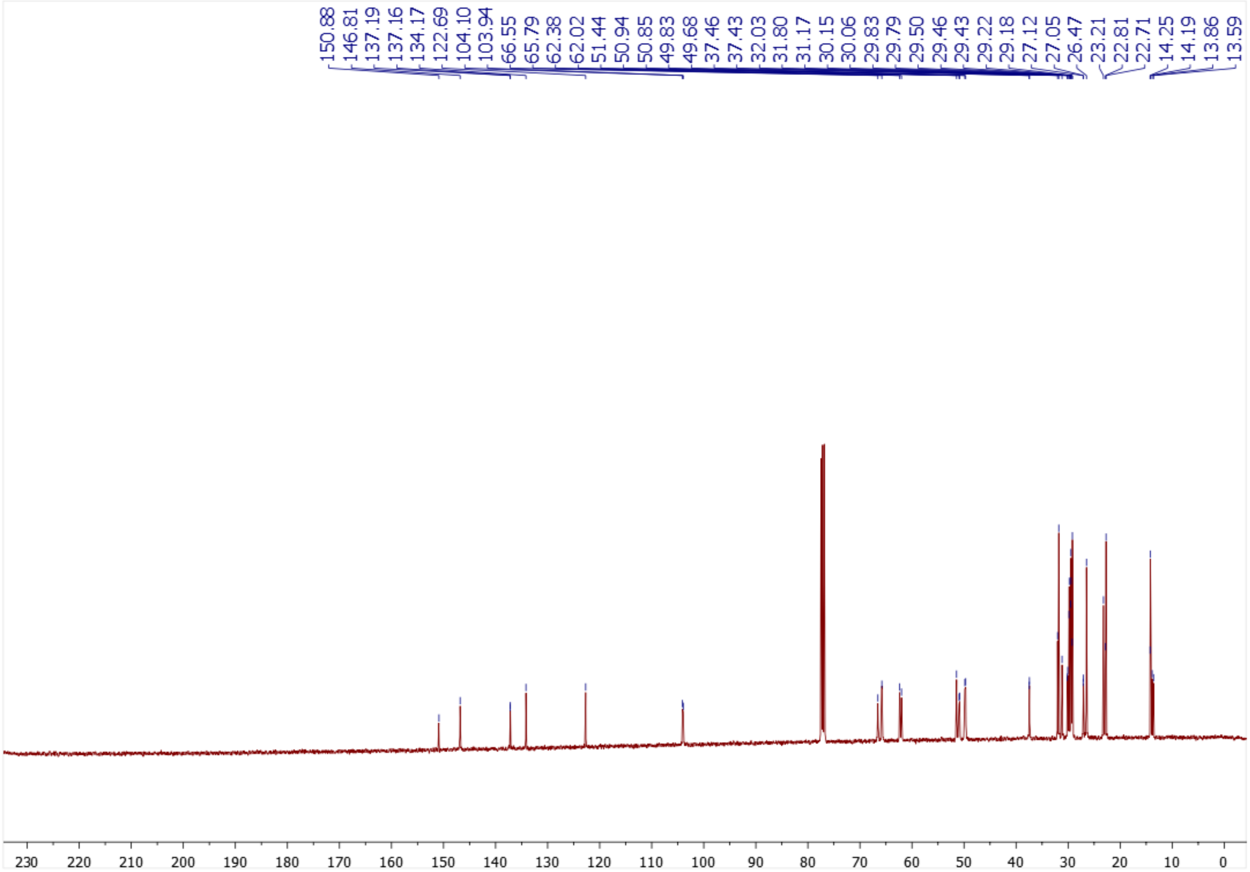

$^{13}C\{H\}$ NMR spectrum of compound **5p$_8$**

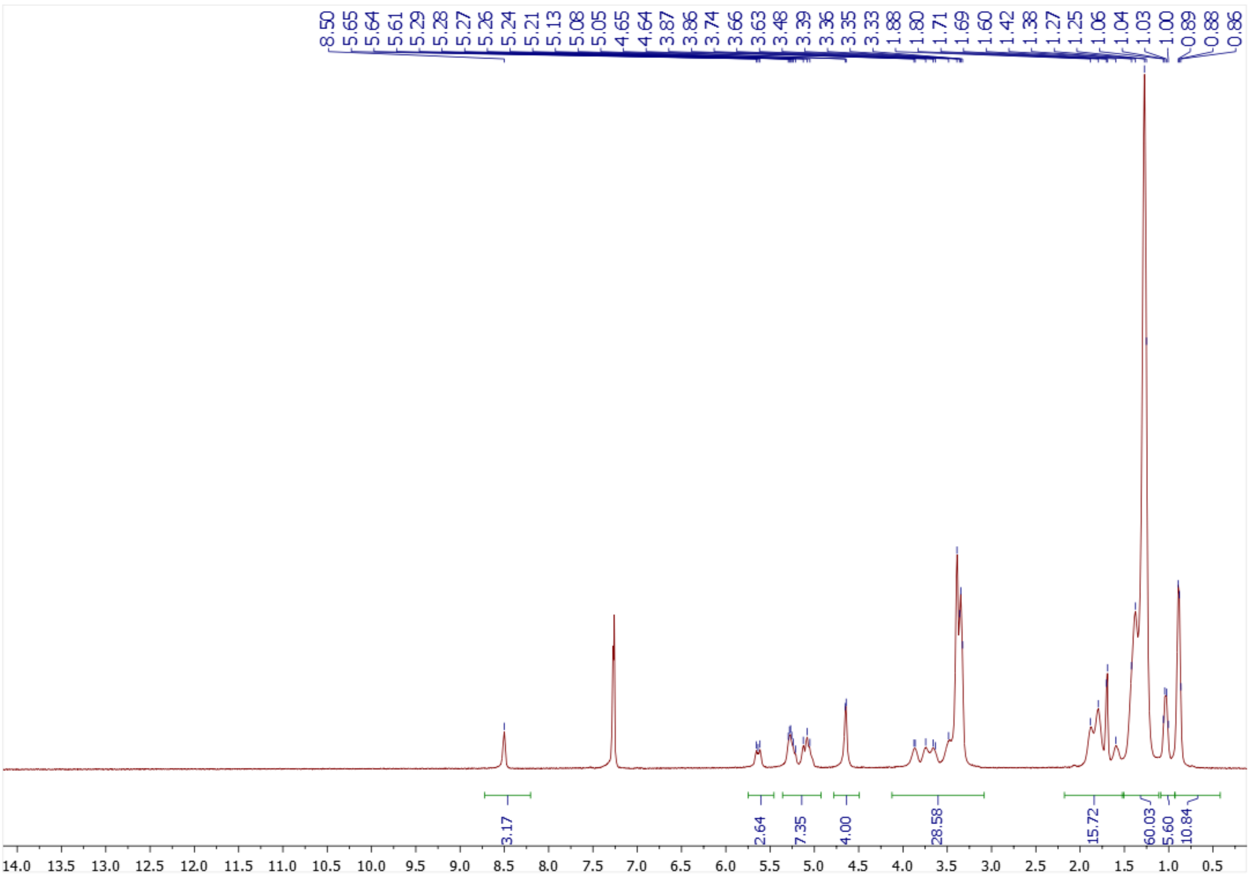

^{1}H NMR spectrum of compound **5p10**

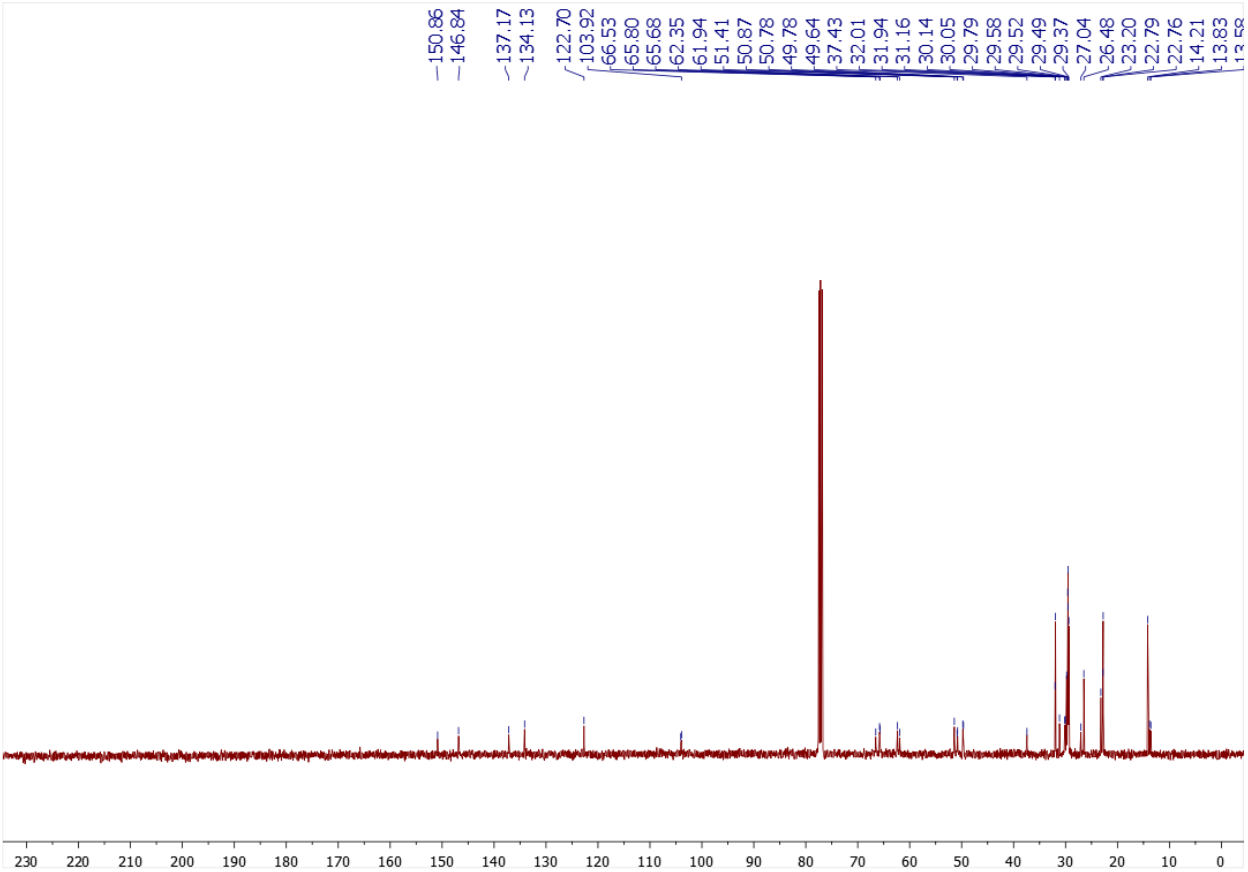

^{13}C{H} NMR spectrum of compound **5p10**

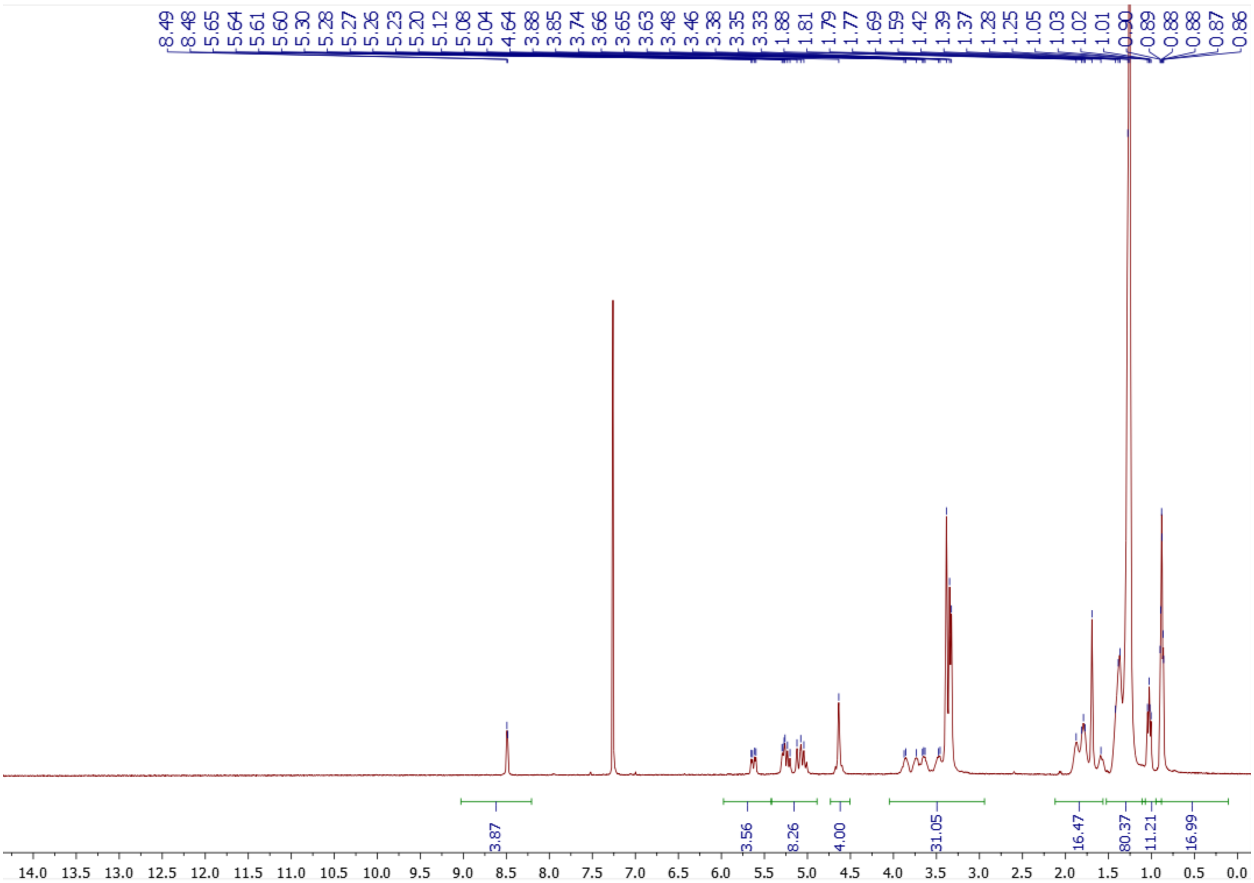

^{1}H NMR spectrum of compound **5p$_{12}$**

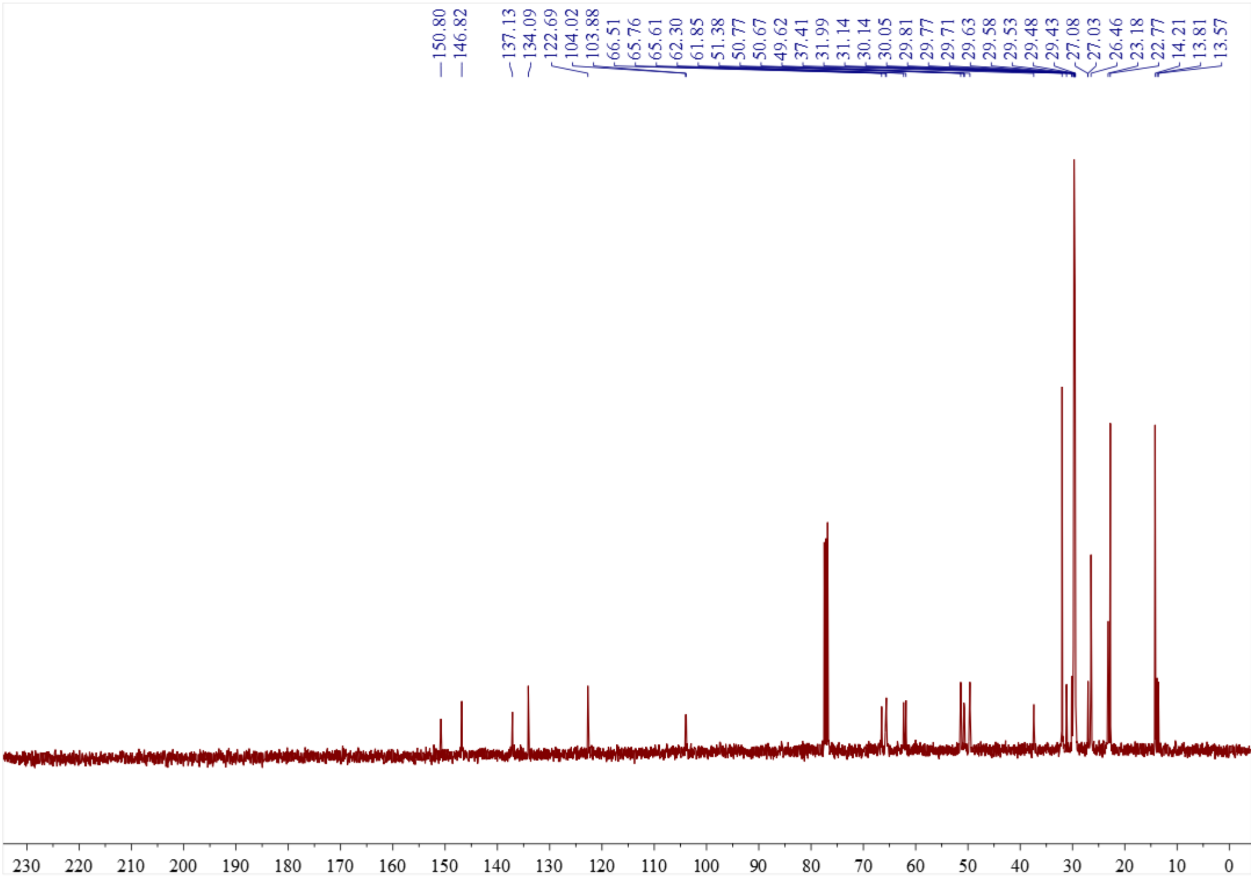

^{13}C{H} NMR spectrum of compound **5p$_{12}$**

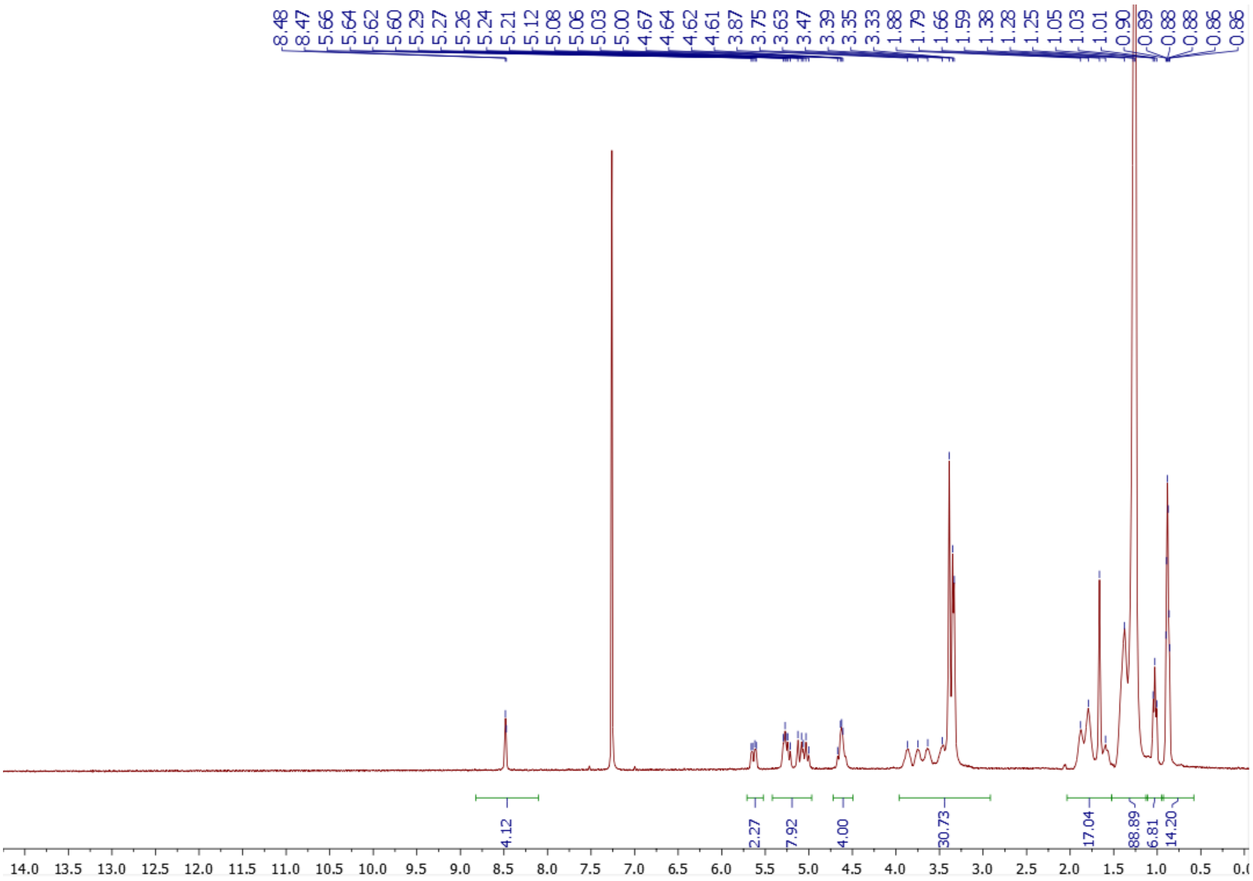

^{1}H NMR spectrum of compound **5p₁₄**

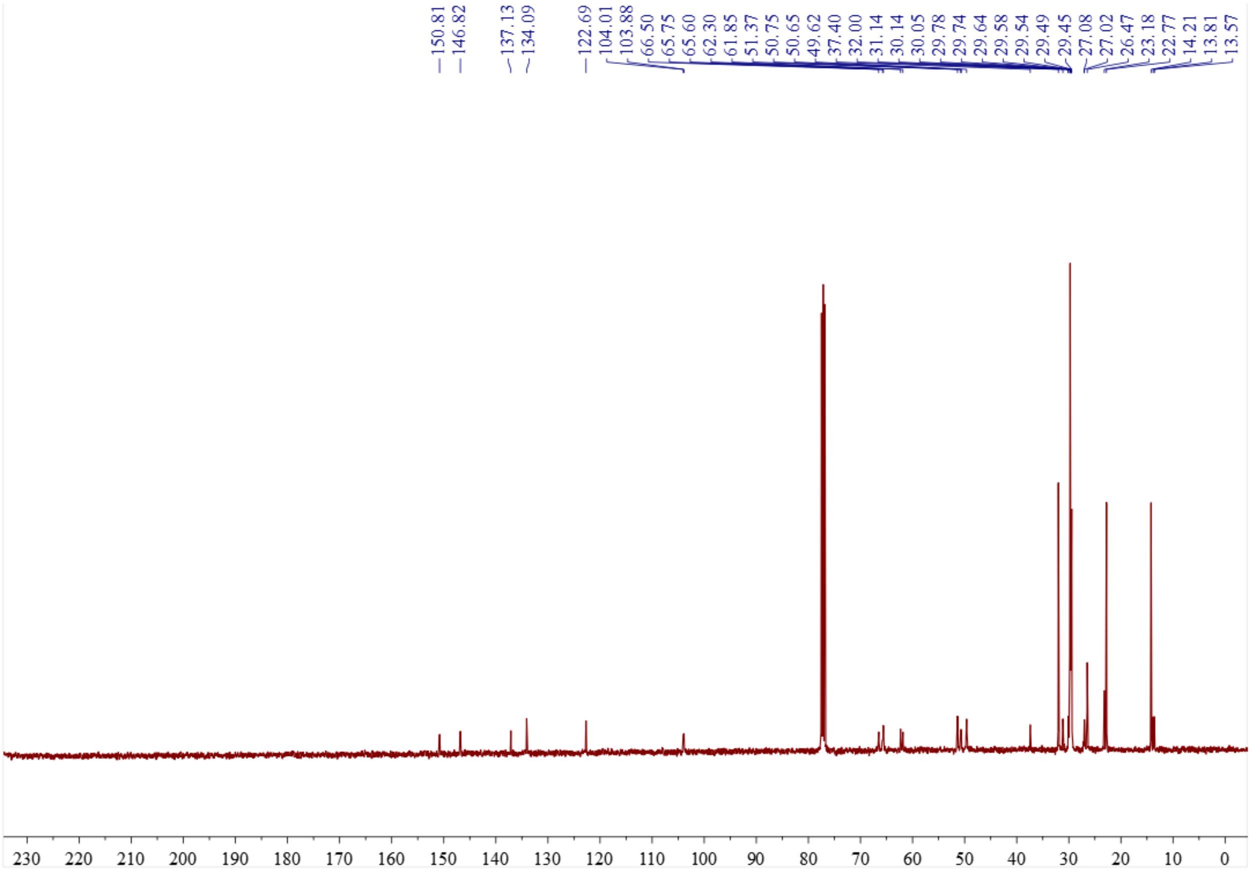

^{13}C{H} NMR spectrum of compound **5p₁₄**

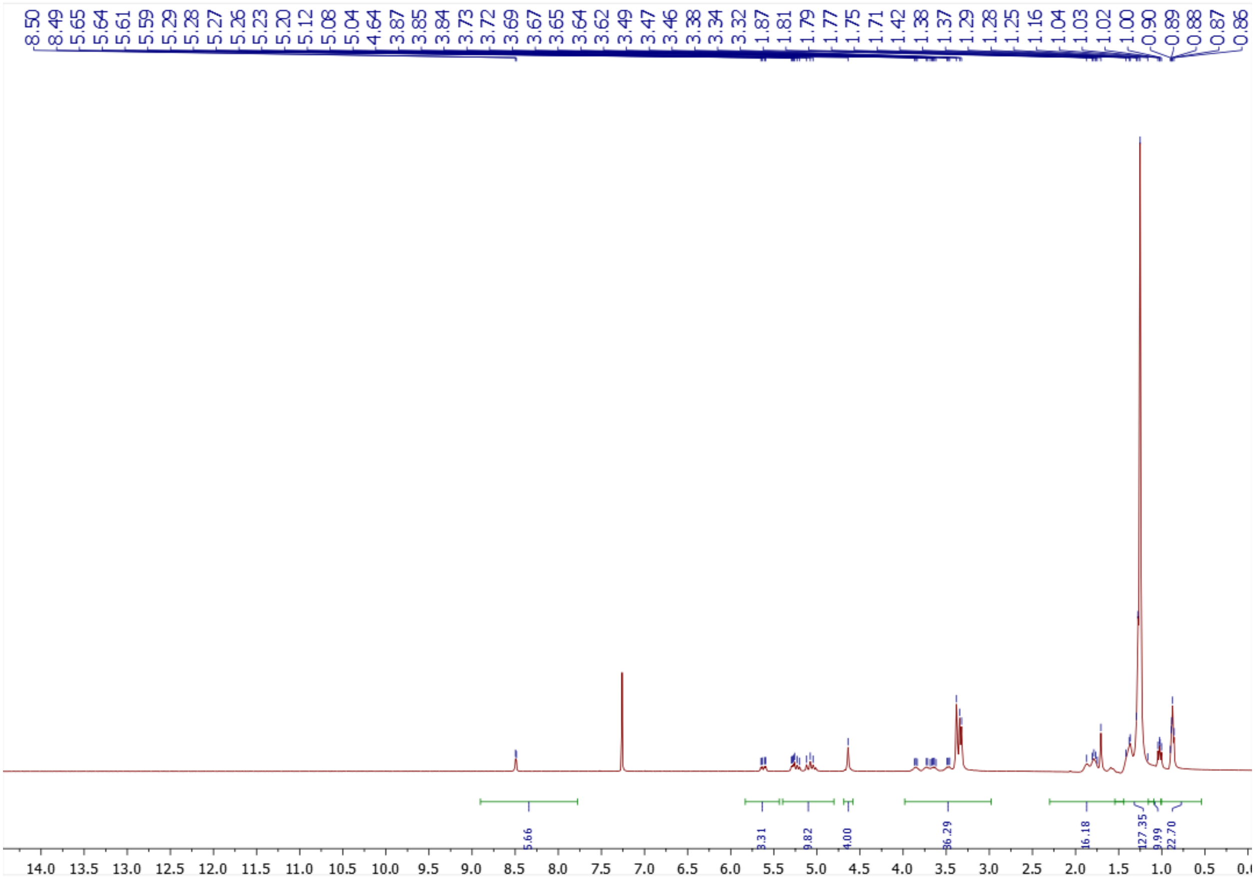

^{1}H NMR spectrum of compound **5p**16

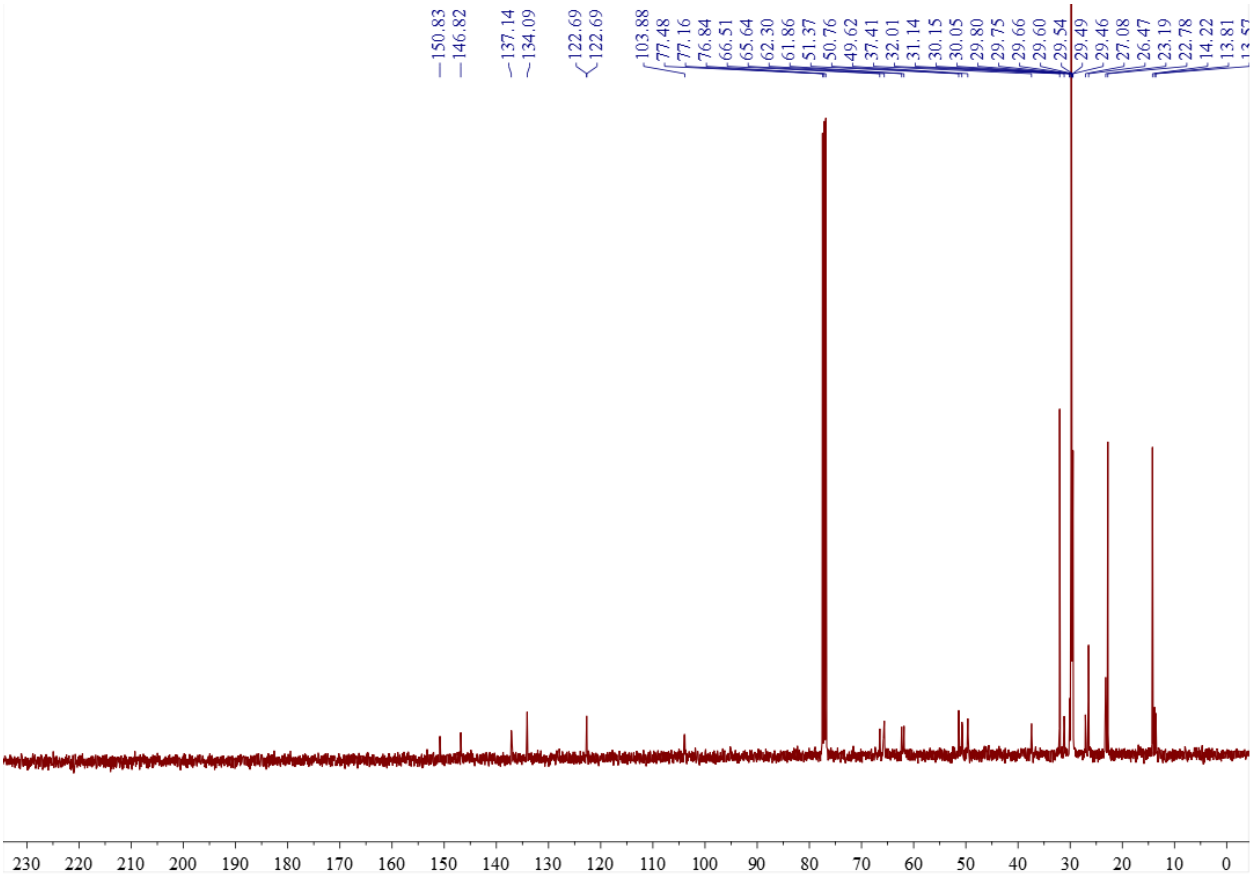

^{13}C{H} NMR spectrum of compound **5p**16

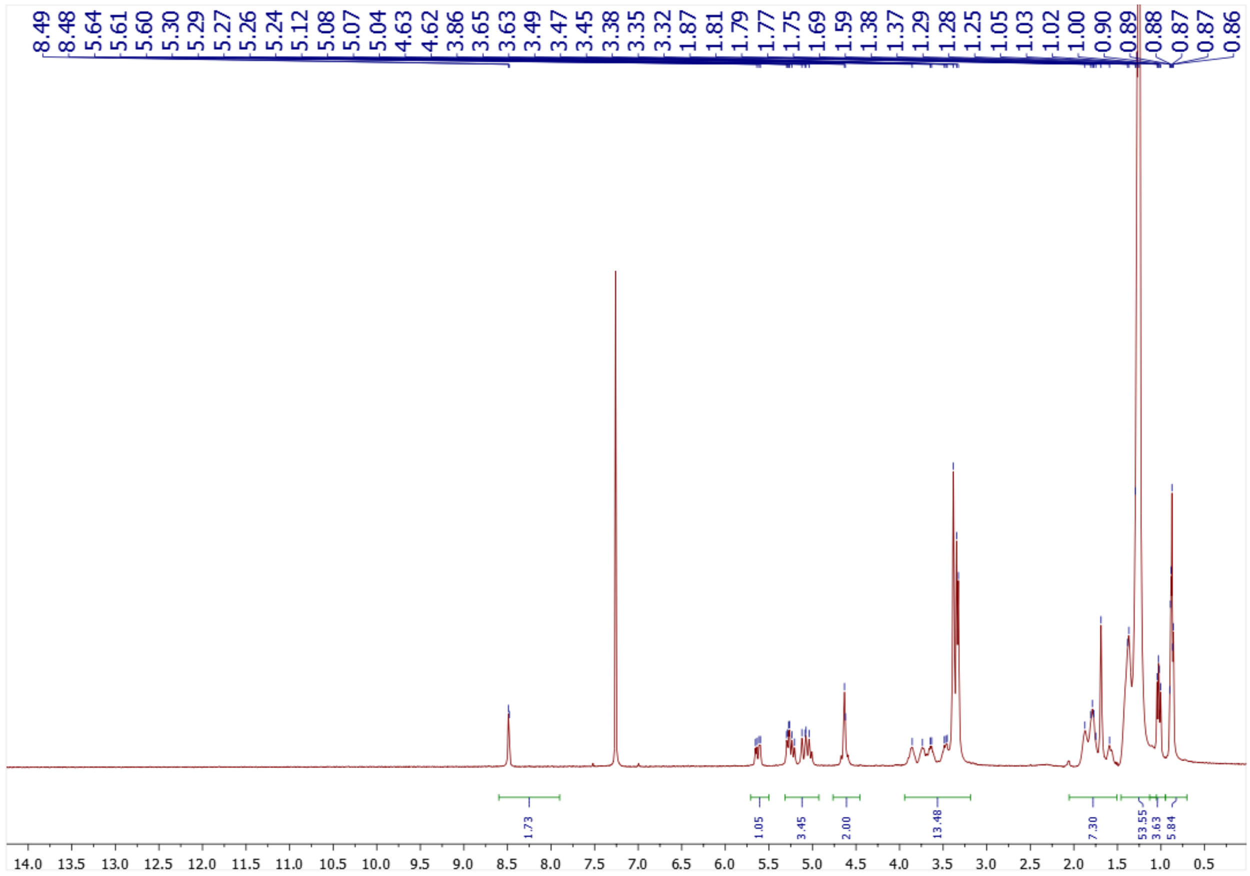

^{1}H NMR spectrum of compound **5p**$_{18}$

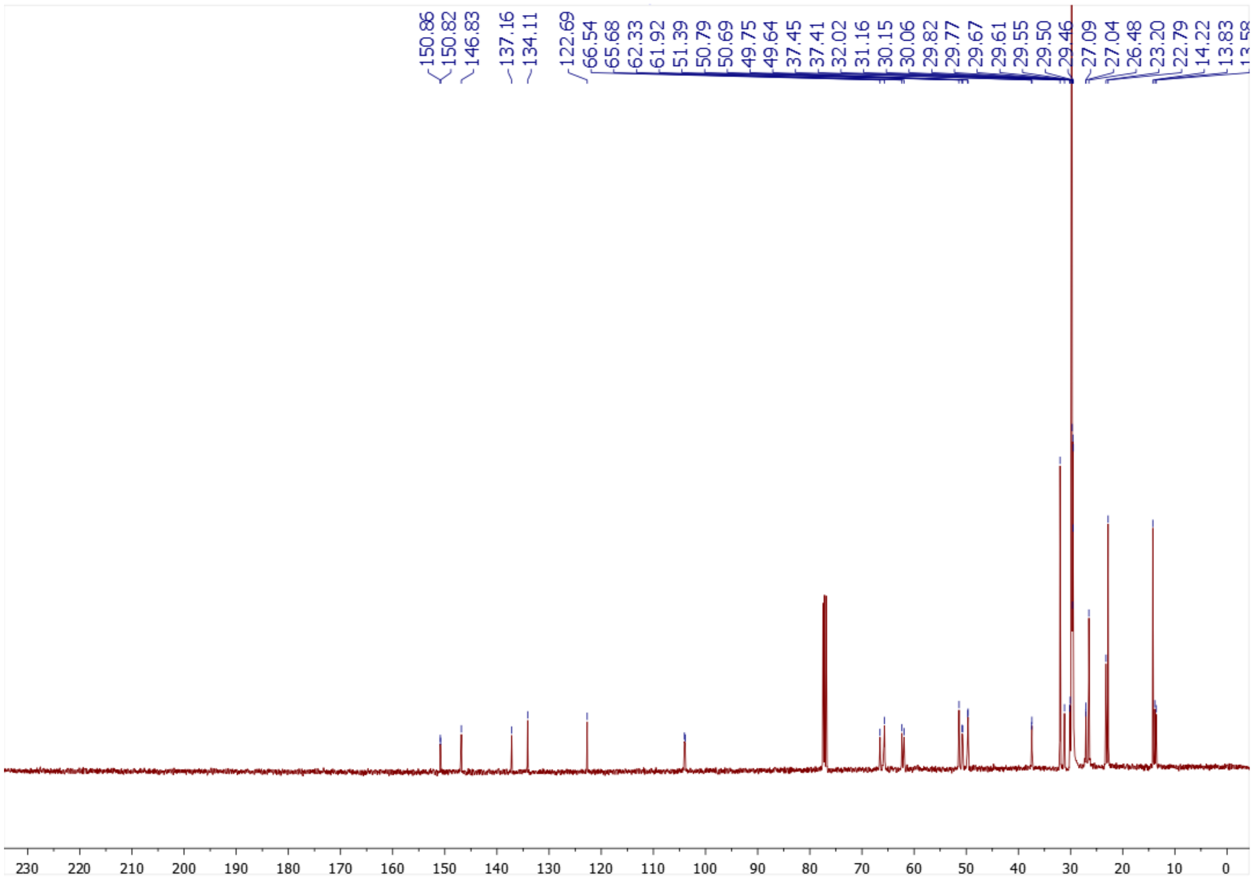

^{13}C{H} NMR spectrum of compound **5p**$_{18}$

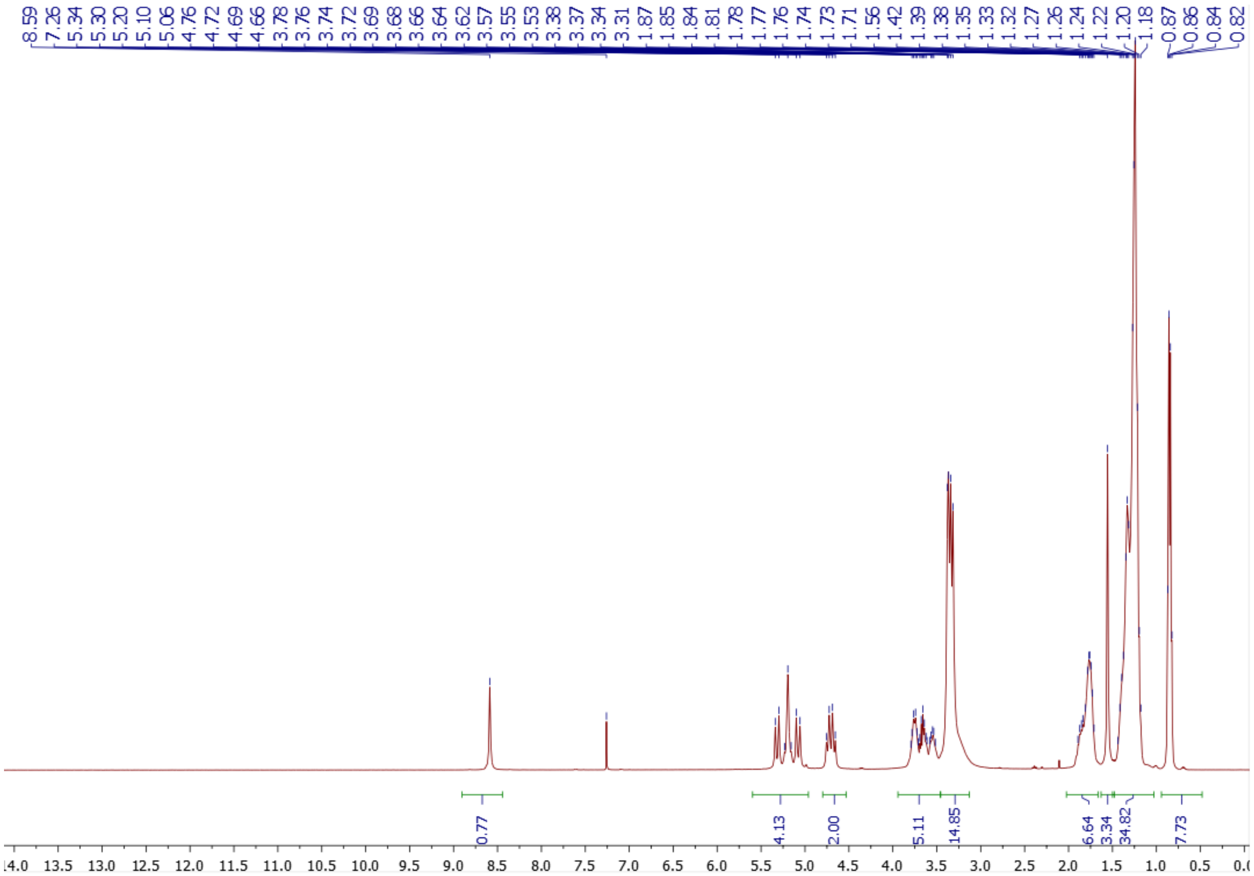

^{1}H NMR spectrum of compound **5q$_{8}$**

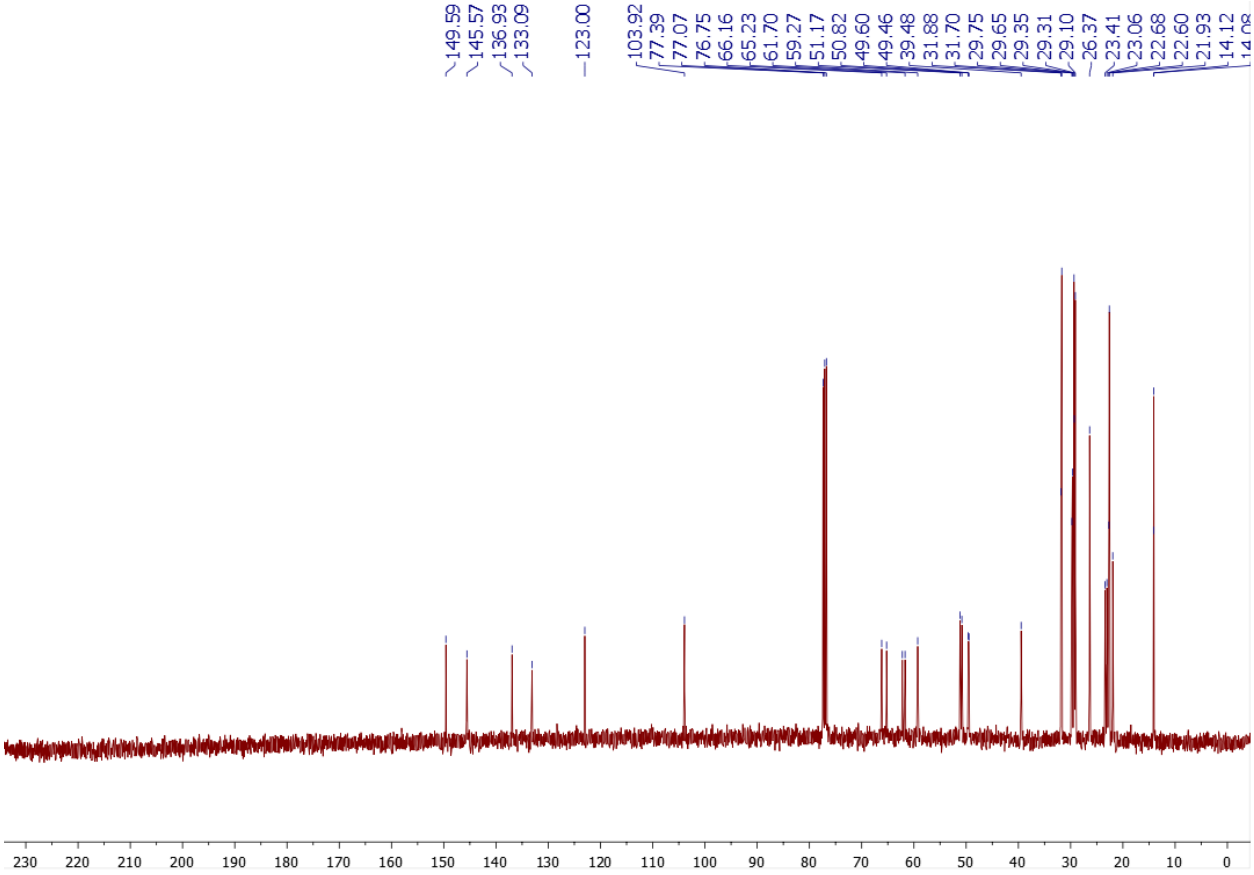

^{13}C{H} NMR spectrum of compound **5q$_{8}$**

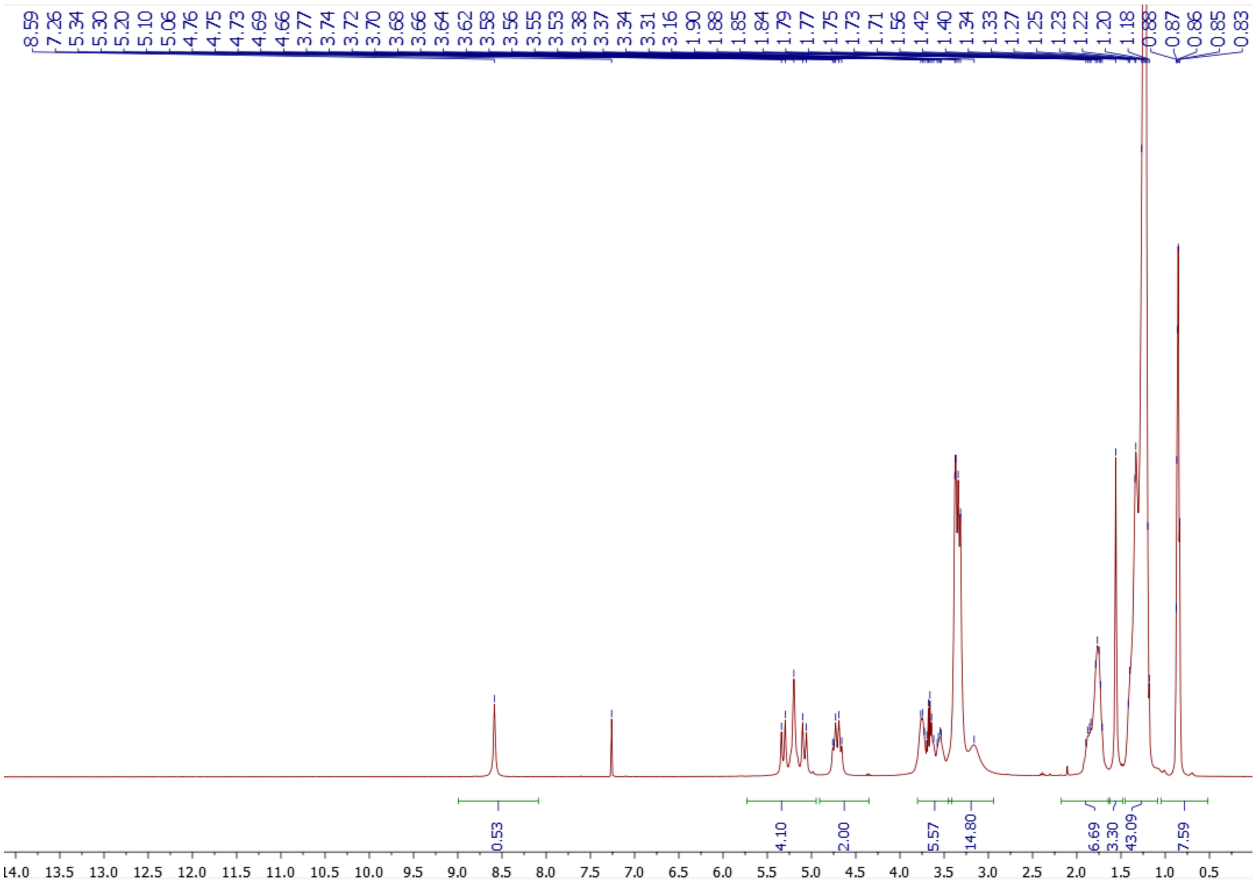

^{1}H NMR spectrum of compound **5q10**

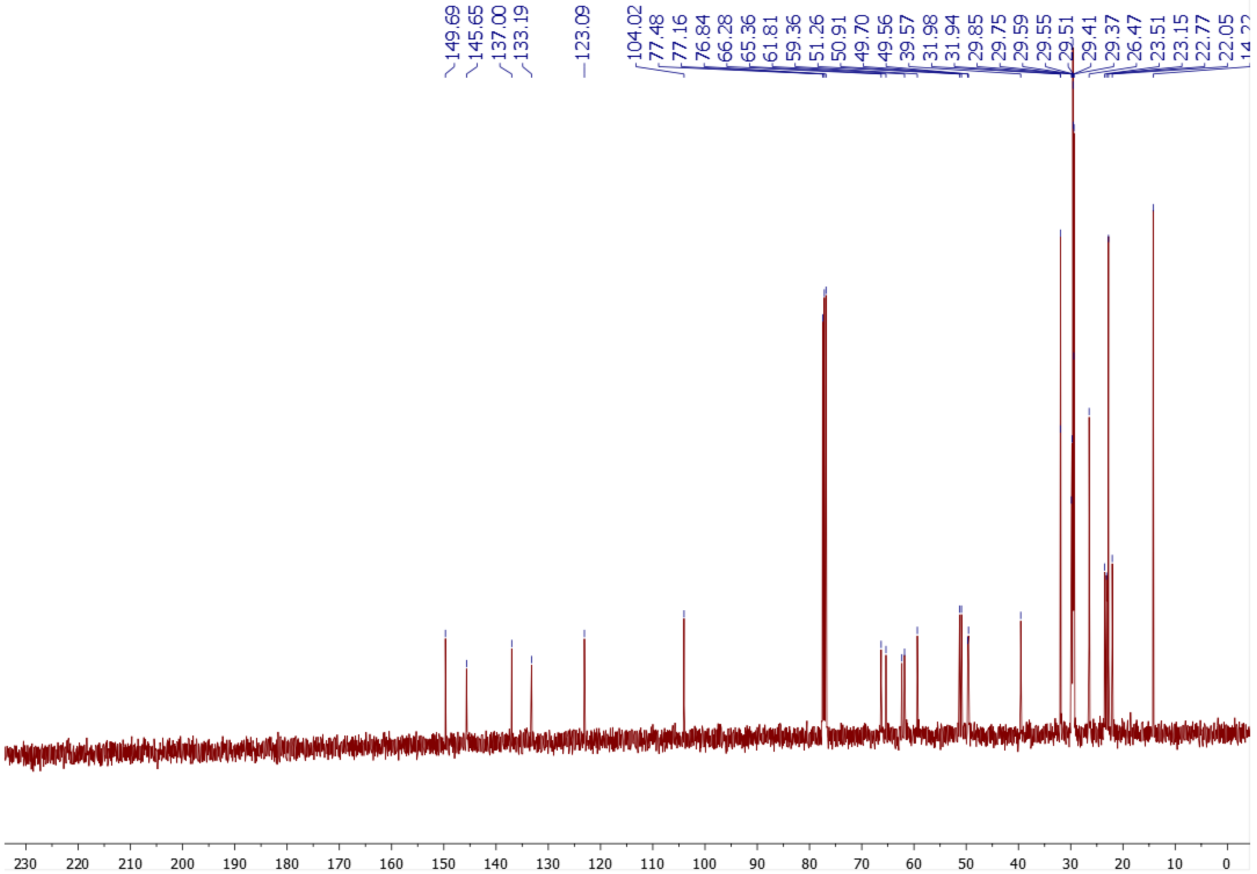

^{13}C{H} NMR spectrum of compound **5q10**

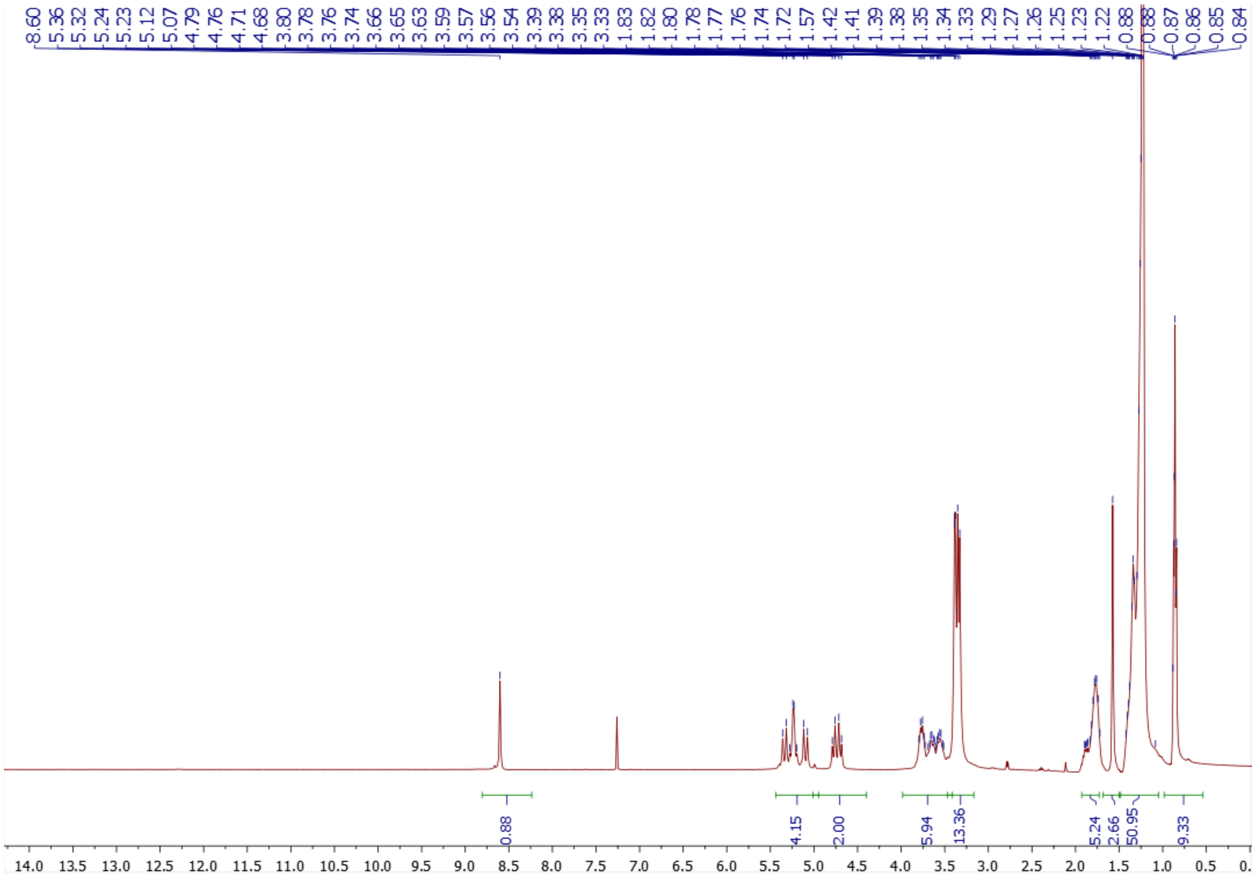

^{1}H NMR spectrum of compound **5q**$_{12}$

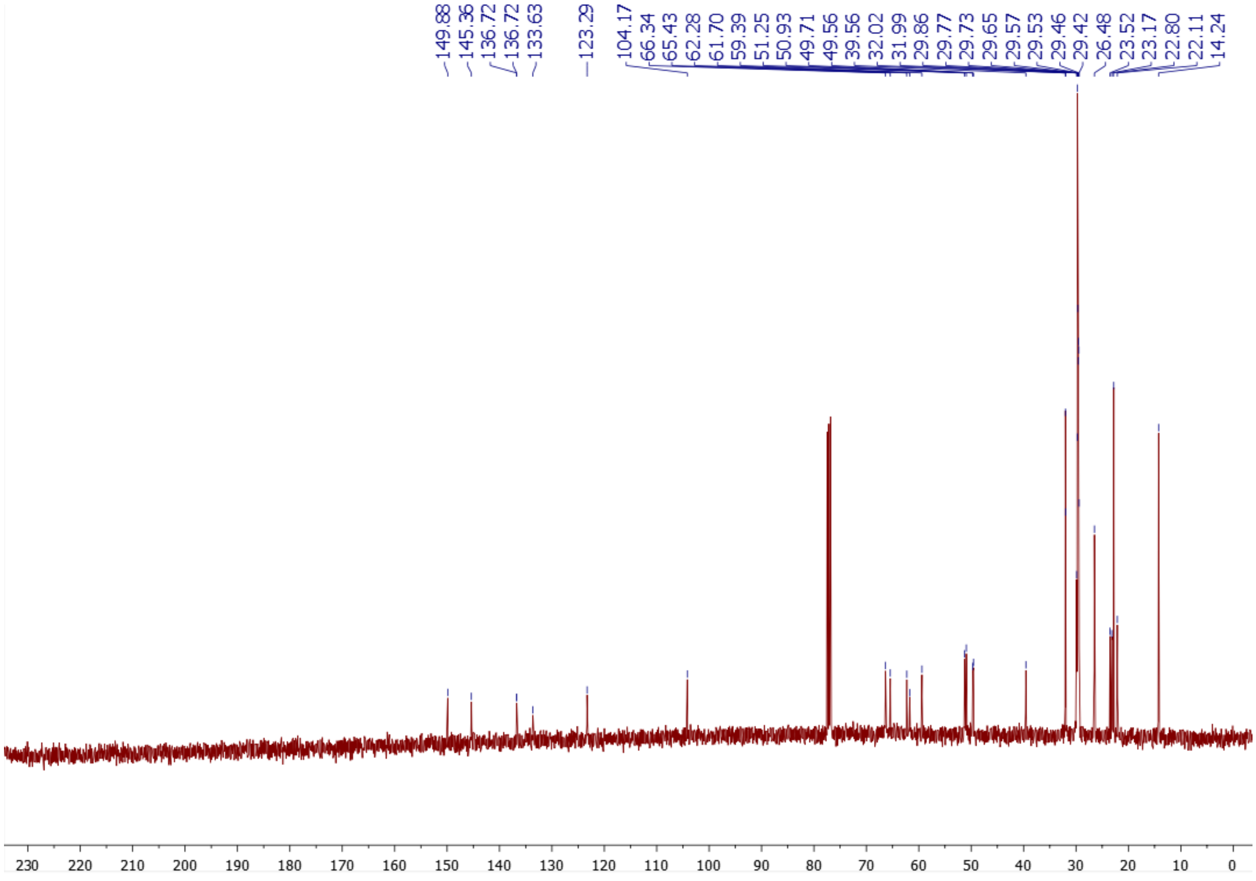

^{13}C{H} NMR spectrum of compound **5q**$_{12}$

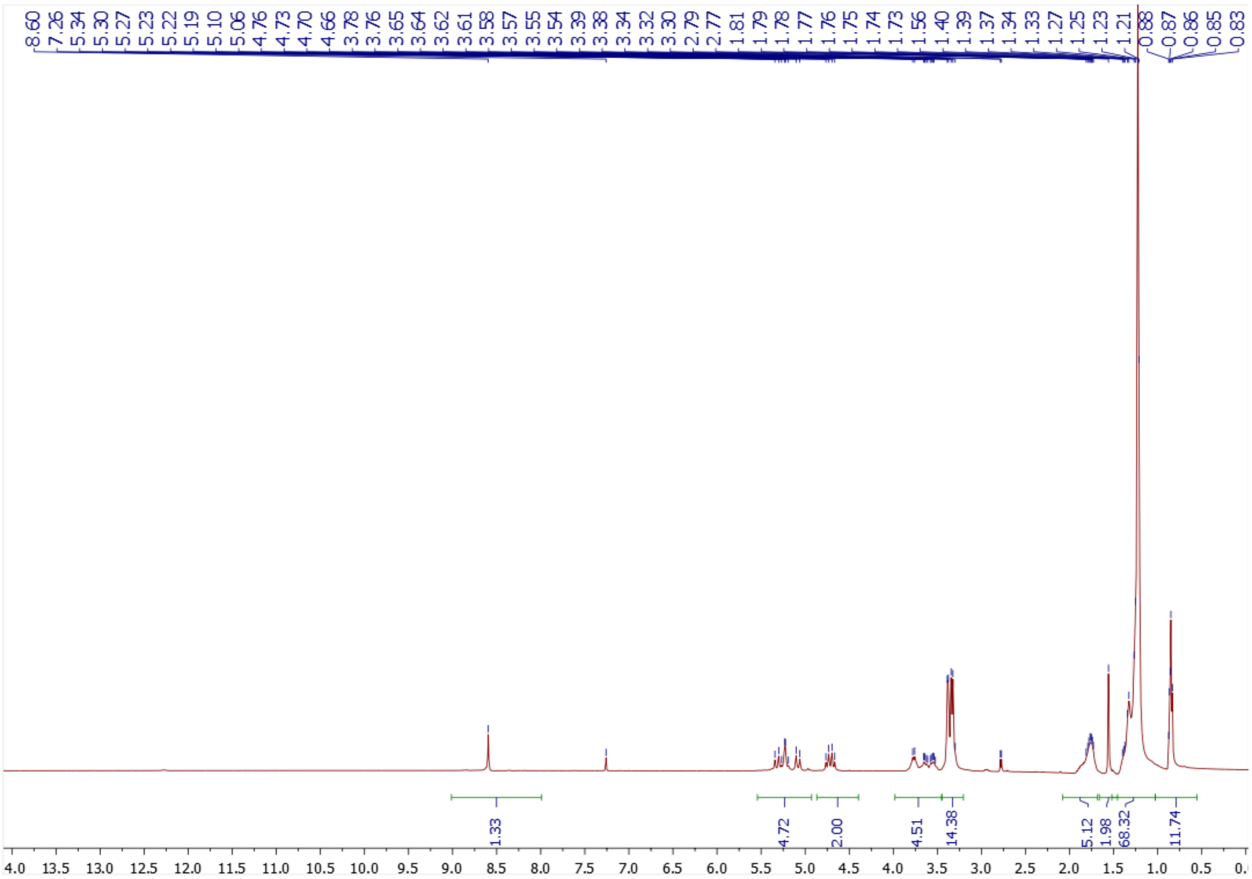

^{1}H NMR spectrum of compound **5q**14

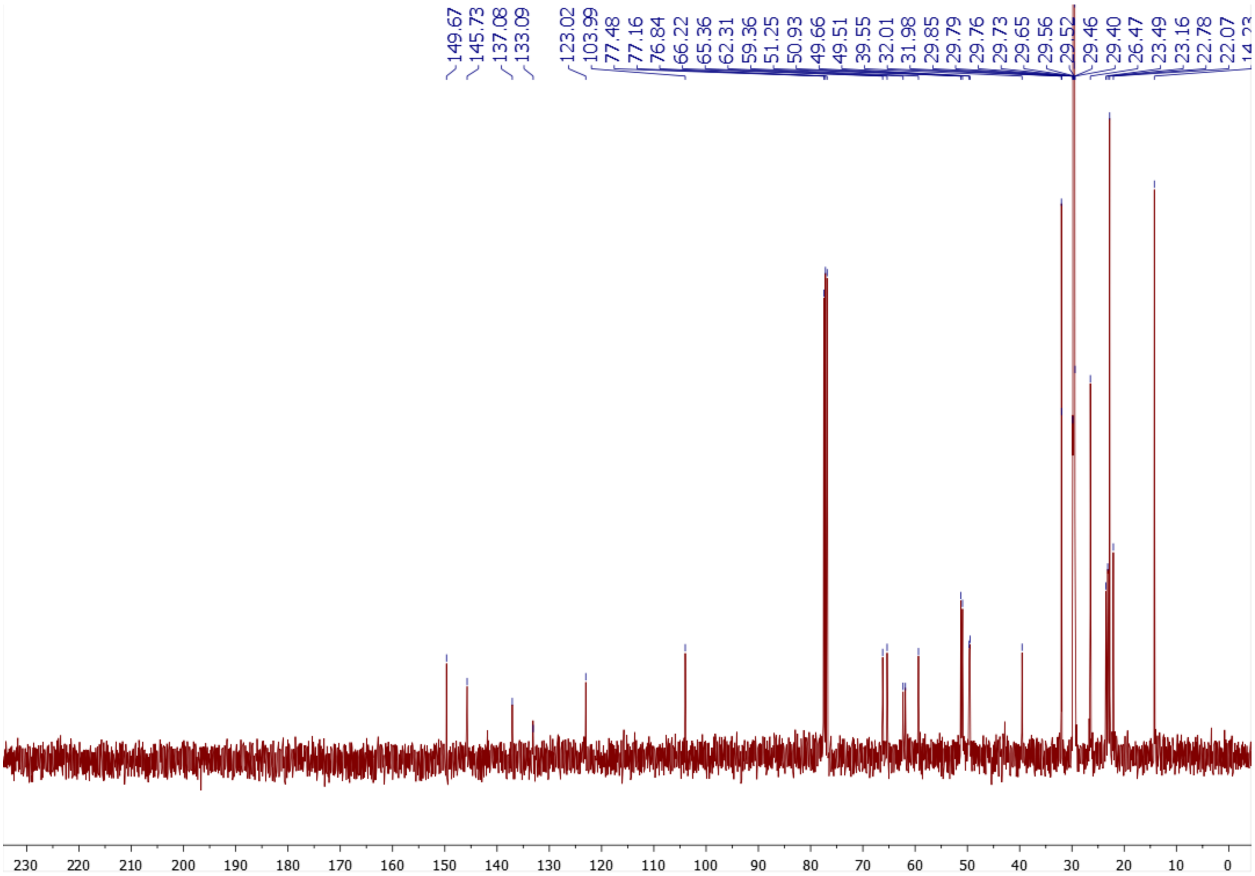

$^{13}C\{H\}$ NMR spectrum of compound **5q**14

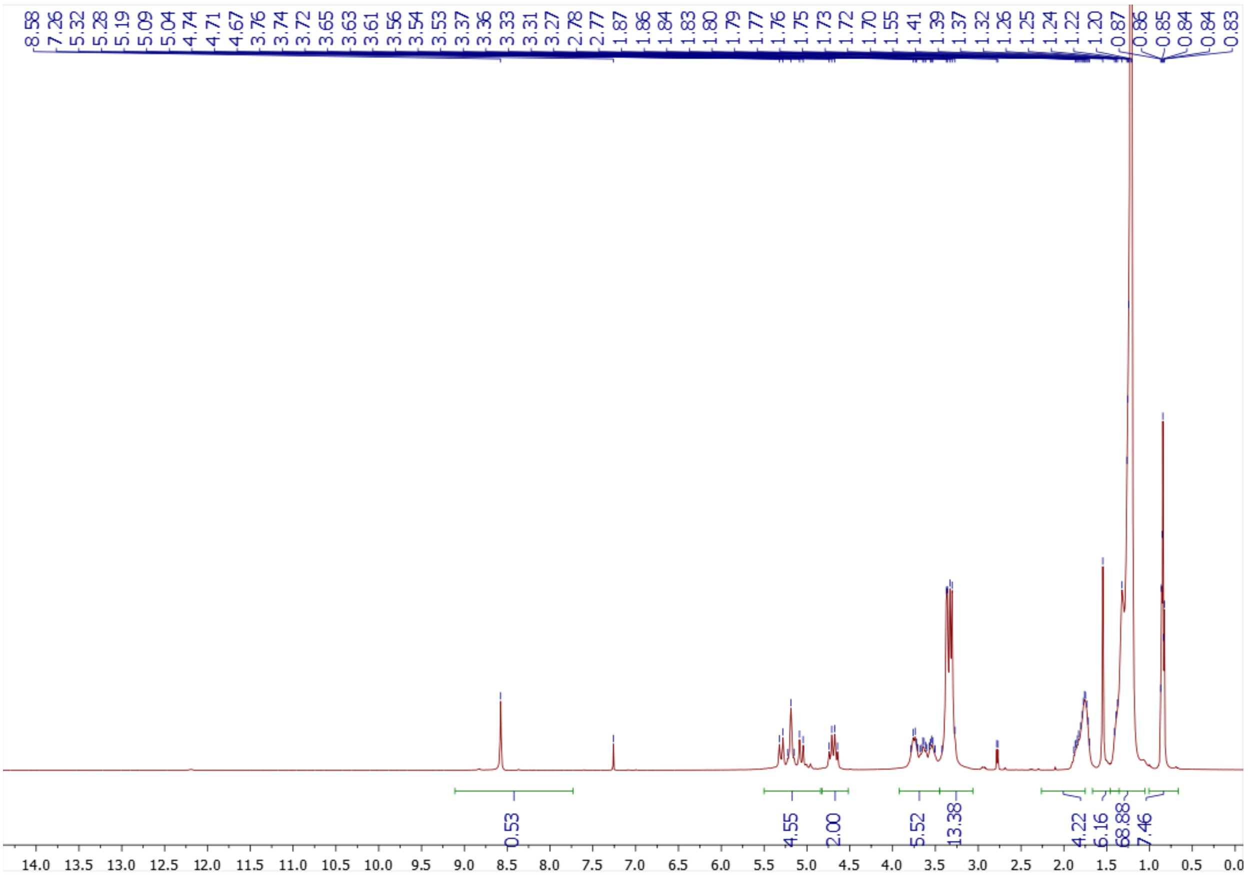

^{1}H NMR spectrum of compound **5q**16

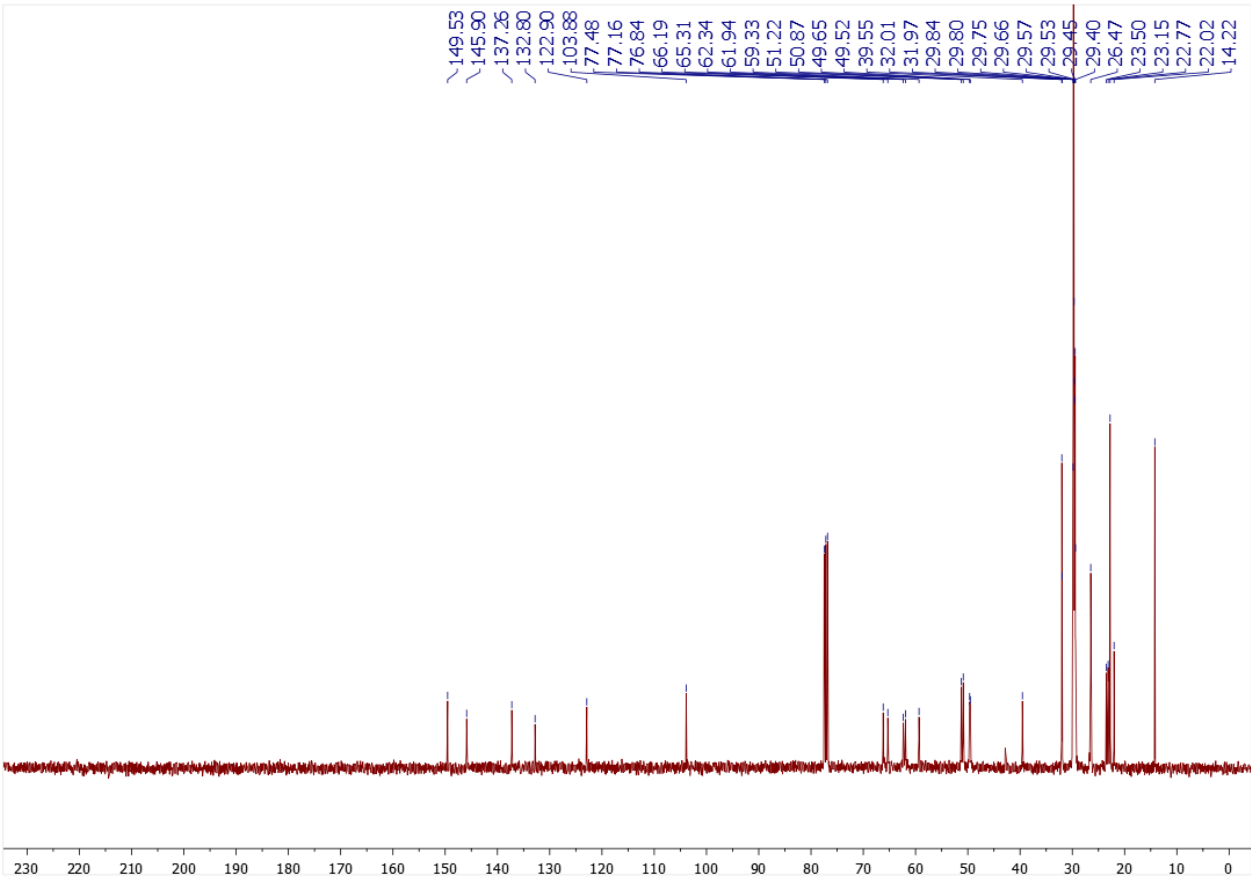

^{13}C{H} NMR spectrum of compound **5q**16

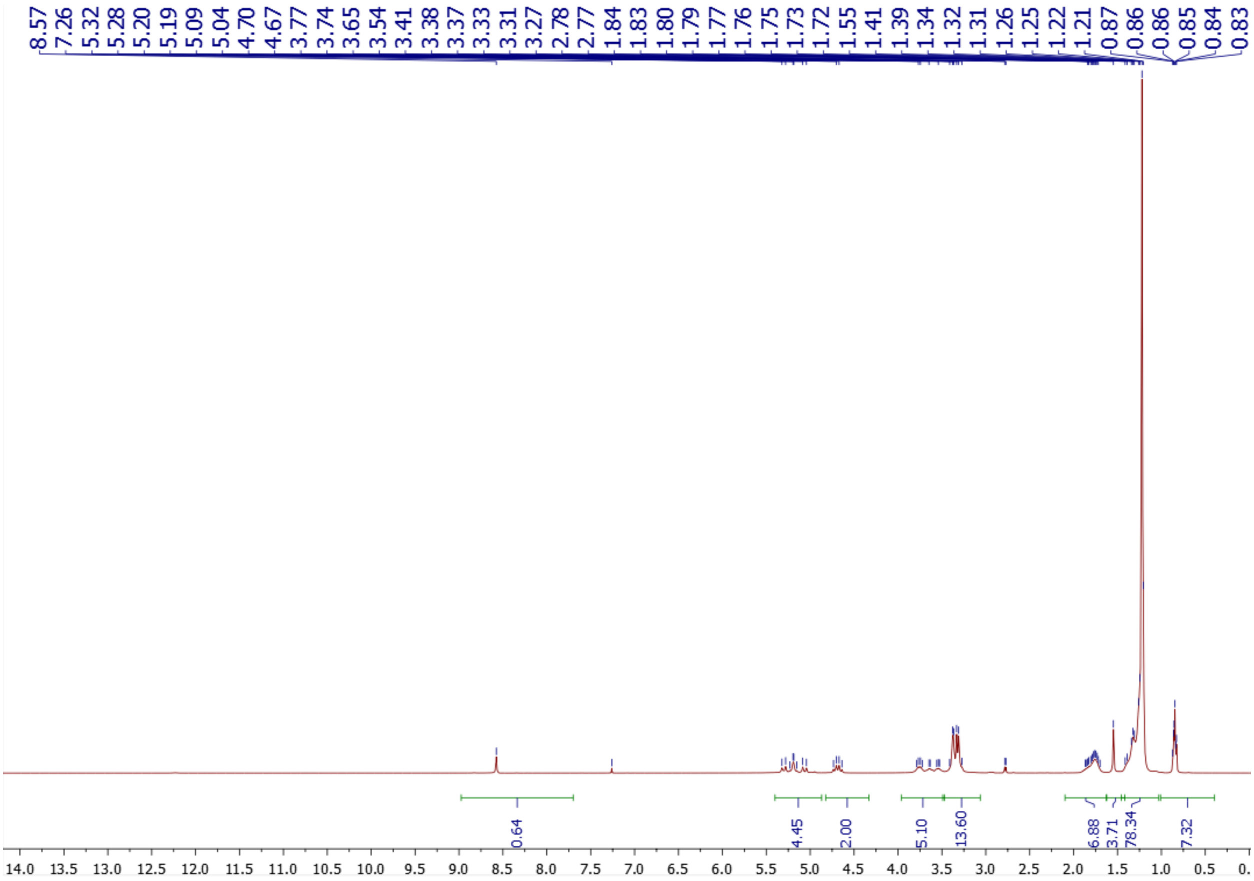

^{1}H NMR spectrum of compound **5q**18

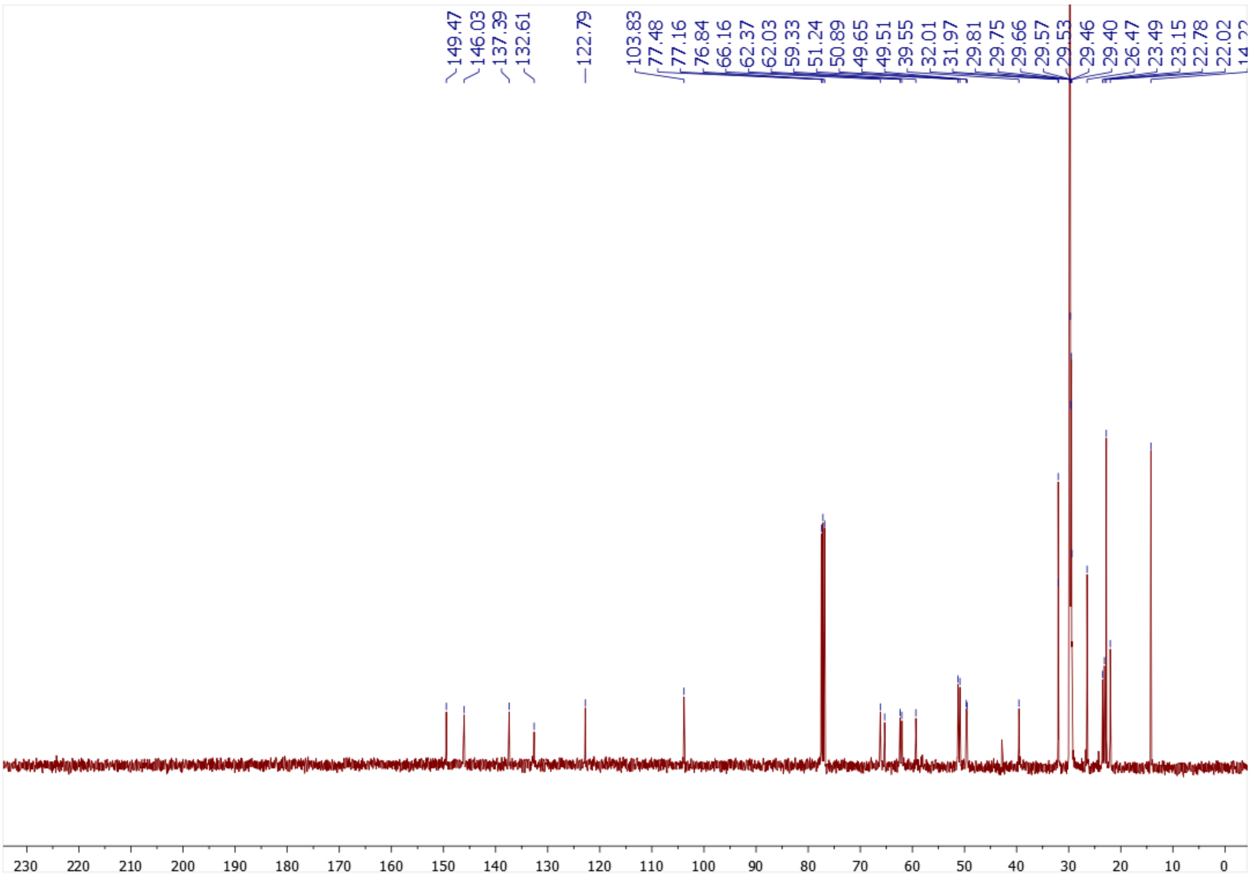

^{13}C{H} NMR spectrum of compound **5q**18

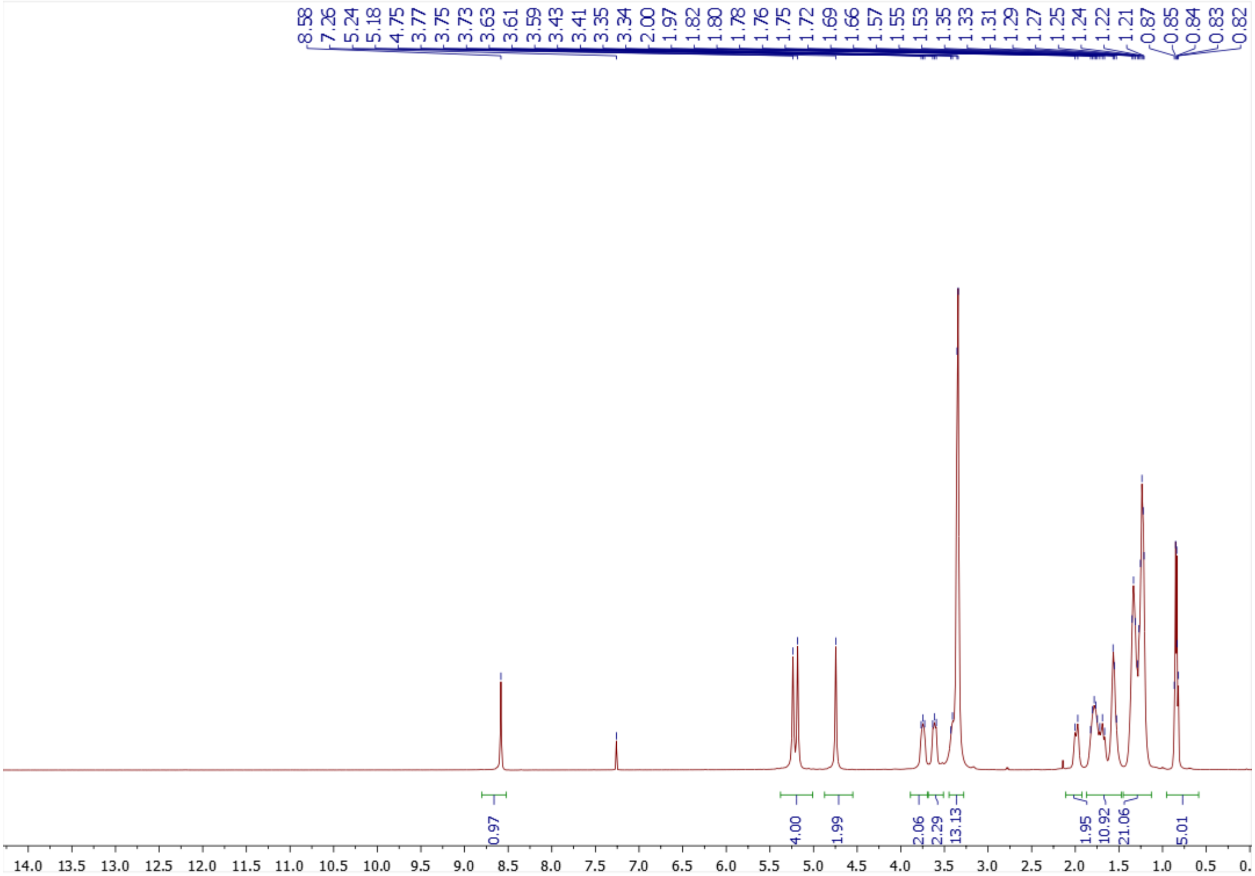

^{1}H NMR spectrum of compound **5r**8

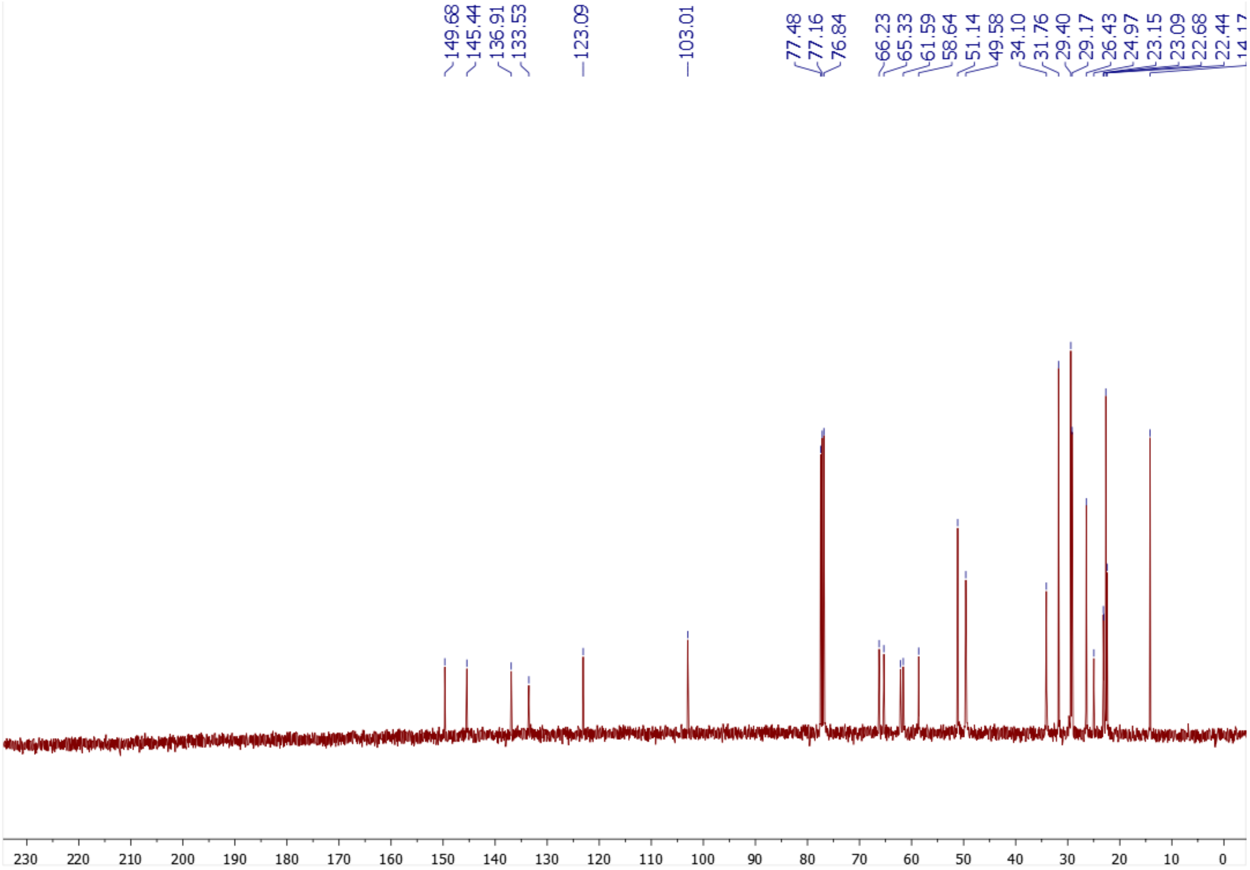

^{13}C{H} NMR spectrum of compound **5r**8

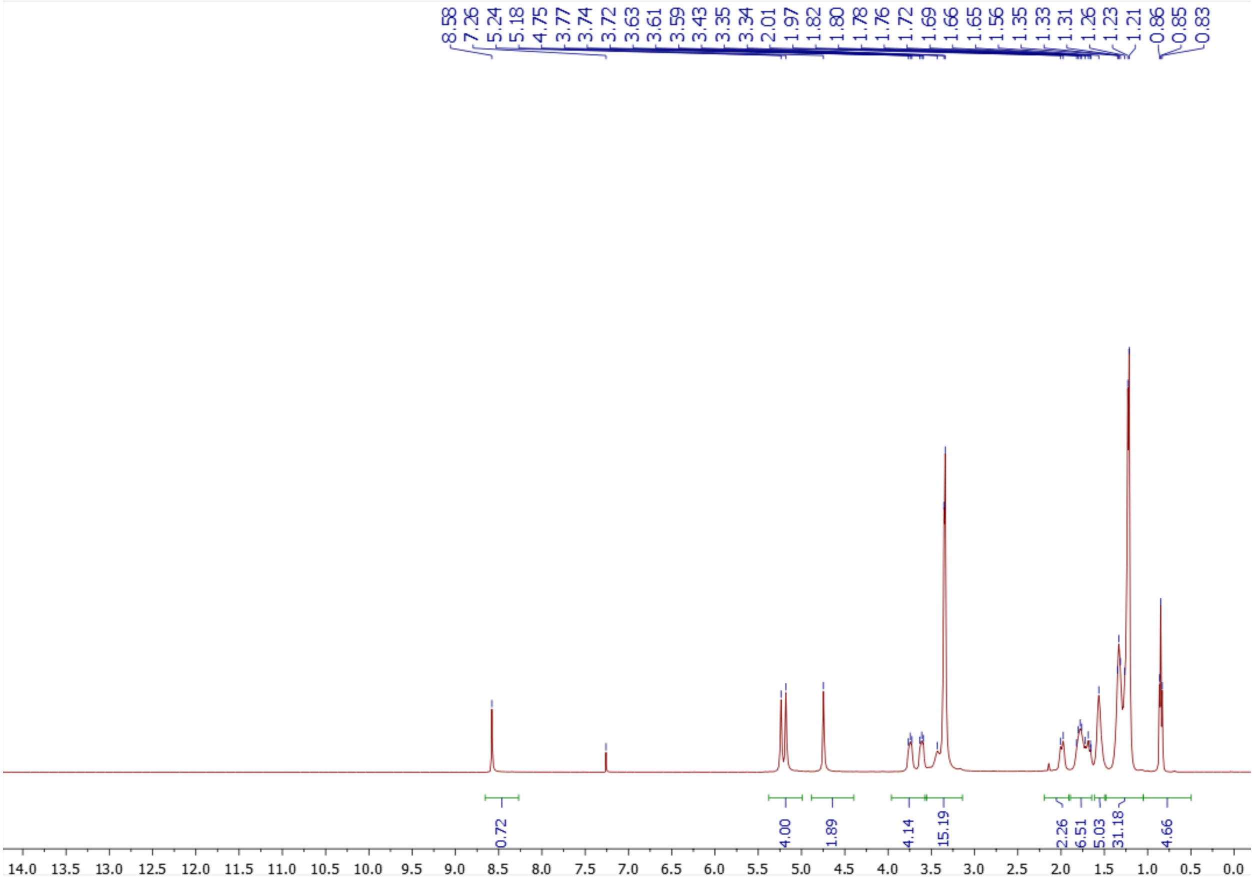

^{1}H NMR spectrum of compound **5r₁₀**

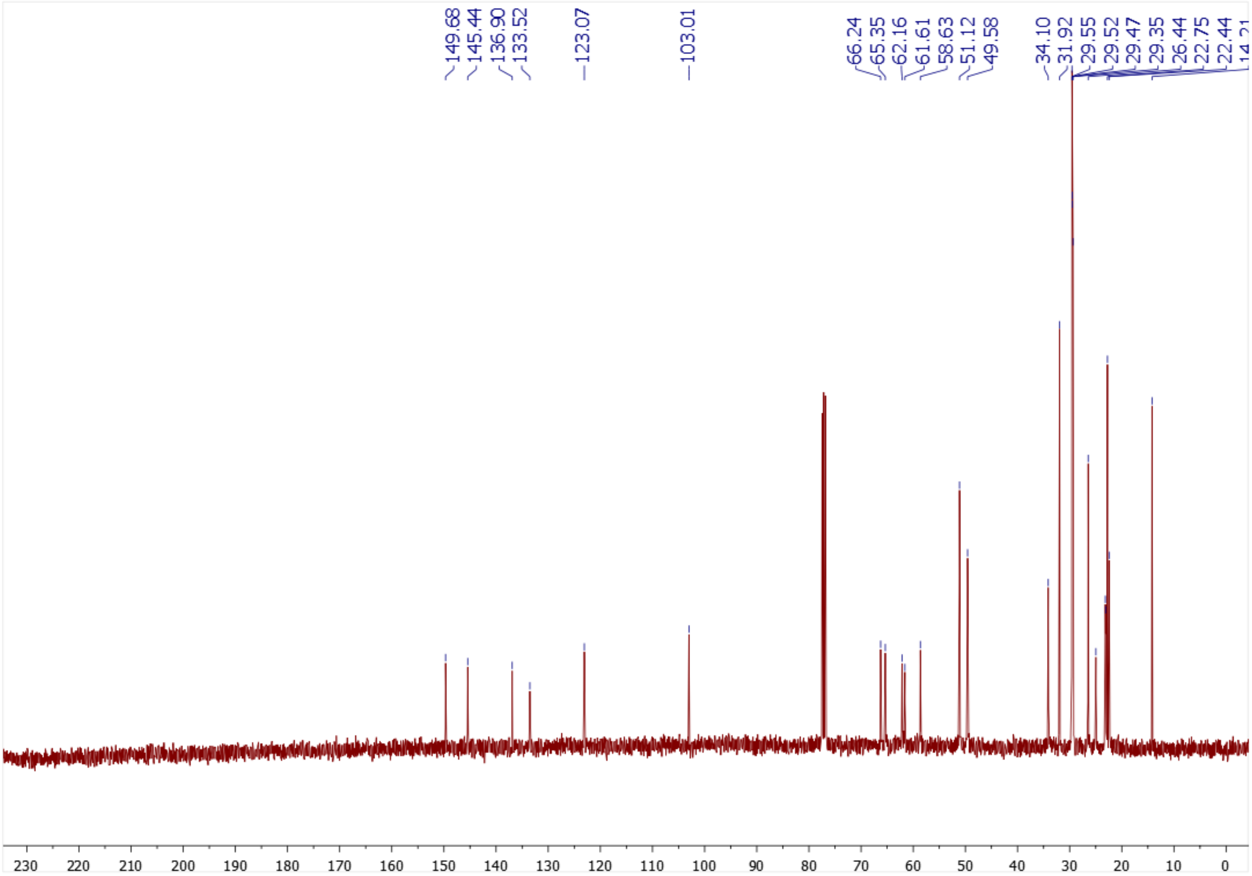

$^{13}C\{H\}$ NMR spectrum of compound **5r₁₀**

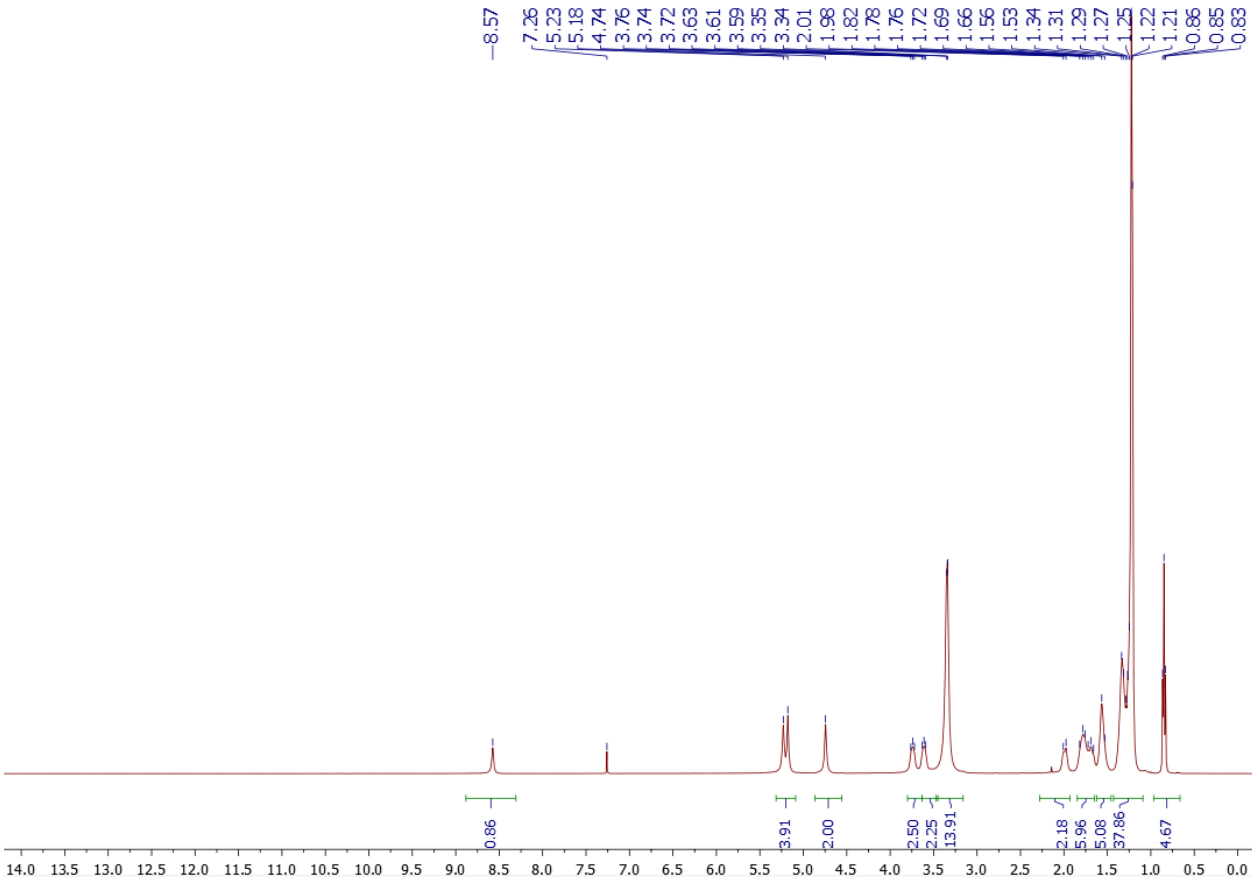

^{1}H NMR spectrum of compound **5r**12

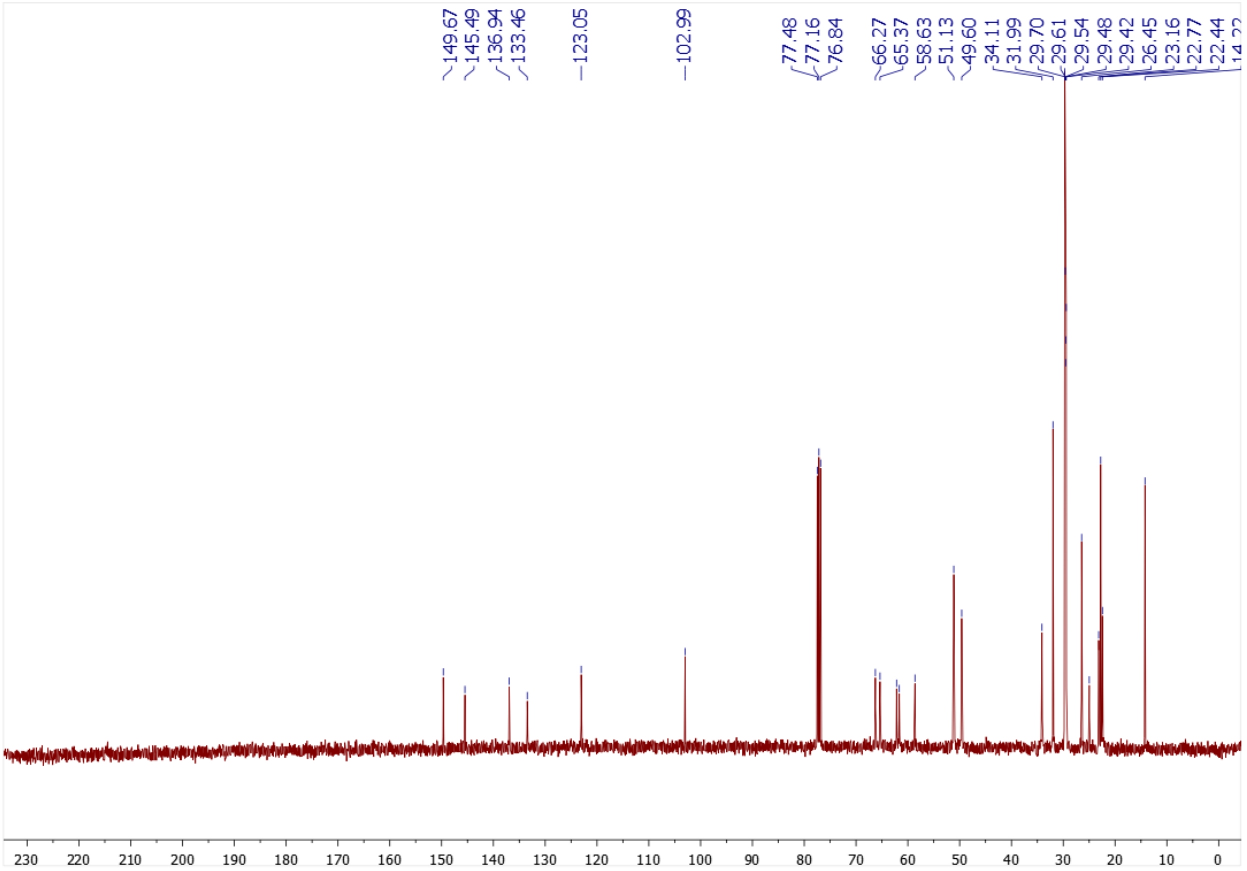

^{13}C{H} NMR spectrum of compound **5r**12

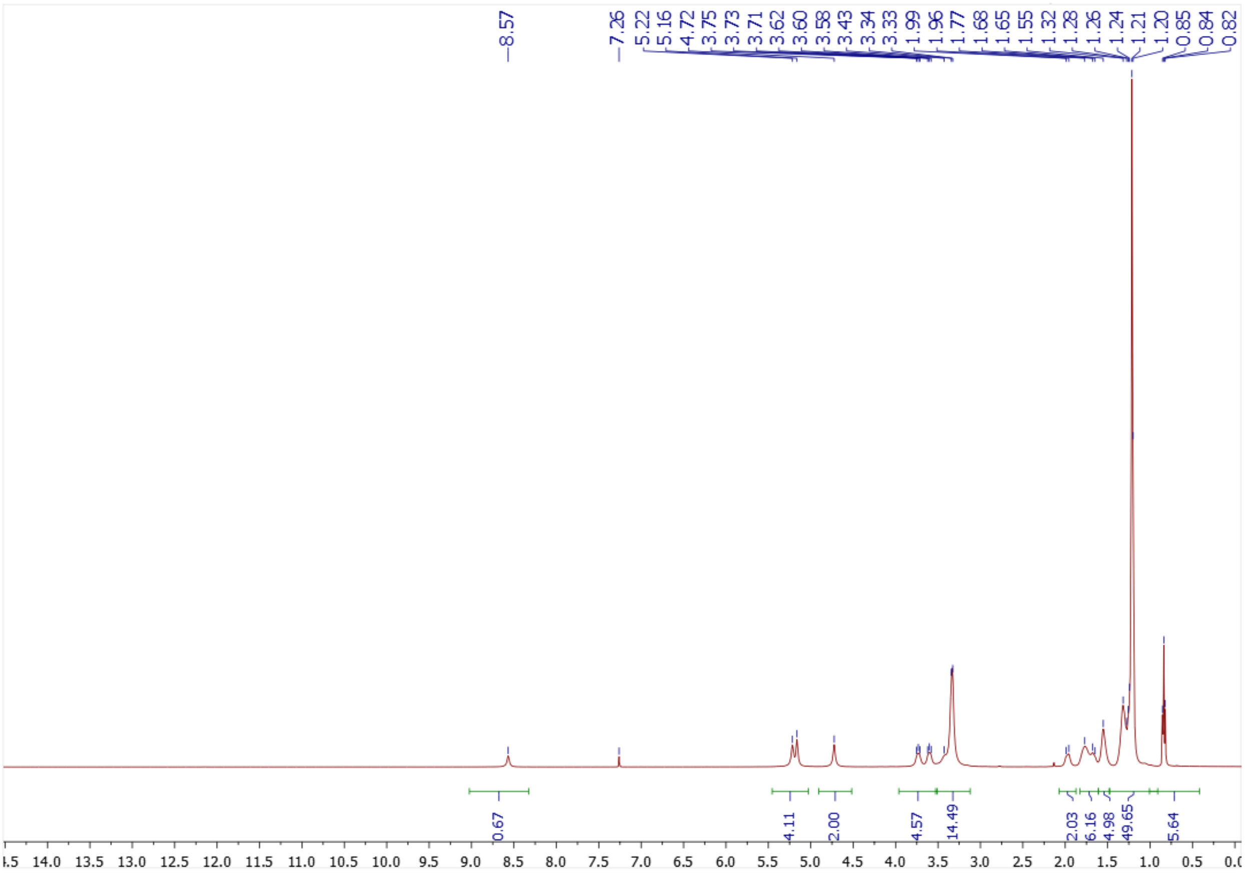

^{1}H NMR spectrum of compound **5r**$_{14}$

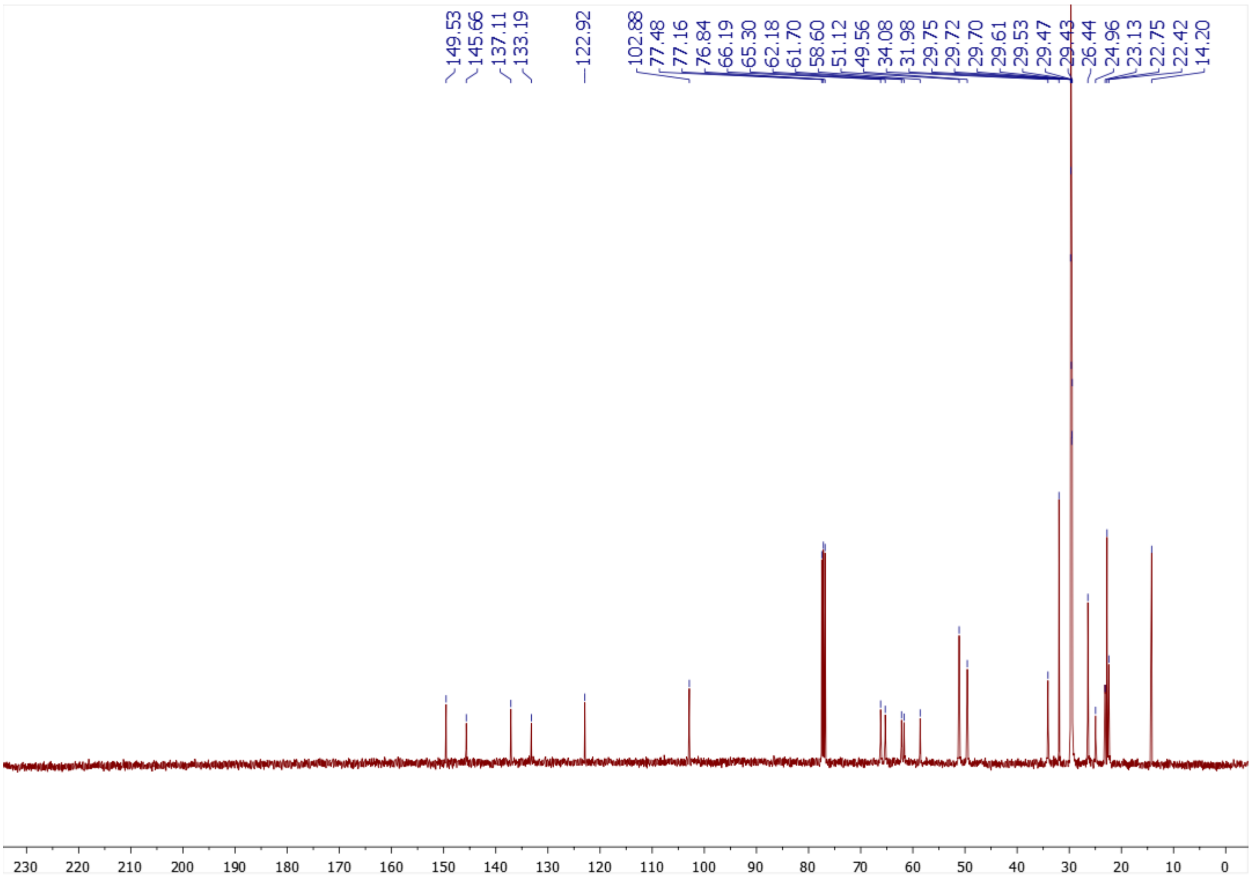

^{13}C{H} NMR spectrum of compound **5r**$_{14}$

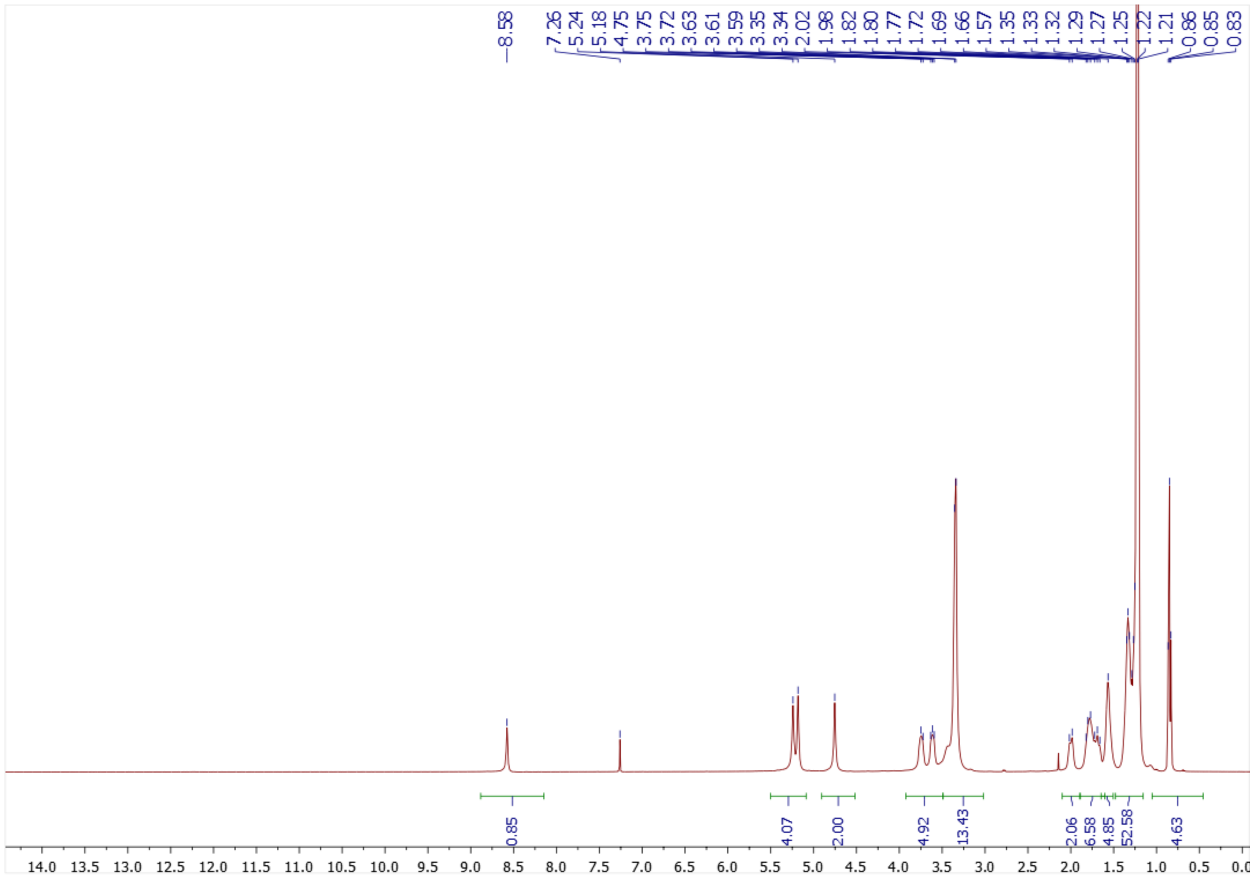

^{1}H NMR spectrum of compound **5r$_{16}$**

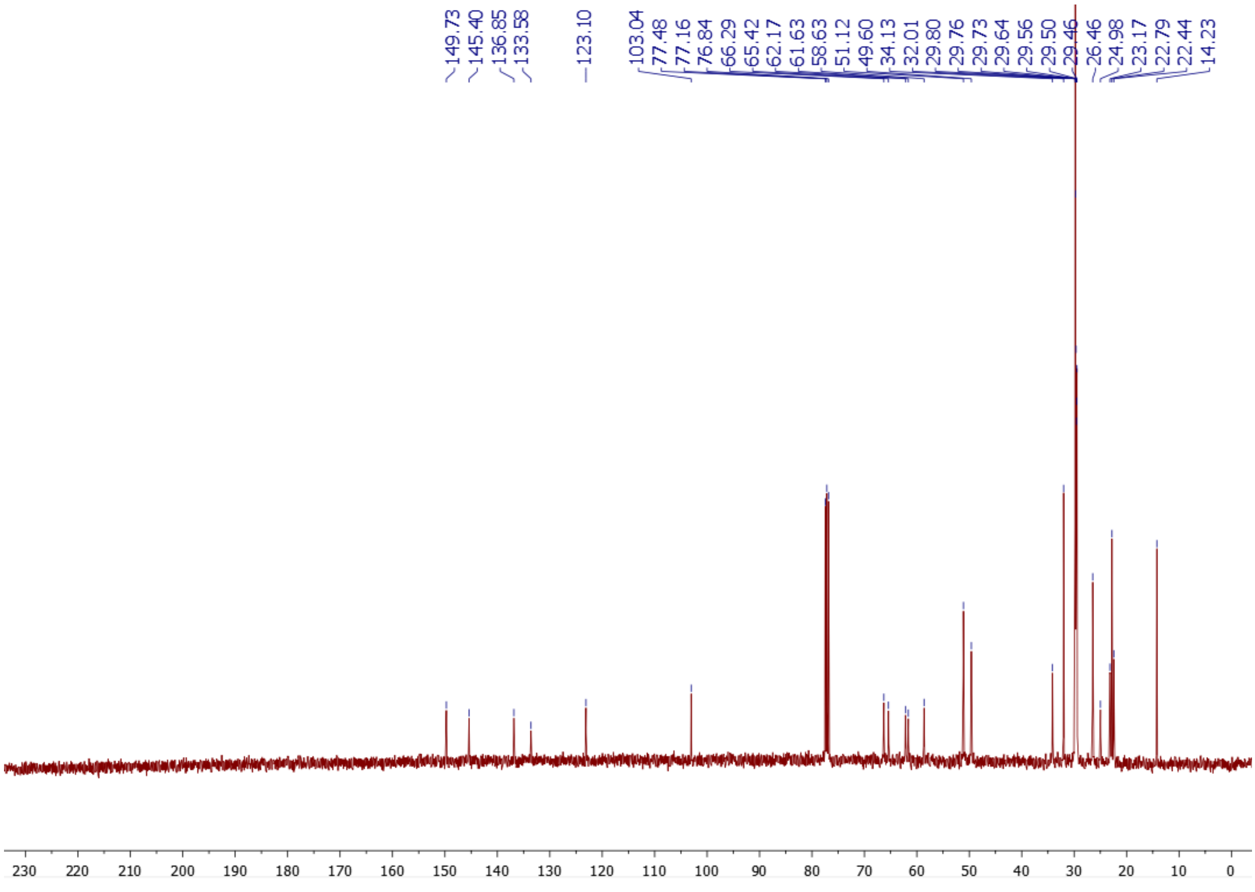

^{13}C{H} NMR spectrum of compound **5r$_{16}$**

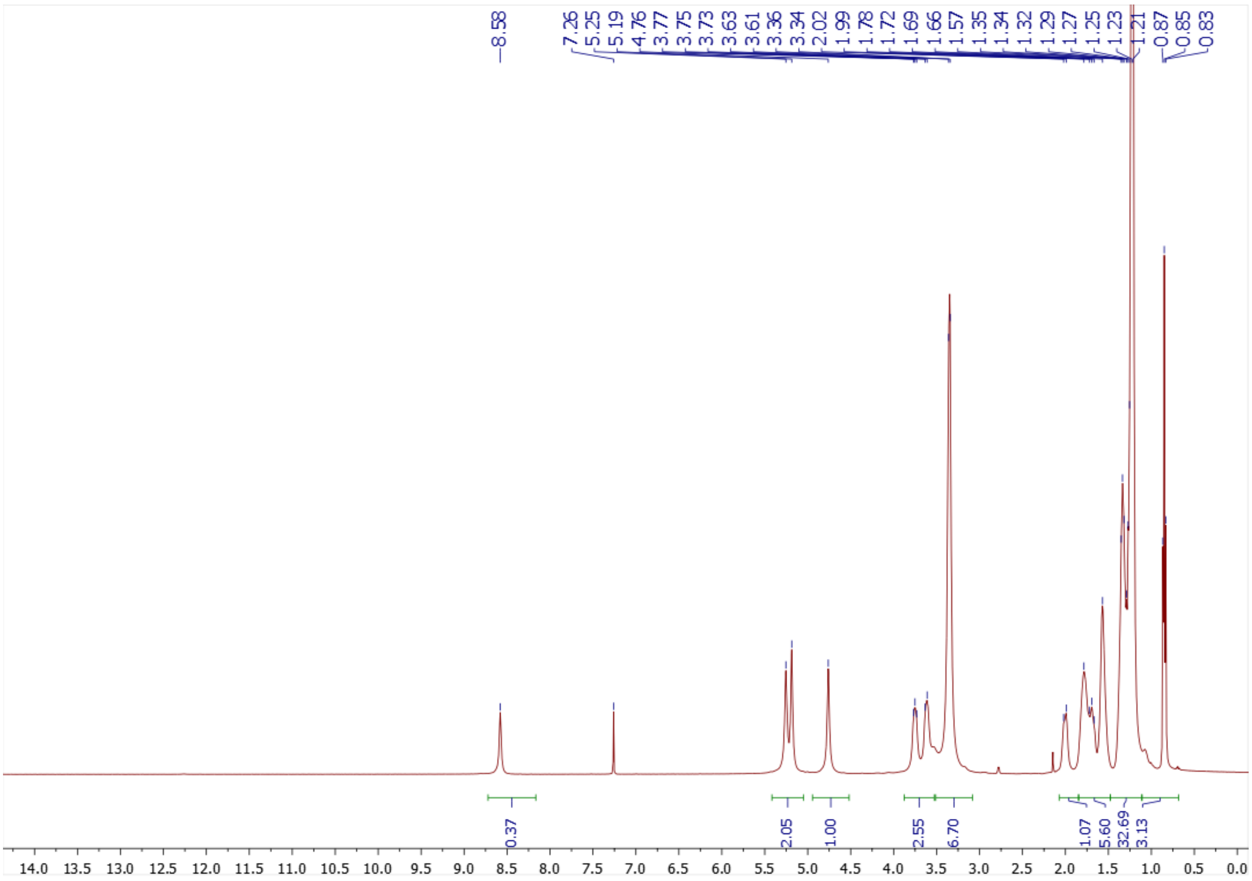

^{1}H NMR spectrum of compound **5r**$_{18}$

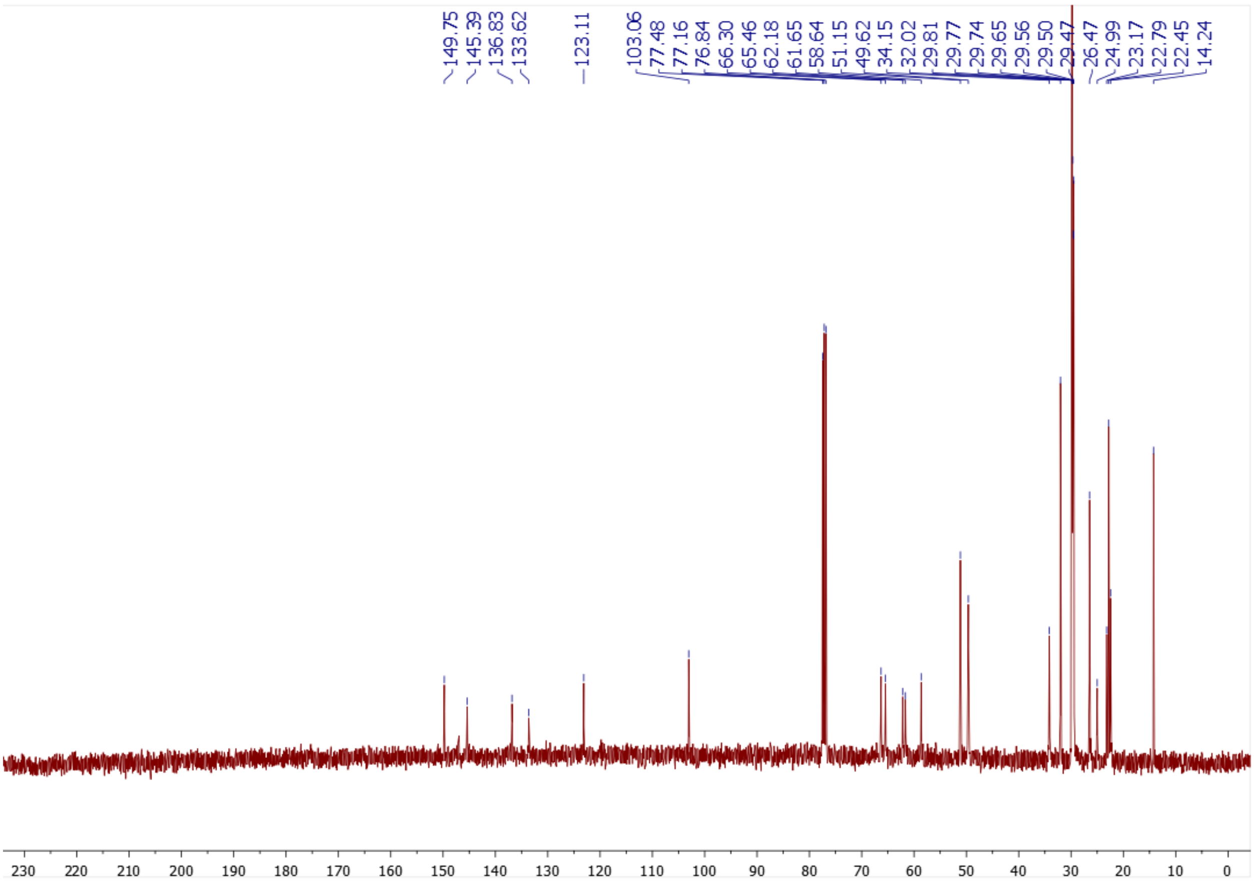

^{13}C{H} NMR spectrum of compound **5r**$_{18}$
